# Bottom-up modular synthesis of well-defined oligo(arylfuran)s

**DOI:** 10.1038/s41467-021-26387-5

**Published:** 2021-10-25

**Authors:** Yang Chen, Pingchuan Shen, Tongxiang Cao, Hao Chen, Zujin Zhao, Shifa Zhu

**Affiliations:** 1 Key Laboratory of Functional Molecular Engineering of Guangdong Province, School of Chemistry and Chemical Engineering, South China University of Technology, Guangzhou, 510640 China; 2 State Key Laboratory of Luminescent Materials and Devices, Guangdong Provincial Key Laboratory of Luminescence from Molecular Aggregates, South China University of Technology, Guangzhou, 510640 China; 3 Guangdong Youmei Institute of Intelligent Bio-manufacturing Co., Ltd, Guangzhou, China

**Keywords:** Organic molecules in materials science, Synthetic chemistry methodology, Single-molecule fluorescence

## Abstract

Oligofurans have attracted great attention in the field of materials over the last decades because of their several advantages, such as strong fluorescence, charge delocalization, and increased solubility. Although unsubstituted or alkyl-substituted oligofurans have been well-established, there is an increasing demand for the development of the aryl decorated oligofuran with structural diversity and unrevealed properties. Here, we report the bottom-up modular construction of chemically and structurally well-defined oligo(arylfuran)s by de novo synthesis of α,β′-bifuran monomers and late-stage bromination, stannylation and subsequent coupling reaction. The preliminary study of the photophysical properties demonstrated that the polarity-sensitive fluorescence emission and high quantum yields in THF solution could be achieved by modulating the aryl groups on the oligo(arylfuran)s. These twisted molecules constitute a new class of oligofuran backbone useful for structure−activities relationship studies. Meanwhile, the experimental studies and calculations showed that tetrafurans have appropriate HOMO energy levels, and could therefore potentially be high-performance *p*-type semiconductors.

## Abstract (web summary)

Oligofurans have attracted great attention because of their strong fluorescence, charge delocalization, and increased solubility. Here the authors show a bottom-up modular construction of chemically and structurally well-defined oligo(arylfuran)s by de novo synthesis of α,β′-bifuran monomers and late-stage bromination, stannylation and subsequent coupling reaction.

## Introduction

**Fig. 1:**
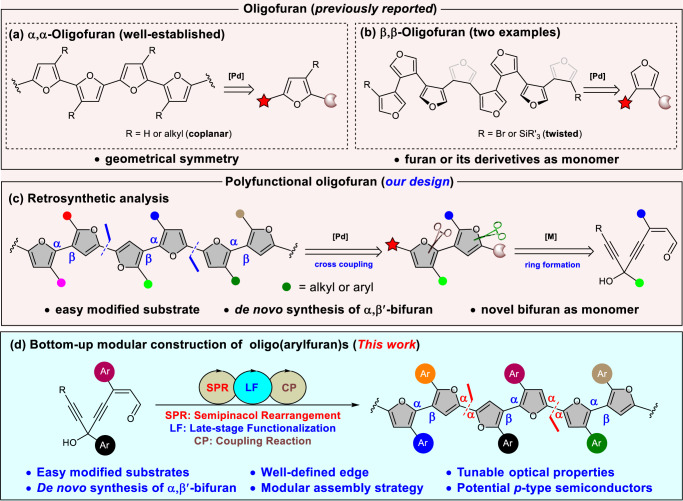
Development of oligofurans. **a** α,α-Oligofurans. **b** β,β-Oligofurans. **c** Retrosynthetic analysis of polyfunctional oligofuran. **d** This work: bottom-up modular synthesis of oligo(arylfuran)s.

The development of operationally straightforward and precise-effective routes for the assembly of heterocycles from simple starting materials is very important for many scientific endeavors. Furan is one of the most important five-membered heterocycles, which have been found to have widespread applications in bioactive pharmaceuticals^1–3^, agrochemicals^4^, and functional materials^5,6^. Furthermore, the non-fused linear oligomers of furan (oligofurans; nF), can be employed as useful building block in synthetic organic chemistry^7,8^ as well as a potential skeleton in materials science^9–11^. In addition, oligofurans were regarded as “green” materials, as furan can be directly obtained from renewable bioresources^12^ and are easily biodegradable^13^. However, compared to the thio-analog, oligothiophenes (nTs), which were considered among the workhorses in the field of organic electronic materials^14^, nF have been overlooked for a long time^15–21^. A decade ago, researchers have already demonstrated that oligofurans exhibited higher fluorescence, better solubility, greater rigidity, and tighter solid-state packing than the corresponding nTs. These promising advantages have spurred massive efforts toward the design and synthesis of novel oligofuran-based materials during the past decade. For example, in 1981 Kauffmann et al.^15^ have achieved the α,α-tetrafuran using the Ullmann homocoupling reaction, and about 20 years later the substituted α,α-pentafuran was reported by the same group through Negishi coupling reaction^16^. In 2010, Bendikov and colleagues^17^ realized the synthesis of the α,α-nonafuran via Stille coupling. The longest α,α-oligofuran (16F-2C_6_) bearing with adjacent C6-alkyl substitution was synthesized by the coupling of soluble furan oligomers (Fig. 1a)^18^. In addition, a unique β-substituted α,α-oligofuran (4F) was synthesized by Zhang and colleagues^19^ via an elegant iterative radical tandem cyclization mediated by a Co (II)-based metalloradical catalyst. Interestingly, it is also about 20 years later from the seminal synthesis of the β,β-oligofuran (8F, *R* = SiR'_3_) by Song and Wong^20^, an iterative cross-coupling synthesis of similar oligofuran (8F, *R* = Br) was achieved and revealed its 3D conformation by Paddon-Row and colleagues^21^ (Fig. 1b). Despite of these achievements in α,α-oligofurans and β,β-oligofurans, the preparation of polyfunctional oligofurans is still challenging.

As Bendikov and colleagues^18^ observed in the 16F-6C_6_ and nF-2C_6_, the adjacent alkyl substituents on the C3 position have no impact on the planarity of the backbone. What’s more, previous study has shown that the slightly twisted conformations of nTs might also provide for favorable electronic communication^22^. Therefore, it is reasonable to postulate that substitution of alkyl substituents with more sterically hindered aryl groups might result in twisted oligofurans as well. Importantly, the incorporation of aryl groups provides a powerful means in modulating the properties of the oligofurans via conjugation effect of arene^23,24^. However, the limited accessibility of versatile 2,3-difunctionalized and 2,4-difunctionalized furans with suitable coupling handles and the potential competition of α-C-H bond of furan during cross-coupling severely impeded efficient synthesis of aryl-decorated oligofurans^20,25^. To the best of our knowledge, there is no successful report on the design and synthesis of polyaryl-substituted oligofurans. In this regard, developing an efficient and reliable synthetic strategy to assemble oligo(arylfuran)s would be challenging but highly desirable.

Recently, transition metal-catalyzed cycloisomerization of allenyl ketones/enynones/enynals has become a straightforward method for the synthesis of functionalized furans^26–31^. Based on our previous work in this area^32–37^ and also inspired by metal carbene-involved semi-pinacol rearrangement^38–44^, we envisioned that the simple and commercially inexpensive starting material enynals might undergo a tandem cycloisomerization/selective alkynyl rearrangement/cycloisomerization reaction to furnish the desired α,β′-bifuran as a monomer, which not only avoid the direct formation of the α,β-connective bond but also improve the efficiency of oligofurans assembly (Fig. 1c).

In this work, we report the realization of this design of bottom-up modular construction of chemically and structurally well-defined oligo(arylfuran)s by de novo synthesis of α,β′-bifuran monomers and late-stage bromination, stannylation, and subsequent coupling reaction (Fig. 1d). This strategy allows the selective and straightforward introduction of different aryl groups into each furan unit of the oligofurans without otherwise tedious process for the introduction of aryl substitution. Meanwhile, the experimental studies and calculations show that tetrafurans have appropriate highest occupied molecule orbital (HOMO) energy levels and could therefore potentially be high-performance *p*-type semiconductors.

## Results and discussion

### Optimization study

Our initial tests were set out by using aryl-enriched enynal **1-1** as the substrate for reaction condition screening. As shown in Table 1, the reactions were conducted in toluene at 60 °C. In our primary investigation, PPh_3_AuCl/AgOTf was ineffective for this reaction with no bifuran product being detected. According to previous report^44^, the gold catalyst was known to react with the substrate with a dialkynyl carbinol substructure in different ways, which probably is the reason for the missing selectivity of the tested gold catalyst. Other transition metals were also tested for this transformation. The coinage metal salts, which were typically good catalysts for enynones^34,45,46^, were almost ineffective for this transformation (entries 1–3). ZnCl_2_, another important catalyst for the cycloisomerization of enynones^47^, could not catalyze this reaction either (entry 4). Gratifyingly, the desired bifuran product **2F1** could be successfully produced in 20% yield when (CH_3_CN)_2_PdCl_2_ was applied as the catalyst (entry 5). The yield could be significantly improved when PtCl_2_ was utilized (entry 6, 63%). It has been reported that the presence of a protonic additive could often improve the reaction efficiency through assisting the protodemetalation of the vinylmetal intermediate^48–50^. As expected, the addition of isopropanol (1.1 eq.) as protonic additive could efficiently enhance the reaction efficiency (entry 7), which finished in shorter time (8 h) and afforded the desired product **2F1** in higher yield (80%). Lowering the reaction concentration further improves the reaction yield to 90% (entry 9). With only 1 mol% of catalyst, the yield dropped to 55% after 72 h (entry 10).

**Table 1:** Optimization of the reaction conditions^a^.


**Entry**	**Cat. (mol%)**	***t*/*h***	**Yield**^**b**^
1	Ph_3_PAuCl (5)/AgOTf (5)	24	ND
2	AgNTf_2_ (10)	24	Trace
3	CuCl (10)	24	Trace
4	ZnCl_2_ (10)	24	ND
5	(CH_3_CN)_2_PdCl_2_ (10)	24	20%
6	PtCl_2_ (5)	24	63%
7^c^	PtCl_2_ (5)	8	80%
8^c,d^	PtCl_2_ (5)	24	90%
9^c,d^	PtCl_2_ (1)	72	55%

*ND* means not detected.

^a^Unless otherwise noted, reactions performed at 0.1 M in toluene using 0.20 mmol substrate and catalyst (5–10 mol%) at 60 °C under a N_2_ atmosphere.

^b^Isolated yields.

^c^1.1 eq. of ^*i*^PrOH as additive.

^d^[**1-1**] = 0.025 M.

### Scope of the investigation

**Fig. 2:**
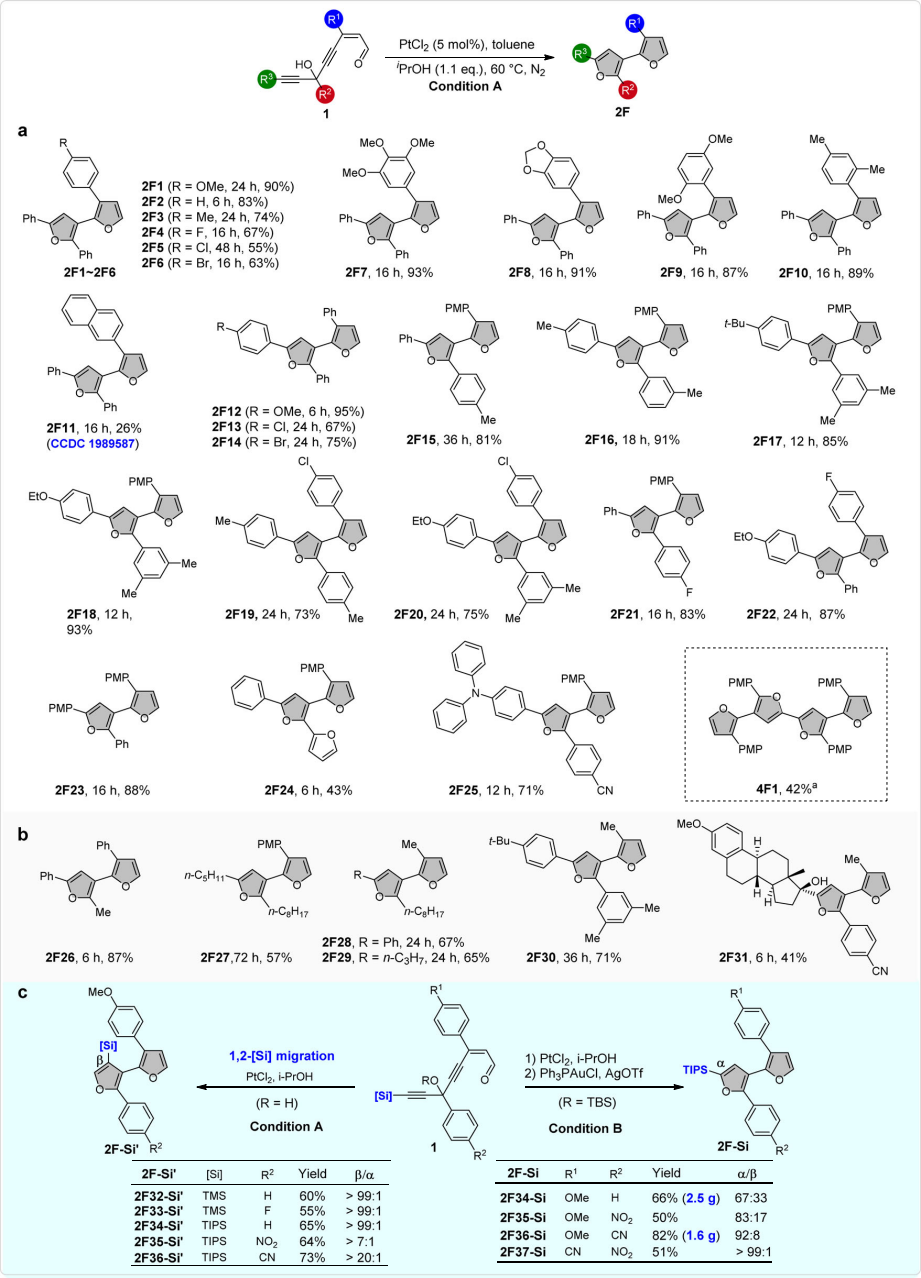
Substrate scope of bifurans. **a** Scope of aryl-substituted bifurans. **b** Scope of alkyl-substituted bifurans. **c** Scope of silyl-substituted bifurans. Unless otherwise noted, the reactions were carried out in 0.2 mmol scale under the standard conditions, PMP = *p*-MeO-C_6_H_4_; (i) PtCl_2_ (5 mol%), ^*i*^PrOH (1.1 eq.), toluene, 60 °C; (ii) (MeCN)_4_CuPF_6_ (5 mol%), toluene, 120 °C. Condition A for 1,2-[Si] migration: PtCl_2_ (5 mol%), ^*i*^PrOH (1.1 eq.), toluene, 60 °C. Condition B for the synthesis of α-silyl-bifuran: (i) PtCl_2_ (5 mol%), ^*i*^PrOH (1.1 eq.), toluene, 60 °C; (ii) PPh_3_AuCl (5 mol%)/AgOTf (5 mol%), toluene, 90 °C. α/β-Regioselectivities were measured based on ^1^H NMR of the crude products.

Having established the optimal reaction conditions (Table 1, entry 8), the substrate scope of this platinum-catalyzed tandem cycloisomerization reaction was then evaluated. As shown in Fig. 2, this reaction could be successfully applied to a variety of enynals **1** with different combinations of groups *R*^1^, *R*^2^, and *R*^3^. The reactions were not strongly affected by the electronic and steric properties, and of groups *R*^1^, *R*^2^, and *R*^3^. In most cases, the yields of bifuran products **2F** are higher than 70% yield. Different halogen atoms and methoxy group at different positions on phenyl ring were all well-tolerated. The enynal with naphthyl of *R*^1^, however, produced the corresponding bifuran **2F11** only in 26%. Interestingly, a trifuran product **2F24** could also be assembled in 43% yield when R^2^ is a furan group. In addition to aryl-bifurans, alkyl-substituted substrates also worked well to afford the desired products (**2F26**–**2F30**). Due to the excellent electron-donating property, the triphenylamine (TPA) unit is frequently installed in organic optoelectronic materials^51^. Gratifyingly, the TPA-containing enynal could smoothly undergo the 1,2-migration rearrangement to form **2F25** in good yield as well. In addition, late-stage modification of ethinylestradiol was illustrated for isolating the corresponding drug conjugates **2F31**, albeit the yield was somewhat low. In addition, the α,β′-bifuran structure was unambiguously confirmed by X-ray crystallographic analysis of **2F11**. The molecular configuration of **2F11** is noncoplanar and with large dihedral angle (66.30°) between two furan units due to the steric hindrance between the neighboring naphthalene and phenyl groups (see Supplementary Fig. 25). To extend the conjugation system of the product, we designed a bis-enynal for the synthesis of tetrafuran through double tandem cycloisomerization. Under the standard reaction conditions, the bis-enynal starting material underwent rapid and complete decomposition but did not give any desired tetrafuran product. We thought that the instability or high reactivity may arise from the two free hydroxyl groups of the starting material. Therefore, a tert-butyldimethylsilyl (TBS)-protected bis-enynal was used instead. Under a slightly modified condition, the desired tetrafuran **4F1** could be produced in 42% yield over two steps.

**Fig. 3:**
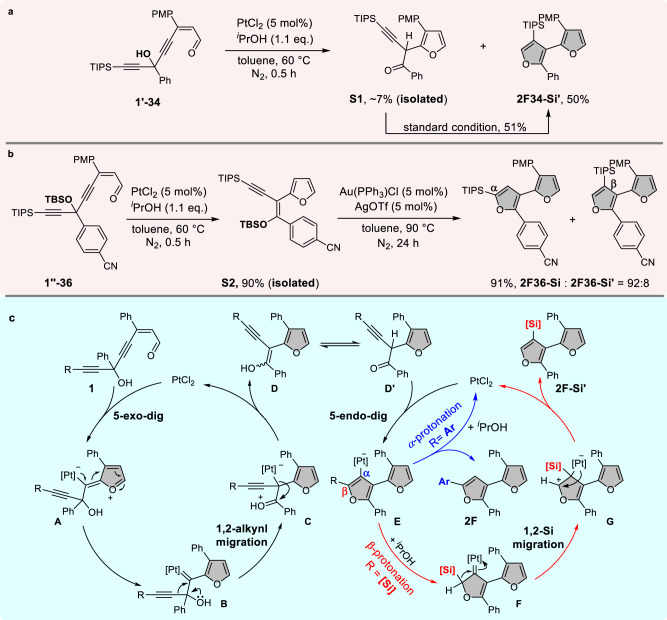
Preliminary mechanism studies and possible mechanism. **a**, **b** Control experiments. **c** Proposed reaction pathway. PMP = *p*-MeO-C_6_H_4_.

As shown in Fig. 2, the bifurans **2F1**–**2F31** have only one α-position open for the potential modifications, such as bromination or stannylation. Such structure will inevitably impede the following applications in the synthesis of oligofurans through cross-coupling reaction. To access the α,β′-bifuran monomer with two α-positions opening for conjugated system extension, we tried to synthesize the enyals with a terminal alkyne group. However, these molecules are unstable and are difficult to prepare. Therefore, we then turned our attention to other alternative ways. It is well known that the use of organosilyl group as a blocking group has been one of the most useful strategies in furan chemistry. Not only are they easy to introduce and remove, but they can be replaced by electrophiles via an ipso-substitution. In addition, the size of organosilyl group also has a pronounced influence on the regioselectivity for the introduction of other groups on the furan ring^52–54^. Therefore, enynals with a silicon terminus on the alkyne were then prepared for the cyclization reactions, aiming at the synthesis of silyl-substituted α,β′-bifuran. As shown in Fig. 2, the cycloisomerization reactions took place smoothly under the standard reaction conditions (PtCl_2_/^*i*^PrOH). However, it is not the desired 2-silylfuran **2F-Si**, but instead a 1,2-Si migration product 3-silylfuran **2F-Si**’. It was demonstrated that functional groups such as methoxy, fluoro, nitro, and nitrile could be well-tolerated under these reaction conditions, affording the C2-unsubstituted 3-silylfurans **2F-Si**’ in good to excellent regioselectivity (Fig. 2c). The results indicated that the substrates with electron-deficient aryl groups produced the corresponding products (**2F35-Si’** and **2F36-Si’**) in a relatively lower regioselectivity. It is supposed that the migration might proceed through a β-silyl palatium carbene intermediate, wherein the 1,2-Si migration is kinetically favored over the 1,2-H^55–58^. To synthesize 2-silyl-bifurans, a stepwise strategy was adopted with TBS-protected enynals as starting material. After systematic screening reaction conditions (see Supplementary Table 2 for details), a stepwise palatium/gold relay-catalytic system was developed for the synthesis of the desired 2-silylfuran **2F-Si**. It was found that a mixture of 2-silylfurans **2F-Si** and 3-silylfurans **2F-Si**’ were often obtained for the electron-rich substrates, but with 2-silylfuran isomer dominated. The yields over two steps ranged from 50% to 82%. The regioselectivity of this reaction was highly dependent upon the electronic properties of the starting materials **1**. The results indicated that the more electronic deficiency of the substrates, the higher α/β-regioselectivity of the bifuran products. For example, the α/β ratio for bearing electron-deficient aryl substituents, the product α/β-regioselectivity increased significantly (**2F35-Si**, **2F36-Si**, and **2F37-Si**). Especially when *R*^1^ = CN, *R*^2^ = NO_2_, 2-silylfuran **2F37-Si** could be obtained with a selectivity of >99/1 in 51% yield. It is noteworthy that a silyl enol ether intermediate could be isolated for the first PtCl_2_-catalyzed step (Fig. 3b). This reaction is quite robust and can be conducted in gram scale. The present methodology constitutes a general and efficient route for the chemodivergent synthesis of silyl bifurans, which are important synthons but not easily available via existing methodologies.

To investigate the reaction mechanism, several control reactions were then performed (Fig. 3a, b). First, when enynal **1’-34** possessing a bulkier triisopropylsilyl terminus was subjected to the standard conditions in a shorter reaction time (0.5 h), the propargyl ketone intermediate **S1** could be isolated in 7% yield. The ketone **S1** could be further converted to **2F-Si’** in 51% yield under standard conditions (Fig. 3a), which indicated that ketone **S1** might be the key intermediate for this cascade reaction. Furthermore, when TBS-protected enynal **1”-36** was employed in the reaction, the silyl enol ether products **S2** of alkynyl migration could be successfully obtained in 90% yield. The silyl enol ether **S2** could be efficiently transformed into the desired bifuran products **2F36-Si** and **2F36-Si’** under the catalysis of the gold catalyst, with 2-silylfuran isomer **2F36-Si** dominated in this process (Fig. 3b).

Based on these preliminary mechanistic observations, a plausible reaction mechanism is proposed in Fig. 3c. First, the alkyne moiety of **1** is activated by the platinum catalyst, which is then attacked by the carbonyl oxygen through 5-exo-dig cyclization to form zwitterionic intermediate **A**, which will isomerize to its resonance structure, (2-furyl)carbene complex **B**. A carbene-involved semi-pinacol rearrangement (1,2-alkyne shift) of intermediate **B** occurs to form intermediate **C**^59^, followed by protodeplatination to enol **D** and then isomerization to propargyl ketone **D’**. A second 5-endo-dig cyclization of **D’** will take place under the catalysis of platinum salt to give furyl-Pt species **E**. When *R* is the aryl group, direct protodemetalation (α-protonation) of **E** will afford the arylated bifuran **2F**. However, when *R* is a silyl group, β-protonation of **E** takes place instead to generate the Pt-carbene **F**, in which the positive charge at the carbene carbon is stabilized by the silicon atom^60^. A 1,2-silicon-over hydrogen migration^61^ then occurs to form **G**, which upon aromatization produces the rearranged product 3-silyl-bifuran **2F-Si’**.

As mentioned in the introduction part, the synthesis of oligofurans mainly relies on the transition metal-catalyzed cross-coupling reactions of appropriate difunctionalized furan monomers^17^. Having established the cascade reaction as a reliable and efficient method for the de novo synthesis of α,β′-bifurans, we then advanced to synthesize bromo- and tin-substituted α,β′-bifuran through the bromination and stannylation reactions^17^. As shown in Supplementary Fig. 1, one or two bromine atoms could be selectively introduced into the backbone of bifuran with NBS as the brominating reagent and the stannylation products were easy accessed as well with Bu_3_SnCl as the tin reagent (see Supplementary Figs. 1 and 2 for more details).

**Fig. 4:**
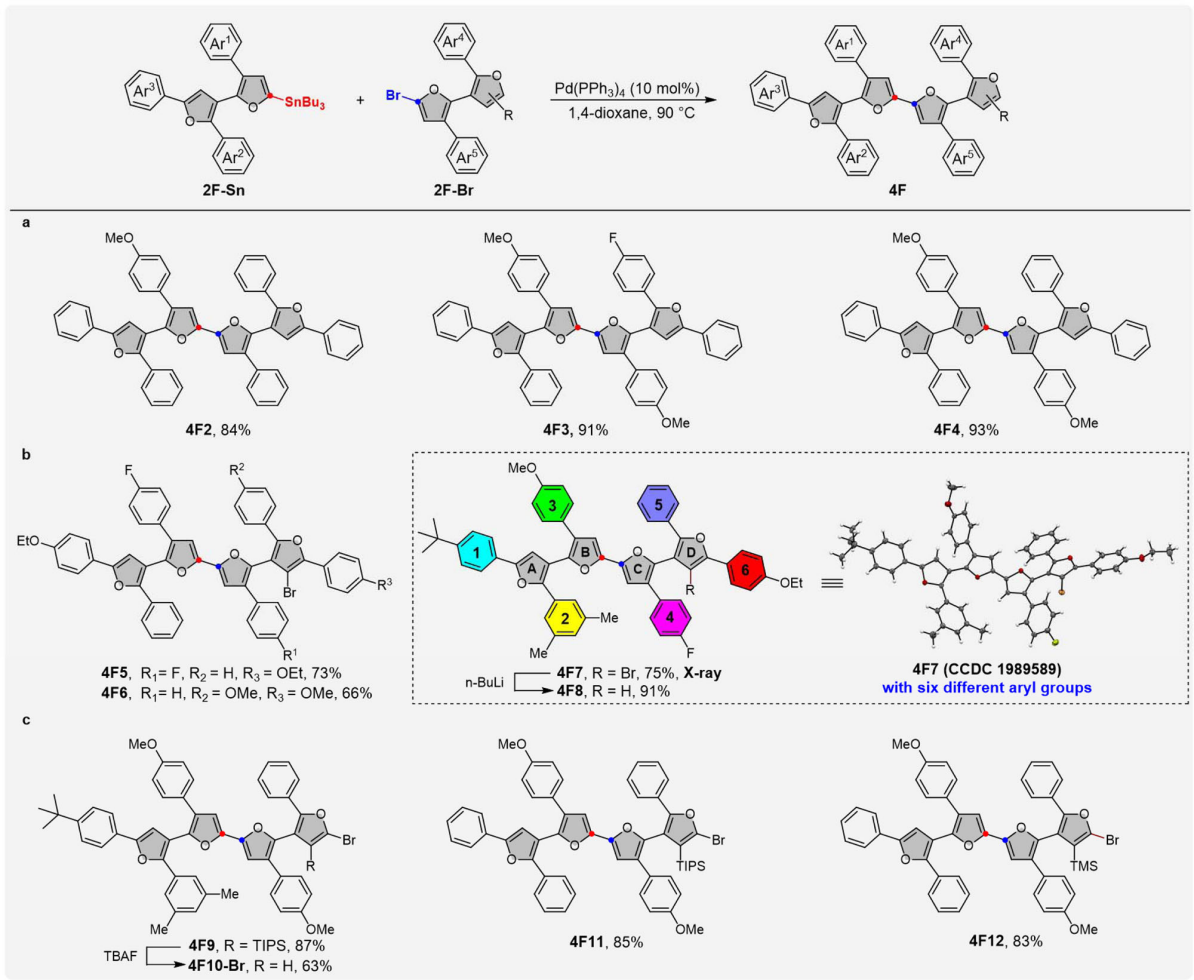
Substrate scope for the synthesis of tetrafurans. **a** Scope of tetrafurans. **b** Scope of 3-bromo-oligofurans. **c** Scope of 3-silyl-2-bromo-oligofurans. Stille couping conditions: Pd(PPh_3_)_4_ (10 mol%), 1,4-dioxane, 90 °C, overnight.

With a class of versatile modular α,β′-bifuran monomers (including bromo- and tin-bifurans) in hand, we then advanced to assemble the desired oligofurans. As shown in Fig. 4, a one-step Stille coupling reaction of bromo-bifurans **2F-Br** or **2F-Br**_**2**_ with stannyl-bifurans **2F-Sn** gave isomerically pure tetrafurans (**4F2**–**4F12**) in good to excellent yields. In addition to the monobromo-bifurans **2F-Br**, 2,3’-dibromo-bifurans could be selectively cross-coupled at 2-bromo-position, leaving 3’-bromine atom unchanged. This unique selectivity provided a good opportunity to access the 3-bromo-oligofuran family (**4F5**–**4F7**). The structure of bromotetrafuran was unambiguously confirmed by X-ray crystallographic analysis of compound **4F7**. The X-ray structure of **4F7** shows that the four furan rings are non-planar. The dihedral angle between A-ring and B-ring is 22.08°. The angle between B-ring and C-ring is 8.47° (almost coplanar), which is similar to α,α-oligofurans^17^. However, the angle between C-ring and D-ring is close to vertical (85.65°), such high torsion angle should arise from the repulsion of two aryl substituents and one bromine atom along the axial. The crystal packing shows no direct *π*–*π* interaction of the oligofuran backbone and features large interplanar distances, which is expected to suppress the fluorescence quenching and increase solid-state emissions. It is worth noting that it is slightly twisted in the backbone of Ar^1^-Ar^2^-A-B-C ring, which is reminiscent of the organoborane acceptor-substituted polythiophene^22^. Treatment of **4F7** with 1.0 eq. of *n*-BuLi produced the oligofuran **4F8** with six different aryl substituents, which is otherwise difficult to synthesize by the conventional methods. As mentioned above, the use of organosilyl group as blocking or masking group has been the most useful in furan chemistry^52^. The silyl group will exert a profound influence upon the regioselectivity of the furan ring. For example, when 3-silyl-2,2’-dibromo-bifurans were used as coupling partners to react with stannyl-bifurans, only the bromine atom on the nonsilyl-furan is reactive for the Stille reactions. Wherein the silyl group (trimethylsilyl TMS or triisopropylsilyl TIPS) served as a good masking group, the bromine close to the bulky silyl group remained unreactive towards the standard Stille coupling reactions (see **4F9**, **4F11**, and **4F12**). More importantly, the masking group of silyls could be easily removed with tetrabutylammonium fluoride (TBAF) and then the reactivity of the bromofuran will be released for the Stille coupling reaction. For instance, upon treatment with TBAF at 50 °C in tetrahydrofuran (THF), **4F9** underwent complete protodesilylation, affording the corresponding tetrafuran **4F10-Br** in 63% yield, which could be utilized as important building block to access longer oligofuran (vide infra).

**Fig. 5:**
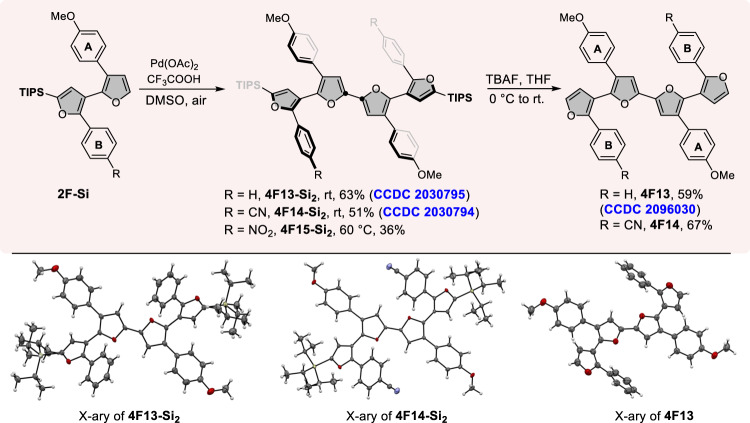
Bottom-up synthesis of tetrafuran through oxidative homocoupling. Oxidative homocoupling reactions were run with 0.25 mmol of **2F-Si** (1.0 eq.), Pd(OAc)_2_ (1.0 eq.), and CF_3_COOH (1.0 eq.) in 1.5 mL DMSO at r.t., isolated yields. Desilication reactions were run with **4F-Si**_**2**_ (0.2 mmol) and tetrabutylammonium fluoride (TBAF) (1.0 M in THF, 0.4 mL, 0.4 mmol) in 4 mL THF.

In addition to the Stille coupling reactions, the direct oxidative homocoupling of two bifurans through the cleavage of two C-H bonds could also be harnessed to assemble the symmetric oligofurans^62^ (Fig. 5). The homocoupling reaction of **2F-Si** with 1.0 eq. of Pd(OAc)_2_ as catalyst and oxygen (air) as the oxidant proceeded smoothly in dimethyl sulfoxide to furnish **4F-Si**_**2**_ products in 36–63% yields (**4F13-Si**_**2**_, **4F14-Si**_**2**_, and **4F15-Si**_**2**_). The masking silyl groups at α-position of **4F-Si**_**2**_ could also be facilely removed with TBAF, affording another type of important monomer **4F13** and **4F14** with four arylfurans in good yields, as the newly released α-positions could be used as potential coupling sites for the synthesis of longer oligofurans. The structures of tetrafuran were confirmed by X-ray crystallographic analysis of compound **4F13-Si**_**2**_, **4F14-Si**_**2**_, and **4F13** (see Supplementary Figs. 29–34). The molecular configuration of these three tetrafurans are the same on the whole. The inside two furan rings are fully coplanar, within the accuracy of atomic positions. The outer two furan rings are non-planar, with the dihedral angles being 51.52° for **4F13-Si**_**2**_, 36.02° for **4F14-Si**_**2**_, and 30.13° for **4F13**, which is similar to that of **4F7** (22.08°). The above X-ray crystallographic analysis of oligo(arylfuran)s indicated that the introduction of different aryl groups would affect the conformation of oligofuran, the steric-hindrance effect between two neighboring aryl groups disrupted the planarity of backbone of these oligo(arylfuran)s, but the coplanarity of α,α-linked furans was almost unaffected.

**Fig. 6:**
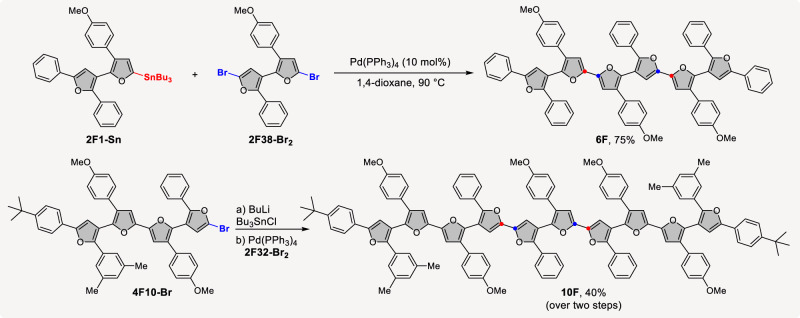
Bottom-up synthesis of sexifuran and decafuran. Reaction conditions: please see pages 61 and 62 of Supplementary Information.

As an exhibition of superiority of the modular and programmable strategy, structurally diverse longer oligofurans were assembled quickly in a controlled and designed manner by choosing different bifurans and tetrafurans as coupling partners (Fig. 6). For example, a double Stille coupling reaction of **2F1-Sn** and **2F38-Br**_**2**_ under the catalysis of Pd(PPh_3_)_4_ successfully afforded isomerically pure sexifuran **6F** in 75% isolated yield. In the same manner, functionalized tetrafuran could be used as a building block directly as well. Taking bromotetrafuran **4F10-Br** as an example, after stannylation with Bu_3_SnCl and coupling with 2,2’-diboromo-bifuran **2F32-Br**_**2**_ under catalysis of Pd(PPh_3_)_4_, the decifuran **10F** with ten arylfuran units could be assembled successfully in 40% yield over two steps. To the best of our knowledge, this also represents the longest α,β-oligofuran.

### Photophysical properties

**Fig. 7:**
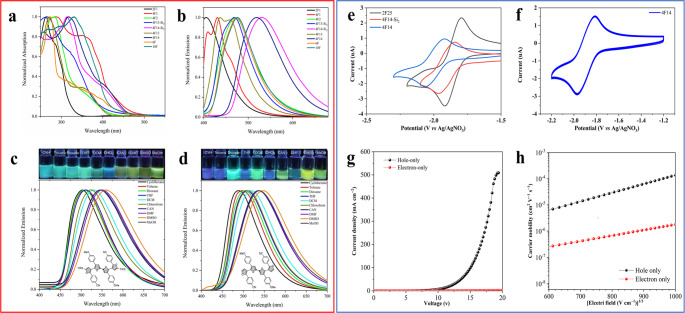
The photophysical, electrochemistry, and carrier transport properties of oligo(arylfuran)s. **a** Absorption spectra of representative oligo(arylfuran)s in DCM (concentration: 10 μM). **b** Emission spectra in DCM (*λ*_ex_ = 365 nm, concentration: 10 μM). **c** Fluorescence spectra of **4F14-Si**_**2**_ in solvents of different polarity (CAN, acetonitrile; DCM, dichloromethane; DMF, *N*,*N*-dimethylformamide; DMSO, dimethyl sulfoxide; THF, tetrahydrofuran) under 365 nm excitation. Concentration: 10 μM. **d** Fluorescence spectra of **4F14** in solvents of different polarity. **e** Cyclic voltammograms of **2F25**, **4F14-Si**_**2**_, and **4F14** measured in DMF containing 0.1 M tetrabutylammonium perchlorate (*n*-Bu_4_NClO_4_) with a scanning rate of 100 mV/s at room temperature. **f** Repetitive CV scans of **4F14** in DMF with 0.1 M *n*-Bu_4_NClO_4_ (scan rate 100 mV/s). **g** Current density–voltage curves of devices H1 and E1. **h** Carrier mobility-electric field curves of devices H1 and E1.

So far, the photophysical properties of oligofurans are only limited to α,α-oligofurans and β,β-oligofurans^15–21^. The ultraviolet (UV)/visible absorption and emission spectra of the representative bifuran (**2F**), tetrafuran (**4F**), sexifuran (**6F**), and decafuran (**10F**) in solutions were then characterized and shown in Fig. 7a, b. The absorption and emission maxima of these complexes vary from 263 to 332 nm and from 430 to 530 nm, respectively, depending on the number of furan units and aryl groups. To the general trends, the absorption was red-shifted as the number of furan units increased, such as the *λ*_max_ of **10F** was bathochromically shifted by 46 nm compared to **2F1**. However, the aryl substituents of oligofurans had much more impact on the photophysical properties than the number of furan units. Oligofurans **4F13-Si**_**2**_ and **4F13**, in which all the aryl groups only having electron donating groups, show blue shifts in the *λ*_max_ relative to push–pull oligofuran **4F14-Si**_**2**_ and **4F14**, respectively. The organosilyl groups on the terminus of the oligofuran have little effect on the UV absorption, such as the **4F14** (318 nm, 403 nm) and **4F14-Si**_**2**_ (313 nm, 401 nm). Collectively, there was no obvious linear correlation as the increment of number of furan, which indicated that the planarity is disrupted^17^.

The solutions of these oligo(aryl)furans in dichloromethane (DCM) were highly fluorescent. The emission wavelengths can effectively cover the whole visible light range from 400 to 700 nm. The maximum fluorescence emission wavelength of bifuran **2F1** was located at 405 nm. With increasing the number of furan units, the fluorescence emission wavelength has a significant red shift, such as **4F13-Si**_**2**_ (469 nm). However, the shift of the fluorescence emission peak will not have larger red shifts when the number of furan rings continues to increase to 6 or 10. This observation is completely different with those non-substituted α,α-oligofurans in the literature^17^. The reason for this phenomenon may be that the oligo(arylfuran)s are not a large conjugation system because of the introduction of aryl groups into the oligofuran backbones, as exemplified by the X-ray structures of **4F7**, **4F13-Si**_**2**_, **4F14-Si**_**2**_, and **4F13**. Oligofuran **4F14** showed relatively strong emission in solution and has a relatively longer emission (519 nm) than other oligofurans. We postulated that this red shift might result from the presence of the para-CN group at the B phenyl group, which augments the efficiency of the CT process. Interestingly, the introduction of silyl group at the α-terminus (**4F14-Si**_**2**_) will make the emission wavelengths further shift to 530 nm^63^, which is the most red-shifted emission. These results indicated that the strategy of combining the push–pull design and silyl effect is a viable one for red-shifting the absorption and emission band of oligofurans, which enable this protocol to prepare near-infrared oligo(arylfuran)s.

In addition, the fluorescence spectroscopic behaviors of **4F14-Si**_**2**_ and **4F14** were also investigated in the solid state. The emission spectra of the solids were shown in Supplementary Fig. 9. In general, the emission bands are nonsymmetric and very broad, whereby the band broadening of **4F14-Si**_**2**_ is significantly more pronounced. Similarly, analysis of the fluorescence spectra of bisilyl substituted tetrafuran derivatives **4F14-Si**_**2**_ demonstrates that silyl substitution makes emission peak shift to a longer wavelength by 93 nm.

Intrigued by the strongly red-shifted emission, we also undertook solvochromicity studies with **2F25**, **4F13-Si**_**2**_, **4F14-Si**_**2**_, **4F14**, and **10F** to scrutinize the effect of the solvent environment on their emission properties (Fig. 7c, d and Supplementary Fig. 10). It is visually evident that compounds **4F14-Si**_**2**_ and **4F14** exhibited positive emission solvatochromism. Their emissions are shifted bathochromically with increasing solvent polarity (cyclohexane to methanol) and the corresponding emissions span the wavelengths from 502 to 559 nm and from 489 nm to 549 nm, respectively, covering blue, green, yellow, and orange regions of visible spectrum (Fig. 7c, d). Such strong solvatochromism occurs due to a combination of intramolecular charge transfer (ICT) and dipole–solvent interactions in solutions^64^. On the contrary, the absorption of **4F14-Si**_**2**_ exhibited little change on changing the solvent polarity (Supplementary Fig. 6). The absorption maximum of **4F14-Si**_**2**_ only changed from 324 to 328 nm by increasing the solvent polarity. Thus, the solvent dependence of the emission shows that the excited state is stabilized in more polar solvents, due to ICT effect^65^.

The solution fluorescence quantum yields (*Φ*_F_s) were estimated for compounds **2F2**–**4F14**, as shown in Table 2. The *Φ*_F_s in film and solid states are also summarized in Supplementary Table 4. The *Φ*_F_s of all the target molecules are high in THF solutions, which are 90.8% for **2F25**, 68.0% for **4F14-Si**_**2**_, 77.9% for **4F13**, and 64.5% for **4F14**, respectively. However, their *Φ*_F_s in film and solid states are decreased, due to promoted non-radiative decay by strong intermolecular interactions. On the other hand, it can be seen that compound **4F13** shows a relatively higher *Φ*_F_ of 38.6% than with those of the other four compounds. This reveals that the introduction of electron-donating substituent (–OMe) alone to oligofuran structures may be beneficial to the fluorescence emission in solid state.

**Table 2:** Physical properties of 2F2, 2F25, 4F14-Si_2_, 4F13, and 4F14.

Compound	*λ*_abs_^a^ (nm)	*Φ*_F_^b^ (%)			*λ*_onset_^e^ (nm)	*E*^re^_onset_^f^ (V)	HOMO/LUMO^g^ (eV)	*E*_g_^h^ (eV)	HOMO/LUMO^i^ (eV)	*E*_g_^i^ (eV)
		THF^c^	Film^d^	Powder						
**2F2**	328	17.8/19.7	5.5	4.2	381	−2.24	−5.41/−2.16	3.25	−5.48/−1.61	3.87
**2F25**	301, 396	80.8/90.8	3.0	3.5	468	−1.52	−5.62/−2.88	2.74	−5.23/−2.15	3.08
**4F14-Si**_**2**_	311, 387	56.3/68.0	7.5	11.5	450	−1.80	−5.44/−2.60	2.84	−5.29/−2.29	3.00
**4F13**	272, 360	68.3/77.9	6.4	38.6	414	−2.12	−5.37/−2.28	3.09	−4.97/−1.62	3.35
**4F14**	294, 385	55.0/64.5	1.1	1.3	451	−1.89	−5.38/−2.54	2.84	−5.38/−2.37	3.01

^a^Measured in DMF (10^−5^ M) under N_2_.

^b^Absolute fluorescence quantum yield determined using a calibrated integrating sphere.

^c^Measured in THF (10^−5^ M) under air and N_2_, respectively.

^d^Film drop-casted on a quartz plate.

^e^Estimated from the onset of the UV/Vis absorption spectra.

^f^*E*_onset_ vs. Fc/Fc^+^ estimated by a CV method using a (Pt) disc electrode, Pt wire as a counter electrode, and Ag/AgNO_3_ as a reference electrode in DMF.

^g^Obtained from CV in DMF/n-Bu_4_NClO_4_ and estimated from LOMO = −(4.40 − *E*^re^_onset_); HUMO = LOMO − *E*_g_.

^h^Obtained from the absorption edge, *E*_g_ = 1240/*λ*_onset_.

^i^Obtained from DFT calculations.

### Thermal and optical stability

All the synthesized oligo(arylfuran)s exhibit excellent solubility in common organic solvents, such as toluene, chloroform, DCM, and THF. Tetrafurans are stable in powdered form and even as a DCM solution when exposed to air (including moisture air) at room temperature for up to at least a few weeks. The simple furan derivatives containing electron-rich groups reported in the previous literature are usually unstable with respect to a combination of light and oxygen, are known to react with singlet oxygen to form unsaturated esters, or decompose via ring opening of furan endoperoxide^25^. The stability of the series of oligo(arylfuran)s was verified by thermogravimetric analysis  (TGA) measurement (Supplementary Fig. 12) and a parameter of *T*_d10_ (10% weight-loss temperature)^66^ is used to describe the thermal durability of these compounds (Supplementary Table 5). The TGA curves of oligo(arylfuran)s indicated that they remained almost unchangeable when the temperature is below 230 °C and the *T*_d10_ values were found to range from 267 °C to 457 °C. The push–pull derivatives **4F14** and **4F14-Si**_**2**_ show higher thermal stability compared with other oligo(arylfuran) compounds. It is worth mentioning that the *T*_d10_ value for the **4F14** can still reach 414 °C even in the atmosphere of air, which suggests that it can satisfy the stability requirements for applications in organic electronic devices. Meanwhile, we also examined the ambient photostability of **2F25**, **4F13-Si**_**2**_, **4F14-Si**_**2**_, **4F13**, and **4F14** measured under the same conditions. All of them remained basically stable during the time tested (Supplementary Figs. 12 and 13). **2F25**, **4F13**, and **4F14** show negligible bleaching, even after prolonged (24 h) exposure. TIPS-substituted **4F** show more significant bleaching with 13% bleaching for **4F13-Si**_**2**_ and 27% bleaching for **4F14-Si**_**2**_ after 24 h.

### Theoretical calculations

**Fig. 8:**
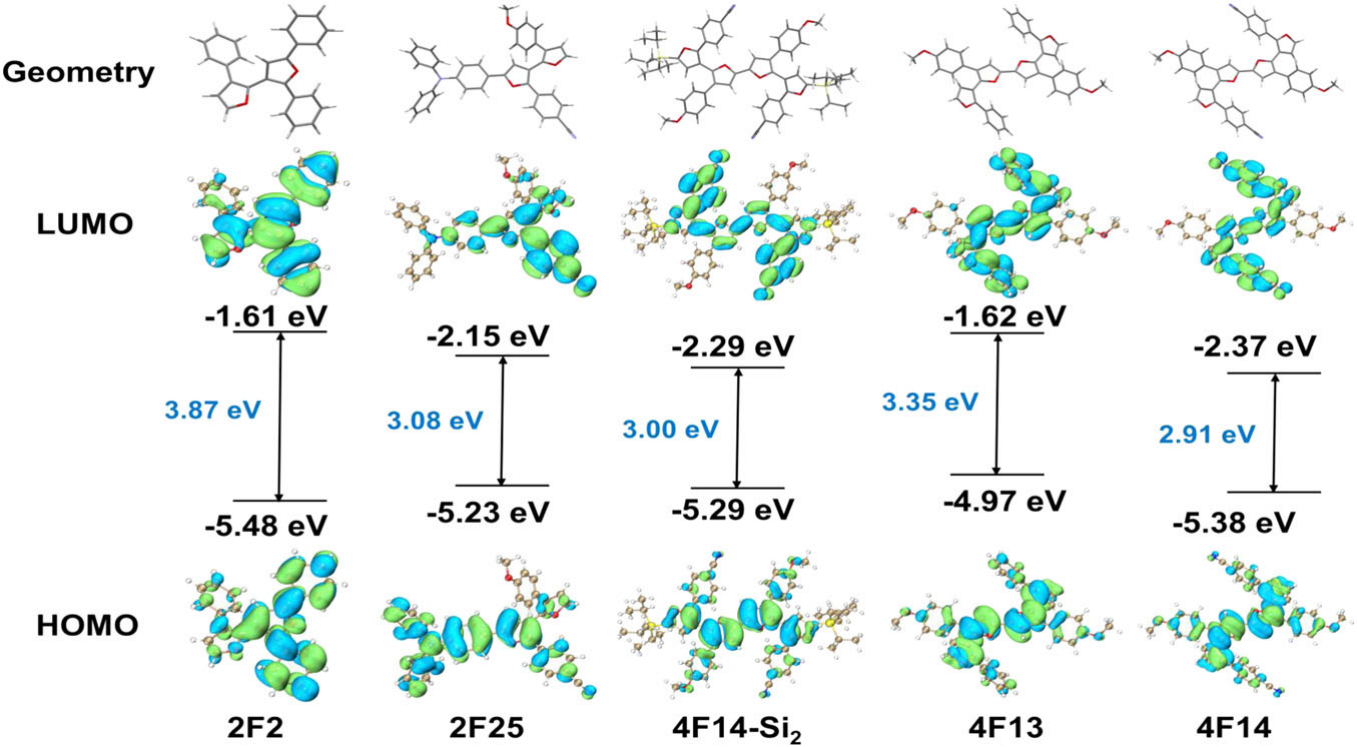
The density functional theory (DFT) calculations of oligo(arylfuran)s. Calculated ground-state geometries, molecular orbital amplitude plots, and energy levels of HOMOs and LUMOs of **2F2**, **2F25**, **4F14-Si**_**2**_, **4F13** and **4F14**.

To further understand the structure–property relationship of the oligofuran derivatives, theoretical calculation based on density functional theory (DFT) were carried out to obtain the optimized geometries and the frontier orbitals distribution of **2F2, 2F25, 4F14-Si**_**2**_**, 4F13**, and **4F14**. As shown in Fig. 8, *ortho*-substituted benzene rings increase the intramolecular steric hindrance; as a result, the planarity and the rigidity of the oligofuran skeletons decrease to some extent. The HOMOs of the oligofuran derivatives mainly distribute on the electron-rich groups such as furan, TPA, and methoxybenzene moieties. For the compounds modified with electron-withdrawing CN groups, namely **2F25**, **4F14-Si**_**2**_, and **4F14**, their lowest unoccupied molecule orbitals (LUMOs) distribute on not only the central furan rings but also the benzene rings modified with CN, which are different with HOMOs, indicating that **2F25**, **4F14-Si**_**2**_, and **4F14** have ICT characters, which is the main reason of their emission spectra showing obvious solvent effect. Besides, the HOMO energy levels of these oligofuran derivatives are calculated to be around −5.2 eV, which are close to those of typical hole-transport materials such as di-(4-(*N*,*N*-ditolyl-amino)-phenyl)cyclohexane (TAPC, HOMO = −5.3 eV)^67^ and *N*,*N*-di(naphthalene-1-yl)-*N*,*N-*diphthalbenzidine (HOMO = −5.3 eV)^68^. Therefore, it is reasonable to speculate that these oligofuran derivatives should have good potential of being applied in organic semiconductors as hole-transport materials. To get deeper insights of the photophysical properties of the oligofuran derivatives, calculation based on time-dependent DFT was carried out using **4F14** as the model. The calculated spectra (Supplementary Fig. 16) are qualitatively consistent with the experimental spectra in terms of both wavelength and the shape of spectra, indicating that the calculated results are reliable. According to the calculated absorption spectrum, *S*_0_ → *S*_1_ transition has obvious ICT characters, because it is dominated by HOMO and LUMO. In order to understand the emission properties of the oligofuran derivatives comprehensively, natural transition orbitals are taken into consideration. As shown in Supplementary Fig. 17, the holes are basically located on the central furan moieties, whereas a large amount of electrons are located on one of the benzene rings modified with CN groups, indicative of obvious ICT characters, which should be the reason of why solvent effect is observed in the emission spectra of **4F14**.

### Electrochemical properties

To investigate the electrochemical properties of these oligofurans, cyclic voltammetry (CV) with ferrocene/ferrocenium (Fc/Fc^+^) couple as the internal standard were carried out. As shown in Fig. 7e and Supplementary Figs. 18–22, **2F2**, **2F25**, **4F14-Si**_**2**_, **4F13**, and **4F14** exhibited one pair of reversible reduction and oxidation peaks, respectively. The LUMO energy levels were calculated to be −2.16, −2.88, −2.60, −2.28, and −2.54 eV, respectively. Their HOMO energy levels could be obtained from optical band gap energies (estimated from the onset wavelength of their UV spectra and the data are listed in Table 2) and LUMO energy levels, which were −5.41, −5.62, −5.44, −5.37, and −5.38 eV, respectively (Table 2). The electrochemical band gaps are roughly consistent with the optical energy gaps estimated from the absorption edges. Good redox properties of these compounds indicated the potential applications in optoelectronic devices. The cyclic voltammograms and HOMO/LUMO data of other precursors (**2F2** and **4F13**) were shown in the Supplementary Information (see Supplementary Figs. 18 and 21). The CV curves remain unchanged under multiple successive potentials scans, indicating the good stability of these oligo(arylfuran)s (Fig. 7f).

### Carrier transport

In order to explore the potential application of oligofuran derivatives as organic semiconductors, the carrier transport ability of **4F14-Si**_**2**_ was investigated as an example by the space-charge-limited-current (SCLC) method. Hole- and electron-only devices with configurations of ITO/TAPC (10 nm)/**4F14-Si**_**2**_ (80 nm)/TAPC (10 nm)/Al (120 nm) (device H1) and ITO/TmPyPB (10 nm)/**4F14-Si**_**2**_ (80 nm)/TmPyPB (10 nm)/LiF (1 nm)/Al (120 nm) (device E1) were fabricated, respectively. In devices H1 and E1, thin layers (10 nm) of TAPC (hole mobility*,* ca. 10^−2^ cm^2^ V^−1^ s^−1^) and TmPyPB (electron mobility*,* ca. 10^−3^ cm^2^ V^−1^ s^−1^) were used as buffer layers between **4F14-Si**_**2**_ and the electrodes. Considering the much thicker **4F14-Si**_**2**_ layer (80 nm), the influence of the thin buffer layers with high carrier mobilities can be excluded during the calculation and the mobility results primarily reflect the intrinsic property of **4F14-Si**_**2**_. Figure 7g shows the current density–voltage (*J–V*) characteristics of electron- and hole-only devices based on **4F14-Si**_**2**_. The *J–V* behaviors show ohmic characteristics. Along with the increase of voltage, the current density grows into space-charge limited, which is quadratically dependent on the applied voltage. The SCLC characteristics can be studied by the reported mehtod^69^. Figure 7h displays the field-dependent hole and electron mobilities. Under the electric field of 600 ~1000 V cm^−1^, the hole mobility is in the range of 5.89 × 10^−6^ ~1.37 × 10^−4^ cm^2^ V^−1^ s^−1^, which is much higher than the electron mobility (3.97 × 10^−7^ ~1.81 × 10^−6^ cm^2^ V^−1^ s^−1^), demonstrating that **4F14-Si**_**2**_ basically is a *p*-type semiconductor and can be used as a hole-transporting material for electrical devices.

## Discussion

In summary, we have developed the bottom-up modular construction of chemically and structurally well-defined multiaryl-substituted oligofurans via a platinum-catalyzed de novo synthesis of bifuran monomers and palladium-catalyzed cross-coupling reaction. It is worth mentioning that tetrafuran **4F8** with six different phenyl substituents could be synthesized by this means. Single-crystal X-ray analyses revealed that the steric-hindrance effect between aryl groups and neighboring furan rings disrupted the planarity of backbone of these oligo(arylfuran)s, but there still have manipulating space in achieving slight twist oligo(arylfuran)s in terms of the crystal data of **4F7**. These twisted oligo(arylfuran)s exhibit excellent solubility in common organic solvents (toluene, chloroform, DCM, and THF) and the high stability towards heat, oxygen, and moisture. More importantly, the photophysical properties could also be well-modulated by installing different aryl groups, such as tunable and polarity-sensitive fluorescence emission and high quantum yields in THF solution. Besides, DFT calculations, electrochemical studies, and carrier transport experiments show that oligo(arylfuran) should have good potential of being applied in organic semiconductors as hole-transport materials. The modular assembled oligo(arylfuran)s generated by varying the aryl substituents in the resulting oligomers together with rapid characterization methods will provide us a way to the accelerated investigation on structure–property relationships. Further investigations of the application of these oligo(arylfuran)s are currently in progress in our laboratory.

## Methods

### General procedure I

Under nitrogen atmosphere, to a solution of enynals **1** (0.2 mmol) in dry toluene (0.025 M), PtCl_2_ (0.05 eq., 2.7 mg) and ^*i*^PrOH (1.1 eq., 13.2 mg) were added. The reaction mixture was then heated to a temperature of 60 °C and stirred for 6–48 h. After the reaction was completed, the reaction mixture was filtered through short silica gel and then the solvent was removed under reduced pressure. The bifuran product was purified by flash column chromatography (silica gel, petroleum ether/AcOEt = 100:1) to yield **2** **F**.

### General procedure II

Under nitrogen atmosphere, to a solution of enynals **1** (0.2 mmol) in dry toluene (0.025 M), PtCl_2_ (0.05 eq., 2.7 mg) and ^*i*^PrOH (1.1 eq., 13.2 mg) were added. The reaction mixture was then heated to a temperature of 60 °C and stirred for 6–48 h. After the reaction was completed, the reaction mixture was filtered through short silica gel and then the solvent was removed under reduced pressure. The bifuran product was purified by flash column chromatography (silica gel, petroleum ether/AcOEt = 100:1) to yield silyl enol ether intermediate **S**.

Under nitrogen atmosphere, to a solution of **S** (0.1 mmol) in dry toluene (0.1 M), Au(PPh_3_)Cl (5 mol%, 2.4 mg) and AgOTf (5 mol%, 1.2 mg) were added. The reaction mixture was then heated to 90 °C and stirred for 12–24 h. After the reaction was completed, the reaction mixture was filtered through short silica gel and then the solvent was removed under reduced pressure. The bifuran product was purified by flash column chromatography (silica gel, petroleum ether/AcOEt = 10:1) to yield **2F-Si**.

## Supplementary information

Supplementary Information

## Data Availability

The authors declare that all other data supporting the findings of this study are available within the article and Supplementary Information files, and also are available from the corresponding author upon reasonable request. The data for X-ray crystallographic structure **2F11**, **4F7**, **4F13-Si**_**2**_, **4F14-Si**_**2**_, and **4F13** has been deposited in the Cambridge Crystallographic Database Center under accession number CCDC: 1989587, 1989589, 2030795, 2030794, and 2096030. These data can be obtained free of charge from The Cambridge Crystallographic Data Centre via www.ccdc.cam.ac.uk/data_request/cif.
