# Supplementary material for: Bottom-up modular synthesis of well-defined oligo(arylfuran)s
Source: Nat Commun. 2021 Oct 25;12:6165. doi: 10.1038/s41467-021-26387-5 (PMC8546054; doi:10.1038/s41467-021-26387-5)
Supplement: Supplementary file 1 — Supplementary Information [file 41467_2021_26387_MOESM1_ESM.pdf]

Supplementary Information

Bottom-up Modular Synthesis of Well-Defined Oligo(arylfuran)s

Yang Chen,[1] Pingchuan Shen,[2] Tongxiang Cao,[1] Hao Chen,[2] Zujin Zhao,*[,2] and Shifa Zhu*[,1,3]

[1]Key Laboratory of Functional Molecular Engineering of Guangdong Province, School of Chemistry and Chemical Engineering, South China University of Technology, Guangzhou 510640, China;
[2]State Key Laboratory of Luminescent Materials and Devices, Guangdong Provincial Key Laboratory of Luminescence from Molecular Aggregates, South China University of Technology, 510640 Guangzhou, China;
[3]Guangdong Youmei Institute of Intelligent Bio-manufacturing Co., Ltd.

Table of contents

I. Supplementatry Methods 3

II. Supplementary Discussion 63

III. Supplementary NMR Spectra 86

IV. Supplementary References 223

I. Supplementatry Methods

General information

All reactions were conducted under dry N_2 atmosphere in Schlenk tube. Catalysts were commercially available. Commercially obtained reagents were used without purification. Reaction starting materials were prepared as depicted in the literature. ^{1}H, ^{13}C NMR spectra were recorded on the Bruker AVANCE 400 (400 MHz for ^{1}H; 101 MHz for ^{13}C; 376 MHz for 19F) and Bruker AVANCE 500 (500 MHz for ^{1}H; 126 MHz for ^{13}C; 471 MHz for 19F), ^{1}H NMR and ^{13}C NMR chemical shifts were determined relative to internal standard TMS at δ 0.0. Chemical shifts (δ) are reported in ppm, and coupling constants (J) are in Hertz (Hz). The following abbreviations were used to explain the multiplicities: s = singlet, d =doublet, t = triplet, q = quartet, m = multiplet, br = broad. Infrared (IR) spectra are recorded on a Nicolet 210 spectrophotometer and were recorded in potassium bromide (KBr) pellet. Mass spectra (MS) were obtained using ESI and DART mass spectrometer. The hybrid functional B3LYP was applied with the 6-311G(d,p) basis set. Energy level of frontier orbitals are obtained by calculating the single-point energy basing on optimized S_0 geometries with functional B3LYP with def2-TZVP basis set. Fluorescence spectra were recorded on Hitachi F-4500 and F-7000 fluorescence spectrophotometers. TGA spectra were recorded on SDT Q600 V8.3 Build 101. Photoluminescence quantum yields were measured using a Hamamatsu absolute PL quantum yield spectrometer C11347 Quantaurus_QY. Cyclic voltammetry was performed on conventional three-electrode system at CHI660E electrochemical workstation (Chenhua, Shanghai, China). All reagents were used as received from commercial sources, unless specified otherwise, or prepared as described in the literature.

Supplementary Table 1. Optimization of the reaction conditions for bifuran synthesis.[a]

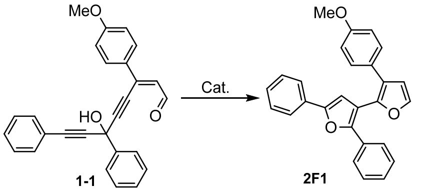

entry	cat. (mol%)	sol.	T(°C)	t/h	Yield[b]
1	$ZnCl_2$ (10)	DCM	rt	8	ND[f]
2	$PtCl_2$ (5)	PhMe	rt	24	NR
3	$ZnCl_2$ (10)	PhMe	60	24	ND
4	$Pd(MeCN)_2Cl_2$ (10)	PhMe	60	24	20%
5	CuCl (10)	PhMe	60	24	trace
6	Ph_3PAuCl (5)/AgOTf (5)	PhMe	60	24	ND
7	$AgNTf_2$ (10)	PhMe	60	24	trace
8	$PtCl_2$ (5)	PhMe	60	24	63%
9[c]	$PtCl_2$ (5)	PhMe	60	8	51%
10[d]	$PtCl_2$ (5)	PhMe	60	8	80%
**11[d,e]**	**$PtCl_2$ (5)**	**PhMe**	**60**	**24**	**90%**
12[d,e]	$PtCl_2$ (1)	PhMe	60	72	55%

[a]Unless otherwise noted, reactions performed at 0.1 M in toluene using 0.20 mmol substrate and catalyst at 60 °C under a N_2 atmosphere. [b]Isolated yields. [c]1.1 equiv. of MeOH as additive. [d]1.1 equiv. of ^{i}PrOH as additive. [e][**1-1**] = 0.025 M. [f]ND: not detected.

2. Supplementary Table 2. Optimization of the reaction conditions for selective 1,2-Si or 1,2-H migration.[a]

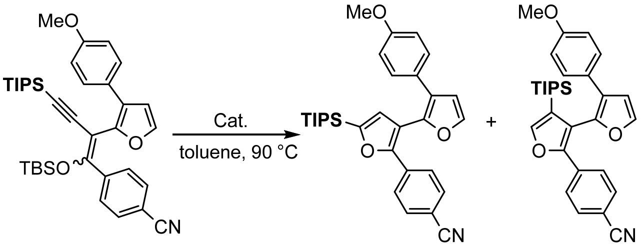

entry	cat. (mol%)	Yield[b]	2F-Si/2F-Si'[c]
1	$Zn(OTf)_2$ (10)	NR	/
2	$Fe(OTf)_2$ (10)	NR	/
3	$Yb(OTf)_3$ (10)	NR	/
4	CuCl (10)	NR	/
5[d]	$Cu(OTf)_2$ (10)	32%	50/50
6[d]	$Cu(CH_3CN)_4PF_6$ (10)	50%	95/5
**7**	**$Au(PPh_3)OTf$ (5)**	**82%**	**92/8**
8[e]	$Au(PPh_3)OTf$ (5)	53%	52/48

[a]Unless otherwise noted, reactions performed at 0.1 M in toluene using 0.20 mmol substrate and catalyst (5 − 10 mol%) at 90 °C under a N_2 atmosphere. [b]Isolated yields. [c]**2F-Si/2F-Si'** regioselectivities were measured based on H[1]NMR of the crude products. [d]120 ℃. [e]Solvent is DCE.

3. General procedure for preparation of enynals.[1]

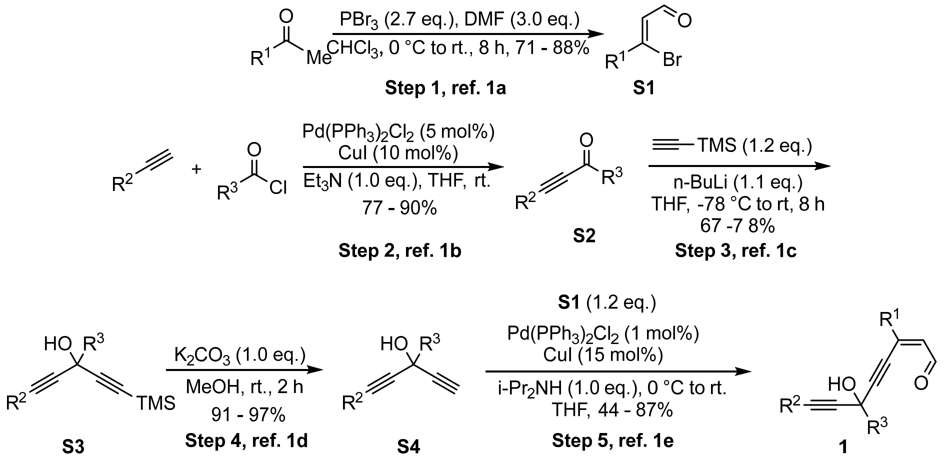

General procedure for preparation of bromo aldehyde (S1).[1a]

To a mixture of DMF (3.0 eq.) and chloroform (0.5 M) was added PBr_3 (2.7 eq.) dropwise at 0 ℃, and the mixture was stirred for 60 min. before the addition of ketone (1.0 eq). The resulting solution was stirred for 8 h at room temperature before it was poured into 300 ml water, neutralized with solid $NaHCO_3$, and extracted with dichloromethane. The extract was washed with a saturated NaCl solution, dried over anhydrous $MgSO_4$, and concentrated under reduced pressure. Purification of the crude product by flash column chromatography (silica gel, petroleum ether/ ethyl acetate = 20:1) gave **S1** with 71 - 88% yield.

General procedure for preparation of (S2).[1b]

In a flask under nitrogen was added CuI (0.05 eq.) to dry THF (0.30 M). Et_3N (2.00 equiv.) was added dropwise, followed by the alkyne (1.0 eq.) and acid chloride (2.0 eq.). The reaction mixture was stirred at room temperature overnight (18 hours). The solution was diluted with Et_2O and washed with water. The organic layers were combined, dried over $MgSO_4$, filtered and the solvents were removed under vacuum. The crude product **S2** was purified on flash chromatography (petroleum ether/ethyl acetate =50:1).

General procedure for preparation of propargylic alcohol (S3).[1c]

Trimethylsilylacetylene (1.2 eq) was dissolved into THF (0.5 M), and the solution was cooled to -78 ℃. To this solution, n-Butyllithium (1.1 eq., 2.5M in hexane) was added. After being stirred for 20 minutes at -78 ℃, **S2** (1.0 eq.) was added. The resulting mixture was allowed to warmed up to room temperature for 8 h, and then the reaction was quenched by saturated NH_4Cl (aq.), and extracted three times with ether. The combined organic layer was dried over $MgSO_4$, and the solvent was removed under a reduced pressure. The residue was purified by collum chromatography (petroleum ether/ethyl acetate = 10:1) to afford the desired **S4** (67 - 78%) as an orange oil.

General procedure for preparation of propargylic alcohol (S4).[1d]

In a 25 mL one-neck round bottom flask equipped with magnetic stir bar was placed **S4** (1.0 eq.) and MeOH (1.0 M). To this stirring mixture was added K_2CO_3 (1.0 eq.) and the solution was stirred for 30 min, when TLC indicated the reaction was complete. Water (20 mL) was added and the solution was extracted (3 × 25 mL, CH_2Cl_2), dried ($MgSO_4$) and concentrated under vacuum. The crude product was purified by flash chromatography (petroleum ether/ethyl acetate = 5:1) to yield **S5** (91 - 97%) as an orange oil.

General procedure for preparation of aryl-enynals (1).[1e]

The bromo aldehyde (1.2 eq.) and propargylic alcohol (1.0 eq.) were placed in a clean and anhydrous round-bottom flask equipped with a stir bar, then anhydrous THF and $^i Pr_2NH$ (1.0 eq.)were added under a nitrogen atmosphere, and the flask was cooled to 0 °C. Subsequently, CuI (15 mol%) and $Pd(PPh_3)_2Cl_2$ (1 mol%) were placed in the reaction flask; then after 20 min at 0 °C. Reaction progress was monitored by thin-layer chromatography (TLC) analysis. After complete consumption of the bromo aldehyde, the reaction was quenched with saturated NH_4Cl and extracted with ethyl acetate (EtOAc). The combined organic layer was washed with brine, dried ($MgSO_4$), and concentrated. The crude material was typically purified by flash chromatography using a petroleum ether/ethyl acetate = 5:1 mixture as eluent to yield the enynals **1** (44 - 91%).

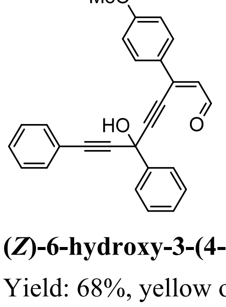

(*Z*)-6-hydroxy-3-(4-methoxyphenyl)-6,8-diphenylocta-2-en-4,7-diynal (1-1)

Yield: 68%, yellow oil, R_f = 0.33 (petroleum ether/AcOEt = 5:1).

^{1}H NMR (500 MHz, CDCl$_3$) δ 10.23 (d, *J* = 8.0 Hz, 1H), 7.91 (dt, *J* = 8.4, 2.3 Hz, 2H), 7.76 – 7.72 (m, 2H), 7.54 – 7.50 (m, 2H), 7.48 – 7.44 (m, 2H), 7.43 – 7.32 (m, 2H), 6.94 – 6.90 (m, 4H), 6.74 (d, *J* = 8.0 Hz, 1H), 3.84 (s, 3H).
^{13}C NMR (126 MHz, CDCl$_3$) δ 193.0, 162.3, 141.2, 140.9, 131.9, 130.2, 129.2, 129.1, 129.0, 128.8, 128.4, 127.5, 125.8, 121.6, 114.4, 101.5, 88.0, 86.3, 66.1, 55.5.
IR (KBr, cm^{-1}) 3730, 2843, 2342, 1732, 1423, 1407 1146, 1092, 826, 546.
HRMS (ESI) ([M+Na]$^+$) Calcd. for [$C_{27}H_{20}NaO_3$]$^+$: 415.1305, Found. 415.1307.

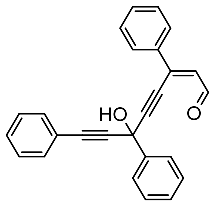

(*Z*)-6-hydroxy-3,6,8-triphenylocta-2-en-4,7-diynal (1-2)
Yield: 78%, yellow oil, R_f = 0.34 (petroleum ether/AcOEt = 5:1).
^{1}H NMR (400 MHz, CDCl$_3$) δ 10.31 (d, *J* = 8.0 Hz, 1H), 8.02 – 7.94 (m, 2H), 7.84 – 7.73 (m, 2H), 7.56 (dd, *J* = 7.6, 1.9 Hz, 2H), 7.53 – 7.47 (m, 3H), 7.45 (d, *J* = 7.3 Hz, 2H), 7.42 (d, *J* = 1.7 Hz, 1H), 7.39 – 7.33 (m, 3H), 6.84 (d, *J* = 8.0 Hz, 1H).
^{13}C NMR (101 MHz, CDCl$_3$) δ 193.4, 141.7, 141.4, 135.0, 132.1, 132.0, 131.4, 129.2, 129.1, 129.0, 128.8, 128.5, 127.3, 125.9, 121.7, 102.3, 88.2, 86.2, 66.0.
IR (KBr, cm^{-1}) 3714, 3521, 2946, 2346, 1633, 1424, 1341, 1081, 992, 857, 553.
HRMS (ESI) ([M+Na]$^+$) Calcd. for [$C_{26}H_{18}NaO_2$]$^+$: 385.1199, Found. 385.1197.

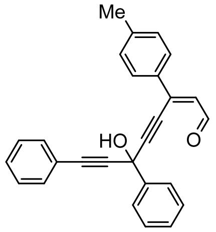

(*Z*)-6-hydroxy-6,8-diphenyl-3-(p-tolyl)octa-2-en-4,7-diynal (1-3)
Yield: 74%, yellow oil, R_f = 0.33 (petroleum ether/AcOEt = 5:1).
^{1}H NMR (400 MHz, CDCl$_3$) δ 10.22 (d, *J* = 8.0 Hz, 1H), 7.91 (dd, *J* = 8.0, 2.2 Hz, 2H), 7.61 (d, *J* = 7.9 Hz, 2H), 7.48 (d, *J* = 7.7 Hz, 2H), 7.42 (t, *J* = 7.6 Hz, 2H), 7.39 – 7.33 (m, 1H), 7.32 – 7.24 (m, 2H), 7.20 (d, *J* = 1.6 Hz, 1H), 7.13 (d, *J* = 8.0 Hz, 2H), 6.74 (d, *J* = 7.9 Hz, 1H), 4.49 (brs, 1H), 2.32 (s, 3H).
^{13}C NMR (101 MHz, CDCl$_3$) δ 193.5, 142.1, 141.8, 141.5, 132.3, 132.0, 131.1, 129.7, 129.2, 129.1, 128.8, 128.5, 127.3, 125.9, 121.8, 88.4, 86.1, 79.7, 66.0, 21.5.
IR (KBr, cm^{-1}) 3719, 3451, 2453, 2246, 1567, 1424, 1325, 1054, 996, 856, 528.
HRMS (ESI) ([M+Na]$^+$) Calcd. for [$C_{27}H_{20}NaO_2$]$^+$: 399.1356, Found. 399.1356.

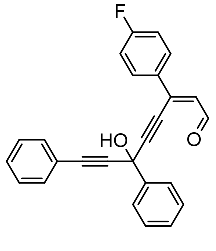

(*Z*)-3-(4-fluorophenyl)-6-hydroxy-6,8-diphenylocta-2-en-4,7-diynal (1-4)
Yield: 71%, yellow oil, R_f = 0.33 (petroleum ether/AcOEt = 5:1).

^{1}H NMR (400 MHz, CDCl$_3$) δ 10.07 (d, *J* = 8.0 Hz, 1H), 7.78 (d, *J* = 7.5 Hz, 2H), 7.57 (t, *J* = 7.1 Hz, 2H), 7.35 (d, *J* = 6.9 Hz, 2H), 7.30 (d, *J* = 7.7 Hz, 1H), 7.25 (d, *J* = 7.1 Hz, 1H), 7.16 (d, *J* = 7.5 Hz, 3H), 7.10 (s, 1H), 6.89 (t, *J* = 8.4 Hz, 2H), 6.58 (d, *J* = 8.0 Hz, 1H), 4.54 (brs, 1H).
^{13}C NMR (101 MHz, CDCl$_3$) δ 193.4, 164.58 (d, *J* = 253.2 Hz), 141.3, 140.6, 132.0, 131.6, 131.13 (d, *J* = 3.3 Hz), 129.44 (d, *J* = 8.7 Hz), 129.20 (d, *J* = 9.3 Hz), 128.9, 128.8, 128.5, 125.9, 121.6, 116.2, 116.0, 102.7, 88.2, 86.2, 79.3, 66.0.
^{19}F NMR (376 MHz, CDCl$_3$) δ -107.81.
IR (KBr, cm^{-1}) 3789, 3345, 2356, 2152, 1538, 1436, 1275, 1153, 974, 736, 628.
HRMS (ESI) ([M+Na]$^+$) Calcd. for [$C_{26}H_{17}FNaO_2$]$^+$: 403.1105, Found. 403.1107.

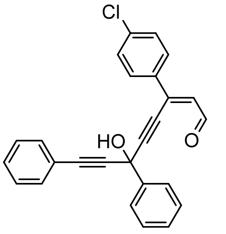

(*Z*)-3-(4-chlorophenyl)-6-hydroxy-6,8-diphenylocta-2-en-4,7-diynal (1-5)
Yield: 63%, yellow oil, R_f = 0.35 (petroleum ether/AcOEt = 5:1).
^{1}H NMR (400 MHz, CDCl$_3$) δ 10.27 (d, J = 7.9 Hz, 1H), 7.94 (d, J = 7.6 Hz, 2H), 7.69 (d, J = 8.2 Hz, 2H), 7.54 (d, J = 7.1 Hz, 2H), 7.47 (dt, J = 14.8, 7.0 Hz, 3H), 7.41 – 7.33 (m, 5H), 6.79 (d, J = 7.9 Hz, 1H), 4.08 (brs, 1H).
^{13}C NMR (101 MHz, CDCl$_3$) δ 193.1, 141.2, 140.2, 137.5, 133.5, 132.1, 131.9, 129.3, 129.2, 128.9, 128.5, 125.8, 121.5, 102.4, 88.0, 86.4, 79.2, 66.0.
IR (KBr, cm^{-1}) 3736, 3621, 2571, 2351, 2108, 1531, 1428, 1361, 937, 852, 578.
HRMS (ESI) ([M+Na]$^+$) Calcd. for [$C_{26}H_{17}ClNaO_2$]$^+$: 419.0809, Found. 419.0809.

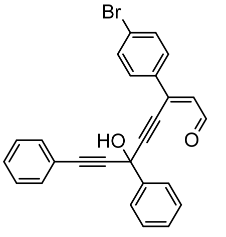

(*Z*)-3-(4-bromophenyl)-6-hydroxy-6,8-diphenylocta-2-en-4,7-diynal (1-6)
Yield: 61%, yellow oil, R_f = 0.35 (petroleum ether/AcOEt = 3:1).
^{1}H NMR (400 MHz, CDCl$_3$) δ 10.26 (d, *J* = 7.9 Hz, 1H), 7.93 (d, *J* = 7.6 Hz, 2H), 7.61 (d, *J* = 8.3 Hz, 2H), 7.56 – 7.46 (m, 6H), 7.46 – 7.40 (m, 1H), 7.36 (q, *J* = 6.9, 6.2 Hz, 3H), 6.78 (d, *J* = 7.9 Hz, 1H), 4.22 (brs, 1H).
^{13}C NMR (101 MHz, CDCl$_3$) δ 193.1, 141.2, 140.3, 133.9, 132.2, 132.1, 131.9, 129.3, 129.2, 128.9, 128.7, 128.5, 126.0, 125.8, 121.5, 102.5, 88.0, 86.4, 79.1, 66.0.
IR (KBr, cm^{-1}) 3718, 3581, 2461, 2379, 2158, 1731, 1478, 1157, 847, 531.
HRMS (ESI) ([M+Na]$^+$) Calcd. for [$C_{26}H_{17}BrNaO_2$]$^+$: 463.0304, Found. 463.0299.

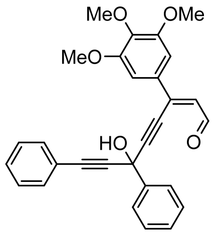

(*Z*)-6-hydroxy-6,8-diphenyl-3-(3,4,5-trimethoxyphenyl)octa-2-en-4,7-diynal (1-7)

Yield: 73%, yellow oil, R_f = 0.31 (petroleum ether/AcOEt = 3:1).

^{1}H NMR (400 MHz, $CDCl_3$) δ 10.26 (d, J = 8.0 Hz, 1H), 7.93 (d, J = 7.6 Hz, 2H), 7.42 (dd, J = 19.8, 7.5 Hz, 4H), 7.35 (d, J = 7.2 Hz, 1H), 7.29 – 7.24 (m, 3H), 6.94 (s, 2H), 6.75 (d, J = 8.0 Hz, 1H), 5.26 (brs, 1H), 3.85 (s, 3H), 3.70 (s, 6H).

^{13}C NMR (101 MHz, $CDCl_3$) δ 193.5, 153.1, 141.7, 141.5, 140.8, 131.8, 130.9, 130.3, 129.1, 128.9, 128.7, 128.44, 128.40, 125.8, 121.7, 104.6, 102.6, 88.5, 85.8, 65.7, 60.9, 56.0.

IR (KBr, cm^{-1}) 3719, 3431, 2579, 2341, 2205, 1891, 1544, 1295, 951, 837, 569.

HRMS (ESI) ([M+Na]$^+$) Calcd. for [$C_{29}H_{24}NaO_5$]$^+$: 475.1516, Found. 475.1520.

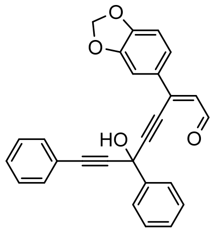

(*Z*)-3-(benzo[d][1,3]dioxol-5-yl)-6-hydroxy-6,8-diphenylocta-2-en-4,7-diynal (1-8)

Yield: 48%, yellow oil, R_f = 0.37 (petroleum ether/AcOEt = 3:1).

^{1}H NMR (400 MHz, $CDCl_3$) δ 10.24 (d, J = 8.1 Hz, 1H), 7.94 (d, J = 7.4 Hz, 2H), 7.54 (d, J = 6.5 Hz, 2H), 7.49 (t, J = 7.4 Hz, 2H), 7.45 – 7.32 (m, 5H), 7.21 (s, 1H), 6.83 (dd, J = 8.0, 4.9 Hz, 1H), 6.70 (d, J = 8.0 Hz, 1H), 6.03 (d, J = 4.5 Hz, 2H).

^{13}C NMR (101 MHz, $CDCl_3$) δ 193.2, 150.5, 148.4, 141.3, 141.0, 132.0, 130.6, 130.5, 129.3, 129.2, 129.1, 128.8, 128.4, 125.8, 123.0, 121.6, 108.6, 106.8, 101.9, 88.1, 86.2, 79.6, 66.0.

IR (KBr, cm^{-1}) 3728, 3624, 2781, 2576, 2301, 1954, 1725, 1417, 936, 871, 580.

HRMS (ESI) ([M+Na]$^+$) Calcd. for [$C_{27}H_{18}NaO_4$]$^+$: 429.1097, Found. 429.1094.

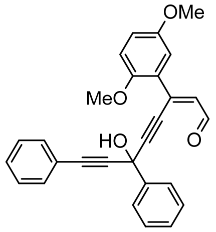

(*E*)-3-(2,5-dimethoxyphenyl)-6-hydroxy-6,8-diphenylocta-2-en-4,7-diynal (1-9)

Yield: 73%, yellow oil, R_f = 0.31 (petroleum ether/AcOEt = 5:1).

^{1}H NMR (400 MHz, $CDCl_3$) δ 10.31, 9.40 (d, J = 8.3 Hz, 1H), 7.90 (d, J = 7.2 Hz, 2H), 7.49 – 7.19 (m, 9H), 7.14, 6.46 (d, J = 8.1 Hz, 1H), 6.85 (d, J = 8.1 Hz, 1H), 6.75 (d, J = 8.9 Hz, 1H), 5.00 (brs, 1H), 3.68 (s, 3H), 3.58 (s, 3H).

^{13}C NMR (101 MHz, $CDCl_3$) δ 194.7, 193.0, 153.3, 153.2, 152.5, 150.7, 141.7, 139.0, 138.4, 136.4, 135.2, 131.9, 131.9, 129.1, 129.0, 129.0, 128.9, 128.8, 128.6, 128.5, 128.4, 126.05, 125.95, 124.6, 123.8,

121.9, 121.8, 117.8, 116.7, 116.5, 115.8, 113.1, 113.0, 100.8, 99.4, 88.72, 88.66, 85.8, 85.7, 84.5, 80.4, 65.9, 65.8, 56.3, 56.1, 55.8, 55.7.

IR (KBr, cm^{-1}) 3716, 3531, 2578, 2247, 2028, 1951, 1738, 1416, 928, 529.

HRMS (ESI) ([M+Na]$^+$) Calcd. for $[C_{28}H_{22}NaO_4]^+$: 445.1410, Found. 445.1411.

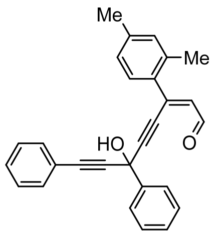

(*E*)-3-(2,4-dimethylphenyl)-6-hydroxy-6,8-diphenylocta-2-en-4,7-diynal (1-10)
Yield: 75%, yellow oil, R_f = 0.33 (petroleum ether/AcOEt = 5:1).
^{1}H NMR (400 MHz, CDCl$_3$) δ 10.22, 9.37 (d, *J* = 8.1 Hz, 1H), 7.82 (t, *J* = 9.1 Hz, 2H), 7.45 (t, *J* = 7.1 Hz, 2H), 7.41 – 7.25 (m, 6H), 7.19 (d, *J* = 13.3 Hz, 1H), 7.00 (p, *J* = 8.4 Hz, 2H), 6.52, 6.36 (d, *J* = 8.2 Hz, 1H), 4.00 (brs, 1H), 2.39 (s, 3H), 2.30 (s, 3H).
^{13}C NMR (101 MHz, CDCl$_3$) δ 193.1, 192.7, 143.9, 143.4, 141.3, 141.2, 140.0, 139.7, 136.9, 136.1, 136.0, 135.9, 134.0, 132.1, 132.0, 131.9, 131.7, 130.7, 130.4, 129.2, 129.1, 129.05, 128.99, 128.83, 128.77, 128.7, 128.5, 128.4, 127.1, 126.60, 125.97, 125.9, 121.8, 121.7, 101.9, 101.4, 88.2, 88.1, 86.2, 86.0, 83.9, 80.6, 21.3, 20.6, 20.0.

IR (KBr, cm^{-1}) 3801, 3578, 2357, 2145, 2091, 1834, 1725, 1519, 951, 578.

HRMS (ESI) ([M+Na]$^+$) Calcd. for $[C_{28}H_{22}NaO_2]^+$: 413.1512, Found. 413.1513.

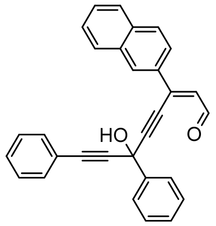

(*Z*)-6-hydroxy-3-(naphthalen-2-yl)-6,8-diphenylocta-2-en-4,7-diynal (1-11)
Yield: 51%, yellow oil, R_f = 0.31 (petroleum ether/AcOEt = 5:1).
^{1}H NMR (400 MHz, CDCl$_3$) δ 10.37 (d, *J* = 8.0 Hz, 1H), 8.32 (s, 1H), 8.02 (d, *J* = 7.9 Hz, 2H), 7.88 – 7.72 (m, 4H), 7.54 (m, 7H), 7.38 (d, *J* = 6.9 Hz, 3H), 6.95 (d, *J* = 7.9 Hz, 1H), 4.08 (brs, 1H).
^{13}C NMR (101 MHz, CDCl$_3$) δ 193.3, 141.3, 134.6, 133.0, 132.3, 132.0, 129.24, 129.19, 129.16, 128.9, 128.7, 128.5, 127.9, 127.7, 126.9, 125.9, 122.9, 121.6, 102.1, 88.2, 86.3, 79.7, 66.1.

IR (KBr, cm^{-1}) 3731, 2917, 2368, 1782, 1581, 1392, 1204, 1047, 948, 873, 572.

HRMS (ESI) ([M+Na]$^+$) Calcd. for $[C_{30}H_{20}NaO_2]^+$: 435.1356, Found. 435.1354.

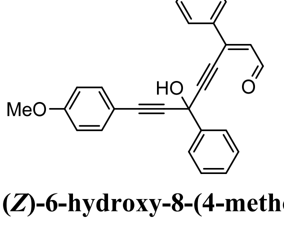

(*Z*)-6-hydroxy-8-(4-methoxyphenyl)-3,6-diphenylocta-2-en-4,7-diynal (1-12)
Yield: 73%, yellow oil, R_f = 0.34 (petroleum ether/AcOEt = 5:1).

^{1}H NMR (400 MHz, $CDCl_3$) δ 10.23 (d, J = 7.8 Hz, 1H), 7.91 (d, J = 7.4 Hz, 2H), 7.70 (d, J = 6.9 Hz, 2H), 7.46 – 7.29 (m, 8H), 6.79 (d, J = 8.1 Hz, 2H), 6.75 (d, J = 8.3 Hz, 1H), 4.71 (brs, 1H), 3.71 (s, 3H).
^{13}C NMR (101 MHz, $CDCl_3$) δ 193.5, 160.2, 141.9, 141.6, 135.0, 133.5, 131.9, 129.02, 128.96, 128.8, 127.3, 125.9, 114.2, 113.8, 102.8, 87.2, 86.2, 79.4, 66.0, 55.4.

IR (KBr, cm^{-1}) 3571, 2815, 2351, 1915, 1587, 1405, 1284, 1158, 959, 836, 529.

HRMS (ESI) ($[M+Na]^+$) Calcd. for $[C_{27}H_{20}NaO_3]^+$: 415.1305, Found. 415.1303.

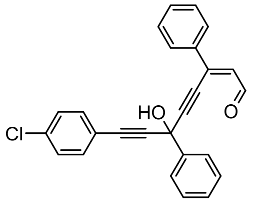

(*Z*)-8-(4-chlorophenyl)-6-hydroxy-3,6-diphenylocta-2-en-4,7-diynal (1-13)
Yield: 75%, yellow oil, R_f = 0.33 (petroleum ether/AcOEt = 5:1).
^{1}H NMR (400 MHz, $CDCl_3$) δ 10.23 (d, J = 8.0 Hz, 1H), 7.88 (d, J = 7.6 Hz, 2H), 7.70 (d, J = 7.8 Hz, 2H), 7.39 (m, 7H), 7.28 – 7.21 (m, 3H), 6.77 (d, J = 8.0 Hz, 1H), 4.35 (s, 1H).
^{13}C NMR (101 MHz, $CDCl_3$) δ 193.4, 141.7, 141.1, 135.3, 134.9, 133.2, 132.1, 131.4, 129.2, 129.0, 128.9, 128.8, 127.3, 125.8, 120.1, 102.1, 89.2, 85.0, 79.7, 66.0.

IR (KBr, cm^{-1}) 3513, 2941, 2573, 1936, 1605, 1371, 1257, 1153, 961, 531.

HRMS (ESI) ($[M+H]^+$) Calcd. for $[C_{26}H_{18}ClO_2]^+$: 397.0990, Found. 397.0986.

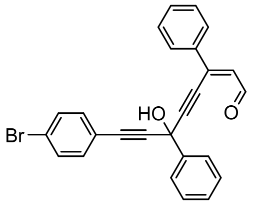

(*Z*)-8-(4-bromophenyl)-6-hydroxy-3,6-diphenylocta-2-en-4,7-diynal (1-14)
Yield: 48%, yellow oil, R_f = 0.31 (petroleum ether/AcOEt = 3:1).
^{1}H NMR (500 MHz, $CDCl_3$) δ 10.23 (d, J = 8.0 Hz, 1H), 7.88 (d, J = 7.4 Hz, 2H), 7.71 (d, J = 7.3 Hz, 2H), 7.46 – 7.30 (m, 10H), 6.77 (d, J = 8.0 Hz, 1H), 4.39 (brs, 1H).
^{13}C NMR (126 MHz, $CDCl_3$) δ 193.3, 141.6, 141.1, 134.9, 133.4, 132.1, 131.8, 131.4, 129.2, 129.0, 128.9, 127.3, 125.8, 123.6, 120.6, 102.0, 89.4, 85.0, 79.7, 66.0.
IR (KBr, cm^{-1}) 3857, 2962, 2212, 1661, 1585, 1486, 1405, 1198, 1143, 1092, 826.
HRMS (ESI) ($[M+H]^+$) Calcd. for $[C_{26}H_{18}BrO_2]^+$: 441.0485, Found. 441.0480.

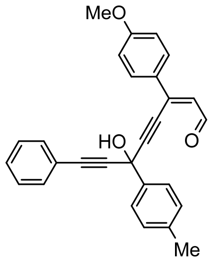

(*Z*)-6-hydroxy-3,8-diphenyl-6-(p-tolyl)octa-2-en-4,7-diynal (1-15)
Yield: 67%, yellow oil, R_f = 0.35 (petroleum ether/AcOEt = 3:1).

^{1}H NMR (500 MHz, CDCl$_3$) δ 10.19 (d, *J* = 8.0 Hz, 1H), 7.78 (d, *J* = 7.8 Hz, 2H), 7.69 (d, *J* = 8.4 Hz, 2H), 7.48 (d, *J* = 6.9 Hz, 2H), 7.33 – 7.28 (m, 2H), 7.25 – 7.19 (m, 3H), 6.85 (d, *J* = 8.4 Hz, 2H), 6.69 (d, *J* = 8.0 Hz, 1H), 3.77 (s, 3H), 2.35 (s, 3H).
^{13}C NMR (126 MHz, CDCl$_3$) δ 193.5, 162.3, 141.4, 139.0, 138.6, 132.0, 129.9, 129.5, 129.1, 129.1, 128.4, 125.8, 121.8, 114.4, 102.1, 88.5, 85.9, 79.5, 65.9, 55.5, 21.2.
IR (KBr, cm^{-1}) 3812, 3451, 2517, 2359, 2146, 1925, 1848, 1446, 987, 593.
HRMS (ESI) ([M+H]$^+$) Calcd. for [$C_{28}H_{23}O_3$]$^+$: 407.1642, Found. 407.1641.

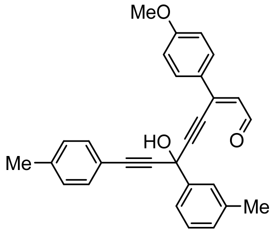

(*Z*)-6-hydroxy-3-phenyl-6-(m-tolyl)-8-(p-tolyl)octa-2-en-4,7-diynal (1-16)
Yield: 63%, yellow oil, R_f = 0.35 (petroleum ether/AcOEt = 5:1).
^{1}H NMR (400 MHz, CDCl$_3$) δ 10.20 (d, *J* = 8.0 Hz, 1H), 7.73 – 7.66 (m, 4H), 7.38 (d, *J* = 8.0 Hz, 2H), 7.31 (t, *J* = 7.6 Hz, 1H), 7.17 (d, *J* = 7.4 Hz, 1H), 7.09 (d, *J* = 7.9 Hz, 2H), 6.84 (d, *J* = 8.8 Hz, 2H), 6.69 (d, *J* = 8.1 Hz, 1H), 4.32 (s, 1H), 3.77 (s, 3H), 2.39 (s, 3H), 2.31 (s, 3H).
^{13}C NMR (101 MHz, CDCl$_3$) δ 193.4, 162.2, 141.4, 141.3, 139.3, 138.5, 131.9, 129.9, 129.8, 129.2, 129.1, 128.7, 127.5, 126.5, 123.0, 118.7, 114.3, 102.3, 87.8, 86.2, 79.5, 66.0, 55.51, 55.47, 21.6.
IR (KBr, cm^{-1}) 3725, 3045, 2971, 2813, 1543, 1237, 1152, 1026, 726, 593.
HRMS (ESI) ([M+H]$^+$) Calcd. for [$C_{29}H_{25}O_3$]$^+$: 421.1798, Found. 421.1794.

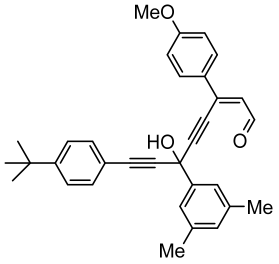

(*Z*)-8-(4-(tert-butyl)phenyl)-6-(3,5-dimethylphenyl)-6-hydroxy-3-phenylocta-2-en-4,7-diynal (1-17)
Yield: 72%, yellow oil, R_f = 0.35 (petroleum ether/AcOEt = 5:1).
^{1}H NMR (500 MHz, CDCl$_3$) δ 10.23 (d, *J* = 8.0 Hz, 1H), 7.71 (d, *J* = 8.9 Hz, 2H), 7.52 (s, 2H), 7.45 (d, *J* = 8.6 Hz, 2H), 7.33 (d, *J* = 8.5 Hz, 2H), 7.00 (s, 1H), 6.86 (d, *J* = 7.9 Hz, 2H), 6.71 (d, *J* = 8.0 Hz, 1H), 3.78 (s, 3H), 2.35 (s, 6H), 1.29 (s, 9H).
^{13}C NMR (126 MHz, CDCl$_3$) δ 193.4, 162.2, 152.4, 141.3, 138.4, 131.7, 130.7, 130.0, 129.9, 129.1, 127.6, 125.4, 123.7, 118.8, 114.3, 102.4, 87.9, 86.1, 79.4, 66.0, 55.5, 34.9, 31.2, 21.5.
IR (KBr, cm^{-1}) 3745, 2956, 2867, 1668, 1552, 1463, 1392, 1228, 1102, 974, 859, 743.
HRMS (ESI) ([M+H]$^+$) Calcd. for [$C_{33}H_{33}O_3$]$^+$: 477.2424, Found. 477.2427.

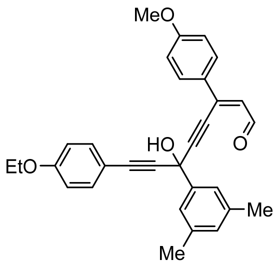

(Z)-6-(3,5-dimethylphenyl)-8-(4-ethoxyphenyl)-6-hydroxy-3-(4-methoxyphenyl)octa-2-en-4,7-diynal (1-18)

Yield: 69%, yellow oil, R_f = 0.35 (petroleum ether/AcOEt = 5:1).

^{1}H NMR (500 MHz, CDCl$_3$) δ 10.22 (d, *J* = 8.0 Hz, 1H), 7.68 (d, *J* = 8.5 Hz, 2H), 7.53 (s, 2H), 7.40 (d, *J* = 8.5 Hz, 2H), 6.98 (s, 1H), 6.83 (d, *J* = 8.4 Hz, 2H), 6.78 (d, *J* = 8.2 Hz, 2H), 6.69 (d, *J* = 8.0 Hz, 1H), 4.66 (s, 1H), 3.94 (q, *J* = 7.3, 6.5 Hz, 2H), 3.75 (s, 3H), 2.34 (s, 6H), 1.35 (t, *J* = 6.6 Hz, 3H).

^{13}C NMR (126 MHz, CDCl$_3$) δ 193.5, 162.2, 159.5, 141.52, 141.47, 138.3, 133.5, 130.6, 129.8, 129.1, 127.5, 123.7, 114.6, 114.3, 113.7, 102.7, 87.4, 86.0, 79.3, 65.9, 63.6, 55.5, 21.5, 14.7.

IR (KBr, cm^{-1}) 3855, 3742, 3623, 3020, 2925, 2855, 1667, 1461, 1285, 1148, 1022, 797, 690.

HRMS (ESI) ([M+H]$^+$) Calcd. for [$C_{31}H_{29}O_4$]$^+$: 465.2060, Found. 465.2066.

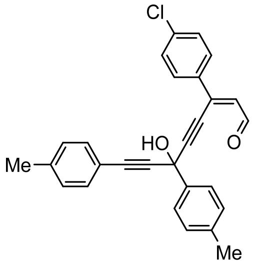

(*Z*)-3-(4-chlorophenyl)-6-hydroxy-6,8-di-p-tolylocta-2-en-4,7-diynal (1-19)

Yield: 51%, yellow oil, R_f = 0.35 (petroleum ether/AcOEt = 5:1).

^{1}H NMR (400 MHz, CDCl$_3$) δ 10.28 (d, *J* = 7.9 Hz, 1H), 7.78 – 7.64 (m, 4H), 7.45 – 7.35 (m, 5H), 7.25 (d, *J* = 7.5 Hz, 1H), 7.17 (d, *J* = 7.9 Hz, 2H), 6.78 (d, *J* = 7.9 Hz, 1H), 3.76 (s, 1H), 2.45 (s, 3H), 2.39 (s, 3H).

^{13}C NMR (101 MHz, CDCl$_3$) δ 193.0, 141.1, 140.1, 139.5, 138.6, 137.4, 133.6, 132.1, 131.8, 129.9, 129.22, 129.18, 128.7, 128.5, 126.4, 122.9, 118.5, 102.6, 87.4, 86.5, 79.0, 66.1, 21.58, 21.57.

IR (KBr, cm^{-1}) 3856, 3742, 2924, 1702, 1506, 1448, 1051, 793, 593.

HRMS (ESI) ([M+Na]$^+$) Calcd. for [$C_{28}H_{21}ClNaO_2$]$^+$: 447.1122, Found. 447.1114.

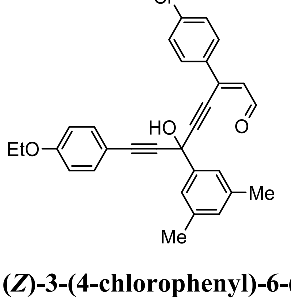

(*Z*)-3-(4-chlorophenyl)-6-(3,5-dimethylphenyl)-8-(4-ethoxyphenyl)-6-hydroxyocta-2-en-4,7-diynal (1-20)

Yield: 71%, yellow oil, R_f = 0.35 (petroleum ether/AcOEt = 2:1).

^{1}H NMR (500 MHz, CDCl$_3$) δ 10.23 (d, *J* = 7.9 Hz, 1H), 7.64 (d, *J* = 8.4 Hz, 2H), 7.50 (s, 2H), 7.40 (d, *J* = 8.4 Hz, 2H), 7.30 (d, *J* = 8.3 Hz, 2H), 6.99 (s, 1H), 6.80 (d, *J* = 8.4 Hz, 2H), 6.72 (d, *J* = 7.9 Hz, 1H), 4.36 (brs, 1H), 3.97 (q, *J* = 7.0, 6.5 Hz, 2H), 2.35 (s, 6H), 1.38 (t, *J* = 6.8 Hz, 3H).

^{13}C NMR (126 MHz, CDCl$_3$) δ 193.3, 159.6, 141.3, 140.4, 138.4, 137.4, 133.6, 133.5, 131.9, 130.7, 129.1, 128.5, 123.6, 114.6, 113.5, 103.2, 87.1, 86.3, 78.7, 66.0, 63.6, 21.5, 14.7.

IR (KBr, cm^{-1}) 3813, 3751, 3062, 2961, 2866, 2212, 1656, 1561, 1440, 1140, 996, 815.

HRMS (ESI) ([M+H]$^+$) Calcd. for [$C_{30}H_{26}ClO_3$]$^+$: 469.1565, Found. 469.1561.

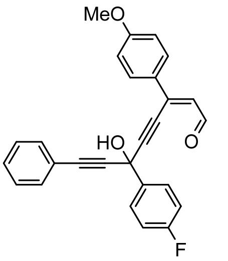

(*Z*)-6-(4-fluorophenyl)-6-hydroxy-3-(4-methoxyphenyl)-8-phenylocta-2-en-4,7-diynal (1-21)

Yield: 55%, yellow oil, R_f = 0.33 (petroleum ether/AcOEt = 5:1).

^{1}H NMR (500 MHz, CDCl$_3$) δ 10.21 (d, *J* = 8.0 Hz, 1H), 7.90 – 7.86 (m, 2H), 7.71 (dd, *J* = 9.0, 1.0 Hz, 2H), 7.53 – 7.49 (m, 2H), 7.36 (m, 3H), 7.13 (t, *J* = 8.7 Hz, 2H), 6.90 (d, *J* = 8.9 Hz, 2H), 6.74 (d, *J* = 8.1 Hz, 1H), 3.83 (s, 3H).

^{13}C NMR (126 MHz, CDCl$_3$) δ 193.1, 163.04 (d, $J_{C\text{-}F}$ = 248.4 Hz), 162.1, 140.89 (d, $J_{C\text{-}F}$ = 3.4 Hz), 131.9, 130.2, 129.3, 129.0, 128.5, 127.87 (d, $J_{C\text{-}F}$ = 8.6 Hz), 127.3, 121.4, 115.66 (d, $J_{C\text{-}F}$ = 21.8 Hz), 114.4, 101.4, 87.9, 86.3, 79.9, 65.4, 55.5.

^{19}F NMR (471 MHz, CDCl$_3$) δ -112.62.

IR (KBr, cm^{-1}) 3736, 3014, 2935, 2815, 2027, 1751, 1601, 1127, 887, 562.

HRMS (ESI) ([M+Na]$^+$) Calcd. for [$C_{27}H_{19}FNaO_3$]$^+$: 433.1210, Found. 433.1214.

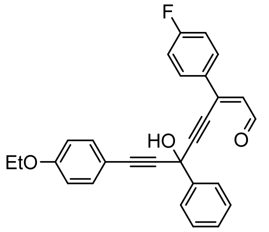

(*Z*)-8-(4-ethoxyphenyl)-3-(4-fluorophenyl)-6-hydroxy-6-phenylocta-2-en-4,7-diynal (1-22)

Yield: 57%, yellow oil, R_f = 0.33 (petroleum ether/AcOEt = 3:1).

^{1}H NMR (500 MHz, CDCl$_3$) δ 10.21 (d, *J* = 7.9 Hz, 1H), 7.92 – 7.87 (m, 2H), 7.75 – 7.67 (m, 2H), 7.47 – 7.34 (m, 5H), 7.08 – 7.00 (m, 2H), 6.84 – 6.78 (m, 2H), 6.71 (d, *J* = 8.0 Hz, 1H), 4.18 (brs, 1H), 3.99 (q, *J* = 7.0 Hz, 2H), 1.39 (t, *J* = 7.0 Hz, 3H).

^{13}C NMR (126 MHz, CDCl$_3$) δ 193.2, 164.54 (d, $J_{C\text{-}F}$ = 253.2 Hz), 159.7, 141.4, 140.4, 133.5, 131.19 (d, $J_{C\text{-}F}$ = 3.4 Hz), 131.2, 129.37 (d, $J_{C\text{-}F}$ = 8.7 Hz), 129.1, 128.8, 125.8, 116.07 (d, $J_{C\text{-}F}$ = 21.9 Hz), 114.6, 113.4, 102.7, 86.8, 86.5, 79.2, 66.0, 63.6, 14.7.

^{19}F NMR (471 MHz, CDCl$_3$) δ -108.08.

IR (KBr, cm^{-1}) 3813, 3758, 3017, 2936, 2819, 2161, 1615, 1534, 1147, 936, 818, 538.

HRMS (ESI) ([M+Na]$^+$) Calcd. for [$C_{28}H_{21}FNaO_3$]$^+$: 447.1367, Found. 447.1362.

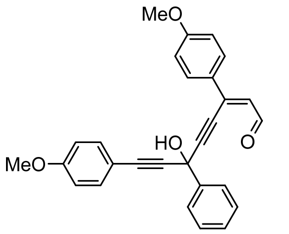

(*Z*)-6-hydroxy-3,8-bis(4-methoxyphenyl)-6-phenylocta-2-en-4,7-diynal (1-23)

Yield: 73%, yellow oil, R_f = 0.33 (petroleum ether/AcOEt = 3:1).

^{1}H NMR (500 MHz, $CDCl_3$) δ 10.18 (d, *J* = 8.1 Hz, 1H), 7.93 – 7.90 (m, 2H), 7.67 – 7.63 (m, 2H), 7.44 – 7.38 (m, 4H), 7.37 – 7.31 (m, 1H), 6.83 – 6.76 (m, 4H), 6.68 (d, *J* = 8.1 Hz, 1H), 3.73 (s, 3H), 3.70 (s, 3H).

^{13}C NMR (126 MHz, $CDCl_3$) δ 193.5, 162.3, 160.2, 141.8, 141.5, 133.5, 129.8, 129.1, 129.0, 128.8, 127.4, 125.9, 114.4, 114.1, 113.8, 102.5, 87.3, 86.2, 79.5, 66.0, 55.5, 55.3.

IR (KBr, cm^{-1}) 3855, 3741, 3059, 2927, 2856, 2211, 1666, 1506, 1143, 1046, 880, 620.

HRMS (ESI) ([M+H]$^+$) Calcd. for [$C_{28}H_{23}O_4$]$^+$: 423.1591, Found. 423.1598.

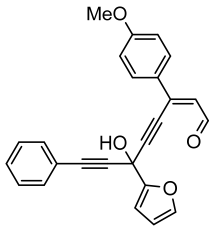

(*Z*)-6-(furan-2-yl)-6-hydroxy-3-(4-methoxyphenyl)-8-phenylocta-2-en-4,7-diynal (1-24)

Yield: 51%, yellow oil, R_f = 0.31 (petroleum ether/AcOEt = 3:1).

^{1}H NMR (400 MHz, $CDCl_3$) δ 10.27 (d, *J* = 8.0 Hz, 1H), 7.77 (d, *J* = 8.7 Hz, 2H), 7.54 (d, *J* = 8.0 Hz, 3H), 7.36 (d, *J* = 7.5 Hz, 3H), 6.92 (d, *J* = 8.7 Hz, 2H), 6.77 (d, *J* = 8.0 Hz, 1H), 6.72 (d, *J* = 2.8 Hz, 1H), 6.44 (dd, *J* = 3.5, 1.8 Hz, 1H), 4.43 (brs, 1H), 3.84 (s, 3H).

^{13}C NMR (101 MHz, $CDCl_3$) δ 193.5, 162.3, 152.1, 143.7, 141.0, 132.0, 130.2, 129.3, 129.1, 128.4, 127.2, 121.4, 114.4, 110.8, 108.2, 99.0, 85.8, 85.2, 79.0, 61.0, 55.5.

IR (KBr, cm^{-1}) 3718, 3035, 2815, 2736, 1568, 1251, 1163, 1037, 736, 578.

HRMS (ESI) ([M+Na]$^+$) Calcd. for [$C_{25}H_{18}NaO_4$]$^+$: 405.1097, Found. 405.1103.

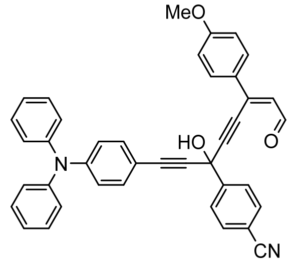

(Z)-4-(1-(4-(diphenylamino)phenyl)-3-hydroxy-6-(4-methoxyphenyl)-8-oxoocta-6-en-1,4-diyn-3-yl)benzonitrile (1-25)

Yield: 73%, yellow oil, R_f = 0.31 (petroleum ether/AcOEt = 3:1).

^{1}H NMR (500 MHz, $CDCl_3$) δ 10.19 (d, *J* = 8.0 Hz, 1H), 8.02 – 7.97 (m, 2H), 7.74 – 7.71 (m, 2H), 7.69 – 7.66 (m, 2H), 7.33 – 7.30 (m, 2H), 7.29 (d, *J* = 1.6 Hz, 1H), 7.27 (d, *J* = 1.4 Hz, 2H), 7.26 (s, 1H), 7.11 – 7.05 (m, 6H), 6.96 (d, *J* = 8.8 Hz, 2H), 6.91 (d, *J* = 8.8 Hz, 2H), 6.74 (d, *J* = 8.0 Hz, 1H), 4.01 (brs, 1H), 3.84 (s, 3H).

^{13}C NMR (126 MHz, CDCl$_3$) δ 192.8, 162.4, 149.1, 146.9, 146.3, 140.6, 132.9, 132.6, 130.4, 129.5, 128.9, 127.2, 126.7, 125.3, 124.0, 121.6, 118.4, 114.4, 113.1, 112.8, 100.7, 87.6, 86.3, 80.3, 65.4, 55.5.
IR (KBr, cm^{-1}) 3034, 2202, 1675, 1585, 1512, 1486, 1328, 1284, 872, 651.
HRMS (ESI) ([M+H]$^+$) Calcd. for [$C_{40}H_{29}N_2O_3$]$^+$: 585.2173, Found. 585.2167.

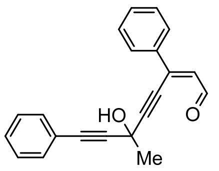

(*Z*)-6-hydroxy-6-methyl-3,8-diphenylocta-2-en-4,7-diynal (1-26)
Yield: 87%, yellow oil, R_f = 0.37 (petroleum ether/AcOEt = 3:1).
^{1}H NMR (400 MHz, CDCl$_3$) δ 10.29 (d, *J* = 8.0 Hz, 1H), 7.77 (d, *J* = 7.5 Hz, 2H), 7.50 – 7.38 (m, 5H), 7.37 – 7.27 (m, 3H), 6.80 (d, *J* = 8.0 Hz, 1H), 3.25 (s, 1H), 2.01 (s, 3H).
^{13}C NMR (101 MHz, CDCl$_3$) δ 193.2, 141.5, 135.1, 132.0, 131.9, 131.3, 129.0, 128.9, 128.4, 127.2, 121.7, 102.9, 89.0, 83.7, 60.9, 31.6.
IR (KBr, cm^{-1}) 3736, 3014, 2947, 2736, 2013, 1666, 1471, 1038, 815, 579.
HRMS (ESI) ([M+Na]$^+$) Calcd. for [$C_{21}H_{16}NaO_2$]$^+$: 323.1043, Found. 323.1044.

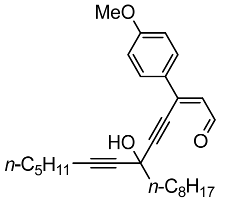

(Z)-6-(hept-1-yn-1-yl)-6-hydroxy-3-(4-methoxyphenyl)tetradec-2-en-4-ynal (1-27)
Yield: 62%, yellow oil, R_f = 0.35 (petroleum ether/AcOEt = 3:1).
^{1}H NMR (500 MHz, CDCl$_3$) δ 10.21 (d, *J* = 8.0 Hz, 1H), 7.78 – 7.66 (m, 2H), 7.00 – 6.83 (m, 2H), 6.71 (d, *J* = 8.1 Hz, 1H), 3.84 (s, 3H), 3.52 (s, 1H), 2.25 (t, *J* = 7.1 Hz, 2H), 2.09 – 1.95 (m, 2H), 1.65 (td, *J* = 7.2, 6.6, 3.4 Hz, 2H), 1.54 (p, *J* = 7.1 Hz, 2H), 1.44 – 1.22 (m, 14H), 0.88 (q, *J* = 7.3 Hz, 6H).
^{13}C NMR (126 MHz, CDCl$_3$) δ 193.4, 162.2, 141.4, 129.6, 128.9, 127.6, 114.2, 103.1, 85.5, 80.1, 64.2, 55.4, 43.9, 31.8, 31.0, 29.5, 29.3, 29.2, 28.1, 24.8, 22.6, 22.1, 18.6, 14.1, 13.9.
IR (KBr, cm^{-1}) 3405, 3117, 2859, 2359, 1641, 1509, 1399, 1084, 991, 841, 558.
HRMS (ESI) ([M+H]$^+$) Calcd. for [$C_{28}H_{39}O_3$]$^+$: 423.2894, Found. 423.2888.

Synthesis of methyl substituted substrates.

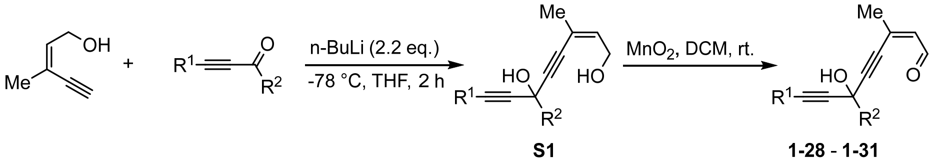

General procedure for preparation of propargylic alcohol (S1).

(Z)-3-Methylpent-2-en-4-yn-1-ol (1.0 eq) was dissolved into THF (0.5 M), and the solution was cooled to -78 ℃. To this solution, n-Butyllithium (2.2 eq., 2.5M in hexane) was added. After being stirred for 20 minutes at -78 ℃, alkynone (1.2 eq.) was added. The resulting mixture was allowed to warmed up to room temperature for 8 h, and then the reaction was quenched by saturated NH_4Cl (aq.), and extracted three times with ether. The combined organic layer was dried over $MgSO_4$, and the solvent was removed

under a reduced pressure. The residue was purified by collum chromatography (petroleum ether/ethyl acetate = 2:1) to afford the desired **S1** (43 - 50%) as an orange oil.

General procedure for preparation of enynals (1).

The **S1** (0.5 mmol) was dissolved in dichloromethane and treated with MnO_2 (870 mg, 20 eq.). After stirring at room temperature over 2 h, the reaction was complete as determined by TLC. Excess MnO_2 was removed by filtration of the reaction mixture through a pad of celite. The filtrate was washed sequentially with brine and dried over $MgSO_4$. The solvent was removed under reduced pressure, and the residue was purified by column chromatography on silica gel and eluted with petroleum ether/ethyl acetate (10/1) to afford the desired product **1-28** – **1-30**.

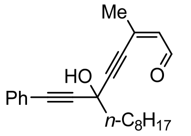

(Z)-6-hydroxy-3-methyl-6-(phenylethynyl)tetradec-2-en-4-ynal (1-28)

Yield: 81%, yellow oil, R_f = 0.33 (petroleum ether/AcOEt = 3:1).

^{1}H NMR (500 MHz, CDCl$_3$) δ 10.03 (d, J = 8.2 Hz, 1H), 7.49 – 7.39 (m, 2H), 7.34 – 7.28 (m, 3H), 6.20 (dd, J = 8.2, 1.7 Hz, 1H), 3.72 (s, 1H), 2.11 (s, 3H), 2.08 – 2.03 (m, 2H), 1.73 – 1.62 (m, 2H), 1.39 (q, J = 7.5 Hz, 2H), 1.37 – 1.24 (m, 8H), 0.97 – 0.81 (m, 3H).

^{13}C NMR (126 MHz, CDCl$_3$) δ 193.0, 141.9, 135.8, 131.8, 128.9, 128.3, 121.9, 100.8, 88.6, 84.2, 64.4, 43.7, 31.8, 29.4, 29.3, 29.2, 24.7, 24.6, 22.7, 14.1.

IR (KBr, cm^{-1}) 3341, 3121, 2358, 1639, 1400, 1086, 991, 1084, 991, 548.

HRMS (ESI) ([M+H]$^+$) Calcd. for [$C_{23}H_{29}O_2$]$^+$: 337.2162, Found. 337.2161.

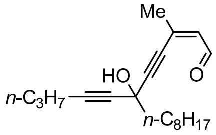

(Z)-6-hydroxy-3-methyl-6-(pent-1-yn-1-yl)tetradec-2-en-4-ynal (1-29)

Yield: 76%, yellow oil, R_f = 0.30 (petroleum ether/AcOEt = 3:1).

^{1}H NMR (500 MHz, CDCl$_3$) δ 10.00 (d, J = 8.2 Hz, 1H), 6.19 (dq, J = 8.3, 1.4 Hz, 1H), 3.09 (s, 1H), 2.22 (t, J = 7.0 Hz, 2H), 2.13 (d, J = 1.5 Hz, 3H), 1.96 – 1.90 (m, 2H), 1.57 (td, J = 14.5, 13.8, 6.5 Hz, 4H), 1.41 – 1.23 (m, 10H), 0.99 (t, J = 7.4 Hz, 3H), 0.88 (t, J = 6.8 Hz, 3H).

^{13}C NMR (126 MHz, CDCl$_3$) δ 192.9, 141.8, 135.7, 101.3, 85.2, 80.2, 79.1, 64.1, 43.8, 31.8, 29.4, 29.2, 29.2, 24.6, 24.6, 22.6, 21.8, 20.6, 14.1, 13.4.

IR (KBr, cm^{-1}) 3081, 2360, 1637, 1400, 1291, 1087, 990, 536.

HRMS (ESI) ([M+H]$^+$) Calcd. for [$C_{20}H_{31}O_2$]$^+$: 303.2319, Found. 303.2313.

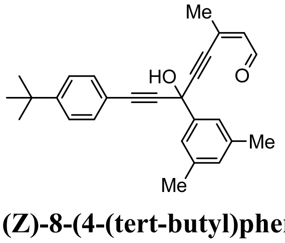

(Z)-8-(4-(tert-butyl)phenyl)-6-(3,5-dimethylphenyl)-6-hydroxy-3-methylocta-2-en-4,7-diynal (1-30)

Yield: 57%, yellow oil, R_f = 0.30 (petroleum ether/AcOEt = 3:1).

^{1}H NMR (400 MHz, CDCl$_3$) δ 10.06 (d, *J* = 8.2 Hz, 1H), 7.52 – 7.44 (m, 4H), 7.42 – 7.36 (m, 2H), 7.05 (s, 1H), 6.24 (dq, *J* = 8.2, 1.5 Hz, 1H), 3.58 (s, 1H), 2.40 (s, 6H), 2.17 (d, *J* = 1.5 Hz, 3H), 1.35 (s, 9H).
^{13}C NMR (101 MHz, CDCl$_3$) δ 192.9, 152.5, 141.5, 141.2, 138.4, 136.2, 131.7, 130.7, 125.4, 123.5, 118.7, 100.4, 87.7, 86.0, 81.0, 65.9, 34.9, 31.2, 24.5, 21.5.
IR (KBr, cm^{-1}) 3202, 2359, 1647, 1400, 1084, 991, 841, 535.
HRMS (ESI) ([M+H]$^+$) Calcd. for [$C_{27}H_{29}O_2$]$^+$: 385.2162, Found. 385.2158.

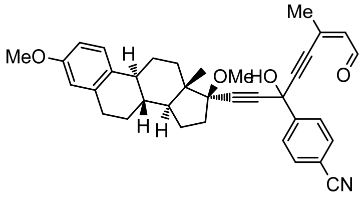

4-((Z)-1-((8R,9S,13S,14S,17S)-3,17-dimethoxy-13-methyl-7,8,9,11,12,13,14,15,16,17-decahydro-6H-cyclopenta[a]phenanthren-17-yl)-3-hydroxy-6-methyl-8-oxoocta-6-en-1,4-diyn-3-yl)benzonitrile (1-31)
Yield: 72%, yellow oil, R_f = 0.34 (petroleum ether/AcOEt = 3:1).
^{1}H NMR (500 MHz, CDCl$_3$) δ 9.97 (dd, *J* = 8.1, 1.3 Hz, 1H), 7.95 – 7.81 (m, 2H), 7.72 (dd, *J* = 8.3, 1.6 Hz, 2H), 7.18 (dd, *J* = 8.5, 2.2 Hz, 1H), 6.71 (dd, *J* = 8.6, 2.8 Hz, 1H), 6.62 (d, *J* = 2.8 Hz, 1H), 6.22 (ddd, *J* = 7.9, 4.2, 1.5 Hz, 1H), 3.77 (s, 3H), 3.39 (d, *J* = 8.0 Hz, 3H), 2.91 – 2.72 (m, 2H), 2.36 – 2.19 (m, 2H), 2.12 (dd, *J* = 3.6, 1.5 Hz, 3H), 2.04 (ddd, *J* = 13.8, 11.8, 3.7 Hz, 1H), 1.91 – 1.75 (m, 4H), 1.68 – 1.58 (m, 2H), 1.54 – 1.39 (m, 3H), 1.36 – 1.21 (m, 1H), 0.89 (s, 3H).
^{13}C NMR (126 MHz, CDCl$_3$) δ 191.9, 191.8, 157.5, 146.0, 140.2, 137.8, 136.7, 136.7, 132.6, 132.1, 132.1, 126.5, 126.5, 126.4, 118.3, 113.9, 112.9, 111.6, 98.7, 88.9, 88.8, 86.3, 85.9, 85.8, 81.8, 64.9, 55.2, 53.6, 50.2, 50.1, 48.2, 48.2, 43.8, 39.1, 36.3, 36.3, 34.4, 29.8, 27.4, 26.5, 24.4, 22.8, 12.8.
IR (KBr, cm^{-1}) 3401, 3081, 1692, 1654, 1598, 1578, 1497, 1448, 1350, 1289, 1238, 1215, 1158, 1091, 1023, 990, 939, 871, 833.
HRMS (ESI) ([M+H]$^+$) Calcd. for [$C_{36}H_{38}NO_4$]$^+$: 548.2795, Found. 548.2799.

Preparation of the symmetrical substrates.[2]

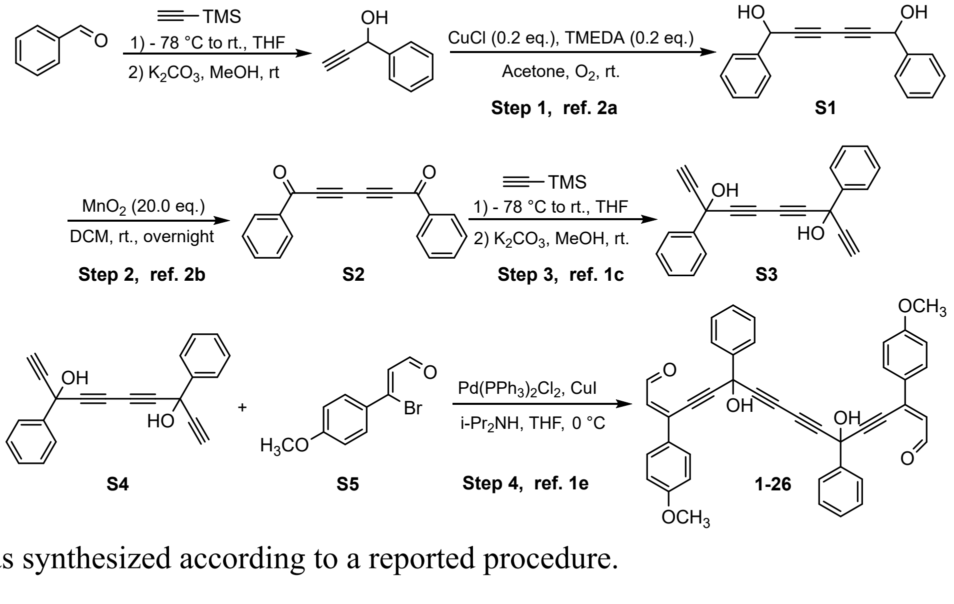

S1 was synthesized according to a reported procedure.

General procedure 1 for preparation of (S2).

The 1,6-diphenylhexa-2,4-diyne-1,6-diol **S1** (0.5 mmol) was dissolved in dichloromethane and treated with MnO_2 (870 mg, 20 eq.). After stirring at room temperature over 12 h, the reaction was complete as determined by TLC. Excess MnO_2 was removed by filtration of the reaction mixture through a pad of celite. The filtrate was washed sequentially with brine and dried over $MgSO_4$. The solvent was removed under reduced pressure, and the residue was purified by column chromatography on silica gel and eluted with petroleum ether/ethyl acetate (10/1) to afford the desired product 1,6-diphenylhexa-2,4-diyne-1,6-dione **S2** (90 mg, 70% yield).

General procedure 2 for preparation of propargylic alcohol (S3).[1c]

Trimethylsilylacetylene (25 mmol) was dissolved into THF (0.5 M), and the solution was cooled to -78 ℃. To this solution, n-Butyllithium (11 mmol, 2.5 M in hexane) was added. After being stirred for 20 minutes at -78℃, **S2** (10 mmol, 1.0 eq.) was added. The resulting mixture was allowed to warmed up to room temperature for 8 h, and then the reaction was quenched by saturated NH_4Cl (aq.), and extracted three times with ether. The combined organic layer was dried over $MgSO_4$, and the solvent was removed under a reduced pressure. The residue was purified by collum chromatography (petroleum ether/ethyl acetate = 5:1) to afford the desired **S3** (3.5 g, 78%) as an orange oil.

In a 25 mL one-neck round bottom flask equipped with magnetic stir bar was placed **S3** (3.5 g, 7.8 mmol) and MeOH (1.0 M). To this stirring mixture was added K_2CO_3 (1.0 g, 1.0 eq.) and the solution was stirred for 30 min, when TLC indicated the reaction was complete. Water (20 mL) was added and the solution was extracted (3 × 25 mL, CH_2Cl_2), dried ($MgSO_4$) and concentrated under vacuum. The crude product was purified by flash chromatography (petroleum ether/ethyl acetate = 5:1) to yield **S4** (7.6 mmol, 97%) as an orange oil.

General procedure for preparation of enynals (1).[1e]

The bromo aldehyde (2.2 mmol, 528 mg) and propargylic alcohol (1 mmol, 310 mg) were placed in a clean and anhydrous round-bottom flask equipped with a stir bar, then anhydrous THF (10 mL) and $^{i}Pr_2NH$ (0.14 mL, 1.0 eq.) were added under a nitrogen atmosphere, and the flask was cooled to 0 °C. Subsequently, CuI (28.3 mg, 15 mol%) and $Pd(PPh_3)_2Cl_2$ (7 mg, 1 mol%) were placed in the reaction flask; then after 30 min at 0 °C, the reaction mixture was warmed to room temperature and the stirring was continued at the same temperature for 1 h. Reaction progress was monitored by thin-layer chromatography (TLC) analysis. After complete consumption of the bromo aldehyde, the reaction was quenched with saturated NH_4Cl and extracted with ethyl acetate (EtOAc). The combined organic layer was washed with brine, dried ($MgSO_4$), and concentrated. The crude material was typically purified by flash chromatography using a petroleum ether/ethyl acetate = 2:1 mixture as eluent to yield the enynals **1-26** (283.6 mg, 45%) as an brown oil.

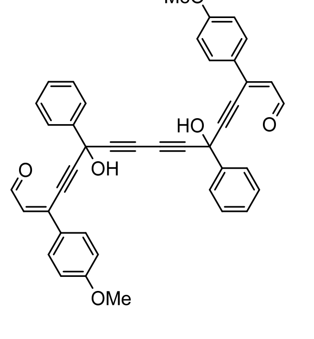

(2Z,14Z)-6,11-dihydroxy-3,14-bis(4-methoxyphenyl)-6,11-diphenylhexadeca-2,14-dien-4,7,9,12-tetraynedial (1-32)

Yield: 45%, brown oil, R_f = 0.35 (petroleum ether/AcOEt = 1:1).

^{1}H NMR (500 MHz, CDCl$_3$) δ 10.11 (d, *J* = 7.9 Hz, 2H), 7.77 (d, *J* = 7.6 Hz, 4H), 7.63 (d, *J* = 8.7 Hz, 4H), 7.43 – 7.33 (m, 8H), 6.84 (d, *J* = 8.5 Hz, 4H), 6.67 (d, *J* = 8.1 Hz, 2H), 3.77 (s, 6H).

^{13}C NMR (126 MHz, CDCl$_3$) δ 193.3, 162.3, 140.9, 140.2, 130.1, 129.3, 129.0, 128.9, 127.1, 125.7, 114.4, 100.3, 80.3, 80.2, 72.8, 69.4, 67.9, 65.7, 55.5.

IR (KBr, cm^{-1}) 3019, 2842, 2512, 1865, 1561, 1378, 1142, 1024, 949, 827, 713, 588.

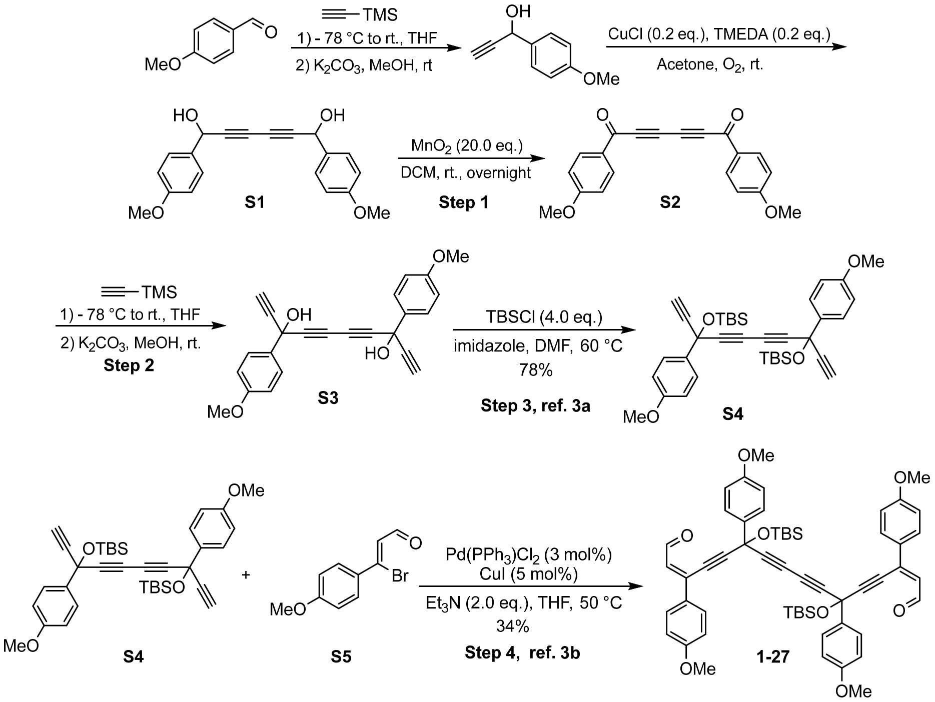

S3 was synthesized according to the above procedures.

General procedure 3 for preparation of (S4).[3a]

To a solution of propargyl alcohol **S3** (2.96 g, 8 mmol) in DMF (40 mL) was added tert-butyldimethylsilyl chloride (4.8 g, 32 mmol) and imidazole (3.28 g, 48 mmol). The solution was stirred and heated to 65 °C for 24 h. The reaction was quenched by addition of water (30 mL) and 0.5 N HCl (50 mL). The Aqueous layer was extracted with ethyl acetate (30 mL) for three times. Combined organic phases were dried with sodium sulfate and concentrated. Crude residue was purified by column chromatography to give desired silyl ether **S4** as colorless oil (3.73 g, 78%).

General procedure 4 for preparation of (1)[3b].

$PdCl_2(PPh_3)_2$ (21 mg, 3 mol%) and CuI (9.5 mg, 5 mol%) were added to a solution of (Z)-3-bromo-3-(4-methoxyphenyl) acrylaldehyde (**S5**) (527.9 mg, 2.2 mmol), 5,10-diethynyl-5,10-bis(4-methoxyphenyl)-2,2,3,3,12,12,13,13-octamethyl-4,11-dioxa-3,12-disilatetradeca-6,8-diyne (**S4**) (598.3 mg, 1.0 mmol) and NEt_3 (0.27 mL, 2 mmol) in THF (5 mL). The mixture was heated under 50 ℃ overnight under N_2. The system was filtered by short silica, then the solvent was evaporated under reduced pressure, and the residue was purified by flash chromatography with petroleum to afford the desired product **1-27** as yellow oil (312 mg, 34%).

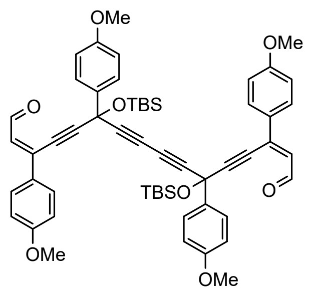

(2Z,14Z)-6,11-bis((tert-butyldimethylsilyl)oxy)-3,6,11,14-tetrakis(4-methoxyphenyl)hexadeca-2,14-dien-4,7,9,12-tetraynedial (1-33)

Yield: 34%, yellow oil, R_f = 0.35 (petroleum ether/AcOEt = 5:1).

^{1}H NMR (500 MHz, $CDCl_3$) δ 10.18 (dd, *J* = 8.1, 2.7 Hz, 2H), 7.71 (d, *J* = 2.1 Hz, 2H), 7.69 (dd, *J* = 4.4, 2.2 Hz, 4H), 7.66 (d, *J* = 2.3 Hz, 2H), 6.95 (d, *J* = 2.2 Hz, 2H), 6.94 (d, *J* = 2.3 Hz, 2H), 6.92 (s, 2H), 6.91 (s, 2H), 3.82 (s, 12H), 0.97 (s, 18H), 0.39 – -0.02 (m, 12H).

^{13}C NMR (126 MHz, $CDCl_3$) δ 192.6, 162.3, 160.0, 140.6, 134.3, 130.3, 128.9, 127.4, 126.8, 114.4, 113.98, 113.97, 101.5, 81.0, 80.04, 80.02, 69.7, 66.4, 55.5, 55.4, 25.7, 18.3, -3.26, -3.28, -3.4.

IR (KBr, cm^{-1}) 3071, 2942, 2423, 1862, 1624, 1578, 1412, 1342, 949, 827, 713, 585.

HRMS (ESI) ($[M-H]^+$) Calcd. for $[C_{56}H_{61}O_8Si_2]^-$: 917.3910, Found. 919.3903.

General procedure for preparation of siyl-enynals.[3]

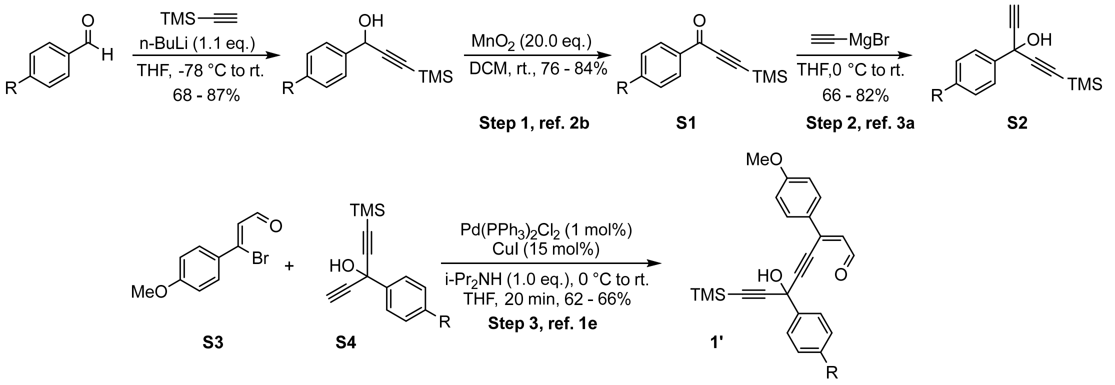

S1 was synthesized according to a reported procedure.[2b]

General procedure 2 for preparation of (S2).[3a]

To a solution of alkynone **S1** (10 mmol) in THF (10 mL) in ice bath, ethynylmagnesium bromide (0.5 M in THF, 30 mL, 15 mmol) was added slowly. Upon completion of addition, ice bath was removed and the mixture was stirred for 6 h at room temperature. The reaction was quenched with 0.5 N HCl (40 mL). The aqueous layer was extracted with ethyl acetate (45 mL) for three times. Combined organic phases were dried with $MgSO_4$ and concentrated in vacuo. The residue was purified by column chromatography on silica gel and eluted with petroleum ether/ethyl acetate (10/1) to afford the desired product 3-phenyl-1-(trimethylsilyl)penta-1,4-diyn-3-ol **S2** (66 - 82% yield).

General procedure 3 for preparation of (1').[1e]

The bromo aldehyde (1.2 eq.) and propargylic alcohol (1.0 eq.) were placed in a clean and anhydrous round-bottom flask equipped with a stir bar, then anhydrous THF and ^{i}Pr$_2$NH (1.0 eq.) were added under a nitrogen atmosphere, and the flask was cooled to 0 °C. Subsequently, CuI (15 mol%) and $Pd(PPh_3)_2Cl_2$

(1 mol%) were placed in the reaction flask; then after 20 min at 0 °C. Reaction progress was monitored by thin-layer chromatography (TLC) analysis. After complete consumption of the bromo aldehyde, the reaction was quenched with saturated NH_4Cl and extracted with ethyl acetate (EtOAc). The combined organic layer was washed with brine, dried ($MgSO_4$), and concentrated. The crude material was typically purified by flash chromatography using a petroleum ether/ethyl acetate = 5:1 mixture as eluent to yield the enynals **1'** (62 - 66%).

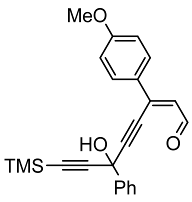

(*Z*)-6-hydroxy-3-(4-methoxyphenyl)-6-phenyl-8-(trimethylsilyl)octa-2-en-4,7-diynal (1'-32)

Yield: 62%, yellow oil, R_f = 0.35 (petroleum ether/AcOEt = 5:1).

^{1}H NMR (500 MHz, $CDCl_3$) δ 9.95 (d, *J* = 8.1 Hz, 1H), 7.69 – 7.66 (m, 2H), 7.48 (d, *J* = 9.0 Hz, 2H), 7.23 (t, *J* = 7.5 Hz, 2H), 7.17 (t, *J* = 7.3 Hz, 1H), 7.06 (s, 1H), 6.69 – 6.64 (m, 2H), 6.50 (d, *J* = 8.1 Hz, 1H), 3.58 (s, 3H), 0.06 (s, 9H).

^{13}C NMR (126 MHz, $CDCl_3$) δ 193.6, 162.5, 141.5, 141.5, 130.1, 129.3, 129.2, 128.9, 127.6, 126.1, 114.6, 104.2, 102.4, 91.4, 79.6, 65.9, 55.7, 0.0.

IR (KBr, cm^{-1}) 3742, 2960, 2868, 2212, 1728, 1658, 1379, 1194, 998, 821, 736.

HRMS (ESI) ($[M+Na]^+$) Calcd. for $[C_{24}H_{24}NaO_3Si]^+$: 411.1387, Found. 411.1379.

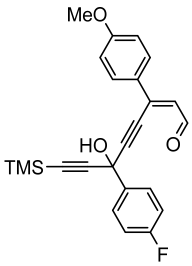

(*Z*)-6-(4-fluorophenyl)-6-hydroxy-3-(4-methoxyphenyl)-8-(trimethylsilyl)octa-2-en-4,7-diynal (1'-33)

Yield: 66%, yellow oil, R_f = 0.35 (petroleum ether/AcOEt = 5:1).

^{1}H NMR (500 MHz, $CDCl_3$) δ 9.96 (d, *J* = 8.0 Hz, 1H), 7.63 – 7.56 (m, 2H), 7.53 – 7.47 (m, 2H), 7.05 (s, 1H), 6.93 – 6.88 (m, 2H), 6.74 – 6.69 (m, 2H), 6.52 (d, *J* = 8.0 Hz, 1H), 3.64 (s, 3H), 0.04 (s, 9H).

^{13}C NMR (126 MHz, $CDCl_3$) δ 193.3, 163.37 (d, J_{C-F} = 248.4 Hz), 162.4, 141.0, 137.12 (d, J_{C-F} = 2.9 Hz), 130.6, 129.3, 128.20 (d, J_{C-F} = 8.5 Hz), 127.7, 115.93 (d, J_{C-F} = 21.9 Hz), 114.7, 103.6, 101.5, 92.2, 80.1, 65.5, 55.8, 0.0.

^{19}F NMR (471 MHz, $CDCl_3$) δ -112.71.

IR (KBr, cm^{-1}) 3127, 2962, 2869, 2218, 1720, 1582, 1346, 1131, 825, 753.

HRMS (ESI) ($[M+H]^+$) Calcd. for $[C_{24}H_{24}FO_3Si]^+$: 407.1473, Found. 407.1464.

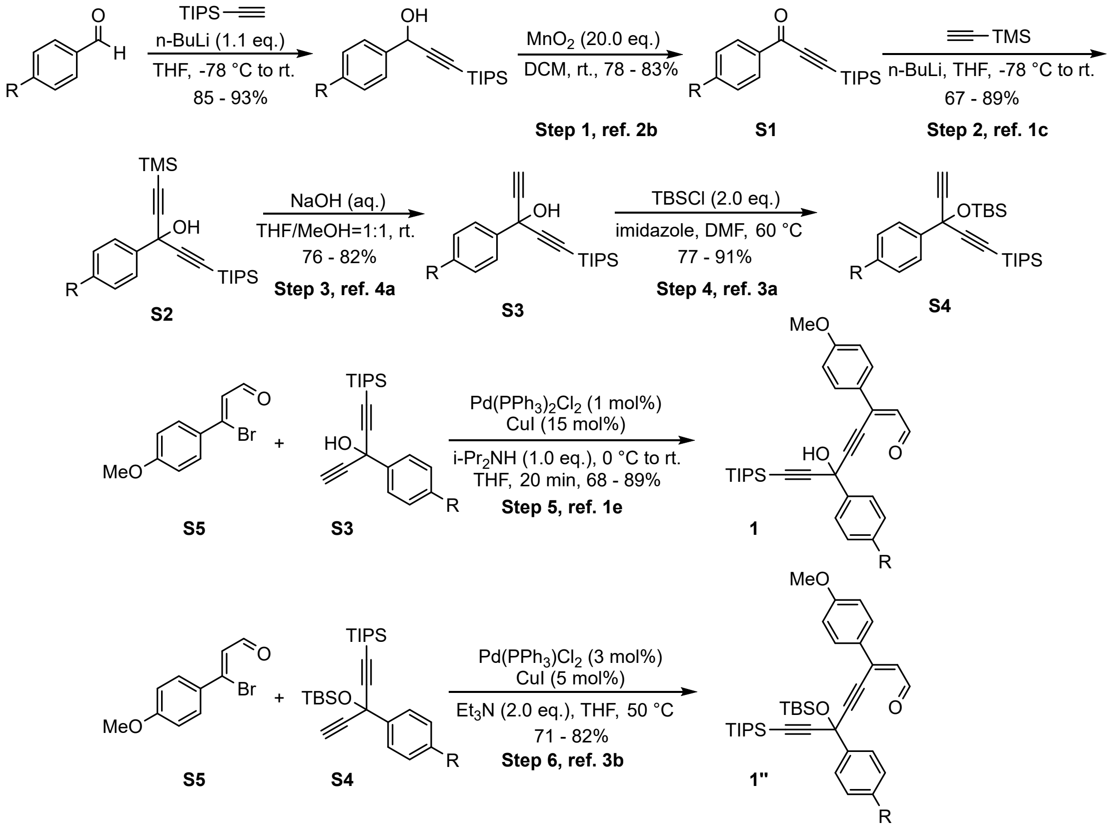

S1 was synthesized according to a reported procedure.[2b]

General procedure 2 for preparation of (S2).[1c]

Trimethylsilylacetylene (1.2 eq) was dissolved into THF (0.5 M), and the solution was cooled to -78 ℃. To this solution, n-Butyllithium (1.1 eq., 2.5 M in hexane) was added. After being stirred for 20 minutes at -78 ℃, alkynone **S1** (1.0 eq.) was added. The resulting mixture was allowed to warmed up to room temperature for 8 h, and then the reaction was quenched by saturated NH_4Cl (aq.), and extracted three times with ether. The combined organic layer was dried over $MgSO_4$, and the solvent was removed under a reduced pressure. The residue was purified by collum chromatography (petroleum ether/ethyl acetate = 10:1) to afford the desired **S2** (67 - 89%) as an orange oil.

General procedure 3 for preparation of (S3).[4a]

To a solution of **S2** (10 mmol) in THF (50 mL) and MeOH (50 mL) was added 20 drops of 1 M NaOH. The solution turned dark yellow instantly. The reaction was stirred for 1 h at rt and quenched via the addition of satd aq NH_4Cl (30 mL). Et_2O (100 mL) was added and the layers were separated. The organic phase was washed with H_2O (50 mL), satd aq NaCl (50 mL), dried ($MgSO_4$), filtered, and the solvent was removed under reduced pressure. Column chromatography (petroleum ether/ethyl acetate = 5:1) to afford **S3** (76 - 82%) as colorless oil. Note: The desilylation can be monitored by TLC. Although the R_f values are nearly identical, the $KMnO_4$ is much more pronounced for the deprotected molecule.

General procedure 4 for preparation of (S4).[3a]

To a solution of propargyl alcohol **S3** (8 mmol) in DMF (4 mL) was added tert-butyldimethylsilyl chloride (2.4 g, 16 mmol) and imidazole (1.64 g, 24 mmol). The solution was stirred and heated to 65 °C for 24 h. The reaction was quenched by addition of water (30 mL) and 0.5 N HCl (50 mL). The Aqueous

layer was extracted with ethyl acetate (30 mL) for three times. Combined organic phases were dried with sodium sulfate and concentrated. Crude residue was purified by column chromatography to give desired silyl ether **S4** as colorless oil (77 - 91%).

General procedure 5 for preparation of (1').[1e]

The bromo aldehyde (1.2 eq.) and propargylic alcohol (1.0 eq.) were placed in a clean and anhydrous round-bottom flask equipped with a stir bar, then anhydrous THF and $^{i}Pr_2NH$ (1.0 eq.)were added under a nitrogen atmosphere, and the flask was cooled to 0 °C. Subsequently, CuI (15 mol%) and $Pd(PPh_3)_2Cl_2$ (1 mol%) were placed in the reaction flask; then after 20 min at 0 °C. Reaction progress was monitored by thin-layer chromatography (TLC) analysis. After complete consumption of the bromo aldehyde, the reaction was quenched with saturated NH_4Cl and extracted with ethyl acetate (EtOAc). The combined organic layer was washed with brine, dried ($MgSO_4$), and concentrated. The crude material was typically purified by flash chromatography using a petroleum ether/ethyl acetate = 5:1 mixture as eluent to yield the enynals **1** (68 - 89%).

General procedure 6 for preparation of (1'').[3b]

$PdCl_2(PPh_3)_2$ (3 mol%) and CuI (5 mol%) were added to a solution of (Z)-3-bromo-3-(4-methoxyphenyl)acrylaldehyde (**S5**) (1.0 mmol), tert-butyldimethyl((3-phenyl-1-(triisopropylsilyl)penta-1,4-diyn-3-yl)oxy)silane (**S4**) (1.0 mmol) and NEt_3 (2 mmol) in THF (5 mL). The mixture was heated under 50 ℃ overnight under N_2. The system was filtered by short silica, then the solvent was evaporated under reduced pressure, and the residue was purified by flash chromatography with petroleum to afford the desired products **1** (71 - 82%).

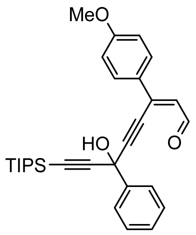

(*Z*)-6-hydroxy-3-(4-methoxyphenyl)-6-phenyl-8-(triisopropylsilyl)octa-2-en-4,7-diynal (1'-34)
Yield: 89%, yellow oil, R_f = 0.35 (petroleum ether/AcOEt = 5:1).
^{1}H NMR (500 MHz, $CDCl_3$) δ 10.17 (d, J = 8.0 Hz, 1H), 7.89 – 7.84 (m, 2H), 7.73 – 7.68 (m, 2H), 7.45 – 7.40 (m, 2H), 7.40 – 7.33 (m, 1H), 6.90 – 6.86 (m, 2H), 6.70 (d, J = 8.1 Hz, 1H), 3.82 (s, 3H), 3.75 (s, 1H), 1.12 (s, 21H).
^{13}C NMR (126 MHz, $CDCl_3$) δ 193.0, 162.2, 141.2, 140.9, 130.1, 129.0, 129.0, 128.7, 127.5, 125.9, 114.3, 105.8, 101.9, 88.3, 79.3, 65.8, 55.5, 18.6, 11.2.
IR (KBr, cm^{-1}) 2963, 2868, 2212, 1663, 1584, 1406, 1332, 1092, 949, 827, 713, 585.
HRMS (ESI) ($[M+Na]^+$) Calcd. for $[C_{30}H_{36}NaO_3Si]^+$: 495.2326, Found. 495.2329.

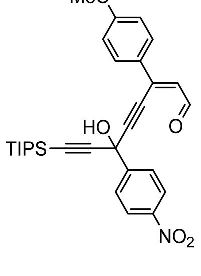

(Z)-6-hydroxy-3-(4-methoxyphenyl)-6-(4-nitrophenyl)-8-(triisopropylsilyl)octa-2-en-4,7-diynal (1'-35)

Yield: 68%, yellow oil, R_f = 0.35 (petroleum ether/AcOEt = 5:1).

^{1}H NMR (500 MHz, $CDCl_3$) δ 10.15 (d, J = 7.9 Hz, 1H), 8.32 – 8.24 (m, 2H), 8.05 – 7.99 (m, 2H), 7.70 – 7.65 (m, 2H), 6.94 – 6.88 (m, 2H), 6.73 (d, J = 8.0 Hz, 1H), 3.85 (s, 3H), 1.14 – 1.06 (m, 21H).

^{13}C NMR (126 MHz, $CDCl_3$) δ 192.5, 162.4, 148.2, 147.7, 140.3, 130.5, 128.8, 127.1, 126.9, 123.9, 114.4, 104.7, 100.3, 89.8, 80.2, 64.9, 55.5, 18.6, 11.1.

IR (KBr, cm^{-1}) 3310, 2912, 2724, 1917, 1724, 1652, 1551, 1292, 827, 523.

HRMS (ESI) ([M+Na]$^+$) Calcd. for $[C_{30}H_{35}NNaO_5Si]^+$: 540.2177, Found. 540.2170.

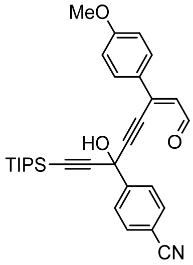

(Z)-4-(3-hydroxy-6-(4-methoxyphenyl)-8-oxo-1-(triisopropylsilyl)octa-6-en-1,4-diyn-3-yl)benzonitrile (1'-36)

Yield: 73%, yellow oil, R_f = 0.35 (petroleum ether/AcOEt = 5:1).

^{1}H NMR (500 MHz, $CDCl_3$) δ 10.14 (d, J = 8.0 Hz, 1H), 8.00 – 7.95 (m, 2H), 7.73 – 7.70 (m, 2H), 7.70 – 7.64 (m, 2H), 6.92 – 6.87 (m, 2H), 6.72 (d, J = 8.0 Hz, 1H), 3.84 (s, 3H), 1.20 – 1.02 (m, 21H).

^{13}NMR (126 MHz, $CDCl_3$) δ 192.9, 162.4, 146.3, 140.7, 132.5, 130.2, 128.9, 127.1, 126.6, 118.4, 114.4, 112.6, 104.9, 100.8, 89.3, 79.8, 65.0, 55.5, 18.6, 11.1.

IR (KBr, cm^{-1}) 3217, 3046, 2871, 2358, 1584, 1406, 1332, 1092, 949, 827, 713, 585.

HRMS (ESI) ([M+H]$^+$) Calcd. for $[C_{31}H_{36}NO_3Si]^+$: 498.2459, Found. 498.2454.

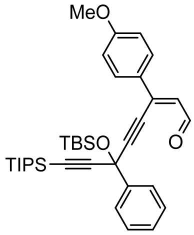

(Z)-6-((tert-butyldimethylsilyl)oxy)-3-(4-methoxyphenyl)-6-phenyl-8-(triisopropylsilyl)octa-2-en-4,7-diynal (1''-34)

Yield: 71%, yellow oil, R_f = 0.35 (petroleum ether/AcOEt =20:1).

^{1}H NMR (500 MHz, $CDCl_3$) δ 10.16 (d, J = 8.0 Hz, 1H), 7.83 – 7.78 (m, 2H), 7.74 – 7.69 (m, 2H), 7.42 – 7.36 (m, 2H), 7.35 – 7.29 (m, 1H), 6.91 – 6.86 (m, 2H), 6.70 (d, J = 8.0 Hz, 1H), 3.83 (s, 3H), 1.16 – 1.06 (m, 21H), 0.96 (s, 9H), 0.35 (s, 3H), 0.30 (s, 3H).

^{13}C NMR (126 MHz, $CDCl_3$) δ 192.8, 162.2, 143.4, 140.9, 130.0, 128.9, 128.4, 128.3, 127.6, 125.5, 114.3, 106.6, 103.4, 88.4, 79.1, 66.6, 55.4, 25.7, 18.6, 18.3, 11.3, -3.2, -3.3.

IR (KBr, cm^{-1}) 3623, 3049, 2923, 2856, 1708, 1503, 1228, 1049, 891, 744, 522.

HRMS (ESI) ([M+H]$^+$) Calcd. for $[C_{36}H_{51}O_3Si_2]^+$: 587.3371, Found. 587.3372.

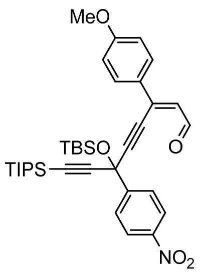

(Z)-6-((tert-butyldimethylsilyl)oxy)-3-(4-methoxyphenyl)-6-(4-nitrophenyl)-8-(triisopropylsilyl)octa-2-en-4,7-diynal (1''-35)

Yield: 82%, yellow oil, R_f = 0.35 (petroleum ether/AcOEt =20:1).

^{1}H NMR (500 MHz, CDCl$_3$) δ 10.15 (d, J = 7.9 Hz, 1H), 8.32 – 8.24 (m, 2H), 8.04 – 7.93 (m, 2H), 7.82 – 7.57 (m, 2H), 6.98 – 6.85 (m, 2H), 6.75 (d, J = 8.0 Hz, 1H), 3.86 (s, 3H), 1.16 – 1.10 (m, 21H), 0.98 (s, 9H), 0.39 (s, 3H), 0.36 (s, 3H).

^{13}C NMR (126 MHz, CDCl$_3$) δ 192.2, 162.4, 150.2, 147.8, 140.1, 130.5, 128.8, 127.2, 126.4, 123.8, 114.4, 105.5, 101.4, 90.0, 80.2, 65.9, 55.5, 25.6, 18.6, 18.3, 11.2, -3.2, -3.3.

IR (KBr, cm^{-1}) 3413, 3319, 2817, 2615, 1819, 1701, 1561, 1459, 662, 512.

HRMS (ESI) ([M+Na]$^+$) Calcd. for [$C_{36}H_{49}NNaO_5Si_2$]$^+$: 654.3041, Found. 654.3041.

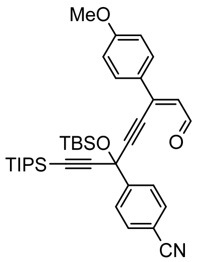

(Z)-4-(3-((tert-butyldimethylsilyl)oxy)-6-(4-methoxyphenyl)-8-oxo-1-(triisopropylsilyl)octa-6-en-1,4-diyn-3-yl)benzonitrile (1''-36)

Yield: 75%, yellow oil, R_f = 0.35 (petroleum ether/AcOEt =20:1)

^{1}H NMR (500 MHz, CDCl$_3$) δ 10.05 (d, J = 8.0 Hz, 1H), 7.90 – 7.76 (m, 2H), 7.67 – 7.51 (m, 4H), 6.87 – 6.76 (m, 2H), 6.65 (d, J = 8.0 Hz, 1H), 3.75 (s, 3H), 1.08 – 0.97 (m, 21H), 0.88 (s, 9H), 0.29 (s, 3H), 0.26 (s, 3H).

^{13}C NMR (126 MHz, CDCl$_3$) δ 192.2, 162.4, 148.4, 140.2, 132.4, 130.5, 128.8, 127.2, 126.2, 118.5, 114.4, 112.3, 105.6, 101.6, 89.7, 80.0, 66.0, 55.5, 25.6, 18.6, 18.3, 11.2, -3.2, -3.3.

IR (KBr, cm^{-1}) 2923, 2701, 2656, 2391, 1708, 1503, 1328, 1244, 970, 826, 631.

HRMS (ESI) ([M+H]$^+$) Calcd. for [$C_{37}H_{50}NO_3Si_2$]$^+$: 612.3324, Found. 612.3316.

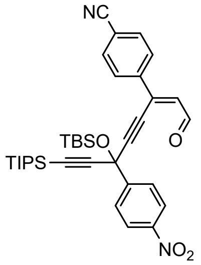

(Z)-4-(3-((tert-butyldimethylsilyl)oxy)-6-(4-methoxyphenyl)-8-oxo-1-(triisopropylsilyl)octa-6-en-1,4-diyn-3-yl)benzonitrile (1''-37)

Yield: 70%, yellow oil, R_f = 0.35 (petroleum ether/AcOEt =15:1).

^{1}H NMR (500 MHz, CDCl$_3$) δ 10.17 (d, *J* = 7.7 Hz, 1H), 8.29 – 8.26 (m, 2H), 7.96 – 7.94 (m, 2H), 7.83 – 7.78 (m, 2H), 7.74 – 7.68 (m, 2H), 6.82 (d, *J* = 7.7 Hz, 1H), 1.14 – 1.07 (m, 21H), 0.97 (s, 9H), 0.36 (s, 3H), 0.32 (s, 3H).
^{13}C NMR (126 MHz, CDCl$_3$) δ 191.5, 149.8, 148.0, 139.1, 138.3, 134.5, 132.7, 127.5, 126.4, 123.9, 117.9, 114.7, 104.9, 103.0, 90.7, 78.7, 65.9, 25.5, 18.5, 11.2, -3.2, -3.3.
IR (KBr, cm^{-1}) 3024, 2819, 2751, 1902, 1703, 1528, 1243, 991, 863, 451.
HRMS (ESI) ([M+H]$^+$) Calcd. for [$C_{36}H_{47}N_2O_4Si_2$]$^+$: 627.3069, Found. 627.3069.

4. General procedure for PtCl$_2$-catalyzed 5-exo-dig Cyclization/ 1,2-Alkynyl Migration/ 5-endo-dig Cyclization/ 1,2-H Migration or 1,2-Si Migration Cascade Reaction.

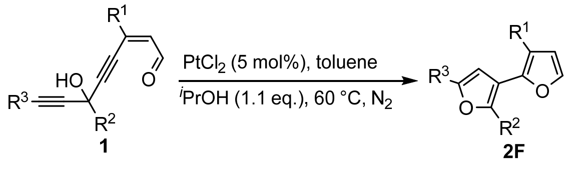

Under nitrogen atmosphere, to a solution of enynals **1** (0.2 mmol) in dry toluene (0.025 M), PtCl$_2$(0.05 eq, 2.7 mg) and ^{i}PrOH (1.1 eq, 13.2 mg) were added. The reaction mixture was then heated to a temperature of 60 °C and stirred for 6 - 48 hours. After the reaction was completed, the reaction mixture was filtered through short silica gel, and then the solvent was removed under reduced pressure. The bifuran product was purified by flash column chromatography (silica gel, petroleum ether/AcOEt = 100:1) to yield **2F1** – **2F25**.

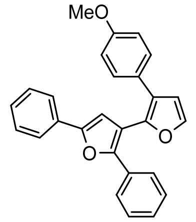

3-(4-methoxyphenyl)-2',5'-diphenyl-2,3'-bifuran (2F1)
Yield: 71 mg, 90%, 24 h, yellow oil, R$_f$ = 0.35 (petroleum ether/AcOEt = 100:1).
^{1}H NMR (400 MHz, CDCl$_3$) δ 7.78 (d, *J* = 7.6 Hz, 2H), 7.64 (d, *J* = 7.5 Hz, 2H), 7.56 (d, *J* = 1.5 Hz, 1H), 7.48 – 7.38 (m, 4H), 7.34 (m, 3H), 7.29 (d, *J* = 3.3 Hz, 1H), 6.84 (d, *J* = 8.7 Hz, 2H), 6.76 (d, *J* = 2.2 Hz, 2H), 3.80 (s, 3H).
^{13}C NMR (101 MHz, CDCl$_3$) δ 158.6, 152.7, 150.5, 142.1, 141.7, 130.5, 130.4, 128.8, 128.6, 128.4, 127.8, 127.7, 125.6, 123.9, 123.4, 114.2, 114.0, 111.9, 109.0, 55.3.
IR (KBr, cm^{-1}) 3062, 2913, 2871, 2627, 1610, 1547, 1493, 1362, 1278, 957, 783, 668.
HRMS (ESI) ([M+H]$^+$) Calcd. for [$C_{27}H_{21}O_3$]$^+$: 393.1485, Found. 393.1489.

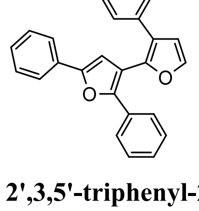

2',3,5'-triphenyl-2,3'-bifuran (2F2)
Yield: 60 mg, 83%, 6 h, yellow solid, m.p. =142-144 ℃ , R$_f$ = 0.37 (petroleum ether/AcOEt = 100:1).

^{1}H NMR (400 MHz, CDCl$_3$) δ 7.63 (d, *J* = 8.1 Hz, 2H), 7.51 – 7.47 (m, 2H), 7.45 (t, *J* = 1.5 Hz, 1H), 7.36 – 7.27 (m, 4H), 7.23 – 7.11 (m, 6H), 7.12 – 7.04 (m, 1H), 6.67 (t, *J* = 1.6 Hz, 1H), 6.62 (d, *J* = 1.2 Hz, 1H).
^{13}C NMR (101 MHz, CDCl$_3$) δ 152.8, 150.6, 142.5, 142.2, 133.0, 130.5, 130.3, 128.8, 128.5, 128.4, 127.81, 127.76, 127.4, 126.9, 125.5, 123.9, 123.7, 114.1, 111.9, 108.9.
IR (KBr, cm^{-1}) 3145, 2910, 2872, 2653, 2219, 1627, 1501, 1346, 1236, 981, 786, 691.
HRMS (ESI) ([M+H]$^+$) Calcd. for [$C_{26}H_{19}O_2$]$^+$: 363.1380, Found. 363.1371.

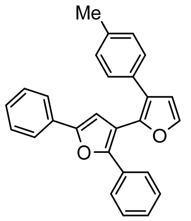

2',5'-diphenyl-3-(p-tolyl)-2,3'-bifuran (2F3)
Yield: 59 mg, 74%, 24 h, yellow oil, R_f = 0.35(petroleum ether/AcOEt = 100:1)
^{1}H NMR (400 MHz, CDCl$_3$) δ 7.76 (d, *J* = 7.7 Hz, 2H), 7.62 (d, *J* = 7.5 Hz, 2H), 7.56 (s, 1H), 7.44 (t, *J* = 7.6 Hz, 2H), 7.39 – 7.24 (m, 7H), 7.09 (d, *J* = 7.6 Hz, 2H), 6.76 (d, *J* = 14.8 Hz, 2H), 2.33 (s, 3H).
^{13}C NMR (101 MHz, CDCl$_3$) δ 152.7, 150.5, 142.1, 136.6, 130.5, 130.3, 130.0, 129.2, 128.8, 128.4, 127.8, 127.7, 127.3, 125.5, 123.9, 123.7, 114.2, 111.9, 109.0, 21.1.
IR (KBr, cm^{-1}) 3089, 2927, 2871, 2653, 2267, 1781, 1627, 1456, 972, 791, 691.
HRMS (ESI) ([M+Na]$^+$) Calcd. for [$C_{27}H_{20}NaO_2$]$^+$: 399.1356, Found. 399.1348.

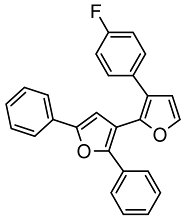

3-(4-fluorophenyl)-2',5'-diphenyl-2,3'-bifuran (2F4)
Yield: 51 mg, 67%, 16 h, yellow oil, R_f = 0.35 (petroleum ether/AcOEt = 100:1).
^{1}H NMR (400 MHz, CDCl$_3$) δ 7.69 (d, *J* = 7.6 Hz, 2H), 7.54 (d, *J* = 7.5 Hz, 2H), 7.48 (s, 1H), 7.34 (dt, *J* = 14.6, 7.1 Hz, 4H), 7.30 – 7.10 (m, 4H), 6.88 (t, *J* = 8.4 Hz, 2H), 6.66 (d, *J* = 8.4 Hz, 2H).
^{13}C NMR (101 MHz, CDCl$_3$) δ 161.93 (d, $J_{C\text{-}F}$ = 246.2 Hz), 153.0, 150.5, 142.4, 130.5, 130.3, 129.21 (d, $J_{C\text{-}F}$ = 3.2 Hz), 129.08 (d, $J_{C\text{-}F}$ = 8.0 Hz), 128.9, 128.5, 127.97 (d, $J_{C\text{-}F}$ = 6.9 Hz), 125.6, 124.0, 122.8, 115.6, 115.4, 113.9, 111.9, 108.8.
^{19}F NMR (376 MHz, CDCl$_3$) δ -115.20.
IR (KBr, cm^{-1}) 3172, 2987, 2819, 2782, 2367, 1829, 1736, 1578, 995, 691.
HRMS (ESI) ([M+H]$^+$) Calcd. for [$C_{26}H_{18}FO_2$]$^+$: 381.1285, Found. 381.1286.

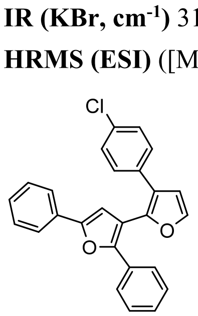

3-(4-chlorophenyl)-2',5'-diphenyl-2,3'-bifuran (2F5)

Yield: 44 mg, 55%, 48 h, yellow oil, R_f = 0.35 (petroleum ether/AcOEt = 100:1).

^{1}H NMR (400 MHz, CDCl$_3$) δ 7.76 (d, J = 7.6 Hz, 2H), 7.58 (d, J = 5.2 Hz, 3H), 7.45 (t, J = 7.5 Hz, 2H), 7.39 – 7.19 (m, 8H), 6.74 (d, J = 2.8 Hz, 2H).

^{13}C NMR (101 MHz, CDCl$_3$) δ 153.0, 150.6, 142.8, 142.4, 132.6, 131.5, 130.3, 130.2, 128.8, 128.6, 128.6, 128.4, 128.0, 127.9, 125.5, 124.0, 122.6, 113.7, 111.7, 108.7.

IR (KBr, cm^{-1}) 3124, 2839, 2726, 2314, 1928, 1720, 1602, 919, 727.

HRMS (ESI) ([M+H]$^+$) Calcd. for [$C_{26}H_{18}ClO_2$]$^+$: 397.0990, Found. 397.0978.

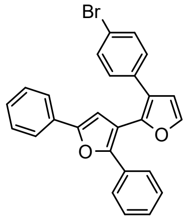

3-(4-bromophenyl)-2',5'-diphenyl-2,3'-bifuran (2F6)

Yield: 55 mg, 63%, 16 h, yellow oil, R_f = 0.35 (petroleum ether/AcOEt = 50:1).

^{1}H NMR (400 MHz, CDCl$_3$) δ 7.71 (d, J = 7.7 Hz, 2H), 7.58 – 7.49 (m, 3H), 7.39 (t, J = 7.5 Hz, 2H), 7.35 – 7.19 (m, 8H), 6.69 (d, J = 2.4 Hz, 2H).

^{13}C NMR (101 MHz, CDCl$_3$) δ 153.0, 150.6, 142.8, 142.5, 132.0, 131.6, 130.3, 130.2, 129.0, 128.8, 128.4, 128.0, 127.9, 125.5, 124.0, 122.6, 120.8, 113.7, 111.6, 108.7.

IR (KBr, cm^{-1}) 3012, 2934, 2810, 2781, 2610, 1991, 1829, 1601, 957, 671.

HRMS (ESI) ([M+Na]$^+$) Calcd. for [$C_{26}H_{17}BrNaO_2$]$^+$: 463.0304, Found. 463.0303.

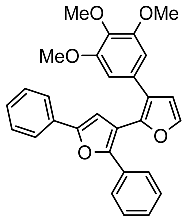

2',5'-diphenyl-3-(3,4,5-trimethoxyphenyl)-2,3'-bifuran (2F7)

Yield: 84 mg, 93%, 16 h, yellow oil, R_f = 0.35 (petroleum ether/AcOEt = 50:1).

^{1}H NMR (400 MHz, CDCl$_3$) δ 7.79 (d, J = 8.2 Hz, 2H), 7.66 – 7.59 (m, 3H), 7.46 (t, J = 7.7 Hz, 2H), 7.38 – 7.27 (m, 4H), 6.87 (s, 1H), 6.81 – 6.77 (m, 1H), 6.70 (s, 2H), 3.86 (s, 3H), 3.73 (s, 6H).

^{13}C NMR (101 MHz, CDCl$_3$) δ 153.2, 152.9, 150.6, 142.4, 142.3, 137.2, 130.5, 130.2, 128.9, 128.6, 128.4, 128.0, 127.9, 125.5, 123.9, 123.6, 114.1, 111.8, 109.0, 104.8, 60.9, 56.0.

IR (KBr, cm^{-1}) 3078, 2915, 2721, 2306, 1817, 1630, 1561, 938, 751.

HRMS (ESI) ([M+Na]$^+$) Calcd. for [$C_{29}H_{24}NaO_5$]$^+$: 475.1516, Found. 475.1521.

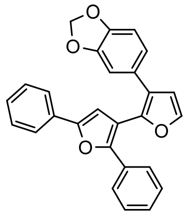

5-(2',5'-diphenyl-[2,3'-bifuran]-3-yl)benzo[d][1,3]dioxole (2F8)

Yield: 74 mg, 91%, 16 h, yellow oil, R_f = 0.33 (petroleum ether/AcOEt = 50:1).

^{1}H NMR (400 MHz, CDCl$_3$) δ 7.62 (d, *J* = 7.7 Hz, 2H), 7.45 (d, *J* = 7.7 Hz, 2H), 7.39 (s, 1H), 7.29 (t, *J* = 7.7 Hz, 2H), 7.14 (dt, *J* = 22.7, 7.0 Hz, 4H), 6.82 – 6.73 (m, 2H), 6.63 (s, 1H), 6.57 (d, *J* = 11.7 Hz, 2H), 5.74 (s, 2H).
^{13}C NMR (101 MHz, CDCl$_3$) δ 152.8, 150.4, 147.7, 146.6, 142.1, 142.0, 130.5, 130.3, 128.8, 128.4, 127.82, 127.80, 127.1, 125.6, 124.0, 123.4, 121.1, 114.0, 112.0, 108.9, 108.4, 108.0, 101.0.
IR (KBr, cm^{-1}) 2981, 1815, 1442, 1305, 1125, 978, 812, 776, 618.
HRMS (ESI) ([M+H]$^+$) Calcd. for [$C_{27}H_{19}O_4$]$^+$: 407.1278, Found. 407.1274.

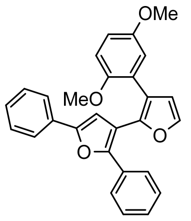

3-(2,5-dimethoxyphenyl)-2',5'-diphenyl-2,3'-bifuran (2F9)
Yield: 74 mg, 87%, 16 h, yellow oil, R_f = 0.33 (petroleum ether/AcOEt = 40:1).
^{1}H NMR (400 MHz, CDCl$_3$) δ 7.77 (d, *J* = 7.6 Hz, 2H), 7.67 (d, *J* = 7.5 Hz, 2H), 7.61 (s, 1H), 7.46 (t, *J* = 7.3 Hz, 3H), 7.40 – 7.26 (m, 4H), 6.90 (s, 1H), 6.86 (s, 1H), 6.84 – 6.74 (m, 3H), 3.73 (s, 3H), 3.66 (s, 3H).
^{13}C NMR (101 MHz, CDCl$_3$) δ 153.4, 152.5, 151.1, 150.0, 143.8, 141.4, 130.8, 130.4, 128.8, 128.3, 127.7, 125.9, 123.9, 123.1, 120.1, 116.2, 114.6, 114.0, 113.6, 112.3, 108.7, 55.9, 55.7.
IR (KBr, cm^{-1}) 3025, 2817, 2310, 1768, 1451, 1378, 1301, 1259, 1025, 813.
HRMS (ESI) ([M+H]$^+$) Calcd. for [$C_{28}H_{23}O_4$]$^+$: 423.1591, Found. 423.1594.

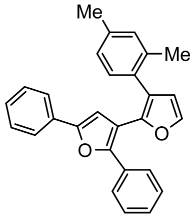

3-(2,4-dimethylphenyl)-2',5'-diphenyl-2,3'-bifuran (2F10)
Yield: 70 mg, 89%, 16 h, yellow oil, R_f = 0.33 (petroleum ether/AcOEt = 100:1).
^{1}H NMR (400 MHz, CDCl$_3$) δ 7.60 (d, *J* = 7.5 Hz, 4H), 7.46 (s, 1H), 7.32 (q, *J* = 7.1 Hz, 4H), 7.27 – 7.15 (m, 2H), 7.08 (d, *J* = 7.7 Hz, 1H), 6.96 (s, 1H), 6.89 (d, *J* = 7.8 Hz, 1H), 6.51 (s, 1H), 6.42 (s, 1H), 2.27 (s, 3H), 2.16 (s, 3H).
^{13}C NMR (101 MHz, CDCl$_3$) δ 152.6, 149.8, 143.4, 141.2, 137.2, 136.3, 131.1, 130.9, 130.4, 130.2, 130.1, 128.7, 128.3, 127.8, 127.7, 126.6, 126.3, 124.0, 123.3, 114.4, 114.2, 108.1, 21.2, 20.4.
IR (KBr, cm^{-1}) 2955, 1737, 1448, 1249, 1033, 985, 837, 755, 698.
HRMS (ESI) ([M+H]$^+$) Calcd. for [$C_{28}H_{23}O_2$]$^+$: 391.1693, Found. 391.1690.

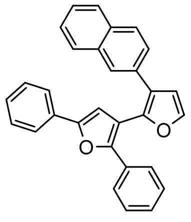

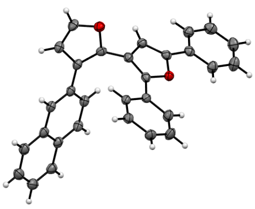

3-(naphthalen-2-yl)-2',5'-diphenyl-2,3'-bifuran (2F11)

Yield: 21 mg, 26%, 16 h, orange solid, m.p. =156-158 ℃, R_f = 0.33 (petroleum ether/AcOEt = 100:1).
^{1}H NMR (400 MHz, CDCl$_3$) δ 7.94 (s, 1H), 7.83 – 7.71 (m, 5H), 7.67 (d, J = 7.7 Hz, 2H), 7.65 – 7.57 (m, 2H), 7.50 – 7.39 (m, 4H), 7.36 – 7.26 (m, 3H), 7.27 – 7.19 (m, 1H), 6.92 (s, 1H), 6.77 (s, 1H).
^{13}C NMR (101 MHz, CDCl$_3$) δ 152.8, 150.7, 150.0, 142.8, 142.3, 133.5, 132.4, 130.6, 130.5, 130.3, 128.8, 128.4, 128.1, 128.0, 127.85, 127.77, 127.6, 126.12, 126.06, 125.85, 125.77, 125.6, 124.0, 123.7, 114.1, 112.1, 108.9.
IR (KBr, cm^{-1}) 2960, 2844, 2211, 1656, 1454, 1383, 1299, 1250, 1179, 1147, 1029, 830.
HRMS (ESI) ([M+H]$^+$) Calcd. for [$C_{30}H_{21}O_2$]$^+$: 413.1536, Found. 413.1530.

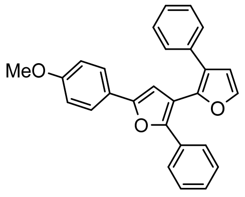

5'-(4-methoxyphenyl)-2',3-diphenyl-2,3'-bifuran (2F12)
Yield: 75 mg, 95%, 6 h, yellow oil, R_f = 0.33 (petroleum ether/AcOEt = 10:1).
^{1}H NMR (400 MHz, CDCl$_3$) δ 7.74 (d, J = 7.1 Hz, 2H), 7.69 (d, J = 6.7 Hz, 2H), 7.62 (s, 1H), 7.55 (d, J = 6.8 Hz, 2H), 7.44 – 7.22 (m, 6H), 7.02 (d, J = 7.4 Hz, 2H), 6.86 (s, 1H), 6.68 (s, 1H), 3.89 (s, 3H).
^{13}C NMR (101 MHz, CDCl$_3$) δ 159.5, 153.0, 150.0, 142.7, 142.3, 133.1, 130.7, 128.6, 128.5, 127.7, 127.5, 126.9, 125.50, 125.47, 123.7, 123.4, 114.3, 114.1, 111.9, 107.5, 55.4.
IR (KBr, cm^{-1}) 3189, 2835, 2716, 2314, 1826, 1571, 1409, 1354, 1281,970, 813.
HRMS (ESI) ([M+H]$^+$) Calcd. for [$C_{27}H_{21}O_3$]$^+$: 393.1485, Found. 393.1493.

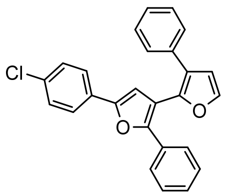

5'-(4-chlorophenyl)-2',3-diphenyl-2,3'-bifuran (2F13)
Yield: 53 mg, 67%, 24 h, yellow oil, R_f = 0.33 (petroleum ether/AcOEt = 10:1).
^{1}H NMR (400 MHz, CDCl$_3$) δ 7.66 (dd, J = 16.7, 7.6 Hz, 4H), 7.60 (s, 1H), 7.48 (d, J = 7.2 Hz, 2H), 7.42 (d, J = 7.9 Hz, 2H), 7.33 (dt, J = 15.7, 7.4 Hz, 5H), 7.28 – 7.19 (m, 1H), 6.82 (s, 1H), 6.75 (s, 1H).
^{13}C NMR (101 MHz, CDCl$_3$) δ 151.7, 150.9, 142.3, 133.4, 133.0, 130.3, 129.0, 128.8, 128.6, 128.5, 128.0, 127.5, 127.0, 125.6, 125.2, 123.9, 114.2, 112.0, 109.4.
IR (KBr, cm^{-1}) 3078, 2912, 2734, 2028, 1902, 1815, 1367, 1195, 1063, 672, 548.
HRMS (ESI) ([M+H]$^+$) Calcd. for [$C_{26}H_{18}ClO_2$]$^+$: 397.0990, Found. 397.0981.

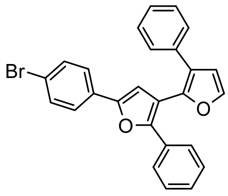

5'-(4-bromophenyl)-2',3-diphenyl-2,3'-bifuran (2F14)
Yield: 69 mg, 75%, 24 h, yellow oil, R_f = 0.33 (petroleum ether/AcOEt = 10:1).
^{1}H NMR (500 MHz, CDCl$_3$) δ 7.37 – 7.32 (m, 4H), 7.31 (d, J = 1.9 Hz, 1H), 7.32 – 7.25 (m, 1H), 7.28 (d, J = 2.0 Hz, 1H), 7.21 – 7.18 (m, 2H), 7.09 – 6.99 (m, 5H), 6.98 – 6.93 (m, 1H), 6.53 (d, J = 1.9 Hz, 1H), 6.48 (s, 1H).

^{13}C NMR (126 MHz, CDCl$_3$) δ 150.6, 149.8, 141.2, 141.1, 131.9, 130.8, 129.2, 128.1, 127.4, 127.3, 126.9, 126.3, 125.9, 124.5, 124.3, 122.8, 120.5, 113.1, 110.9, 108.4.

IR (KBr, cm^{-1}) 3114, 2928, 2860, 1710, 1506, 1296, 1042, 885, 568.

HRMS (ESI) ([M+Na]$^+$) Calcd. for [$C_{26}H_{17}BrNaO_2$]$^+$: 463.0304, Found. 463.0299.

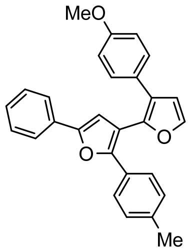

3,5'-diphenyl-2'-(p-tolyl)-2,3'-bifuran (2F15)

Yield: 66 mg, 81%, 36 h, yellow oil, R_f = 0.33 (petroleum ether/AcOEt = 10:1).

^{1}H NMR (400 MHz, CDCl$_3$) δ 7.77 – 7.73 (m, 2H), 7.54 (d, *J* = 1.9 Hz, 1H), 7.53 – 7.50 (m, 2H), 7.47 – 7.41 (m, 2H), 7.40 (d, *J* = 2.2 Hz, 1H), 7.39 – 7.37 (m, 1H), 7.34 – 7.31 (m, 1H), 7.17 – 7.12 (m, 2H), 6.85 – 6.81 (m, 2H), 6.75 (d, *J* = 1.9 Hz, 1H), 6.73 (s, 1H), 3.80 (s, 3H), 2.37 (s, 3H).

^{13}C NMR (101 MHz, CDCl$_3$) δ 158.6, 152.4, 150.8, 142.0, 141.8, 137.7, 131.9, 130.4, 129.1, 128.8, 128.6, 127.8, 127.6, 125.6, 125.5, 123.9, 123.2, 114.0, 111.8, 108.9, 55.2, 21.3.

IR (KBr, cm^{-1}) 3017, 2925, 2734, 2253, 2081, 1773, 1524, 1342, 774, 556.

HRMS (ESI) ([M+H]$^+$) Calcd. for [$C_{28}H_{23}O_3$]$^+$: 407.1642, Found. 407.1640.

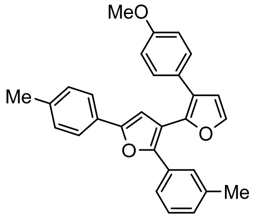

3-phenyl-2'-(m-tolyl)-5'-(p-tolyl)-2,3'-bifuran (2F16)

Yield: 77 mg, 91%, 18 h, yellow oil, R_f = 0.33 (petroleum ether/AcOEt = 20:1).

^{1}H NMR (500 MHz, CDCl$_3$) δ 7.61 (dt, *J* = 8.2, 1.6 Hz, 2H), 7.50 – 7.48 (m, 1H), 7.37 – 7.31 (m, 4H), 7.19 (d, *J* = 7.8 Hz, 2H), 7.15 (t, *J* = 7.6 Hz, 1H), 7.01 (d, *J* = 7.5 Hz, 1H), 6.79 – 6.74 (m, 2H), 6.69 (q, *J* = 1.5 Hz, 1H), 6.66 – 6.62 (m, 1H), 3.73 (s, 3H), 2.36 (s, 3H), 2.28 (s, 3H).

^{13}C NMR (126 MHz, CDCl$_3$) δ 158.6, 152.9, 150.2, 142.0, 137.8, 137.6, 130.5, 129.4, 128.5, 128.3, 127.7, 126.2, 125.7, 123.9, 123.2, 122.7, 113.9, 111.8, 108.2, 55.2, 21.6, 21.4.

IR (KBr, cm^{-1}) 3031, 2959, 2869, 2218, 1658, 1612, 1181, 909, 698, 538.

HRMS (ESI) ([M+H]$^+$) Calcd. for [$C_{29}H_{25}O_3$]$^+$: 421.1798, Found. 421.1800.

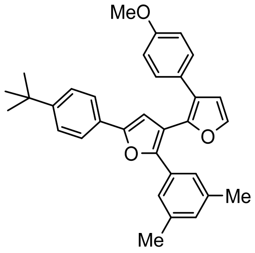

5'-(4-(tert-butyl)phenyl)-2'-(3,5-dimethylphenyl)-3-phenyl-2,3'-bifuran (2F17)

Yield: 81 mg, 85%, 12 h, yellow oil, R_f = 0.33 (petroleum ether/AcOEt = 20:1).

^{1}H NMR (500 MHz, CDCl$_3$) δ 7.59 – 7.52 (m, 2H), 7.40 – 7.35 (m, 1H), 7.34 – 7.28 (m, 2H), 7.26 – 7.19 (m, 2H), 7.07 (d, *J* = 5.1 Hz, 2H), 6.74 (s, 1H), 6.68 – 6.62 (m, 2H), 6.61 – 6.53 (m, 2H), 3.61 (s, 3H), 2.13 (s, 6H), 1.22 (s, 9H).

^{13}C NMR (126 MHz, CDCl$_3$) δ 158.6, 152.8, 150.9, 150.5, 142.1, 141.9, 137.7, 130.5, 129.5, 128.5, 127.8, 125.8, 125.7, 123.8, 123.5, 123.2, 114.0, 111.8, 108.4, 55.3, 34.7, 31.4, 21.5.

IR (KBr, cm^{-1}) 3073, 2923, 2855, 2211, 1706, 1665, 1598, 1454, 1139, 850.

HRMS (ESI) ([M+H]$^+$) Calcd. for [$C_{33}H_{33}O_3$]$^+$: 477.2424, Found. 477.2431.

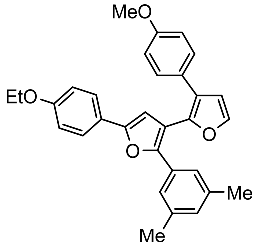

2'-(3,5-dimethylphenyl)-5'-(4-ethoxyphenyl)-3-phenyl-2,3'-bifuran (2F18)

Yield: 87 mg, 93%, 12 h, yellow oil, R_f = 0.33 (petroleum ether/AcOEt = 20:1)

^{1}H NMR (500 MHz, CDCl$_3$) δ 7.64 (d, *J* = 7.9 Hz, 2H), 7.49 (s, 1H), 7.33 (d, *J* = 7.7 Hz, 2H), 7.14 (s, 2H), 6.92 (d, *J* = 8.2 Hz, 2H), 6.83 (s, 1H), 6.77 (d, *J* = 7.9 Hz, 2H), 6.69 (s, 1H), 6.56 (s, 1H), 4.05 (q, *J* = 7.0 Hz, 2H), 3.74 (s, 3H), 2.24 (s, 6H), 1.42 (t, *J* = 7.0 Hz, 3H).

^{13}C NMR (126 MHz, CDCl$_3$) δ 158.7, 158.5, 152.7, 150.0, 142.1, 141.8, 137.6, 130.5, 129.4, 128.5, 125.8, 125.4, 123.4, 123.3, 123.1, 114.8, 113.91, 113.88, 111.8, 107.2, 63.6, 55.2, 21.4, 14.8.

IR (KBr, cm^{-1}) 2921, 1665, 1439, 1324, 1008, 942, 737, 689.

HRMS (ESI) ([M+H]$^+$) Calcd. for [$C_{31}H_{29}O_4$]$^+$: 465.2060, Found. 465.2052.

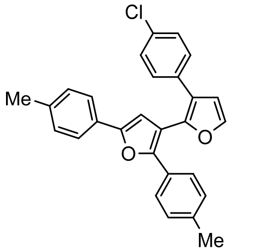

3-(4-chlorophenyl)-2',5'-di-p-tolyl-2,3'-bifuran (2F19)

Yield: 62 mg, 73%, 24 h, yellow oil, R_f = 0.33 (petroleum ether/AcOEt = 20:1).

^{1}H NMR (500 MHz, CDCl$_3$) δ 7.76 – 7.70 (m, 2H), 7.61 (s, 1H), 7.48 – 7.37 (m, 4H), 7.34 – 7.23 (m, 5H), 7.13 (s, 1H), 6.77 (d, *J* = 14.2 Hz, 2H), 2.46 (s, 3H), 2.39 (s, 3H).

^{13}C NMR (126 MHz, CDCl$_3$) δ 153.2, 150.4, 143.1, 142.4, 137.9, 137.8, 132.6, 131.7, 130.4, 129.6, 128.8, 128.7, 128.4, 127.6, 126.2, 124.0, 122.8, 122.5, 113.6, 111.7, 108.0, 21.6, 21.4.

IR (KBr, cm^{-1}) 3198, 2862, 2605, 2157, 1792, 1654, 1283, 901, 743, 584.

HRMS (ESI) ([M+Na]$^+$) Calcd. for [$C_{28}H_{21}ClNaO_2$]$^+$: 447.1122, Found. 447.1117.

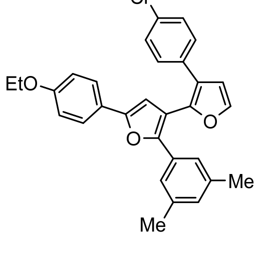

3-(4-chlorophenyl)-2'-(3,5-dimethylphenyl)-5'-(4-ethoxyphenyl)-2,3'-bifuran (2F20)

Yield: 70 mg, 75%, 24 h, yellow oil, R_f = 0.33 (petroleum ether/AcOEt = 20:1).

^{1}H NMR (500 MHz, CDCl$_3$) δ 7.66 – 7.60 (m, 2H), 7.50 (d, *J* = 1.4 Hz, 1H), 7.29 (dd, *J* = 8.3, 1.6 Hz, 2H), 7.15 (dd, *J* = 8.3, 1.4 Hz, 2H), 7.09 (s, 2H), 6.91 (d, *J* = 8.6 Hz, 2H), 6.83 (s, 1H), 6.67 (t, *J* = 1.5 Hz, 1H), 6.55 (t, *J* = 1.1 Hz, 1H), 4.03 (q, *J* = 7.0 Hz, 2H), 2.22 (s, 6H), 1.40 (t, *J* = 7.0 Hz, 3H).

^{13}C NMR (126 MHz, CDCl$_3$) δ 158.8, 153.0, 150.1, 143.2, 142.2, 137.8, 132.5, 131.8, 130.3, 129.6, 128.6, 125.4, 123.3, 123.2, 122.3, 114.8, 113.4, 111.6, 107.0, 63.6, 21.4, 14.9.

IR (KBr, cm^{-1}) 3081, 2846, 2743, 2521, 1693, 1512, 1482, 1165, 972, 594.

HRMS (ESI) ([M+H]$^+$) Calcd. for [$C_{30}H_{26}ClO_3$]$^+$: 469.1565, Found. 469.1562.

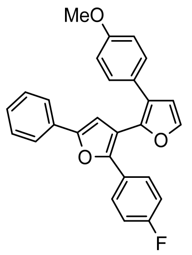

2'-(4-fluorophenyl)-3-(4-methoxyphenyl)-5'-phenyl-2,3'-bifuran (2F21)

Yield: 68 mg, 83%, 16 h, yellow oil, R_f = 0.33 (petroleum ether/AcOEt = 10:1).

^{1}H NMR (400 MHz, CDCl$_3$) δ 7.61 (d, *J* = 7.8 Hz, 2H), 7.48 – 7.39 (m, 3H), 7.30 (t, *J* = 7.6 Hz, 2H), 7.21 (t, *J* = 8.8 Hz, 3H), 6.87 (t, *J* = 8.6 Hz, 2H), 6.68 (d, *J* = 8.4 Hz, 2H), 6.63 (s, 1H), 6.60 (s, 1H), 3.66 (s, 3H).

^{13}C NMR (101 MHz, CDCl$_3$) δ 162.35 (d, $J_{C\text{-}F}$ = 248.1 Hz), 158.7, 152.7, 149.4, 142.1, 141.6, 130.2, 128.8, 128.6, 127.8, 127.44 (d, $J_{C\text{-}F}$ = 8.1 Hz), 126.86 (d, $J_{C\text{-}F}$ = 3.2 Hz), 125.4, 123.9, 123.4, 115.5, 115.3, 113.97 (d, $J_{C\text{-}F}$ = 6.2 Hz), 111.9, 108.8, 55.3.

^{19}F NMR (376 MHz, CDCl$_3$) δ -113.32.

IR (KBr, cm^{-1}) 3172, 2965, 2701, 2355, 1805, 1781, 1472, 1201, 943, 687.

HRMS (ESI) ([M+Na]$^+$) Calcd. for [$C_{27}H_{19}FNaO_3$]$^+$: 433.1210, Found. 433.1211.

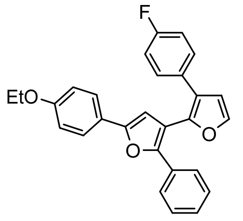

5'-(4-ethoxyphenyl)-3-(4-fluorophenyl)-2'-phenyl-2,3'-bifuran (2F22)

Yield: 74 mg, 87%, 24 h, yellow oil, R_f = 0.33 (petroleum ether/AcOEt = 20:1).

^{1}H NMR (500 MHz, CDCl$_3$) δ 7.60 (d, *J* = 8.5 Hz, 2H), 7.52 (d, *J* = 7.4 Hz, 2H), 7.47 (d, *J* = 1.8 Hz, 1H), 7.35 – 7.29 (m, 2H), 7.22 (t, *J* = 7.6 Hz, 2H), 7.18 – 7.14 (m, 1H), 6.87 (t, *J* = 9.0 Hz, 4H), 6.64 (d, *J* = 2.0 Hz, 1H), 6.52 (s, 1H), 3.96 (q, *J* = 7.0 Hz, 2H), 1.36 (t, *J* = 7.0 Hz, 3H).

^{13}C NMR (126 MHz, CDCl$_3$) δ 161.90 (d, $J_{C\text{-}F}$ = 246.2 Hz), 159.0, 153.2, 149.8, 142.7, 142.3, 130.6, 129.27 (d, $J_{C\text{-}F}$ = 3.3 Hz), 129.06 (d, $J_{C\text{-}F}$ = 8.0 Hz), 128.5, 127.8, 125.47 (d, $J_{C\text{-}F}$ = 6.4 Hz), 123.1, 122.7, 115.5, 115.4, 114.9, 113.9, 111.9, 107.2, 63.6, 14.9.

^{19}F NMR (471 MHz, CDCl$_3$) δ -115.26.

IR (KBr, cm^{-1}) 3058, 2842, 2746, 2218, 1959, 1810, 1485, 1193, 1017, 906, 691, 648.

HRMS (ESI) ([M+H]$^+$) Calcd. for [$C_{28}H_{22}FO_3$]$^+$: 425.1547, Found. 425.1542.

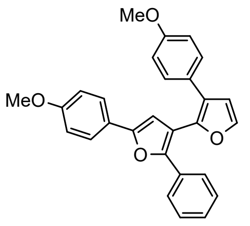

3,5'-bis(4-methoxyphenyl)-2'-phenyl-2,3'-bifuran (2F23)

Yield: 78 mg, 88%, 16 h, yellow oil, R_f = 0.33 (petroleum ether/AcOEt = 30:1).

^{1}H NMR (500 MHz, $CDCl_3$) δ 7.55 – 7.51 (m, 2H), 7.49 – 7.42 (m, 2H), 7.38 (t, *J* = 1.9 Hz, 1H), 7.26 – 7.20 (m, 2H), 7.19 – 7.12 (m, 2H), 7.12 – 7.05 (m, 1H), 6.84 – 6.78 (m, 2H), 6.69 – 6.63 (m, 2H), 6.61 – 6.55 (m, 1H), 6.46 (d, *J* = 2.5 Hz, 1H), 3.69 (s, 3H), 3.61 (s, 3H).

^{13}C NMR (126 MHz, $CDCl_3$) δ 159.4, 158.6, 152.9, 149.8, 142.0, 141.9, 130.7, 128.6, 128.4, 127.6, 125.6, 125.5, 125.4, 123.4, 123.3, 114.3, 114.0, 111.8, 107.5, 55.4, 55.2.

IR (KBr, cm^{-1}) 3052, 2918, 2854, 1712, 1509, 1258, 1063, 907, 740, 610.

HRMS (ESI) ([M+Na]$^+$) Calcd. for [$C_{28}H_{22}NaO_4$]$^+$: 445.1410, Found. 445.1402.

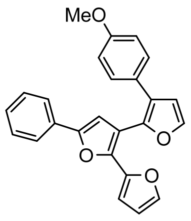

3''-(4-methoxyphenyl)-5'-phenyl-2,2':3',2''-terfuran (2F24)

Yield: 33 mg, 43%, 6 h, yellow oil, R_f = 0.33 (petroleum ether/AcOEt = 50:1), Note: This compound is a little bit unstable.

^{1}H NMR (500 MHz, $CDCl_3$) δ 7.68 (d, *J* = 7.7 Hz, 2H), 7.53 (s, 1H), 7.38 (t, *J* = 7.6 Hz, 3H), 7.35 (s, 1H), 7.33 (s, 1H), 7.28 (d, *J* = 7.3 Hz, 1H), 6.82 (d, *J* = 8.5 Hz, 2H), 6.67 (s, 2H), 6.56 (d, *J* = 2.9 Hz, 1H), 6.38 (s, 1H), 3.78 (s, 3H).

^{13}C NMR (126 MHz, $CDCl_3$) δ 158.7, 152.7, 145.4, 142.5, 142.4, 141.9, 141.2, 130.1, 128.8, 128.7, 127.8, 125.8, 124.5, 124.0, 123.5, 113.9, 113.8, 112.2, 111.4, 108.2, 107.7, 55.3.

IR (KBr, cm^{-1}) 3063, 2909, 2852, 2663, 1892, 1781, 1670, 1564, 1393, 1161, 966, 795, 692.

HRMS (ESI) ([M+H]$^+$) Calcd. for [$C_{25}H_{19}O_4$]$^+$: 383.1278, Found. 383.1280.

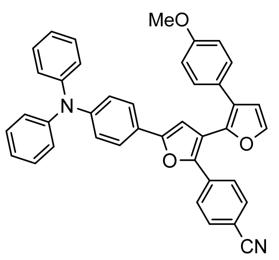

4-(5'-(4-(diphenylamino)phenyl)-3-(4-methoxyphenyl)-[2,3'-bifuran]-2'-yl)benzonitrile (2F25)

Yield: 83 mg, 71%, 12 h, yellow solid, m.p. = 184 - 187 ℃, R_f = 0.33 (petroleum ether/AcOEt = 20:1)

^{1}H NMR (500 MHz, $CDCl_3$) δ 7.58 (t, *J* = 8.0 Hz, 4H), 7.54 (d, *J* = 1.9 Hz, 1H), 7.50 (s, 1H), 7.49 (s, 1H), 7.31 – 7.26 (m, 6H), 7.13 (d, *J* = 8.0 Hz, 4H), 7.11 (s, 1H), 7.09 – 7.05 (m, 3H), 6.77 (d, *J* = 8.4 Hz, 2H), 6.72 (d, *J* = 1.9 Hz, 1H), 6.66 (s, 1H), 3.77 (s, 3H).

^{13}C NMR (126 MHz, CDCl$_3$) δ 158.8, 154.4, 148.1, 147.3, 147.1, 142.5, 140.8, 134.4, 132.0, 129.4, 128.5, 125.3, 125.2, 125.1, 124.8, 124.1, 123.5, 123.4, 123.0, 119.1, 117.3, 114.0, 112.2, 110.0, 108.1, 55.3.

IR (KBr, cm^{-1}) 3426, 3275, 3169, 3083, 2360, 1637, 1400, 1087, 989.

HRMS (ESI) ([M+Na]$^+$) Calcd. for [$C_{40}H_{28}N_2NaO_3$]$^+$: 607.1992, Found. 607.1986.

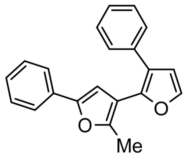

2'-methyl-3,5'-diphenyl-2,3'-bifuran (2F26)

Yield: 52 mg, 87%, 6 h, yellow oil, R_f = 0.33(petroleum ether/AcOEt = 100:1)

^{1}H NMR (400 MHz, CDCl$_3$) δ 7.64 (d, J = 7.7 Hz, 2H), 7.54 (s, 1H), 7.49 (d, J = 7.6 Hz, 2H), 7.44 – 7.38 (m, 4H), 7.36 – 7.24 (m, 2H), 6.70 – 6.63 (m, 2H), 2.31 (s, 3H).

^{13}C NMR (101 MHz, CDCl$_3$) δ 151.9, 149.8, 143.5, 141.5, 133.9, 130.7, 128.7, 128.6, 128.1, 127.2, 126.9, 123.5, 122.2, 113.9, 112.5, 105.7, 13.4.

IR (KBr, cm^{-1}) 3173, 2935, 2781, 2699, 1672, 1561, 987, 834, 725.

HRMS (ESI) ([M+H]$^+$) Calcd. for [$C_{21}H_{17}O_2$]$^+$: 301.1223, Found. 301.1225.

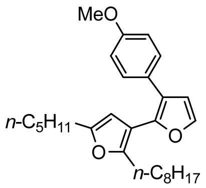

3-(4-methoxyphenyl)-2'-octyl-5'-pentyl-2,3'-bifuran (2F27)

Yield: 48 mg, 57%, 72 h, yellow oil, R_f = 0.30 (petroleum ether/AcOEt = 100:1)

^{1}H NMR (500 MHz, CDCl$_3$) δ 7.32 (d, J = 1.9 Hz, 1H), 7.23 (dt, J = 8.3, 1.8 Hz, 2H), 6.81 – 6.75 (m, 2H), 6.44 (d, J = 1.9 Hz, 1H), 5.78 (s, 1H), 3.72 (s, 3H), 2.46 (dt, J = 11.0, 7.6 Hz, 4H), 1.54 – 1.47 (m, 2H), 1.46 – 1.40 (m, 2H), 1.27 – 1.20 (m, 5H), 1.20 – 1.16 (m, 2H), 1.16 – 1.09 (m, 8H), 0.81 (d, J = 6.6 Hz, 2H), 0.79 (d, J = 1.9 Hz, 1H), 0.77 (d, J = 7.2 Hz, 2H).

^{13}C NMR (126 MHz, CDCl$_3$) δ 157.4, 153.3, 151.3, 142.7, 139.9, 128.1, 125.5, 120.1, 112.8, 111.2, 110.6, 104.3, 54.2, 30.8, 30.3, 28.2, 28.2, 28.2, 27.1, 26.8, 26.6, 26.4, 21.6, 21.4, 13.1, 13.0.

IR (KBr, cm^{-1}) 2361, 1637, 1527, 1400, 1298, 991, 516.

HRMS (ESI) ([M+H]$^+$) Calcd. for [$C_{28}H_{39}O_3$]$^+$: 423.2894, Found. 423.2895.

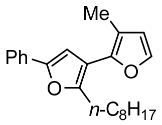

3-methyl-2'-octyl-5'-phenyl-2,3'-bifuran (2F28)

Yield: 45 mg, 67%, 24 h, yellow oil, R_f = 0.30 (petroleum ether/AcOEt = 100:1)

^{1}H NMR (500 MHz, CDCl$_3$) δ 7.71 – 7.58 (m, 2H), 7.42 – 7.31 (m, 3H), 7.22 (t, J = 7.4 Hz, 1H), 6.73 (s, 1H), 6.28 (d, J = 1.8 Hz, 1H), 2.85 (t, J = 7.6 Hz, 2H), 2.14 (s, 3H), 1.73 (p, J = 7.5 Hz, 2H), 1.41 – 1.33 (m, 2H), 1.33 – 1.19 (m, 8H), 0.87 (t, J = 6.8 Hz, 3H).

^{13}C NMR (126 MHz, CDCl$_3$) δ 153.3, 151.8, 144.1, 140.5, 130.9, 128.7, 127.1, 123.5, 115.8, 114.0, 113.6, 105.2, 32.0, 29.4, 29.3, 29.3, 28.6, 27.7, 22.7, 14.2, 11.0.

IR (KBr, cm^{-1}) 2358, 1636, 1400, 1087, 989, 529.

HRMS (ESI) ([M+H]$^+$) Calcd. for [$C_{23}H_{29}O_2$]$^+$: 337.2162, Found. 337.2160.

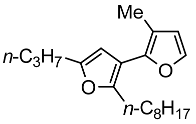

3-methyl-2'-octyl-5'-propyl-2,3'-bifuran (2F29)

Yield: 39 mg, 65%, 24 h, yellow oil, R_f = 0.30 (petroleum ether/AcOEt = 100:1)

^{1}H NMR (500 MHz, CDCl$_3$) δ 7.29 (s, 1H), 6.25 (d, *J* = 1.8 Hz, 1H), 6.08 (s, 1H), 2.74 (t, *J* = 7.6 Hz, 2H), 2.56 (t, *J* = 7.5 Hz, 2H), 2.09 (s, 3H), 1.77 – 1.57 (m, 4H), 1.38 – 1.16 (m, 10H), 0.97 (t, *J* = 7.3 Hz, 3H), 0.87 (t, *J* = 6.6 Hz, 3H).

^{13}C NMR (126 MHz, CDCl$_3$) δ 154.2, 151.5, 144.7, 140.1, 115.0, 113.8, 111.7, 105.0, 31.9, 30.0, 29.3, 29.3, 29.3, 28.6, 27.5, 22.7, 21.4, 14.1, 13.8, 10.9.

IR (KBr, cm^{-1}) 2359, 1642, 1400, 1086, 992, 673, 561.

HRMS (ESI) ([M+H]$^+$) Calcd. for [$C_{20}H_{31}O_2$]$^+$: 303.2319, Found. 303.2315.

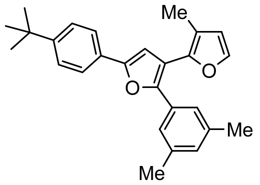

5'-(4-(tert-butyl)phenyl)-2'-(3,5-dimethylphenyl)-3-methyl-2,3'-bifuran (2F30)

Yield: 54 mg, 71%, 36 h, yellow oil, R_f = 0.35 (petroleum ether/AcOEt = 100:1)

^{1}H NMR (500 MHz, CDCl$_3$) δ 7.69 (d, *J* = 8.2 Hz, 1H), 7.49 – 7.33 (m, 2H), 7.22 (s, 1H), 6.91 (s, 0H), 6.77 (d, *J* = 1.4 Hz, 0H), 6.34 (d, *J* = 1.8 Hz, 0H), 2.30 (s, 2H), 1.91 (s, 1H), 1.35 (s, 3H).

^{13}C NMR (126 MHz, CDCl$_3$) δ 152.6, 150.7, 149.5, 143.6, 141.3, 137.8, 130.9, 129.4, 127.8, 125.7, 123.8, 123.5, 117.5, 113.8, 113.7, 108.0, 34.7, 31.3, 21.5, 10.9.

IR (KBr, cm^{-1}) 2613, 1901, 1672, 1401, 1282, 998, 721.

HRMS (ESI) ([M+H]$^+$) Calcd. for [$C_{27}H_{29}O_2$]$^+$: 385.2162, Found. 385.2154.

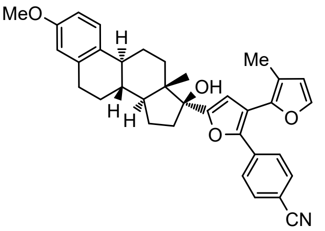

4-(5'-((8R,9S,13S,14S,17S)-17-hydroxy-3-methoxy-13-methyl-7,8,9,11,12,13,14,15,16,17-decahydro-6H-cyclopenta[a]phenanthren-17-yl)-3-methyl-[2,3'-bifuran]-2'-yl)benzonitrile (2F31)

Yield: 44 mg, 41%, 6 h, yellow oil, R_f = 0.35 (petroleum ether/AcOEt = 20:1)

^{1}H NMR (400 MHz, CDCl$_3$) δ 7.68 – 7.58 (m, 4H), 7.45 (d, *J* = 1.8 Hz, 1H), 7.22 (d, *J* = 8.6 Hz, 1H), 6.74 (dd, *J* = 8.6, 2.8 Hz, 1H), 6.67 (d, *J* = 2.8 Hz, 1H), 6.55 (s, 1H), 6.39 (d, *J* = 1.9 Hz, 1H), 6.32 (dd, *J* = 3.4, 2.0 Hz, 1H), 3.80 (s, 3H), 3.07 – 2.74 (m, 2H), 2.49 – 2.39 (m, 2H), 2.38 – 2.31 (m, 2H), 2.18 (ddd, *J* = 16.2, 11.5, 2.1 Hz, 1H), 1.99 (td, *J* = 6.3, 3.5 Hz, 1H), 1.95 (s, 3H), 1.88 – 1.76 (m, 2H), 1.75 – 1.59 (m, 3H), 1.56 – 1.34 (m, 2H), 1.06 (s, 1H).

^{13}C NMR (101 MHz, CDCl$_3$) δ 157.6, 151.8, 146.4, 143.9, 142.5, 141.9, 137.9, 134.8, 132.6, 132.2, 127.7, 126.0, 125.5, 119.1, 118.5, 116.3, 114.1, 113.9, 111.5, 110.1, 109.4, 56.2, 55.2, 46.9, 44.2, 37.2,

35.6, 31.5, 29.7, 27.8, 26.7, 16.7, 11.0.

IR (KBr, cm^{-1}) 3401, 3223, 3181, 2360, 1638, 1400, 1291, 1086, 989, 536.

HRMS (ESI) ([M+H]$^+$) Calcd. for [$C_{35}H_{36}NO_4$]$^+$: 534.2639, Found. 534.2641.

Preparation of 4F1.

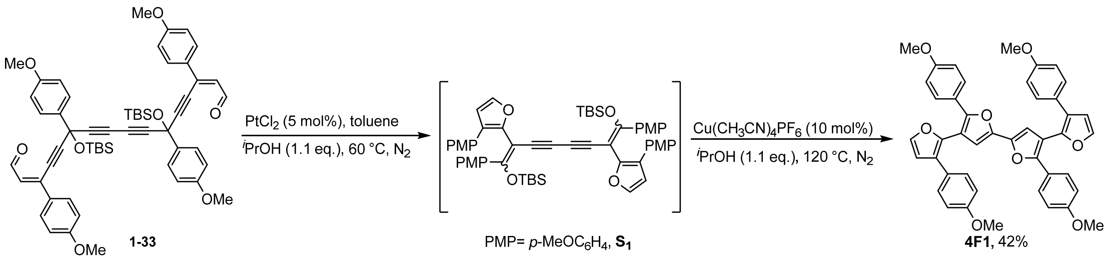

Under nitrogen atmosphere, to a solution of enynals **1-33** (183.7 mg, 0.2 mmol) in dry toluene (0.025 M), $PtCl_2$(0.05 eq, 2.7 mg) and ^{i}PrOH (1.1 eq, 13.2 mg) were added. The reaction mixture was then heated to a temperature of 60 ℃ and stirred for 48 hours. After the reaction was completed, the reaction mixture was filtered through short silica gel, and then the solvent was removed under reduced pressure. **S1** was purified by flash column chromatography (silica gel, petroleum ether/AcOEt = 20:1) .

Under nitrogen atmosphere, to a solution of **S1** (0.1 mmol) in dry toluene (0.1 M), $Cu(CH_3CN)_4PF_6$ (10 mol%, 3.7 mg) and ^{i}PrOH (1.1 eq, 6.6 mg)were added. The reaction mixture was then heated to a temperature of 120 °C and stirred for 24 h. After the reaction was completed, the reaction mixture was filtered through short silica gel, and then the solvent was removed under reduced pressure. The bifuran product was purified by flash column chromatography (silica gel, petroleum ether/AcOEt = 10:1) to yield **4F1** (58 mg, 42% for two steps).

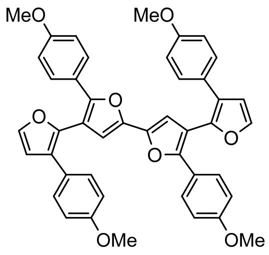

2',3,3''',5''-tetrakis(4-methoxyphenyl)-2,3':5',2'':4'',2'''-quaterfuran (4F1)

Yield: 58 mg, 42%, 24 h, yellow oil, R_f = 0.33 (petroleum ether/AcOEt =10:1).

^{1}H NMR (500 MHz, CDCl$_3$) δ 7.51 (d, J = 1.8 Hz, 2H), 7.44 (d, J = 8.7 Hz, 4H), 7.33 (d, J = 8.5 Hz, 4H), 6.80 (d, J = 3.3 Hz, 4H), 6.78 (d, J = 3.5 Hz, 4H), 6.70 (d, J = 1.8 Hz, 2H), 6.67 (s, 2H), 3.79 (s, 3H), 3.77 (s, 3H).

^{13}C NMR (126 MHz, CDCl$_3$) δ 159.4, 158.6, 150.6, 144.4, 142.1, 141.5, 128.5, 127.0, 125.5, 123.3, 123.1, 114.0, 113.9, 112.4, 111.8, 109.2, 55.3, 55.2.

IR (KBr, cm^{-1}) 3013, 2922, 2843, 2726, 2241, 1753, 1326, 1029, 922, 819, 723, 631.

HRMS (ESI) ([M+H]$^+$) Calcd. for [$C_{44}H_{35}O_8$]$^+$: 691.2326, Found. 691.2328.

5. General procedure for $PtCl_2$ select 1,2-Si migration reaction to yield β-Si-bifurans.

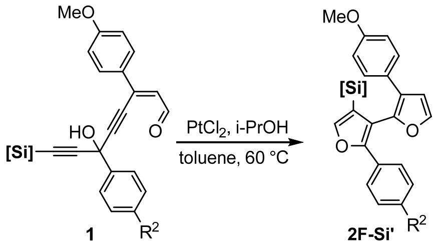

Under nitrogen atmosphere, to a solution of enynals (0.2 mmol) in dry toluene (0.025 M), $PtCl_2$(0.05 eq, 2.7 mg) and ^{i}PrOH (1.1 eq, 13.2 mg) were added. The reaction mixture was then heated to a temperature of 60 °C and stirred for 6-48 hours. After the reaction was completed, the reaction mixture was filtered through short silica gel, and then the solvent was removed under reduced pressure. The bifuran product was purified by flash column chromatography (silica gel, petroleum ether/AcOEt = 100:1) to yield **2F32-Si'** – **2F36-Si'**.

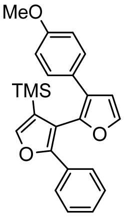

(3-(4-methoxyphenyl)-2'-phenyl-[2,3'-bifuran]-4'-yl)trimethylsilane (2F32-Si')

Yield: 49 mg, 60%, 48 h, yellow oil, R_f = 0.35 (petroleum ether/AcOEt = 20:1).

^{1}H NMR (500 MHz, $CDCl_3$) δ 7.59 (d, J = 1.8 Hz, 1H), 7.47 – 7.42 (m, 4H), 7.26 – 7.22 (m, 5H), 7.21 – 7.16 (m, 1H), 6.80 – 6.70 (m, 3H), 3.75 (s, 3H), 0.00 (s, 6H).

^{13}C NMR (126 MHz, $CDCl_3$) δ 159.6, 154.2, 148.1, 143.6, 143.4, 131.6, 129.7, 129.1, 128.9, 126.8, 126.3, 124.8, 124.7, 116.8, 115.1, 112.3, 56.4, 0.0.

IR (KBr, cm^{-1}) 2930, 2854, 2212, 1663, 1566, 1384, 1142, 973 , 911, 763, 732, 642.

HRMS (ESI) ([M+Na]$^+$) Calcd. for $[C_{25}H_{26}NaO_2Si]^+$: 411.1387, Found. 411.1390.

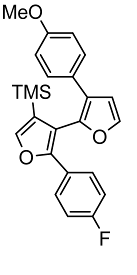

(2'-(4-fluorophenyl)-3-(4-methoxyphenyl)-[2,3'-bifuran]-4'-yl)trimethylsilane (2F33-Si')

Yield: 45 mg, 55%, 48 h, yellow oil, R_f = 0.35 (petroleum ether/AcOEt = 20:1).

^{1}H NMR (500 MHz, $CDCl_3$) δ 7.58 – 7.55 (m, 1H), 7.42 (s, 1H), 7.38 (ddt, J = 9.1, 5.3, 1.2 Hz, 2H), 7.21 – 7.16 (m, 2H), 6.90 (t, J = 8.7 Hz, 2H), 6.81 – 6.65 (m, 3H), 3.73 (s, 3H), -0.00 (s, 9H).

^{13}C NMR (126 MHz, $CDCl_3$) δ 163.44 (d, J_{C-F} = 247.8 Hz), 159.6, 153.3, 148.0, 143.5, 129.0, 128.19 (d, J_{C-F} = 8.2 Hz), 127.89 (d, J_{C-F} = 3.2 Hz), 126.7, 124.78 (d, J_{C-F} = 10.7 Hz), 116.8, 116.6, 115.1, 112.4, 56.4, 0.0.

^{19}F NMR (471 MHz, $CDCl_3$) δ -113.6.

IR (KBr, cm^{-1}) 3019, 2910, 2254, 1701, 1622, 1573, 1402, 1339, 982, 829, 634.

HRMS (ESI) ([M+Na]$^+$) Calcd. for $[C_{24}H_{23}FNaO_3Si]^+$: 429.1293, Found. 429.1292.

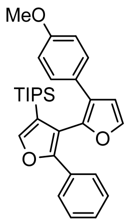

triisopropyl(3-(4-methoxyphenyl)-2'-phenyl-[2,3'-bifuran]-4'-yl)silane (2F34-Si')

Yield: 64 mg, 65%, 48 h, yellow oil, R_f = 0.35 (petroleum ether/AcOEt = 20:1).

^{1}H NMR (500 MHz, CDCl$_3$) δ 7.53 (d, *J* = 2.0 Hz, 1H), 7.46 (s, 1H), 7.40 – 7.32 (m, 2H), 7.22 – 7.17 (m, 2H), 7.17 – 7.11 (m, 3H), 6.69 – 6.67 (m, 2H), 6.67 – 6.65 (m, 1H), 3.70 (s, 3H), 0.97 (t, *J* = 2.7 Hz, 3H), 0.93 (d, *J* = 6.1 Hz, 9H).

^{13}C NMR (126 MHz, CDCl$_3$) δ 158.3, 153.5, 148.4, 142.7, 141.8, 130.4, 128.5, 128.0, 127.6, 125.8, 125.1, 123.7, 118.2, 115.7, 113.8, 111.5, 55.2, 18.7, 11.6.

IR (KBr, cm^{-1}) 2924, 2855, 2212, 1662, 1558, 1449, 1390, 1230, 1034, 916, 852, 690.

HRMS (ESI) ([M+Na]$^+$) Calcd. for [$C_{30}H_{36}NaO_3Si$]$^+$: 495.2326, Found. 495.2316.

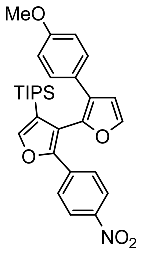

triisopropyl(3-(4-methoxyphenyl)-2'-(4-nitrophenyl)-[2,3'-bifuran]-4'-yl)silane (2F35-Si')

Yield: 66 mg, 64%, 48 h, yellow oil, R_f = 0.35 (petroleum ether/AcOEt = 20:1). (Note: β/α > 7:1)

^{1}H N)MR (500 MHz, CDCl$_3$) δ 8.02 (d, *J* = 8.9 Hz, 2H), 7.58 – 7.55 (m, 2H), 7.49 – 7.43 (m, 2H), 7.13 – 7.03 (m, 2H), 6.72 (d, *J* = 1.9 Hz, 1H), 6.68 – 6.64 (m, 2H), 3.69 (s, 3H), 1.02 – 0.92 (m, 21H).

^{13}C NMR (126 MHz, CDCl$_3$) δ 158.6, 150.7, 150.2, 146.4, 142.4, 141.5, 136.1, 127.8, 125.4, 123.8, 119.7, 119.3, 114.0, 111.7, 55.2, 18.7, 11.7.

IR (KBr, cm^{-1}) 2924, 2855, 2212, 1662, 1558, 1449, 1390, 1230, 1034, 916, 852, 690.

HRMS (ESI) ([M+Na]$^+$) Calcd. for [$C_{30}H_{35}NNaO_5Si$]$^+$: 540.2177, Found. 540.2185.

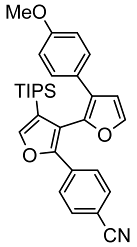

4-(3-(4-methoxyphenyl)-4'-(triisopropylsilyl)-[2,3'-bifuran]-2'-yl)benzonitrile (2F36-Si')

Yield: 73 mg, 73%, 48 h, yellow oil, R_f = 0.35 (petroleum ether/AcOEt = 20:1). (Note: β/α > 20:1)

^{1}H NMR (500 MHz, CDCl$_3$) δ 7.48 (d, *J* = 2.0 Hz, 1H), 7.46 (s, 1H), 7.35 (q, *J* = 8.4 Hz, 4H), 7.03 – 6.99 (m, 2H), 6.63 (d, *J* = 1.9 Hz, 1H), 6.62 – 6.58 (m, 2H), 3.63 (s, 3H), 0.93 – 0.83 (m, 21H).

^{13}C NMR (126 MHz, CDCl$_3$) δ 158.5, 151.0, 149.8, 142.3, 141.6, 134.3, 132.2, 127.8, 125.3, 125.2, 124.1, 119.1, 118.92, 118.87, 113.9, 111.7, 110.5, 55.2, 18.7, 18.6, 11.6.

IR (KBr, cm^{-1}) 2924, 2855, 2212, 1662, 1558, 1449, 1390, 1230, 1034, 916, 852, 690.

HRMS (ESI) ([M+H]$^+$) Calcd. for [$C_{31}H_{36}NO_3Si$]$^+$: 498.2459, Found. 498.2451.

6. General procedure for $PtCl_2$ and Au-catalyzed Select 1,2-H migration Reaction to yield α-Si-bifurans.

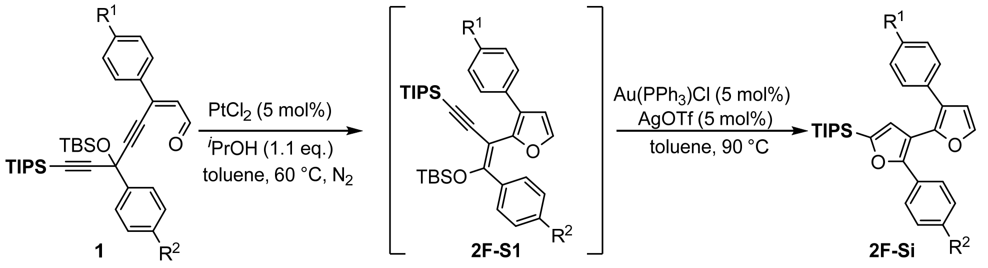

Under nitrogen atmosphere, to a solution of enynals **1** (0.2 mmol) in dry toluene (0.025 M), $PtCl_2$(0.05 eq, 2.7 mg) and ^{i}PrOH (1.1 eq, 13.2 mg) were added. The reaction mixture was then heated to a temperature of 60 °C and stirred for 6-48 hours. After the reaction was completed, the reaction mixture was filtered through short silica gel, and then the solvent was removed under reduced pressure. The bifuran product was purified by flash column chromatography (silica gel, petroleum ether/AcOEt = 100:1) to yield **2F-S1**.

Under nitrogen atmosphere, to a solution of **2F-S1** (0.1 mmol) in dry toluene (0.1 M), Au(PPh_3)Cl (5 mol%, 2.4 mg) and AgOTf (5 mol%, 1.2 mg) were added. The reaction mixture was then heated to a temperature of 90 °C and stirred for 12 - 24 h. After the reaction was completed, the reaction mixture was filtered through short silica gel, and then the solvent was removed under reduced pressure. The bifuran product was purified by flash column chromatography (silica gel, petroleum ether/AcOEt = 10:1) to yield **2F-Si**.

6.1 The gram-scale synthesis of product 2F34-Si.

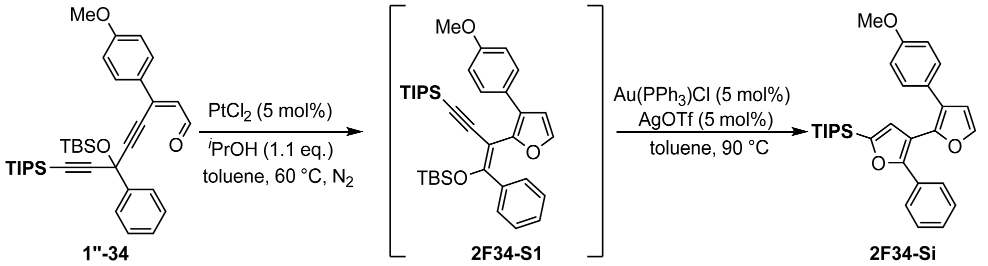

Under nitrogen atmosphere, to a solution of enynals **1''-34** (7.6 mmol, 4.5 g) in dry toluene (200 mL), $PtCl_2$(0.05 eq, 101 mg) and ^{i}PrOH (1.1 eq, 501.6 mg) were added. The reaction mixture was then heated to a temperature of 60 °C and stirred for 48 hours. After the reaction was completed, the reaction mixture was filtered through short silica gel, and then the solvent was removed under reduced pressure. The bifuran product was purified by flash column chromatography (silica gel, petroleum ether/AcOEt = 100:1) to yield **2F34-S1**.

Under nitrogen atmosphere, to a solution of **2F34-S1** (6.6 mmol, 3.9 g) in dry toluene (0.1 M), Au(PPh_3)Cl (5 mol%, 163 mg) and AgOTf (5 mol%, 84.4 mg) were added. The reaction mixture was then heated to a temperature of 90 °C and stirred for 24 h. After the reaction was completed, the reaction mixture was filtered through short silica gel, and then the solvent was removed under reduced pressure. The bifuran product was purified by flash column chromatography (silica gel, petroleum ether/AcOEt = 10:1) to yield **2F34-Si** and **2F34-Si'**.

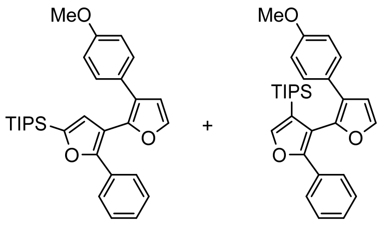

triisopropyl(3-(4-methoxyphenyl)-2'-phenyl-[2,3'-bifuran]-5'-yl)silane (2F34-Si) and triisopropyl(3-(4-methoxyphenyl)-2'-phenyl-[2,3'-bifuran]-4'-yl)silane (2F34-Si')

Yield: 2.5 g, 66% for two steps, α:β = 67:33, yellow oil, R_f = 0.35 (petroleum ether/AcOEt =20:1).

^{1}H NMR (500 MHz, CDCl$_3$) δ 7.53 (d, J = 7.8 Hz, 2H), 7.49 (d, J = 2.1 Hz, 1H), 7.31 (d, J = 8.7 Hz, 2H), 7.27 (s, 1H), 7.21 (d, J = 7.3 Hz, 1H), 6.77 – 6.74 (m, 2H), 6.71 (d, J = 1.9 Hz, 1H), 6.65 (s, 1H), 3.76 (s, 3H), 1.13 (d, J = 7.5 Hz, 21H).

^{13}C NMR (126 MHz, CDCl$_3$) δ 158.4, 156.4, 154.9, 141.7, 131.0, 128.5, 128.3, 127.6, 125.5, 125.4, 113.8, 111.6, 77.3, 77.0, 76.8, 55.3, 18.7, 18.6, 11.3, 11.0.

IR (KBr, cm^{-1}) 3542, 3109, 2835, 2521, 1306, 1141, 1015, 725, 509.

HRMS (ESI) ([M+H]$^+$) Calcd. for [$C_{30}H_{37}O_3Si$]$^+$: 473.2506, Found. 473.2501.

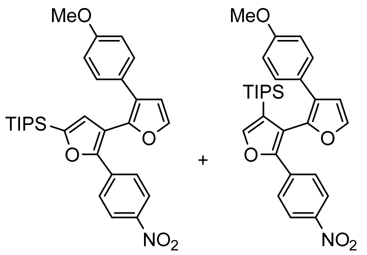

triisopropyl(3-(4-methoxyphenyl)-2'-(4-nitrophenyl)-[2,3'-bifuran]-5'-yl)silane (2F35-Si) and triisopropyl(3-(4-methoxyphenyl)-2'-(4-nitrophenyl)-[2,3'-bifuran]-4'-yl)silane (2F35-Si')

Yield: 52 mg, 50% for two steps, α:β = 83:17, brown oil, R_f = 0.35 (petroleum ether/AcOEt =20:1).

^{1}H NMR (500 MHz, CDCl$_3$) δ 8.13 – 8.07 (m, 2H), 8.06 (s, 0.12H), 8.04 – 8.00 (m, 0.61H), 7.66 – 7.60 (m, 2H), 7.58 – 7.56 (m, 0.55H), 7.53 (d, J = 1.9 Hz, 1H), 7.47 (d, J = 9.0 Hz, 0.58H), 7.31 (d, J = 9.7 Hz, 0.16H), 7.28 (s, 1H), 7.14 – 7.05 (m, 0.5H), 6.78 – 6.73 (m, 3H), 6.72 (d, J = 1.9 Hz, 1H), 6.69 – 6.65 (m, 0.46H), 3.74 (s, 3H), 3.69 (s, 0.64H), 1.34 (p, J = 7.4 Hz, 3H), 1.14 (d, J = 7.5 Hz, 18H), 1.10 (s, 0.6H), 0.97 (d, J = 7.1 Hz, 3.6H).

^{13}C NMR (126 MHz, CDCl$_3$) δ 159.3, 158.8, 158.5, 151.9, 150.7, 150.2, 148.7, 146.4, 146.3, 142.4, 142.3, 141.5, 141.2, 139.5, 136.6, 136.1, 129.7, 128.6, 127.8, 125.7, 125.4, 125.23, 125.16, 124.3, 123.9, 123.8, 123.7, 119.7, 119.3, 115.7, 114.0, 113.9, 112.1, 111.7, 55.24, 55.18, 18.7, 18.6, 11.6, 11.0.

IR (KBr, cm^{-1}) 3014, 2823, 2523, 1772, 1624, 1406, 1115, 1025, 624, 522.

HRMS (ESI) ([M+H]$^+$) Calcd. for [$C_{30}H_{36}NO_5Si$]$^+$: 518.2357, Found. 518.2361.

6.2 The gram-scale synthesis of product 2F36-Si.

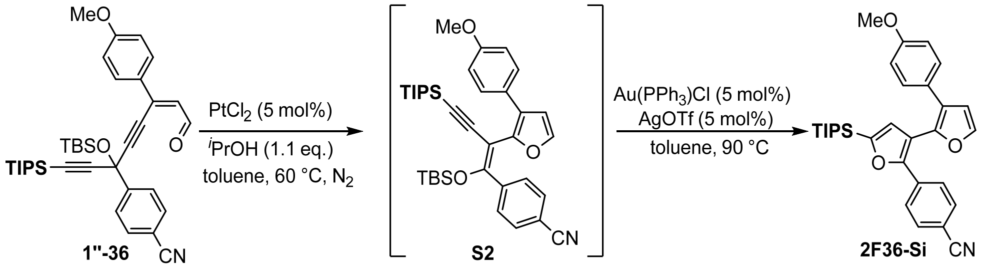

Under nitrogen atmosphere, to a solution of enynal **1''-36** (8.1 mmol, 5.0 g) in dry toluene (200 mL), $PtCl_2$(0.05 eq, 107.7 mg) and ^{i}PrOH (1.1 eq, 534.6 mg) were added. The reaction mixture was then heated to a temperature of 60 ℃ and stirred for 48 hours. After the reaction was completed, the reaction mixture was filtered through short silica gel, and then the solvent was removed under reduced pressure. The bifuran product was purified by flash column chromatography (silica gel, petroleum ether/AcOEt = 100:1) to yield **S2** (4.5 g, 90%).

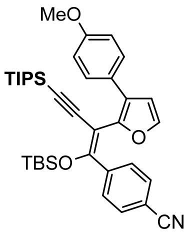

(E)-4-(1-((tert-butyldimethylsilyl)oxy)-2-(3-(4-methoxyphenyl)furan-2-yl)-4-(triisopropylsilyl)but-1-en-3-yn-1-yl)benzonitrile (S2)

Yield: 4.5 g, 90%, yellow oil, R_f = 0.35 (petroleum ether/AcOEt =10:1).

^{1}H NMR (500 MHz, $CDCl_3$) δ 7.88 (d, *J* = 8.0 Hz, 2H), 7.64 (d, *J* = 8.0 Hz, 2H), 7.48 – 7.41 (m, 3H), 6.90 (d, *J* = 8.3 Hz, 2H), 6.61 (d, *J* = 2.0 Hz, 1H), 3.82 (s, 3H), 0.86 (s, 21H), 0.70 (s, 9H), -0.28 (s, 6H).

^{13}C NMR (126 MHz, $CDCl_3$) δ 158.52, 158.48, 143.2, 142.1, 141.6, 131.4, 129.6, 128.7, 126.0, 123.5, 118.6, 113.7, 112.3, 111.4, 103.6, 100.2, 95.1, 55.3, 25.3, 18.5, 18.0, 11.2, -4.6.

IR (KBr, cm^{-1}) 2960, 2844, 2211, 1656, 1454, 1383, 1299, 1250, 1179, 1147, 1029, 830.

HRMS (ESI) ($[M+Na]^+$) Calcd. for $[C_{37}H_{50}NO_3Si_2]^+$: 612.3324, Found. 612.3316.

Under nitrogen atmosphere, to a solution of **S2** (3.5 mmol, 2.1 g) in dry toluene (0.1 M), $Au(PPh_3)Cl$ (5 mol%, 86.5 mg) and AgOTf (5 mol%, 44.8 mg) were added. The reaction mixture was then heated to a temperature of 90 ºC and stirred for 24 h. After the reaction was completed, the reaction mixture was filtered through short silica gel, and then the solvent was removed under reduced pressure. The bifuran product was purified by flash column chromatography (silica gel, petroleum ether/AcOEt = 10:1) to yield **2F36-Si** and **2F36-Si'**.

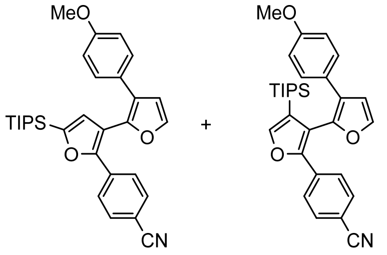

triisopropyl(3-(4-methoxyphenyl)-2'-(4-cyanophenyl)-[2,3'-bifuran]-5'-yl)silane (2F36-Si) and triisopropyl(3-(4-methoxyphenyl)-2'-(4-cyanophenyl)-[2,3'-bifuran]-4'-yl)silane (2F36-Si')

Yield: 1.6 g, 82% for two steps, α:β = 92:8, yellow solid, m.p. = 184 - 187 ℃, R_f = 0.33 (petroleum ether/AcOEt =10:1).

^{1}H NMR (500 MHz, $CDCl_3$) δ 7.57 (d, *J* = 8.6 Hz, 2H), 7.53 – 7.50 (m, 2H), 7.50 (s, 1H), 7.45 – 7.40 (m, 0.31H), 7.27 – 7.23 (m, 2H), 7.10 – 7.06 (m, 0.18H), 6.77 – 6.73 (m, 2H), 6.72 – 6.70 (m, 2H), 6.70 (s, 0.10H), 6.69 – 6.65 (m, 0.19H), 3.76 (s, 3H), 3.71 (s, 0.15H), 1.40 – 1.28 (m, 3H), 1.26 (d, *J* = 2.1 Hz, 0.15H), 1.13 (d, *J* = 7.5 Hz, 18H), 0.96 (d, *J* = 6.9 Hz, 0.9 H).

^{13}C NMR (126 MHz, CDCl$_3$) δ 158.8, 158.7, 152.2, 149.8, 142.3, 142.2, 141.3, 134.8, 132.2, 132.1, 128.5, 127.8, 125.6, 125.30, 125.27, 124.1, 123.7, 119.0, 115.0, 114.0, 113.9, 112.0, 111.7, 110.5, 110.3, 55.3, 55.2, 18.7, 18.6, 11.6, 11.0.
IR (KBr, cm^{-1}) 2915, 2829, 2201, 1924, 1827, 1615, 1439, 1223, 815, 641, 532.
HRMS (ESI) ([M+Na]$^+$) Calcd. for [$C_{31}H_{35}NNaO_3Si$]$^+$: 520.2284, Found. 520.2277.

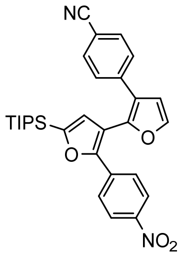

4-(2'-(4-nitrophenyl)-5'-(triisopropylsilyl)-[2,3'-bifuran]-3-yl)benzonitrile (2F37-Si)
Yield: 52 mg, 51% for two steps, α:β = 100:0, yellow solid, m.p. = 172 - 174 ℃, R_f = 0.35 (petroleum ether/AcOEt =10:1).
^{1}H NMR (500 MHz, CDCl$_3$) δ 8.16 – 8.12 (m, 2H), 7.62 (d, J = 2.1 Hz, 1H), 7.60 (d, J = 2.1 Hz, 2H), 7.53 – 7.49 (m, 2H), 7.46 – 7.43 (m, 2H), 6.79 (d, J = 2.0 Hz, 1H), 6.68 (s, 1H), 1.39 – 1.31 (m, 3H), 1.14 (d, J = 7.5 Hz, 18H).
^{13}C NMR (126 MHz, CDCl$_3$) δ 160.1, 152.7, 146.7, 143.5, 143.1, 137.5, 136.1, 132.3, 127.8, 125.7, 125.3, 124.0, 123.9, 122.7, 118.7, 114.5, 111.6, 110.5, 18.5, 10.9.
IR (KBr, cm^{-1}) 2936, 2846, 1910, 1827, 1638, 1521, 1228, 1049, 891, 744, 522.
HRMS (ESI) ([M+H]$^+$) Calcd. for [$C_{30}H_{33}N_2O_4Si$]$^+$: 513.2204, Found. 513.2195.

7. General procedure for the bromination of bifurans.

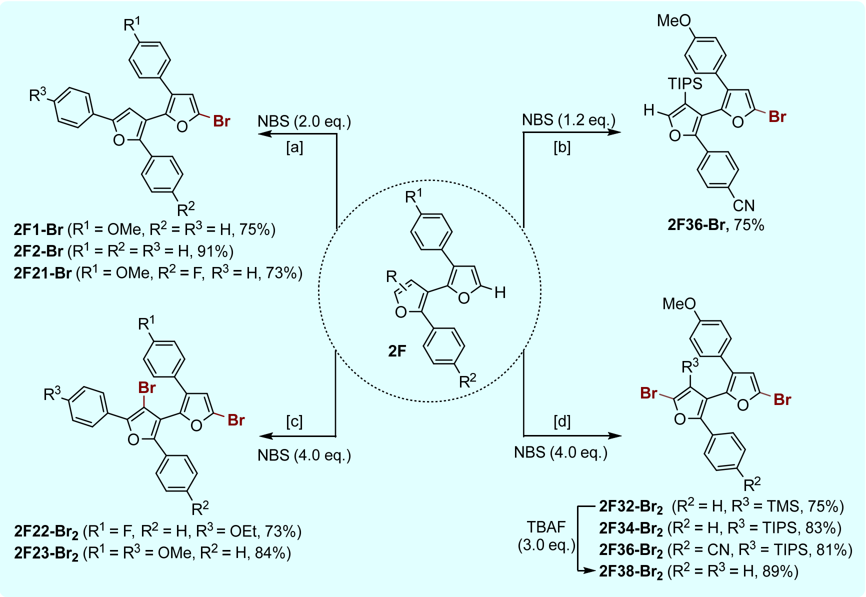

Supplementary Figure 1. The bromination of bifurans. See below for details.

The bromination of bifurans reaction selectivity could be easily tuned by simply changing the NBS equivalents. Typically, the α-position of bifuran showed higher reactivity than β-position. For the non-silyl bifuran, there are only one free α-position open for bromination. When two equivalents of NBS were applied, the α-bromo-difuran **2F-Br** could be obtained in good to excellent yields (**2F1-Br**, **2F2-Br**, **2F21-Br**). Surprisingly, two bromine atoms could be introduced when four equivalents

of NBS were used and giving the desired dibromoproducts **2F22-Br$_2$** and **2F23-Br$_2$** in excellent yields (the second bromine was installed at the β-position). When β-silyl bifurans were applied as the starting materials, in which there are two α- positions open for modifications, one or two bromine atoms can also be selectively introduced under similar reaction conditions. The silyl group is very important for modulating the electronic and steric properties of the bifuran, which endows two furans with quite different reactivity towards bromination. For example, when 1.2 equivalent of NBS was applied, the bromination selectively took placed at the non-silyl furan ring (**2F36-Br**). Increasing the NBS amount to four equivalents will result in dibromination at both α-positions (**2F32-Br$_2$**, **2F34-Br$_2$** and **2F36-Br$_2$**). The silyl-dibromobifuran **2F32-Br$_2$** could be easily desilylated with TBAF, giving the 2,2'-dibromo-α,β'-bifuran **2F38-Br$_2$** in 89% yield.

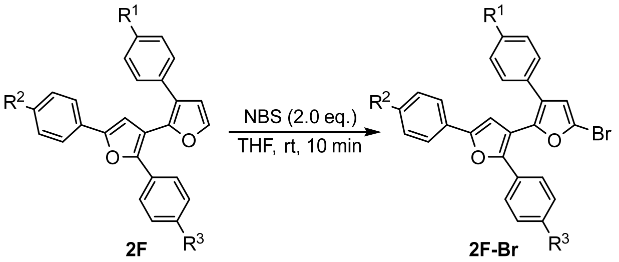

Under nitrogen atmosphere, N-Bromosuccinimide (NBS; 2.0 eq) was added into a solution of **2F** (0.2 mmol, 1.0 eq.) in THF (4 mL) and the reaction mixture was stirred at room temperature for10 min. Triethylamine (5 mL) was then added and the solvent was evaporated. The product was further purified by flash column chromatography on silica gel with petroleum ether/ethyl acetate (50:1) as the eluent to yield compound **2F-Br** (73 - 91%) as a colorless oil.

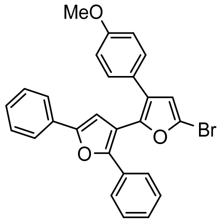

5-bromo-3-(4-methoxyphenyl)-2',5'-diphenyl-2,3'-bifuran (2F1-Br)

Yield: 53 mg, 75%, yellow oil, R_f = 0.35 (petroleum ether/AcOEt =50:1).

^{1}H NMR (400 MHz, CDCl$_3$) δ 8.08 (d, J = 7.8 Hz, 1H), 7.71 (d, J = 7.7 Hz, 1H), 7.58 (d, J = 7.7 Hz, 1H), 7.53 (d, J = 7.6 Hz, 1H), 7.45 (t, J = 7.7 Hz, 1H), 7.38 (q, J = 7.1, 6.5 Hz, 1H), 7.33 – 7.18 (m, 7H), 6.78 (s, 1H), 6.75 (d, J = 4.6 Hz, 1H), 6.71 (s, 1H), 3.71 (s, 6H).

^{13}C NMR (101 MHz, CDCl$_3$) δ 159.1, 159.0, 152.8, 151.6, 150.6, 148.1, 143.7, 141.1, 130.2, 130.2, 129.4, 129.2, 128.81, 128.77, 128.7, 128.6, 128.5, 128.42, 128.39, 128.0, 127.9, 126.0, 125.65, 125.56, 125.2, 124.4, 124.1, 124.0, 123.2, 121.8, 114.9, 114.2, 114.1, 113.3, 113.2, 112.9, 108.8, 101.3, 55.2.

IR (KBr, cm^{-1}) 3057, 2942, 2865, 2740, 2207, 1563, 1447, 1380, 1234, 1011, 863.

HRMS (ESI) ([M+H]$^+$) Calcd. for [$C_{27}H_{20}BrO_3$]$^+$: 471.0590, Found. 471.0597.

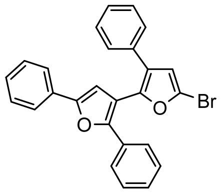

5-bromo-2',3,5'-triphenyl-2,3'-bifuran (2F2-Br)

Yield: 86 mg, 91%, yellow oil, R_f = 0.35 (petroleum ether/AcOEt =50:1).

^{1}H NMR (500 MHz, CDCl$_3$) δ 7.61 (d, J = 7.8 Hz, 2H), 7.48 (d, J = 7.7 Hz, 2H), 7.30 (t, J = 7.6 Hz, 2H), 7.23 (d, J = 7.4 Hz, 2H), 7.20 – 7.14 (m, 3H), 7.14 – 7.09 (m, 3H), 7.09 – 7.02 (m, 1H), 6.62 (s, 1H), 6.56 (s, 1H).

^{13}C NMR (126 MHz, CDCl$_3$) δ 152.9, 150.7, 144.5, 132.21, 130.15, 130.2, 128.8, 128.6, 128.4, 128.0, 127.9, 127.4, 127.3, 126.3, 125.6, 113.3, 113.0, 108.8.

IR (KBr, cm^{-1}) 3057, 2942, 2865, 2740, 2207, 1563, 1447, 1380, 1234, 1011, 863.

HRMS (ESI) ([M+Na]$^+$) Calcd. for $[C_{26}H_{17}BrNaO_2]^+$: 463.0304, Found. 463.0303.

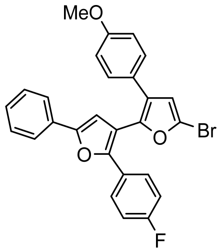

5-bromo-2'-(4-fluorophenyl)-3-(4-methoxyphenyl)-5'-phenyl-2,3'-bifuran (2F21-Br)

Yield: 71 mg, 73%, yellow oil, R_f = 0.35 (petroleum ether/AcOEt =50:1).

^{1}H NMR (400 MHz, CDCl$_3$) δ 7.74 (d, $J_{C\text{-}F}$ = 7.5 Hz, 2H), 7.62 – 7.52 (m, 2H), 7.44 (t, $J_{C\text{-}F}$ = 7.6 Hz, 2H), 7.38 – 7.22 (m, 3H), 6.99 (t, $J_{C\text{-}F}$ = 8.7 Hz, 2H), 6.78 (d, $J_{C\text{-}F}$ = 8.3 Hz, 3H), 6.64 (s, 1H), 3.78 (s, 3H).

^{13}C NMR (101 MHz, CDCl$_3$) δ 162.45 (d, J = 248.6 Hz), 159.0, 152.9, 149.5, 143.6, 130.1, 128.8, 128.4, 127.9, 127.49 (d, J = 8.1 Hz), 126.58 (d, J = 3.1 Hz), 125.97, 124.3, 123.9, 121.9, 115.38 (d, J = 21.8 Hz), 114.0, 113.3, 112.9, 108.7, 55.3.

^{19}F NMR (376 MHz, CDCl$_3$) δ -112.94.

IR (KBr, cm^{-1}) 3022, 2954, 2741, 2593, 2206, 1781, 1327, 1206, 1182, 981, 873

HRMS (ESI) ([M+Na]$^+$) Calcd. for $[C_{27}H_{18}BrFNaO_3]^+$: 511.0316, Found. 511.0323.

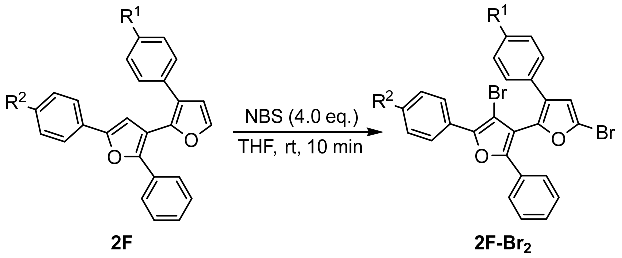

2F **2F-Br$_2$**

Under nitrogen atmosphere, N-Bromosuccinimide (NBS; 4.0 eq.) was added into a solution of **2F** (0.2 mmol, 1.0 eq.) in THF (4 mL) and the reaction mixture was stirred at room temperature for 10 min. Triethylamine (5 mL) was then added and the solvent was evaporated. The product was further purified by flash column chromatography on silica gel with petroleum ether/ethyl acetate (50:1) as the eluent to yield compound **2Fx-Br$_2$** and **2Fw-Br$_2$**.

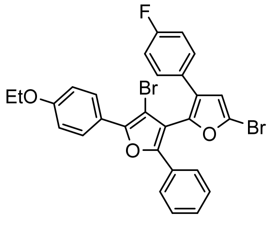

4',5-dibromo-5'-(4-ethoxyphenyl)-3-(4-fluorophenyl)-2'-phenyl-2,3'-bifuran (2F22-Br$_2$)

Yield: 71 mg, 73%, white solid, m.p. = 172 - 174 ℃, R_f = 0.35 (petroleum ether/AcOEt =30:1).

^{1}H NMR (500 MHz, CDCl$_3$) δ 8.01 – 7.97 (m,2H), 7.53 – 7.43 (m,2H), 7.32 – 7.22 (m, 5H), 6.99 – 6.94 (m, 2H), 6.94 – 6.88 (m, 2H), 6.69 (s,1H), 4.06 (q, J = 7.0 Hz, 2H), 1.42 (t, J = 7.0 Hz, 3H).

^{13}C NMR (126 MHz, CDCl$_3$) δ 162.17 (d, $J_{C\text{-}F}$ = 247.3 Hz), 159.2, 151.0, 148.6, 142.0, 129.2, 128.8, 128.6, 128.54 (d, $J_{C\text{-}F}$ = 8.0 Hz), 127.83 (d, $J_{C\text{-}F}$ = 1.8 Hz), 127.2, 125.1, 123.5, 121.9, 115.70 (d, $J_{C\text{-}F}$ = 21.6 Hz), 114.6, 114.2, 112.9, 99.2, 14.8.

^{19}F NMR (471 MHz, CDCl$_3$) δ -114.1.

IR (KBr, cm^{-1}) 3078, 2951, 2858, 2209, 1661, 1565, 1388, 1235, 967, 846, 653.

HRMS (ESI) ([M+Na]$^+$) Calcd. for [$C_{28}H_{19}Br_2FNaO_3$]$^+$: 602.9577, Found. 602.9576.

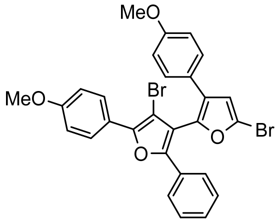

4',5-dibromo-3,5'-bis(4-methoxyphenyl)-2'-phenyl-2,3'-bifuran (2F23-Br$_2$)

Yield: 101 mg, 84%, white solid, m.p. = 166 - 168 ℃, R_f = 0.35 (petroleum ether/AcOEt =50:1).

^{1}H NMR (500 MHz, CDCl$_3$) δ 8.04 – 7.98 (m, 2H), 7.55 – 7.47 (m, 2H), 7.33 – 7.21 (m, 5H), 6.98 (dd, J = 9.1, 2.1 Hz, 2H), 6.78 (d, J = 2.4 Hz, 1H), 6.76 (d, J = 2.2 Hz, 1H), 6.73 – 6.68 (m, 1H), 3.84 (s, 3H), 3.71 (s, 3H).

^{13}C NMR (126 MHz, CDCl$_3$) δ 159.8, 159.0, 151.0, 148.3, 141.3, 129.3, 128.7, 128.5, 128.4, 128.0, 127.2, 125.1, 124.2, 123.1, 122.2, 114.7, 114.2, 114.1, 112.8, 99.7, 55.4, 55.2.

IR (KBr, cm^{-1}) 3060, 2923, 2855, 2212, 1661, 1565, 1388, 1235, 993, 689.

HRMS (ESI) ([M+Na]$^+$) Calcd. for [$C_{28}H_{20}Br_2NaO_4$]$^+$: 600.9621, Found. 600.9622.

[c] The selective monobromination of 2F36-Si'.

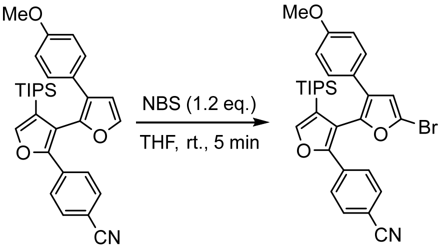

2F36-Si' **2F36-Br**, 75%

Under nitrogen atmosphere, N-Bromosuccinimide (NBS, 34 mg, 1.2 eq.) was added into a solution of **2F36-Si'** (0.16 mmol, 79.6 mg, 1.0 eq.) in THF (4 mL) and the reaction mixture was stirred at room temperature for 10 min. triethylamine (5 mL) was then added and the solvent was evaporated. he obtained crude product was purified with silica gel column chromatography (petroleum ether/AcOEt = 20:1) to afford **2F36-Br** (69.0 mg, 75%) as yellow oil.

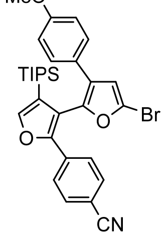

4-(5-bromo-3-(4-methoxyphenyl)-4'-(triisopropylsilyl)-[2,3'-bifuran]-2'-yl)benzonitrile (2F36-Br)
Yield: 69 mg, 75%, yellow oil, R_f = 0.35 (petroleum ether/AcOEt =30:1).
^{1}H NMR (500 MHz, CDCl$_3$) δ 7.53 (s, 1H), 7.49 – 7.43 (m, 4H), 7.09 – 6.96 (m, 2H), 6.66 (d, *J* = 8.8 Hz, 2H), 6.62 (s, 1H), 3.70 (s, 3H), 1.09 – 0.89 (m, 21H).
^{13}C NMR (126 MHz, CDCl$_3$) δ 158.9, 150.9, 149.9, 143.7, 134.1, 132.2, 127.8, 126.7, 125.5, 124.1, 122.2, 119.0, 118.8, 117.8, 114.0, 112.9, 110.8, 55.2, 18.7, 11.7.
IR (KBr, cm^{-1}) 2941, 2876, 2613, 2534, 2271, 1937, 1782, 1498, 954, 642.
HRMS (ESI) ([M+H]$^+$) Calcd. for [$C_{31}H_{35}BrNO_3Si$]$^+$: 576.1564, Found. 576.1565.

[d] The dibromination of silylated bifurans.

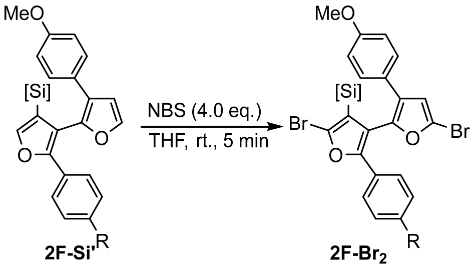

2F-Si' 2F-Br$_2$

Under nitrogen atmosphere, N-Bromosuccinimide (NBS, 4.0 eq) was added into a solution of **2F-Si'** (0.2 mmol, 1.0 eq.) in THF (4 mL) and the reaction mixture was stirred at room temperature for 10 min. Triethylamine (5 mL) was then added and the solvent was evaporated. The product was further purified by flash column chromatography on silica gel with petroleum ether/ethyl acetate (50:1) as the eluent to yield compound **2F-Br$_2$** as yellow solid.

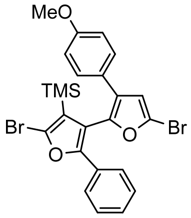

(5,5'-dibromo-3-(4-methoxyphenyl)-2'-phenyl-[2,3'-bifuran]-4'-yl)trimethylsilane (2F32-Br$_2$)
Yield: 90 mg, 75%, yellow solid, m.p. = 158 - 160 ℃, R_f = 0.35 (petroleum ether/AcOEt =30:1).
^{1}H NMR (500 MHz, CDCl$_3$) δ 7.34 – 7.27 (m, 2H), 7.18 – 7.10 (m, 4H), 7.09 – 7.05 (m, 2H), 6.69 – 6.65 (m, 2H), 6.57 (d, *J* = 1.9 Hz, 1H), 3.64 (s, 3H), -0.00 (s, 9H).
^{13}C NMR (126 MHz, CDCl$_3$) δ 160.1, 155.9, 144.3, 130.2, 129.7, 129.4, 129.0, 128.3, 126.2, 125.2, 123.33, 123.28, 118.0, 115.3, 113.7, 56.3, 0.0.
IR (KBr, cm^{-1}) 3061, 2939, 2845, 2213, 1727, 1660, 1446, 1241, 969, 763.
HRMS (ESI) ([M+Na]$^+$) Calcd. for [$C_{24}H_{22}Br_2NaO_3Si$]$^+$: 566.9597, Found. 566.9583.

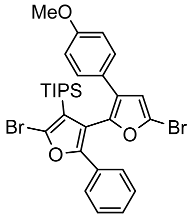

(5,5'-dibromo-3-(4-methoxyphenyl)-2'-phenyl-[2,3'-bifuran]-4'-yl)triisopropylsilane (2F34-Br$_2$)
Yield: 108 mg, 83%, yellow solid, m.p. = 174-176 ℃, R_f = 0.33 (petroleum ether/AcOEt =30:1).

^{1}H NMR (500 MHz, $CDCl_3$) δ 7.39 – 7.32 (m, 2H), 7.27 – 7.16 (m,3H), 7.11 – 7.04 (m, 2H), 6.81 – 6.65 (m, 2H), 6.62 (s, 1H), 3.71 (s, 3H), 1.23 – 1.13 (m, 3H), 1.06 (s, 9H), 0.92 (s, 9H).

^{13}C NMR (126 MHz, $CDCl_3$) δ 158.8, 155.6, 143.2, 129.4, 129.1, 128.6, 128.4, 127.9, 127.0, 125.3, 124.2, 121.9, 118.7, 117.5, 114.0, 112.7, 55.2, 18.9, 11.7.

IR (KBr, cm^{-1}) 3069, 2923, 1662, 1501, 1303, 1149, 1054, 966, 740.

HRMS (ESI) ($[M+Na]^+$) Calcd. for $[C_{30}H_{34}Br_2NaO_3Si]^+$: 651.0536, Found. 651.0545.

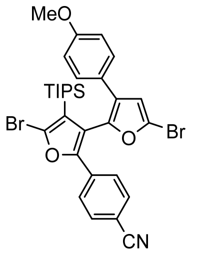

4-(5,5'-dibromo-3-(4-methoxyphenyl)-4'-(triisopropylsilyl)-[2,3'-bifuran]-2'-yl)benzonitrile (2F36-Br_2)

Yield: 106 mg, 81%, yellow solid, m.p. = 191-193 ℃, R_f = 0.33 (petroleum ether/AcOEt =10:1).

^{1}H NMR (500 MHz, $CDCl_3$) δ 7.52 – 7.47 (m, 2H), 7.44 – 7.36 (m, 2H), 7.11 – 6.99 (m, 2H), 6.78 – 6.67 (m, 2H), 6.65 (s, 1H), 3.72 (s, 3H), 1.27 – 1.18 (m, 3H), 1.07 (s, 9H), 0.94 (s, 9H).

^{13}C NMR (126 MHz, $CDCl_3$) δ 159.0, 153.1, 142.1, 132.8, 132.4, 131.4, 127.7, 127.5, 125.3, 123.6, 122.6, 120.6, 119.8, 118.6, 114.1, 112.8, 111.4, 55.2, 18.8, 11.7.

IR (KBr, cm^{-1}) 3029, 2937, 2781, 2345, 2118, 1901, 1782, 1298, 924, 746.

HRMS (ESI) ($[M+H]^+$) Calcd. for $[C_{31}H_{34}Br_2NO_3Si]^+$: 654.0669, Found. 654.0661.

Desilication of bifurans.

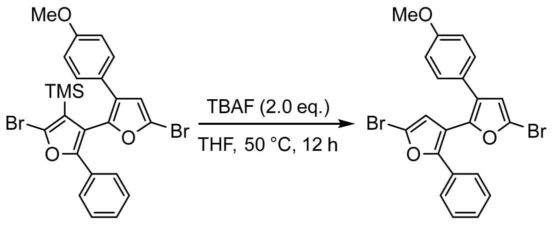

2F32-Br_2 **2F38-Br_2**

A mixture of **2F32-Br_2** (0.2 mmol) and TBAF (1.0 M in THF, 0.4 mL, 0.4 mmol) was stirred in THF (4 mL) at 50 ℃ for 12 h. The reaction was quenched with sat. NH_4Cl (5 mL). The aqueous phase was extracted with AcOEt (3 × 20 mL). The combined organic layer was washed with brine (10 mL) and dried over $MgSO_4$. The solvents were evaporated in vacuo. The obtained crude product was purified with silica gel column chromatography (petroleum ether/AcOEt = 50:1) to afford **2F38-Br_2** (89%) as yellow oil.

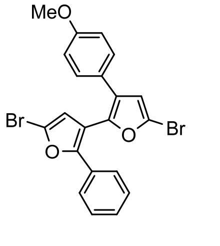

5,5'-dibromo-3-(4-methoxyphenyl)-2'-phenyl-2,3'-bifuran (2F38-Br_2)

Yield: 84 mg, 89%, yellow oil, R_f = 0.33 (petroleum ether/AcOEt =30:1).

^{1}H NMR (500 MHz, CDCl$_3$) δ 7.38 (d, *J* = 6.6 Hz, 2H), 7.19 – 7.14 (m, 3H), 7.11 (d, *J* = 8.5 Hz, 2H), 6.68 (d, *J* = 8.4 Hz, 2H), 6.50 (s, 1H), 6.30 (s, 1H), 3.68 (s, 3H).

^{13}C NMR (126 MHz, CDCl$_3$) δ 159.1, 153.2, 142.4, 129.4, 128.5, 128.3, 126.5, 125.5, 124.1, 122.1, 121.9, 114.7, 114.1, 113.40, 113.36, 55.3.

IR (KBr, cm^{-1}) 3192, 2937, 2519, 2204, 2016, 1863, 1626, 1305, 993, 728.

HRMS (ESI) ([M+H]$^+$) Calcd. for [$C_{21}H_{15}Br_2O_3$]$^+$: 472.9382, Found. 472.9380.

8. General procedure for the tinification of bifurans.

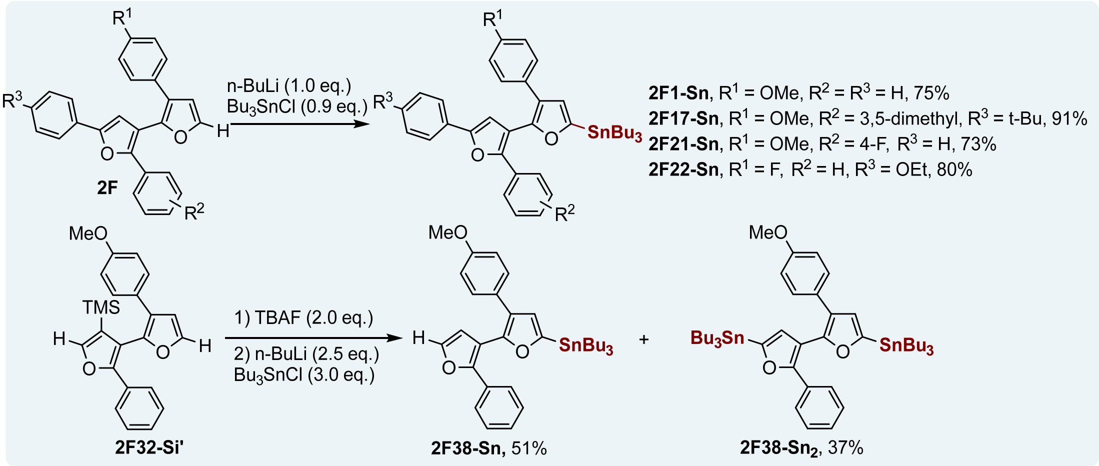

Supplementary Figure 2. The tinification of bifurans.

The stannylation reactions were similar with the bromination with Bu_3SnCl as the tin reagent. As shown in Supplementary Fig. 2, the tributyltin bifurans **2F-Sn** could be obtained in 73-91% yields through the lithiation of **2F** with n-butyllithium followed by the addition of tributyltin chloride. When 3-silyl-bifuran was applied as the starting material, after desilylation with TBAF, one or two tributyltin groups could be installed at the α-positions under BuLi/Bu_3SnCl conditions. These bromo-bifurans and tin-bifurans could be utilized as highly useful coupling partners in the synthesis of oligofurans through Stille coupling reactions.

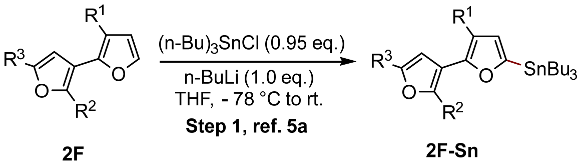

A 2.5 M solution of n-BuLi in hexane (0.2 mL, 0.5 mmol, 1.0 eq.) was added dropwise into a solution of **2F** (0.5 mmol) in dry THF (10 mL) at -78 °C under N_2. The reaction mixture was stirred for 20 min at -78 °C. Bu_3SnCl (0.48 mmol, 0.95 eq.) was then added dropwise and the reaction mixture was allowed to reach room temperature and stirred for 2 h. The mixture was quenched with water, extracted with hexane, dried ($MgSO_4$), and evaporated. Repetitive flash chromatography on basified (NEt_3) silica using hexane as eluent gave pure **2F-Sn** as a light green oil (83-91% yield)

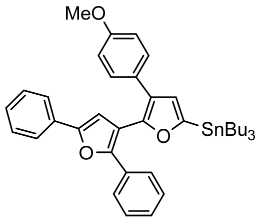

tributyl(3-(4-methoxyphenyl)-2',5'-diphenyl-[2,3'-bifuran]-5-yl)stannane (2F1-Sn)
Yield: 256 mg, 75%, yellow oil, R_f = 0.33 (petroleum ether/AcOEt =50:1).
^{1}H NMR (400 MHz, CDCl$_3$) δ 7.68 (d, *J* = 7.1 Hz, 2H), 7.61 (d, *J* = 7.0 Hz, 2H), 7.39 – 7.31 (m, 4H), 7.29 – 7.16 (m, 4H), 6.85 (s, 2H), 6.77 (d, *J* = 8.7 Hz, 1H), 6.70 – 6.62 (m, 2H), 3.69 (s, 3H), 1.64 – 1.51 (m, 6H), 1.40 – 1.26 (m, 6H), 1.15 – 1.05 (m, 6H), 0.96 – 0.80 (m, 9H).
^{13}C NMR (101 MHz, CDCl$_3$) δ 160.5, 158.5, 152.6, 150.3, 146.6, 130.9, 130.6, 128.82, 128.76, 128.3, 127.7, 127.6, 126.3, 125.8, 124.0, 123.9, 123.4, 115.1, 114.0, 108.9, 55.3, 55.2, 29.1, 27.3, 13.8, 10.3.
IR (KBr, cm^{-1}) 3059, 2926, 2859, 1700, 1508, 1453, 1252, 1054, 955, 809, 698.
HRMS (ESI) ([M+Na]$^+$) Calcd. for [$C_{39}H_{46}NaO_3Sn$]$^+$: 705.2361, Found. 705.2357.

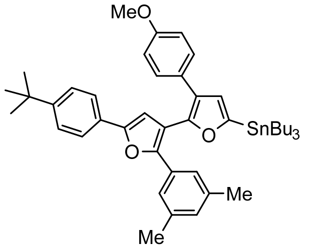

tributyl(5'-(4-(tert-butyl)phenyl)-2'-(3,5-dimethylphenyl)-3-(4-methoxyphenyl)-[2,3'-bifuran]-5-yl)stannane (2F17-Sn)
Yield: 349 mg, 91%, yellow oil, R_f = 0.33 (petroleum ether/AcOEt =50:1).
^{1}H NMR (500 MHz, CDCl$_3$) δ 7.67 – 7.62 (m, 2H), 7.43 – 7.40 (m, 2H), 7.36 – 7.32 (m, 2H), 7.18 – 7.15 (m, 2H), 6.85 – 6.82 (m, 2H), 6.79 – 6.73 (m, 2H), 6.66 – 6.61 (m, 1H), 3.73 (s, 3H), 2.25 (s, 6H), 1.62 – 1.49 (m, 6H), 1.38 – 1.26 (m, 18H), 1.14 – 1.04 (m, 6H), 0.93 – 0.77 (m, 12H).
^{13}C NMR (126 MHz, CDCl$_3$) δ 160.2, 158.3, 152.5, 150.7, 150.3, 146.8, 137.5, 130.7, 129.3, 128.5, 128.0, 126.4, 125.7, 123.8, 123.6, 123.5, 123.0, 114.7, 113.9, 108.4, 55.2, 34.7, 31.3, 29.0, 27.3, 21.5, 13.7, 10.2.
IR (KBr, cm^{-1}) 2966, 2872, 2744, 2208, 1669, 1580, 1468, 1083, 999, 917.
HRMS (ESI) ([M+H]$^+$) Calcd. for [$C_{45}H_{59}O_3Sn$]$^+$: 767.3481, Found. 767.3481.

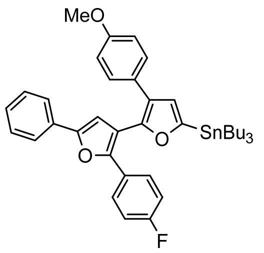

tributyl(2'-(4-fluorophenyl)-3-(4-methoxyphenyl)-5'-phenyl-[2,3'-bifuran]-5-yl)stannane (2F21-Sn)
Yield: 256 mg, 73%, yellow oil, R_f = 0.35 (petroleum ether/AcOEt =50:1).

^{1}H NMR (400 MHz, CDCl$_3$) δ 7.75 (dd, *J* = 7.3, 1.7 Hz, 2H), 7.65 – 7.59 (m, 2H), 7.47 – 7.38 (m, 4H), 7.35 – 7.29 (m, 1H), 7.06 – 6.98 (m,2H), 6.91 – 6.82 (m,3H), 6.74 (s, 1H), 3.82 (s, 3H), 1.69 – 1.58 (m, 6H), 1.45 – 1.34 (m, 6H), 1.20 – 1.10 (m, 6H), 0.95 (t, *J* = 7.3 Hz, 9H).
^{13}C NMR (101 MHz, CDCl$_3$) δ 163.27 (d, $J_{C\text{-}F}$ = 44.9 Hz), 161.0, 160.7, 158.5, 130.4, 128.71 (d, $J_{C\text{-}F}$ = 7.5 Hz), 127.7, 127.64 (d, $J_{C\text{-}F}$ = 4.0 Hz), 127.17 (d, $J_{C\text{-}F}$ = 3.3 Hz), 123.9, 123.3, 115.3, 115.1, 114.7, 113.9, 108.7, 55.3, 29.0, 27.2, 13.7, 10.3.
^{19}F NMR (376 MHz, CDCl$_3$) δ -113.8.
IR (KBr, cm^{-1}) 2939, 2863, 1710, 1633, 1441, 1228, 1137, 749.
HRMS (ESI) ([M+H]$^+$) Calcd. for [$C_{39}H_{46}FO_3Sn$]$^+$: 701.2447, Found. 701.2448.

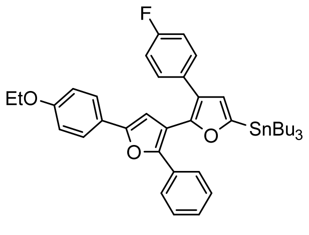

tributyl(5'-(4-ethoxyphenyl)-3-(4-fluorophenyl)-2'-phenyl-[2,3'-bifuran]-5-yl)stannane (2F22-Sn)
Yield: 286 mg, 80%, yellow oil, R$_f$ = 0.35 (petroleum ether/AcOEt =50:1).
^{1}H NMR (500 MHz, CDCl$_3$) δ 7.61 – 7.54 (m, 4H), 7.44 – 7.35 (m, 2H), 7.28 – 7.21 (m, 2H), 7.20 – 7.13 (m, 1H), 6.94 – 6.82 (m, 5H), 6.54 – 6.46 (m, 1H), 3.92 (q, *J* = 7.0 Hz, 2H), 1.69 – 1.53 (m, 6H), 1.42 – 1.27 (m, 9H), 1.20 – 1.04 (m, 6H), 1.02 – 0.82 (m, 9H).
^{13}C NMR (126 MHz, CDCl$_3$) δ 162.8, 159.92 (d, $J_{C\text{-}F}$ = 242.5 Hz), 158.9, 153.1, 153.1, 130.99, 131.96, 130.0, 129.23 (d, $J_{C\text{-}F}$ = 7.6 Hz), 128.4, 127.5, 125.56 (d, $J_{C\text{-}F}$ = 19.2 Hz), 123.8, 123.3, 122.75, 122.72, 115.39 (d, $J_{C\text{-}F}$ = 21.2 Hz), 114.9, 114.8, 107.1, 63.6, 29.2, 27.39, 27.37, 14.9, 13.9, 10.41, 10.39.
^{19}F NMR (471 MHz, CDCl$_3$) δ -115.6.
IR (KBr, cm^{-1}) 3061, 2930, 2856, 2743, 2212, 1958, 1662, 1566, 1451, 1389, 913, 852.
HRMS (ESI) ([M+Na]$^+$) Calcd. for [$C_{40}H_{47}FNaO_3Sn$]$^+$: 737.2423, Found. 737.2422.

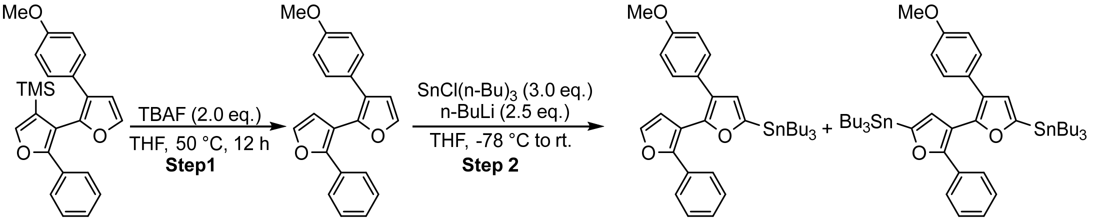

2F32-Si' **2F38** **2F38-Sn**, 51% **2F38-Sn$_2$**, 37%

Step 1: A mixture of **2F32-Si'** (0.2 mmol) and TBAF (1.0 M in THF, 0.4 mL, 0.4 mmol) was stirred in THF (4 mL) at 50 ℃ for 12 h. The reaction was quenched with sat. NH_4Cl (5 mL). The aqueous phase was extracted with AcOEt (3 × 20 mL). The combined organic layer was washed with brine (10 mL) and dried over $MgSO_4$. The solvents were evaporated in vacuo. The obtained crude product was purified with silica gel column chromatography (petroleum ether/AcOEt = 50:1) to afford **2F38** as yellow oil.

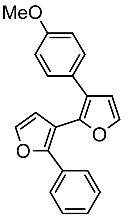

3-(4-methoxyphenyl)-2'-phenyl-2,3'-bifuran (2F38)

Yield: 48 mg, 75%, yellow oil, R_f = 0.33 (petroleum ether/AcOEt =30:1).

^{1}H NMR (500 MHz, CDCl$_3$) δ 7.45 – 7.40 (m, 3H), 7.38 (d, J = 1.8 Hz, 1H), 7.24 – 7.16 (m, 4H), 7.15 – 7.10 (m, 1H), 6.73 – 6.68 (m, 2H), 6.61 – 6.59 (m, 1H), 6.37 – 6.33 (m, 1H), 3.68 (s, 3H).

^{13}C NMR (126 MHz, CDCl$_3$) δ 158.6, 151.1, 141.9, 141.8, 141.6, 130.6, 128.6, 128.3, 127.8, 125.6, 123.2, 113.9, 113.7, 112.1, 111.8, 55.2.

IR (KBr, cm^{-1}) 2962, 2872, 2213, 1722, 1669, 1169, 1127, 1010, 908, 842.

HRMS (ESI) ([M+Na]$^+$) Calcd. for [$C_{21}H_{16}NaO_3$]$^+$: 339.0992, Found. 339.0990.

Step 2: A 2.5 M solution of n-BuLi in hexane (0.5 mL, 1.25 mmol, 2.5 eq.) was added dropwise into a solution of **2F38** (0.5 mmol) in dry THF (10 mL) at -78 °C under N_2. The reaction mixture was stirred for 20 min at -78 °C. (n-Bu)$_3$SnCl (414.4 μL, 1.5 mmol, 3.0 eq.) was then added dropwise and the reaction mixture was allowed to reach room temperature and stirred for 2 h. The mixture was quenched with water, extracted with hexane, dried ($MgSO_4$), and evaporated. Repetitive flash chromatography on basified (NEt_3) silica using petroleum as eluent gave pure **2F38-Sn** (155 mg, 51%) and **2F38-Sn$_2$** (166 mg, 37%) as light green oil.

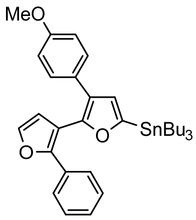

tributyl(3-(4-methoxyphenyl)-2'-phenyl-[2,3'-bifuran]-5-yl)stannane (2F38-Sn)

Yield: 155 mg, 51%, light green oil, R_f = 0.41 (petroleum).

^{1}H NMR (500 MHz, CDCl$_3$) δ 7.51 – 7.47 (m, 3H), 7.33 – 7.29 (m, 2H), 7.27 – 7.22 (m, 1H), 7.20 – 7.15 (m, 1H), 6.79 – 6.73 (m, 2H), 6.70 – 6.67 (m, 1H), 6.57 – 6.51 (m, 1H), 3.75 (s, 3H), 1.65 – 1.51 (m, 6H), 1.41 – 1.29 (m, 6H), 1.16 – 1.03 (m, 6H), 0.90 (t, J = 7.4 Hz, 9H).

^{13}C NMR (126 MHz, CDCl$_3$) δ 160.6, 158.4, 155.5, 142.7, 141.7, 131.1, 128.4, 128.2, 127.3, 125.8, 125.4, 125.2, 122.6, 113.8, 112.1, 111.6, 55.2, 29.0, 27.1, 13.7, 10.3.

IR (KBr, cm^{-1}) 2910, 2239, 1901, 1726, 1341, 1223, 1134, 1019, 826

HRMS (ESI) ([M+Na]$^+$) Calcd. for [$C_{33}H_{42}NaO_3Sn$]$^+$: 629.2048, Found. 629.2056.

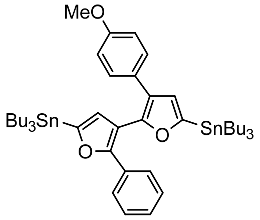

(3-(4-methoxyphenyl)-2'-phenyl-[2,3'-bifuran]-5,5'-diyl)bis(tributylstannane) (2F38-Sn$_2$)

Yield: 166 mg, 37%, light green oil, R_f = 0.35 (petroleum).

^{1}H NMR (500 MHz, CDCl$_3$) δ 7.53 – 7.50 (m, 2H), 7.34 – 7.30 (m, 2H), 7.23 (d, J = 7.8 Hz, 2H), 7.18 – 7.14 (m, 1H), 6.83 – 6.79 (m, 1H), 6.78 – 6.74 (m, 2H), 6.52 – 6.51 (m, 1H), 3.76 (s, 3H), 1.64 – 1.51 (m, 12H), 1.40 – 1.24 (m, 12H), 1.15 – 1.03 (m, 12H), 0.95 – 0.76 (m, 18H).

^{13}C NMR (126 MHz, CDCl$_3$) δ 160.1, 159.9, 158.2, 155.2, 147.4, 131.4, 128.5, 128.1, 127.1, 126.4, 125.5, 125.1, 123.6, 122.5, 113.7, 112.9, 55.2, 29.0, 27.2, 27.1, 13.7, 13.6, 10.3, 10.2.

IR (KBr, cm^{-1}) 2918, 2817, 2618, 2427, 2046, 1715, 1571, 1447, 1317, 1237, 649.
HRMS (ESI) ([M+H]$^+$) Calcd. for $[C_{45}H_{69}O_3Sn_2]^+$: 897.3285, Found. 897.3282.

9. General procedure for the synthetic of tetrafurans.

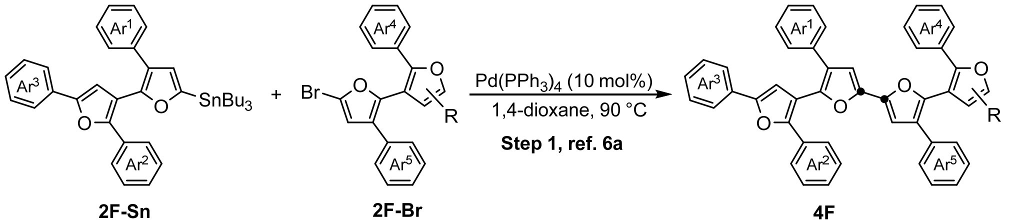

A mixture of **2F-Sn** (0.2 mmol, 1.0 eq.), **2F-Br** (0.22 mmol, 1.1 eq.), and $Pd(PPh_3)_4$ (0.02 mmol, 10 mol%) in dry dioxane (20 mL) was heated under nitrogen at 100 - 110 ℃ for 24 h. The solvent was evaporated under reduced pressure and the resulting residue was dissolved in ethyl acetate. This solution was passed through celite to remove Pd. The solution was evaporated, and the residue was chromatographed on silica gel using (70:30) as an eluent to furnish compound **4F** (63% – 93%).

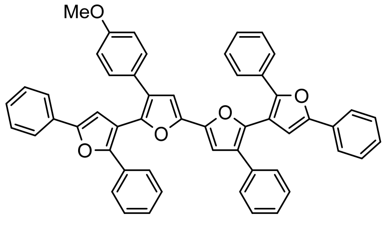

3'-(4-methoxyphenyl)-2,2''',4'',5,5'''-pentaphenyl-3,2':5',2'':5'',3'''-quaterfuran (4F2)
Yield: 127 mg, 84%, yellow solid, m.p. = 187 - 189 ℃, R_f = 0.33 (petroleum ether/AcOEt =20:1).
^{1}H NMR (400 MHz, CDCl$_3$) δ 7.79 – 7.64 (m, 8H), 7.41 (t, J = 7.9 Hz, 6H), 7.36 – 7.20 (m, 13H), 6.85 (d, J = 11.5 Hz, 2H), 6.81 – 6.74 (m, 4H), 3.75 (s, 3H).
^{13}C NMR (101 MHz, CDCl$_3$) δ 158.8, 152.8, 150.6, 145.6, 145.4, 141.3, 132.7, 130.52, 130.49, 130.2, 128.8, 128.7, 128.5, 128.3, 127.9, 127.8, 127.5, 127.1, 125.8, 125.5, 125.2, 124.0, 114.0, 113.9, 113.8, 108.8, 55.3.
IR (KBr, cm^{-1}) 2981, 2813, 2561, 2349, 2201, 1734, 1618, 1592, 1392, 1037, 649.
HRMS (ESI) ([M+Na]$^+$) Calcd. for $[C_{53}H_{36}NaO_5]^+$: 775.2455, Found. 775.2447.

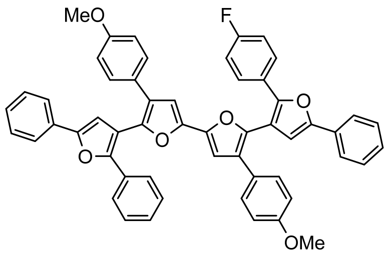

2-(4-fluorophenyl)-3',4''-bis(4-methoxyphenyl)-2''',5,5'''-triphenyl-3,2':5',2'':5'',3'''-quaterfuran (4F3)
Yield: 146 mg, 91%, yellow solid, m.p. = 151 - 153 ℃, R_f = 0.33 (petroleum ether/AcOEt =10:1).
^{1}H NMR (400 MHz, CDCl$_3$) δ 7.80 – 7.71 (m, 6H), 7.71 – 7.64 (m, 2H), 7.45 (t, J = 7.6 Hz, 4H), 7.41 – 7.30 (m, 9H), 7.01 (t, J = 8.6 Hz, 2H), 6.90 – 6.85 (m, 2H), 6.85 – 6.78 (m, 6H), 3.80 (s, 6H).
^{13}C NMR (101 MHz, CDCl$_3$) δ 158.87 (d, $J_{C\text{-}F}$ = 4.2 Hz), 152.8, 150.5, 149.4, 145.5, 145.4, 141.3, 141.0, 130.6, 130.21 (d, $J_{C\text{-}F}$ = 9.3 Hz), 128.72 (d, $J_{C\text{-}F}$ = 14.6 Hz), 128.3, 127.9, 127.9, 127.8, 127.64 (d, $J_{C\text{-}F}$ =

8.2 Hz), 125.7, 125.2, 125.1, 125.0, 124.0, 123.9, 115.4, 115.2, 114.0, 113.9, 113.7, 108.74, 108.66, 107.5, 55.3.
^{19}F NMR (376 MHz, CDCl$_3$) δ -113.1.
IR (KBr, cm^{-1}) 2942, 1657, 1505, 1436, 1225, 1090, 928, 841, 744.
HRMS (ESI) ([M+H]$^+$) Calcd. for [$C_{54}H_{38}FO_6$]$^+$: 801.2647, Found. 801.2654.

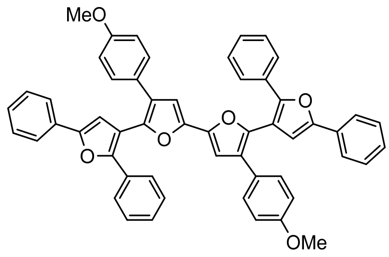

3',4''-bis(4-methoxyphenyl)-2,2''',5,5'''-tetraphenyl-3,2':5',2'':5'',3'''-quaterfuran (4F4)
Yield: 146 mg, 93%, yellow solid, m.p. = 167 - 169 ℃, R_f = 0.33 (petroleum ether/AcOEt =20:1).
^{1}H NMR (500 MHz, CDCl$_3$) δ 7.72 (dd, *J* = 17.6, 7.8 Hz, 8H), 7.41 (t, *J* = 7.6 Hz, 4H), 7.34 (d, *J* = 8.3 Hz, 4H), 7.30 (t, *J* = 7.5 Hz, 6H), 7.25 – 7.20 (m, 2H), 6.83 (s, 2H), 6.79 (s, 2H), 6.77 (s, 4H), 3.76 (s, 6H).
^{13}C NMR (126 MHz, CDCl$_3$) δ 158.8, 152.8, 150.4, 145.4, 141.1, 130.5, 130.2, 128.8, 128.6, 128.3, 127.9, 127.8, 125.7, 125.1, 125.1, 124.0, 114.0, 113.9, 108.8, 107.4, 55.3.
IR (KBr, cm^{-1}) 3062, 2938, 2856, 2743, 2683, 2212, 1958, 1564, 1387, 851.
HRMS (ESI) ([M+Na]$^+$) Calcd. for [$C_{54}H_{38}NaO_6$]$^+$: 805.2561, Found. 805.2562.

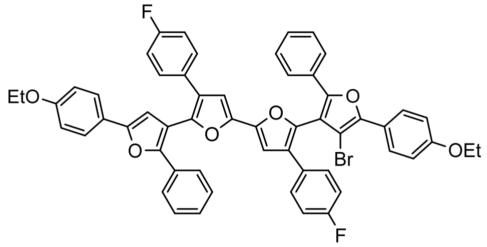

4-bromo-5,5'''-bis(4-ethoxyphenyl)-3',4''-bis(4-fluorophenyl)-2,2'''-diphenyl-3,2':5',2'':5'',3'''-quaterfuran (4F5)
Yield: 135 mg, 73%, yellow solid, m.p. = 134 - 136 ℃, R_f = 0.33 (petroleum ether/AcOEt =10:1).
^{1}H NMR (500 MHz, CDCl$_3$) δ 8.06 – 7.98 (m, 2H), 7.67 – 7.61 (m, 4H), 7.57 – 7.53 (m, 2H), 7.38 – 7.32 (m, 4H), 7.32 – 7.18 (m, 7H), 7.03 – 6.96 (m, 2H), 6.96 – 6.86 (m, 7H), 6.68 – 6.57 (m, 1H), 4.14 – 4.04 (m, 4H), 1.48 – 1.40 (m, 6H).
^{13}C NMR (126 MHz, CDCl$_3$) δ 1162.99 (d, $J_{C\text{-}F}$ = 6.5 Hz), 161.03 (d, $J_{C\text{-}F}$ = 6.5 Hz), 159.2, 158.9, 153.2, 150.9, 149.8, 148.6, 146.8, 145.4, 142.3, 139.5, 130.5, 129.4, 129.09 (d, $J_{C\text{-}F}$ = 8.0 Hz), 128.7, 128.53 (d, $J_{C\text{-}F}$ = 8.2 Hz), 128.41 (d, $J_{C\text{-}F}$ = 3.1 Hz), 128.3, 127.8, 127.3, 126.8, 125.54 (d, $J_{C\text{-}F}$ = 19.1 Hz), 125.0, 124.4, 123.0, 122.0, 115.7, 115.50 (d, $J_{C\text{-}F}$ = 3.2 Hz), 115.3, 114.9, 114.8, 114.6, 113.4, 108.0, 106.81 (d, $J_{C\text{-}F}$ = 19.6 Hz), 99.5, 77.3, 77.0, 76.8, 63.62, 63.60, 14.83, 14.81.
^{19}F NMR (471 MHz, CDCl$_3$) δ -114.7, -115.0.
IR (KBr, cm^{-1}) 2960, 2867, 2207, 1665, 1385, 1222, 1135, 999, 867, 821.
HRMS (ESI) ([M+Na]$^+$) Calcd. for [$C_{56}H_{39}BrF_2NaO_6$]$^+$: 947.1790, Found. 947.1802.

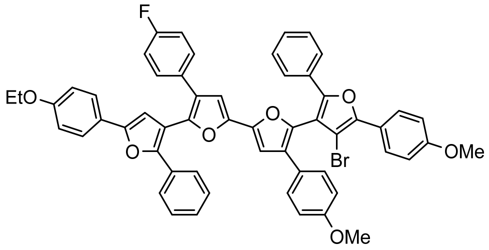

4-bromo-5'''-(4-ethoxyphenyl) -4''-(4-fluorophenyl)-3',5-bis(4-methoxyphenyl)-2,2'''-diphenyl-3,2':5',2'':5'',3'''-quaterfuran (4F6)

Yield: 112 mg, 66%, yellow solid, m.p. = 146 - 148 ℃, R_f = 0.33 (petroleum ether/AcOEt =10:1).

^{1}H NMR (500 MHz, CDCl$_3$) δ 8.07 – 8.03 (m, 2H), 7.64 (t, J = 7.6 Hz, 4H), 7.59 – 7.55 (m, 2H), 7.37 – 7.32 (m, 4H), 7.32 – 7.26 (m, 4H), 7.24 – 7.19 (m, 2H), 7.03 – 6.99 (m, 2H), 6.97 (s, 1H), 6.95 – 6.92 (m, 2H), 6.92 – 6.85 (m, 3H), 6.82 – 6.74 (m, 2H), 6.63 (s, 1H), 4.07 (q, J = 7.0 Hz, 2H), 3.87 (s, 3H), 3.74 (s, 3H), 1.43 (t, J = 6.9 Hz, 3H).

^{13}C NMR (126 MHz, CDCl$_3$) δ 161.97 (d, $J_{C\text{-}F}$ = 246.5 Hz), 159.7, 158.87 (d, $J_{C\text{-}F}$ = 6.3 Hz), 153.2, 150.8, 149.8, 148.3, 146.6, 145.6, 142.2, 138.7, 130.5, 129.5, 129.08 (d, $J_{C\text{-}F}$ = 8.0 Hz), 128.80 (d, $J_{C\text{-}F}$ = 3.2 Hz), 128.7, 128.4, 128.3, 128.0, 127.8, 127.4, 127.3, 125.6, 125.5, 125.1, 124.8, 124.4, 123.0, 122.3, 115.5, 115.3, 115.3, 114.8, 114.11, 114.10, 113.4, 107.7, 106.9, 106.8, 99.9, 77.3, 77.0, 76.8, 63.6, 55.4, 55.2, 14.8.

^{19}F NMR (471 MHz, CDCl$_3$) δ -115.1.

IR (KBr, cm^{-1}) 3061, 2938, 2857, 2212, 1661, 1565, 1342, 1142, 1005, 763.

HRMS (ESI) ([M+H]$^+$) Calcd. for [$C_{56}H_{41}BrFO_7$]$^+$: 923.2014, Found. 923.2019.

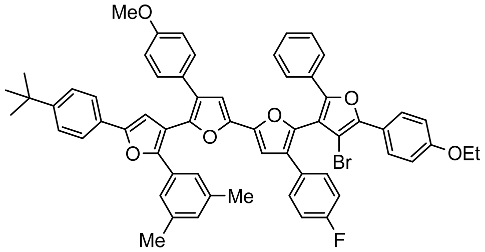

4-bromo-5'''-(4-(tert-butyl)phenyl)-2'''-(3,5-dimethylphenyl)-5-(4-ethoxyphenyl)-3'-(4-fluorophenyl)-4''-(4-methoxyphenyl)-2-phenyl-3,2':5',2'':5'',3'''-quaterfuran (4F7)

Yield: 150 mg, 75%, yellow solid, m.p. = 110 - 113 ℃, R_f = 0.33 (petroleum ether/AcOEt =10:1).

^{1}H NMR (500 MHz, CDCl$_3$) δ 7.95 – 7.89 (m, 2H), 7.56 (d, J = 8.4 Hz, 2H), 7.48 – 7.41 (m, 2H), 7.32 (d, J = 8.4 Hz, 2H), 7.27 – 7.20 (m, 6H), 7.16 (t, J = 7.5 Hz, 2H), 7.13 – 7.07 (m, 1H), 6.91 – 6.79 (m, 6H), 6.75 (s, 1H), 6.69 – 6.55 (m, 3H), 3.95 (q, J = 7.0 Hz, 2H), 3.61 (s, 3H), 2.14 (s, 6H), 1.31 (t, J = 7.0 Hz, 3H), 1.23 (s, 9H).

^{13}C NMR (126 MHz, CDCl$_3$) δ 162.07 (d, $J_{C\text{-}F}$ = 246.9 Hz), 159.2, 158.8, 152.9, 151.0, 150.9, 150.4, 148.6, 147.1, 145.0, 141.9, 139.3, 137.7, 130.5, 129.7, 129.4, 128.8, 128.7, 128.60 (d, $J_{C\text{-}F}$ = 15.4 Hz), 127.7, 127.3, 126.9, 125.7, 125.3, 125.09 (d, $J_{C\text{-}F}$ = 5.9 Hz), 123.82 (d, $J_{C\text{-}F}$ = 8.2 Hz), 122.1, 115.7, 115.6, 115.1, 114.7, 114.0, 113.6, 108.3, 108.1, 106.5, 99.6, 63.6, 55.3, 34.8, 31.3, 21.5, 14.9.

^{19}F NMR (471 MHz, CDCl$_3$) δ -114.6.

IR (KBr, cm^{-1}) 2946, 2856, 2211, 1740, 1664, 1571, 1479, 1047, 927, 786.

HRMS (ESI) ([M+Na]$^+$) Calcd. for [$C_{61}H_{50}BrFNaO_6$]$^+$: 999.2667, Found. 999.2660.

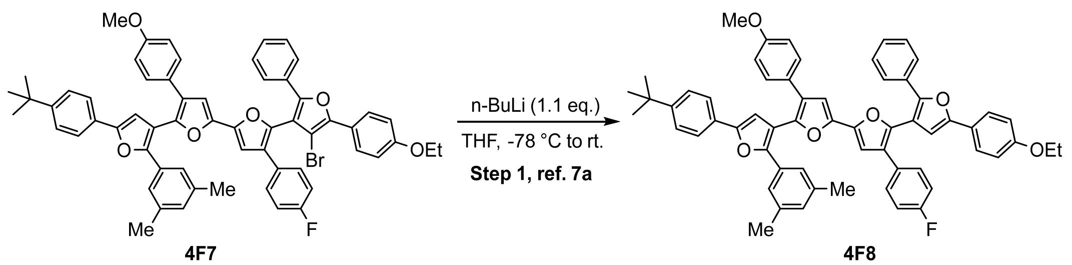

4F7 (59.7 mg, 0.06 mmol) was dissolved in THF (1 mL) and the resulting solution was cooled to –78 °C. A solution of n-BuLi (2.5 M in hexanes, 0.026 mL, 0.066 mmol, 1.1 mol equiv) was added dropwise, the resulting solution was stirred at –78 °C for 1 h then quenched with methanol and allowed to warm to RT. The solution was then diluted in diethyl ether (5 mL) and washed with water (2 × 5 mL). The organic phase was dried over $MgSO_4$ and solvent was removed under reduced pressure. The resulting residue was purified by flash column chromatography (silica gel, petroleum ether/AcOEt = 10:1) to yield **4F8** as a yellow solid (49.1 mg, 91%).

5-(4-(tert-butyl)phenyl)-2-(3,5-dimethylphenyl)-5'''-(4-ethoxyphenyl)-4''-(4-fluorophenyl)-3'-(4-methoxyphenyl)-2'''-phenyl-3,2':5',2'':5'',3'''-quaterfuran (4F8)

Yield: 49.1 mg, 91%, yellow solid, m.p. = 125 - 128 ℃, R_f = 0.33 (petroleum ether/AcOEt =10:1).

^{1}H NMR (500 MHz, $CDCl_3$) δ 7.69 – 7.60 (m, 6H), 7.45 – 7.41 (m, 2H), 7.38 – 7.29 (m, 6H), 7.28 – 7.23 (m, 2H), 7.22 – 7.16 (m, 1H), 6.96 – 6.88 (m, 4H), 6.86 (s, 1H), 6.84 (d, J = 2.6 Hz, 2H), 6.79 – 6.73 (m, 2H), 6.72 (s, 1H), 6.63 (s, 1H), 4.08 (q, J = 7.0 Hz, 2H), 3.76 (s, 3H), 2.25 (s, 6H), 1.44 (t, J = 7.0 Hz, 3H), 1.35 (s, 9H).

^{13}C NMR (126 MHz, $CDCl_3$) δ 161.95 (d, $J_{C\text{-}F}$ = 246.7 Hz), 158.9, 158.8, 153.2, 152.8, 150.9, 150.4, 149.9, 145.8, 145.1, 142.0, 141.7, 137.6, 130.50 (d, $J_{C\text{-}F}$ = 6.9 Hz), 130.1, 129.6, 129.04 (d, $J_{C\text{-}F}$ = 8.0 Hz), 128.86 (d, $J_{C\text{-}F}$ = 3.3 Hz), 128.6, 128.3, 127.8, 127.7, 125.7, 125.54, 125.45, 125.3, 125.0, 124.3, 123.8, 123.7, 123.1, 115.5, 115.3, 114.8, 113.9, 113.54, 113.47, 108.0, 107.7, 107.1, 106.9, 63.6, 55.3, 34.7, 31.3, 21.4, 14.8.

^{19}F NMR (471 MHz, $CDCl_3$) δ -115.1.

IR (KBr, cm^{-1}) 3017, 2829, 2514, 2291, 1781, 1571, 1479, 1247, 1017, 886.

HRMS (ESI) ([M+H]$^+$) Calcd. for $[C_{61}H_{52}FO_6]^+$: 899.3742, Found. 899.3754.

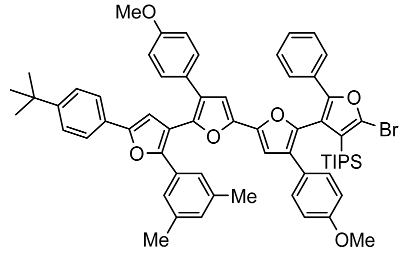

(5-bromo-5'''-(4-(tert-butyl)phenyl)-2'''-(3,5-dimethylphenyl)-3',4''-bis(4-methoxyphenyl)-2-phenyl-[3,2':5',2'':5'',3'''-quaterfuran]-4-yl)triisopropylsilane (4F9)

Yield: 178 mg, 87%, yellow solid, m.p. = 141 - 143 ℃, R_f = 0.33 (petroleum ether/AcOEt =20:1).

^{1}H NMR (500 MHz, $CDCl_3$) δ 7.68 (d, J = 8.1 Hz, 2H), 7.50 (s, 1H), 7.45 (t, J = 7.9 Hz, 3H), 7.40 (d, J = 7.6 Hz, 1H), 7.37 – 7.30 (m, 3H), 7.21 – 7.13 (m, 5H), 6.89 – 6.85 (m, 2H), 6.83 (s, 1H), 6.78 (d, J = 8.5 Hz, 2H), 6.73 (d, J = 4.6 Hz, 1H), 6.71 – 6.63 (m, 2H), 3.75 (s, 3H), 3.70 (s, 3H), 2.26 (s, 6H), 1.35 (s, 9H), 1.28 – 1.22 (m, 3H), 1.11 – 0.91 (m, 18H).

^{13}C NMR (126 MHz, CDCl$_3$) δ158.79, 158.75, 158.6, 158.4, 155.7, 153.7, 152.81, 152.77, 150.92, 150.89, 150.5, 150.4, 148.6, 145.7, 145.6, 145.4, 145.1, 142.2, 141.5, 141.4, 140.5, 137.6, 137.6, 130.5, 130.4, 129.6, 128.7, 128.64, 128.62, 128.5, 128.0, 127.9, 127.8, 125.7, 125.4, 125.33, 125.28, 125.2, 125.0, 124.97, 124.9, 123.8, 123.7, 118.7, 118.3, 118.2, 115.4, 114.0, 113.9, 113.8, 108.0, 107.98, 107.6, 107.3, 106.5, 106.3, 55.3, 55.2, 34.7, 31.3, 21.4, 18.8, 11.7.
IR (KBr, cm^{-1}) 2910, 2738, 2019, 1892, 1713, 1483, 1336, 1071, 910, 786.
HRMS (ESI) ([M+H]$^+$) Calcd. for [$C_{63}H_{66}BrO_6Si$]$^+$: 1025.3807, Found. 1025.3804.

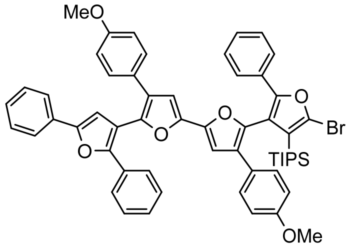

(5-bromo-3',4''-bis(4-methoxyphenyl)-2,2''',5'''-triphenyl-[3,2':5',2'':5'',3'''-quaterfuran]-4-yl)triisopropylsilane (4F11)
Yield: 160 mg, 85%, yellow solid, m.p. = 129 - 131 ℃, R$_f$ = 0.34 (petroleum ether/AcOEt =20:1).
^{1}H NMR (500 MHz, CDCl$_3$) δ 7.74 (d, *J* = 7.9 Hz, 2H), 7.71 – 7.66 (m, 2H), 7.44 – 7.38 (m, 4H), 7.35 (d, *J* = 8.7 Hz, 2H), 7.29 (t, *J* = 7.7 Hz, 3H), 7.26 – 7.20 (m, 4H), 7.20 – 7.10 (m, 2H), 6.85 (dd, *J* = 7.8, 1.9 Hz, 2H), 6.82 – 6.76 (m, 3H), 6.70 (d, *J* = 8.7 Hz, 2H), 3.75 (s, 3H), 3.71 (s, 3H), 1.30 – 1.19 (m, 3H), 1.08 (d, *J* = 7.3 Hz, 9H), 0.94 (d, *J* = 7.3 Hz, 9H).
^{13}C NMR (126 MHz, CDCl$_3$) δ 158.9, 158.6, 155.6, 152.8, 150.4, 145.6, 145.4, 141.2, 140.6, 130.5, 130.3, 129.4, 129.3, 128.8, 128.7, 128.3, 127.93, 127.90, 127.8, 126.1, 125.8, 125.3, 125.2, 124.9, 124.0, 118.8, 118.3, 114.0, 113.9, 108.7, 107.6, 106.6, 55.3, 55.2, 18.99, 18.95, 11.8.
IR (KBr, cm^{-1}) 2946, 2856, 2211, 1740, 1664, 1571, 1479, 1047, 927, 786.
HRMS (ESI) ([M+Na]$^+$) Calcd. for [$C_{57}H_{53}BrNaO_6Si$]$^+$: 963.2687, Found. 963.2617.

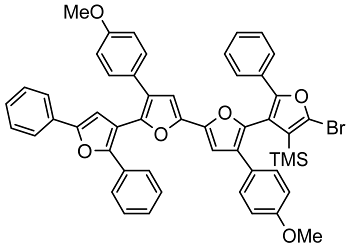

(5-bromo-3',4''-bis(4-methoxyphenyl)-2,2''',5'''-triphenyl-[3,2':5',2'':5'',3'''-quaterfuran]-4-yl)trimethylsilane (4F12)
Yield: 146 mg, 83%, yellow solid, m.p. = 151 - 154 ℃, R$_f$ = 0.33 (petroleum ether/AcOEt =15:1).
^{1}H NMR (500 MHz, CDCl$_3$) δ 7.73 (d, *J* = 7.7 Hz, 2H), 7.70 (d, *J* = 8.0 Hz, 2H), 7.46 – 7.38 (m, 4H), 7.35 (d, *J* = 8.5 Hz, 2H), 7.29 (q, *J* = 7.5 Hz, 3H), 7.24 – 7.16 (m, 6H), 6.87 (d, *J* = 6.0 Hz, 2H), 6.79 (s, 2H), 6.77 (d, *J* = 2.7 Hz, 2H), 6.75 (s,1H), 3.76 (s, 3H), 3.74 (s, 3H), 0.09 (s, 9H).
^{13}C NMR (126 MHz, CDCl$_3$) δ 159.9, 159.8, 155.7, 153.9, 151.4, 146.9, 146.4, 142.4, 141.6, 131.6, 131.3, 130.3, 129.8, 129.7, 129.6, 129.3, 129.2, 128.94, 128.91, 128.85, 128.7, 127.3, 126.9, 126.20, 126.17, 126.1, 125.8, 125.0, 123.3, 118.6, 115.1, 115.0, 114.9, 109.7, 108.7, 107.5, 56.3, 56.3, 0.0.
IR (KBr, cm^{-1}) 3028, 2938, 2863, 1713, 1451, 1236, 1133, 1023, 958, 894, 698, 579.
HRMS (ESI) ([M+Na]$^+$) Calcd. for [$C_{51}H_{41}BrNaO_6Si$]$^+$: 879.1748, Found. 879.1742.

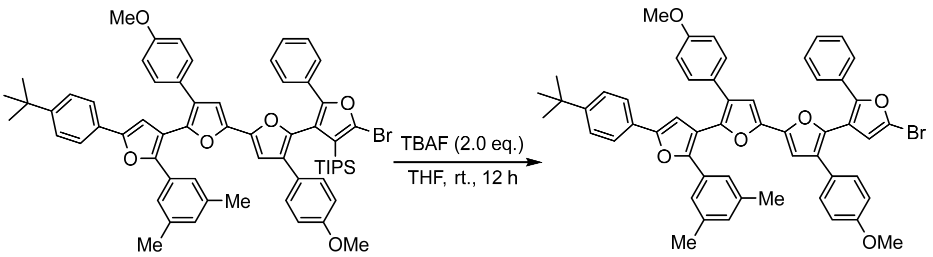

4F9 **4F10-Br**

A mixture of **4F9** (0.1 mmol) and TBAF (1.0 M in THF, 0.2mL, 0.2 mmol) was stirred in THF (4 mL) at rt. for 12 h. The reaction was quenched with sat. NH_4Cl (5 mL). The aqueous phase was extracted with AcOEt (3 × 20 mL). The combined organic layer was washed with brine (10 mL) and dried over MgSO4. The solvents were evaporated in vacuo. The obtained crude product was purified with silica gel column chromatography (petroleum ether/AcOEt = 10:1) to afford **4F10-Br** as yellow oil.

5-bromo-5'''-(4-(tert-butyl)phenyl)-2'''-(3,5-dimethylphenyl)-3',4''-bis(4-methoxyphenyl)-2-phenyl-3,2':5',2'':5'',3'''-quaterfuran (4F10-Br)

Yield: 55 mg, 63%, yellow oil, R_f = 0.31 (petroleum ether/AcOEt =20:1).

^{1}H NMR (500 MHz, $CDCl_3$) δ 7.59 (d, *J* = 7.4 Hz, 2H), 7.49 (d, *J* = 6.2 Hz, 2H), 7.36 (d, *J* = 8.1 Hz, 2H), 7.29 – 7.09 (m, 9H), 6.78 (s, 1H), 6.74 – 6.66 (m, 6H), 6.63 (s, 1H), 6.33 (s, 1H), 3.69 (s, 3H), 3.68 (s, 3H), 2.17 (s, 6H), 1.27 (s, 9H).

^{13}C NMR (126 MHz, $CDCl_3$) δ 158.9, 158.7, 153.0, 152.8, 150.9, 150.3, 145.7, 145.0, 141.7, 139.8, 137.6, 130.4, 129.7, 129.6, 128.7, 128.6, 128.29, 128.26, 127.6, 125.69, 125.67, 125.3, 125.0, 124.9, 123.8, 123.7, 121.9, 114.6, 114.2, 114.0, 113.9, 113.5, 108.0, 107.7, 107.3, 55.28, 55.27, 34.7, 31.3, 21.4.

IR (KBr, cm^{-1}) 3041, 2942, 2857, 1618, 1572, 1462, 1364, 1147, 958, 643.

HRMS (ESI) ($[M+H]^+$) Calcd. for $[C_{54}H_{46}BrO_6]^+$: 869.2472, Found. 869.2464.

10. General procedure for the oxidative coupling of bifurans.

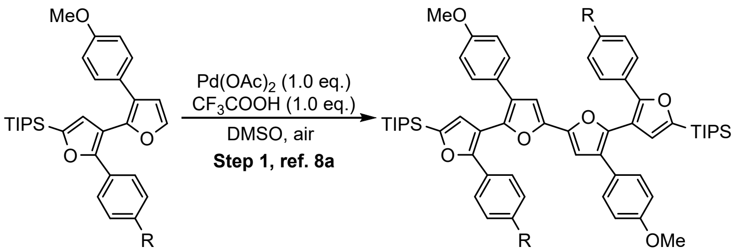

2F-Si **4F**

A seal tube containing $Pd(OAc)_2$ (1.0 eq.) was evacuated, then, DMSO (1.5 mL) and trifluoroacetic acid (TFA) (1.0 eq.) were sequentially added to the system via syringe. After that, bifurans **2F-Si** (0.2 mmol, 1.0 eq.) (Note: mixtures of α-Si-bifuran and β-Si-bifuran, the β-Si-bifuran did not react under this condition) were added and the reaction mixture was stirred at room temperature (sometimes at 80 °C) until completion of the reaction (TLC). Then water (5 mL) was added, and the resulting solution was extracted with dimethyl ether (15 mL × 4); The combined organic layer was washed with aqueous saturated brine solution, dried over $MgSO_4$, filtered and concentrated under reduced pressure. Purification by flash chromatography afforded the homocoupling product.

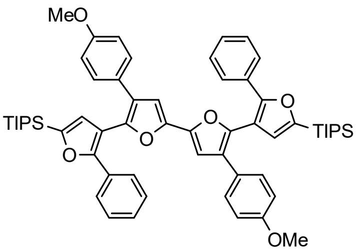

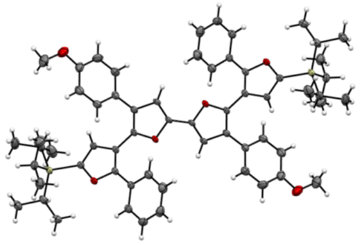

(3',4''-bis(4-methoxyphenyl)-2,2'''-diphenyl-[3,2':5',2'':5'',3'''-quaterfuran]-5,5'''-diyl)bis(triisopropylsilane) (4F13-Si$_2$)

Yield: 80 mg (α-Si-bifuran: β-Si-bifuran = 67:33), 63%, yellow solid, m.p. = 189 - 192 ℃, R_f = 0.33 (petroleum ether/AcOEt =10:1).

^{1}H NMR (500 MHz, CDCl$_3$) δ 7.66 (d, J = 7.8 Hz, 4H), 7.33 – 7.27 (m, 9H), 7.22 (t, J = 7.4 Hz, 3H), 6.82 (s, 2H), 6.76 (d, J = 8.4 Hz, 4H), 6.68 (s, 2H), 3.76 (s, 6H), 1.35 – 1.32 (m, 6H), 1.18 – 1.10 (m, 36H).

^{13}C NMR (101 MHz, CDCl$_3$) δ 158.6, 156.4, 155.0, 145.3, 141.8, 131.0, 128.6, 128.3, 127.7, 125.7, 125.3, 125.3, 124.6, 113.8, 111.8, 107.0, 55.3, 18.6, 11.0.

IR (KBr, cm^{-1}) 3018, 2942, 2726, 2364, 2019, 1901, 1823, 1682, 1246, 786.

HRMS (ESI) ([M+Na]$^+$) Calcd. for [$C_{60}H_{70}NaO_6Si_2$]$^+$: 965.4603, Found. 965.4607.

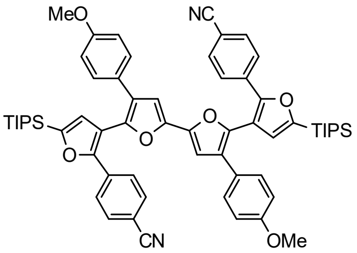

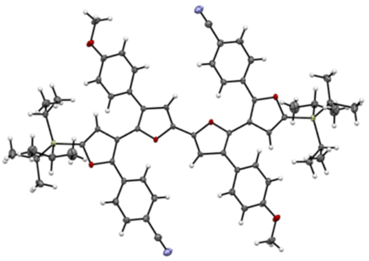

4,4'-(3',4''-bis(4-methoxyphenyl)-5,5'''-bis(triisopropylsilyl)-[3,2':5',2'':5'',3'''-quaterfuran]-2,2'''-diyl)dibenzonitrile (4F14-Si$_2$)

Yield: 95 mg (α-Si-bifuran: β-Si-bifuran = 92:8), 51%, yellow solid, m.p. = 191 - 193 ℃, R_f = 0.35 (petroleum ether/AcOEt =10:1).

^{1}H NMR (500 MHz, CDCl$_3$) δ 7.73 – 7.68 (m, 4H), 7.54 – 7.50 (m, 4H), 7.29 – 7.25 (m, 4H), 6.83 (s, 2H), 6.79 – 6.75 (m, 4H), 6.75 (s, 2H), 3.78 (s, 6H), 1.40 – 1.31 (m, 6H), 1.14 (d, 36H).

^{13}C NMR (126 MHz, CDCl$_3$) δ 159.0, 158.9, 152.3, 145.4, 141.0, 134.7, 132.1, 128.6, 125.8, 125.6, 125.4, 124.7, 118.9, 114.6, 114.0, 110.5, 107.7, 55.3, 18.6, 11.0.

IR (KBr, cm^{-1}) 3046, 2891, 2781, 2189, 1972, 1728, 1552, 946, 734.

HRMS (ESI) ([M+Na]$^+$) Calcd. for [$C_{62}H_{68}N_2NaO_6Si_2$]$^+$: 1015.4508, Found. 1015.4509.

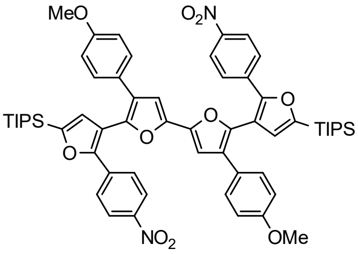

(3',4''-bis(4-methoxyphenyl)-2,2'''-bis(4-nitrophenyl)-[3,2':5',2'':5'',3'''-quaterfuran]-5,5'''-diyl)bis(triisopropylsilane) (4F15-Si$_2$)

Yield: 63 mg (α-Si-bifuran: β-Si-bifuran = 83:17), 36%, dark red oil, R_f = 0.35 (petroleum ether/AcOEt =10:1).

¹H NMR (500 MHz, CDCl₃) δ 8.11 (d, *J* = 8.6 Hz, 4H), 7.75 (d, *J* = 8.6 Hz, 4H), 7.28 (s, 2H), 7.27 (s, 2H), 6.86 (s, 2H), 6.79 – 6.76 (m, 4H), 6.76 (s, 2H), 3.76 (s, 6H), 1.39 – 1.32 (m, 6H), 1.17 – 1.11 (m, 36H).

^{13}C NMR (126 MHz, $CDCl_3$) δ 159.5, 159.0, 152.0, 146.4, 145.4, 140.9, 136.5, 128.7, 125.8, 125.8, 125.4, 124.7, 123.7, 115.3, 114.0, 107.8, 55.3, 18.6, 11.0.

IR (KBr, cm^{-1}) 3182, 2937, 2254, 1957, 1629, 1556, 1283, 1071, 910, 786.

HRMS (ESI) ($[M+Na]^+$) Calcd. for $[C_{60}H_{68}N_2NaO_{10}Si_2]^+$: 1055.4305, Found. 1055.4313.

10.1 General procedure for protodesilylation of 4F.

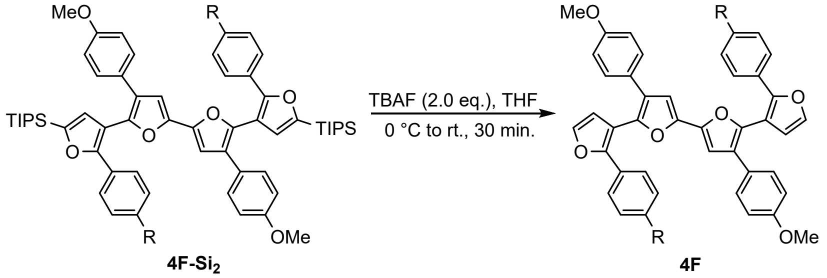

A mixture of **4F-Si₂** (0.2 mmol) and TBAF (1.0 M in THF, 0.4 mL, 0.4 mmol) was stirred in THF (4 mL) at 0 ℃. After 0.5 h of stirring at room temperature, the reaction was quenched with sat. NH_4Cl (5 mL). The aqueous phase was extracted with AcOEt (3 × 20 mL). The combined organic layer was washed with brine (10 mL) and dried over $MgSO_4$. The solvents were evaporated in vacuo. The obtained crude product was purified with silica gel column chromatography (petroleum ether/AcOEt = 20:1) to afford **4F** as yellow solid.

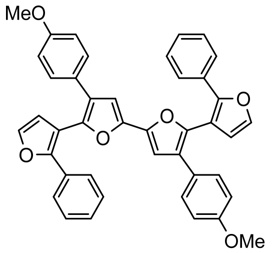

3',4''-bis(4-methoxyphenyl)-2,2'''-diphenyl-3,2':5',2'':5'',3'''-quaterfuran (4F13)

Yield: 77 mg, 59%, yellow solid, m.p. = 211 - 214 ℃, R_f = 0.33 (petroleum ether/AcOEt =20:1).

¹H NMR (500 MHz, $CDCl_3$) δ 7.63 – 7.60 (m, 4H), 7.48 (d, *J* = 1.9 Hz, 2H), 7.28 (dq, *J* = 8.9, 2.5, 1.8 Hz, 8H), 7.24 – 7.20 (m, 2H), 6.79 – 6.78 (m, 2H), 6.78 – 6.76 (m, 4H), 6.47 (d, *J* = 1.9 Hz, 2H), 3.77 (s, 6H).

^{13}C NMR (126 MHz, $CDCl_3$) δ 158.8, 151.2, 145.3, 141.7, 141.3, 130.6, 128.7, 128.3, 127.9, 125.8, 125.2, 125.0, 113.9, 113.5, 111.8, 107.4, 55.3.

IR (KBr, cm^{-1}) 3063, 2941, 1730, 1663, 1363, 1142, 1011, 920, 698.

HRMS (ESI) ($[M+Na]^+$) Calcd. for $[C_{42}H_{30}NaO_6]^+$: 653.1935, Found. 653.1929.

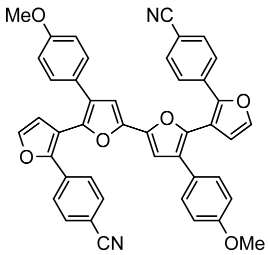

4,4'-(3',4''-bis(4-methoxyphenyl)-[3,2':5',2'':5'',3'''-quaterfuran]-2,2'''-diyl)dibenzonitrile (4F14)
Yield: 91 mg, 67%, yellow solid, m.p. = 262 - 264 ℃, R_f = 0.35 (petroleum ether/AcOEt =10:1).
^{1}H NMR (500 MHz, CDCl$_3$) δ 7.68 (d, *J* = 8.2 Hz, 4H), 7.56 (s, 2H), 7.50 (d, *J* = 8.2 Hz, 4H), 7.25 (s, 3H), 6.79 (s, 4H), 6.78 (s, 2H), 6.56 (d, 2H), 3.78 (s, 6H).
^{13}C NMR (126 MHz, CDCl$_3$) δ 159.1, 148.4, 145.4, 143.2, 140.6, 134.4, 132.0, 128.7, 125.93, 125.87, 124.6, 118.8, 114.6, 114.1, 113.9, 110.7, 108.0, 55.3.
IR (KBr, cm^{-1}) 3113, 2977, 2548, 1920, 1663, 1458, 1011, 745.
HRMS (ESI) ([M+H]$^+$) Calcd. for [$C_{44}H_{29}N_2O_6$]$^+$: 681.2020, Found. 681.2028.

11. Synthesis of 6F and 10F.

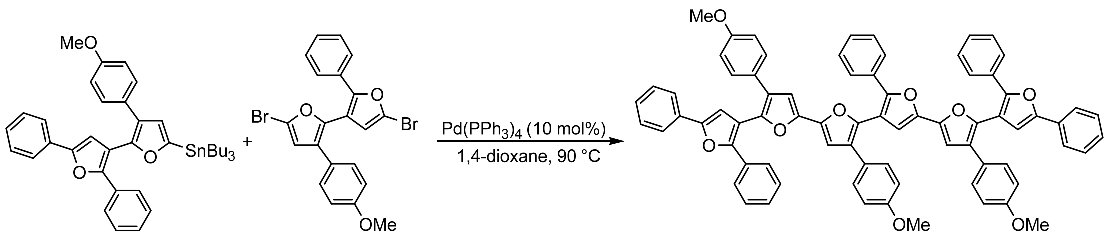

Synthesis of 6F: A mixture of **2F1-Sn** (0.2 mmol, 1.0 eq.), **2F38-Br$_2$** (0.42 mmol, 2.1 eq.), and Pd(PPh$_3$)$_4$ (0.02 mmol, 10 mol%) in dry dioxane (20 mL) was heated under nitrogen at 90 ℃ for 24 h. The solvent was evaporated under reduced pressure and the resulting residue was dissolved in ethyl acetate. This solution was passed through celite to remove Pd. The resulting residue was purified by flash column chromatography (silica gel, petroleum ether/AcOEt = 5:1) to yield **6F** (75%).
Sexifuran (6F):
Yield: 165 mg, 75%, yellow solid, m.p. = 182 - 184 ℃, R_f = 0.31 (petroleum ether/AcOEt =5:1).
^{1}H NMR (500 MHz, CDCl$_3$) δ 7.65 (t, *J* = 7.1 Hz, 4H), 7.62 – 7.59 (m, 4H), 7.58 (s, 1H), 7.36 – 7.29 (m, 6H), 7.27 (s, 1H), 7.25 (s, 2H), 7.24 – 7.19 (m, 7H), 7.18 (s, 4H), 7.17 – 7.12 (m, 3H), 6.90 (s, 1H), 6.77 – 6.72 (m, 4H), 6.72 – 6.68 (m, 4H), 6.67 (s, 1H), 6.57 (s, 1H), 3.71 (s, 3H), 3.68 (s, 3H), 3.67 (s, 3H).
^{13}C NMR (126 MHz, CDCl$_3$) δ 157.83, 157.80, 157.76, 151.7, 149.5, 149.45, 149.36, 144.5, 144.4, 144.1, 144.0, 140.5, 140.2, 139.7, 129.5, 129.4, 129.23, 129.21, 129.17, 127.7, 127.6, 127.3, 127.2, 127.0, 126.87, 126.85, 126.7, 124.8, 124.73, 124.69, 124.3, 124.14, 124.09, 124.0, 122.9, 113.0, 112.9, 112.8, 112.74, 112.67, 108.0, 107.7, 107.5, 107.0, 106.4, 54.25, 54.22.
IR (KBr, cm^{-1}) 3061, 2927, 2857, 1714, 1614, 1510, 1453, 1364, 1147, 958, 743.
HRMS (ESI) ([M+Na]$^+$) Calcd. for [$C_{75}H_{52}NaO_9$]$^+$: 1119.3504, Found. 1119.3504.

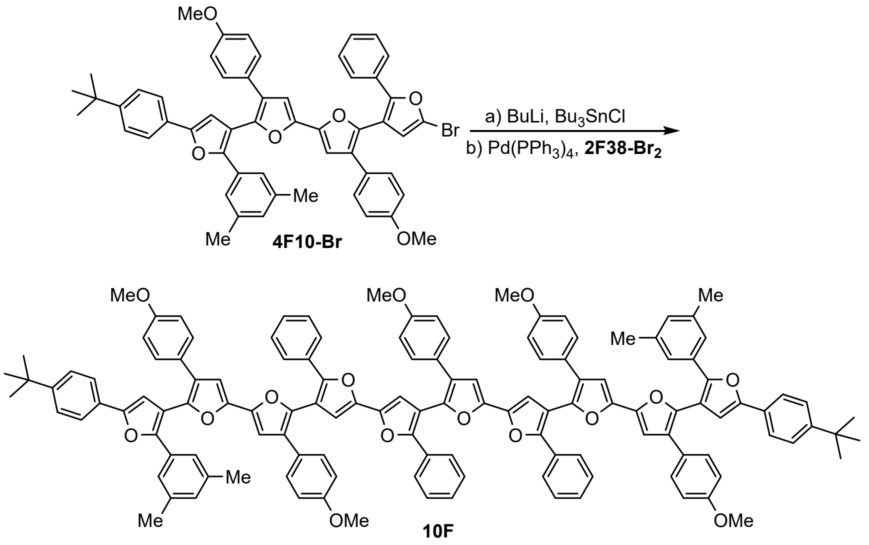

Synthesis of 10F: A 2.5 M solution of n-BuLi in hexane (0.04 mL, 0.11 mmol, 1.1 eq.) was added dropwise into a solution of **4F10-Br** (0.1 mmol) in dry THF (10 mL) at -78 °C under N_2. The reaction mixture was stirred for 20 min at -78 °C, $(n\text{-}Bu)_3SnCl$ (0.1 mmol, 1.0 eq.) was then added dropwise and the reaction mixture was allowed to reach room temperature and stirred for 2 h. The mixture was quenched with water, extracted with hexane, dried ($MgSO_4$), and evaporated. Repetitive flash chromatography on basified (NEt_3) silica using petroleum as eluent gave pure **4F-Sn** (76%) as light green oil.

A mixture of **4F-Sn** (0.1 mmol, 2.1 eq.), **2F38-Br₂** (0.05 mmol, 1.0 eq.), and $Pd(PPh_3)_4$ (0.005 mmol, 10 mol%) in dry dioxane (10 mL) was heated under nitrogen at 90 ℃ for 12 h. The solvent was evaporated under reduced pressure and the resulting residue was dissolved in ethyl acetate. This solution was passed through celite to remove Pd. The resulting residue was purified by flash column chromatography (silica gel, petroleum ether/AcOEt = 3:1) to yield **10F** (40% for two steps).

Decafuran (10F):

Yield: 50 mg, 40% for two steps, yellow oil, R_f = 0.31 (petroleum ether/AcOEt =3:1).

^{1}H NMR (500 MHz, $CDCl_3$) δ 7.69 – 7.64 (m, 6H), 7.62 (d, *J* = 7.6 Hz, 4H), 7.44 (d, *J* = 8.0 Hz, 4H), 7.39 (d, *J* = 8.4 Hz, 2H), 7.32 (ddd, *J* = 10.6, 7.9, 4.6 Hz, 13H), 7.28 (d, *J* = 7.9 Hz, 3H), 7.25 – 7.21 (m, 4H), 7.21 – 7.18 (m, 1H), 6.99 (s, 1H), 6.86 (d, *J* = 3.0 Hz, 3H), 6.84 (s, 2H), 6.83 (d, *J* = 2.1 Hz, 2H), 6.81 (s, 3H), 6.80 – 6.77 (m, 6H), 6.76 (d, *J* = 1.8 Hz, 2H), 6.72 (d, *J* = 3.2 Hz, 2H), 6.67 (s, 1H), 3.79 (s, 3H), 3.77 – 3.74 (m, 12H), 2.26 (d, *J* = 3.1 Hz, 12H), 1.35 (s, 18H).

^{13}C NMR (126 MHz, $CDCl_3$) δ 160.7, 160.4, 158.9, 158.79, 158.70, 158.7, 152.71, 152.70, 150.8, 150.7, 150.5, 150.3, 145.7, 145.6, 145.24, 145.17, 145.16, 145.0, 144.85, 144.81, 141.61, 141.59, 141.0, 140.6, 140.5, 137.6, 130.4, 130.2, 130.03, 130.01, 129.5, 128.7, 128.6, 128.5, 128.3, 128.28, 128.1, 128.06, 128.0, 127.6, 125.8, 125.72, 125.69, 125.66, 125.42, 125.36, 125.3, 125.02, 125.0, 124.97, 124.94, 124.93, 123.8, 123.6, 114.04, 113.99, 113.98, 113.87, 113.7, 113.6, 113.5, 109.6, 109.4, 109.1, 108.04, 107.98, 107.5, 107.3, 107.2, 55.3, 55.2, 34.7, 31.3, 21.4.

IR (KBr, cm^{-1}) 3623, 3049, 2923, 2856, 2304, 1927, 1708, 1492, 1301, 1224, 1049, 891, 744, 522.

HRMS (ESI) ($[M+Na]^+$) Calcd. for $[C_{129}H_{104}NaO_{15}]^+$: 1915.7267, Found. 1915.4175.

II. Supplementary Discussion

1. control experiments and proposed reaction pathway.

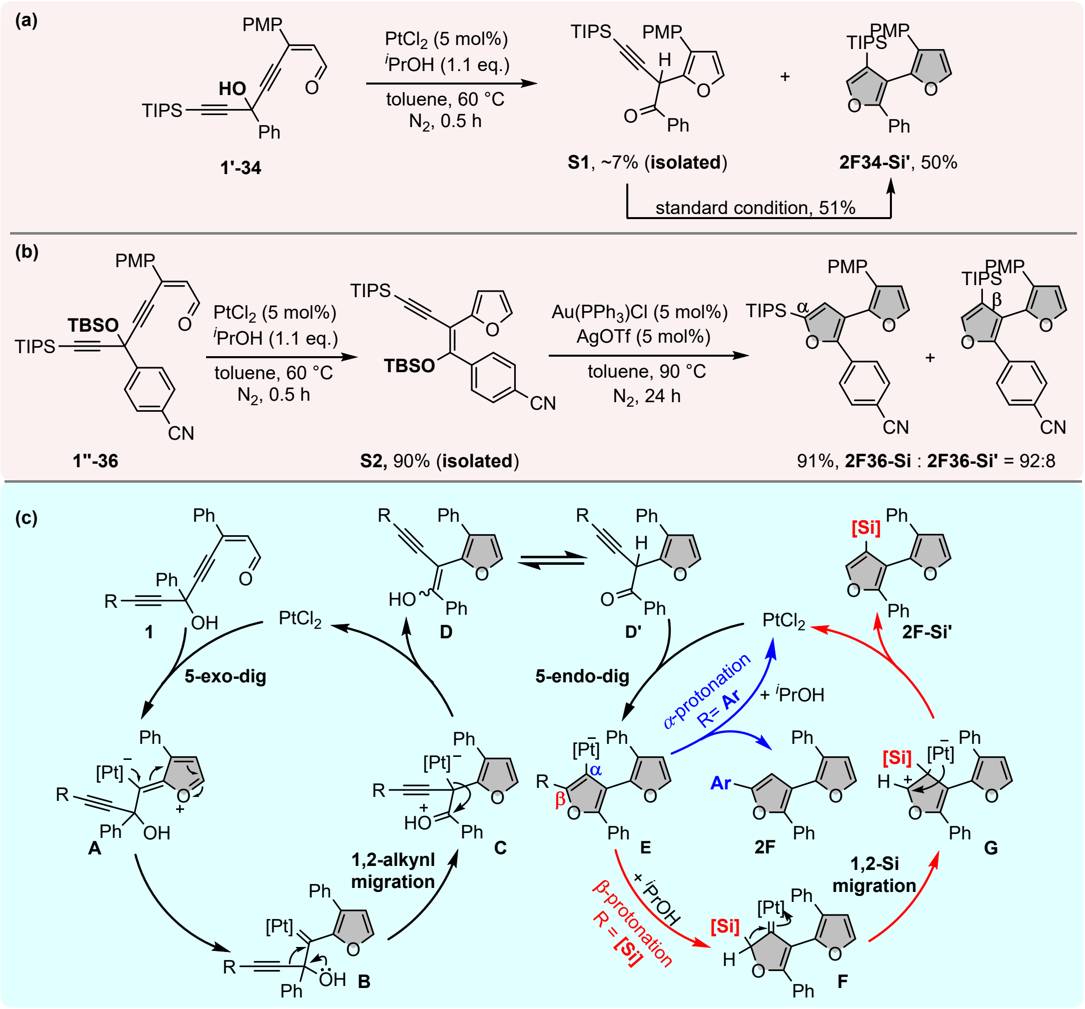

Supplementary Figure 3. Preliminary mechanism studies and possible mechanism. a, b control experiments. **c** proposed reaction pathway.

To investigate the reaction mechanism, several control reactions were then performed (supplementary Figs. 3a and 3b). Firstly, when enynal **1'-34** possessing a bulkier triisopropylsilyl terminus was subjected to the standard conditions in a shorter reaction time, the propargyl ketone intermediate **S1** could be isolated in 7% yield. The ketone **S1** could be further converted to **2F-Si'** in 51% yield under standard conditions (supplementary Fig. 3a), which indicated that propargyl ketone **S1** might be the key intermediate for this cascade reaction. Furthermore, when TBS-protected enynal **1''-36** was employed in the reaction, the silyl enol ether products S2 of alkynyl migration could be successfully obtained in 90% yield. The silyl enol ether **S2** could be efficiently transformed into the desired bifuran products **2F36-Si** and **2F36-Si'** under the catalysis of the gold catalyst, with 2-silylfuran isomer dominated in this process (supplementary Fig. 3b).

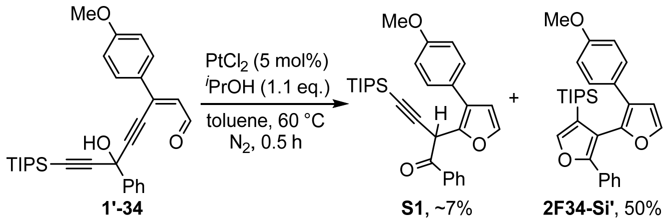

Under nitrogen atmosphere, to a solution of enynal **1'-34** (0.1 mmol) in dry toluene (0.025 M), $PtCl_2$(0.05 eq, 1.3 mg) and ^{i}PrOH (1.1 eq, 6.6 mg) were added. The reaction mixture was then heated to a temperature of 60 °C and stirred for 30 min. After the reaction was completed, the reaction mixture was filtered through short silica gel, and then the solvent was removed under reduced pressure. The propargyl ketone intermediate **S1** was purified by flash column chromatography (silica gel, petroleum ether/AcOEt = 20:1).

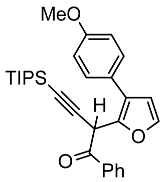

2-(3-(4-methoxyphenyl)furan-2-yl)-1-phenyl-4-(triisopropylsilyl)but-3-yn-1-one (S1)

Yield: 3 mg, 7%, yellow oil, R_f = 0.31 (petroleum ether/AcOEt =20:1).

^{1}H NMR (500 MHz, $CDCl_3$) δ 7.45 (t, J = 1.6 Hz, 1H), 7.36 – 7.31 (m, 4H), 7.29 – 7.24 (m, 3H), 6.94 – 6.90 (m, 2H), 6.51 (t, J = 1.7 Hz, 1H), 5.25 (d, J = 2.4 Hz, 1H), 3.81 (s, 3H), 1.03 – 0.94 (m, 21H).

^{13}C NMR (126 MHz, $CDCl_3$) δ 182.9, 158.9, 145.0, 142.4, 135.8, 129.5, 129.3, 128.7, 127.7, 125.6, 124.7, 114.2, 112.0, 103.6, 98.8, 77.3, 77.1, 76.8, 58.4, 55.3, 18.4, 10.9.

IR (KBr, cm^{-1}) 3108, 2927, 2453, 2208, 1691, 1435, 1004, 915.

HRMS (ESI) ($[M+H]^+$) Calcd. for $[C_{30}H_{37}O_3Si]^+$: 473.2506, Found. 473.2502.

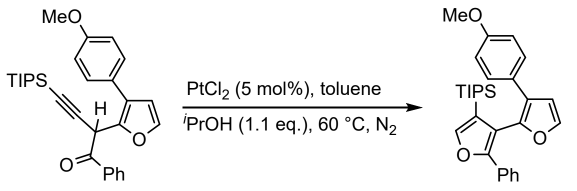

Under nitrogen atmosphere, to a solution of propargyl ketone intermediate **S1** (0.05 mmol) in dry toluene (0.025 M), $PtCl_2$ (0.05 eq, 1.0 mg) and ^{i}PrOH (1.1 eq, 3.3 mg) were added. The reaction mixture was then heated to a temperature of 60 °C and stirred for 48 h. After the reaction was completed, the reaction mixture was filtered through short silica gel, and then the solvent was removed under reduced pressure. The bifuran **2F34-Si'** (12 mg, 51%) was purified by flash column chromatography (silica gel, petroleum ether/AcOEt = 20:1).

Based on preliminary mechanistic observations, a plausible reaction mechanism is proposed in supplementary Fig. 3c. First, the alkyne moiety of **1** is activated by the platinum catalyst, which is then attacked by the carbonyl oxygen through 5-exo-dig cyclization to form zwitterionic intermediate or its resonance structure, (2-furyl)carbene complex **B**. As we propose an unprecedented 1,2-alkyne shift onto the platinum carbene to form intermediate **C**,[9,10] followed by subsequent protodeplatination to

intermediate **D'**. Then platinum activate the carbon-carbon triple bond of propargyl ketone **D'** again, which subsequently undergoes 5-endo-dig cyclization with the ketone oxygen atom to afford furyl-Pt species **E**, followed by its β-protonation at the vinyl-platinum moiety, would generate the Pt-carbene **F** when R= [Si], in which the positive charge at the carbene carbon is stabilized by the silicon atom,[11] a subsequent facile 1,2-Si over H migration in **G** would furnish 3-silyl bifuran **2F-Si'**.[12] In the case of R = Ar, a 1,2-H shift in **F** or direct a protodemetalation (α-protonation) of the vinyl-Pt moiety **E** afford the arylated bifurans **2F**. Finally, the metal is protonated and the catalyst is regenerated.

2. Photophysical data and spectra of oligo(arylfuran)s in DCM.

Table 3. Photophysical data of oligo(arylfuran)s in DCM.

Compound	λ_{abs}[a] (nm)	λ_{em}[b] (nm)	$\Delta\lambda$[c] (nm)
**2F1**	263, 286	405	119
**4F1**	286, 353	430	77
**4F2**	282	474	192
**4F13**	271, 350	437	87
**4F13-Si2**	265, 324	469	145
**4F14**	318, 403	519	116
**4F14-Si2**	313, 401	530	129
**6F**	276	474	198
**10F**	332	476	144

[a]Absorption. [b]Emission. [c]$\Delta\lambda = \lambda em - \lambda abs$. Concentration: 10 μM; λex= 365 nm.

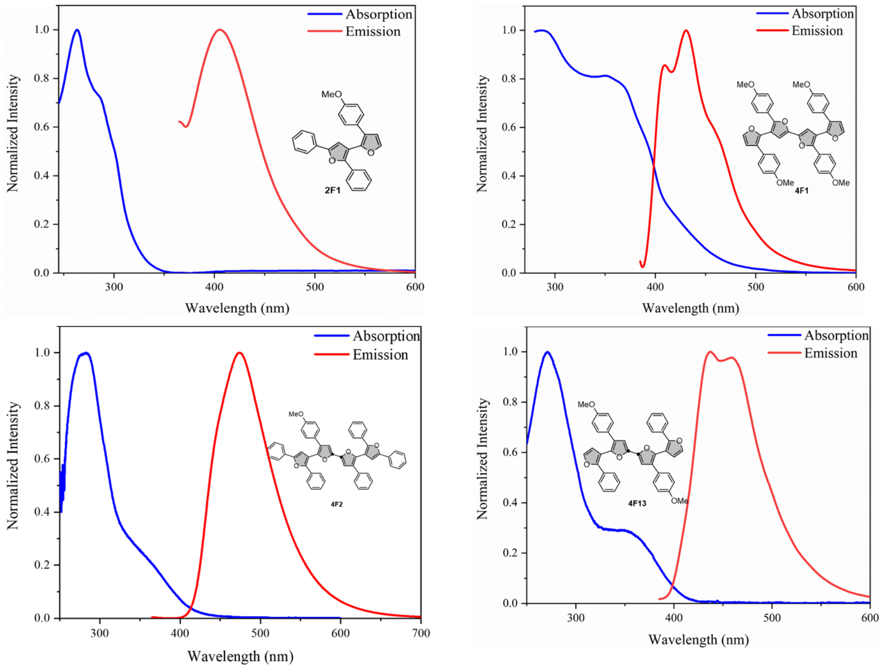

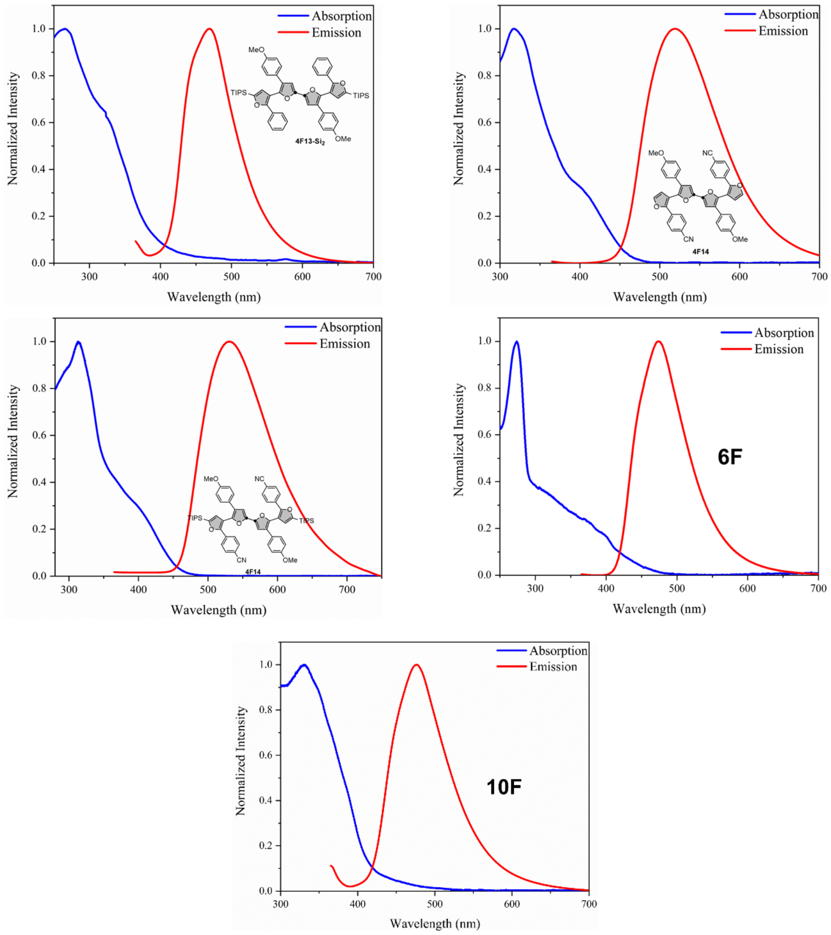

Supplementary Figure 4. Absorption spectra and emission spectra of representative oligo(arylfuran)s in DCM. (λex = 365 nm, Concentration: 10 μM).

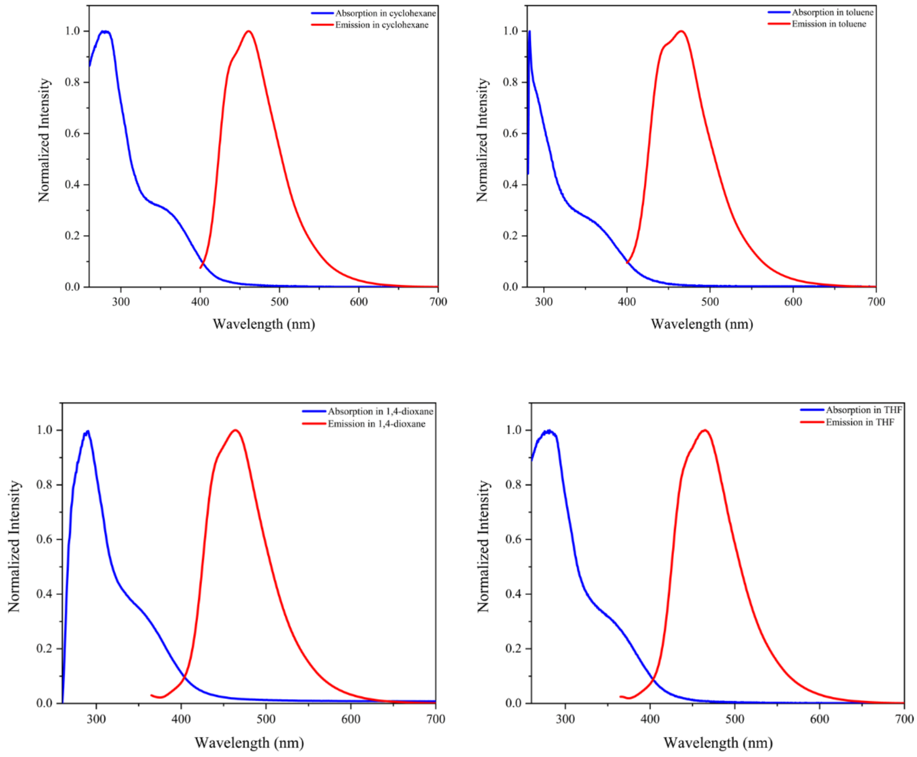

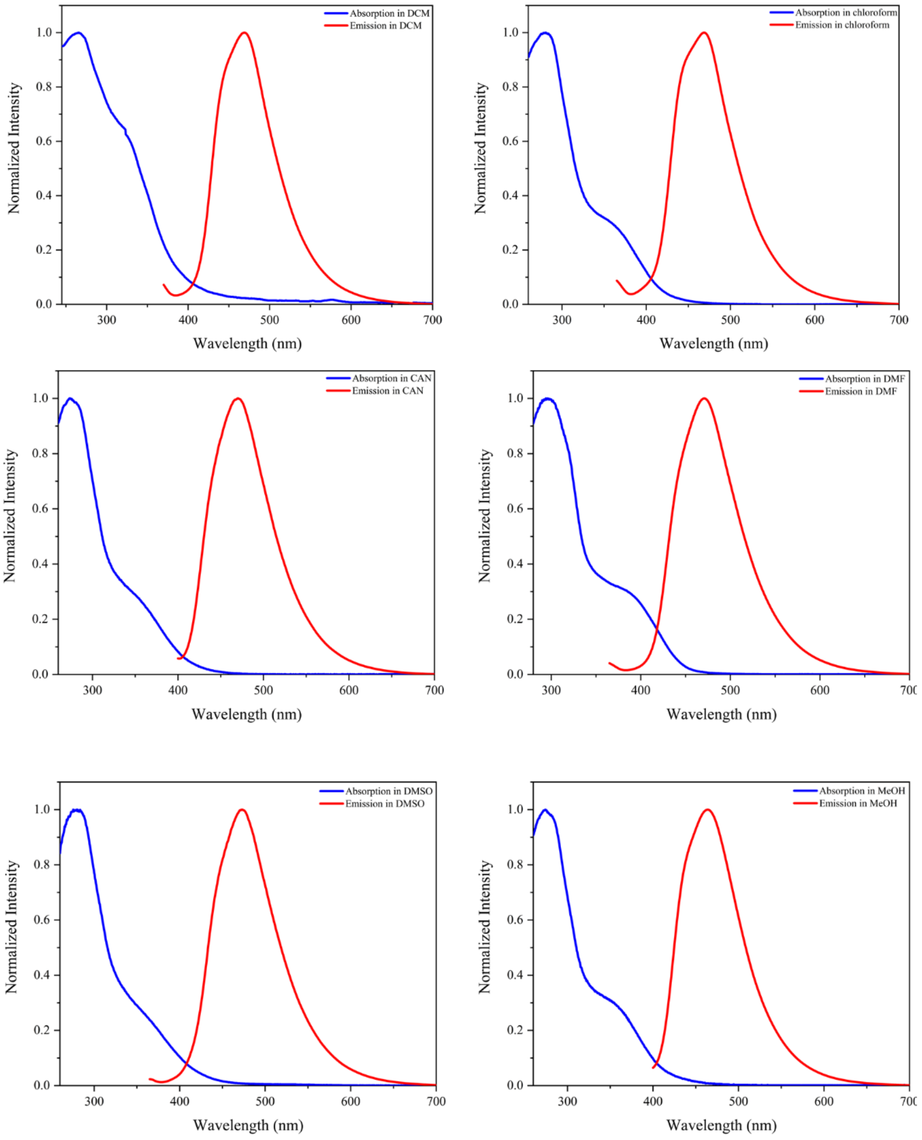

Supplementary Figure 5. Absorption and emission spectra of **4F13-Si$_2$** in different solvents (λex =365 nm, 10 μM).

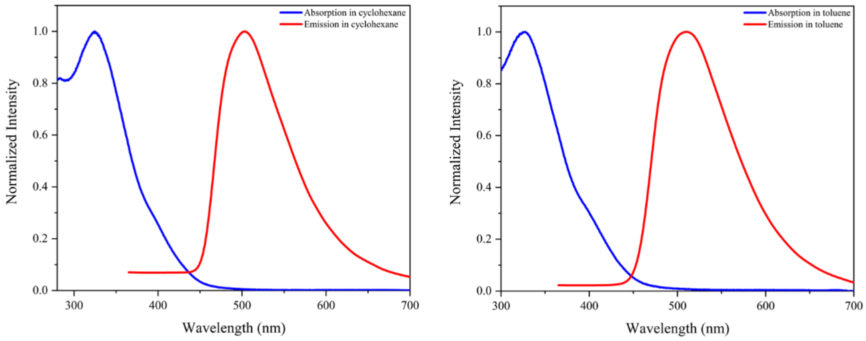

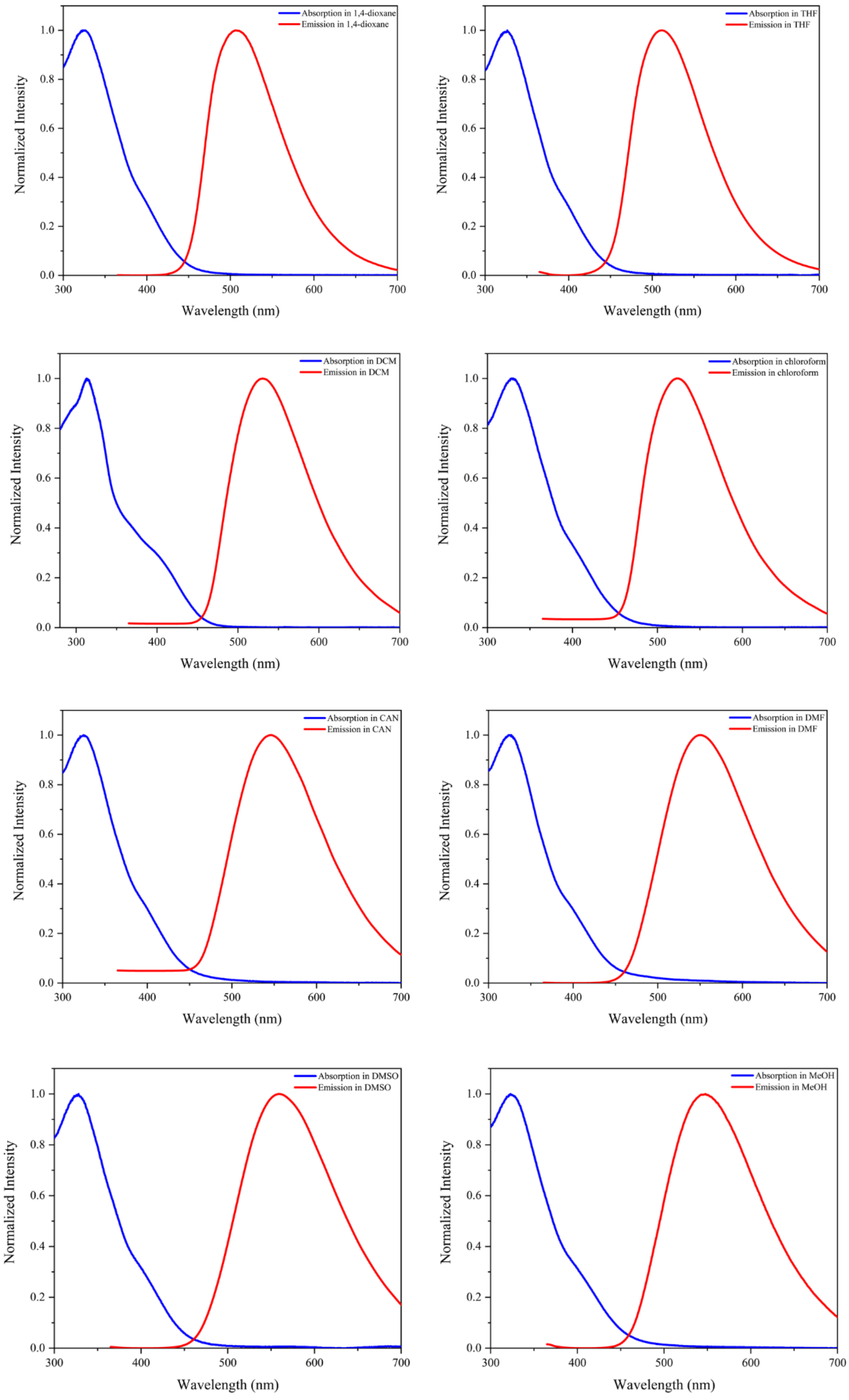

Supplementary Figure 6. Absorption and emission spectra of **4F14-Si$_2$** in different solvents (λex =365 nm, 10 μM).

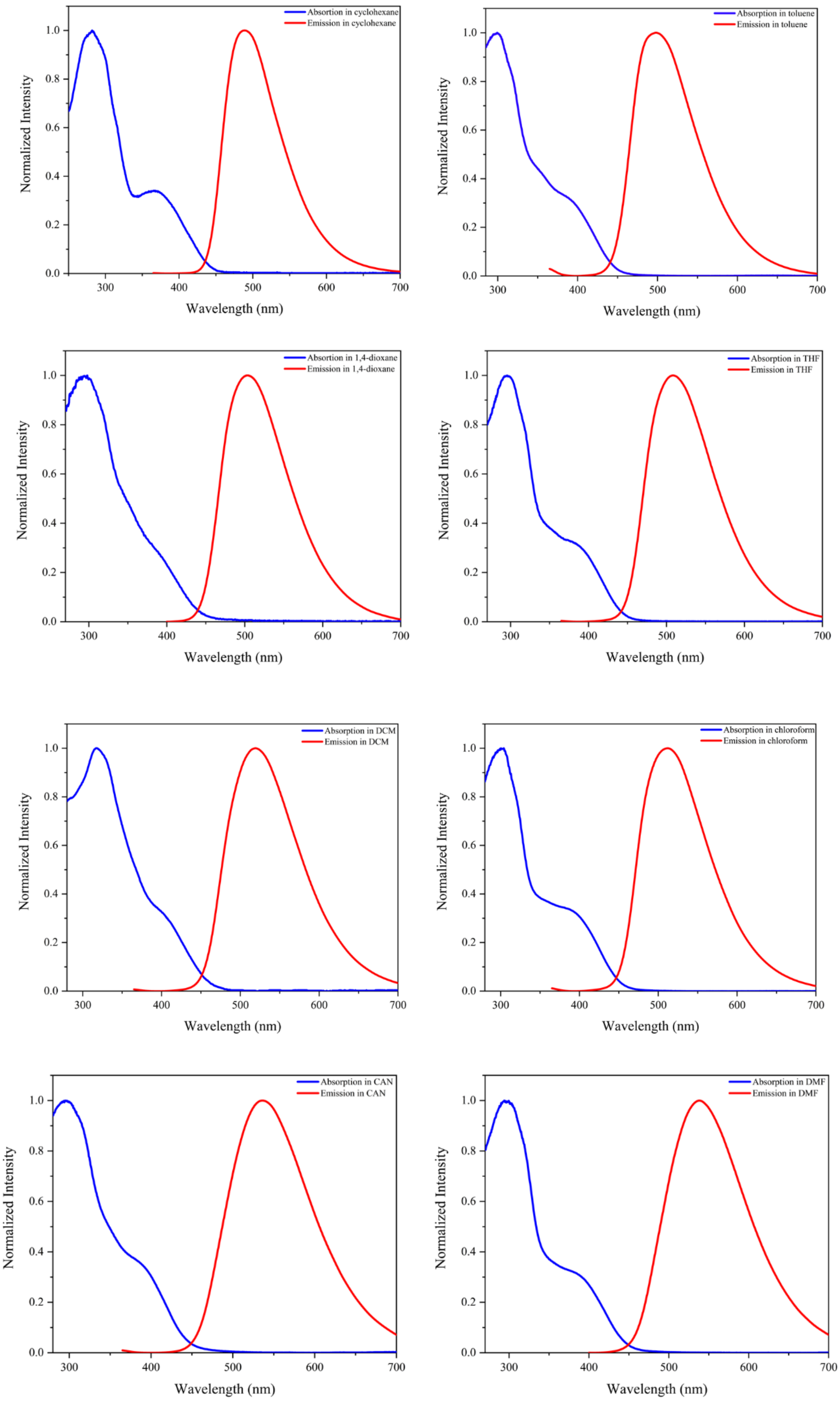
Absortion in cyclohexane
Emission in cyclohexane
Absorption in toluene
Emission in toluene
Absortion in 1,4-dioxane
Emission in 1,4-dioxane
Absorption in THF
Emission in THF
Absorption in DCM
Emission in DCM
Absorption in chloroform
Emission in chloroform
Absorption in CAN
Emission in CAN
Absorption in DMF
Emission in DMF
Normalized Intensity
Wavelength (nm)

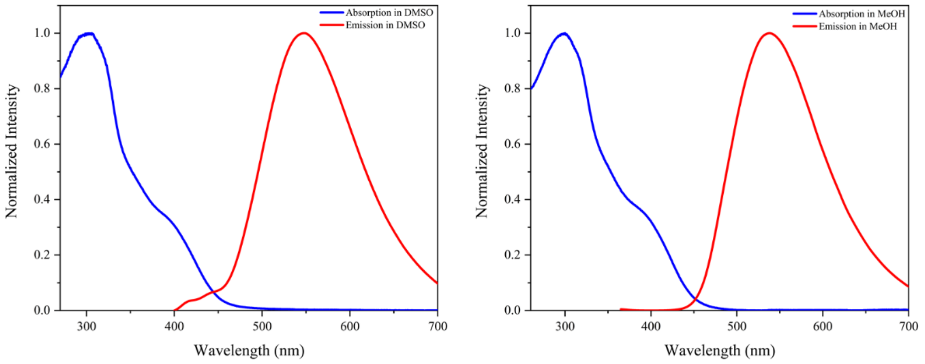

Supplementary Figure 7. Absorption and emission spectra of **4F14** in different solvents (λex =365 nm, 10 μM).

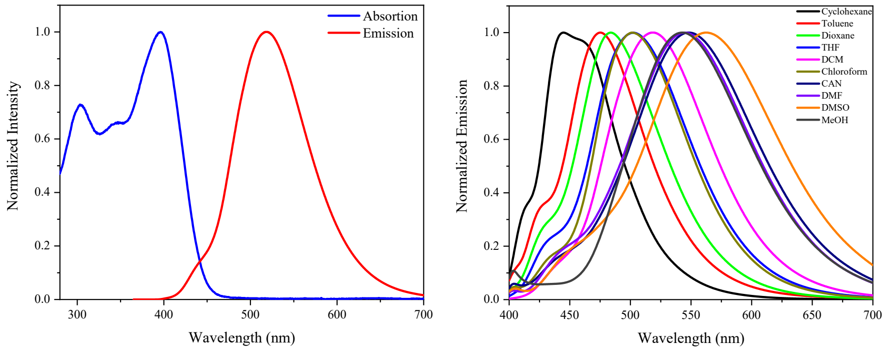

Supplementary Figure 8. left: Absorption spectra of **2F25** in DCM (10 μM). right: Emission spectra of **2F25** in different solvents.[a] λex =365 nm, Concentration:10 μM

3. Fluorescence spectra of 4F14 and 4F14-Si$_2$ in the solid-state (powder)[a]

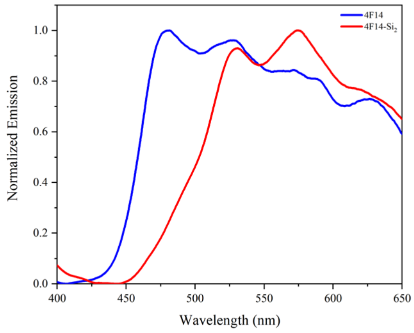

Supplementary Figure 9. Emission spectra of **4F14** and **4F14-Si$_2$** in the solid-state (powder). [a]λex =365 nm.

4. Solvatochromism of oligofurans[a]

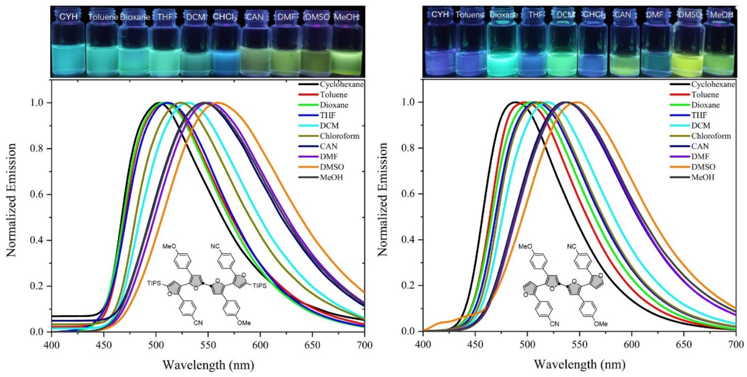

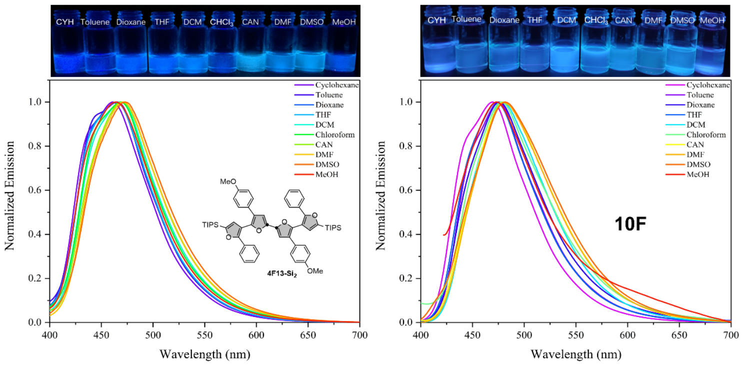

Supplementary Figure 10. Emission spectra of oligofurans in different solvents.$^{a}\lambda$ex =365 nm, Concentration:10 μM.

Supplementary Table 4. fluorescence quantum yields of 2F2-4F14.

	Φ_F[a] (%)			
**Compound**	THF[b]	THF[c]	Film[d]	Powder
**2F2**	17.8	19.7	5.5	14.6
**2F25**	80.8	90.8	3.0	3.5
**4F5**	12.0	13.0	5.7	1.0
**4F7**	9.0	10.0	1.9	0.8
**4F13-Si2**	54.5	60.8	3.2	6.1
**4F14-Si2**	56.3	68.0	7.5	11.5
**4F13**	68.3	77.9	6.4	38.6
**4F14**	55.0	64.5	1.1	1.3

[a]Absolute fluorescence quantum yield determined using a calibrated integrating sphere. [b]Measured in THF (10^{-5} M) under air. [c]Measured in THF (10^{-5} M) under N_2. [d]Film drop-casted on a quartz plate.

5. Excitation spectra of oligofurans

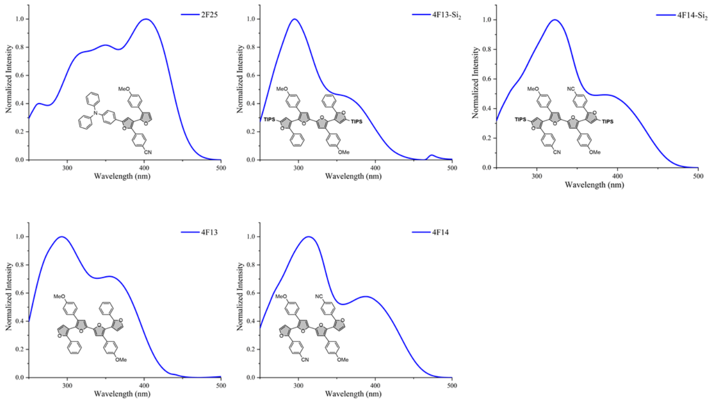

Supplementary Figure 11. Excitation spectra of representative oligo(arylfuran)s in DCM (Concentration: 10 μM) (emission wavelength are 517 nm, 469 nm, 530 nm, 437 nm and 519 nm, respectively.)

The excitation spectra of five compounds (**2F25**, **4F13-Si₂**, **4F14-Si₂**, **4F13** and **4F14**) have been measured in DCM. As shown in scheme 1, **2F25** had an excitation peak at 400 nm in the excitation spectrum. Broad peaks in the region 280-370 nm in the excitation spectra of **2F25** relates to the triphenylamine (TPA) structure. The other four tetrafurans (**4F13-Si₂**, **4F14-Si₂**, **4F13** and **4F14**) all have two excitation peaks at around 280-400 nm in the excitation spectrum, which are coincident with their UV absorptions shown in Supplementary Figure 4. Meanwhile, the excitation peaks of the push-pull oligofurans **4F14-Si₂** and **4F14** have significant red shifts relative to oligofurans **4F13-Si₂** and **4F13**, respectively.

6. Thermal and optical stability of oligo(arylfuran)s.

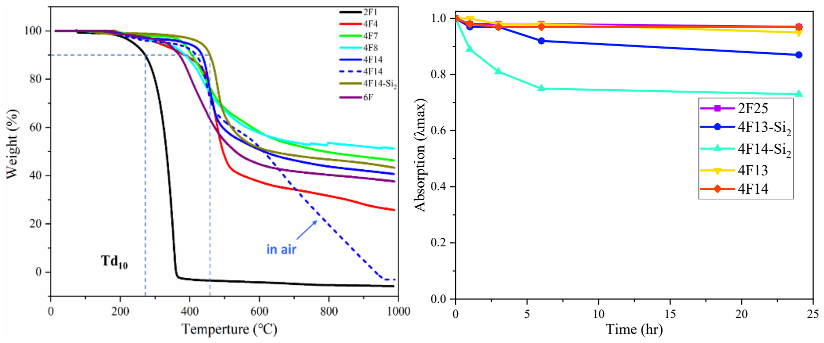

Supplementary Figure 12. (left) Thermogravimetric analysis (TGA) of **2F1** - **6F** under N_2, Note: T_{d10} = 10% weight-loss temperature, rate: 25 °C/min. (right) Photostability of solutions of **2F25**, **4F13-Si₂**, **4F14-Si₂**, **4F13** and **4F14** in DMSO in ambient light at room temperature.

Supplementary Table 5. Thermogravimetric data of 2F1 - 6F.

Entry	Compounds	Td_{10}
1	**2F1**	267 ℃
2	**4F4**	385 ℃
3	**4F7**	386 ℃
4	**4F8**	405 ℃
5	**4F14**	434 ℃
6	**4F14** (in air)	414 ℃
7	**4F14-Si₂**	457 ℃
8	**6F**	369 ℃

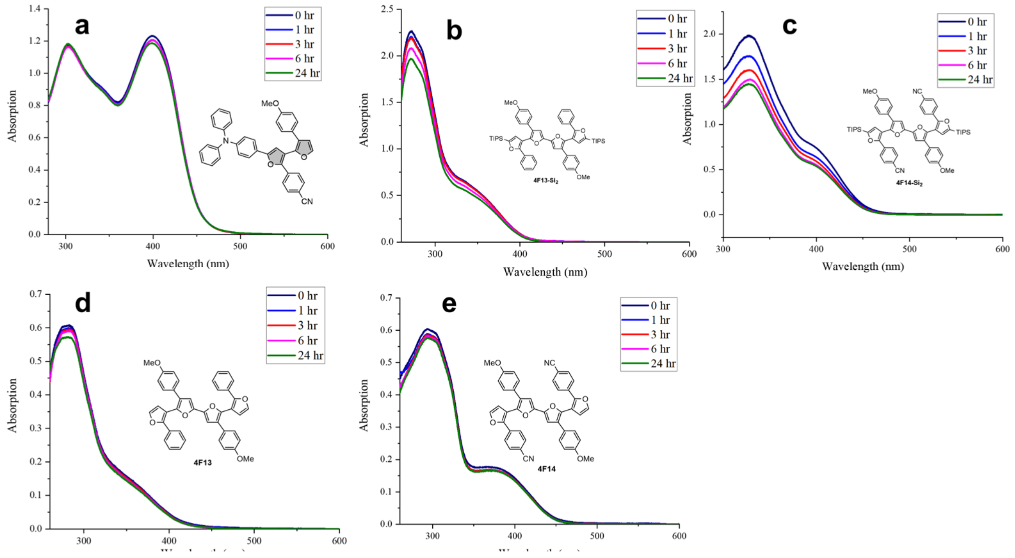

Supplementary Figure 13. Photostability at normal light in DMSO at room temperature a) **2F25**; b) **4F13-Si$_2$**; c) **4F14-Si$_2$**; d) **4F13**; e) **4F14**.

7. DFT and TD-DFT calculation Methods

Theoretical Calculation about oligofuran derivatives in the isolated state were carried out with the Gaussian 16 program[13] basing on density functional theory (DFT) and time-dependent density functional theory (TD-DFT), respectively. The hybrid functional B3LYP[14-16] and 6-311G(d,p) basis set[17] were applied for geometry optimization both in ground state and excited state. Energy level of frontier molecular orbitals are obtained by calculating the single-point energy basing on optimized S_0 geometries with functional B3LYP with def2-TZVP basis set.[18] Absorption and fluorescent spectra are obtained by calculating the vertical excitation energy based on the optimized geometries of S_0 and S_1 respectively with functional CAM-B3LYP[19] and def2-TZVP basis set. Absorption spectral analysis and natural transition orbitals (NTOs) analysis was performed with Multiwfn.[20]

8. DFT calculation

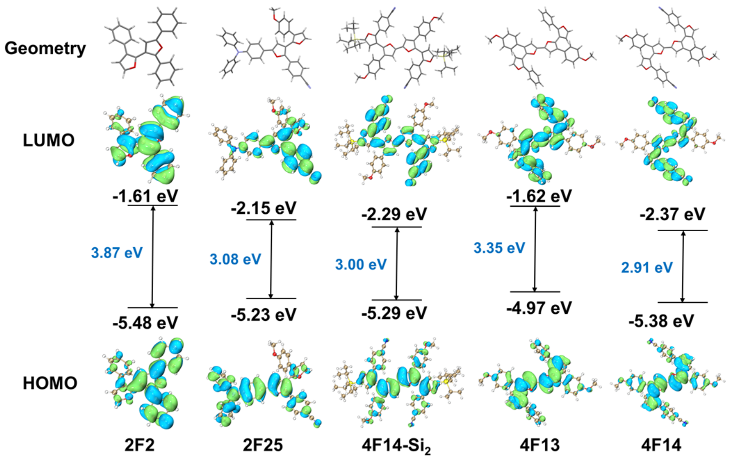

Supplementary Figure 14. Calculated molecular orbital amplitude plots and energy levels of HOMOs and LUMOs of **2F2**, **2F25**, **4F14-Si$_2$**, **4F13** and **4F14**.

9. TD-DFT calculation

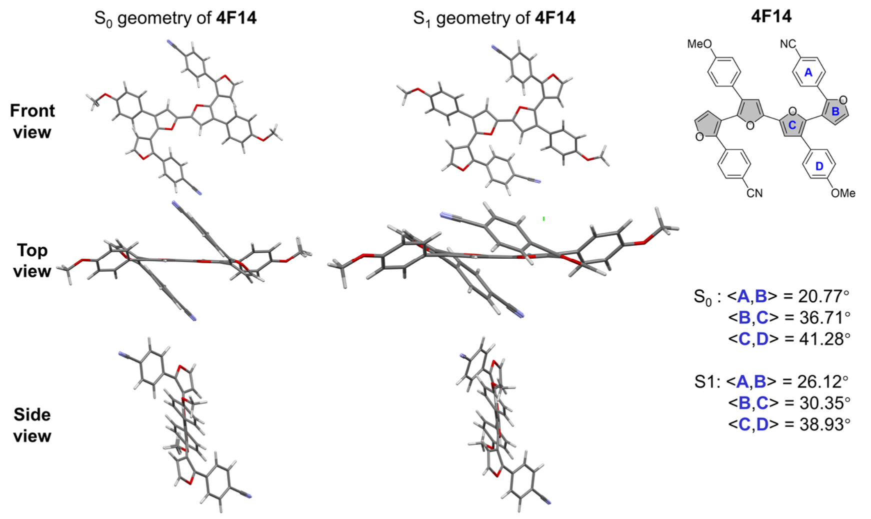

Supplementary Figure 15. Molecular geometries of **4F14** in S_0 and S_1 states.

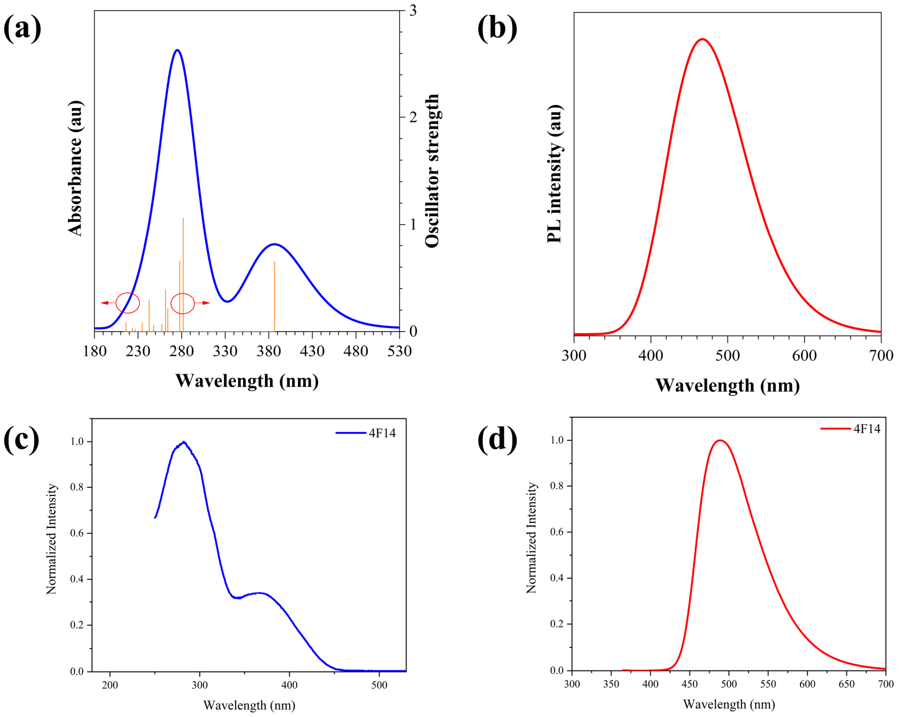

Supplementary Figure 16. Theoretical (gas-phase) and experimental (tested in cyclohexane solution) spectra of **4F14**. a) Theoretical and c) experimental absorption spectra. b) Theoretical and d) experimental fluorescence spectra.

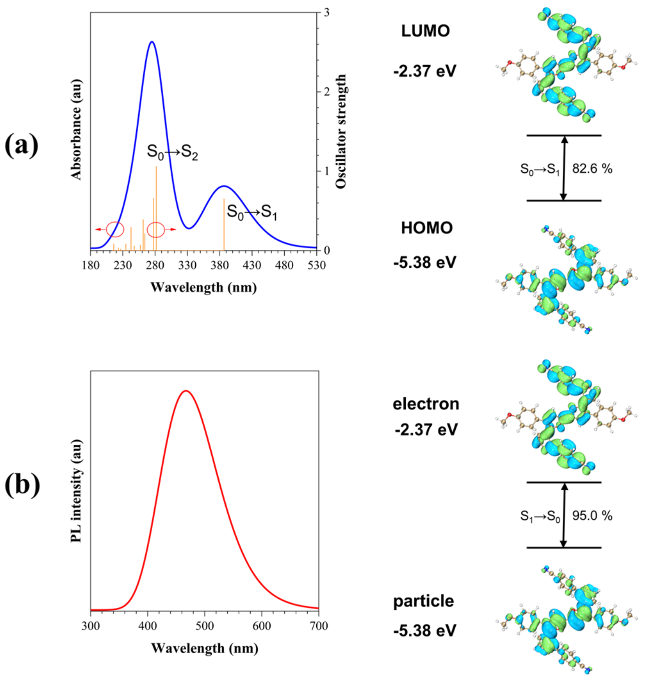

Supplementary Figure 17. a) Calculated absorption and frontier molecule orbitals dominating the $S_0 \rightarrow S_1$ transition. b) Calculated fluorescence spectrum and the major natural transition orbitals of **4F14**.

10. Electrochemistry properties of 2F22-4F14.

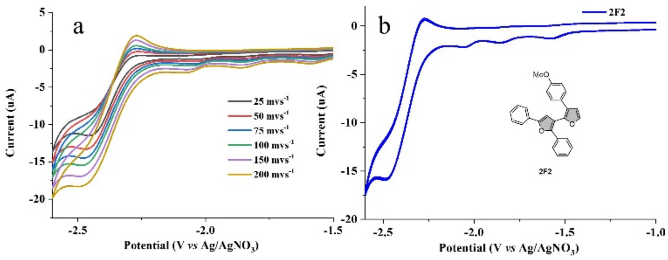

Supplementary Figure 18. (a) CV of **2F2** in DMF with 0.1 M *n*-Bu_4NClO_4 at different scan speeds. (b) repetitive CV scans of **2F2** in DMF with 0.1 M *n*-Bu_4NClO_4 (scan rate 100 mV/s). Fc/Fc^+ = 0.40 V vs SCE under these conditions.

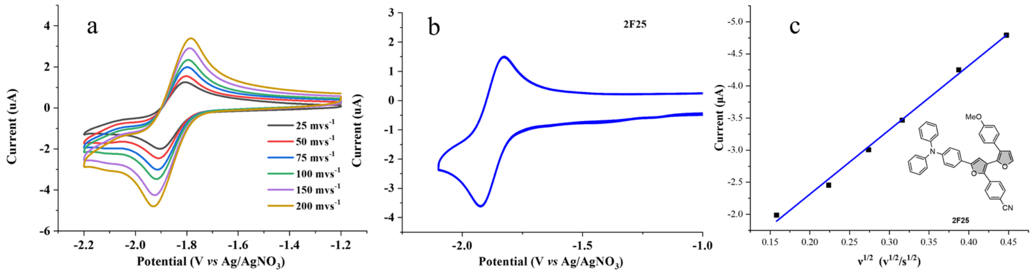

Supplementary Figure 19. (a) CV of **2F25** in DMF with 0.1 M *n*-Bu_4NClO_4 at different scan speeds. (b) repetitive CV scans of **2F25** in DMF with 0.1 M *n*-Bu_4NClO_4 (scan rate 100 mV/s). Fc/Fc^+ = 0.40 V vs SCE under these conditions. (c). Plots of the current density against one-half square of the scan speed for **2F25** in DMF with 0.1 M *n*-Bu_4NClO_4.

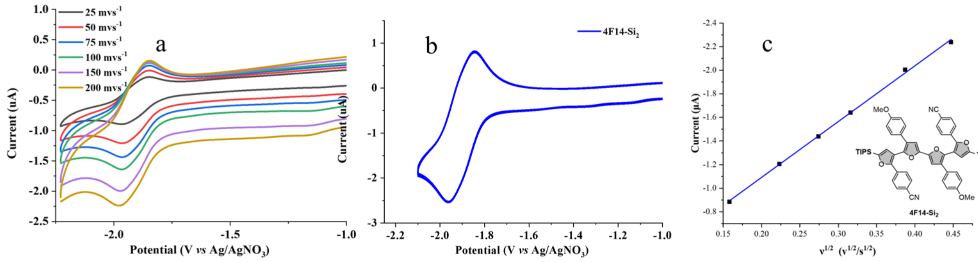

Supplementary Figure 20. (a) CV of **4F14-Si2** in DMF with 0.1 M *n*-Bu4NClO4 at different scan speeds. (b) repetitive CV scans of **4F14-Si2** in DMF with 0.1 M *n*-Bu4NClO4 (scan rate 100 mV/s). Fc/Fc^{+} = 0.40 V vs SCE under these conditions. (c). Plots of the current density against one-half square of the scan speed for **4F14-Si2** in DMF with 0.1 M n-Bu4NClO4.

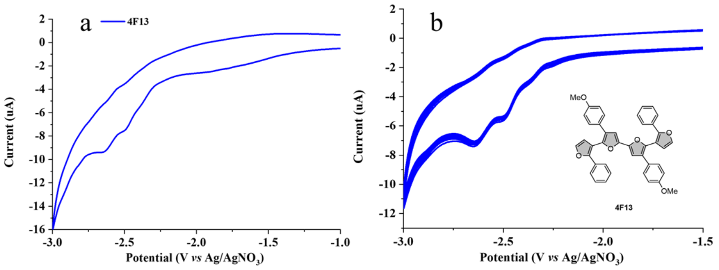

Supplementary Figure 21. (a) CV of **4F13** in DMF with 0.1 M *n*-Bu4NClO4 at 100 mvs^{-1} scan speeds. (b) repetitive CV scans of **4F14-Si2** in DMF with 0.1 M *n*-Bu4NClO4 (scan rate 100 mV/s). Fc/Fc^{+} = 0.40 V vs SCE under these conditions.

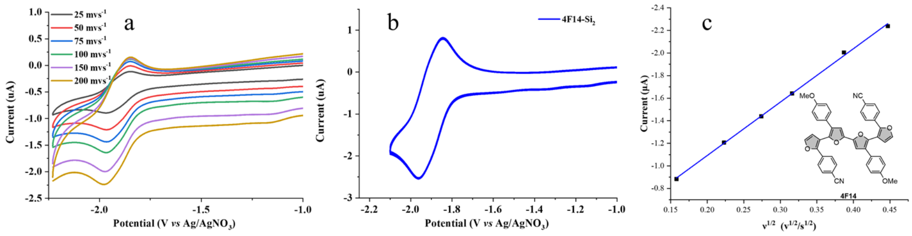

Supplementary Figure 22. (a) CV of **4F14** in DMF with 0.1 M *n*-Bu4NClO4 at different scan speeds. (b) repetitive CV scans of **4F14** in DMF with 0.1 M *n*-Bu4NClO4 (scan rate 100 mV/s). Fc/Fc^{+} = 0.40 V vs SCE under these conditions. (c). Plots of the current density against one-half square of the scan speed for **4F14** in DMF with 0.1 M *n*-Bu4NClO4.

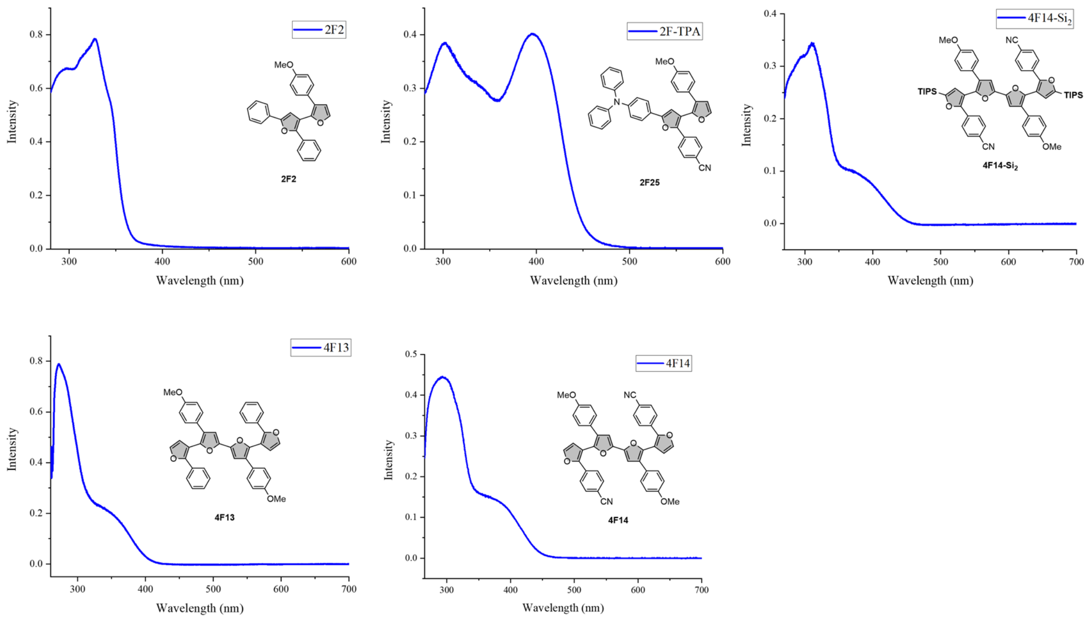

Supplementary Figure 23. Absorption spectra of **2F2**, **2F25**, **4F14-Si2**, **4F13** and **4F14** in DMF (Concentration: 10 μM).

Supplementary Table 6. Physical properties of 2F2, 2F25, 4F14-Si2, 4F13 and 4F14.

Compound	λ_{abs}[a] (nm)	Φ_F[b] (%) THF[c]	Film[d]	Powder	λ_{onset}[e] (nm)	E_{onset}^{re}[f] (V)	HOMO/ LUMO[g] (eV)	E_g[h] (eV)	HOMO/ LUMO[i] (eV)	E_g[i] (eV)
**2F2**	328	17.8/ 19.7	5.5	4.2	381	-2.24	-5.41/ -2.16	3.25	-5.48/ -1.61	3.87
**2F25**	301, 396	80.8/ 90.8	3.0	3.5	468	-1.52	-5.62/ -2.88	2.74	-5.23/ -2.15	3.08
**4F14-Si2**	311, 387	56.3/ 68.0	7.5	11.5	450	-1.80	-5.44/ -2.60	2.84	-5.29/ -2.29	3.00
**4F13**	272, 360	68.3/ 77.9	6.4	38.6	414	-2.12	-5.37/ -2.28	3.09	-4.97/ -1.62	3.35
**4F14**	294, 385	55.0/ 64.5	1.1	1.3	451	-1.89	-5.38/ -2.54	2.84	-5.38/ -2.37	3.01

[a]Measured in DMF (10^{-5} M). [b]Absolute fluorescence quantum yield determined using a calibrated integrating sphere. [c]Measured in THF (10^{-5} M) under air and N_2, respectively. [d]Film drop-casted on a quartz plate. [e]Measured from the onset of absorption in the UV/Vis absorption spectrum of solutions. [f]$\boldsymbol{E}_{onse}$ versus Fc/Fc^{+} estimated by a CV method using a (Pt) disc electrode, Pt wire as a counter electrode, and $Ag/AgNO_3$ as a reference electrode in DMF. [g]Obtained from CV in DMF/n-Bu_4NClO_4 and estimated from LOMO = -(4.40 - E_{onse}^{re}); HUMO = LOMO - E_g. [h]Calculated from the absorption edge, $E_g = 1240/\lambda_{onset}$. [i]Obtained from DFT calculations.

11. Carrier mobility calculation.

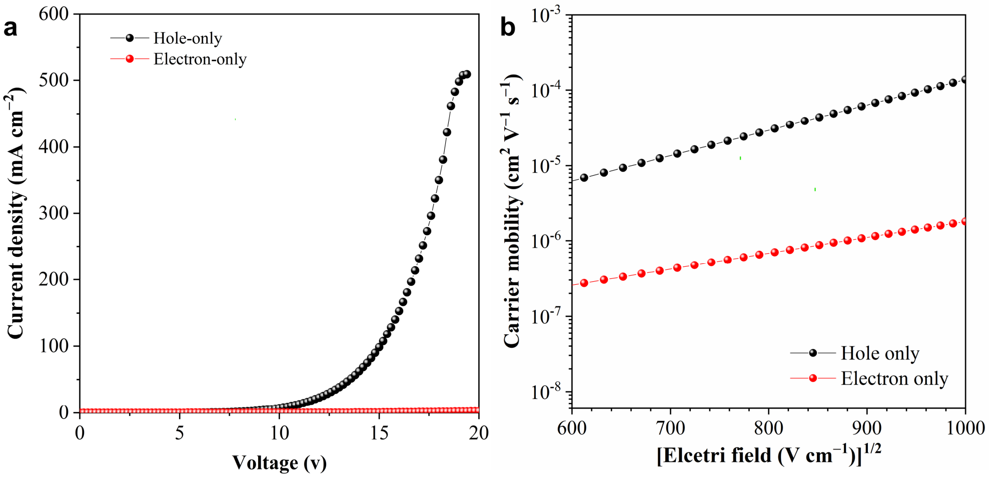

Supplementary Figure 24. (a) Current density–voltage curves of devices H1 and E1. (b) Carrier mobility- electric field curves of devices H1 and E1.

Hole- and electron-only devices with configurations of ITO/TAPC (10 nm)/**4F14-Si$_2$** (80 nm)/TAPC (10 nm)/Al (120 nm) (device H1) and ITO/TmPyPB (10 nm)/ **4F14-Si$_2$** (80 nm)/TmPyPB (10 nm)/LiF (1 nm)/Al (120 nm) (device E1) were fabricated, respectively. In devices H1 and E1, thin layers (10 nm) of TAPC (hole mobility $\mu_h \approx 10^{-2}$ cm^2 V^{-1} s^{-1}) and TmPyPB (electron mobility $\mu_e \approx 10^{-3}$ cm^2 V^{-1} s^{-1}) were used as buffer layers between **4F14-Si$_2$** and the electrodes. Considering the much thicker **4F14-Si$_2$** layer (80 nm), the influence of the thin buffer layers with high carrier mobilities can be excluded during the calculation, and the mobility results primarily reflect the intrinsic property of **4F14-Si$_2$**. The space-charge-limited current (SCLC) property can be described via the Mott-Gurney equation (1), and the carrier mobility (μ) of organic semiconductors can be calculated according to the Poole–Frenkel formula (2), where the ε_0 is the free-space permittivity (8.85×10^{-14} C V^{-1} cm^{-1}), ε_r is the relative dielectric constant (assumed to be 3.0 for organic semiconductors), E is the electric field, μ_0 is the zero-field mobility, and γ is the Poole-Frenkel factor and L is the thickness of **4F14-Si$_2$**.

$$J = \frac{9}{8}\varepsilon_0\varepsilon_r\mu\frac{E^2}{L} = \frac{9}{8}\varepsilon_0\varepsilon_r\frac{V^2}{L^3}\mu_0\exp\left(0.891\gamma\sqrt{\frac{V}{L}}\right) \quad (1)$$

$$\mu = \mu_0\exp\left(\gamma\sqrt{E}\right) \quad (2)$$

By fitting the J–V curves in SCLC region to Equation (2), the μ_0 and γ values are obtained, thus generating the field-dependent electron mobility according to Equation (2).

12. General X-ray Procedures

Experimental Procedure Single crystals of **2F11**, **4F7**, **4F13-Si₂**, **4F14-Si₂**, **4F13** were recrystallized by MeOH/AcOEt solvent system. A suitable crystal was selected and measured on a Agilent SuperNova, Dual, Cu at zero, AtlasS2 diffractometer. The crystal was kept at 100.0(2) K during data collection.

1. Crystallography information for 2F11.

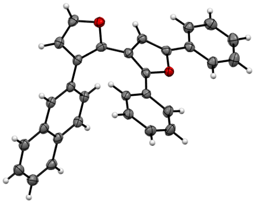

Supplementary Figure 25. Ellipsoid drawing of the crystal structure of **2F11**. The crystal of **2F11** was obtained by slow evaporation of MeOH/AcOEt solution at rt.

(a) **(b)**

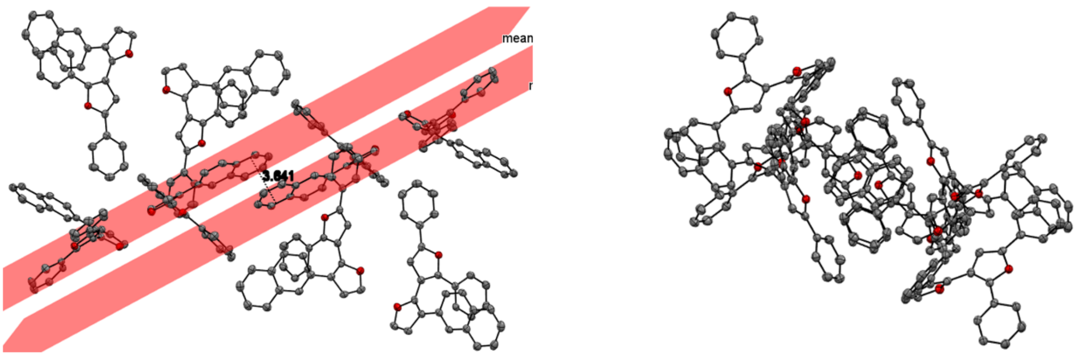

Supplementary Figure 26. (a) The crystal packing of two molecules of **2F11**. The inserted two red planes encompass the furan backbones and distance of 3.641 Å is measured between them; (b) The vertical view of the packing mode in **2F11** (C-H protons are omitted for clarity).

Supplementary Table 7. Crystal data and structure refinement for **2F11.**

CCDC	1989587
Identification code	119-1
Empirical formula	$C_{30}H_{20}O_2$
Formula weight	412.46
Temperature/K	100.00(10)
Crystal system	orthorhombic
Space group	Pbcn
a/Å	34.1192(10)
b/Å	5.9359(2)
c/Å	20.7496(6)
α/°	90

β/°	90
γ/°	90
Volume/Å^3	4202.4(2)
Z	8
ρ_{calc}g/cm^3	1.304
μ/mm^{-1}	0.080
F(000)	1728.0
Crystal size/mm^3	0.13 × 0.12 × 0.11
Radiation	MoKα (λ = 0.71073)
2Θ range for data collection/°	3.926 to 49.998
Index ranges	$-37 \leqslant h \leqslant 40, -7 \leqslant k \leqslant 7, -20 \leqslant l \leqslant 24$
Reflections collected	27254
Independent reflections	3693 [R_{int}= 0.0421, R_{sigma}= 0.0300]
Data/restraints/parameters	3693/0/289
Goodness-of-fit on F^2	1.005
Final R indexes [I>=2σ (I)]	R_1= 0.0398, wR_2 = 0.0935
Final R indexes [all data]	R_1= 0.0539, wR_2 = 0.1031
Largest diff. peak/hole / e Å^{-3}	0.15/-0.17

2. Crystallography information for 4F7.

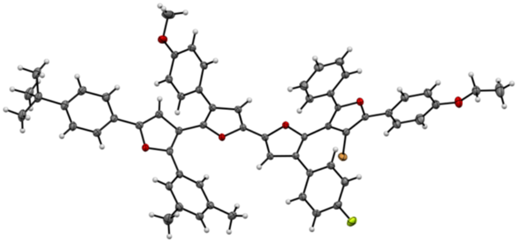

Supplementary Figure 27. Ellipsoid drawing of the crystal structure of **4F7**. The crystal of **4F7** was obtained by slow evaporation of MeOH/AcOEt solution at rt.

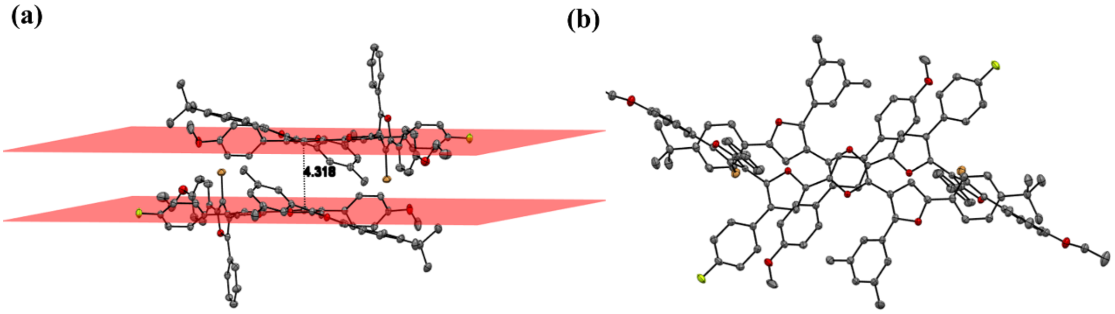

Supplementary Figure 28. (a) The crystal packing of two molecules of **4F7**. The inserted two red planes encompass the furan backbones and distance of 4.318 Å is measured between them; (b) The vertical view of the packing mode in **4F7** (C-H protons are omitted for clarity)

Supplementary Table 8. Crystal data and structure refinement for 4F7.

CCDC	1989589
Identification code	EA-2
Empirical formula	$C_{61}H_{50}BrFO_6$
Formula weight	977.92
Temperature/K	100.00(10)
Crystal system	triclinic
Space group	P-1
a/Å	12.9533(4)
b/Å	13.8928(5)
c/Å	17.2197(5)
α/°	87.642(3)
β/°	73.975(3)
γ/°	64.049(3)
Volume/Å^3	2666.67(17)
Z	2
ρ_{calc}g/cm^3	1.218
μ/mm^{-1}	1.473
F(000)	1016.0
Crystal size/mm^3	0.13 × 0.12 × 0.11
Radiation	CuKα (λ = 1.54184)
2Θ range for data collection/°	5.362 to 147.366
Index ranges	-13 ⩽ h ⩽ 16, -17 ⩽ k ⩽ 14, -20 ⩽ l ⩽ 21
Reflections collected	19228
Independent reflections	10451 [R_{int} = 0.0400, R_{sigma} = 0.0510]
Data/restraints/parameters	10451/0/629
Goodness-of-fit on F^2	1.058
Final R indexes [I>=2σ (I)]	R_1 = 0.0552, wR_2 = 0.1559
Final R indexes [all data]	R_1 = 0.0616, wR_2 = 0.1618
Largest diff. peak/hole / e Å^{-3}	1.67/-0.96

3. Crystallography information for 4F13-Si$_2$.

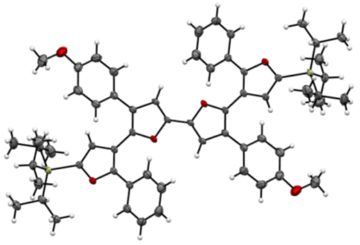

Supplementary Figure 29. Ellipsoid drawing of the crystal structure of **4F13-Si$_2$**. The crystal of **4F13-Si$_2$** was obtained by slow evaporation of MeOH/AcOEt solution at rt.

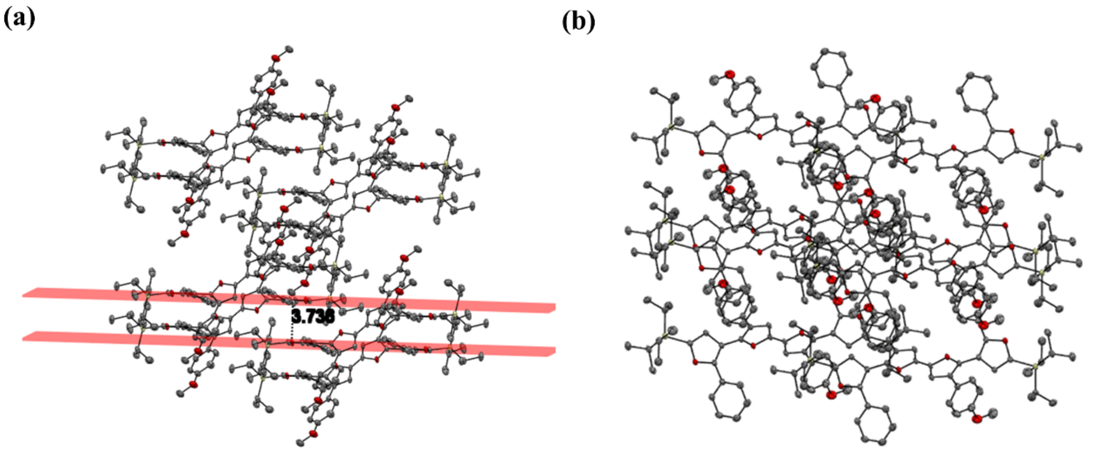

Supplementary Figure 30. (a) The crystal packing of two molecules of **4F13-Si$_2$**. The inserted two red planes encompass the furan backbones and distance of 3.736 Å is measured between them; (b) The vertical view of the packing mode in **4F13-Si$_2$** (C-H protons are omitted for clarity).

Supplementary Table 9. Crystal data and structure refinement for 4F13-Si$_2$.

CCDC	2030795
Identification code	11-2-B
Empirical formula	$C_{60}H_{70}O_6Si_2$
Formula weight	943.34
Temperature/K	149.99(10)
Crystal system	triclinic
Space group	P-1
a/Å	8.5487(4)
b/Å	11.2835(6)
c/Å	14.9313(9)
α/°	106.309(5)
β/°	100.763(5)
γ/°	104.098(4)
Volume/Å^3	1289.33(13)
Z	1
ρ_{calc}g/cm^3	1.215
μ/mm^{-1}	1.024
F(000)	506.0
Crystal size/mm^3	0.14 × 0.1 × 0.08
Radiation	Cu Kα (λ = 1.54184)
2Θ range for data collection/°	6.414 to 147.574
Index ranges	$-10 \leqslant h \leqslant 7, -13 \leqslant k \leqslant 13, -18 \leqslant l \leqslant 18$
Reflections collected	8812
Independent reflections	5037 [R_{int} = 0.0622, R_{sigma} = 0.0806]
Data/restraints/parameters	5037/0/314

Goodness-of-fit on F^2	1.034
Final R indexes [I>=2σ (I)]	$R_1 = 0.0598$, $wR_2 = 0.1504$
Final R indexes [all data]	$R_1 = 0.0776$, $wR_2 = 0.1683$
Largest diff. peak/hole / e Å^{-3}	0.39/-0.37

4. Crystallography information for 4F14-Si$_2$.

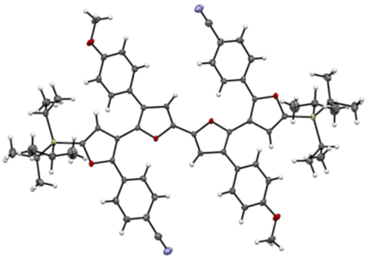

Supplementary Figure 31. Ellipsoid drawing of the crystal structure of **4F14-Si$_2$**. The crystal of **4F14-Si$_2$** was obtained by slow evaporation of MeOH/AcOEt solution at rt.

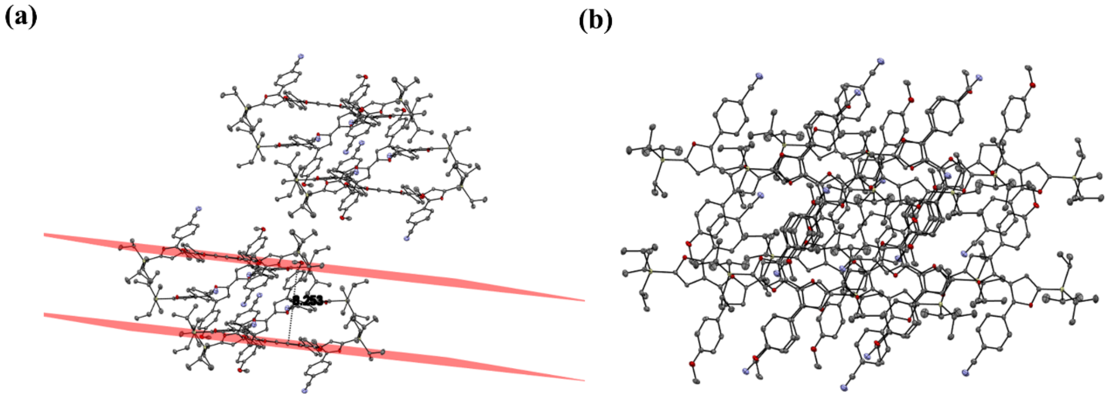

Supplementary Figure 32. (a) The crystal packing of two molecules of **4F14-Si$_2$**. The inserted two red planes encompass the furan backbones and distance of 8.253 Å is measured between them; (b) The vertical view of the packing mode in **4F14-Si$_2$** (C-H protons are omitted for clarity)

Supplementary Table 9. Crystal data and structure refinement for 4F14-Si$_2$.

CCDC	2030794
Identification code	338-3
Empirical formula	$C_{62}H_{68}N_2O_6Si_2$
Formula weight	993.36
Temperature/K	133(40)
Crystal system	triclinic
Space group	P-1
a/Å	11.6260(9)
b/Å	12.8431(19)
c/Å	19.3368(18)
α/°	79.206(12)
β/°	73.307(8)
γ/°	85.035(9)

Volume/Å^3	2715.0(5)
Z	2
ρ_{calc}g/cm^3	1.215
μ/mm^{-1}	0.119
F(000)	1060.0
Crystal size/mm^3	0.13 × 0.12 × 0.11
Radiation	Mo Kα (λ = 0.71073)
2Θ range for data collection/°	3.722 to 49.996
Index ranges	-13 ⩽ h ⩽ 13, -12 ⩽ k ⩽ 15, -22 ⩽ l ⩽ 22
Reflections collected	20298
Independent reflections	9557 [R_{int} = 0.0413, R_{sigma} = 0.0694]
Data/restraints/parameters	9557/0/663
Goodness-of-fit on F^2	1.043
Final R indexes [I>=2σ (I)]	R_1 = 0.0515, wR_2 = 0.1074
Final R indexes [all data]	R_1 = 0.0692, wR_2 = 0.1178
Largest diff. peak/hole / e Å^{-3}	0.40/-0.33

5. Crystallography information for 4F13.

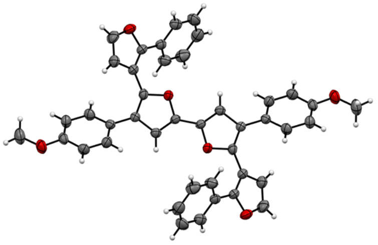

Supplementary Figure 33. Ellipsoid drawing of the crystal structure of **4F13**. The crystal of **4F13** was obtained by slow evaporation of MeOH/AcOEt solution at rt.

(a) **(b)**

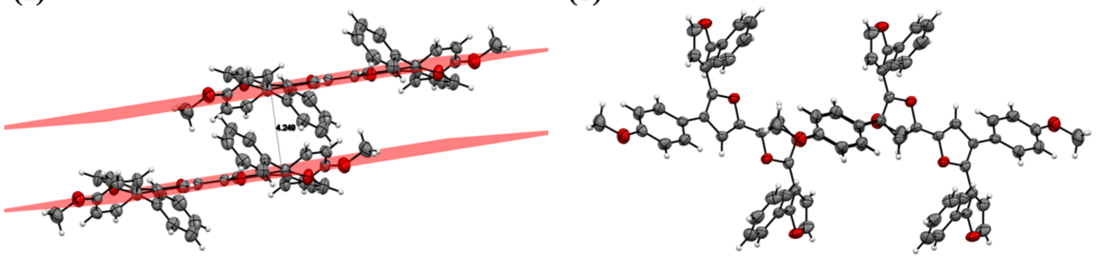

Supplementary Figure 34. (a) The crystal packing of two molecules of **4F13**. The inserted two red planes encompass the furan backbones and distance of 4.249 Å is measured between them; (b) The vertical view of the packing mode in **4F13** (C-H protons are omitted for clarity)

Supplementary Table 9. Crystal data and structure refinement for 4F13.

CCDC	2096030
Identification code	exp_9268
Empirical formula	$C_{21}H_{15}O_3$
Formula weight	315.33
Temperature/K	293(2)
Crystal system	triclinic
Space group	P-1
a/Å	9.2565(8)
b/Å	9.2655(7)
c/Å	9.6672(8)
α/°	100.506(7)
β/°	101.912(7)
γ/°	94.371(7)
Volume/Å^3	792.06(12)
Z	2
ρ_{calc}g/cm^3	1.322
μ/mm^{-1}	0.088
F(000)	330.0
Crystal size/mm^3	0.21 × 0.18 × 0.1
Radiation	Mo Kα (λ = 0.71073)
2Θ range for data collection/°	6.756 to 49.998
Index ranges	-10 ≤ h ≤ 11, -11 ≤ k ≤ 10, -11 ≤ l ≤ 11
Reflections collected	6657
Independent reflections	2769 [R_{int} = 0.0479, R_{sigma} = 0.0921]
Data/restraints/parameters	2769/0/218
Goodness-of-fit on F^2	1.039
Final R indexes [I>=2σ (I)]	R_1 = 0.0628, wR_2 = 0.0946
Final R indexes [all data]	R_1 = 0.1169, wR_2 = 0.1149
Largest diff. peak/hole / e Å^{-3}	0.18/-0.20

III. Supplementary NMR Spectra

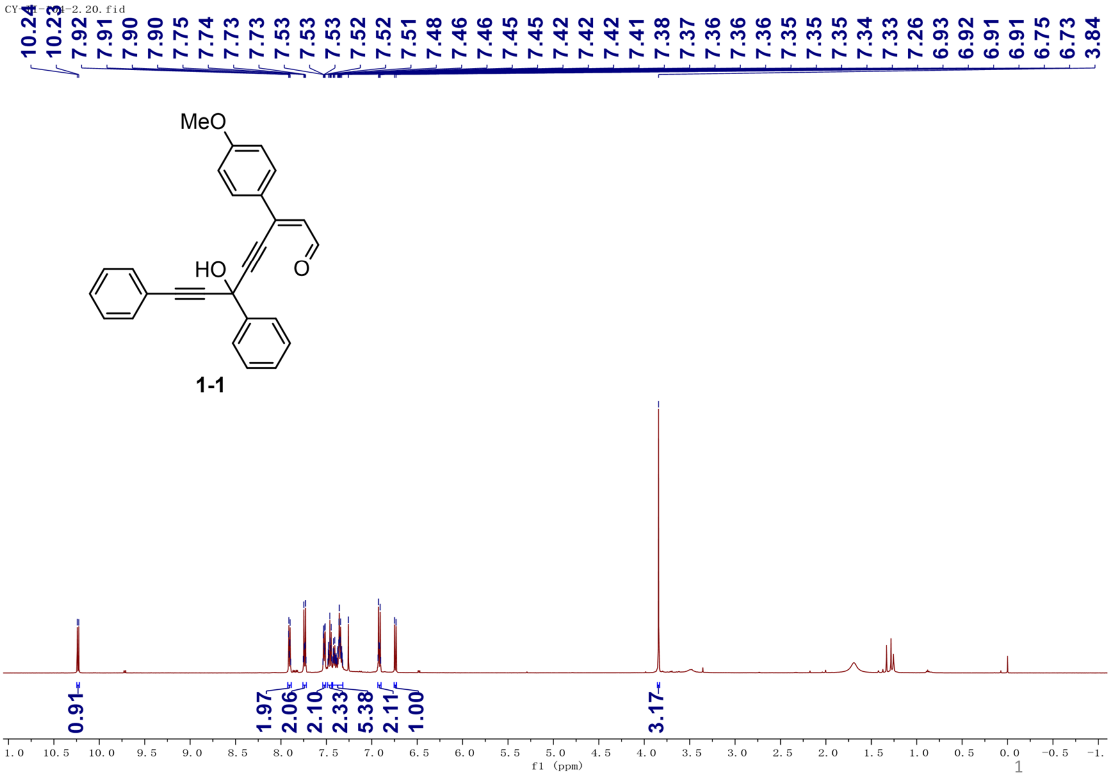

Supplementary Figure 35. ^{1}H NMR (500 MHz, $CDCl_3$) spectra for compound **1-1**

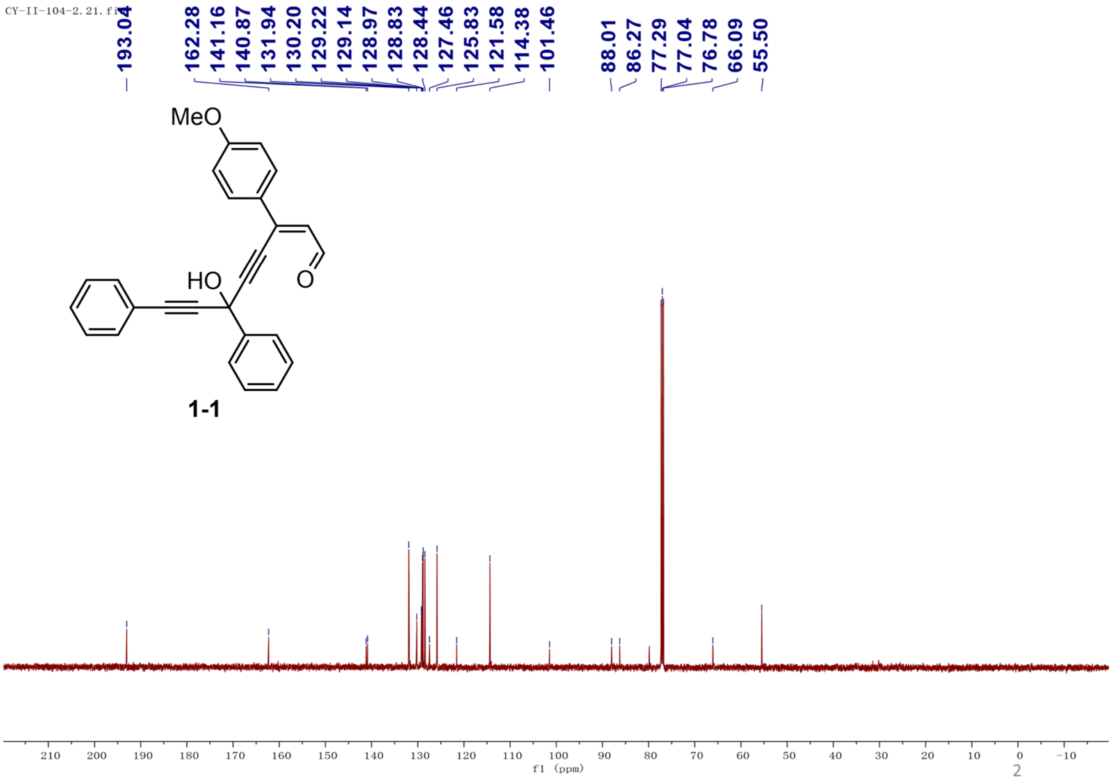

Supplementary Figure 36. ^{13}C NMR (126 MHz, $CDCl_3$) spectra for compound **1-1**

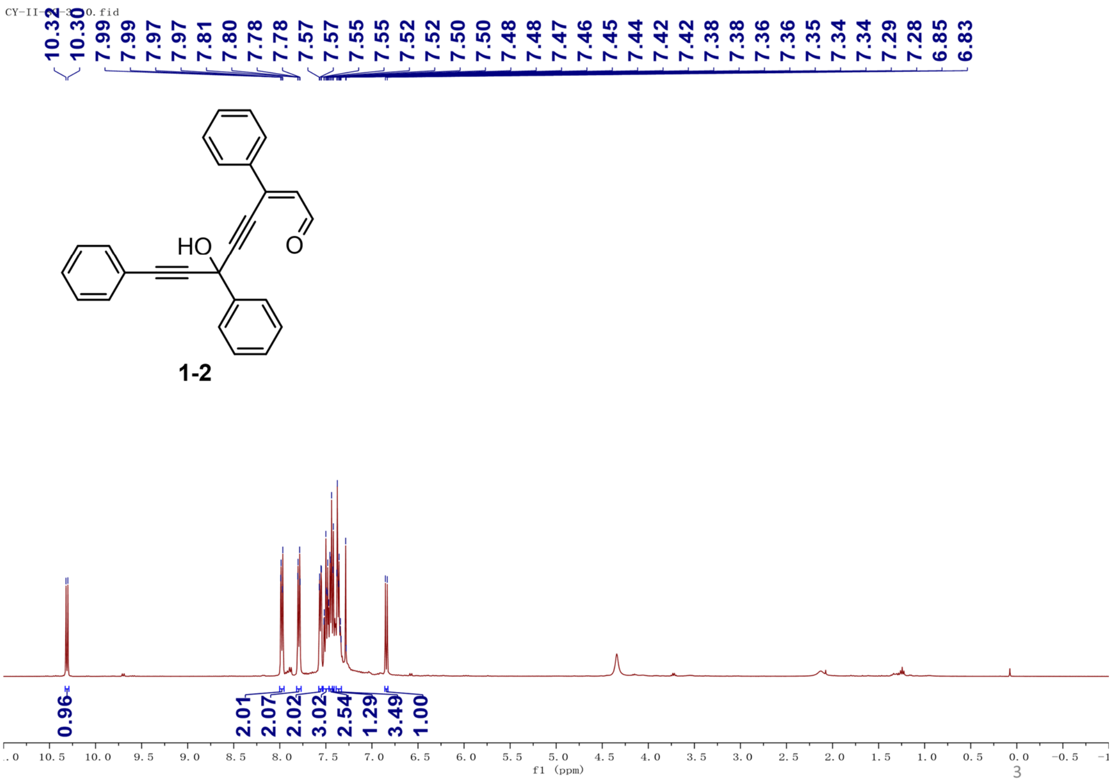

Supplementary Figure 37. ^{1}H NMR (400 MHz, $CDCl_3$) spectra for compound **1-2**

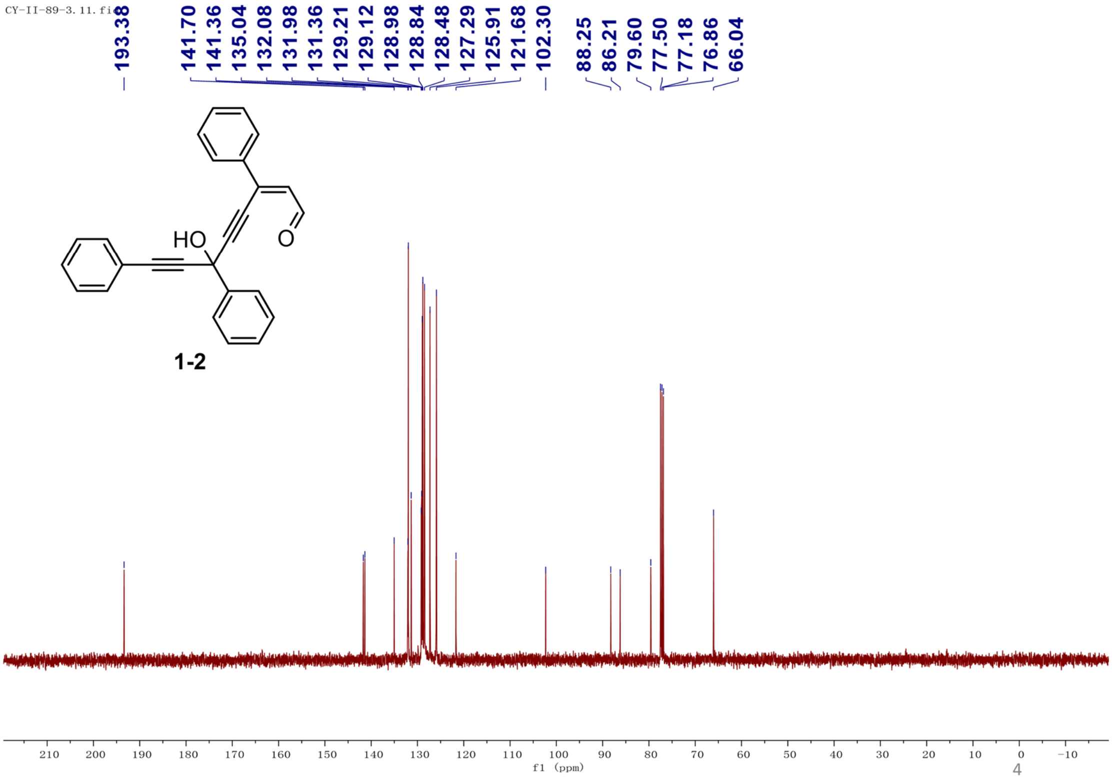

Supplementary Figure 38. ^{13}C NMR (101 MHz, $CDCl_3$) spectra for compound **1-2**

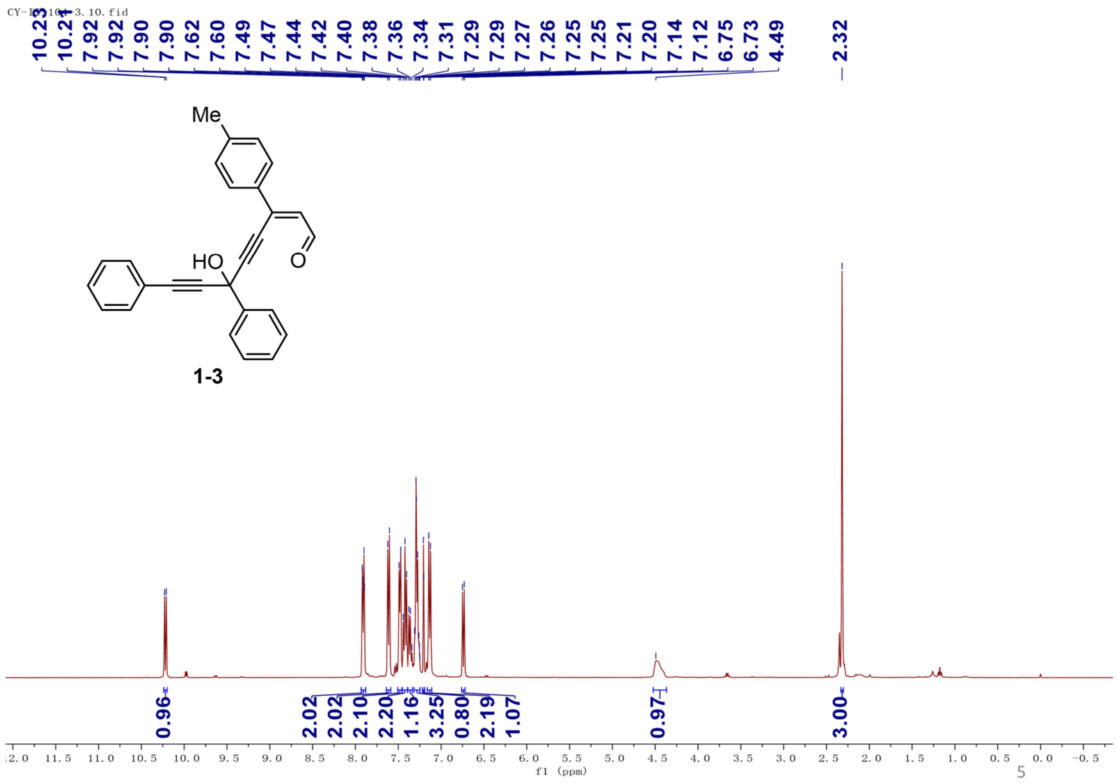

Supplementary Figure 39. ^{1}H NMR (400 MHz, $CDCl_3$) spectra for compound **1-3**

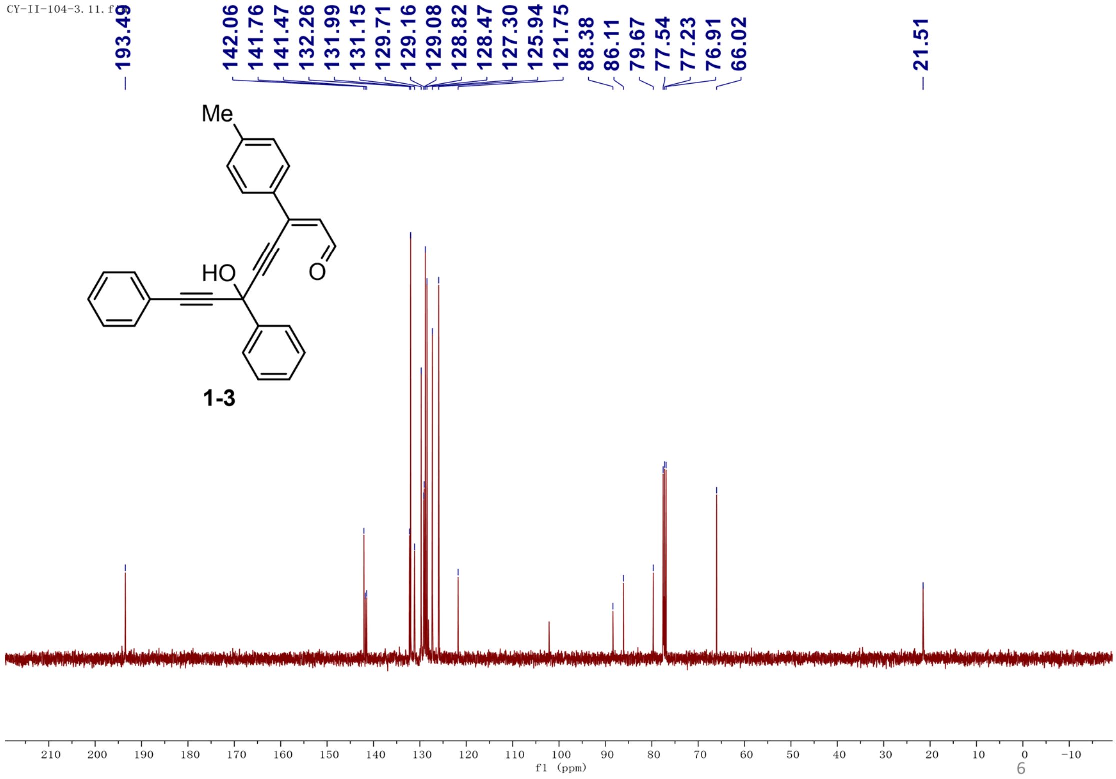

Supplementary Figure 40. ^{13}C NMR (101 MHz, $CDCl_3$) spectra for compound **1-3**

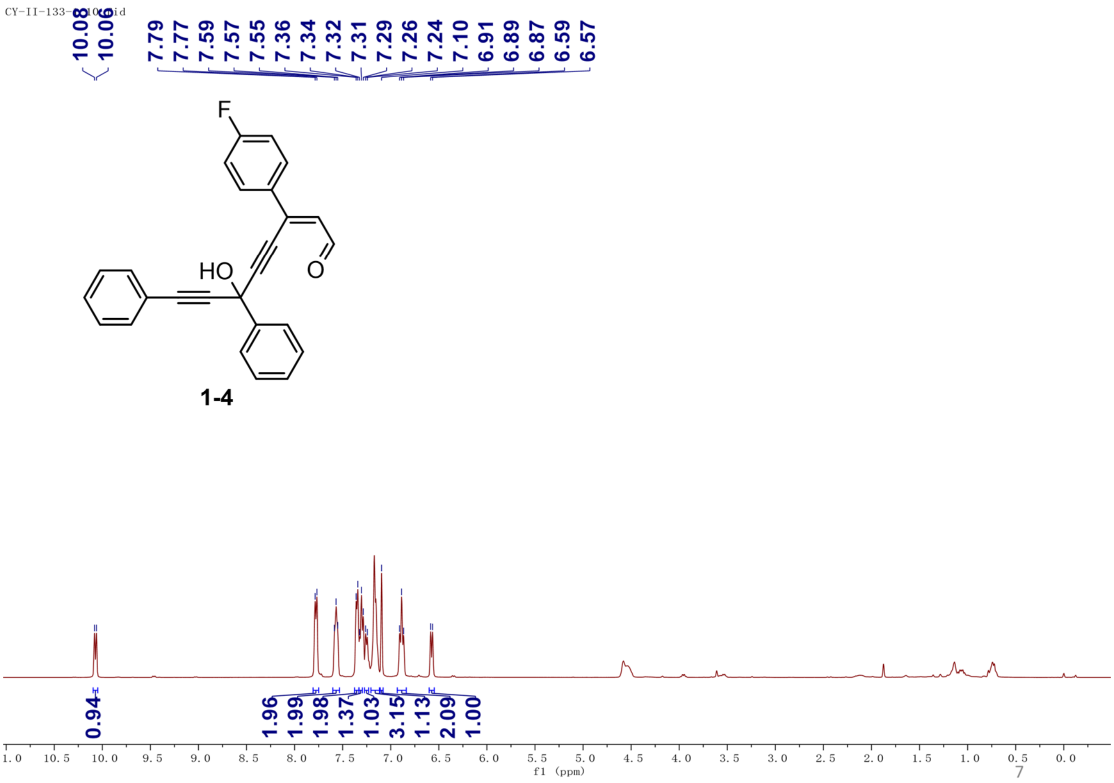

Supplementary Figure 41. ^{1}H NMR (400 MHz, $CDCl_3$) spectra for compound **1-4**

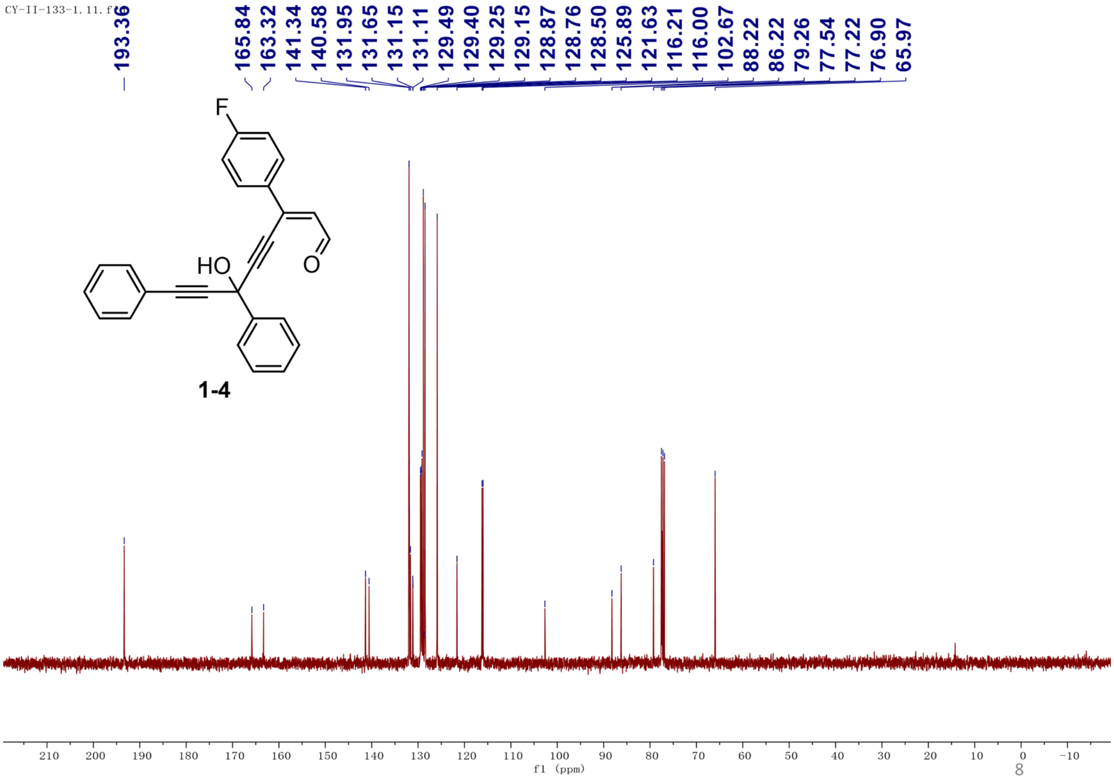

Supplementary Figure 42. ^{13}C NMR (101 MHz, $CDCl_3$) spectra for compound **1-4**

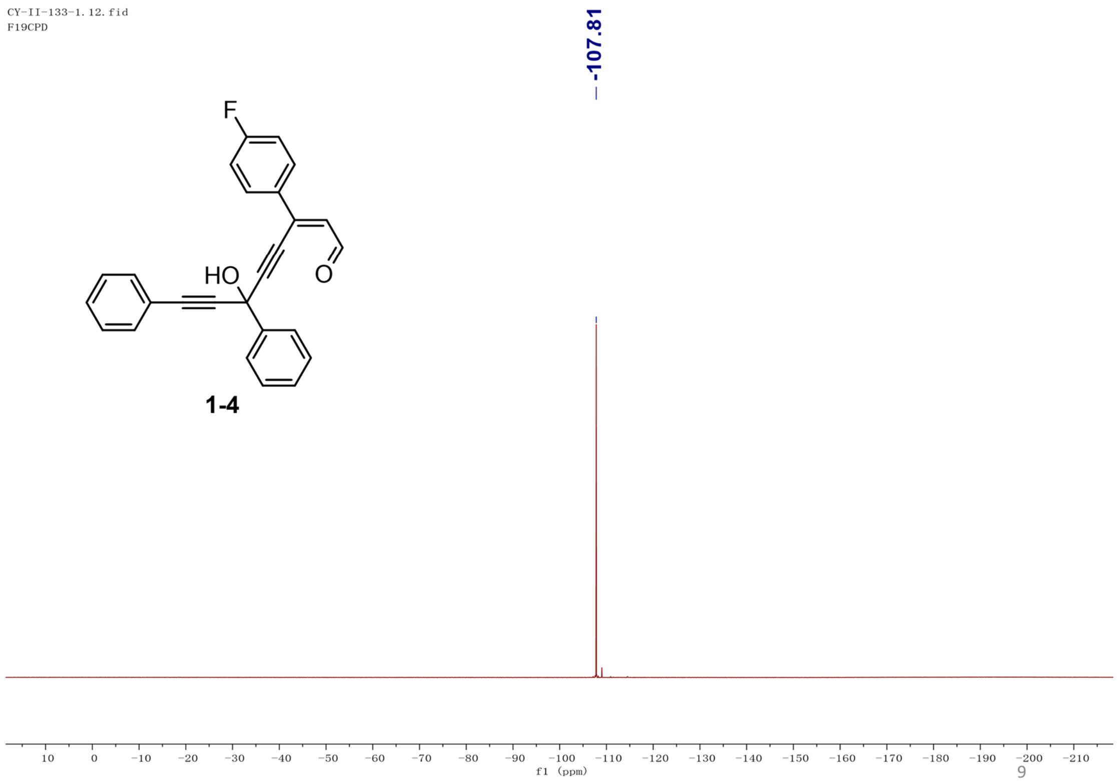

Supplementary Figure 43. ^{19}F NMR (376 MHz, $CDCl_3$) spectra for compound **1-4**

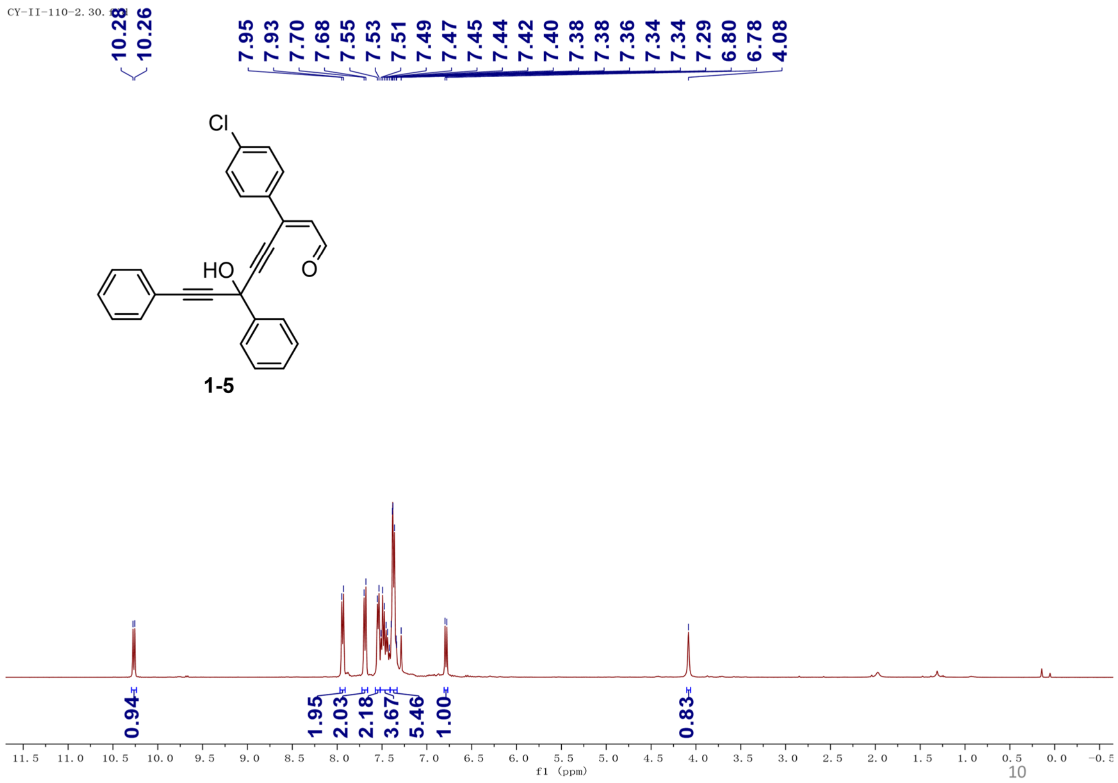

Supplementary Figure 44. ^{1}H NMR (400 MHz, $CDCl_3$) spectra for compound **1-5**

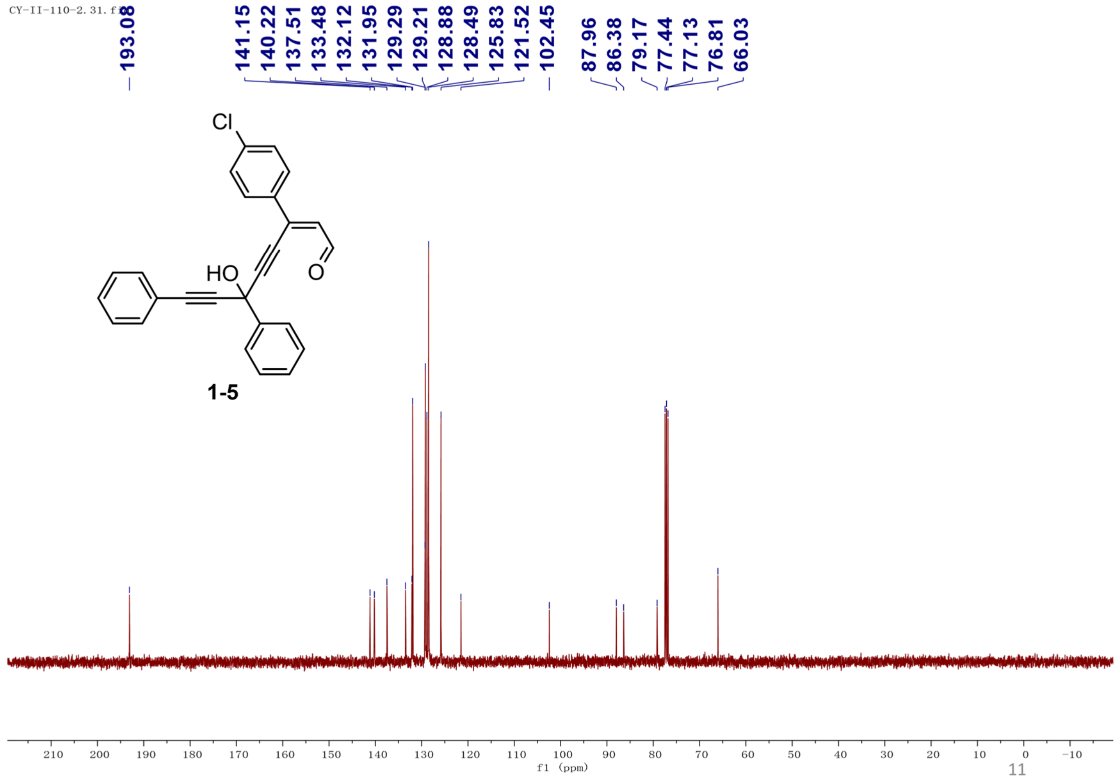

Supplementary Figure 45. ^{13}C NMR (101 MHz, $CDCl_3$) spectra for compound **1-5**

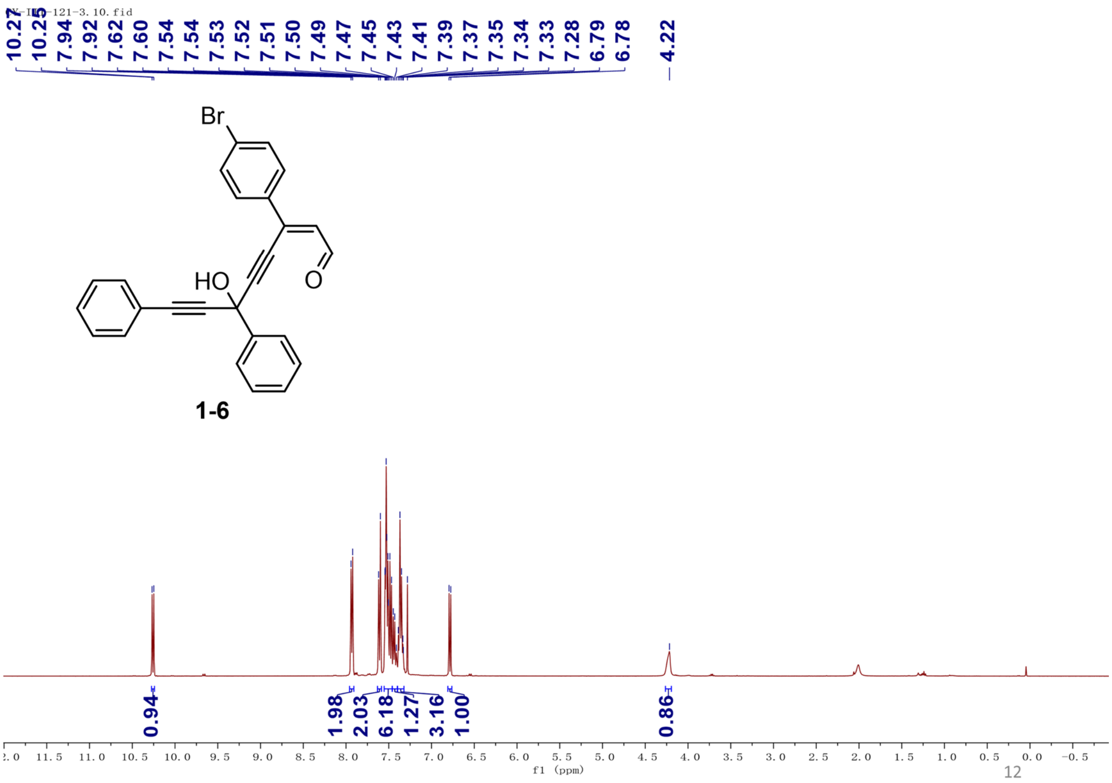

Supplementary Figure 46. ^{1}H NMR (400 MHz, $CDCl_3$) spectra for compound **1-6**

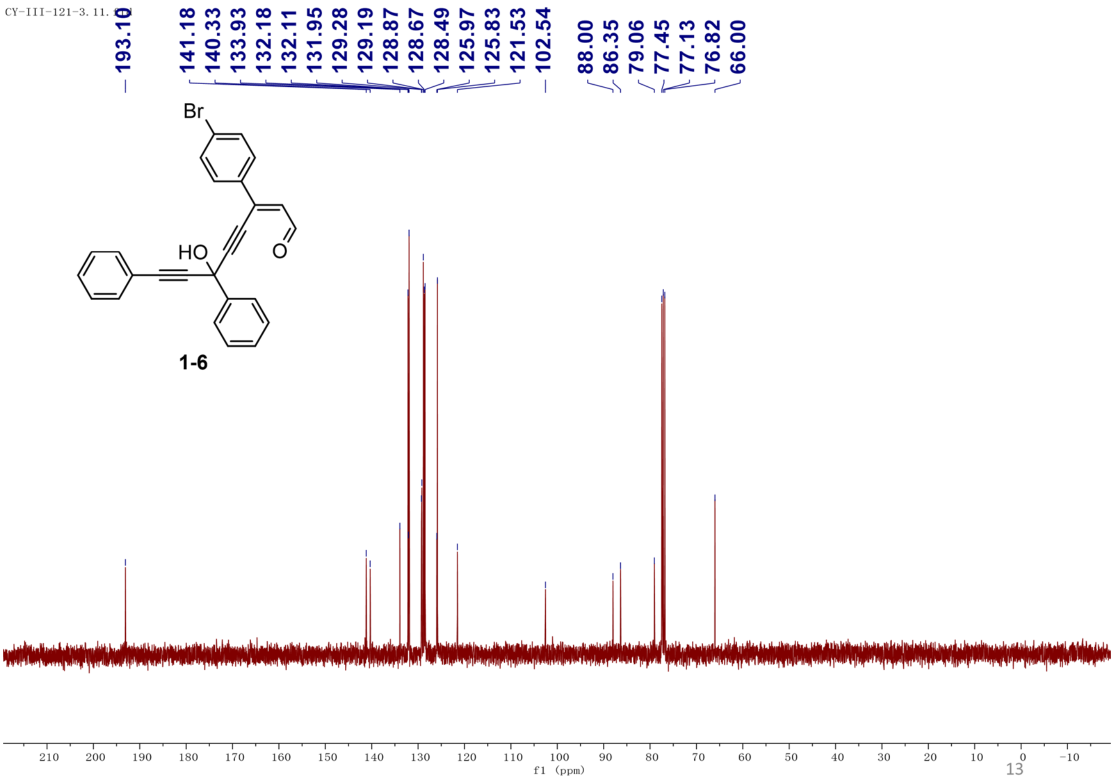

Supplementary Figure 47. ^{13}C NMR (101 MHz, $CDCl_3$) spectra for compound **1-6**

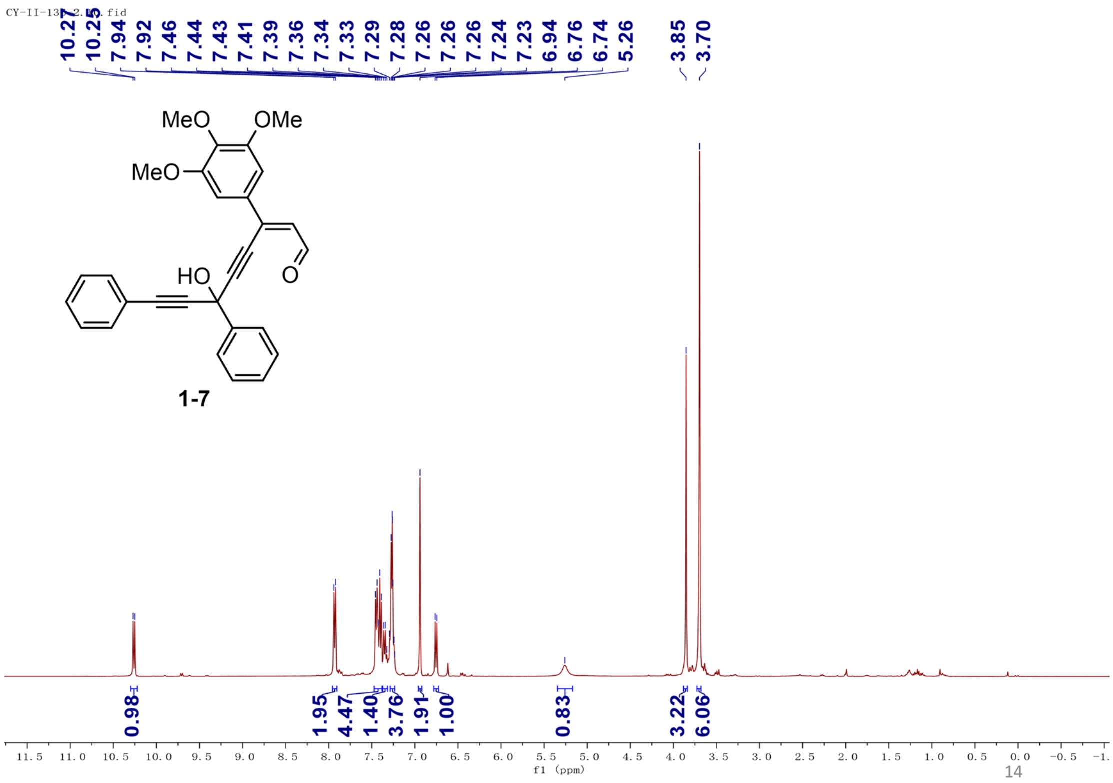

Supplementary Figure 48. ^{1}H NMR (400 MHz, $CDCl_3$) spectra for compound **1-7**

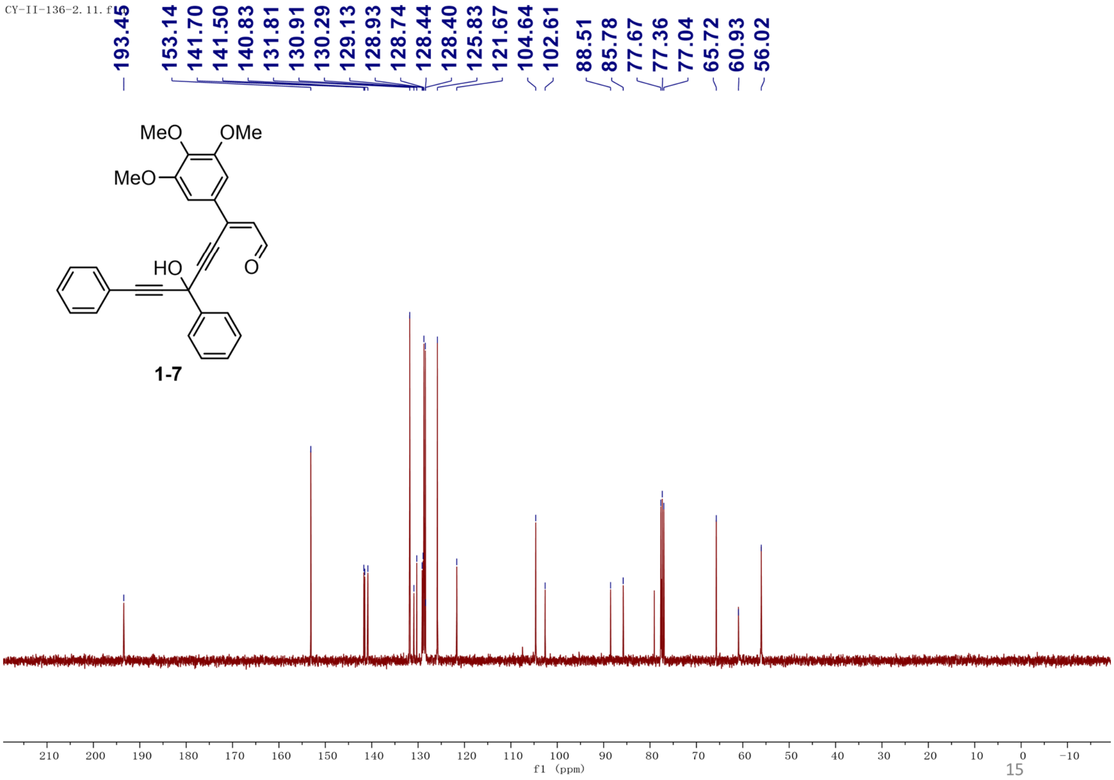

Supplementary Figure 49. ^{13}C NMR (101 MHz, $CDCl_3$) spectra for compound **1-7**

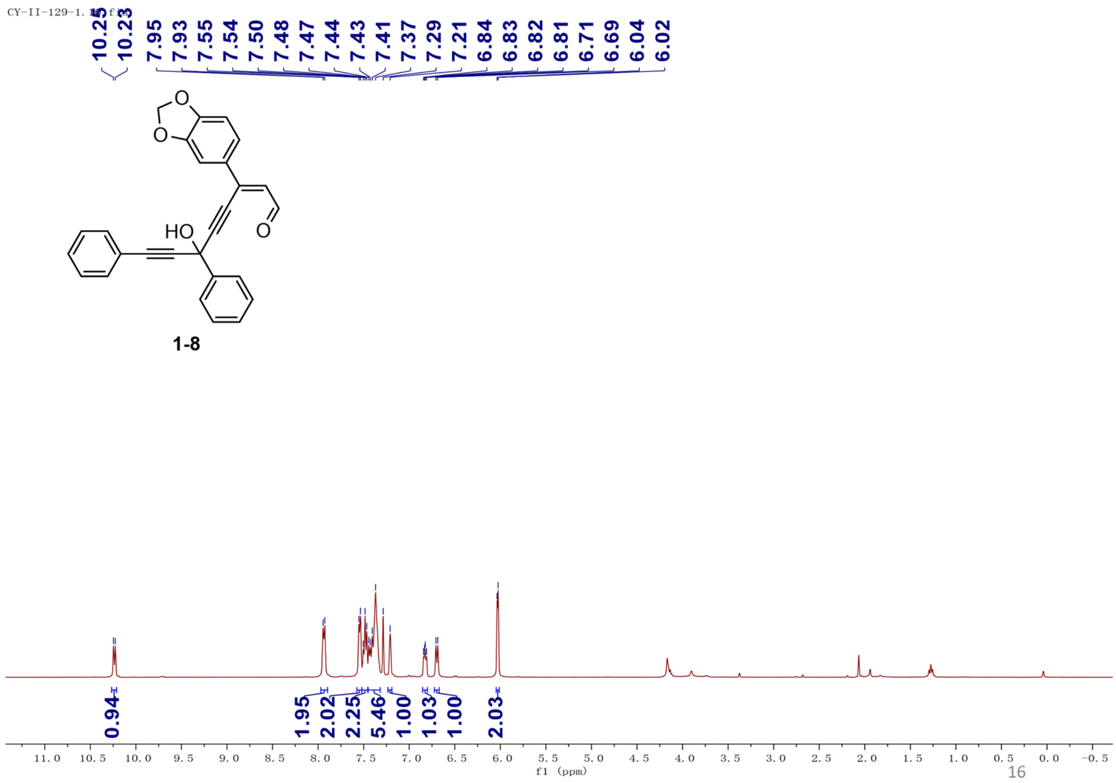

Supplementary Figure 50. ^{1}H NMR (400 MHz, $CDCl_3$) spectra for compound **1-8**

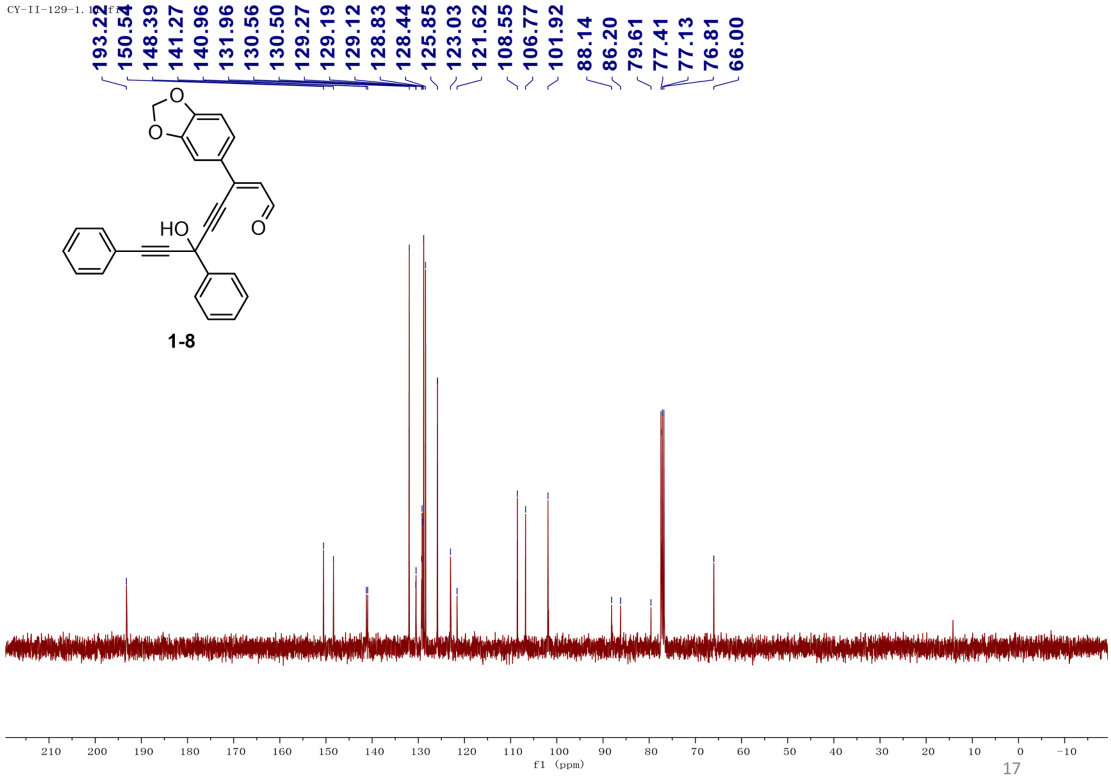

Supplementary Figure 51. ^{13}C NMR (101 MHz, $CDCl_3$) spectra for compound **1-8**

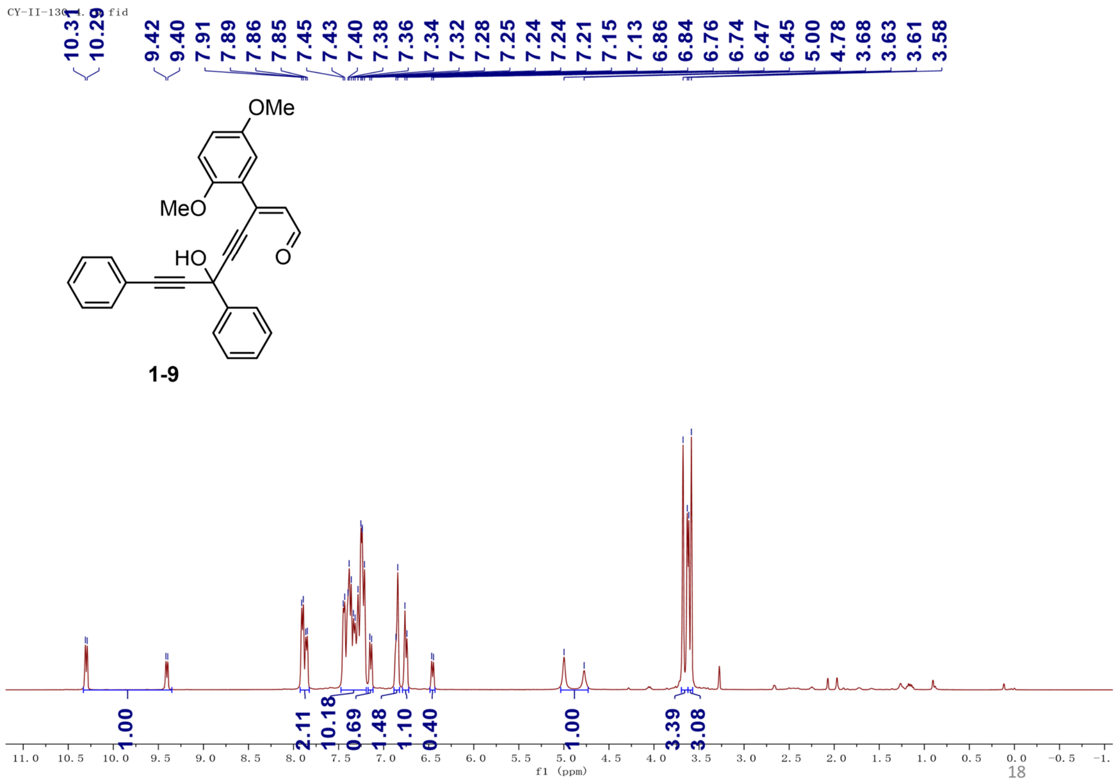

Supplementary Figure 52. ^{1}H NMR (400 MHz, $CDCl_3$) spectra for compound **1-9**

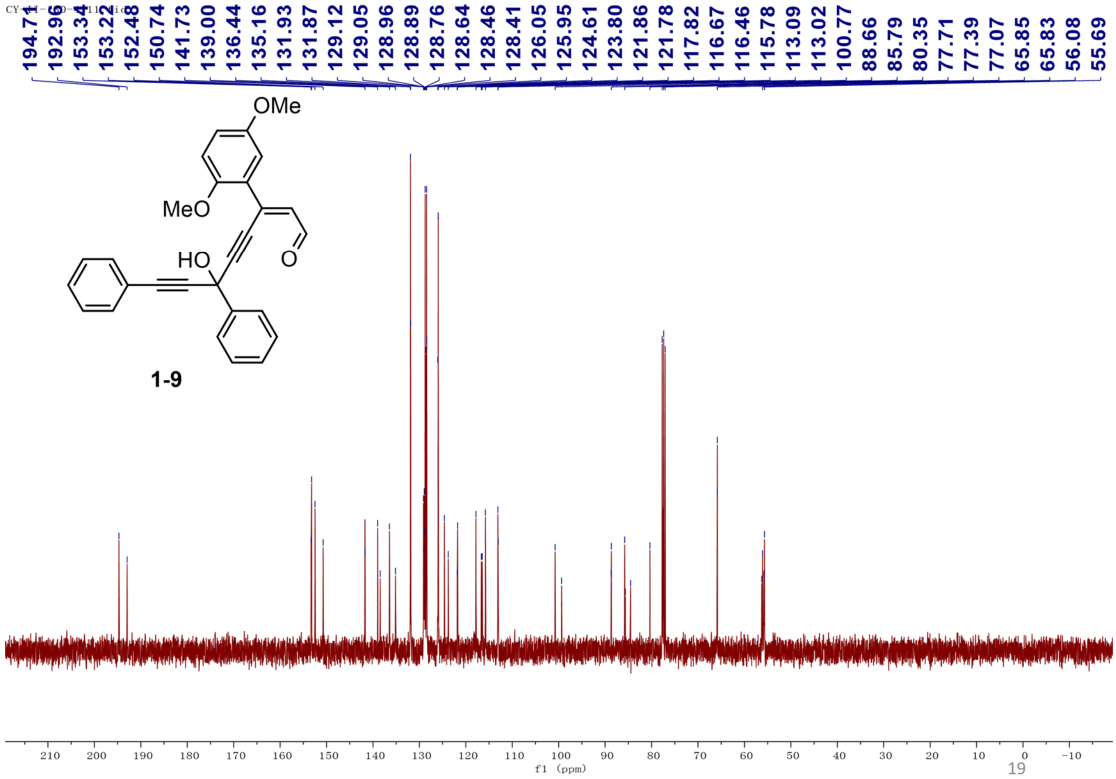

Supplementary Figure 53. ^{13}C NMR (101 MHz, $CDCl_3$) spectra for compound **1-9**

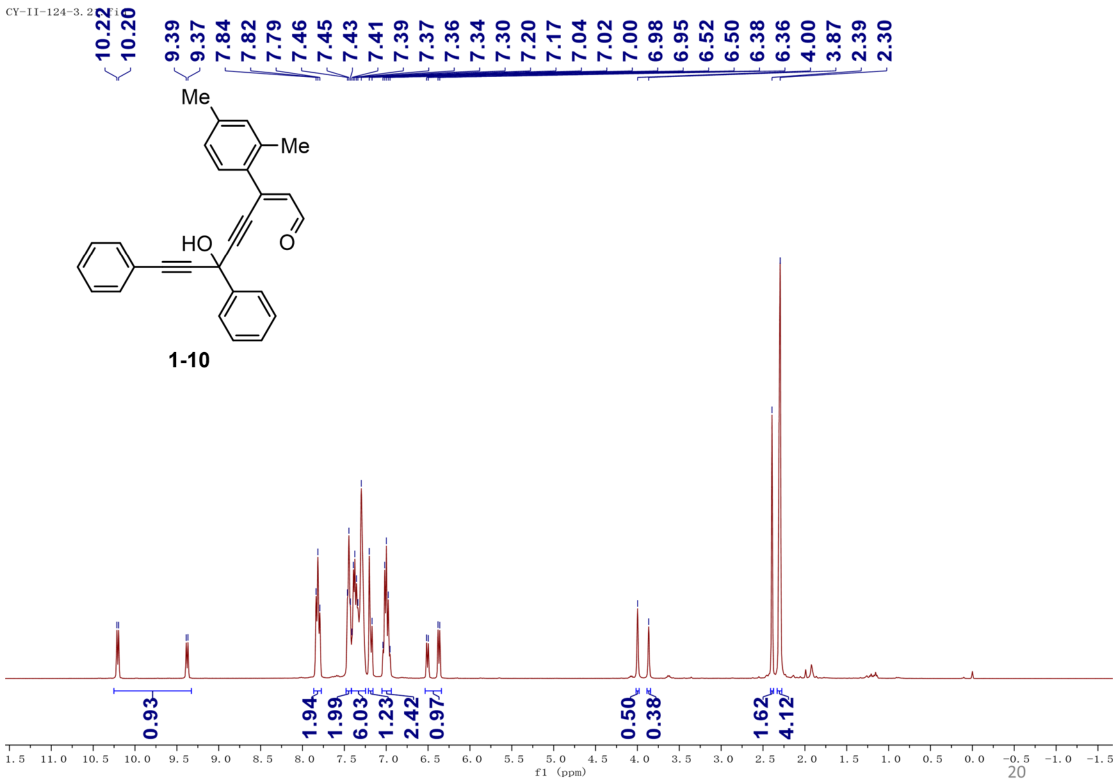

Supplementary Figure 54. ^{1}H NMR (400 MHz, $CDCl_3$) spectra for compound **1-10**

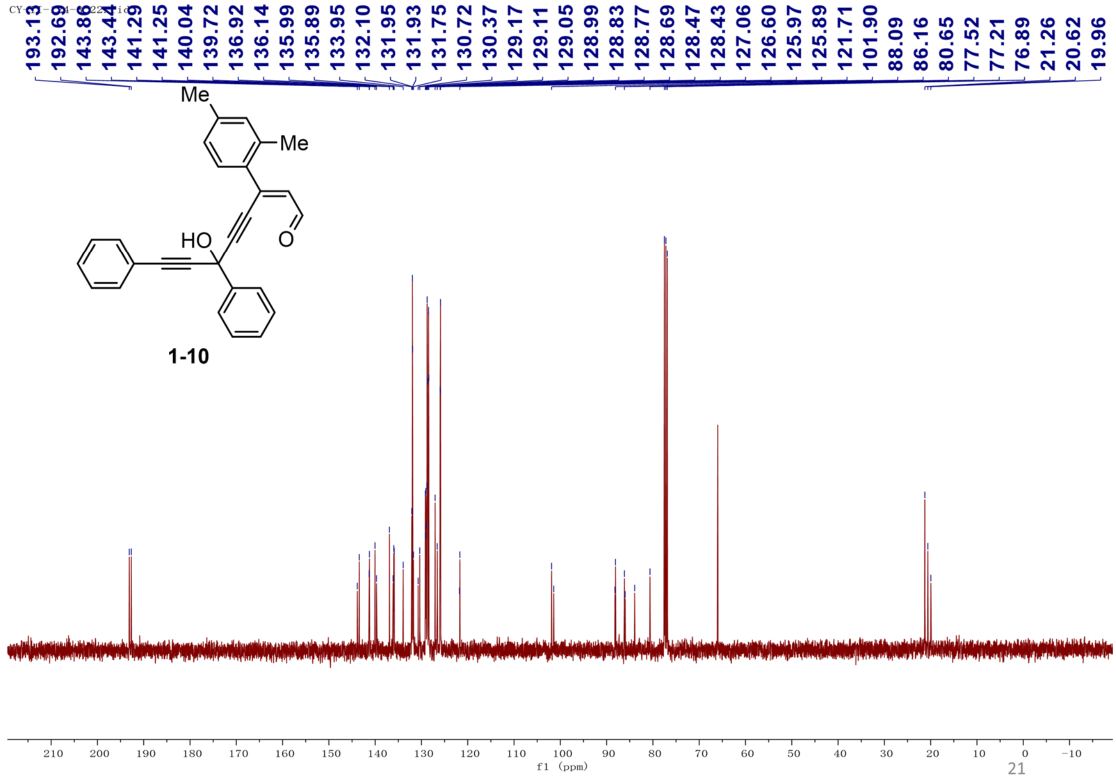

Supplementary Figure 55. ^{13}C NMR (101 MHz, $CDCl_3$) spectra for compound **1-10**

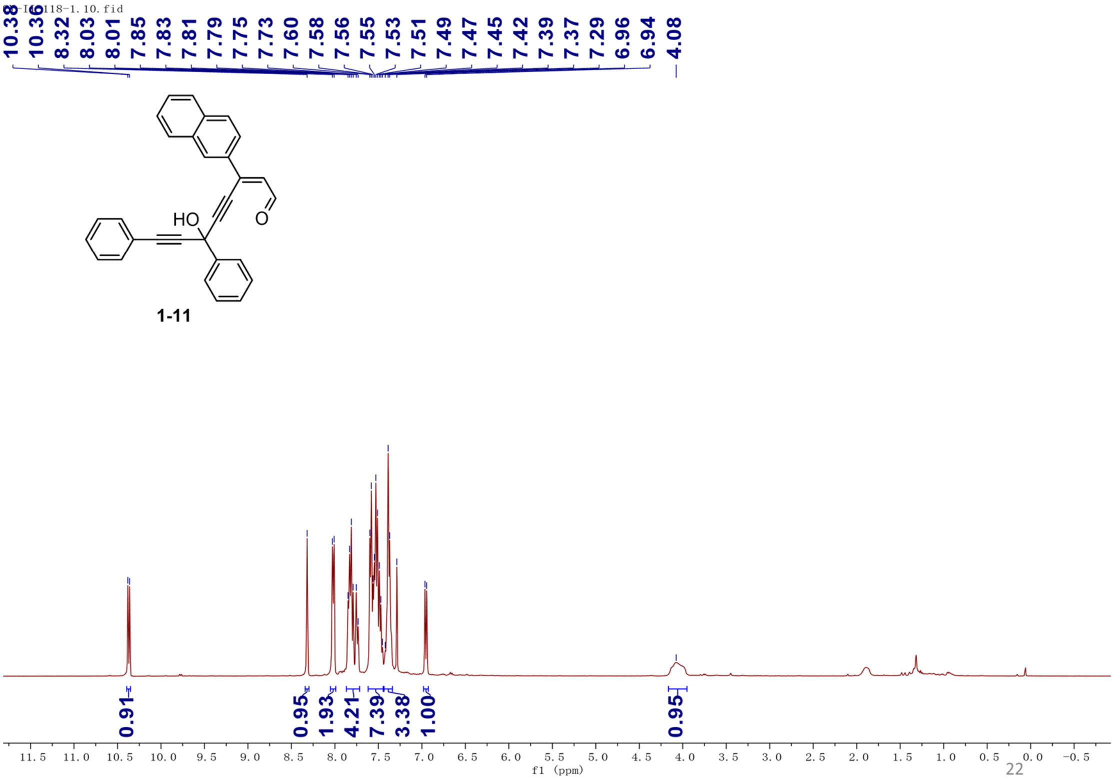

Supplementary Figure 56. ^{1}H NMR (400 MHz, $CDCl_3$) spectra for compound **1-11**

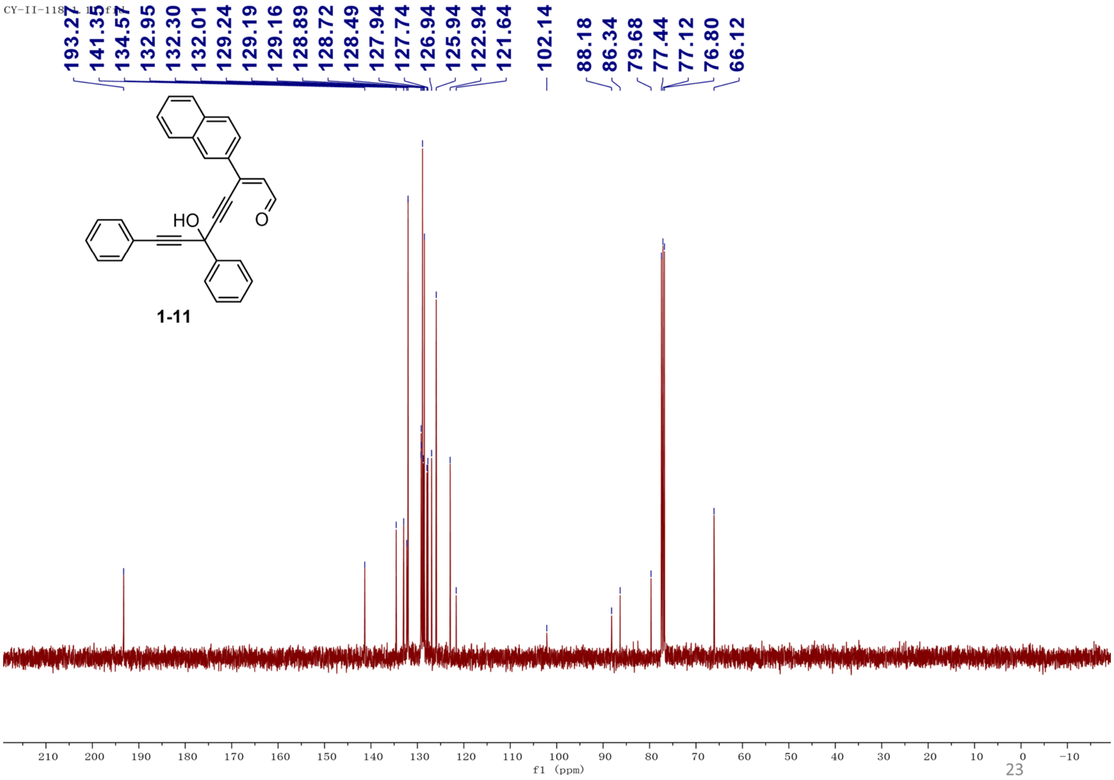

Supplementary Figure 57. ^{13}C NMR (101 MHz, $CDCl_3$) spectra for compound **1-11**

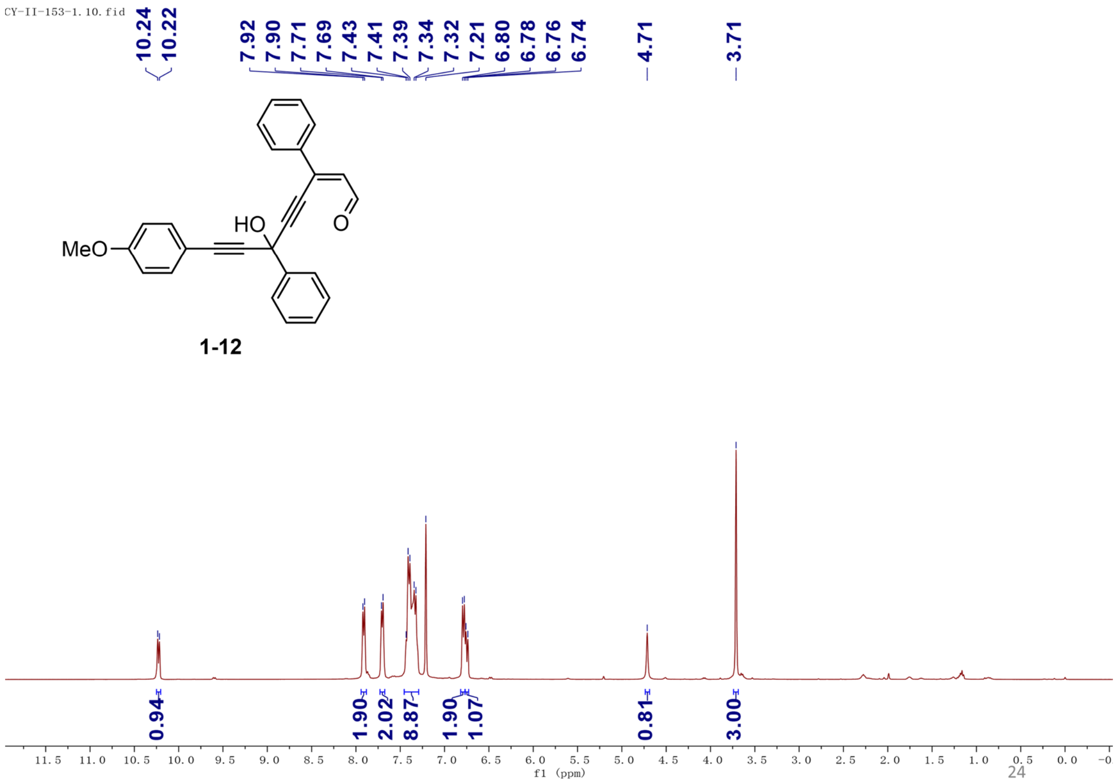

Supplementary Figure 58. ^{1}H NMR (400 MHz, $CDCl_3$) spectra for compound **1-12**

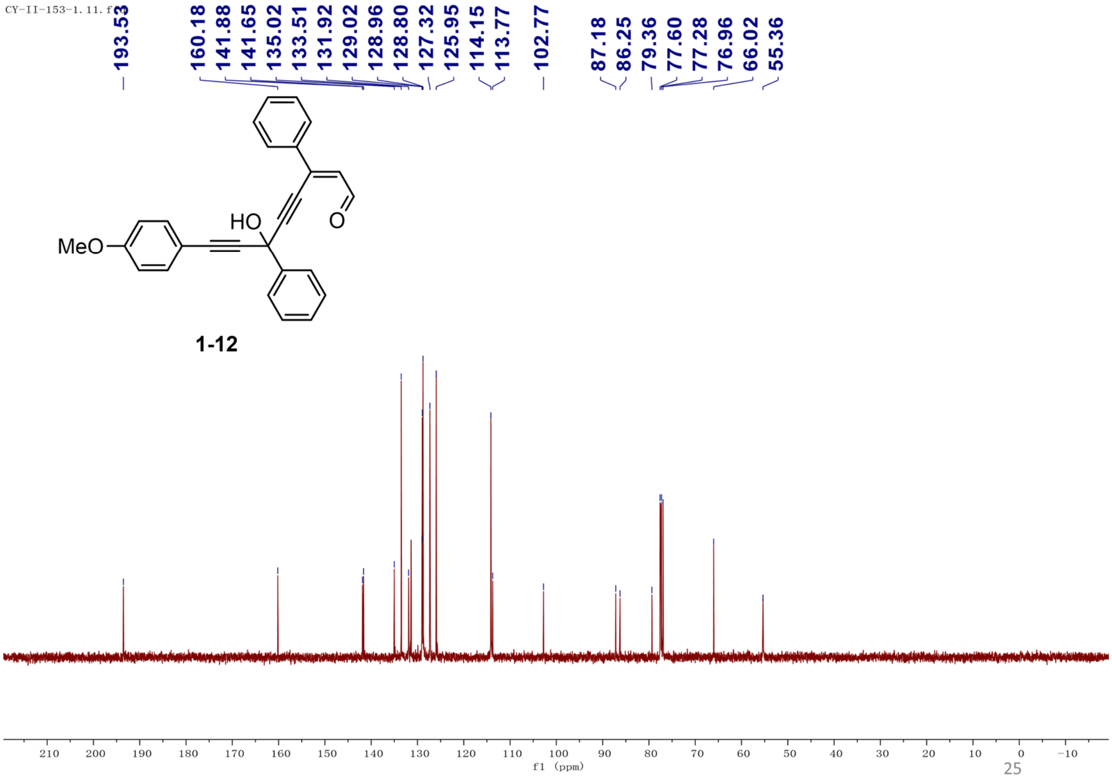

Supplementary Figure 59. ^{13}C NMR (101 MHz, $CDCl_3$) spectra for compound **1-12**

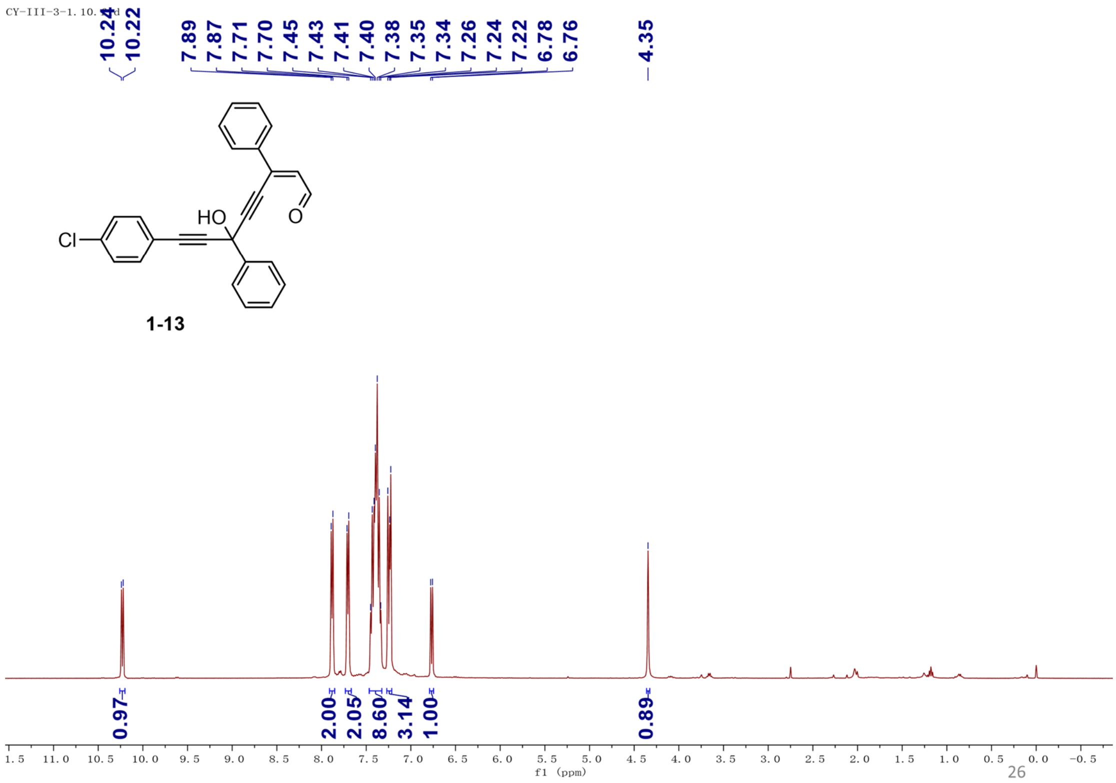

Supplementary Figure 60. ^{1}H NMR (400 MHz, $CDCl_3$) spectra for compound **1-13**

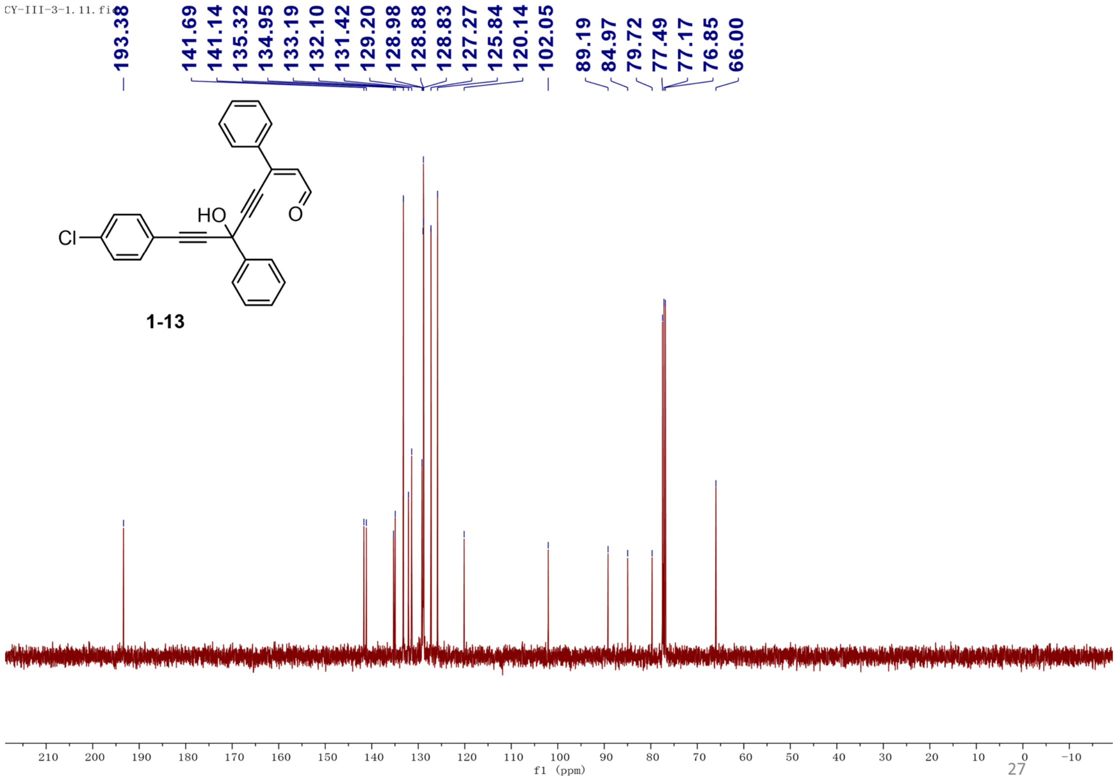

Supplementary Figure 61. ^{13}C NMR (101 MHz, $CDCl_3$) spectra for compound **1-13**

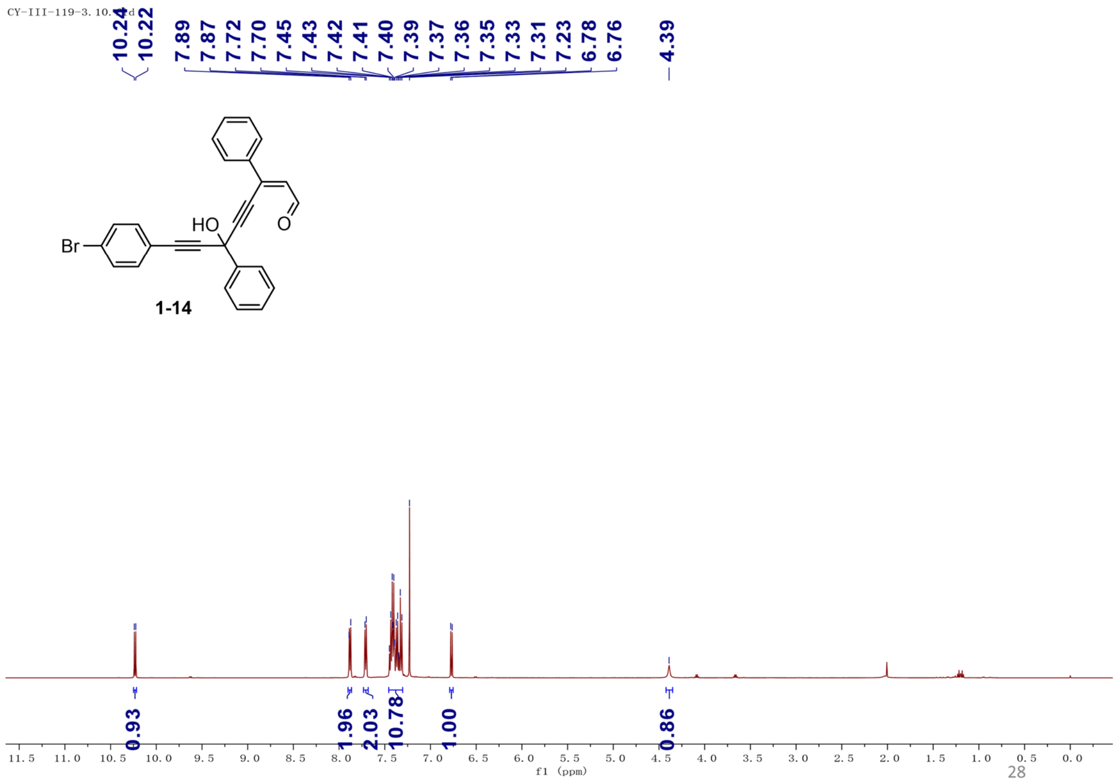

Supplementary Figure 62. ^{1}H NMR (400 MHz, $CDCl_3$) spectra for compound **1-14**

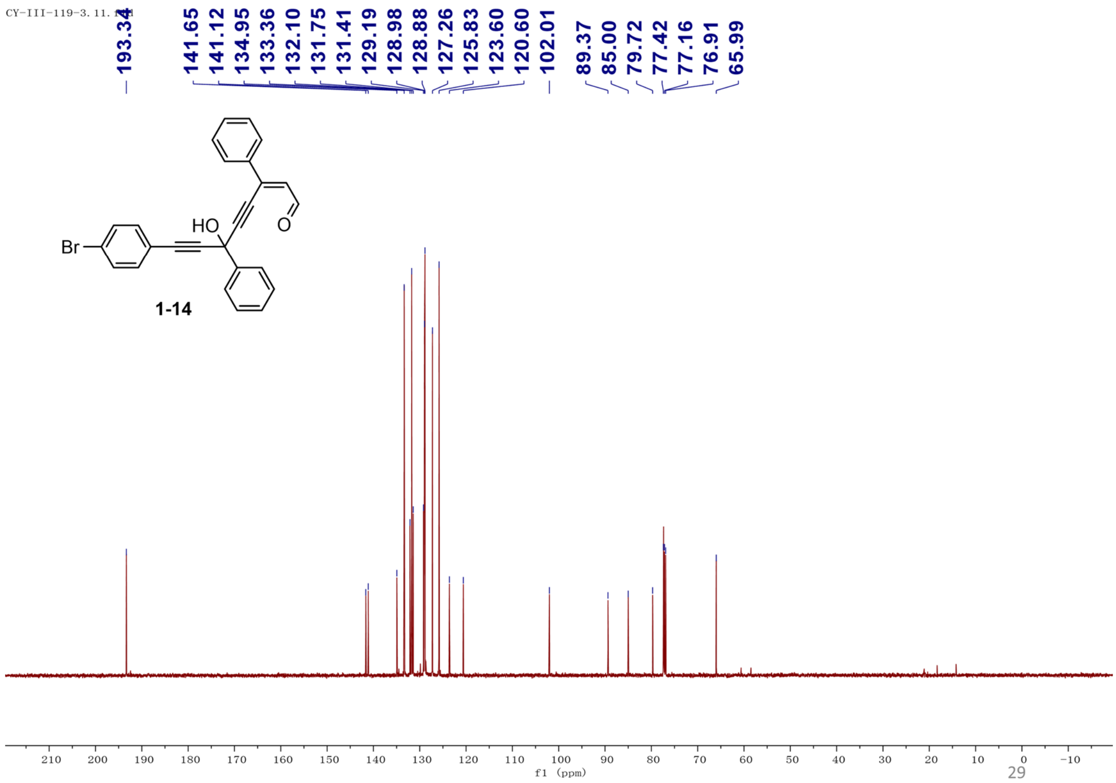

Supplementary Figure 63. ^{13}C NMR (101 MHz, $CDCl_3$) spectra for compound **1-14**

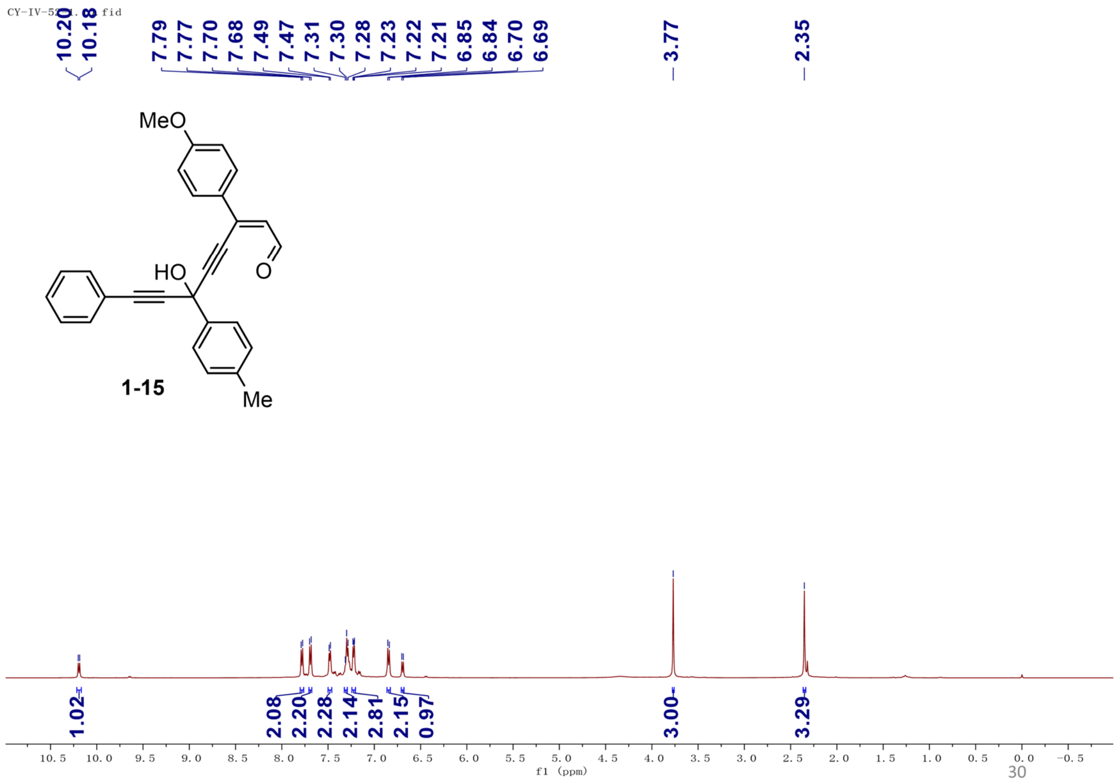

Supplementary Figure 64. ^{1}H NMR (500 MHz, $CDCl_3$) spectra for compound **1-15**

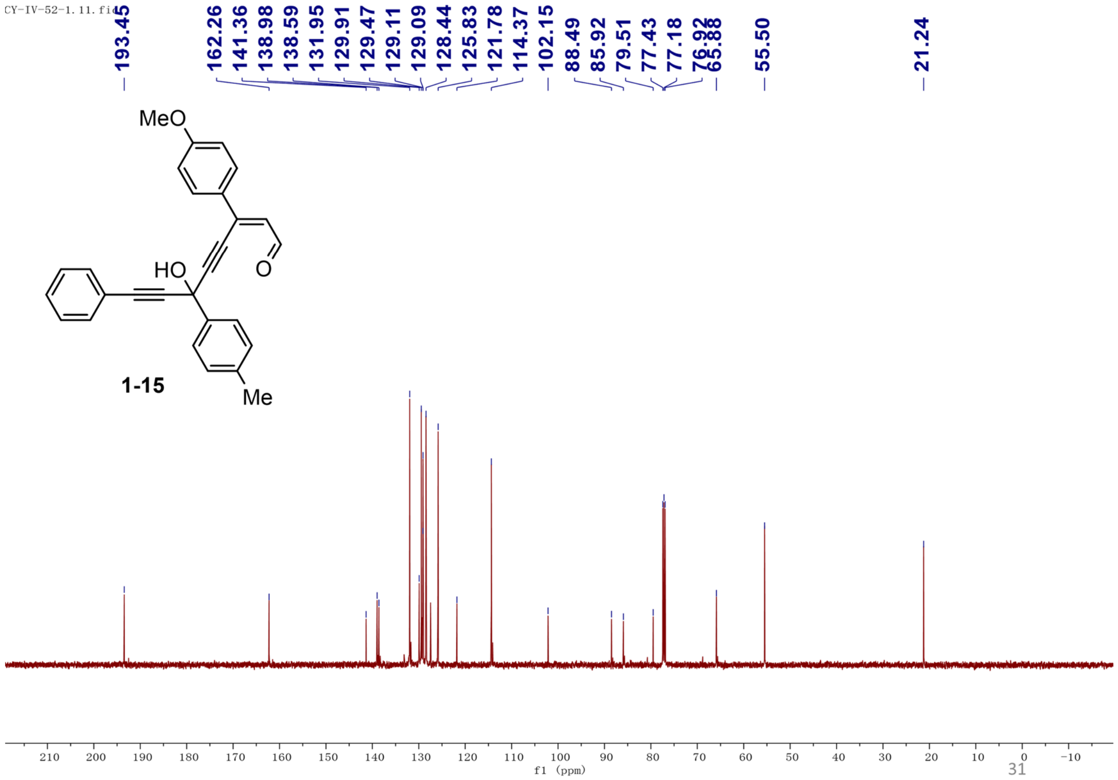

Supplementary Figure 65. ^{13}C NMR (126 MHz, $CDCl_3$) spectra for compound **1-15**

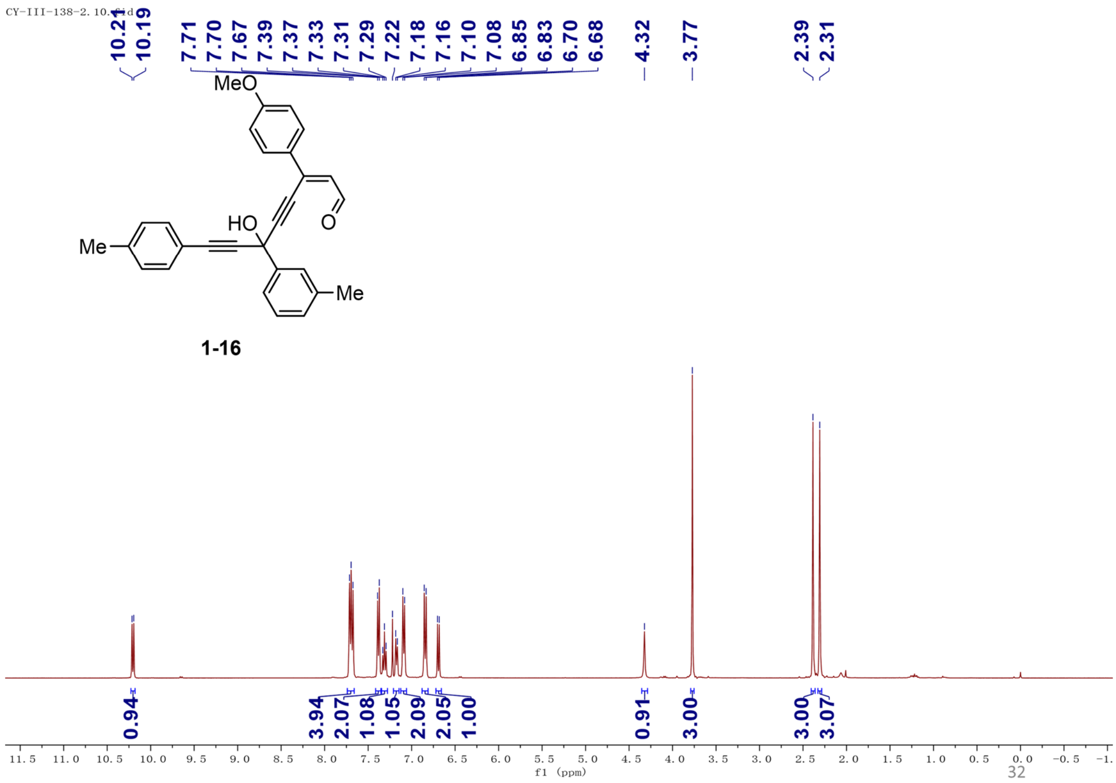

Supplementary Figure 66. ^{1}H NMR (400 MHz, $CDCl_3$) spectra for compound **1-16**

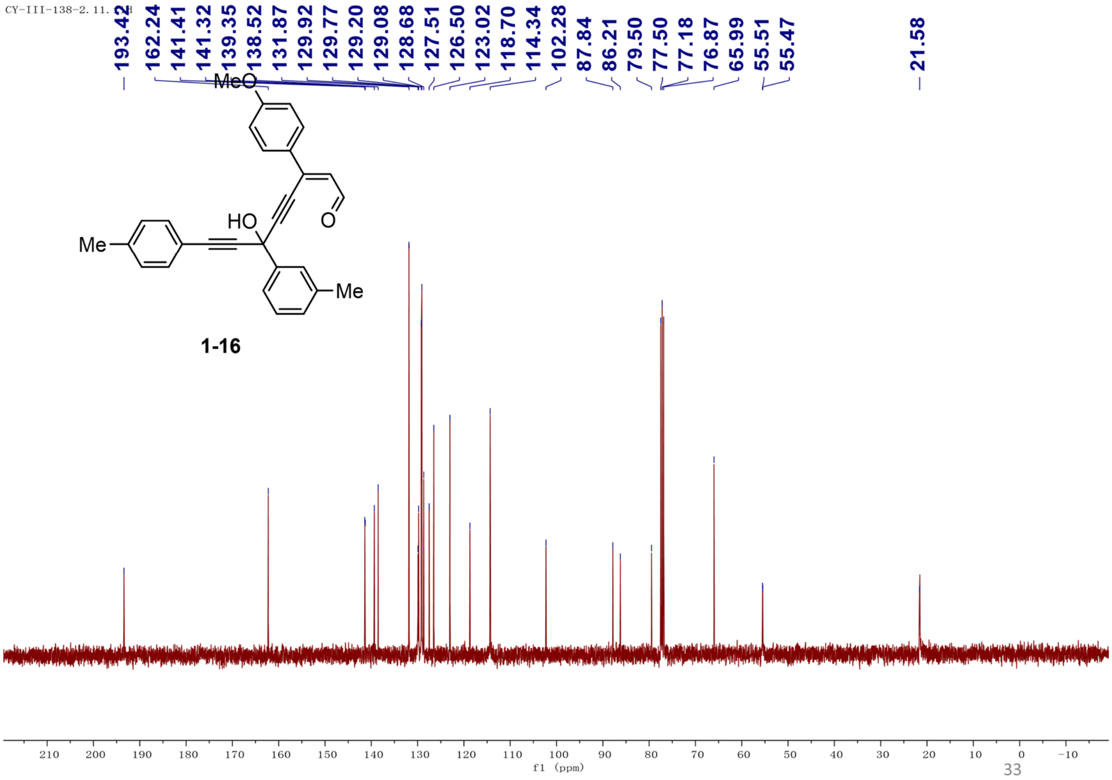

Supplementary Figure 67. ^{13}C NMR (101 MHz, $CDCl_3$) spectra for compound **1-16**

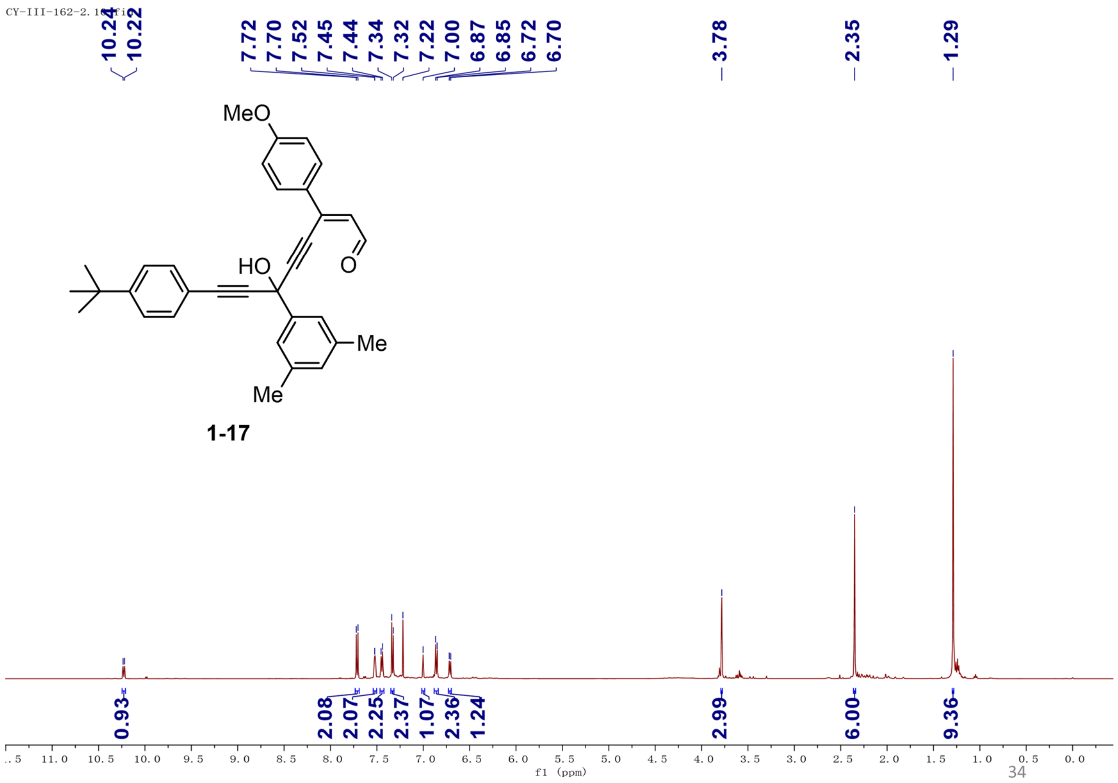

Supplementary Figure 68. ^{1}H NMR (500 MHz, $CDCl_3$) spectra for compound **1-17**

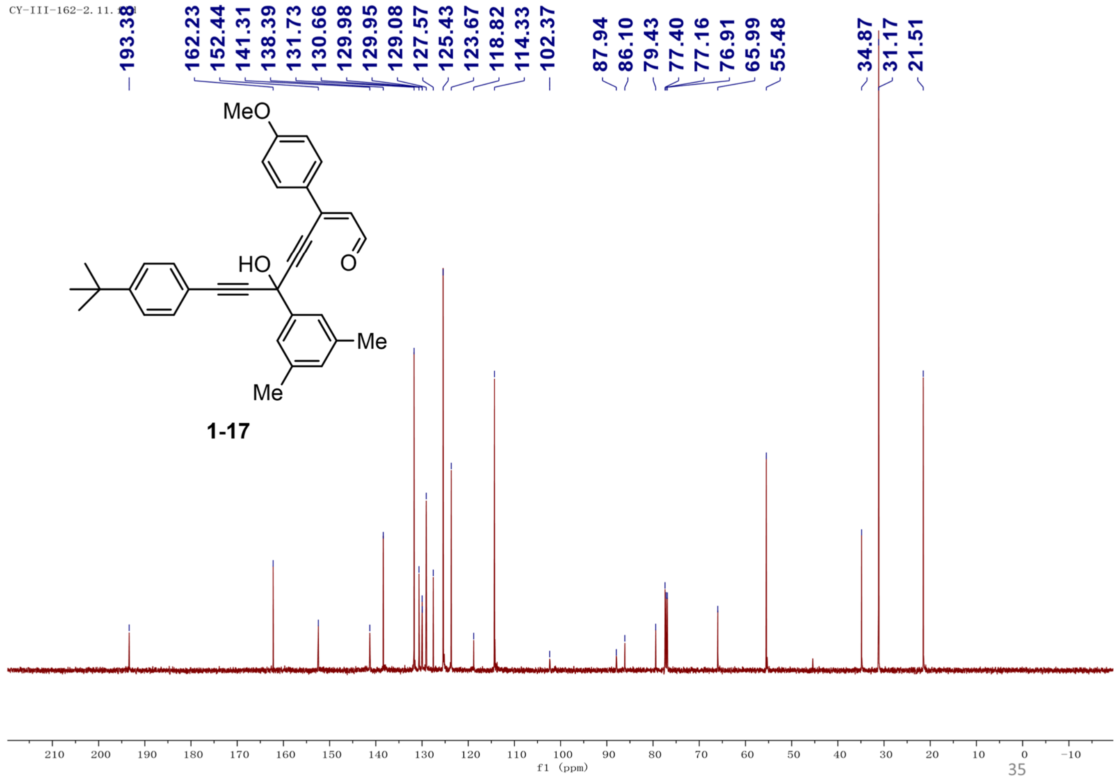

Supplementary Figure 69. ^{13}C NMR (126 MHz, $CDCl_3$) spectra for compound **1-17**

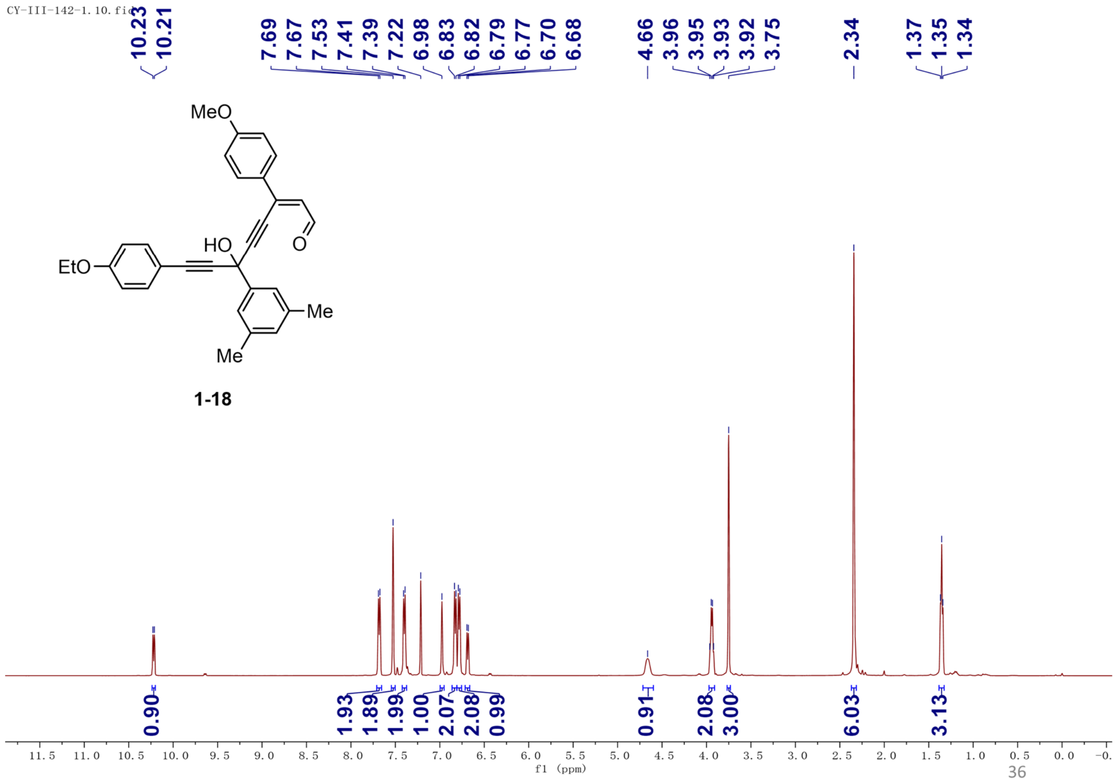

Supplementary Figure 70. ^{1}H NMR (500 MHz, $CDCl_3$) spectra for compound **1-18**

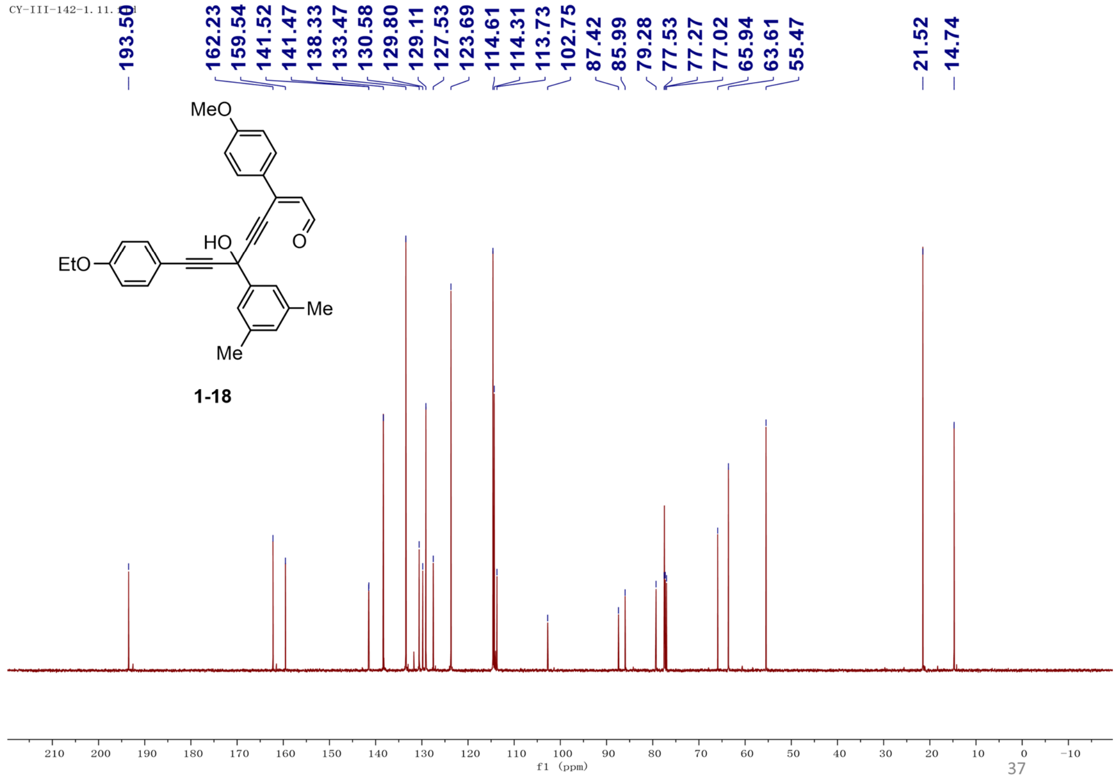

Supplementary Figure 71. ^{13}C NMR (126 MHz, $CDCl_3$) spectra for compound **1-18**

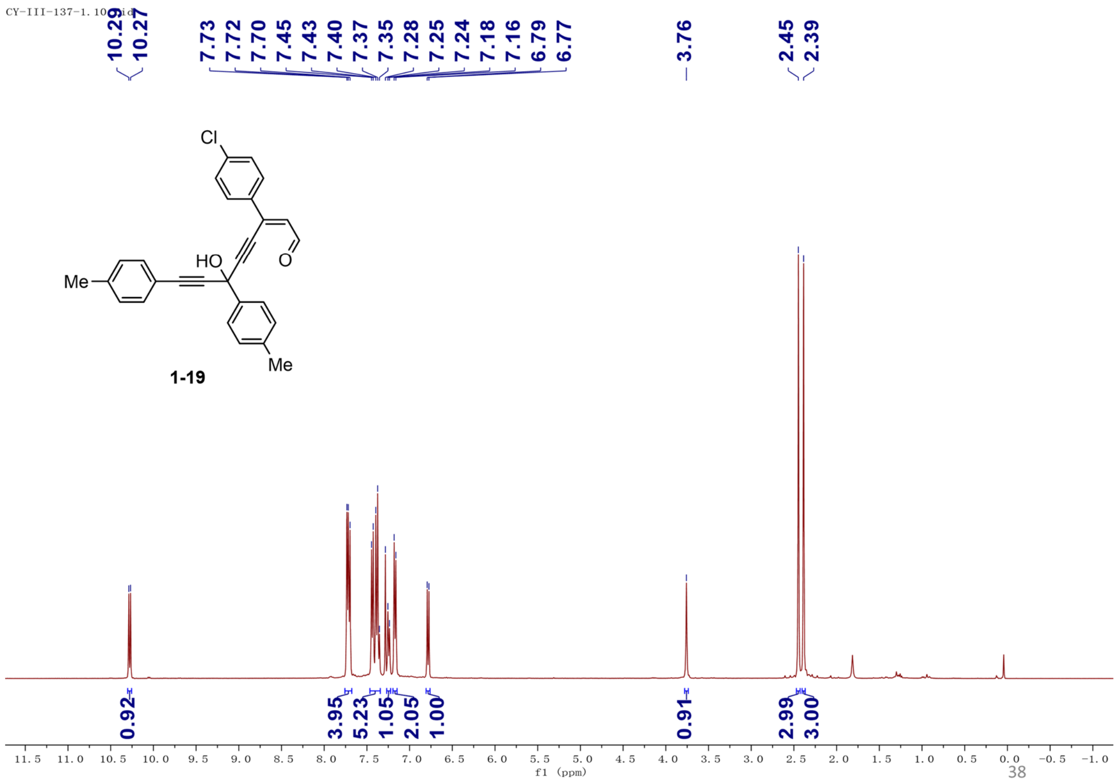

Supplementary Figure 72. ^{1}H NMR (400 MHz, $CDCl_3$) spectra for compound **1-19**

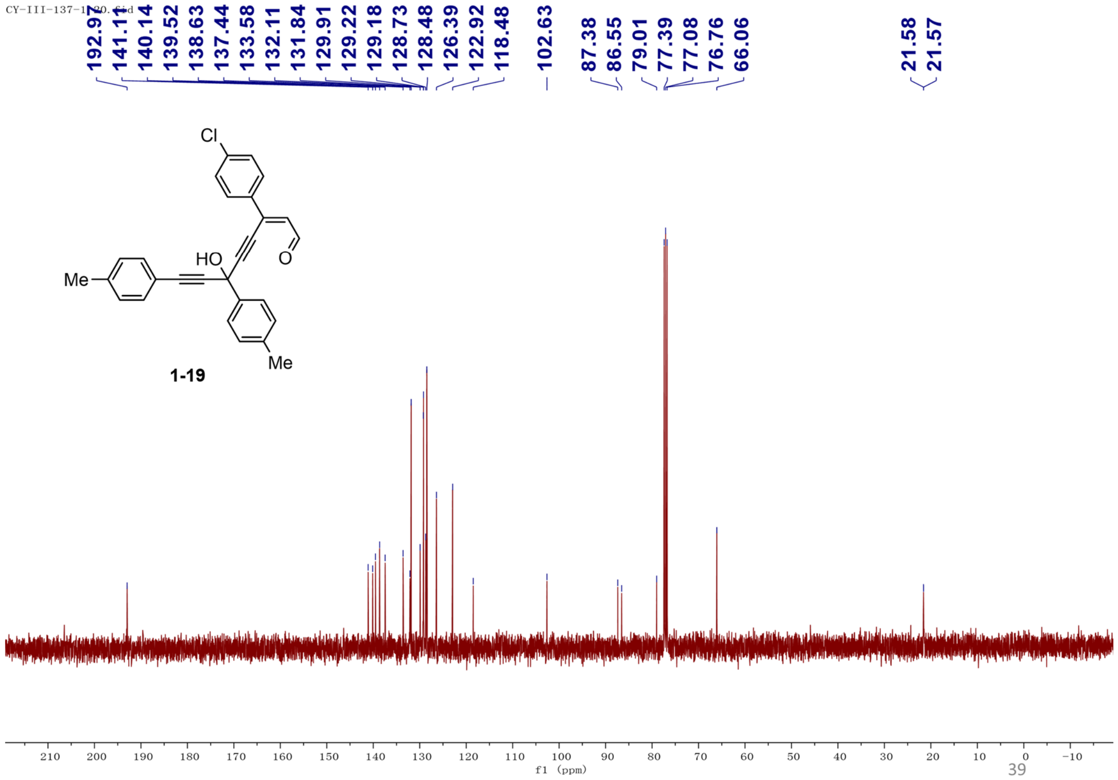

Supplementary Figure 73. ^{13}C NMR (101 MHz, $CDCl_3$) spectra for compound **1-19**

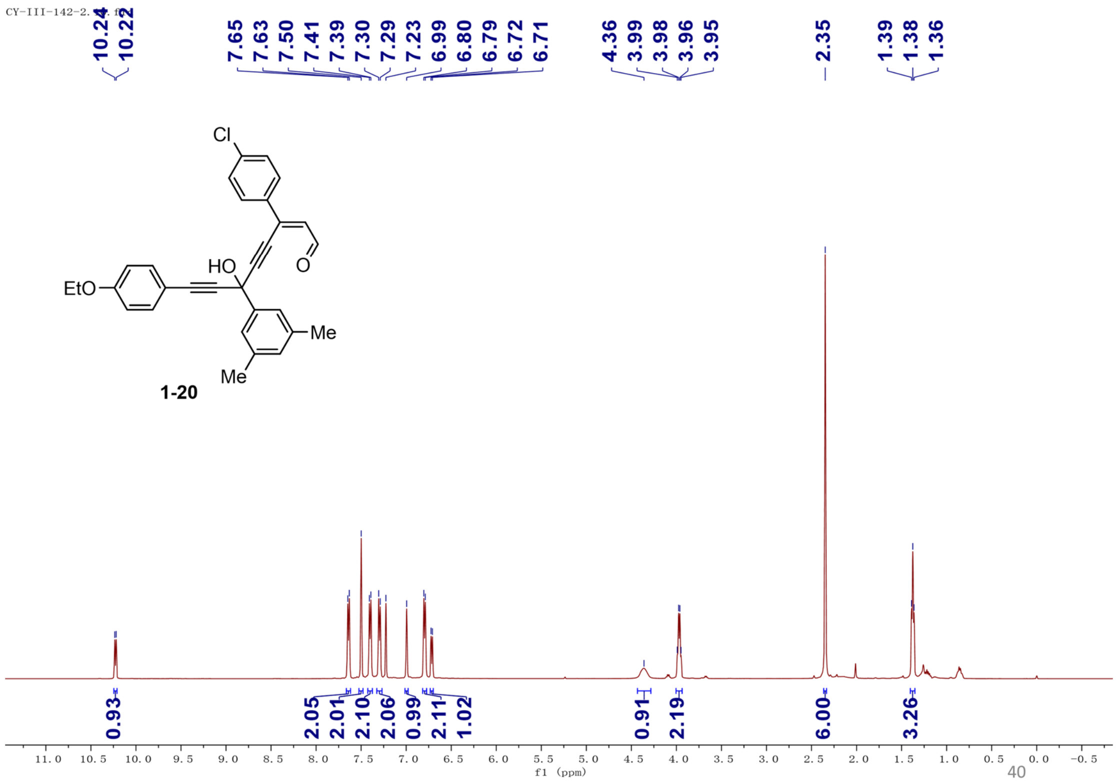

Supplementary Figure 74. ^{1}H NMR (500 MHz, $CDCl_3$) spectra for compound **1-20**

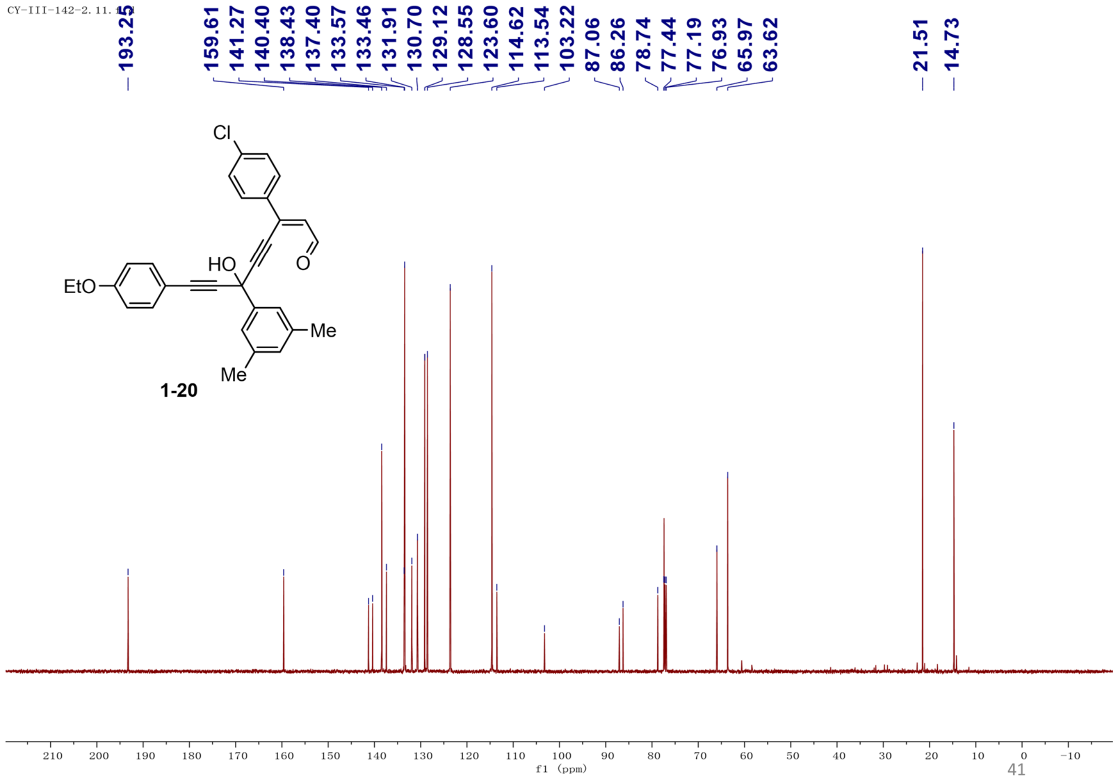

Supplementary Figure 75. ^{13}C NMR (126 MHz, $CDCl_3$) spectra for compound **1-20**

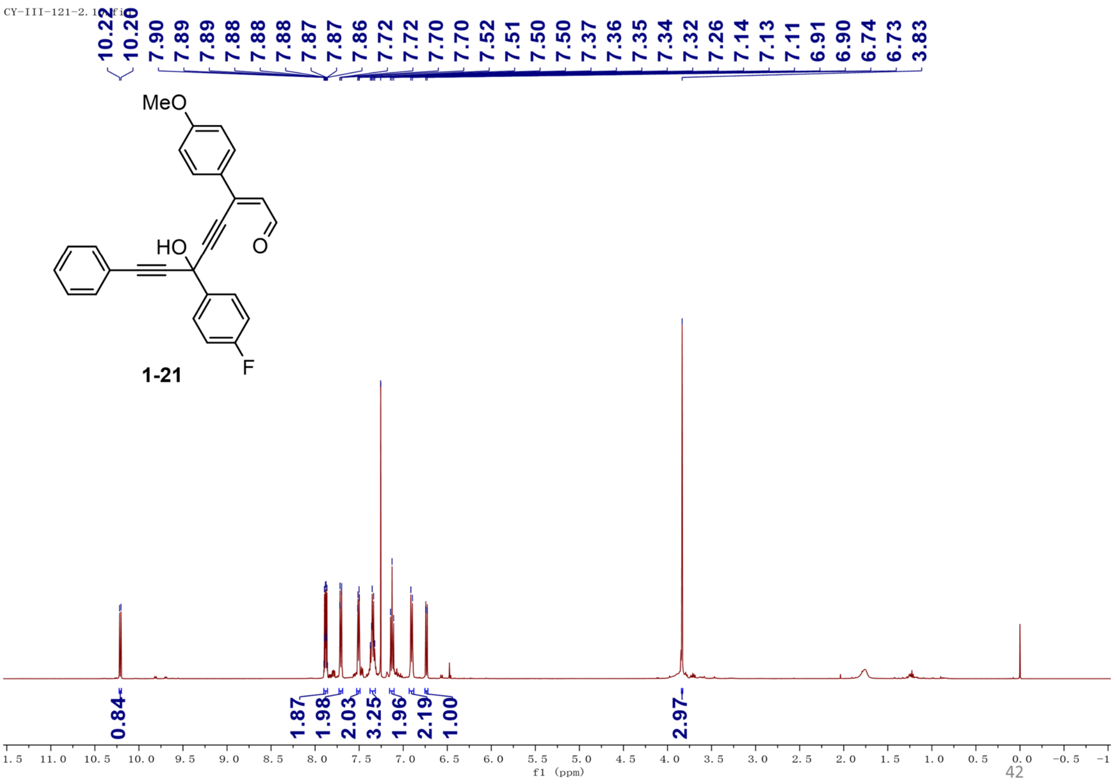

Supplementary Figure 76. ^{1}H NMR (500 MHz, CDCl$_3$) spectra for compound **1-21**

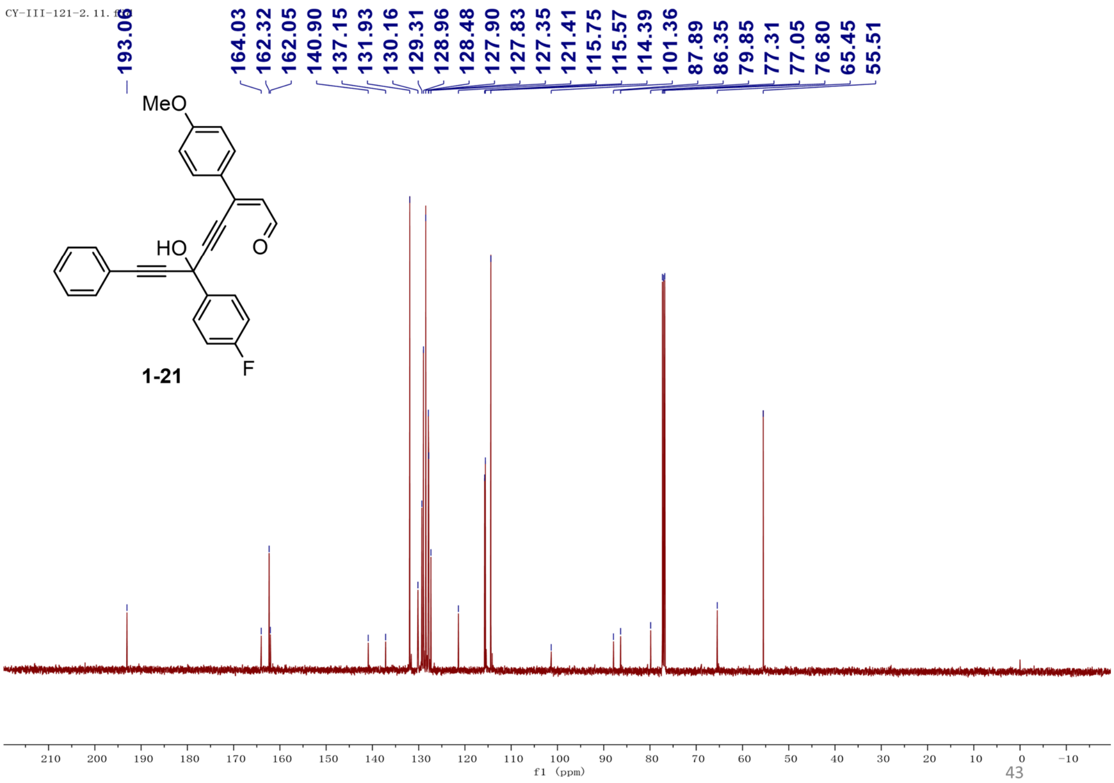

Supplementary Figure 77. ^{13}C NMR (126 MHz, CDCl$_3$) spectra for compound **1-21**

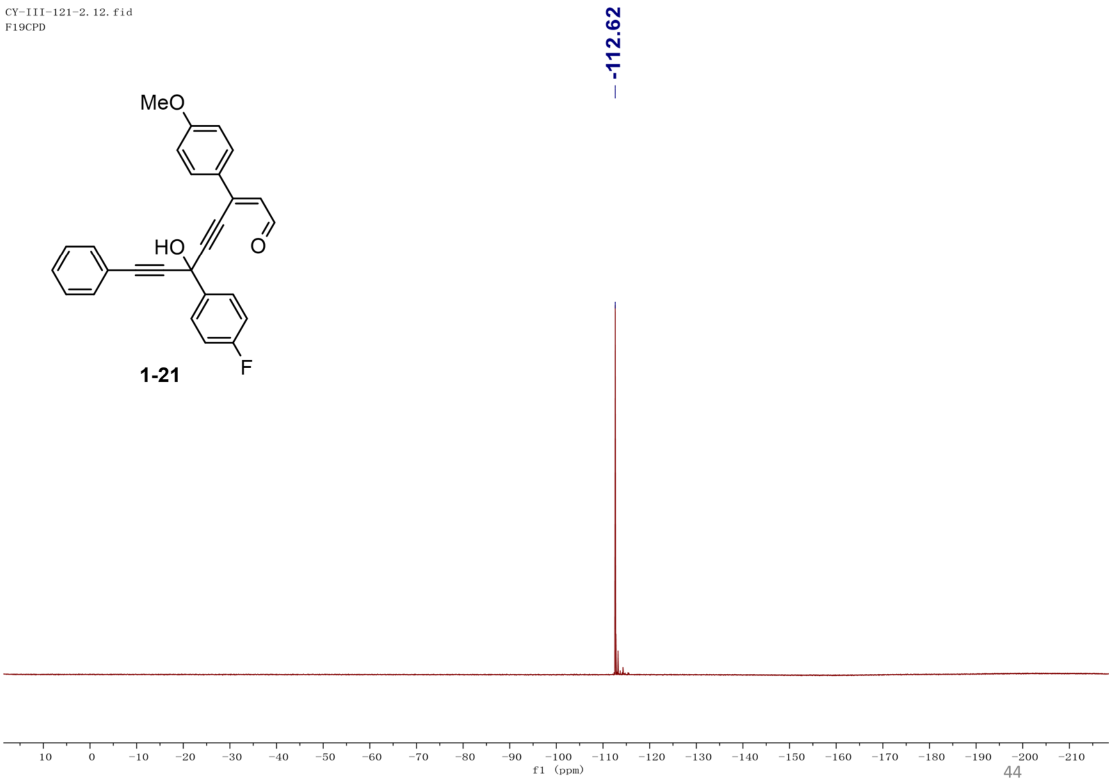

Supplementary Figure 78. ^{19}F NMR (471 MHz, $CDCl_3$) spectra for compound **1-21**

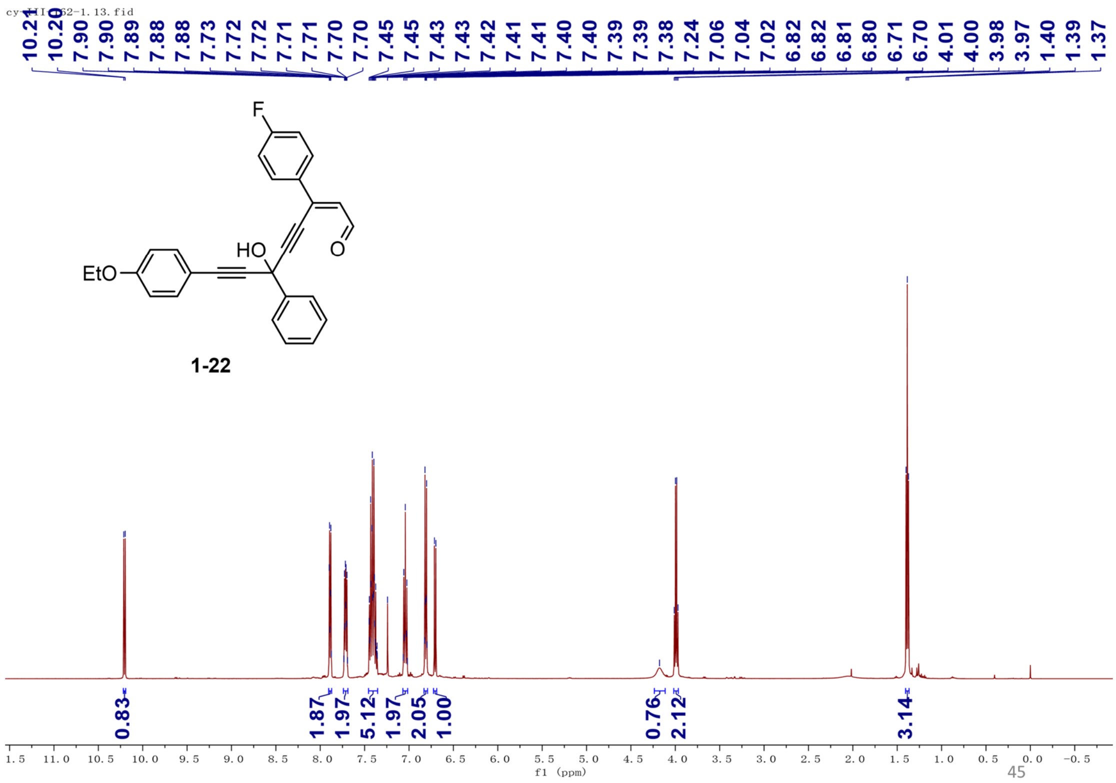

Supplementary Figure 79. ^{1}H NMR (500 MHz, $CDCl_3$) spectra for compound **1-22**

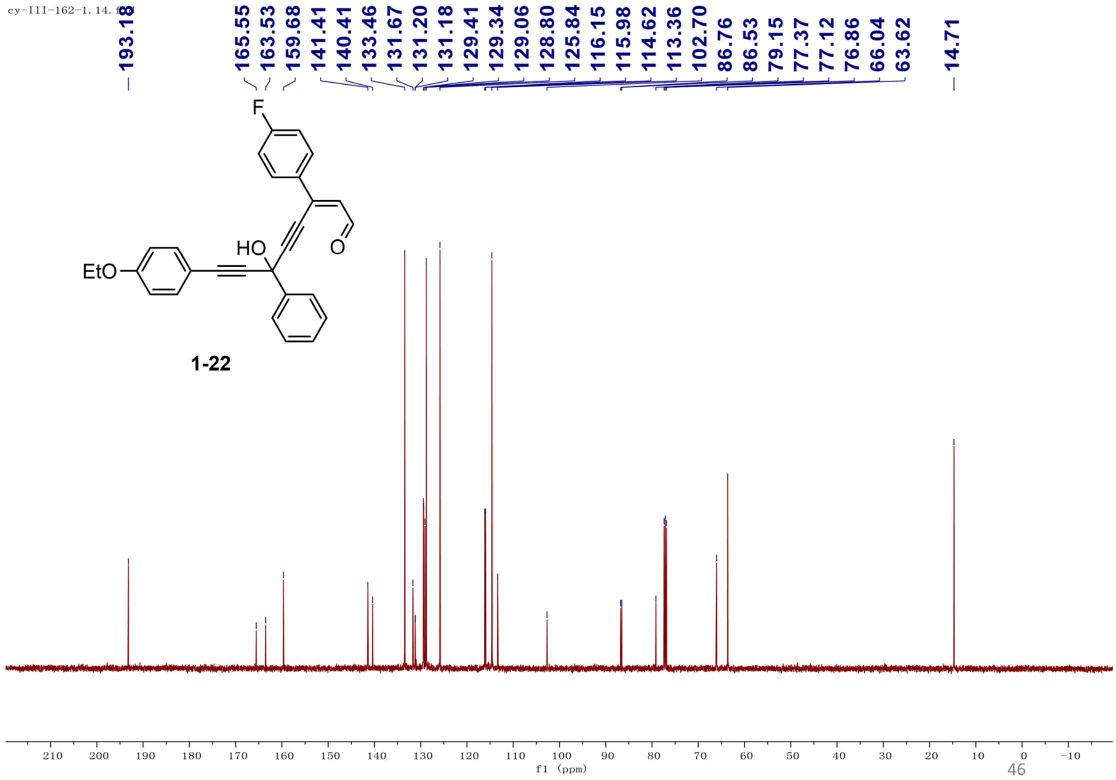

Supplementary Figure 80. ^{13}C NMR (126 MHz, $CDCl_3$) spectra for compound **1-22**

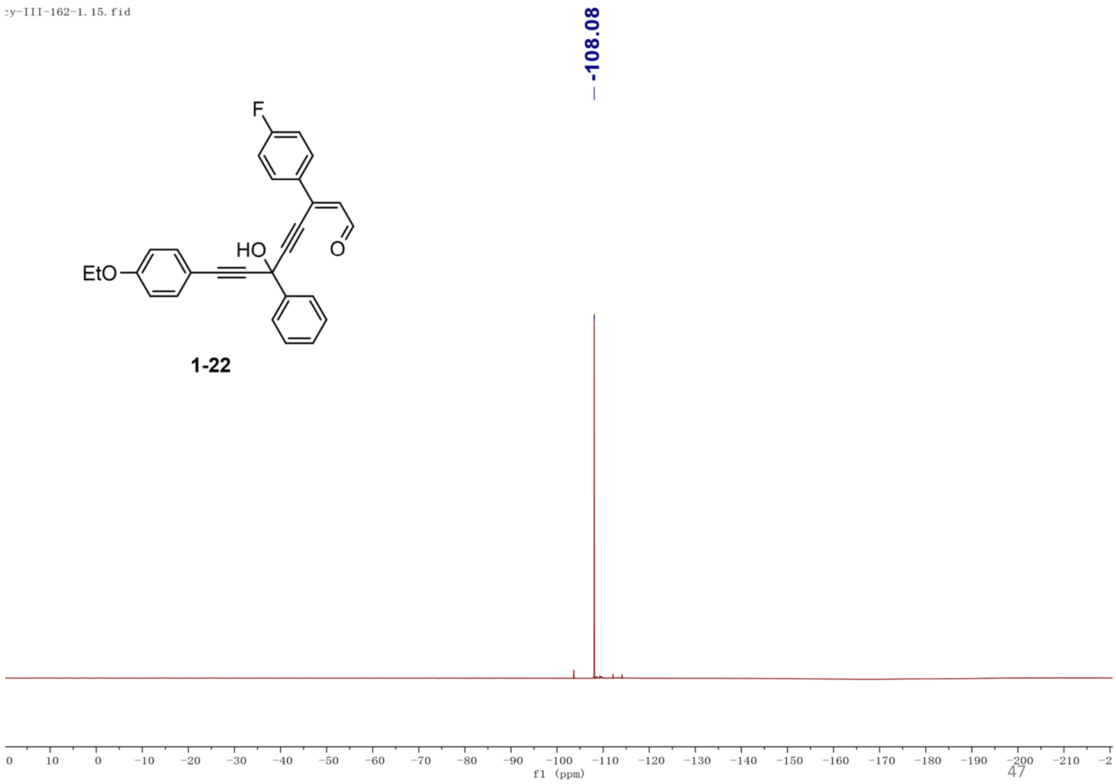

Supplementary Figure 81. ^{19}F NMR (471 MHz, $CDCl_3$) spectra for compound **1-22**

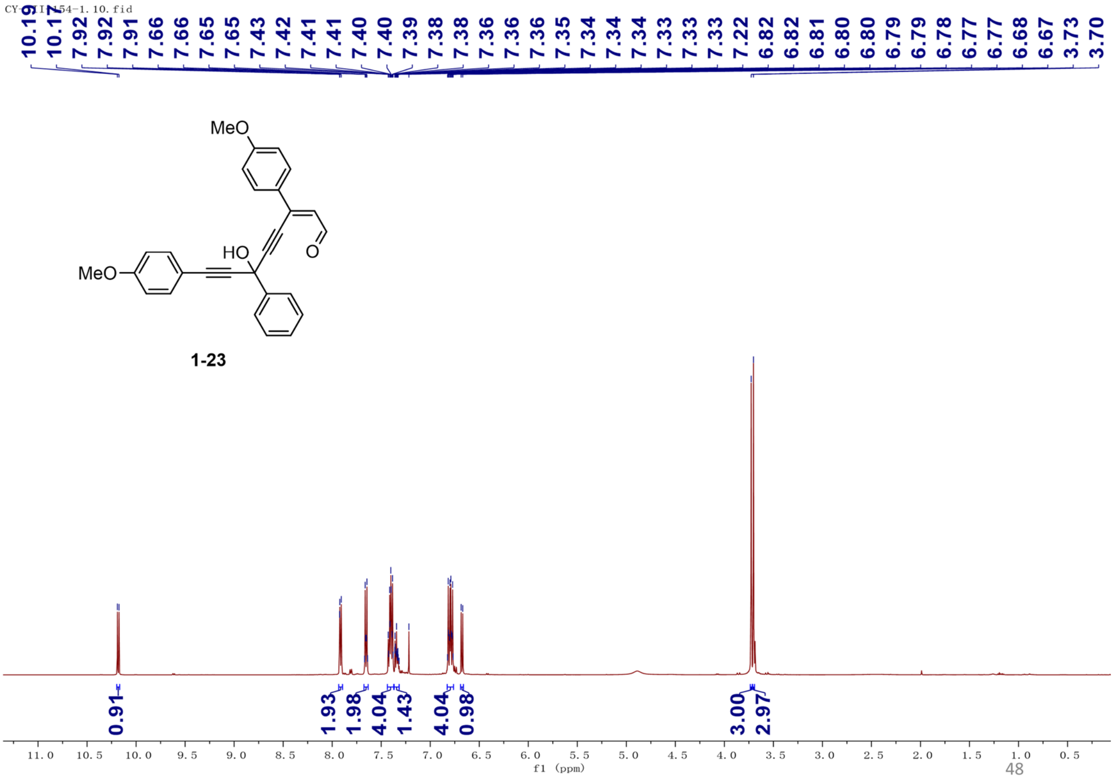

Supplementary Figure 82. ^{1}H NMR (500 MHz, $CDCl_3$) spectra for compound **1-23**

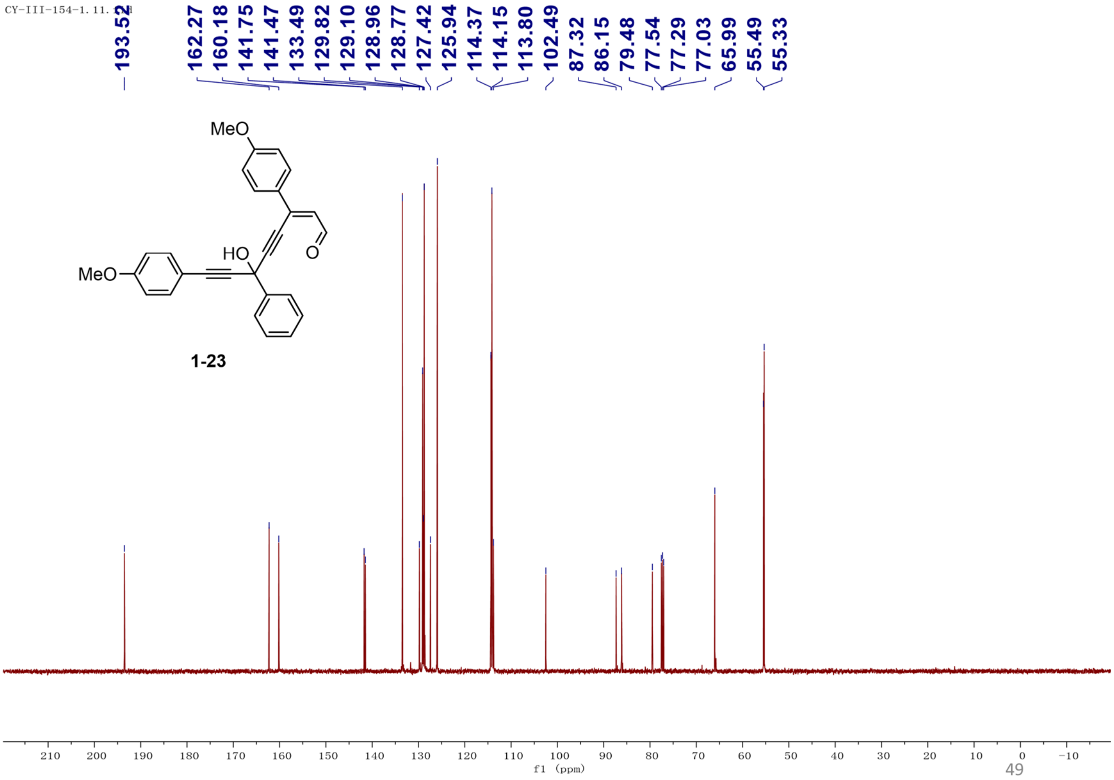

Supplementary Figure 83. ^{13}C NMR (126 MHz, $CDCl_3$) spectra for compound **1-23**

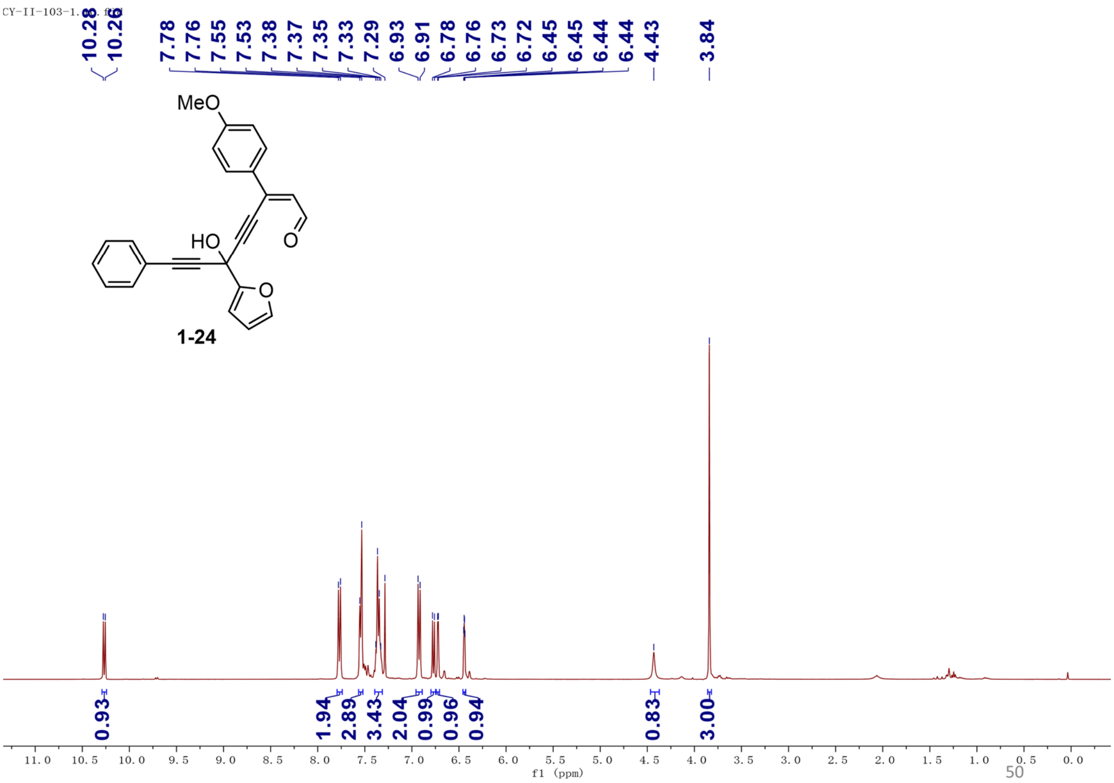

Supplementary Figure 84. ^{1}H NMR (400 MHz, $CDCl_3$) spectra for compound **1-24**

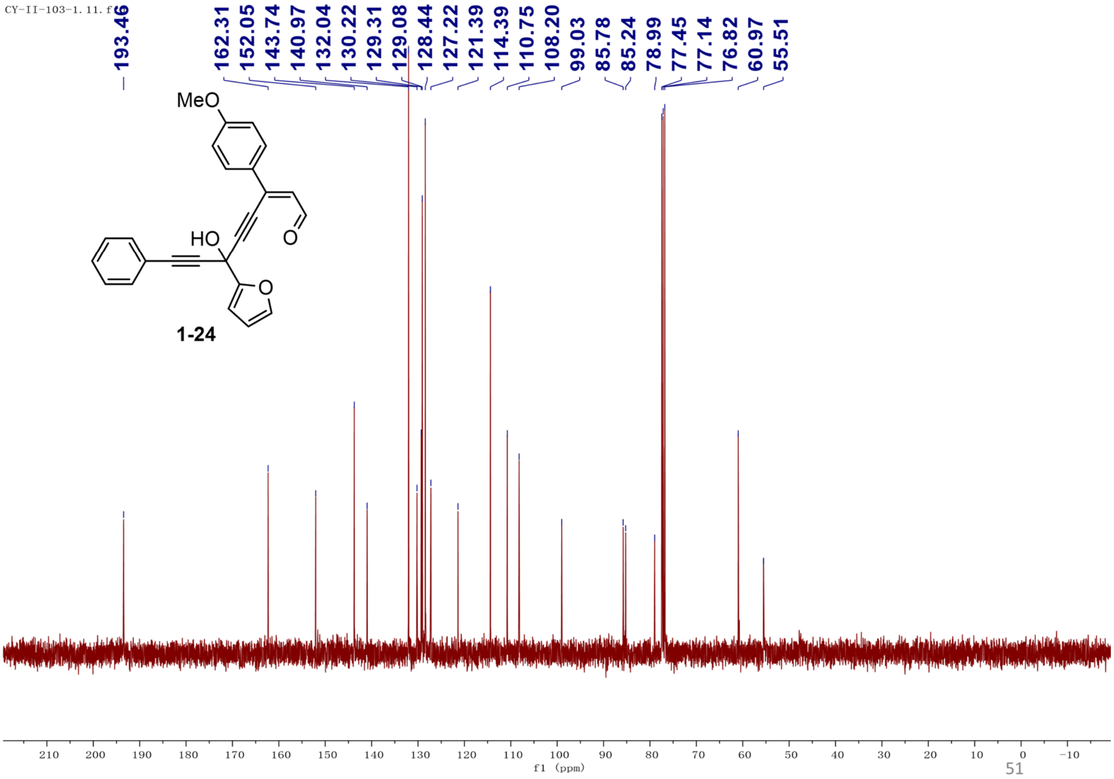

Supplementary Figure 85. ^{13}C NMR (101 MHz, $CDCl_3$) spectra for compound **1-24**

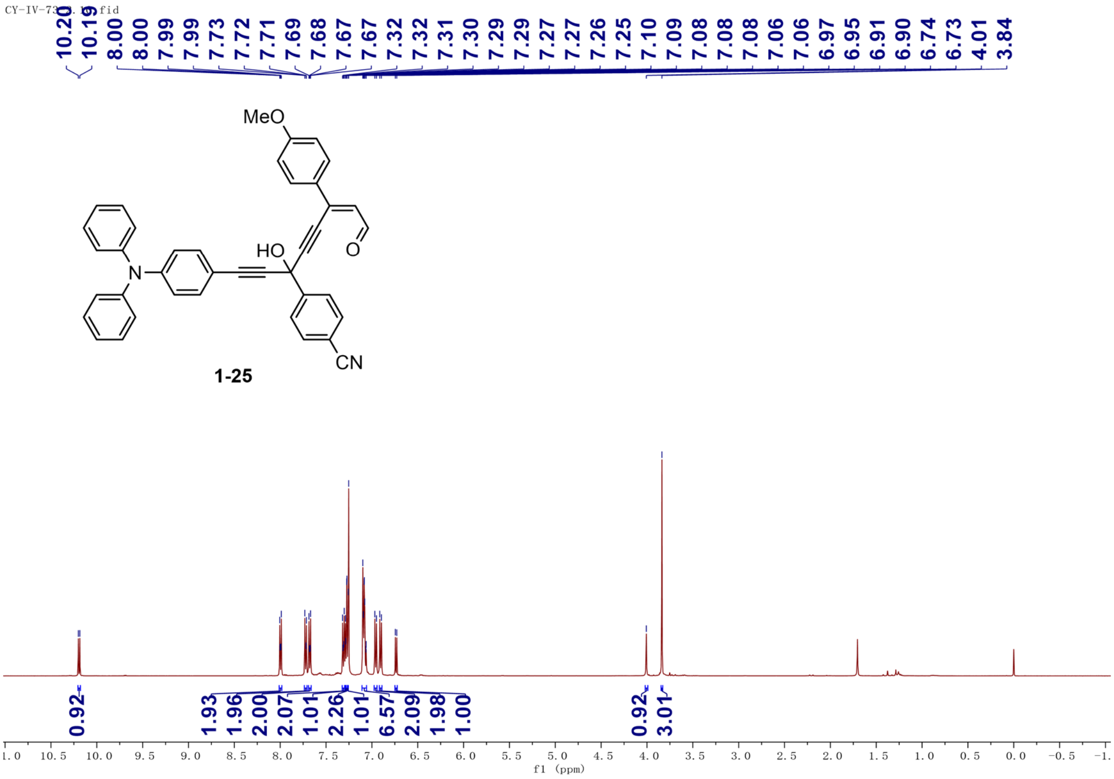

Supplementary Figure 86. ^{1}H NMR (500 MHz, $CDCl_3$) spectra for compound **1-25**

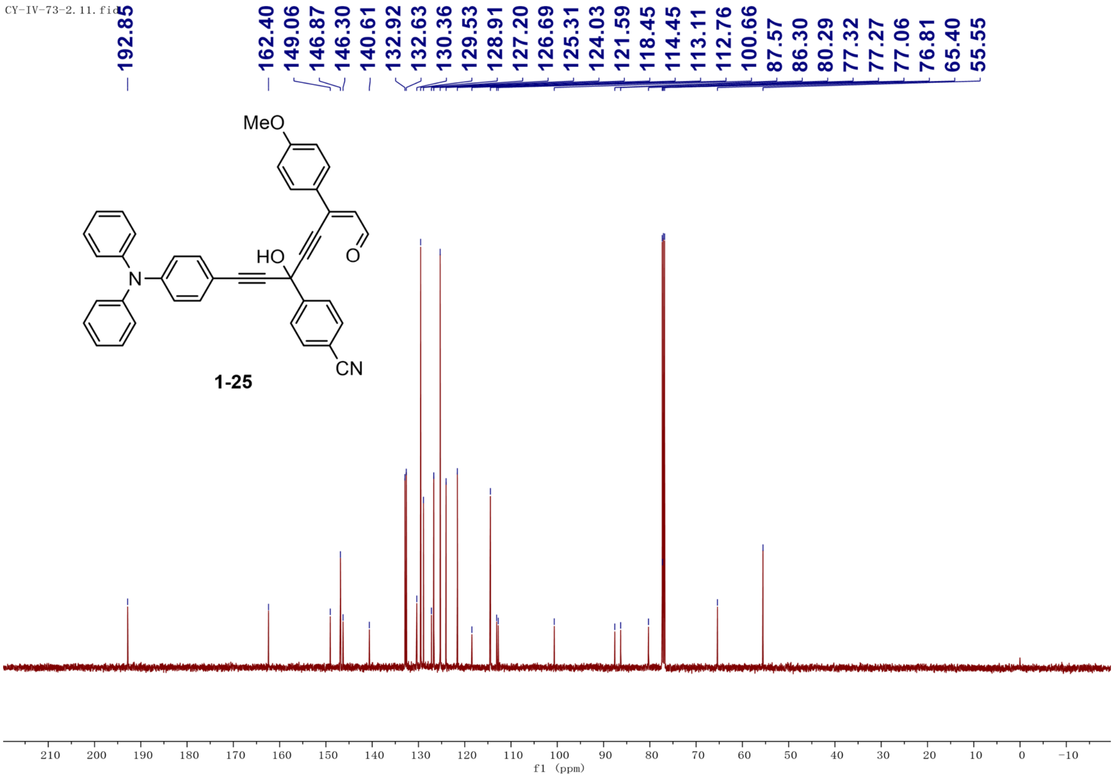

Supplementary Figure 87. ^{13}C NMR (126 MHz, $CDCl_3$) spectra for compound **1-25**

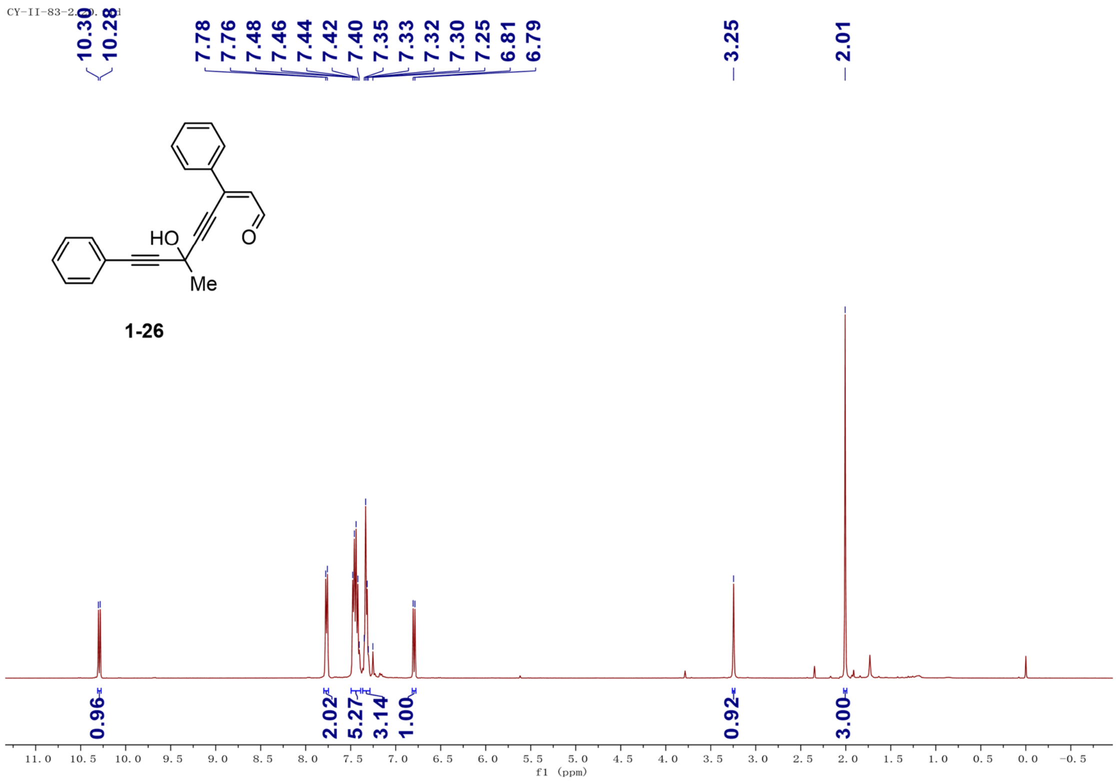

Supplementary Figure 88. ^{1}H NMR (400 MHz, $CDCl_3$) spectra for compound **1-26**

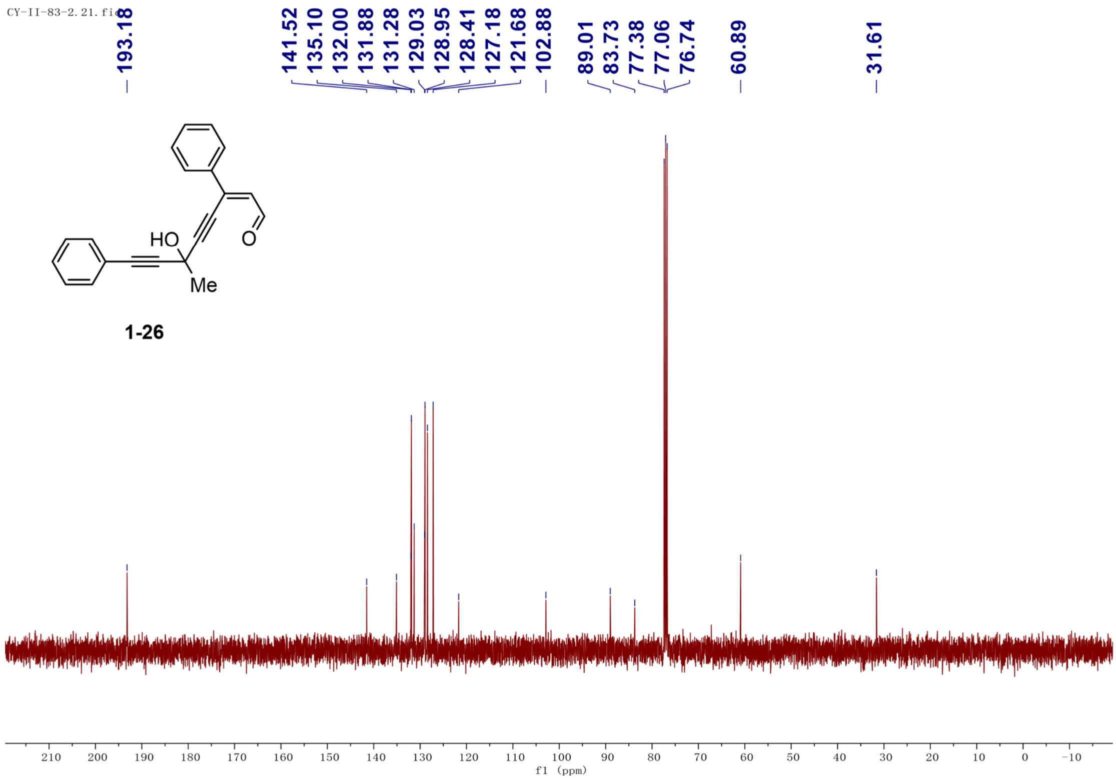

Supplementary Figure 89. ^{13}C NMR (101 MHz, $CDCl_3$) spectra for compound **1-26**

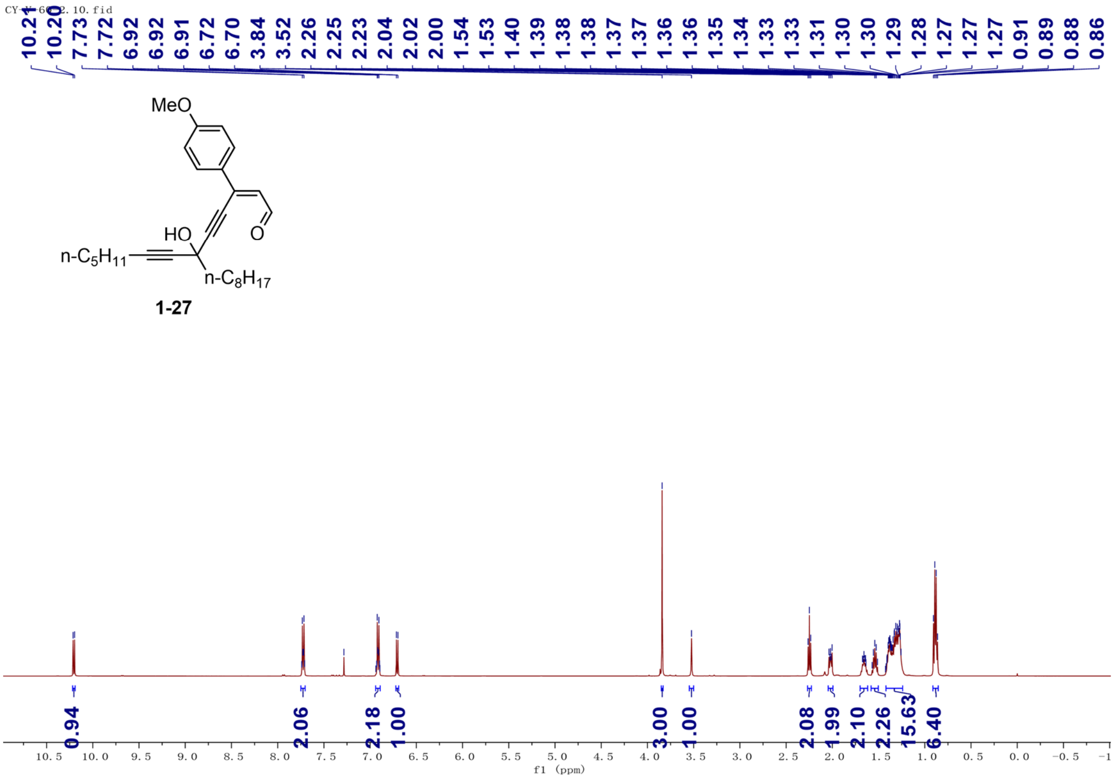

Supplementary Figure 90. ^{1}H NMR (500 MHz, $CDCl_3$) spectra for compound **1-27**

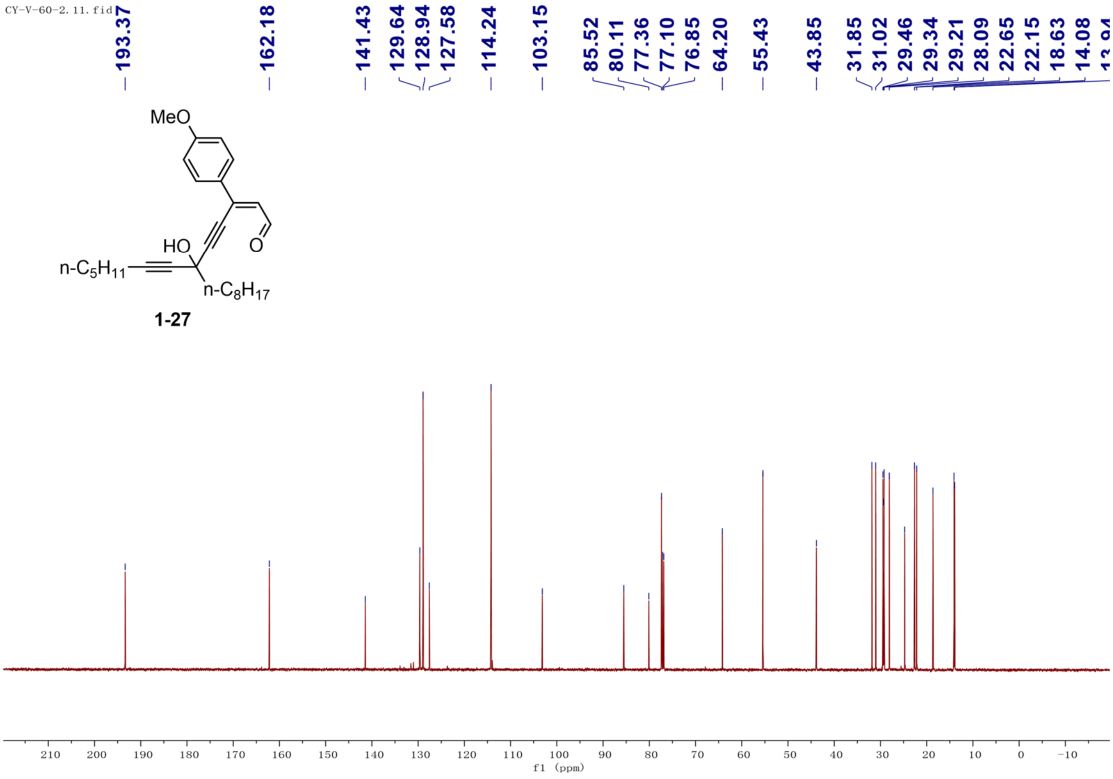

Supplementary Figure 91. ^{13}C NMR (126 MHz, $CDCl_3$) spectra for compound **1-27**

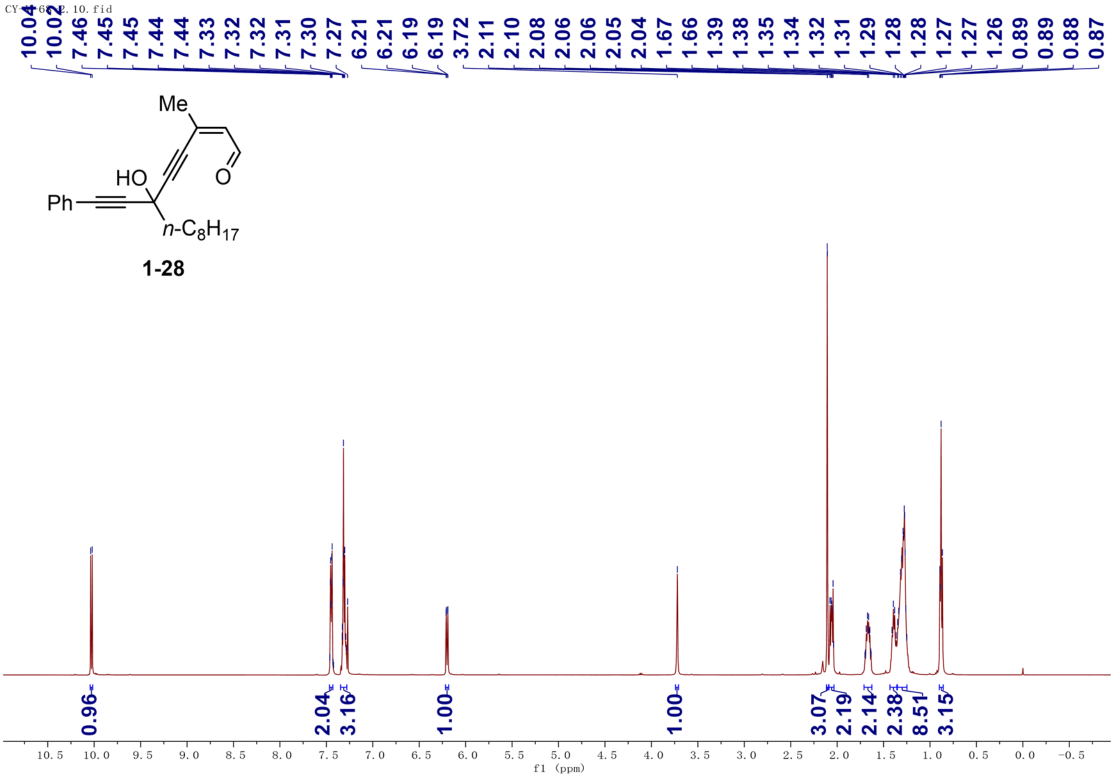

Supplementary Figure 92. ^{1}H NMR (500 MHz, $CDCl_3$) spectra for compound **1-28**

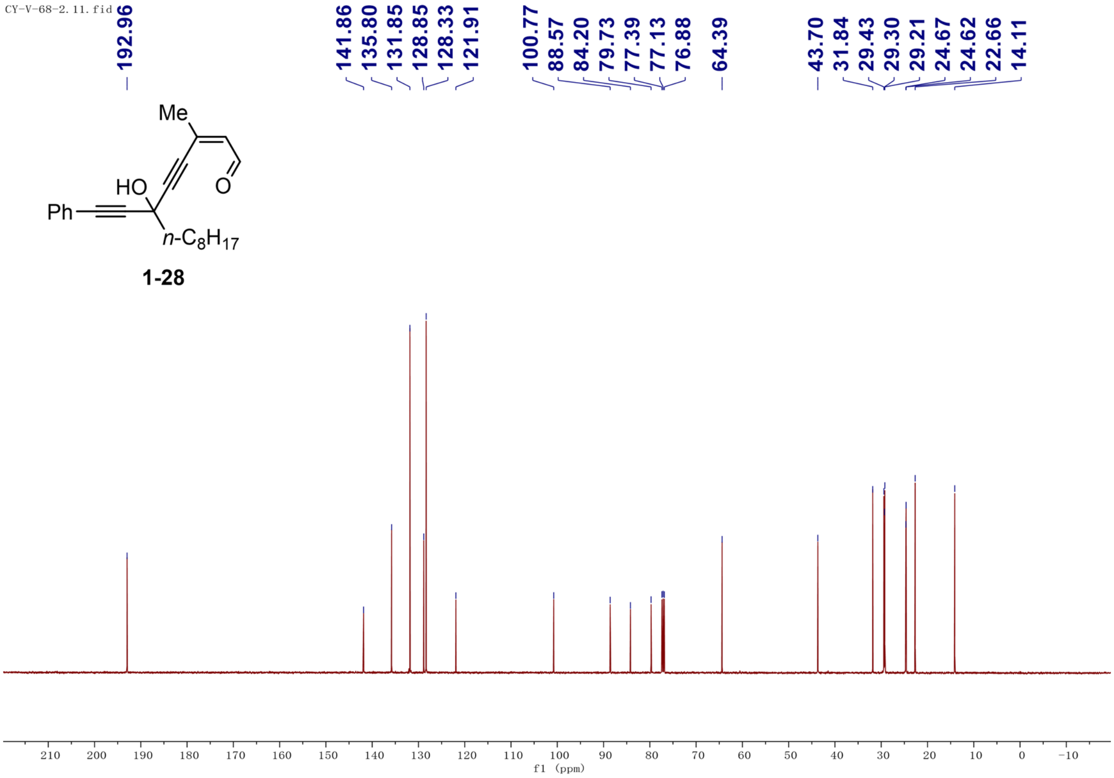

Supplementary Figure 93. ^{13}C NMR (126 MHz, $CDCl_3$) spectra for compound **1-28**

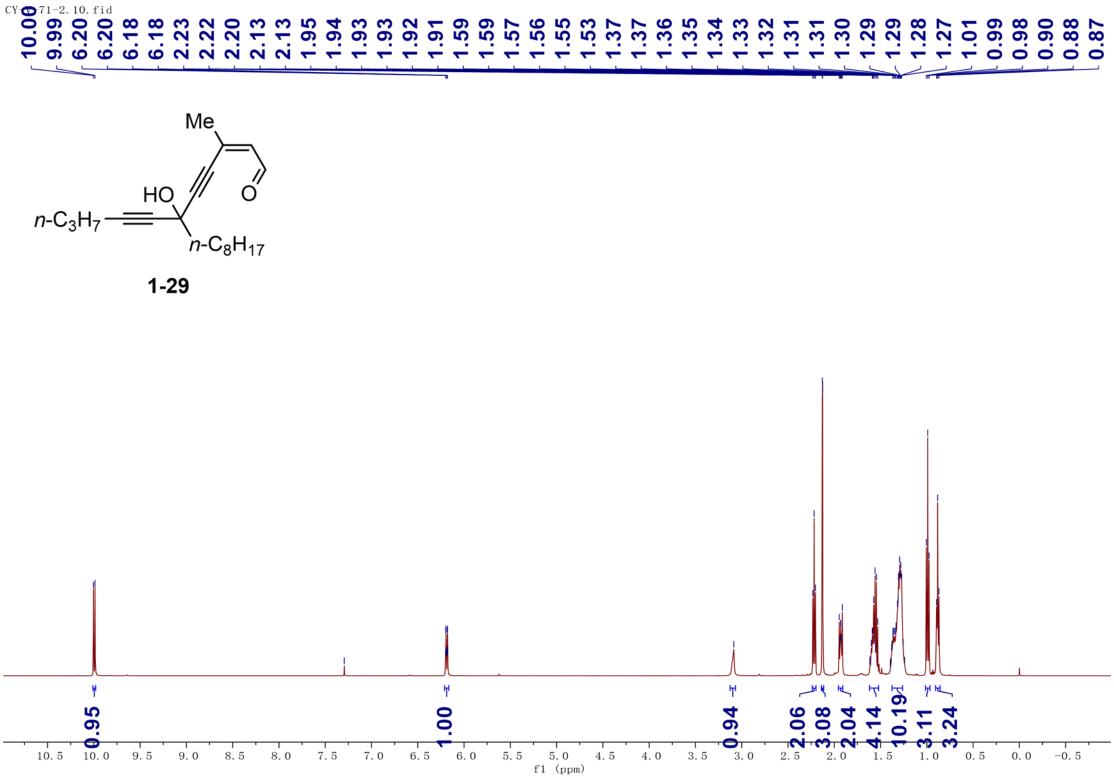

Supplementary Figure 94. ^{1}H NMR (500 MHz, $CDCl_3$) spectra for compound **1-29**

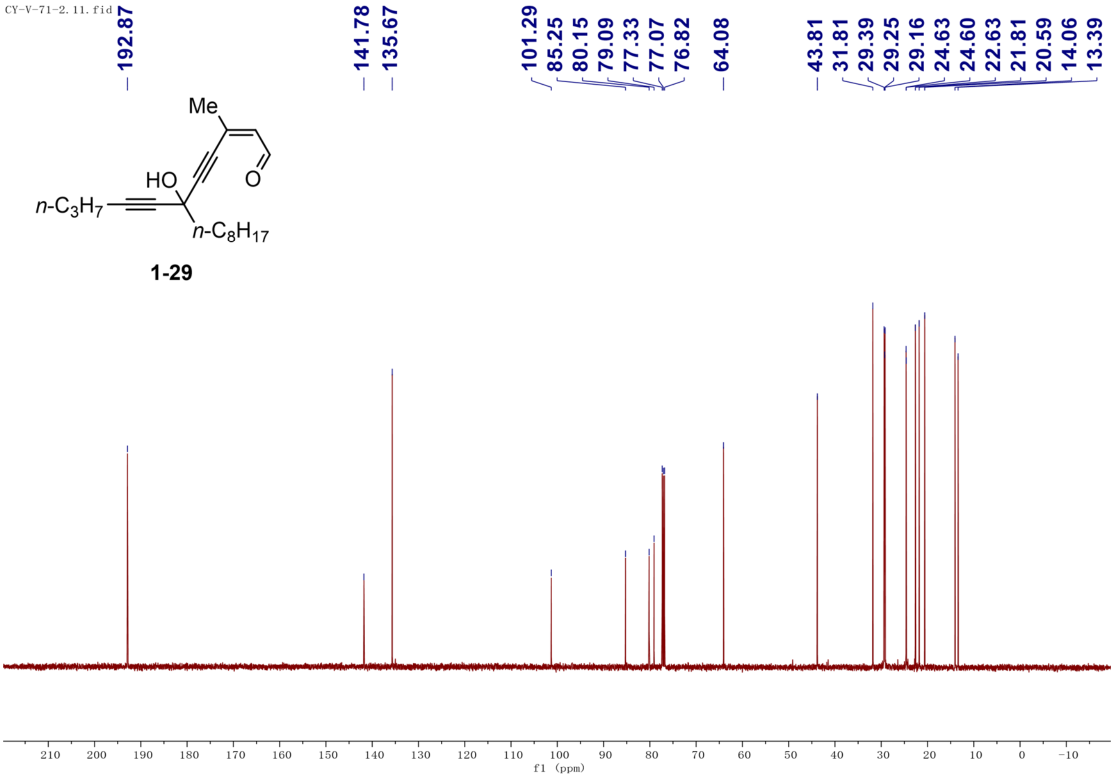

Supplementary Figure 95. ^{13}C NMR (126 MHz, $CDCl_3$) spectra for compound **1-29**

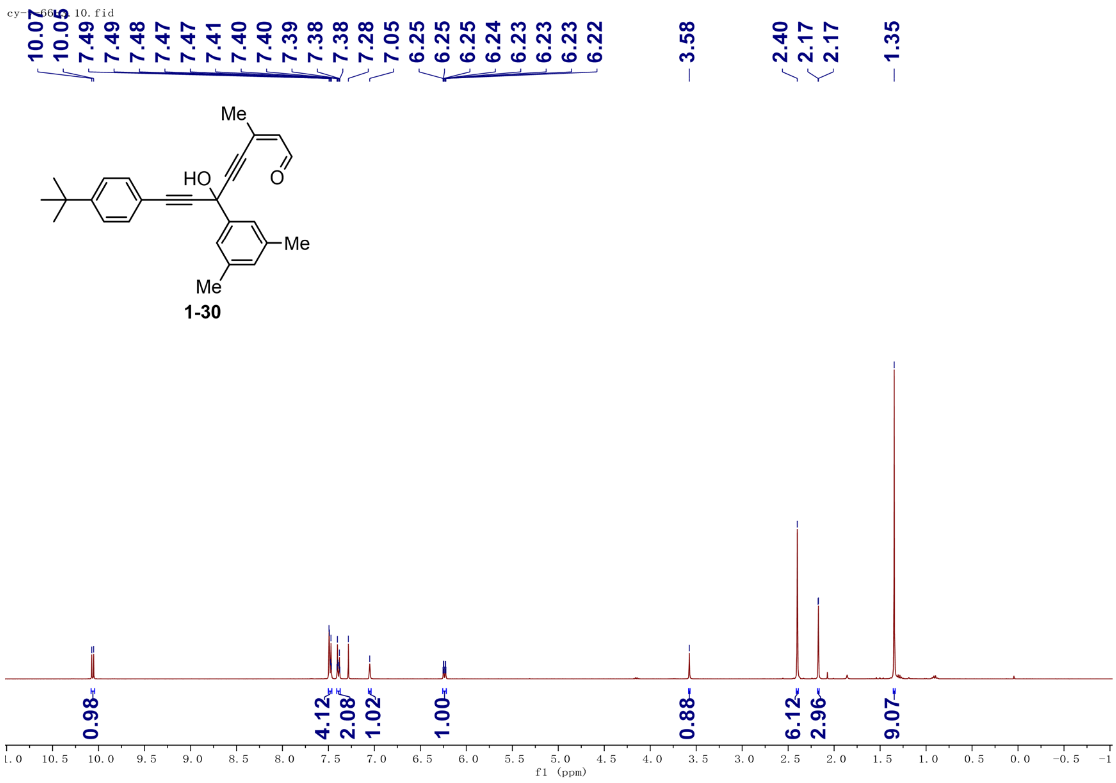

Supplementary Figure 96. ^{1}H NMR (400 MHz, $CDCl_3$) spectra for compound **1-30**

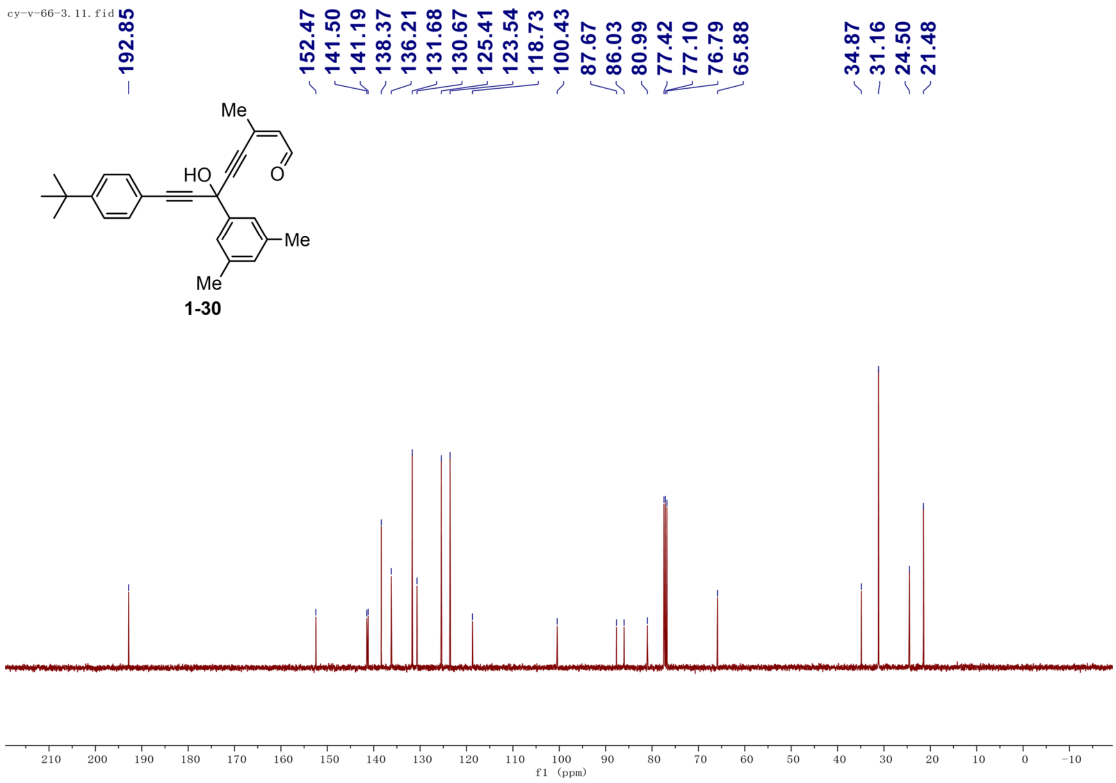

Supplementary Figure 97. ^{13}C NMR (101 MHz, $CDCl_3$) spectra for compound **1-30**

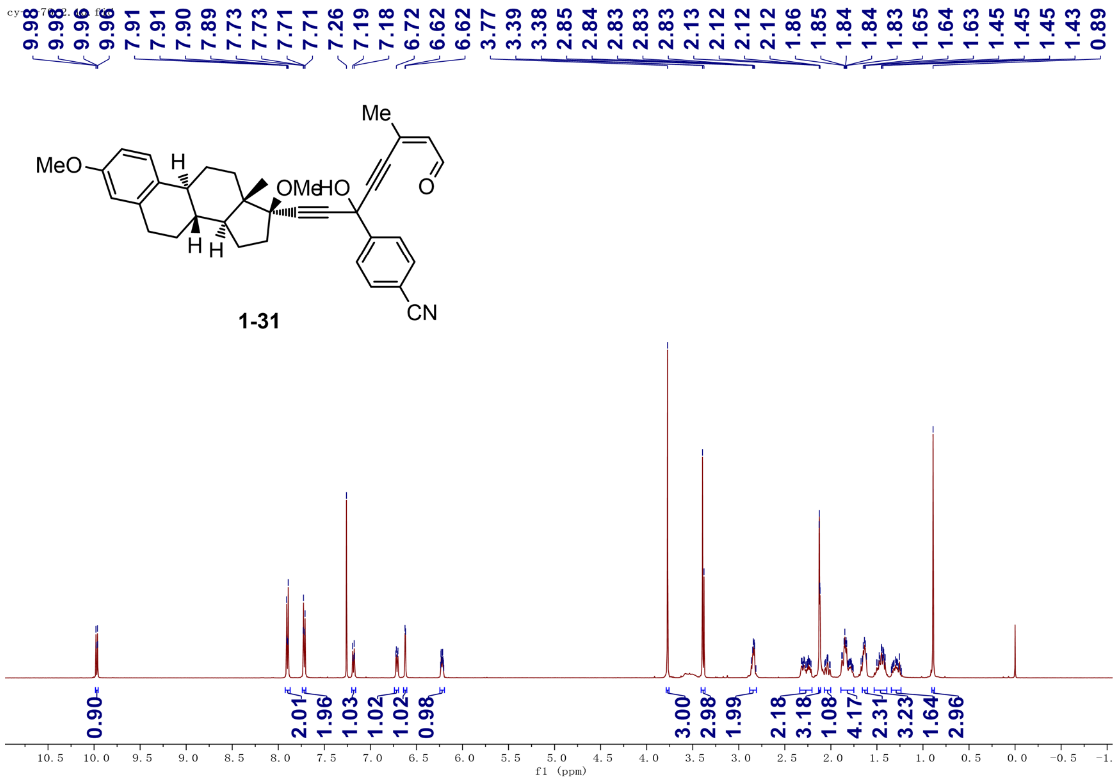

Supplementary Figure 98. ^{1}H NMR (500 MHz, $CDCl_3$) spectra for compound **1-31**

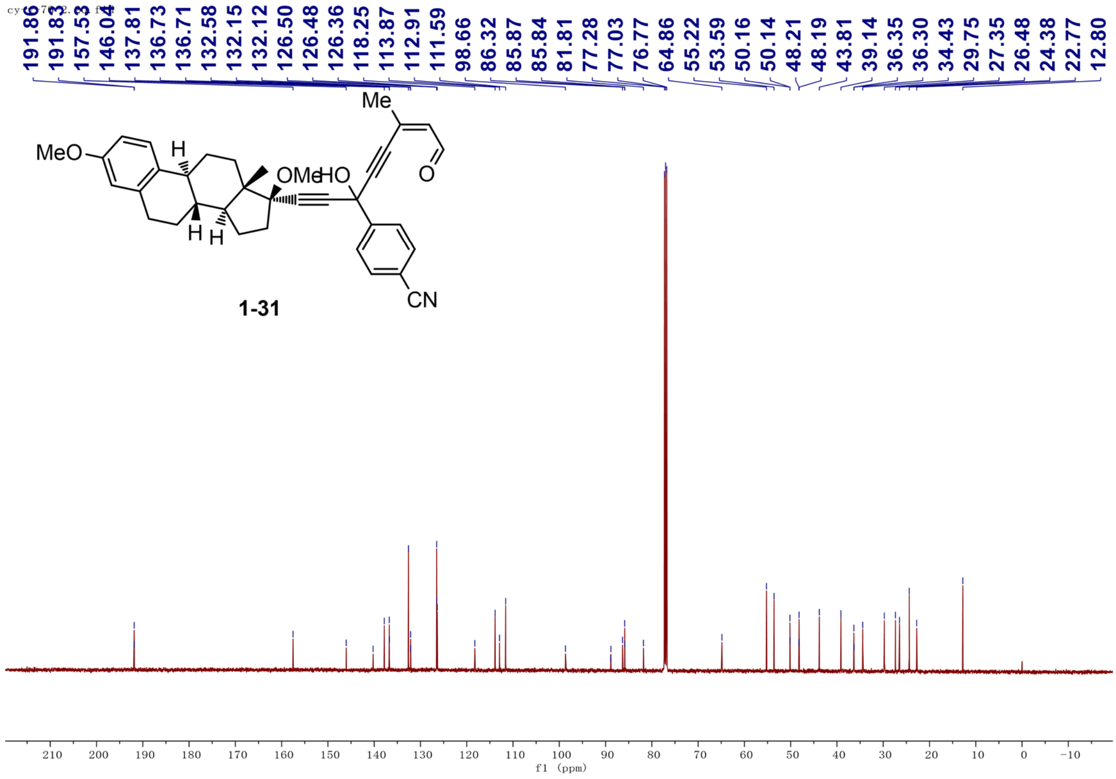

Supplementary Figure 99. ^{13}C NMR (126 MHz, $CDCl_3$) spectra for compound **1-31**

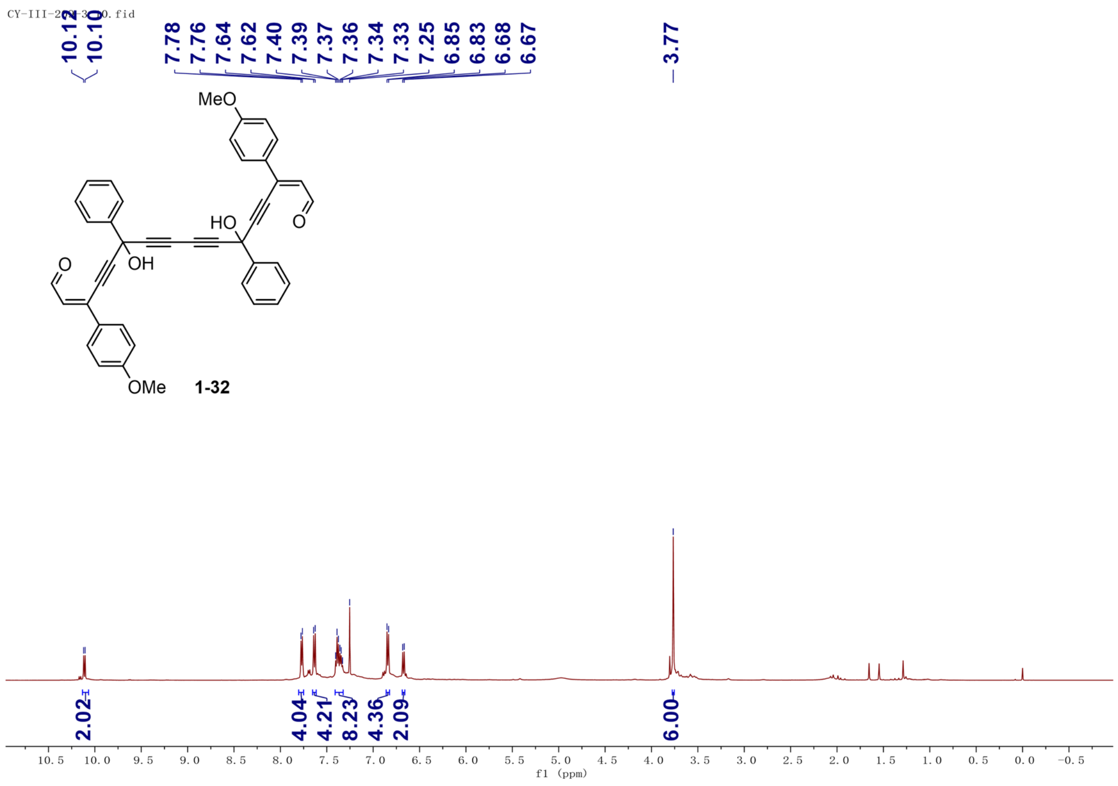

Supplementary Figure 100. ^{1}H NMR (500 MHz, $CDCl_3$) spectra for compound **1-32**

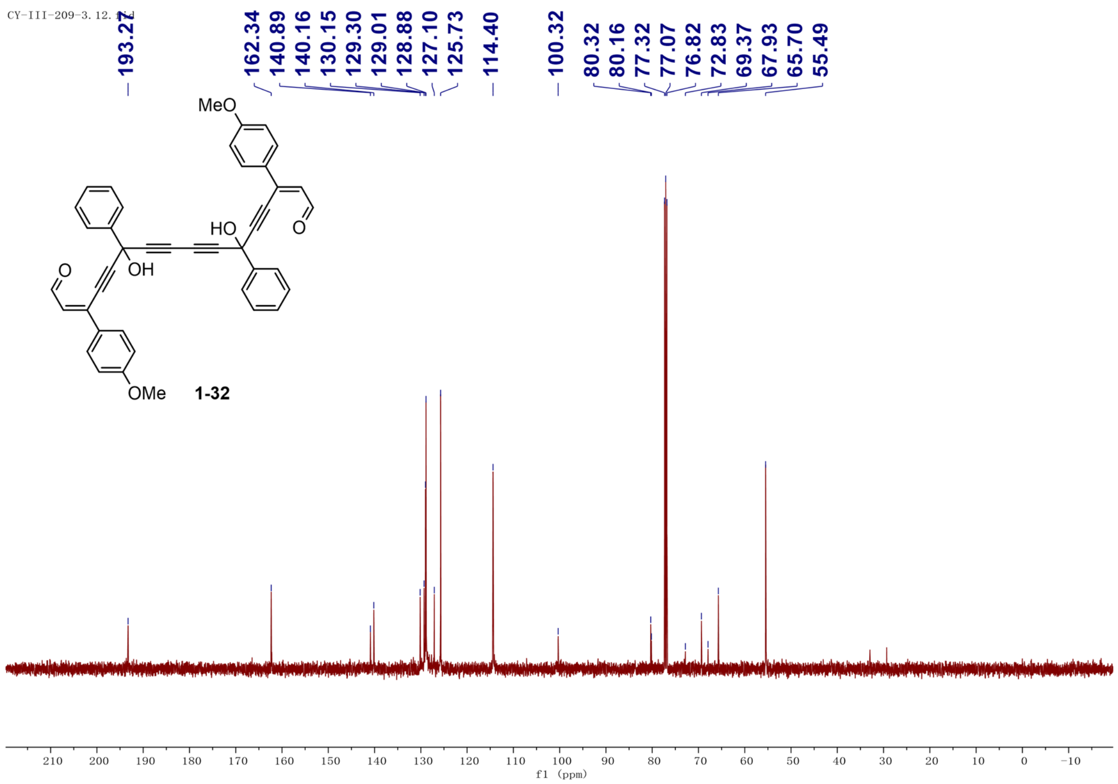

Supplementary Figure 101. ^{13}C NMR (126 MHz, $CDCl_3$) spectra for compound **1-32**

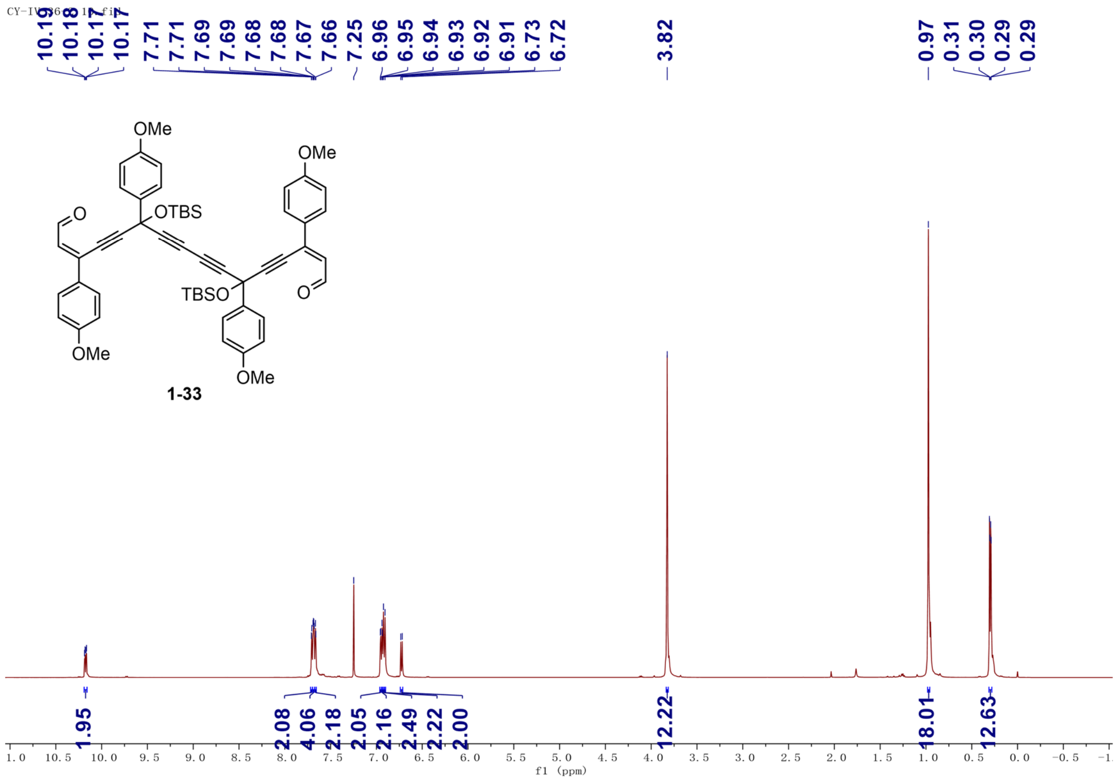

Supplementary Figure 102. ^{1}H NMR (500 MHz, $CDCl_3$) spectra for compound **1-33**

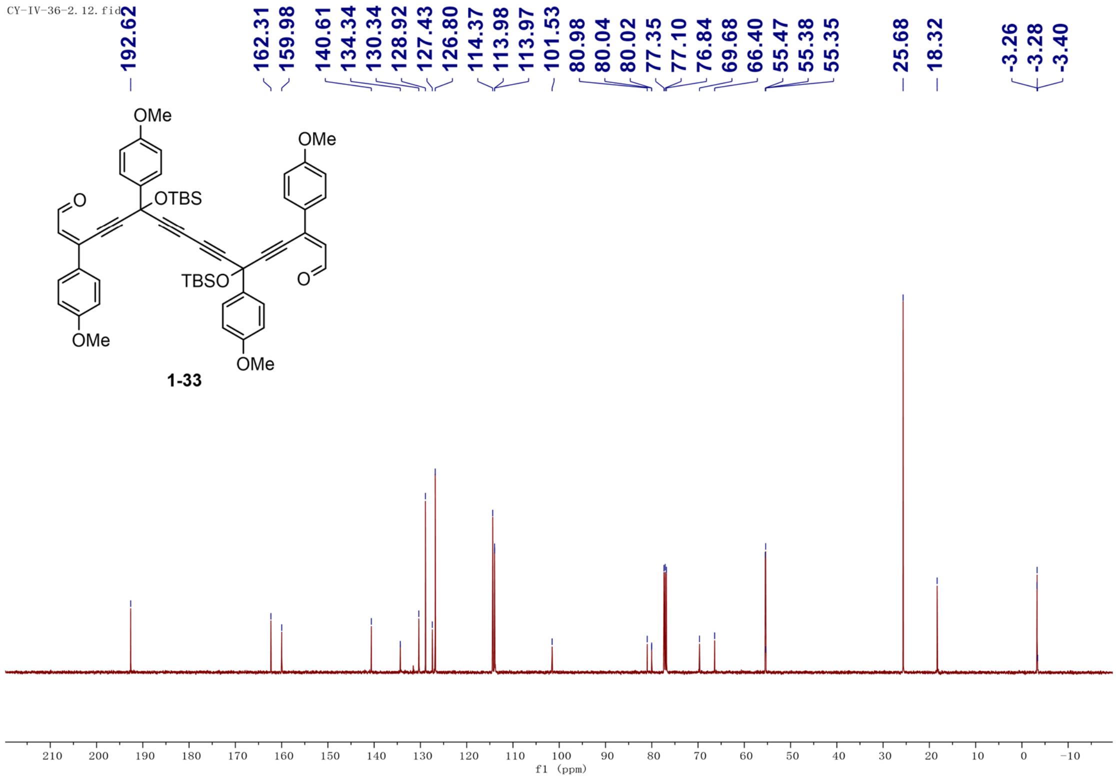

Supplementary Figure 103. ^{13}C NMR (126 MHz, $CDCl_3$) spectra for compound **1-33**

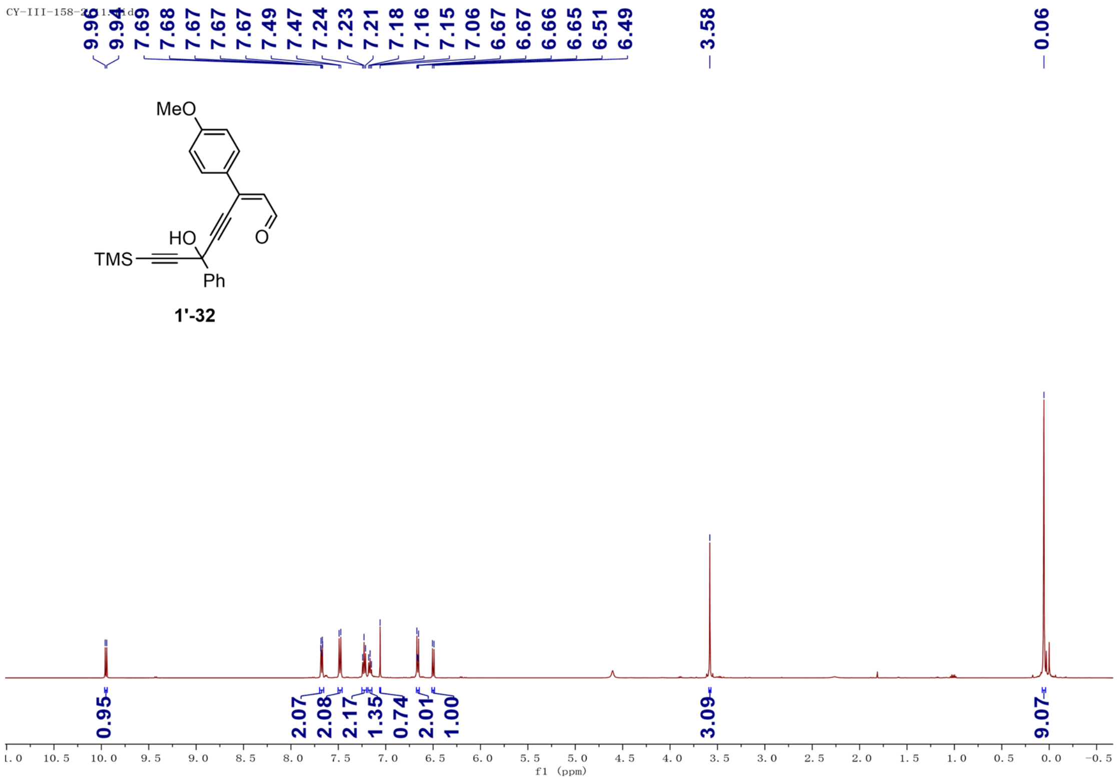

Supplementary Figure 104. ^{1}H NMR (500 MHz, $CDCl_3$) spectra for compound **1'-32**

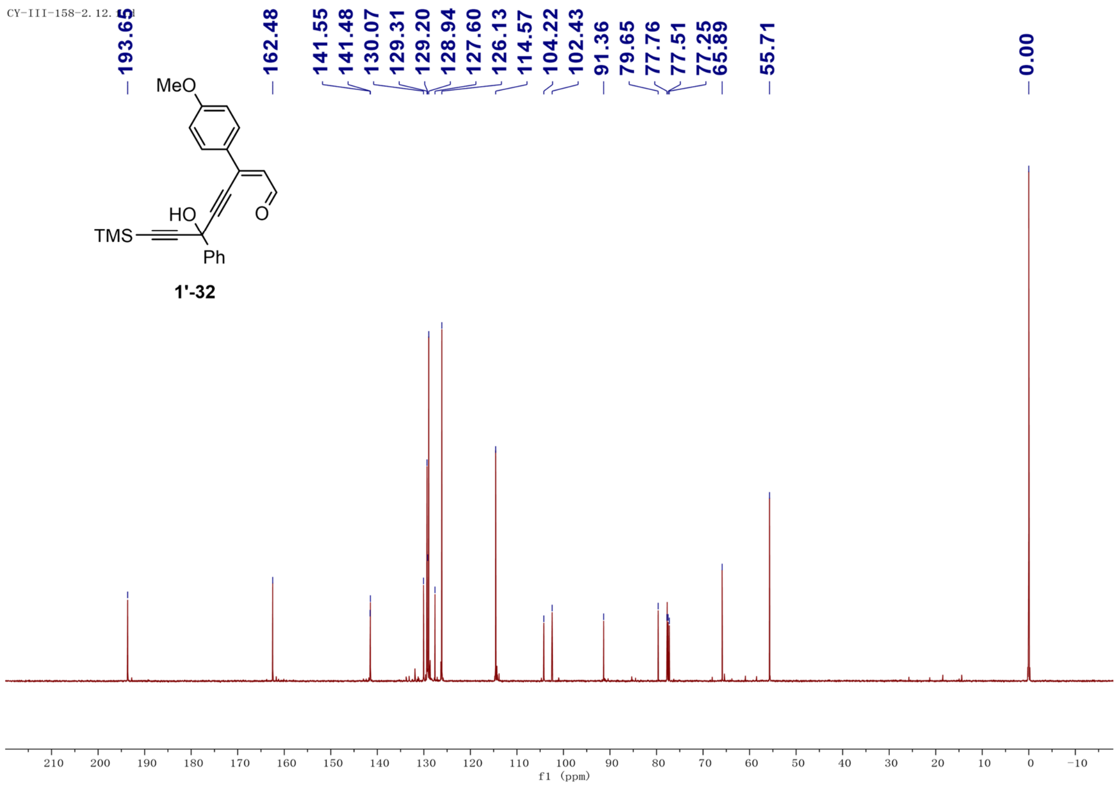

Supplementary Figure 105. ^{13}C NMR (126 MHz, $CDCl_3$) spectra for compound **1'-32**

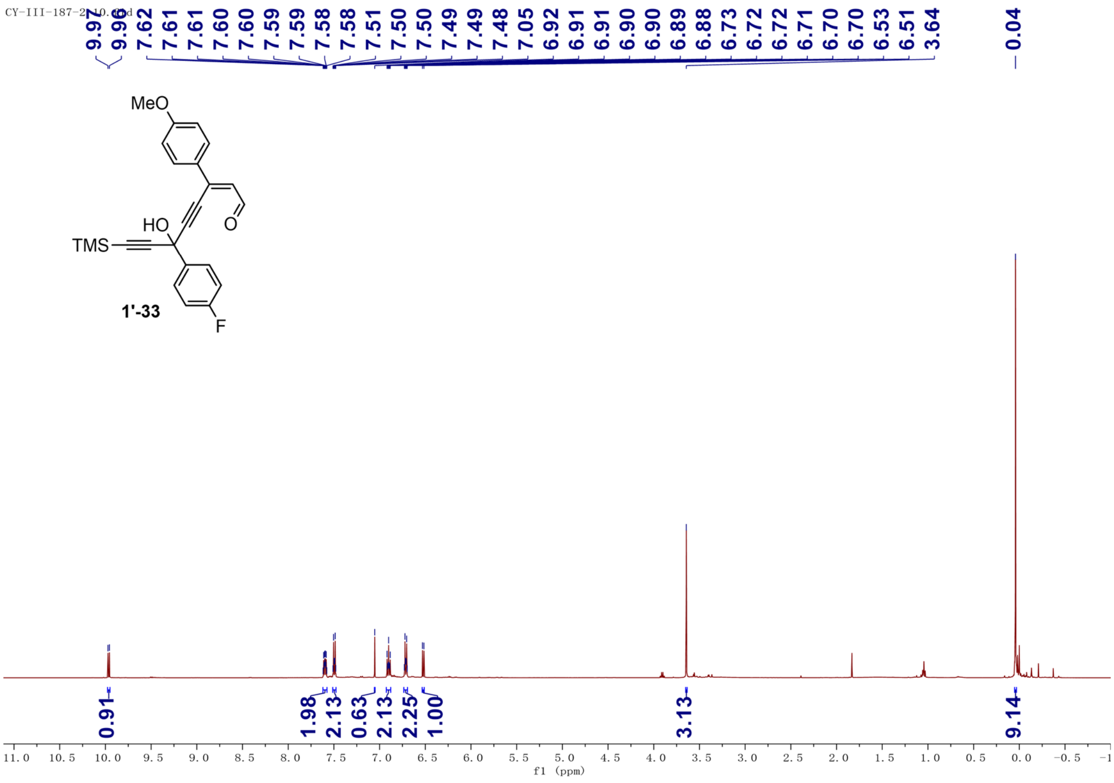

Supplementary Figure 106. ^{1}H NMR (500 MHz, $CDCl_3$) spectra for compound **1'-33**

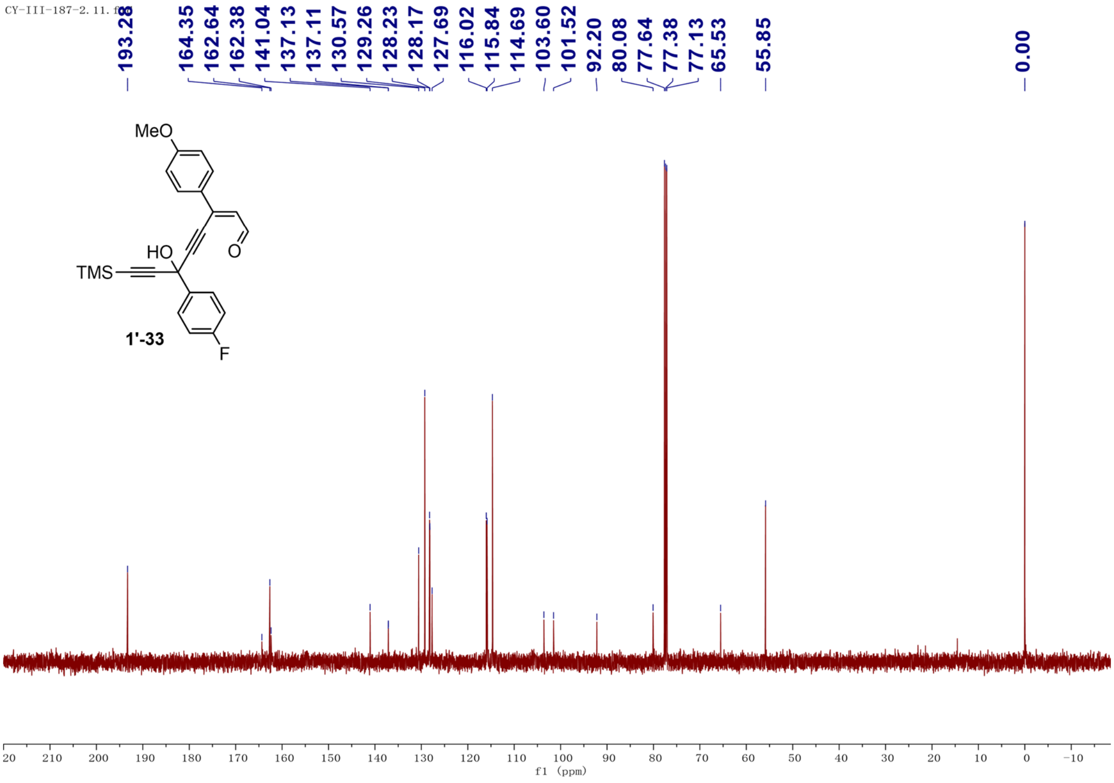

Supplementary Figure 107. ^{13}C NMR (126 MHz, $CDCl_3$) spectra for compound **1'-33**

CY-III-187-2.21.fid

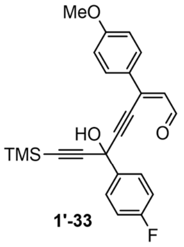

1'-33

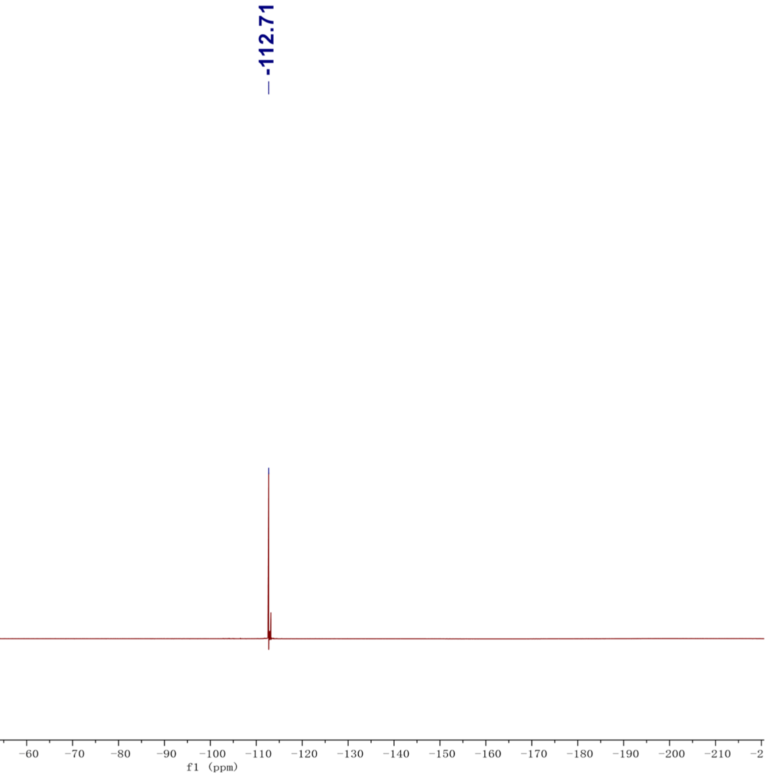

Supplementary Figure 108. ^{19}F NMR (471 MHz, $CDCl_3$) spectra for compound **1'-33**

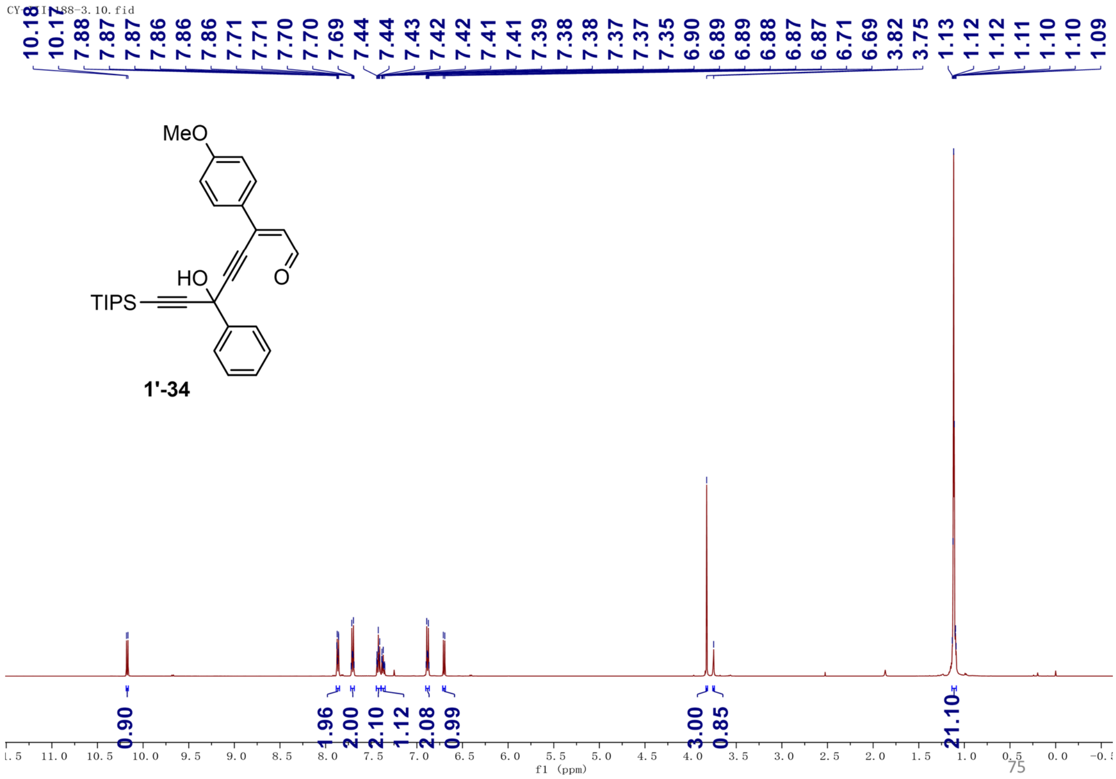

Supplementary Figure 109. ^{1}H NMR (500 MHz, $CDCl_3$) spectra for compound **1'-34**

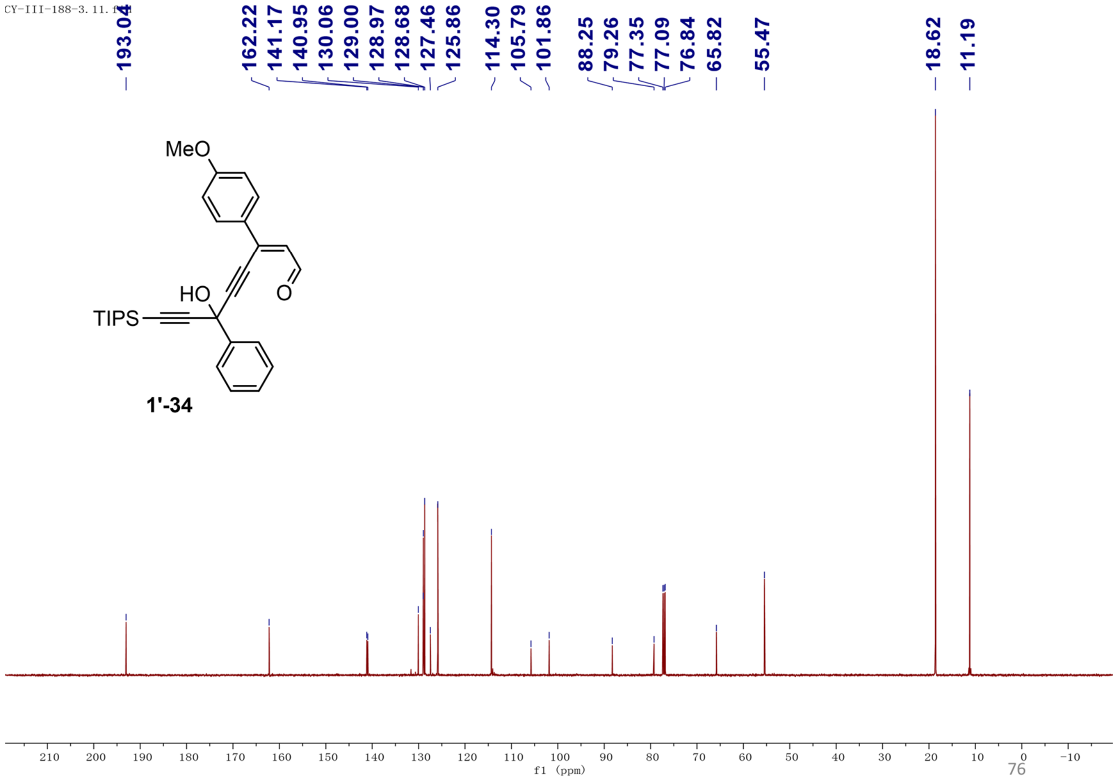

Supplementary Figure 110. ^{13}C NMR (126 MHz, $CDCl_3$) spectra for compound **1'-34**

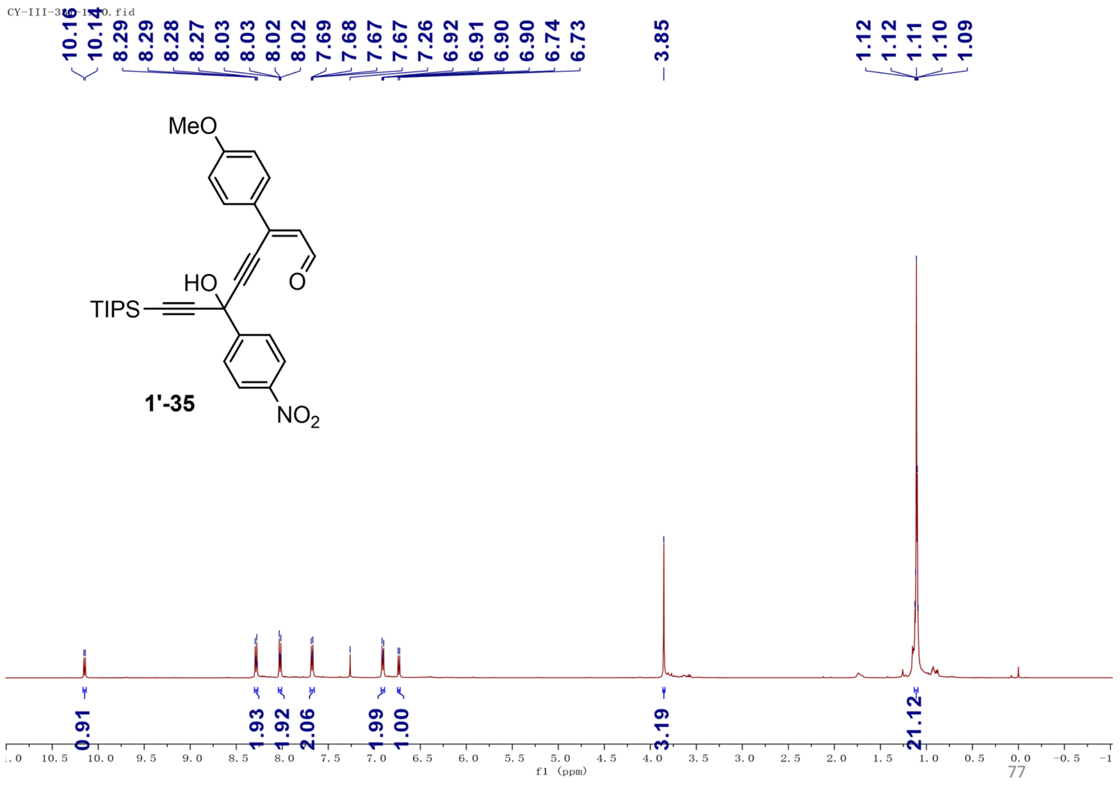

Supplementary Figure 111. ^{1}H NMR (500 MHz, $CDCl_3$) spectra for compound **1'-35**

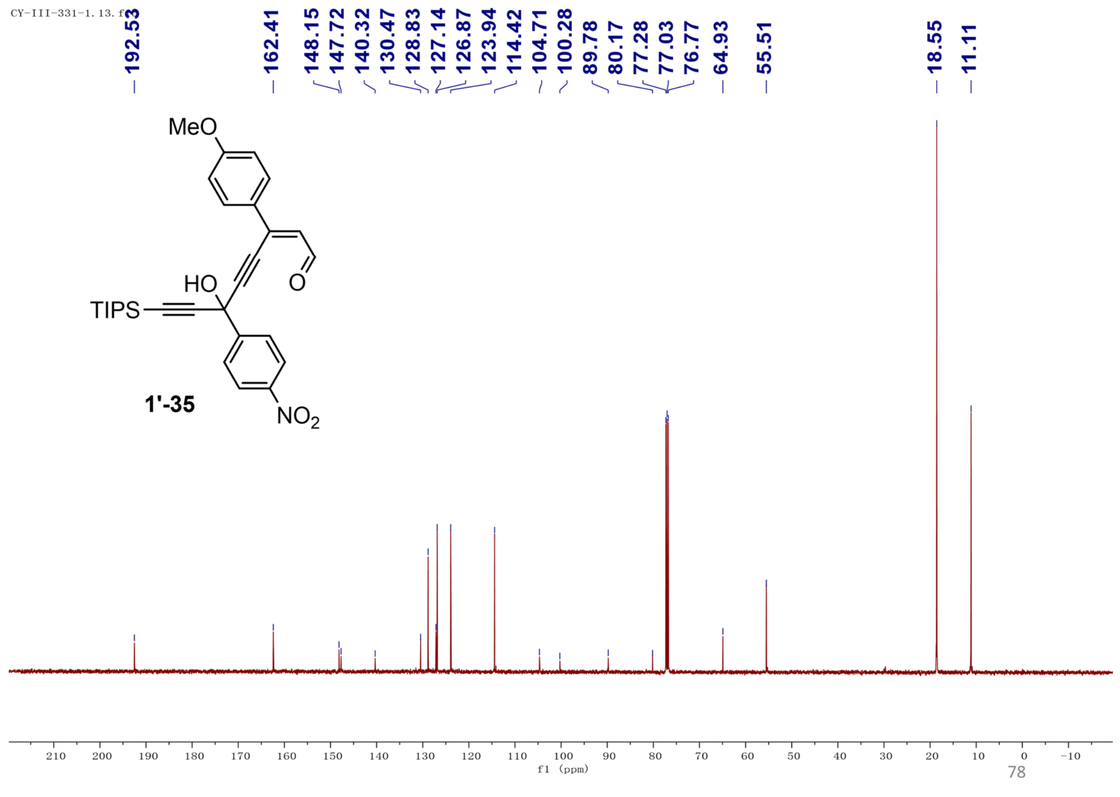

Supplementary Figure 112. ^{13}C NMR (126 MHz, $CDCl_3$) spectra for compound **1'-35**

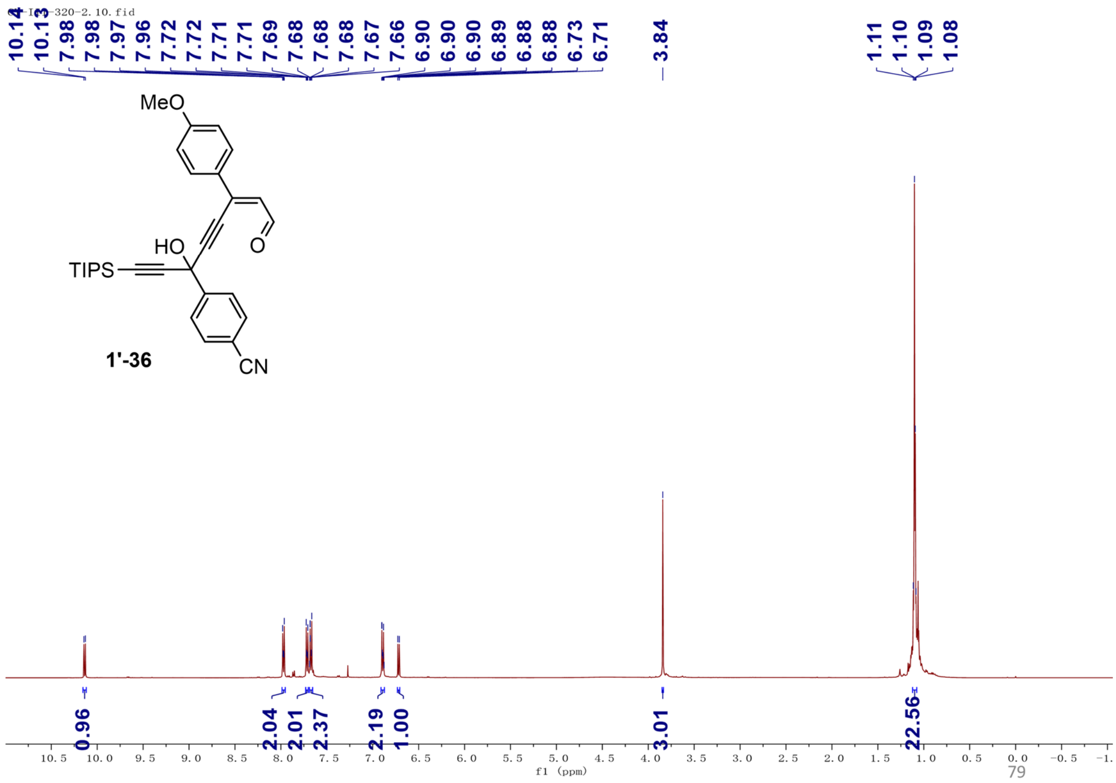

Supplementary Figure 113. ^{1}H NMR (500 MHz, $CDCl_3$) spectra for compound **1'-36**

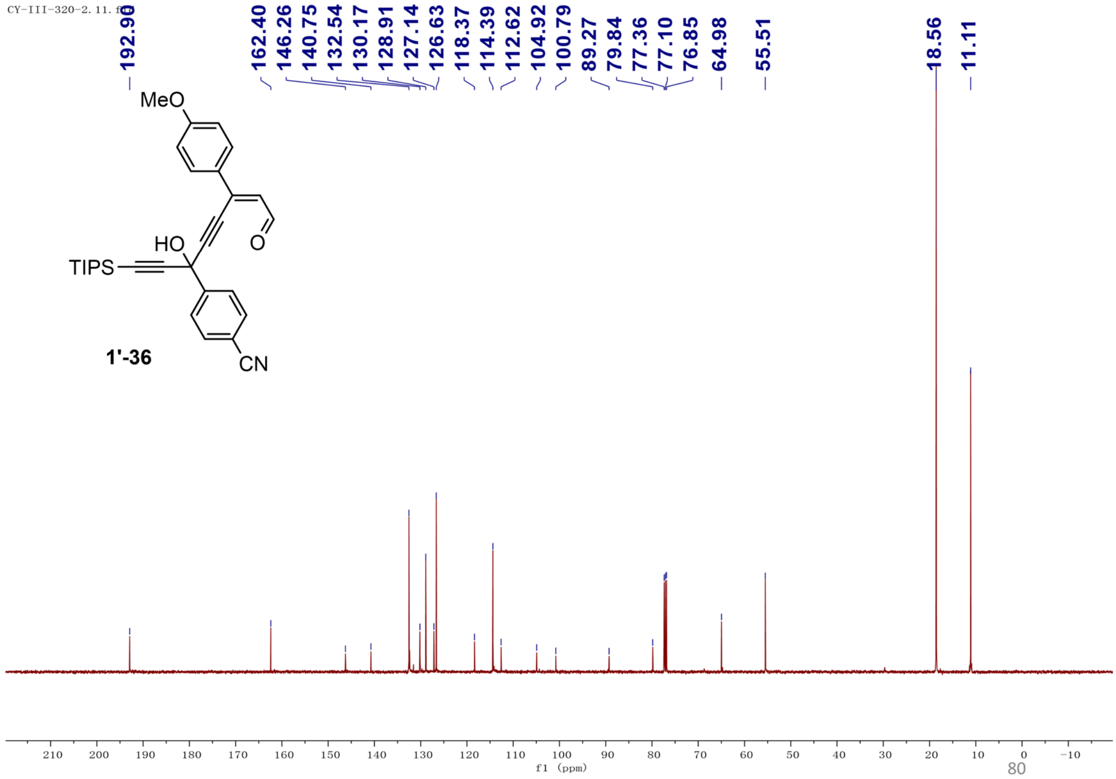

Supplementary Figure 114. ^{13}C NMR (126 MHz, $CDCl_3$) spectra for compound **1'-36**

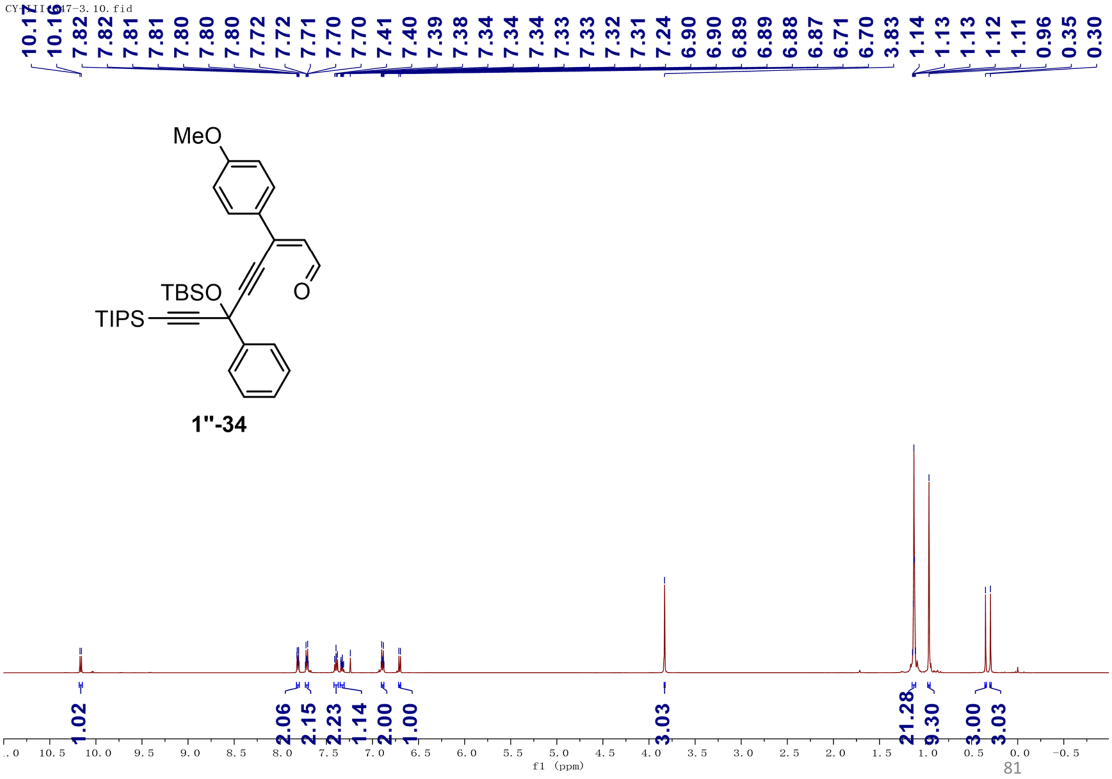

Supplementary Figure 115. ^{1}H NMR (500 MHz, $CDCl_3$) spectra for compound **1''-34**

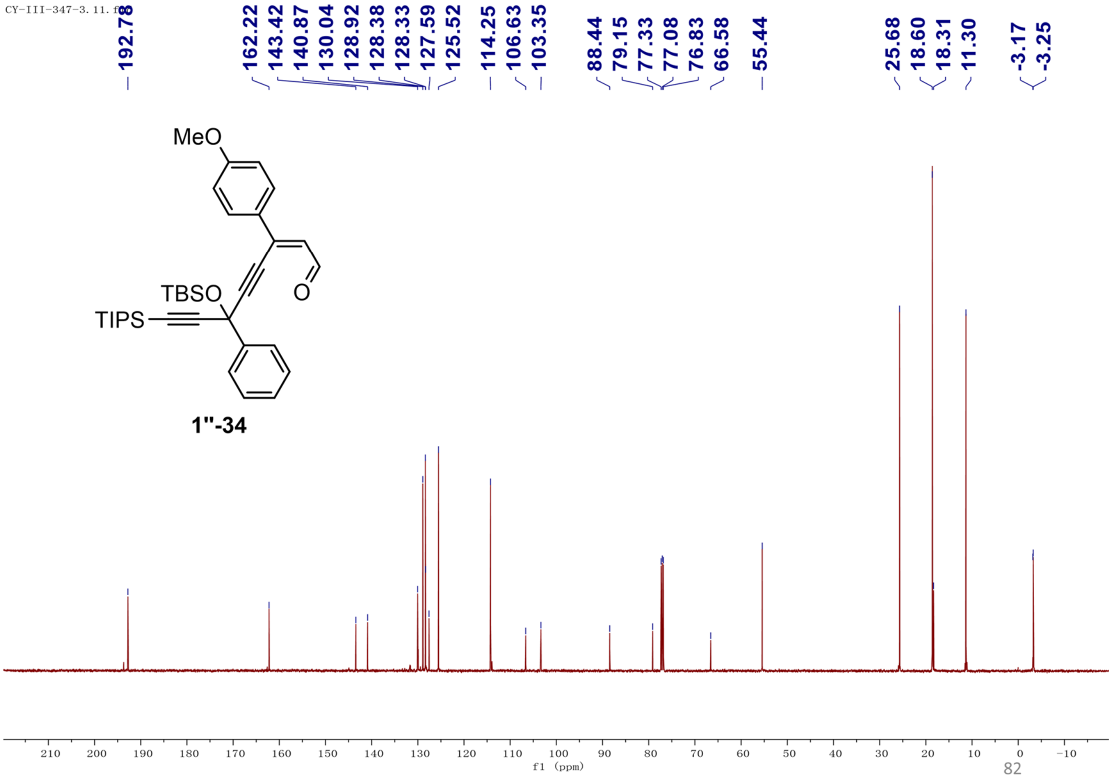

Supplementary Figure 116. ^{13}C NMR (126 MHz, $CDCl_3$) spectra for compound **1''-34**

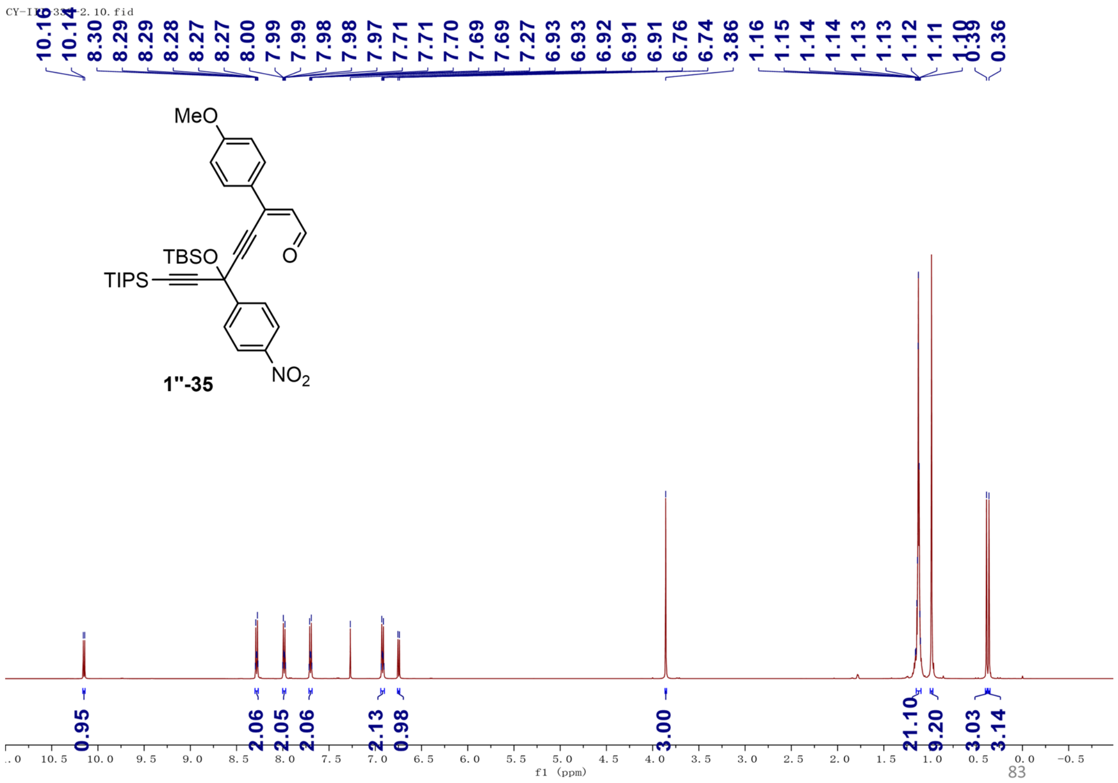

Supplementary Figure 117. ^{1}H NMR (500 MHz, $CDCl_3$) spectra for compound **1''-35**

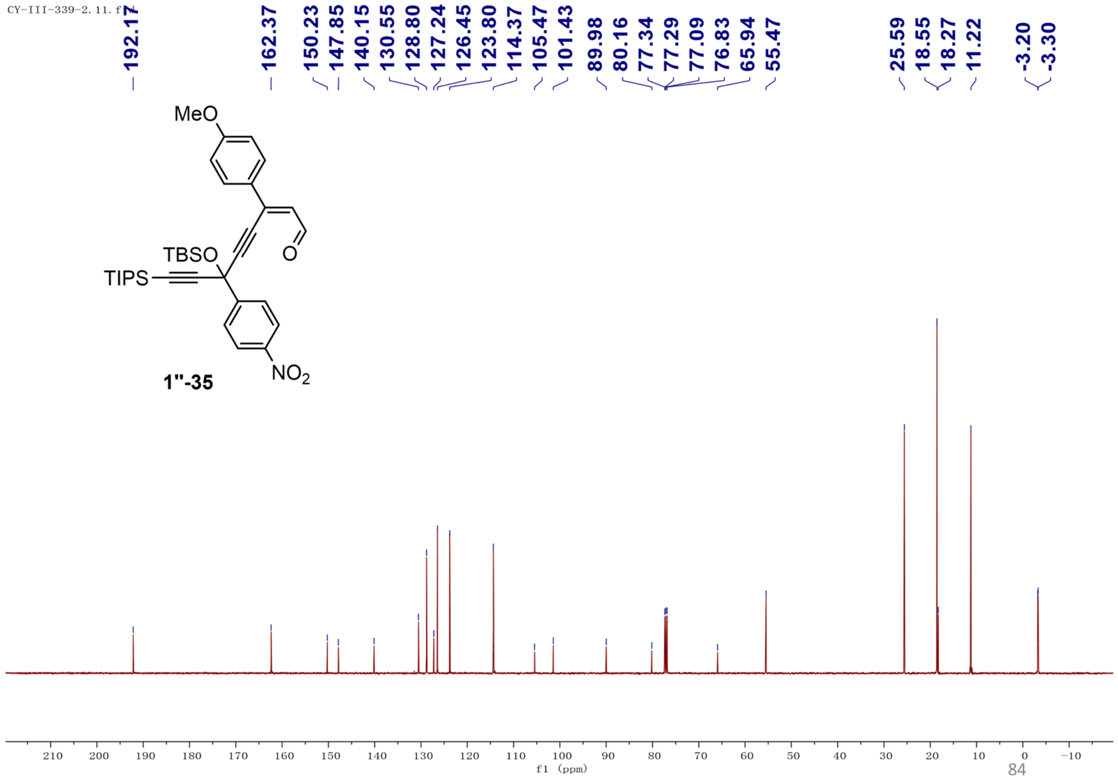

Supplementary Figure 118. ^{13}C NMR (126 MHz, $CDCl_3$) spectra for compound **1''-35**

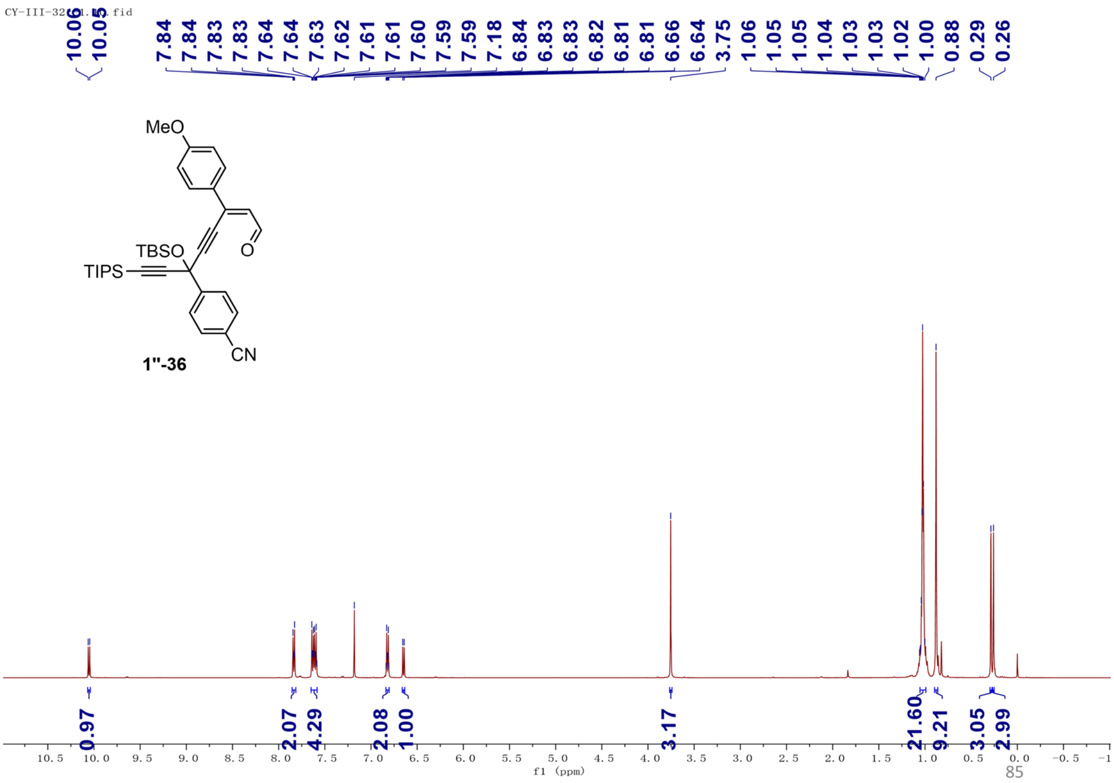

Supplementary Figure 119. ^{1}H NMR (500 MHz, $CDCl_3$) spectra for compound **1''-36**

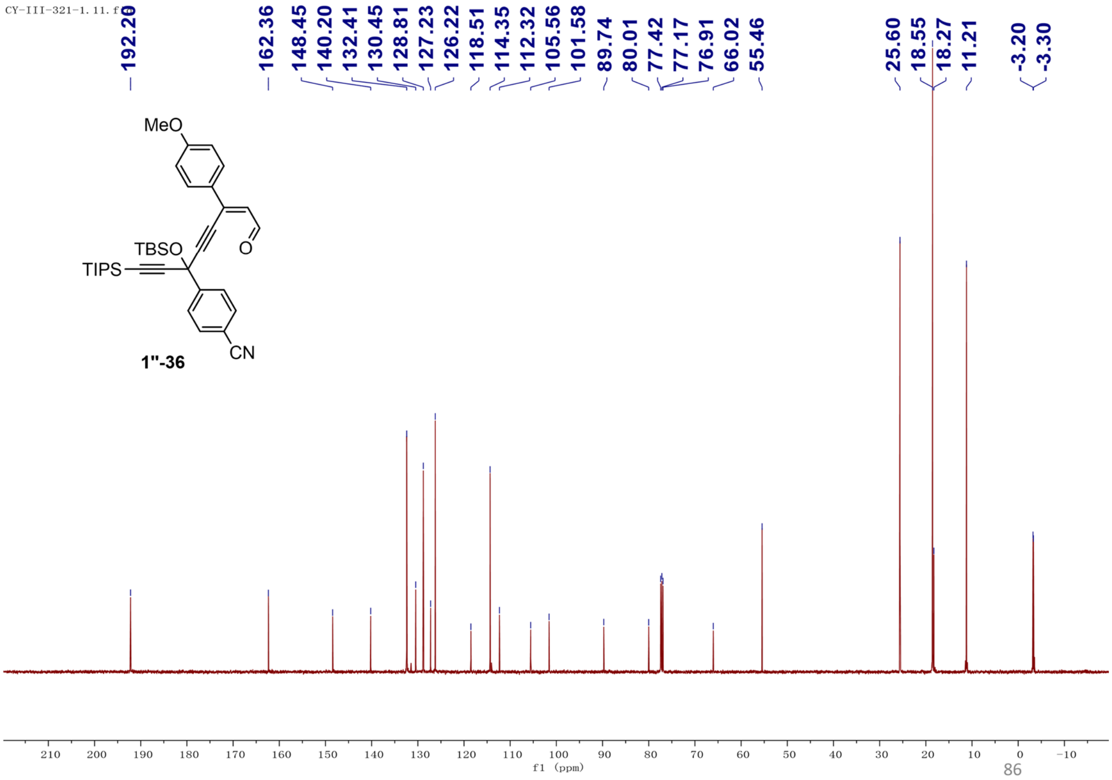

Supplementary Figure 120. ^{13}C NMR (126 MHz, $CDCl_3$) spectra for compound **1''-36**

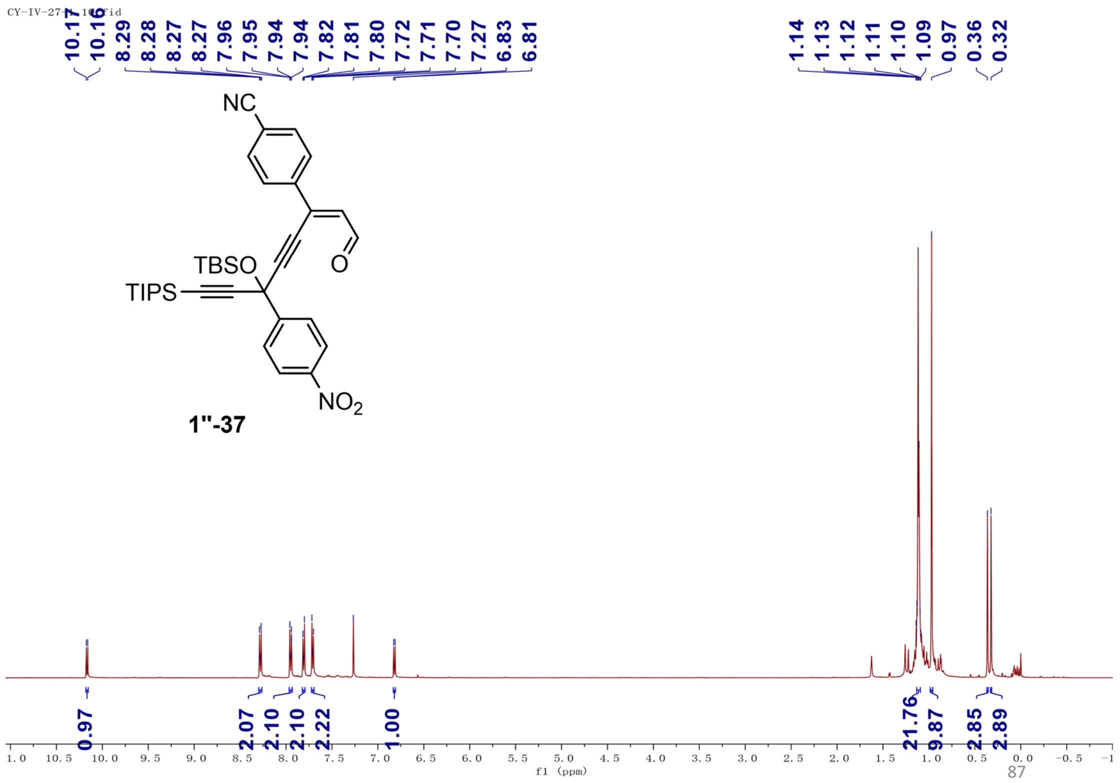

Supplementary Figure 121. ^{1}H NMR (500 MHz, $CDCl_3$) spectra for compound **1''-37**

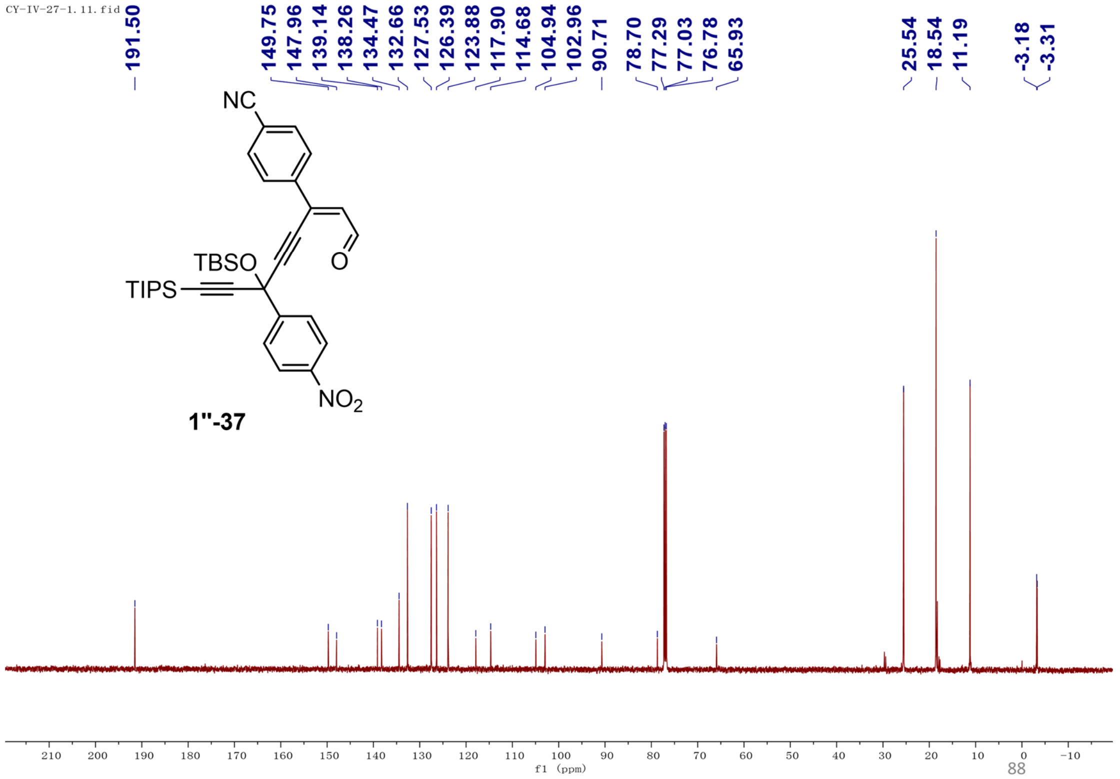

Supplementary Figure 122. ^{13}C NMR (126 MHz, $CDCl_3$) spectra for compound **1''-37**

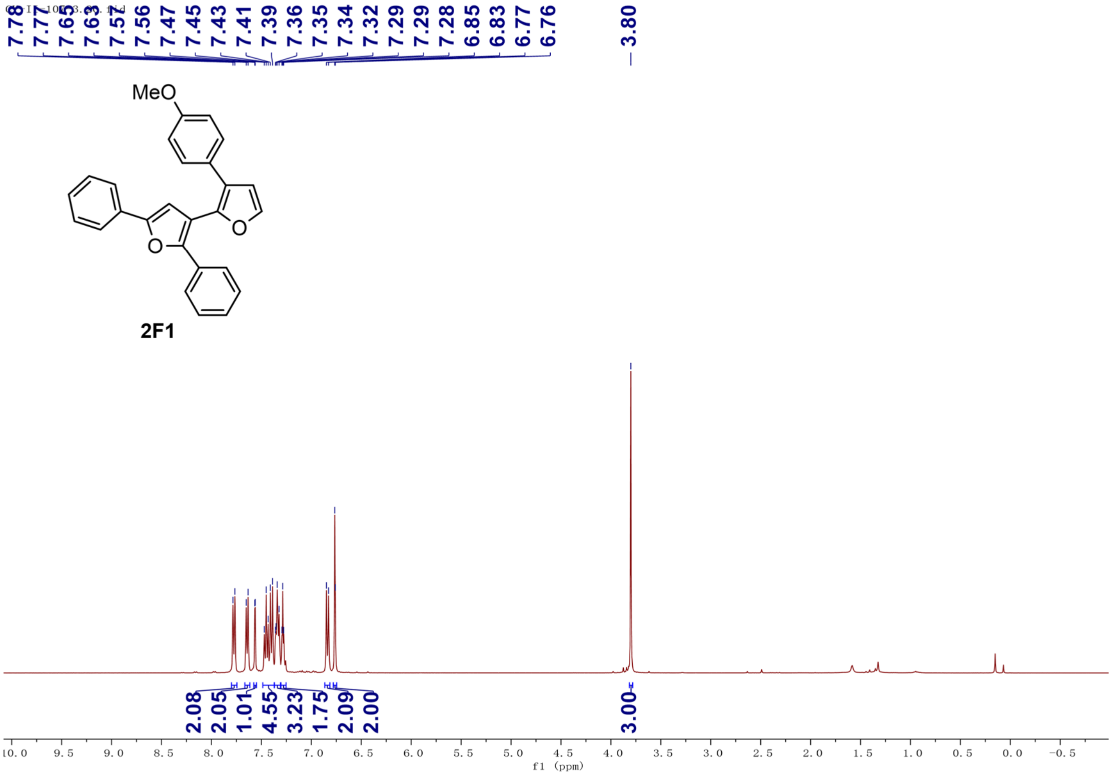

Supplementary Figure 123. ^{1}H NMR (400 MHz, $CDCl_3$) spectra for compound **2F-1**

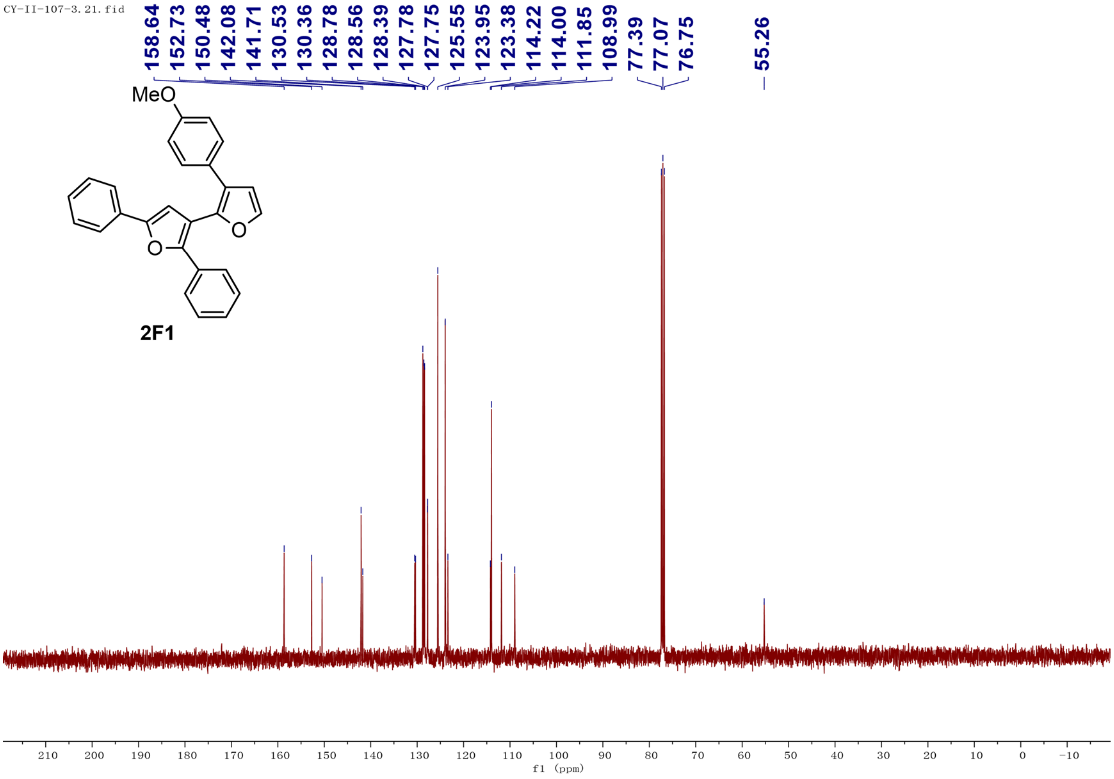

Supplementary Figure 124. ^{13}C NMR (101 MHz, $CDCl_3$) spectra for compound **2F1**

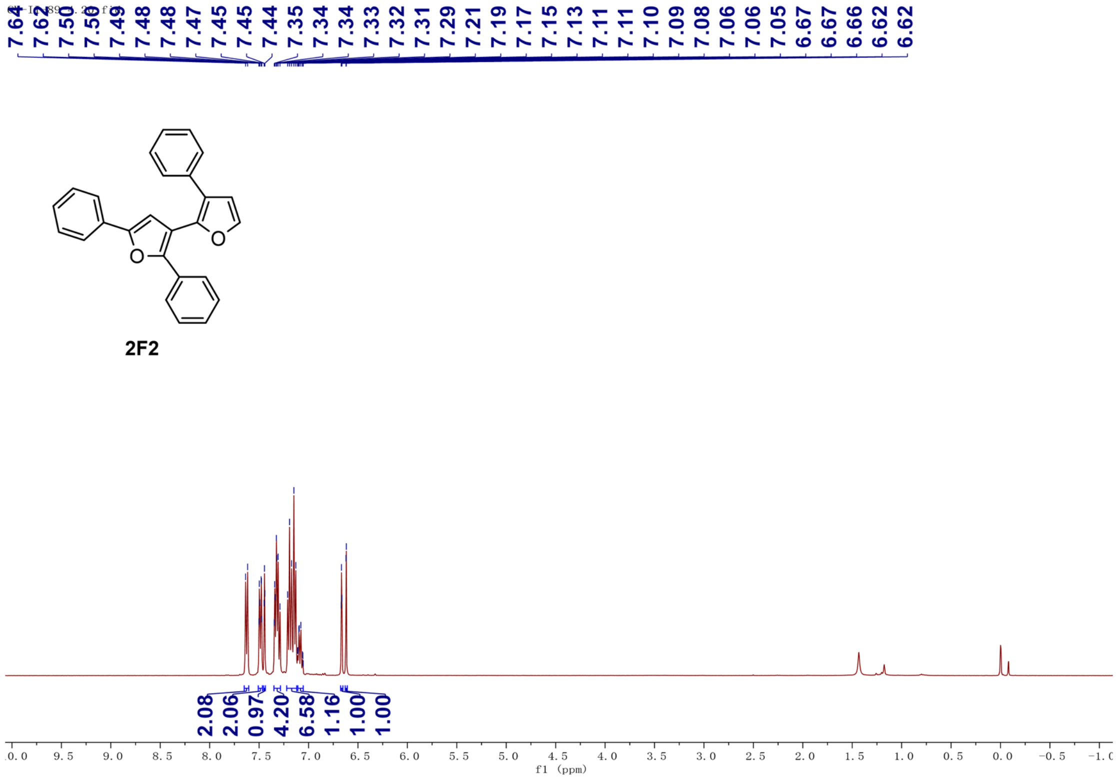

Supplementary Figure 125. ^{1}H NMR (400 MHz, $CDCl_3$) spectra for compound **2F2**

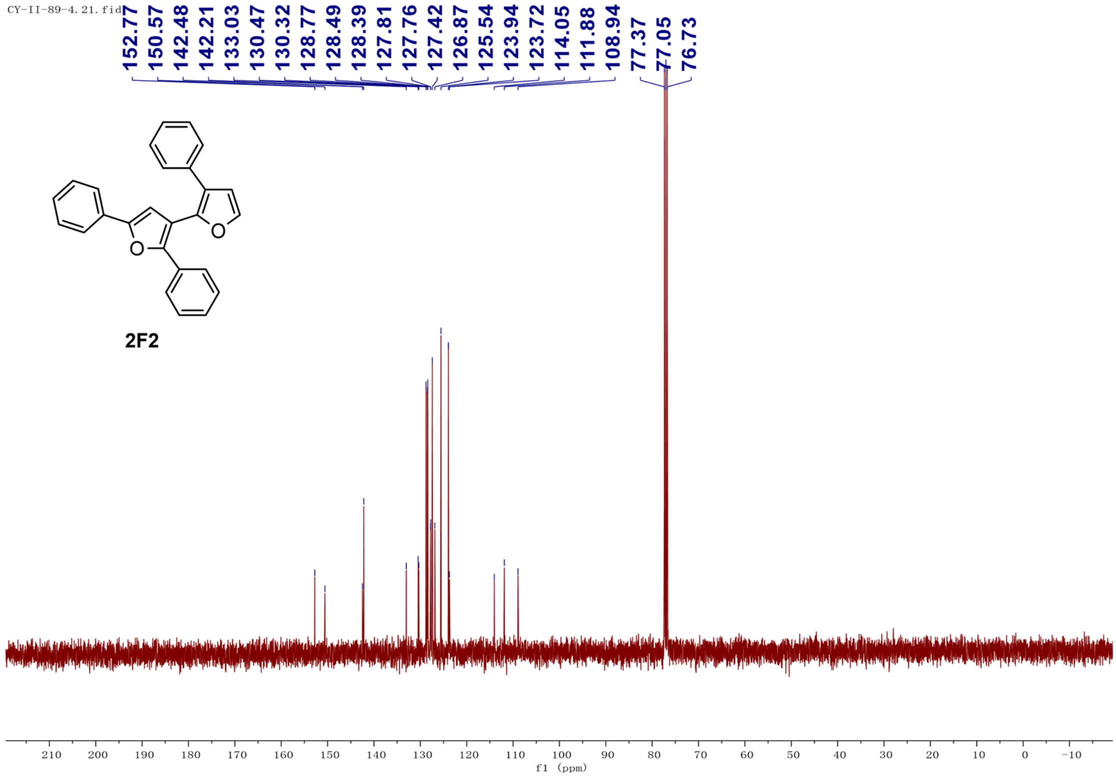

Supplementary Figure 126. ^{13}C NMR (101 MHz, $CDCl_3$) spectra for compound **2F2**

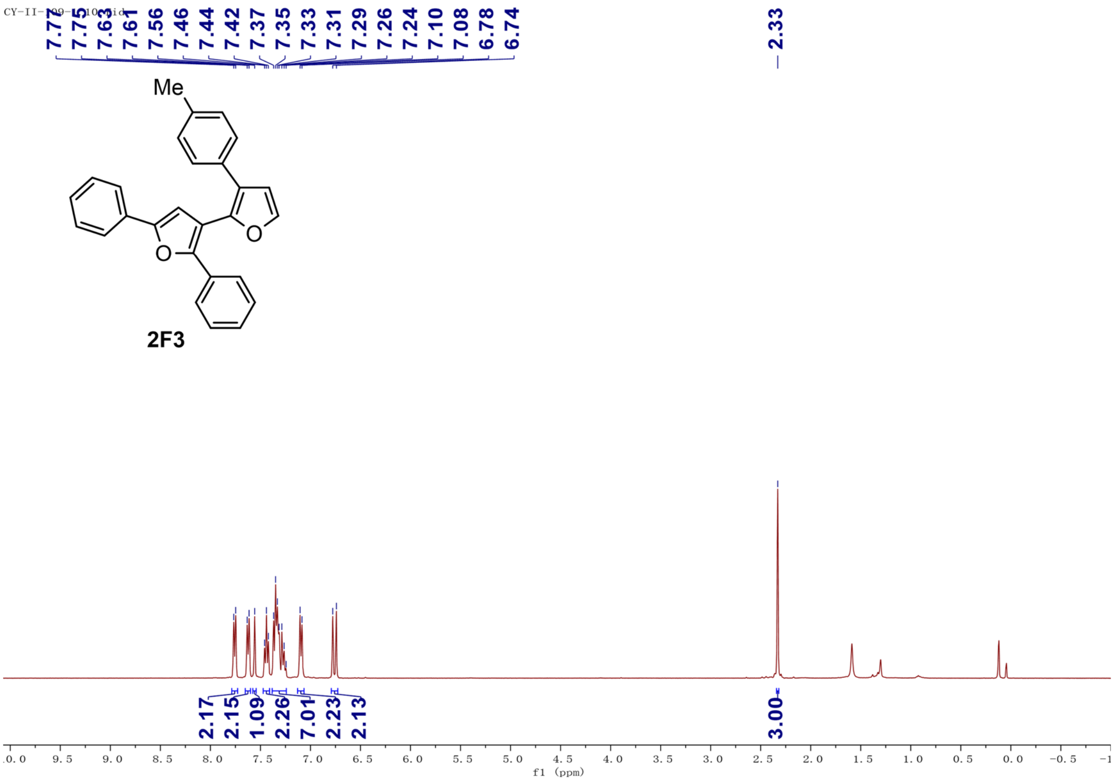

Supplementary Figure 127. ^{1}H NMR (400 MHz, $CDCl_3$) spectra for compound **2F3**

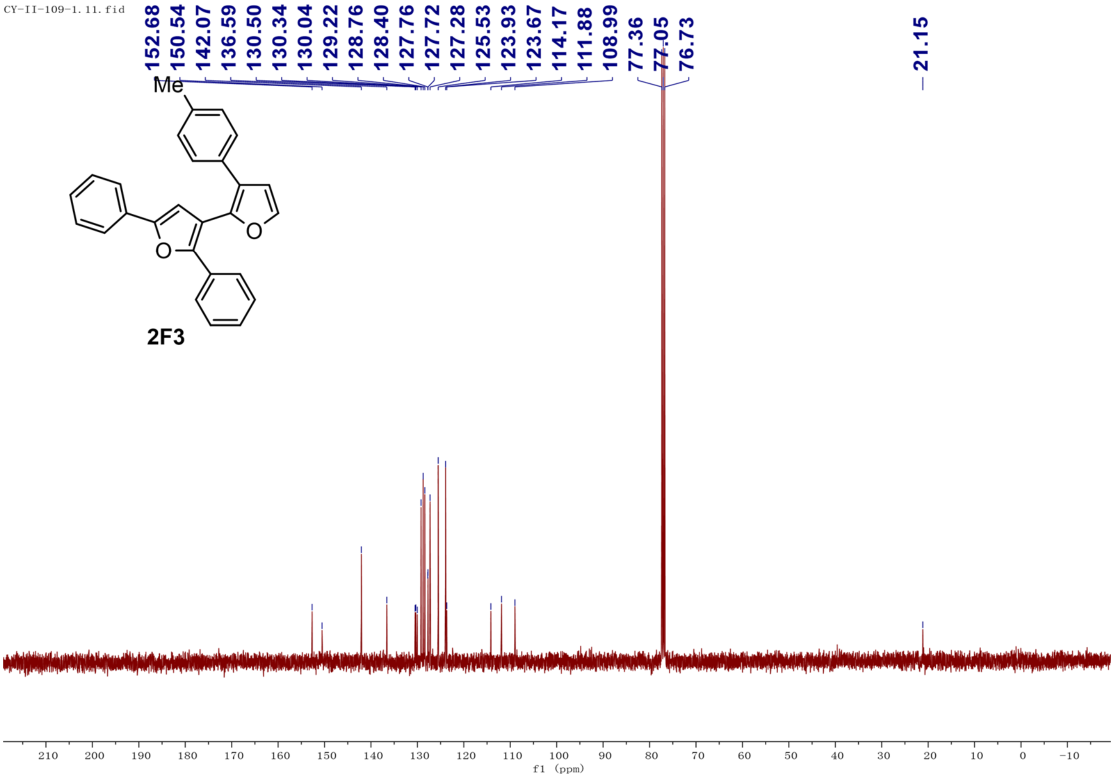

Supplementary Figure 128. ^{13}C NMR (101 MHz, $CDCl_3$) spectra for compound **2F3**

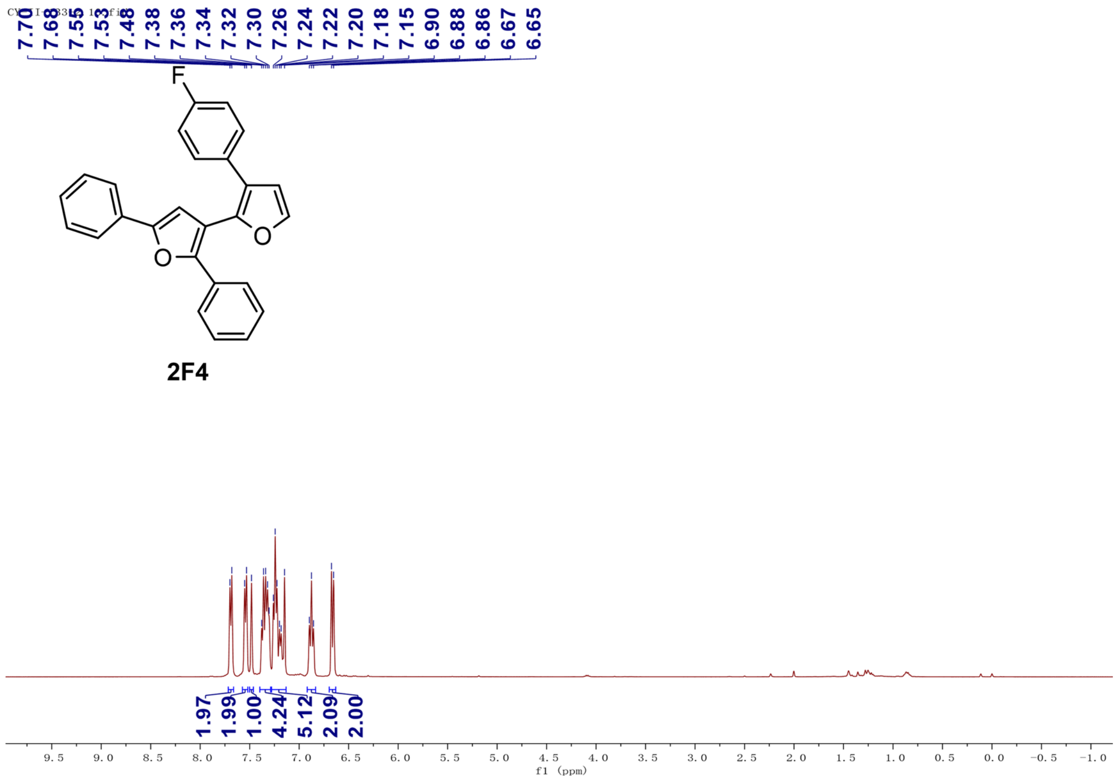

Supplementary Figure 129. ^{1}H NMR (400 MHz, $CDCl_3$) spectra for compound **2F4**

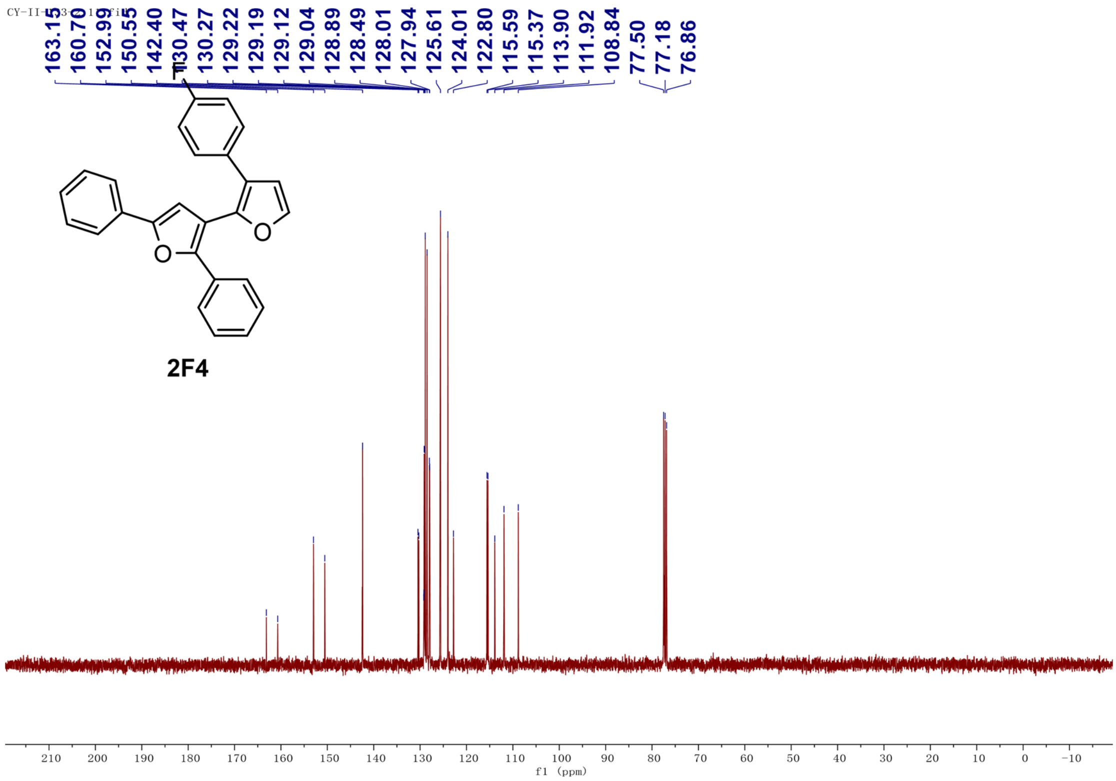

Supplementary Figure 130. ^{13}C NMR (101 MHz, $CDCl_3$) spectra for compound **2F4**

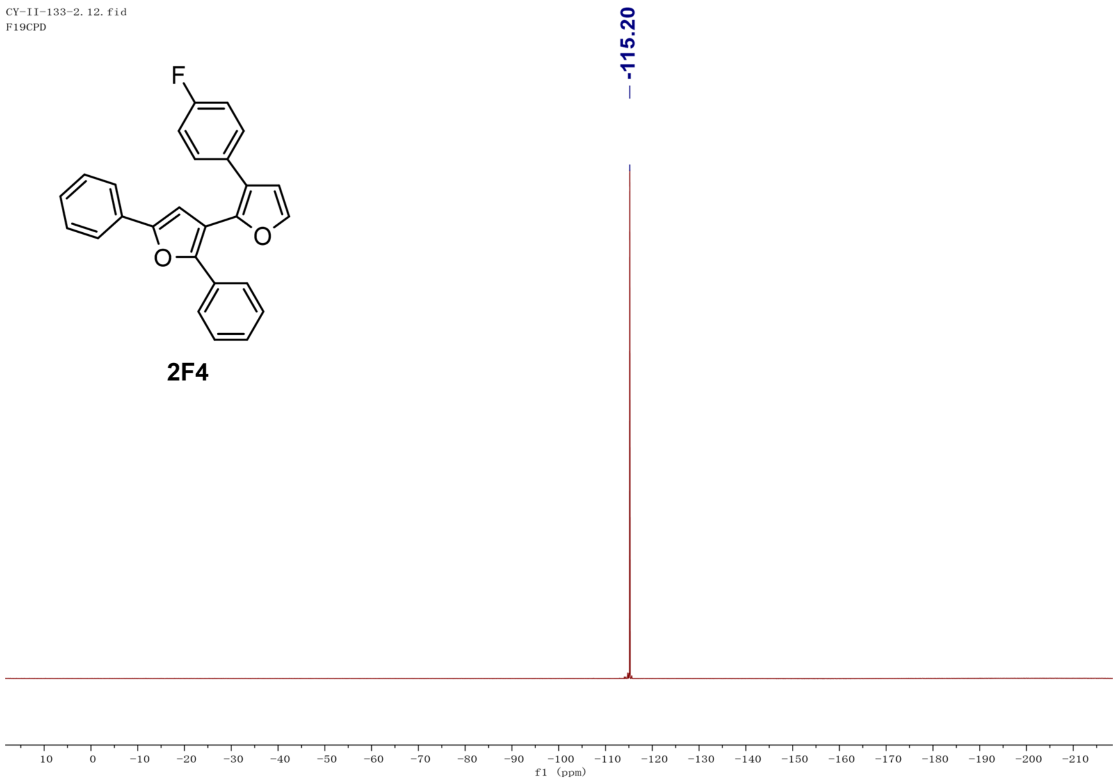

Supplementary Figure 131. ^{19}F NMR (376 MHz, $CDCl_3$) spectra for compound **2F4**

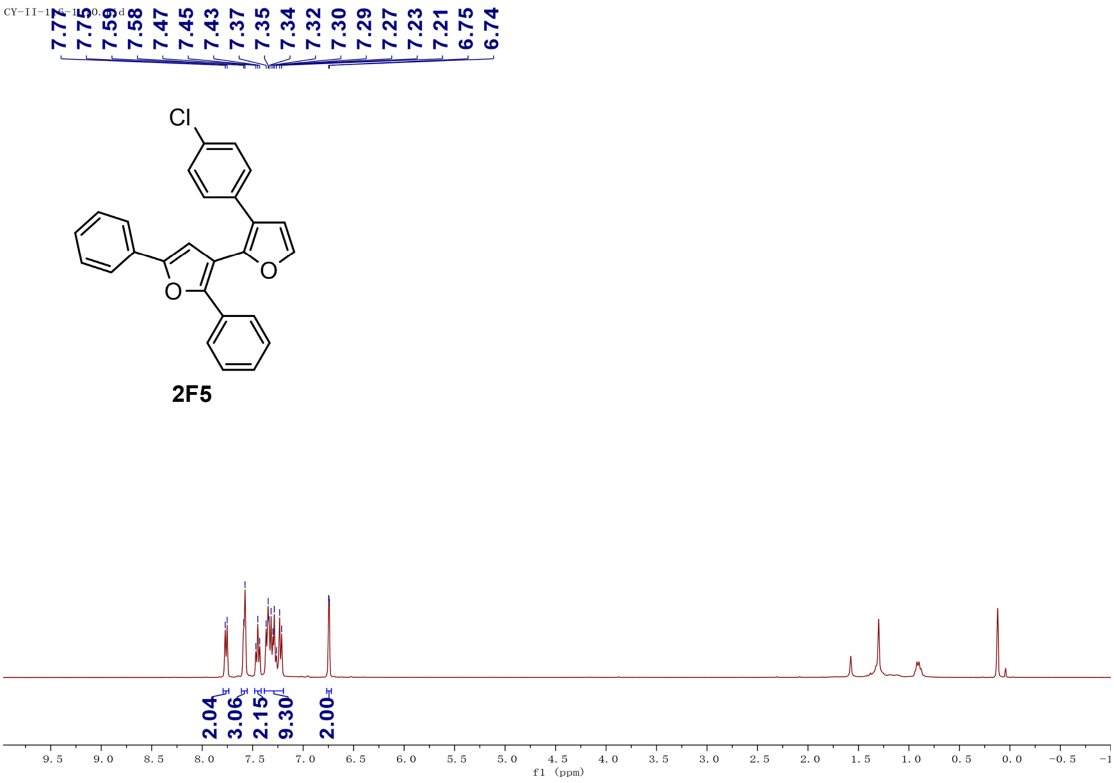

Supplementary Figure 132. ^{1}H NMR (400 MHz, $CDCl_3$) spectra for compound **2F5**

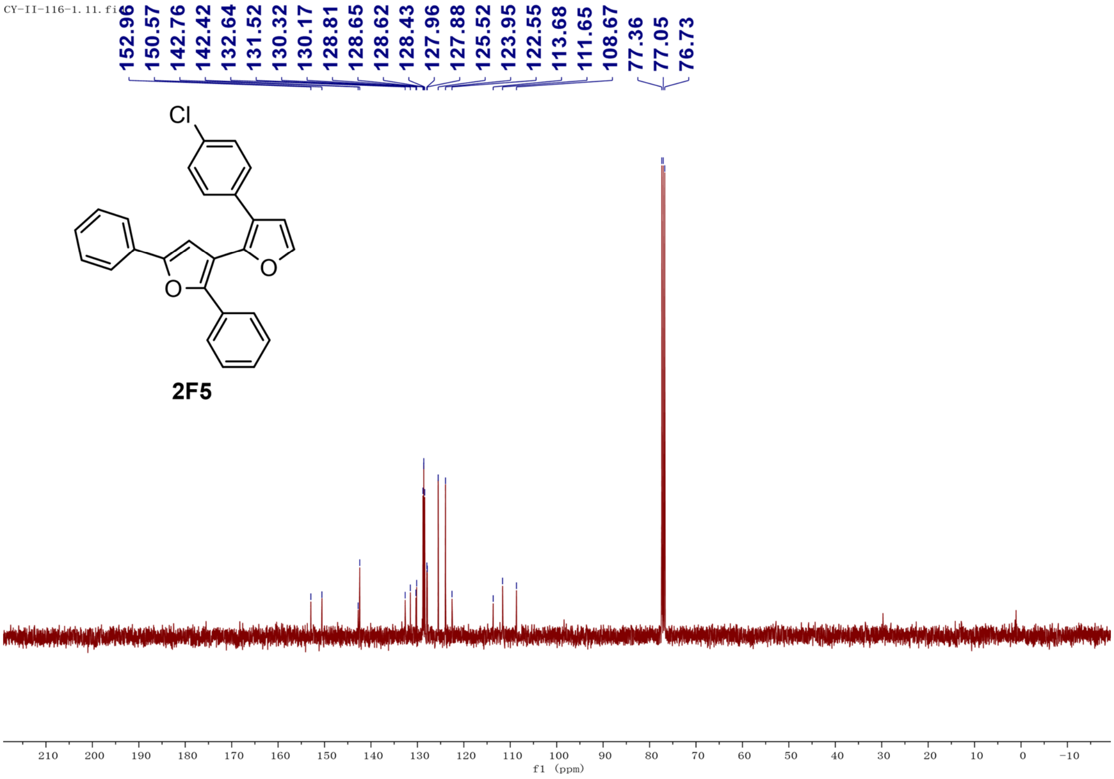

Supplementary Figure 133. ^{13}C NMR (101 MHz, $CDCl_3$) spectra for compound **2F5**

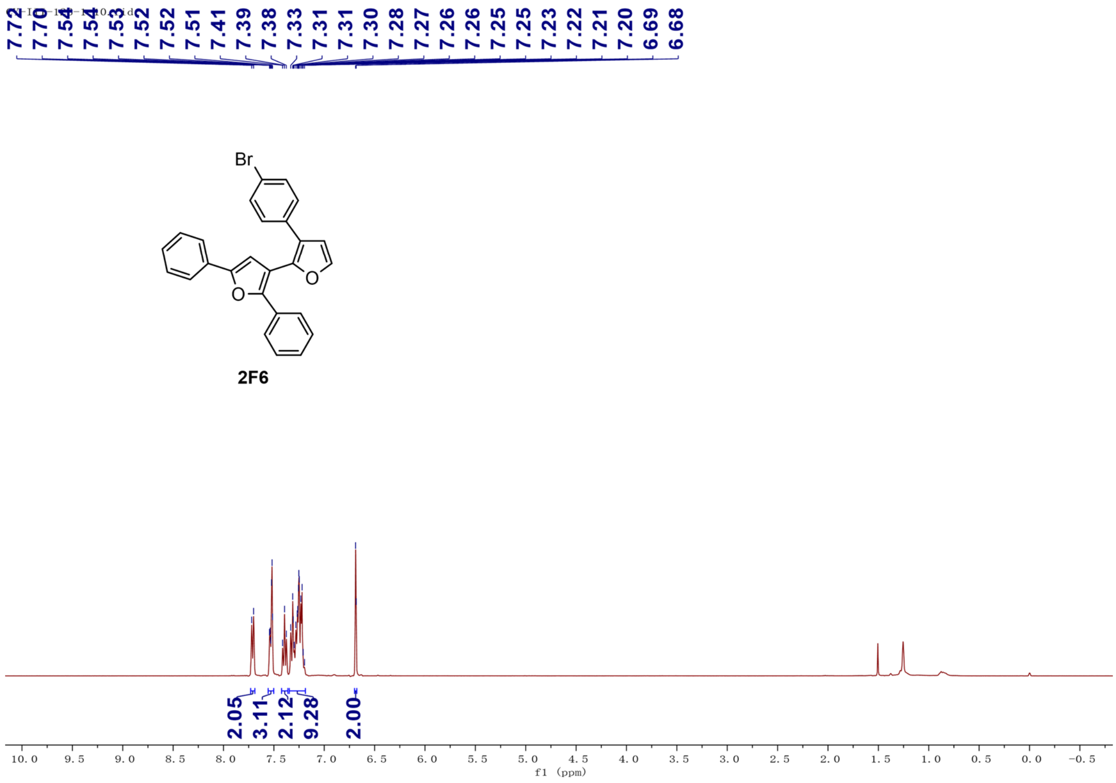

Supplementary Figure 134. ^{1}H NMR (400 MHz, $CDCl_3$) spectra for compound **2F6**

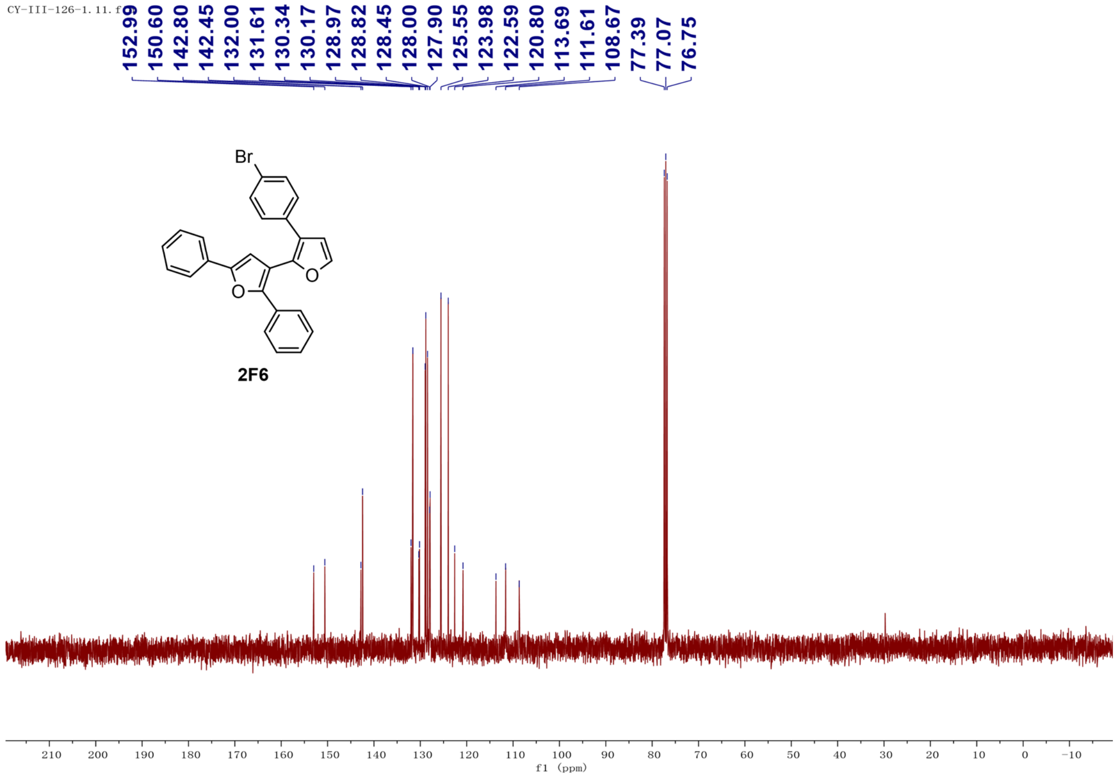

Supplementary Figure 135. ^{13}C NMR (101 MHz, $CDCl_3$) spectra for compound **2F6**

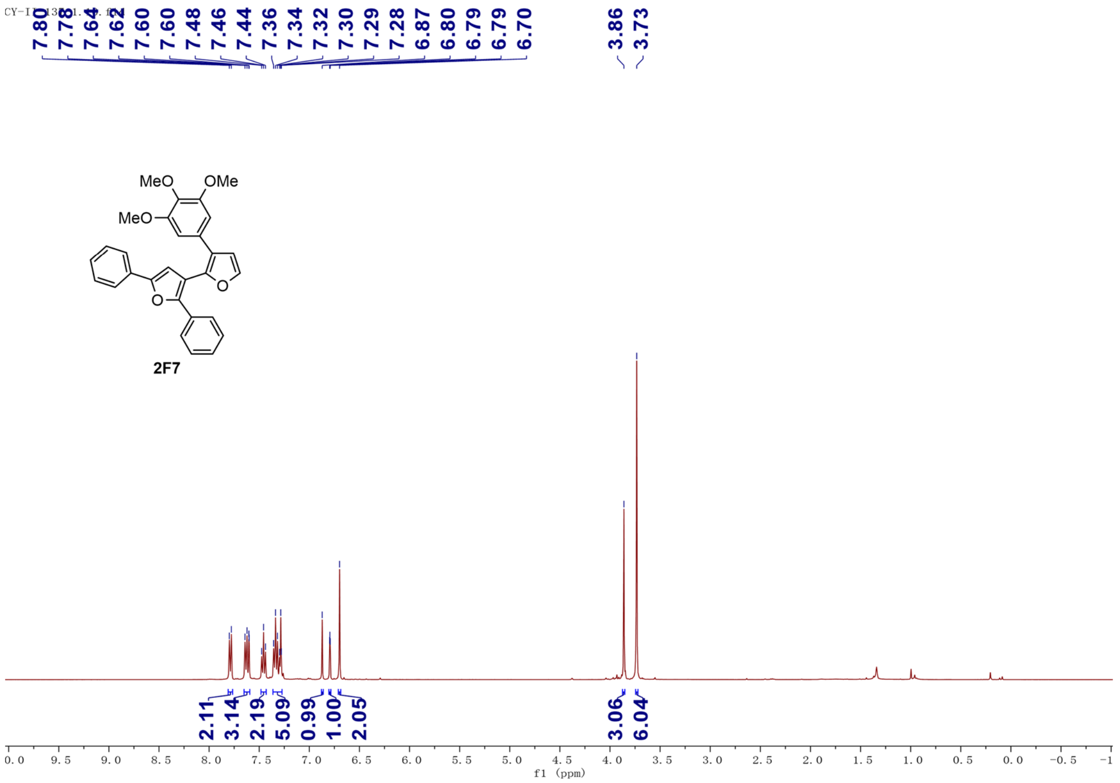

Supplementary Figure 136. ^{1}H NMR (400 MHz, $CDCl_3$) spectra for compound **2F7**

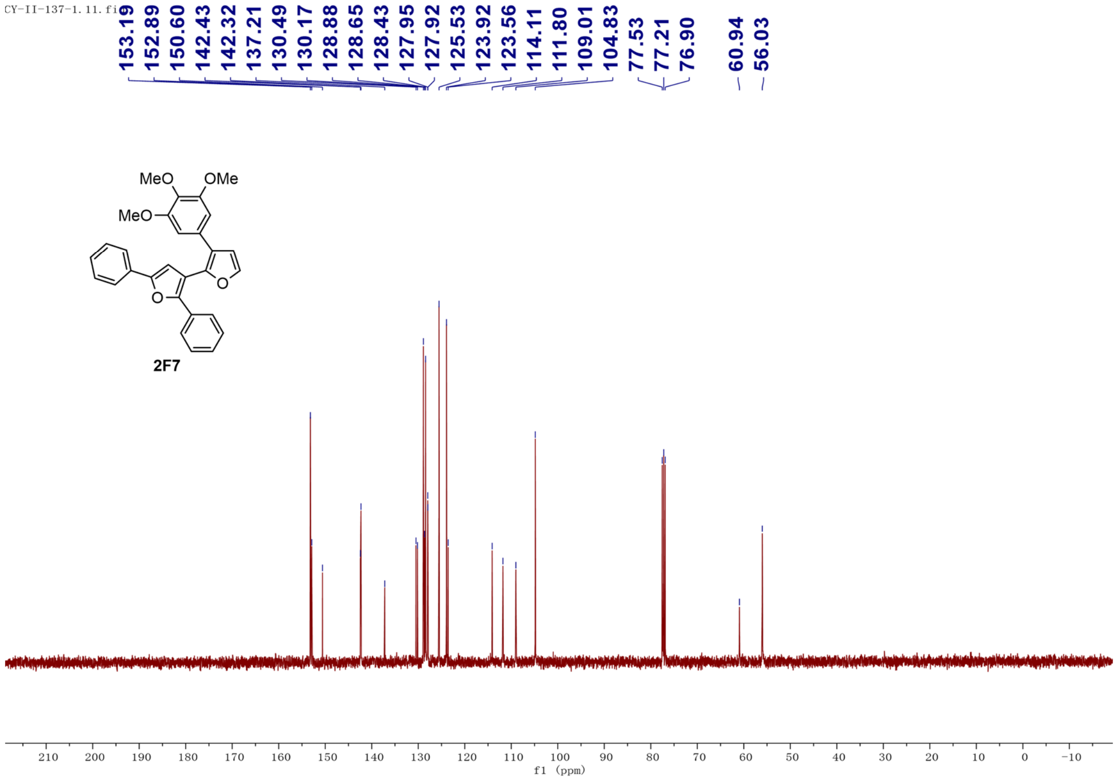

Supplementary Figure 137. ^{13}C NMR (101 MHz, $CDCl_3$) spectra for compound **2F7**

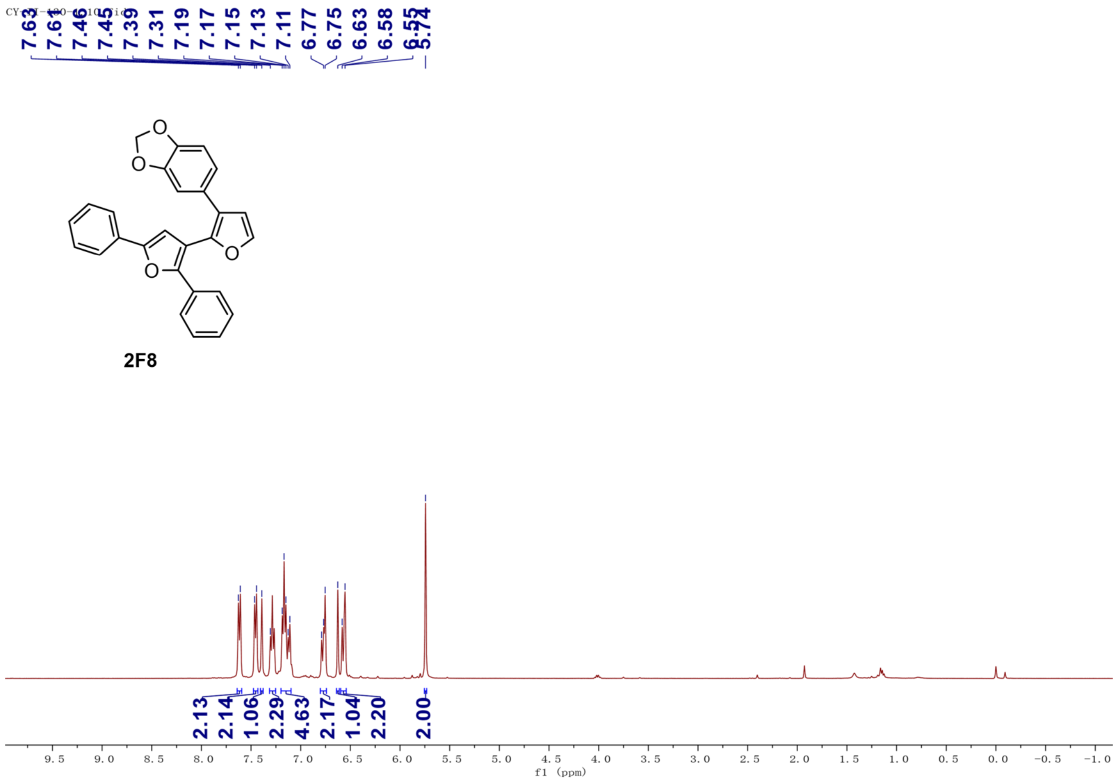

Supplementary Figure 138. ^{1}H NMR (400 MHz, $CDCl_3$) spectra for compound **2F8**

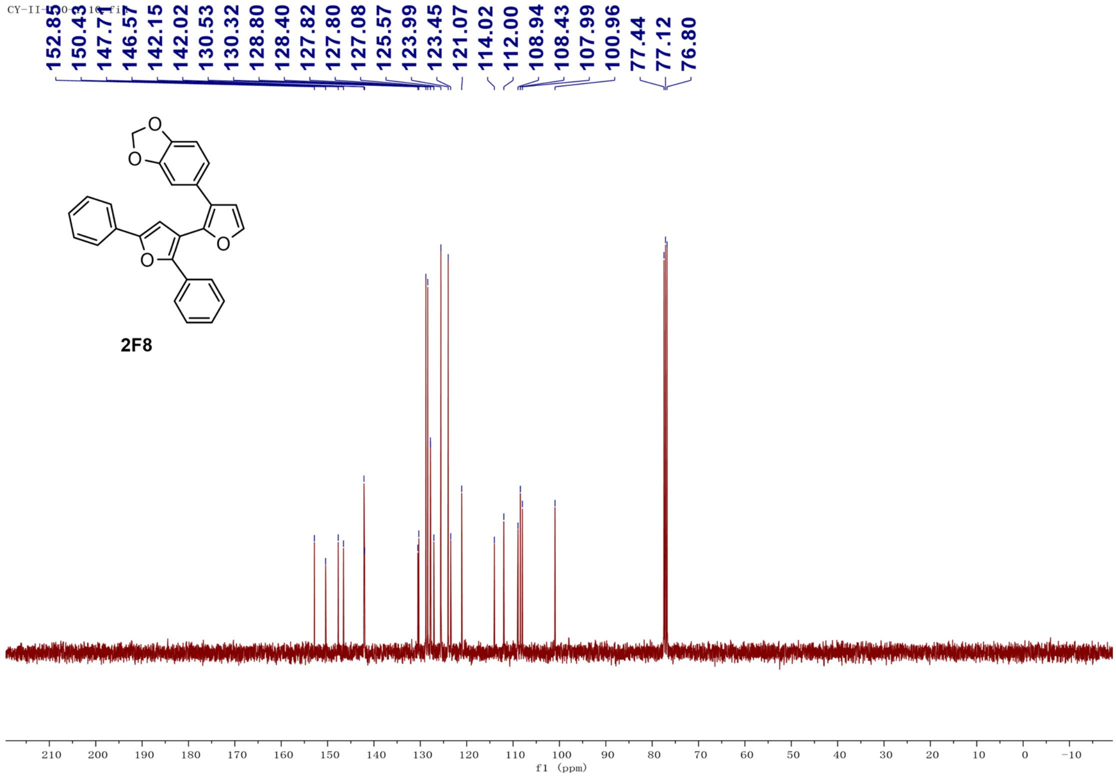

Supplementary Figure 139. ^{13}C NMR (101 MHz, $CDCl_3$) spectra for compound **2F8**

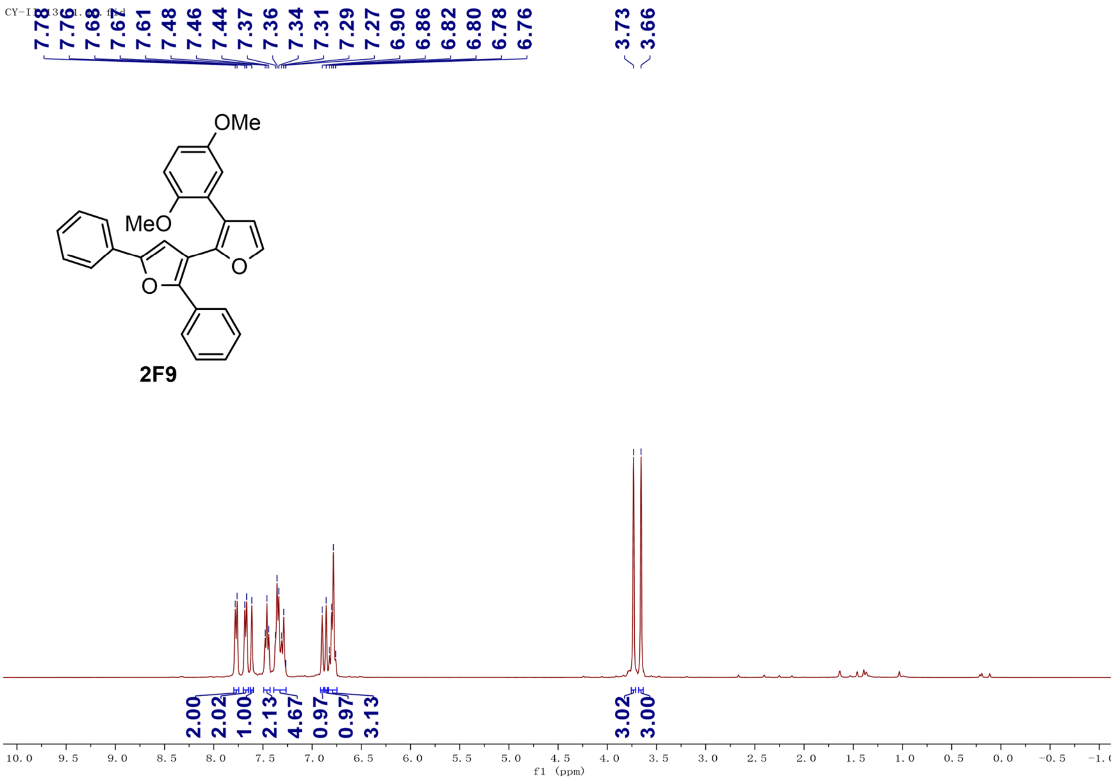

Supplementary Figure 140. ^{1}H NMR (400 MHz, $CDCl_3$) spectra for compound **2F9**

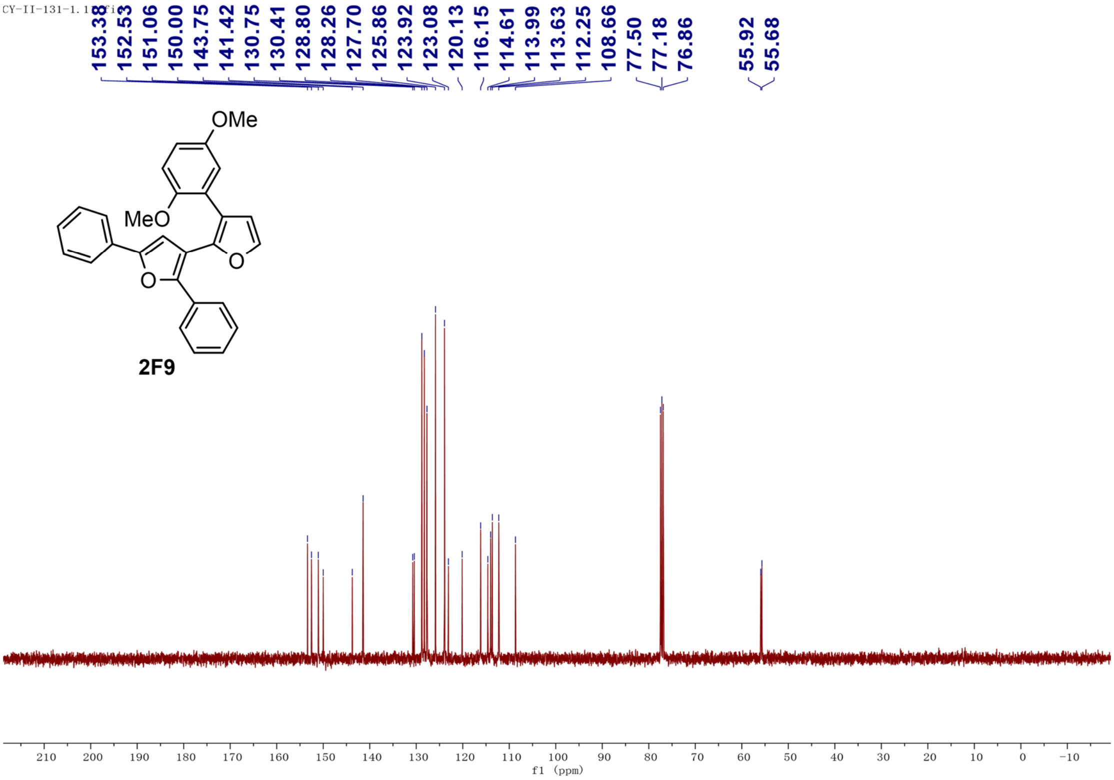

Supplementary Figure 141. ^{13}C NMR (101 MHz, $CDCl_3$) spectra for compound **2F9**

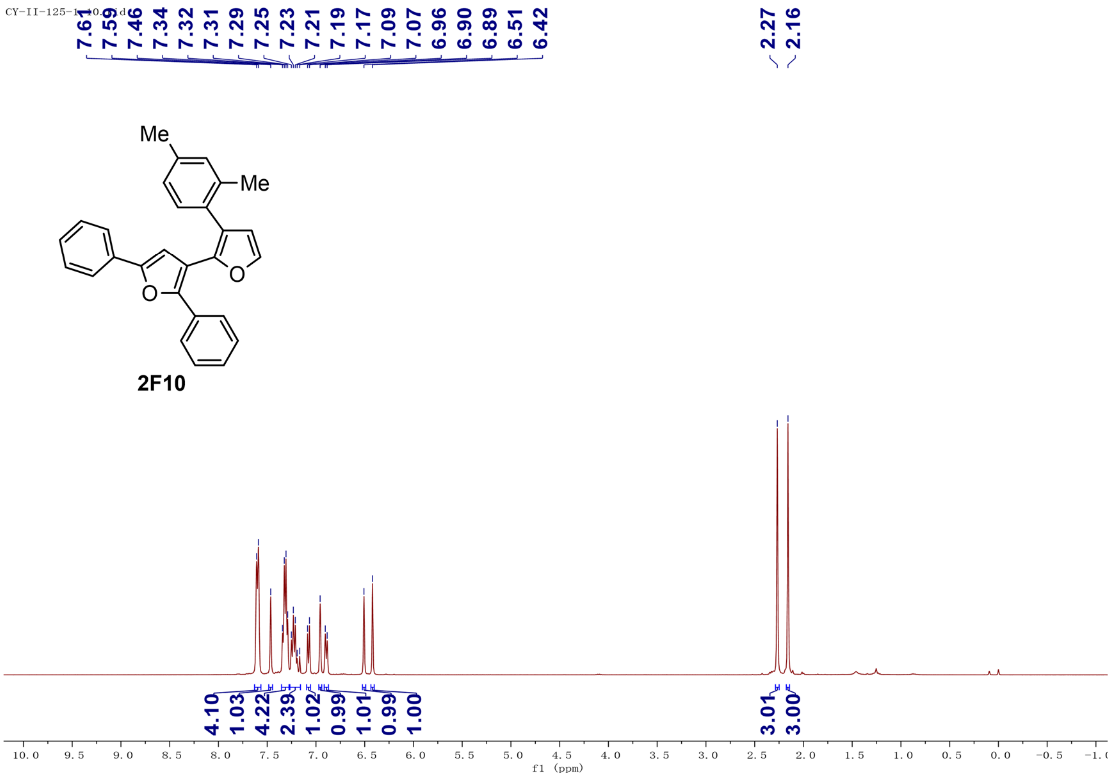

Supplementary Figure 142. ^{1}H NMR (400 MHz, $CDCl_3$) spectra for compound **2F10**

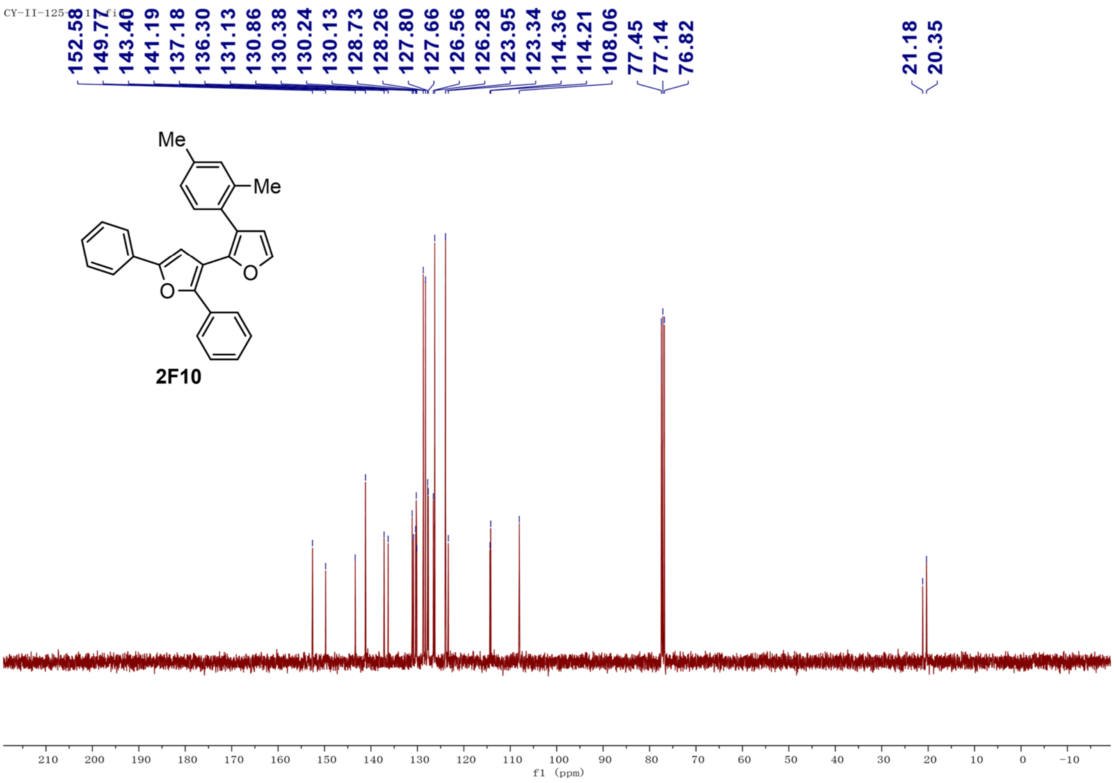

Supplementary Figure 143. ^{13}C NMR (101 MHz, $CDCl_3$) spectra for compound **2F10**

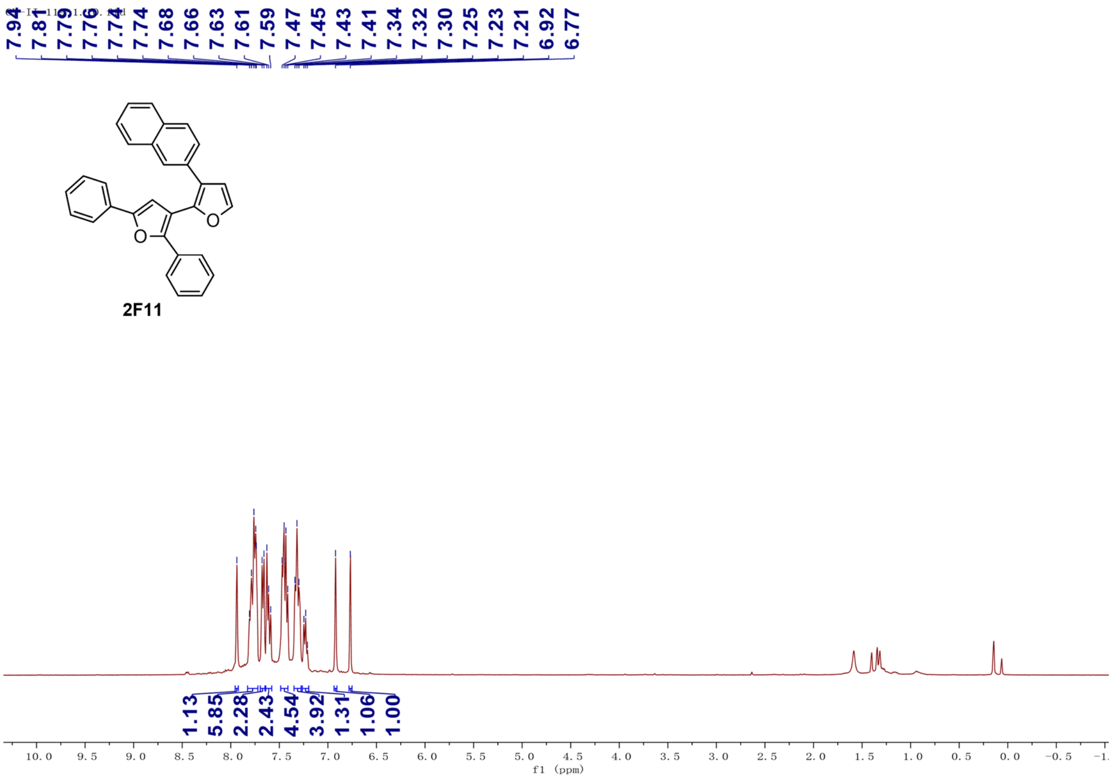

Supplementary Figure 144. ^{1}H NMR (400 MHz, $CDCl_3$) spectra for compound **2F11**

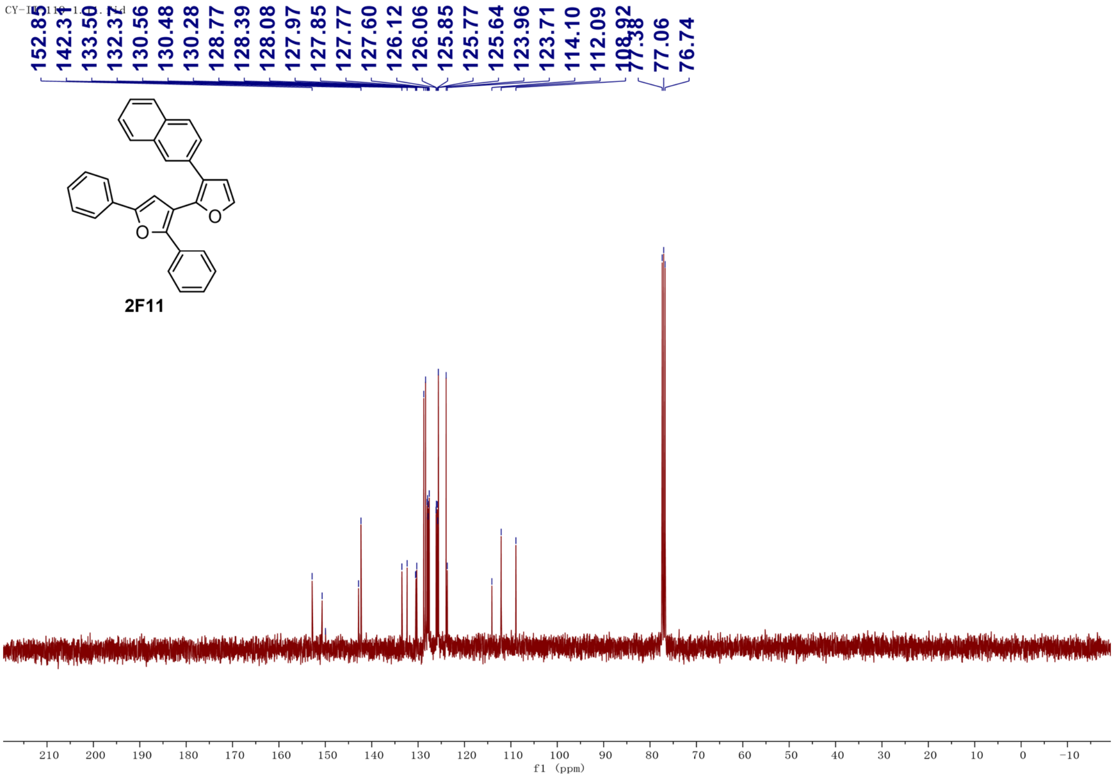

Supplementary Figure 145. ^{13}C NMR (101 MHz, $CDCl_3$) spectra for compound **2F11**

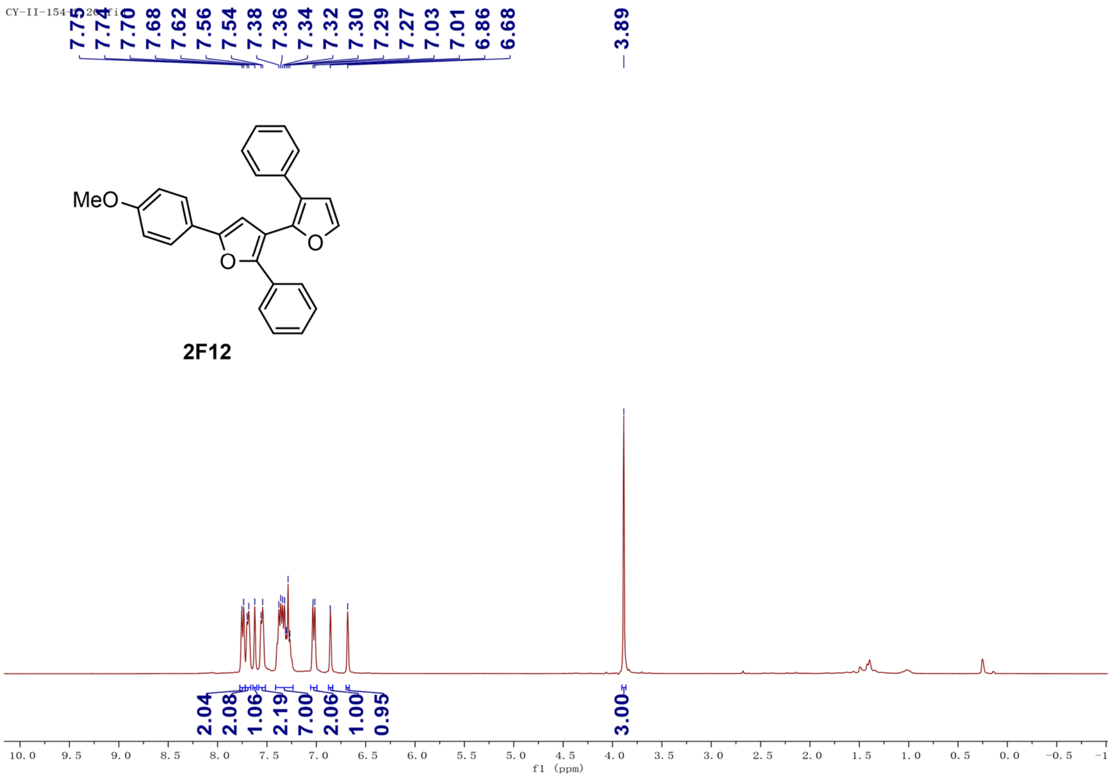

Supplementary Figure 146. ^{1}H NMR (400 MHz, $CDCl_3$) spectra for compound **2F12**

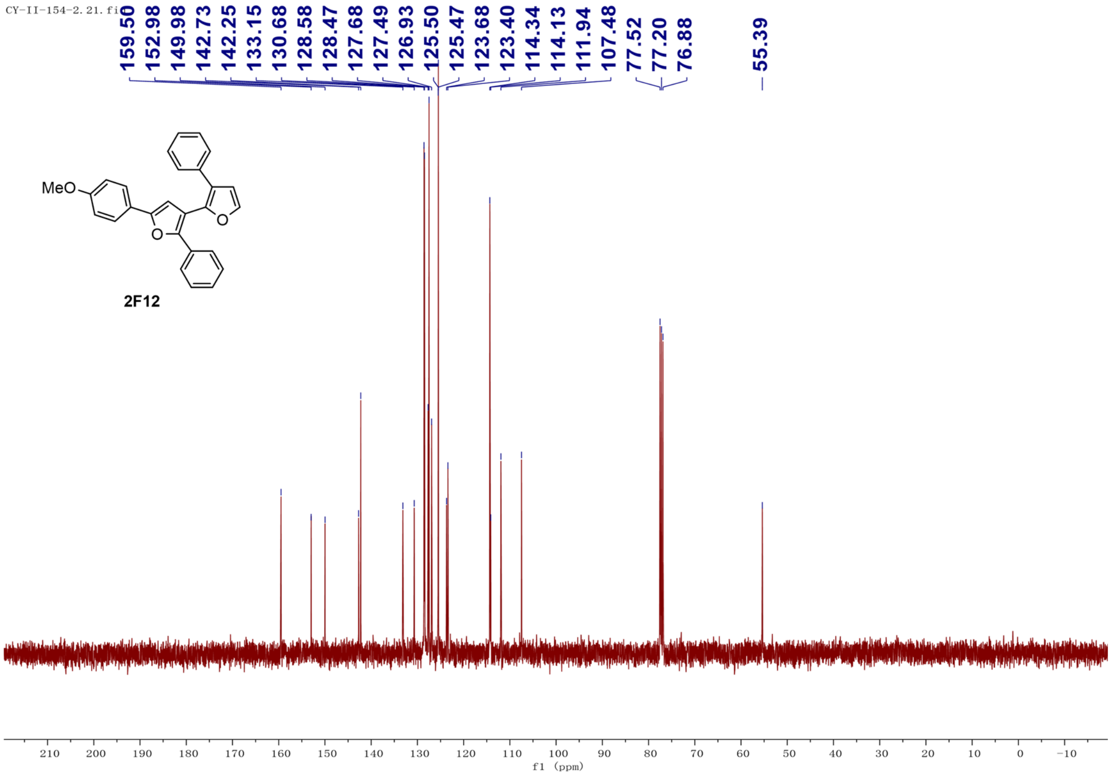

Supplementary Figure 147. ^{13}C NMR (101 MHz, $CDCl_3$) spectra for compound **2F12**

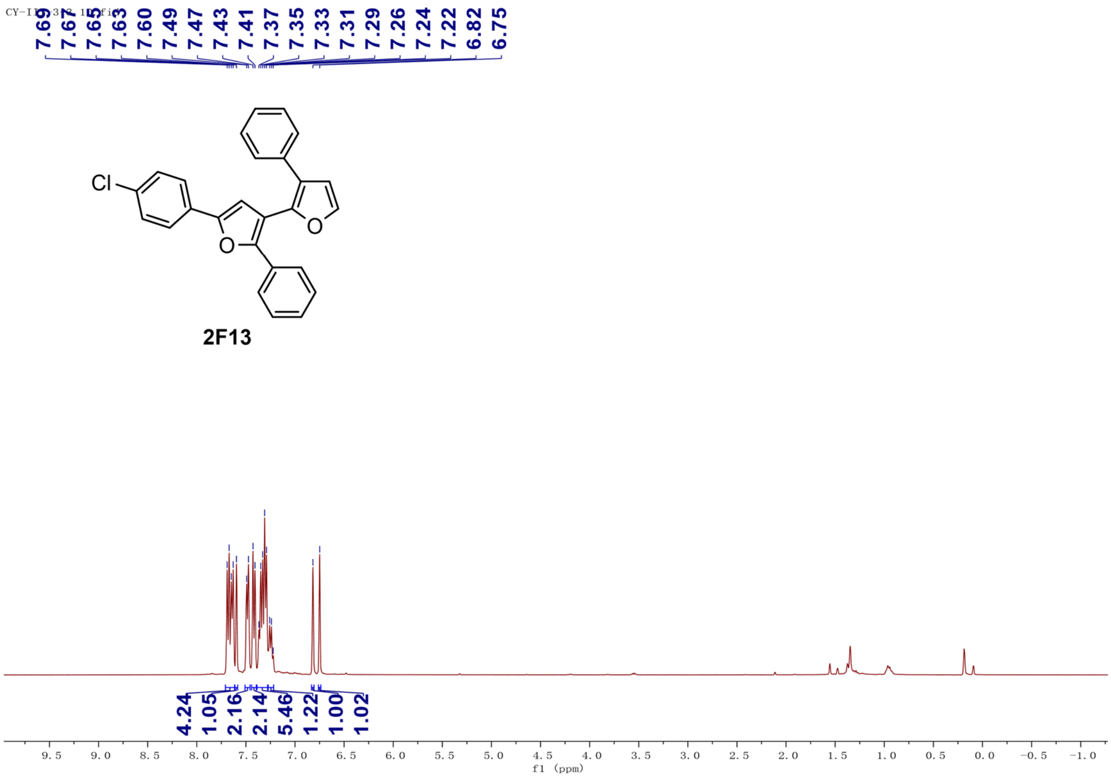

Supplementary Figure 148. ^{1}H NMR (400 MHz, $CDCl_3$) spectra for compound **2F13**

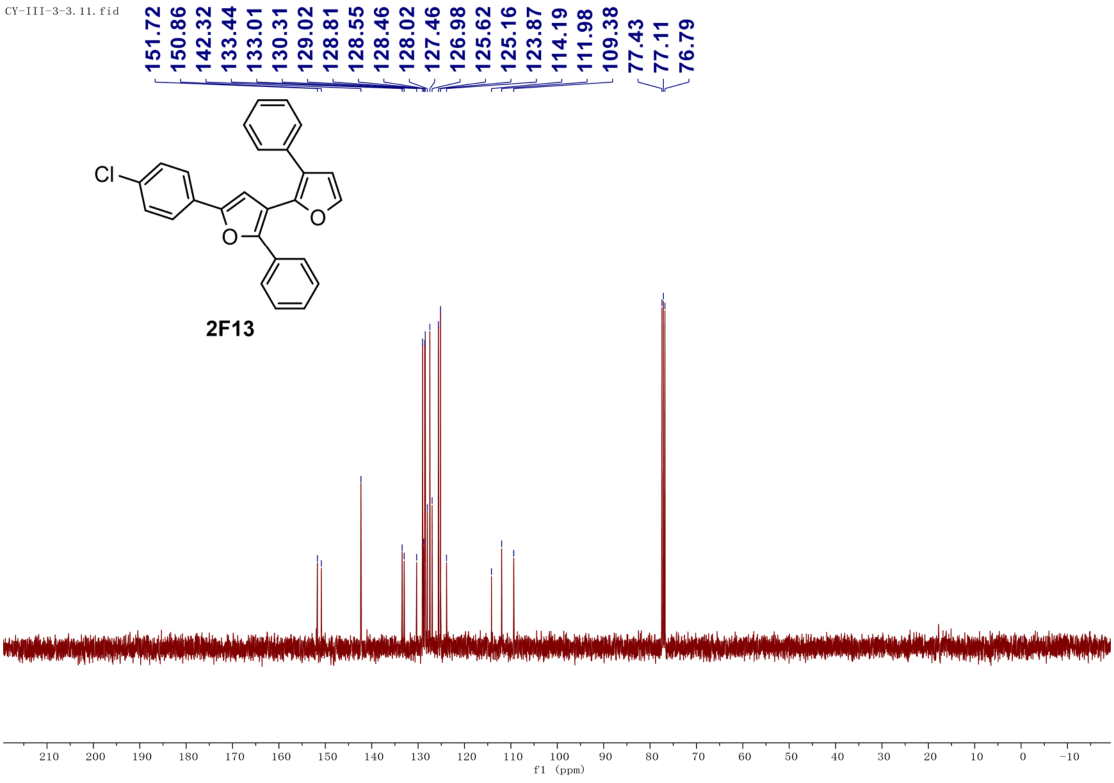

Supplementary Figure 149. ^{13}C NMR (101 MHz, $CDCl_3$) spectra for compound **2F13**

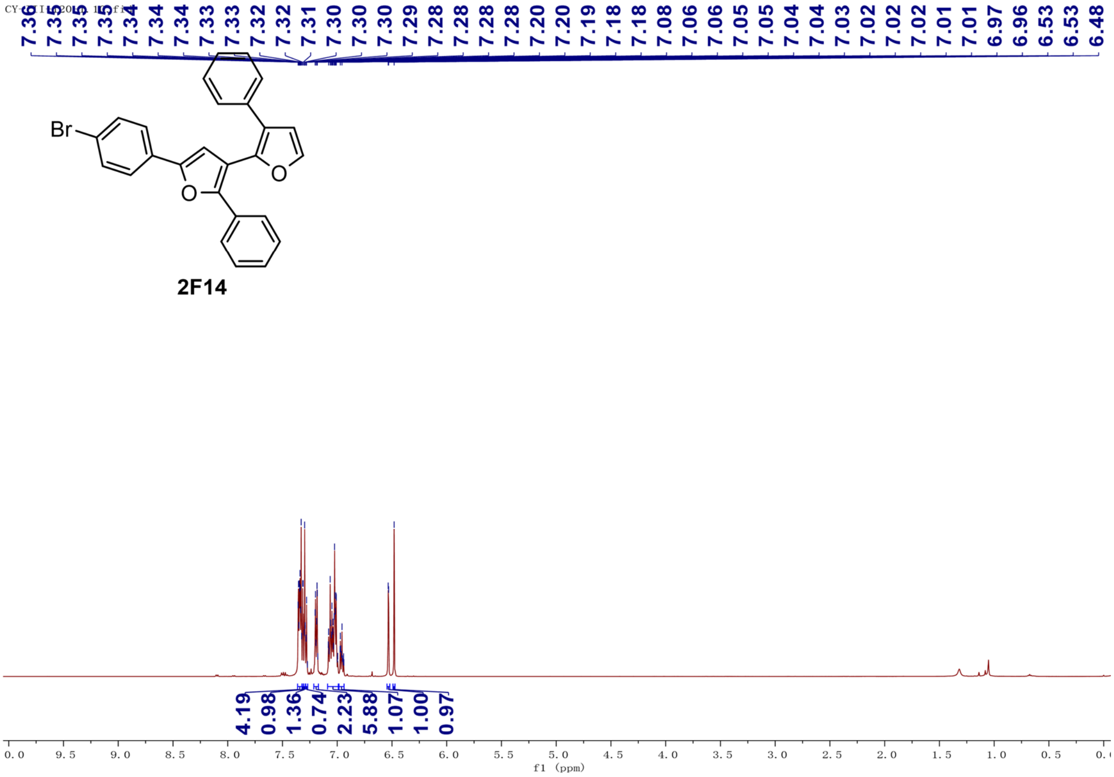

Supplementary Figure 150. ^{1}H NMR (500 MHz, $CDCl_3$) spectra for compound **2F14**

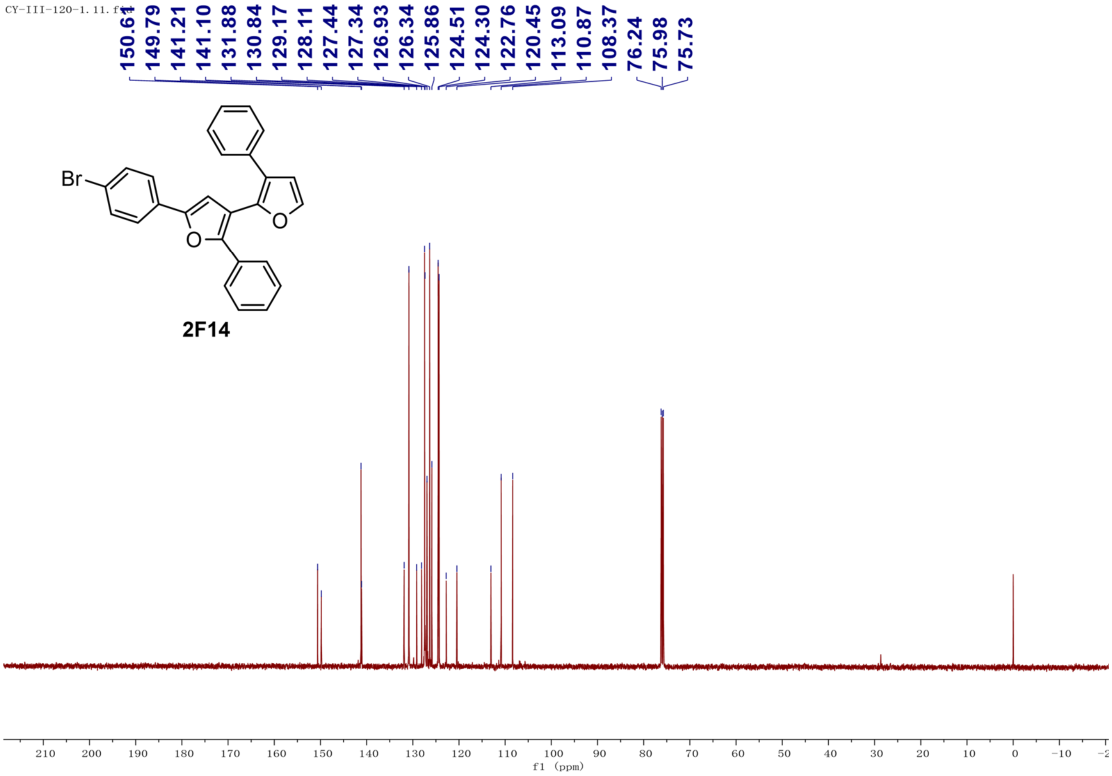

Supplementary Figure 151. ^{13}C NMR (126 MHz, $CDCl_3$) spectra for compound **2F14**

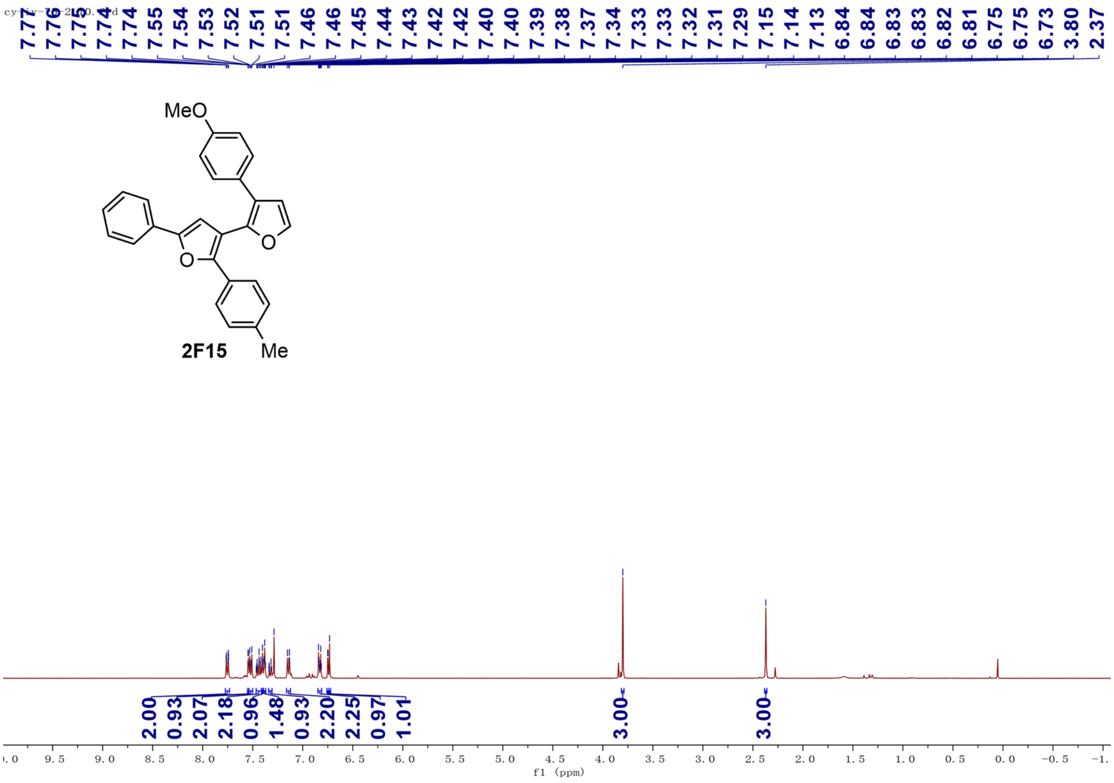

Supplementary Figure 152. ^{1}H NMR (400 MHz, $CDCl_3$) spectra for compound **2F15**

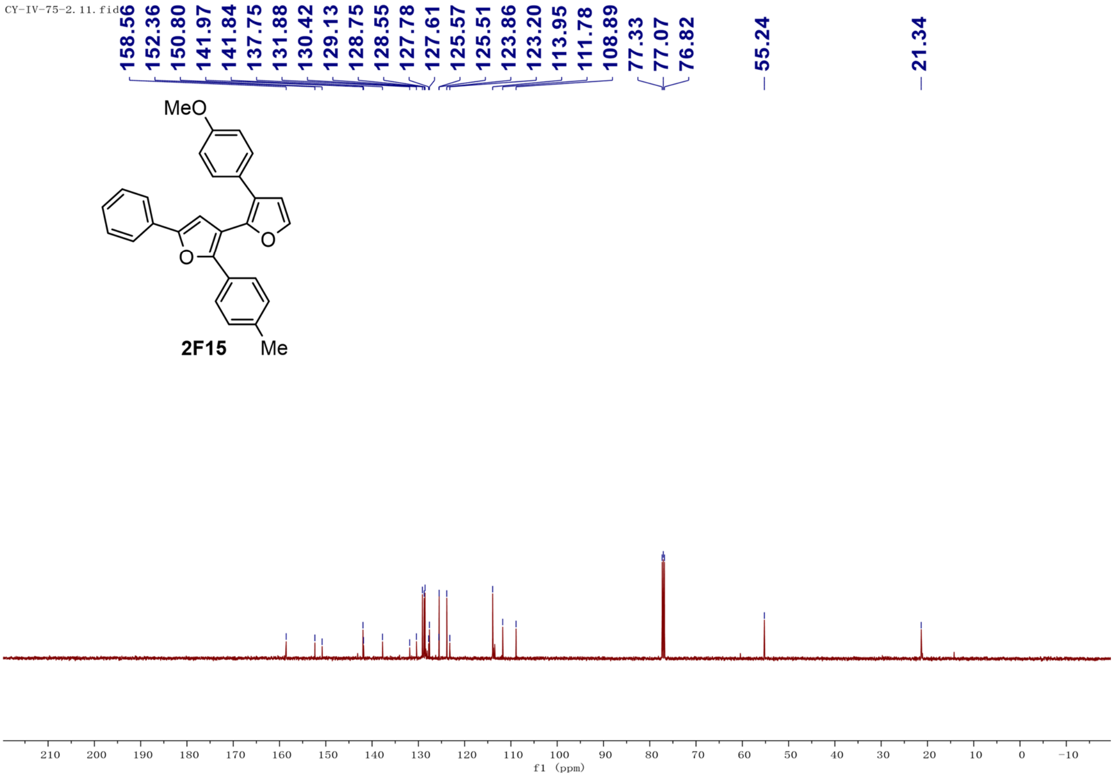

Supplementary Figure 153. ^{13}C NMR (101 MHz, $CDCl_3$) spectra for compound **2F15**

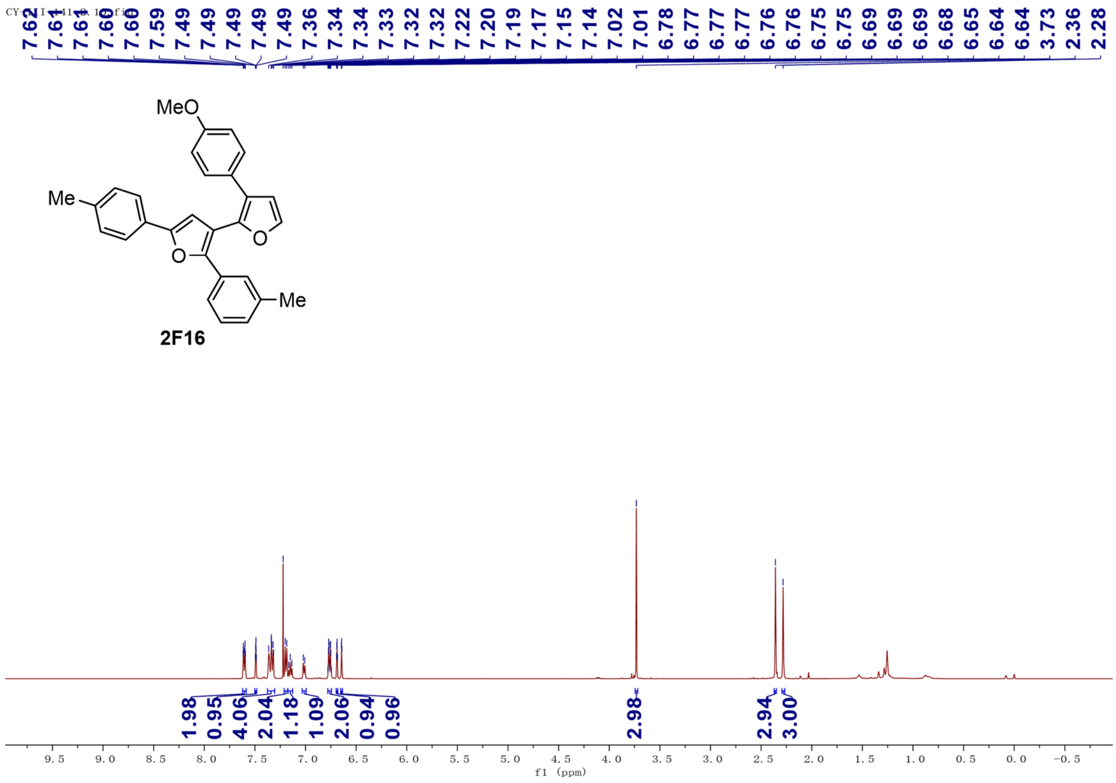

Supplementary Figure 154. ^{1}H NMR (500 MHz, $CDCl_3$) spectra for compound **2F16**

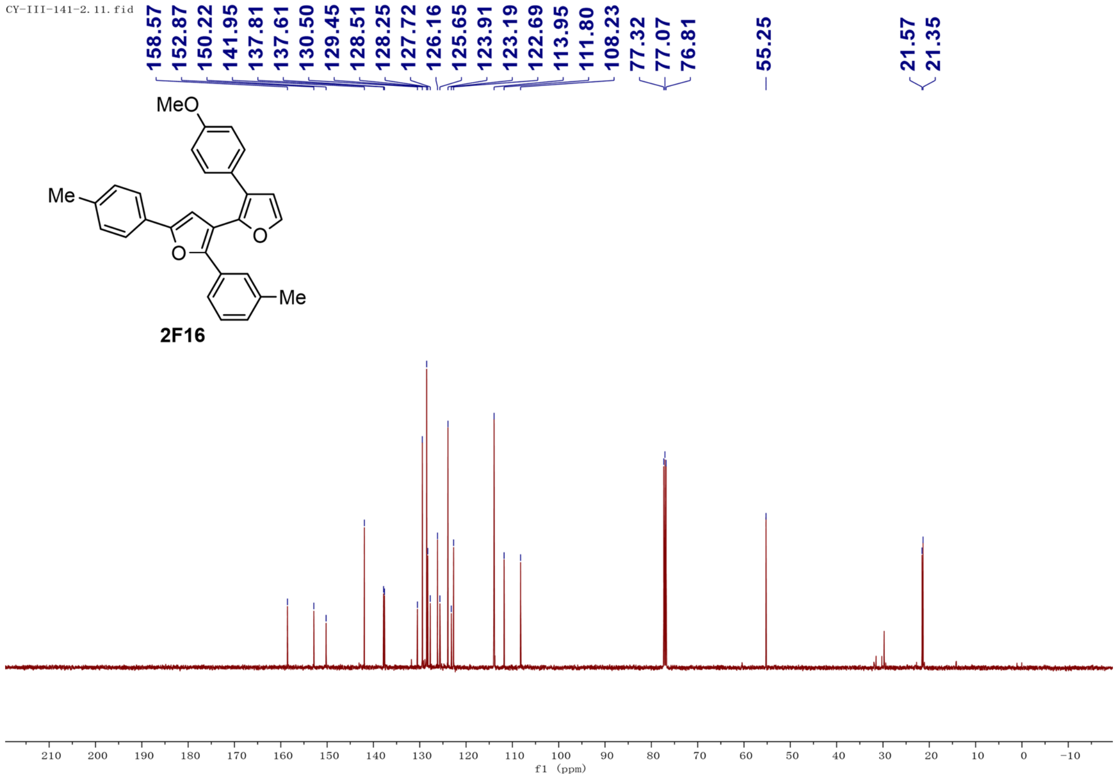

Supplementary Figure 155. ^{13}C NMR (126 MHz, $CDCl_3$) spectra for compound **2F16**

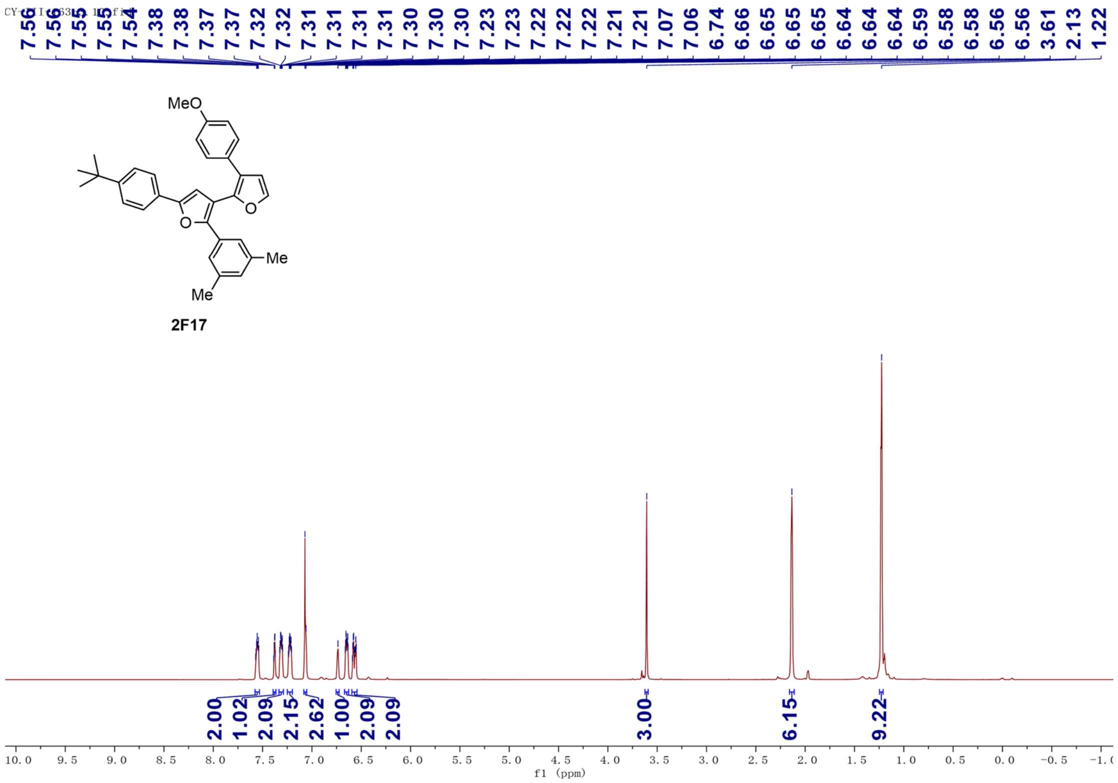

Supplementary Figure 156. ^{1}H NMR (500 MHz, $CDCl_3$) spectra for compound **2F17**

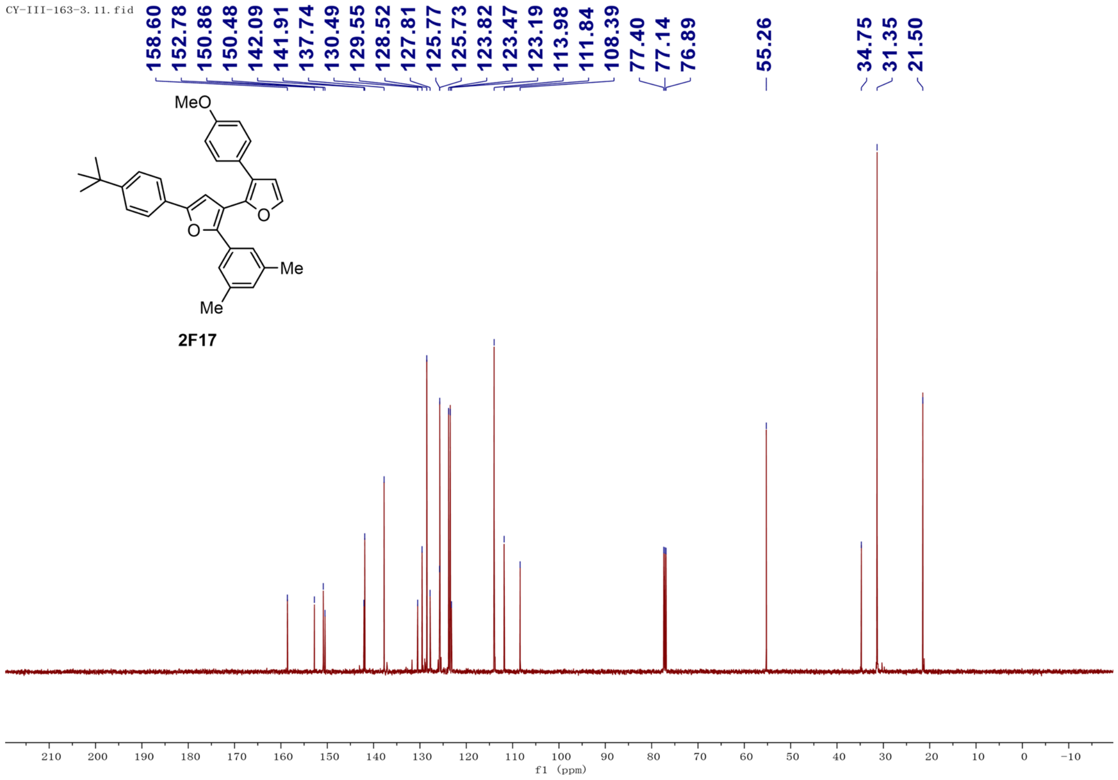

Supplementary Figure 157. ^{13}C NMR (126 MHz, $CDCl_3$) spectra for compound **2F17**

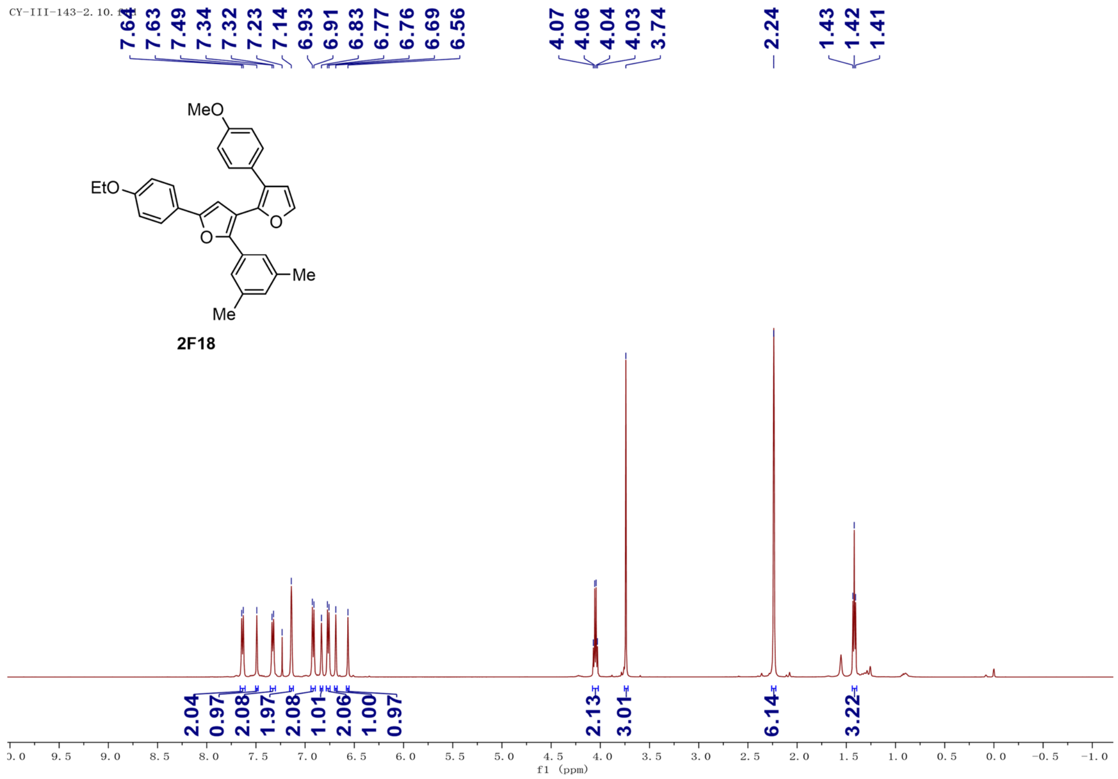

Supplementary Figure 158. ^{1}H NMR (500 MHz, $CDCl_3$) spectra for compound **2F18**

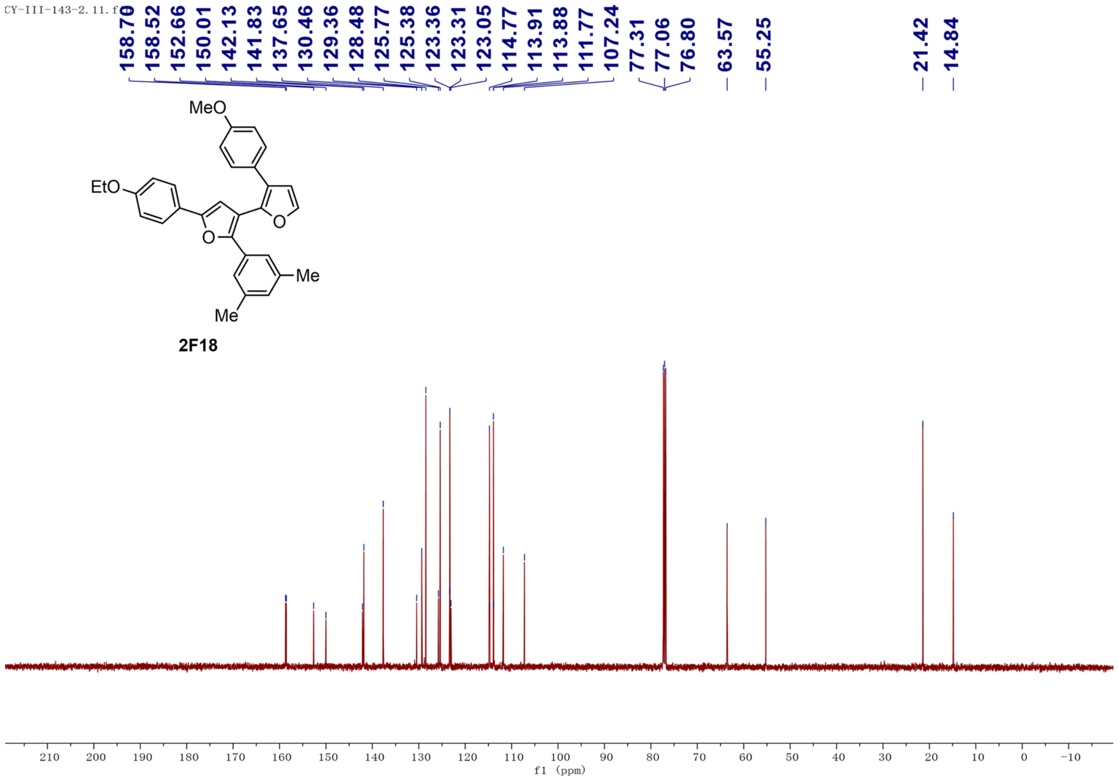

Supplementary Figure 159. ^{13}C NMR (126 MHz, $CDCl_3$) spectra for compound **2F18**

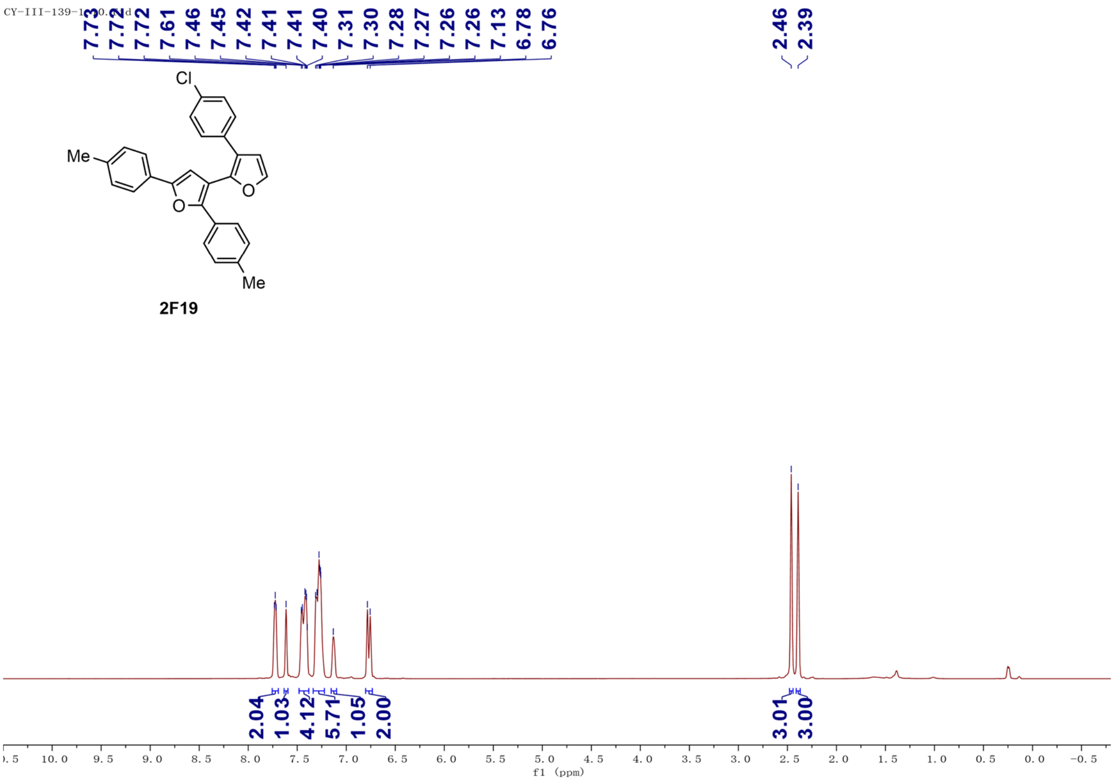

Supplementary Figure 160. ^{1}H NMR (500 MHz, $CDCl_3$) spectra for compound **2F19**

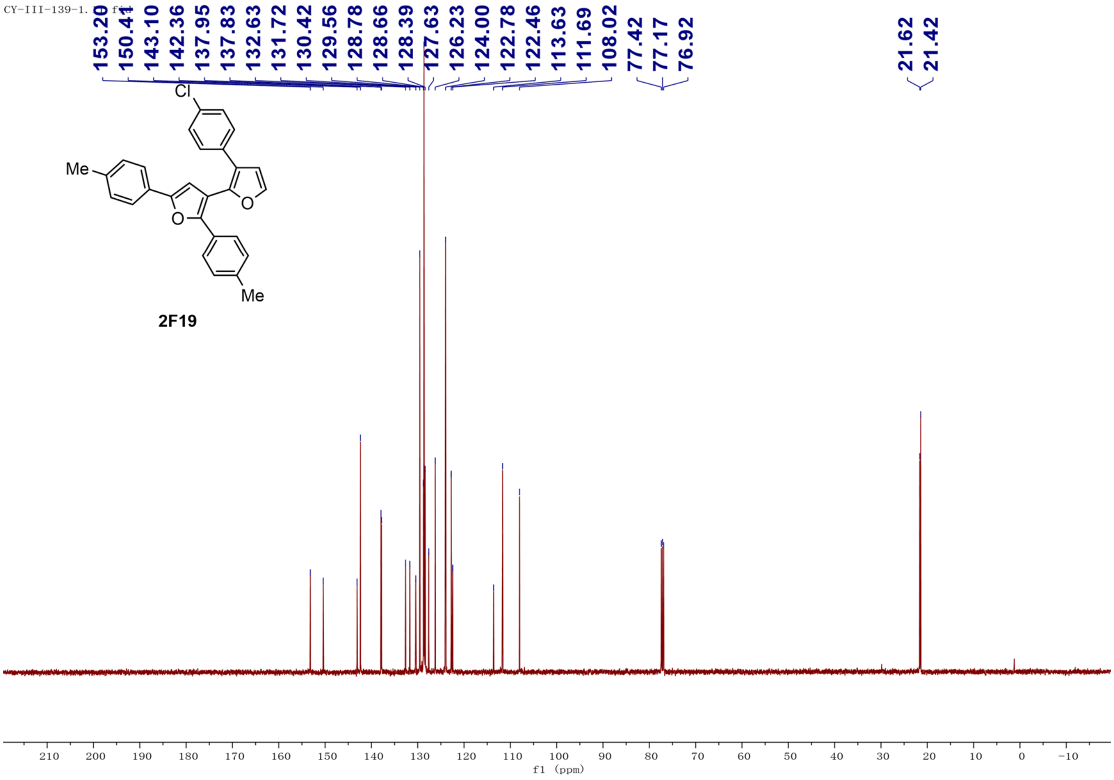

Supplementary Figure 161. ^{13}C NMR (126 MHz, $CDCl_3$) spectra for compound **2F19**

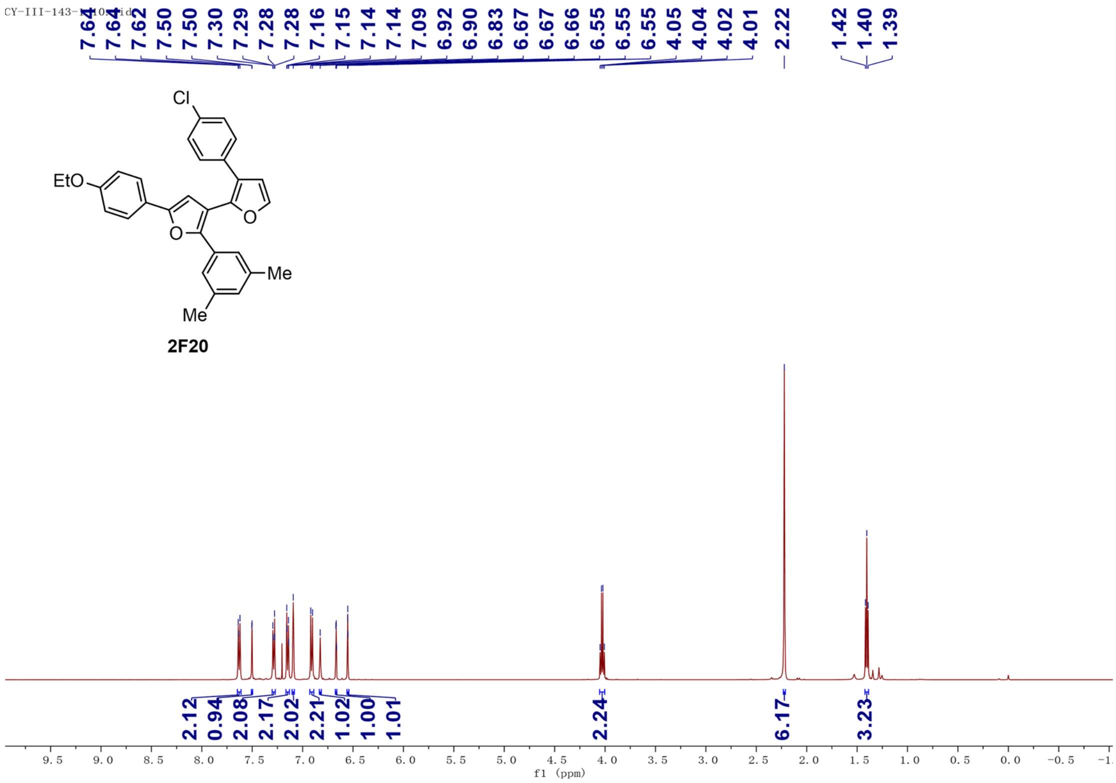

Supplementary Figure 162. ^{1}H NMR (500 MHz, $CDCl_3$) spectra for compound **2F20**

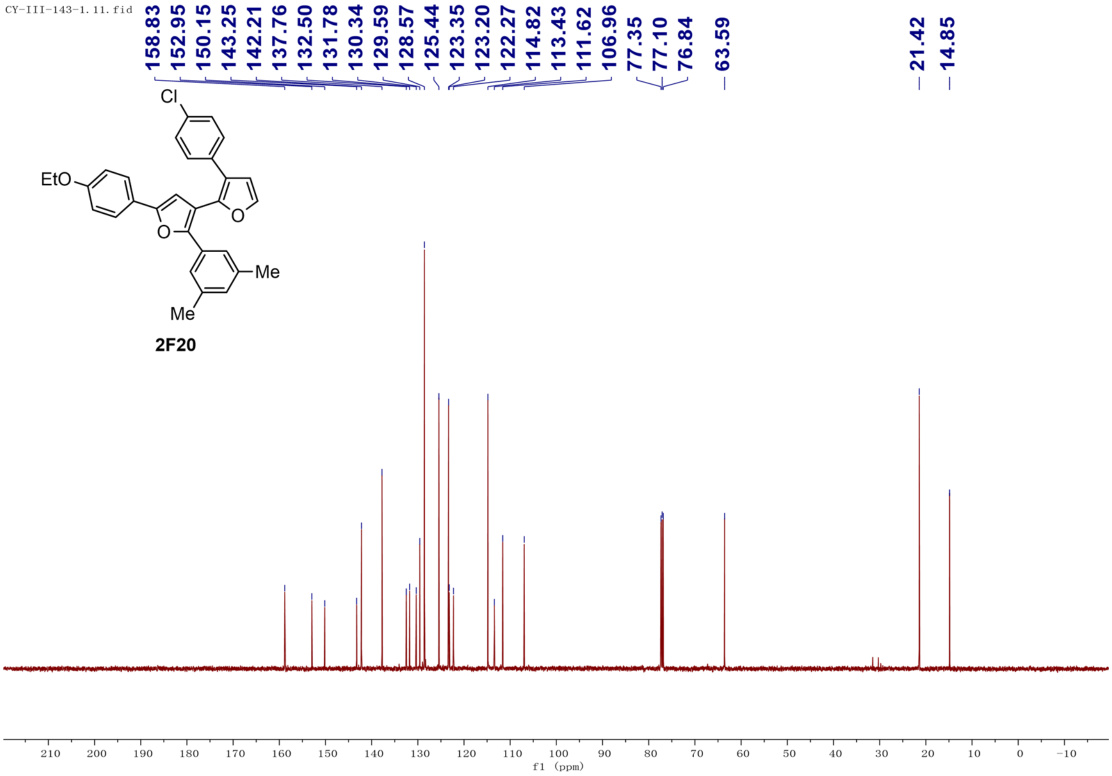

Supplementary Figure 163. ^{13}C NMR (126 MHz, $CDCl_3$) spectra for compound **2F20**

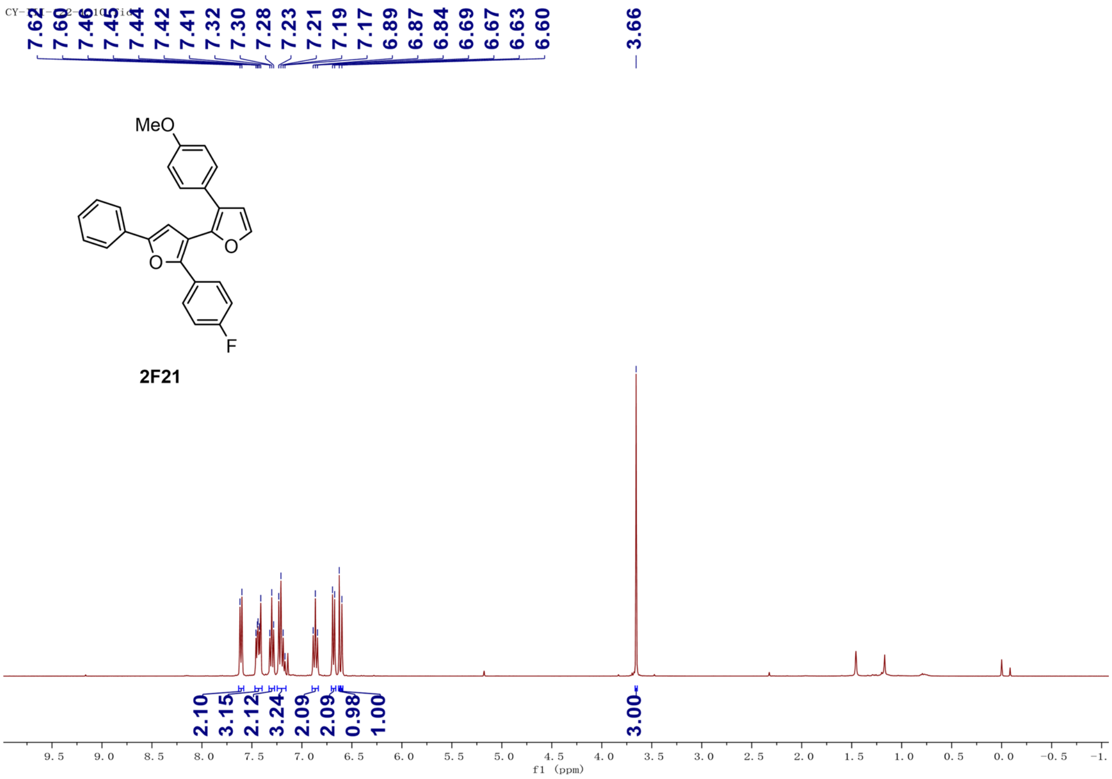

Supplementary Figure 164. ^{1}H NMR (400 MHz, $CDCl_3$) spectra for compound **2F21**

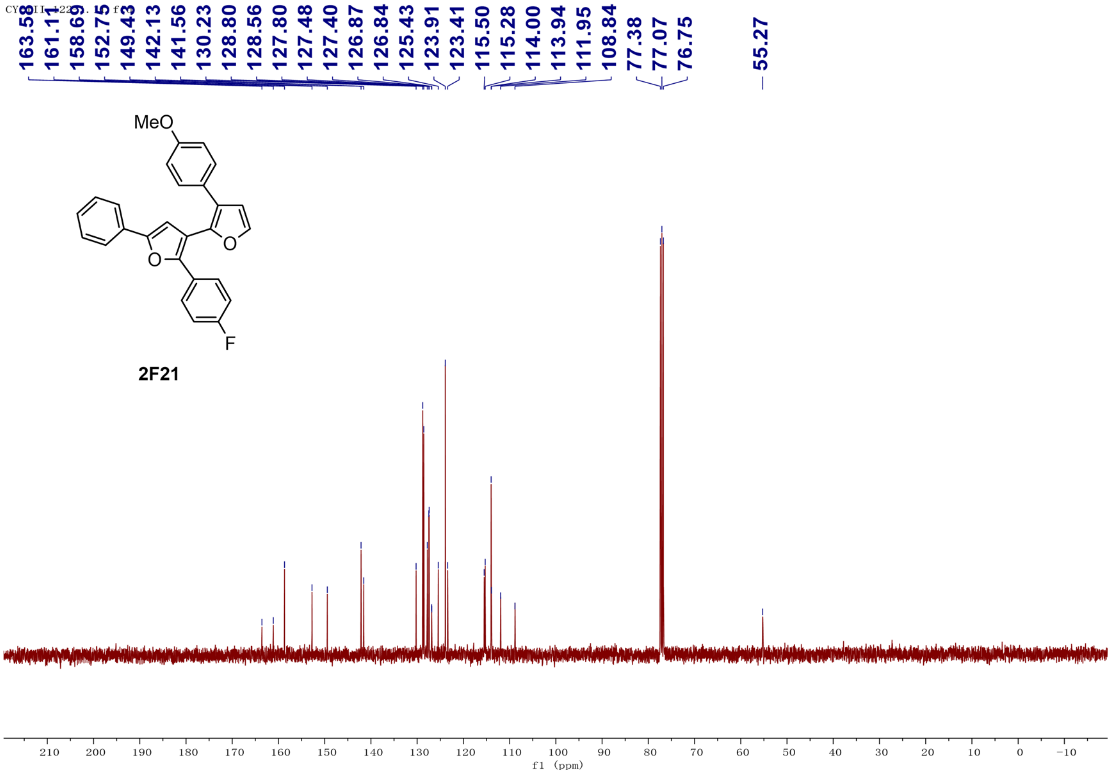

Supplementary Figure 165. ^{13}C NMR (101 MHz, $CDCl_3$) spectra for compound **2F21**

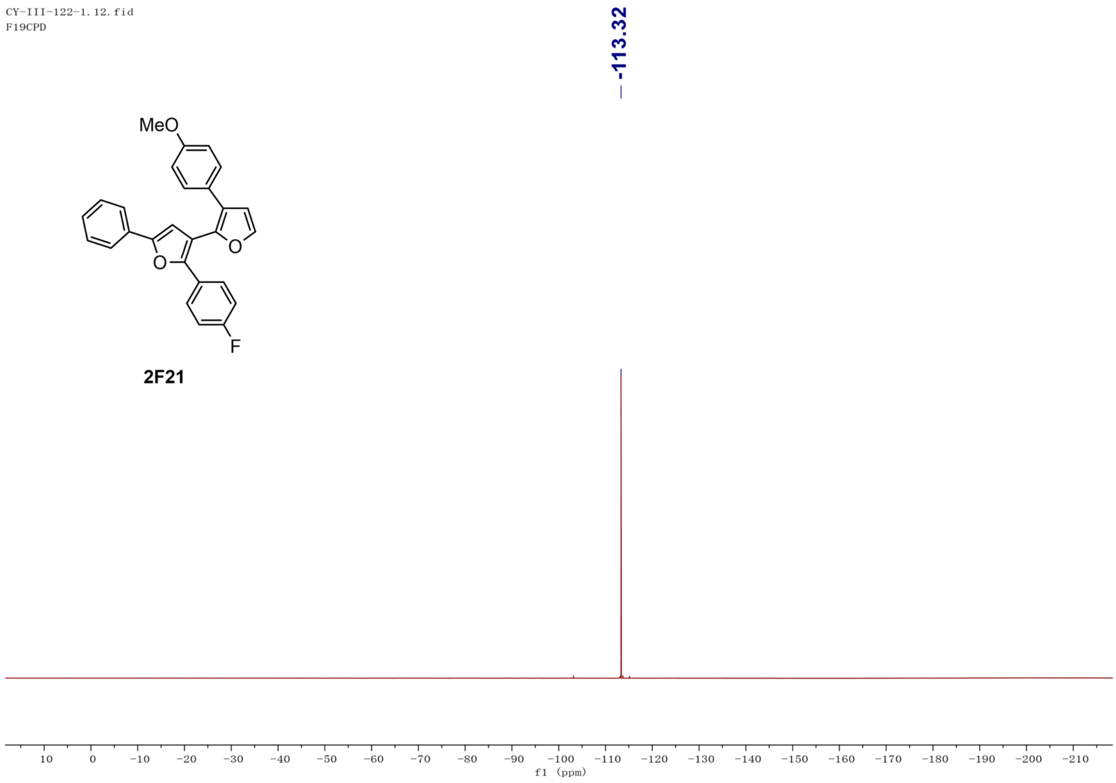

Supplementary Figure 166. ^{19}F NMR (376 MHz, $CDCl_3$) spectra for compound **2F21**

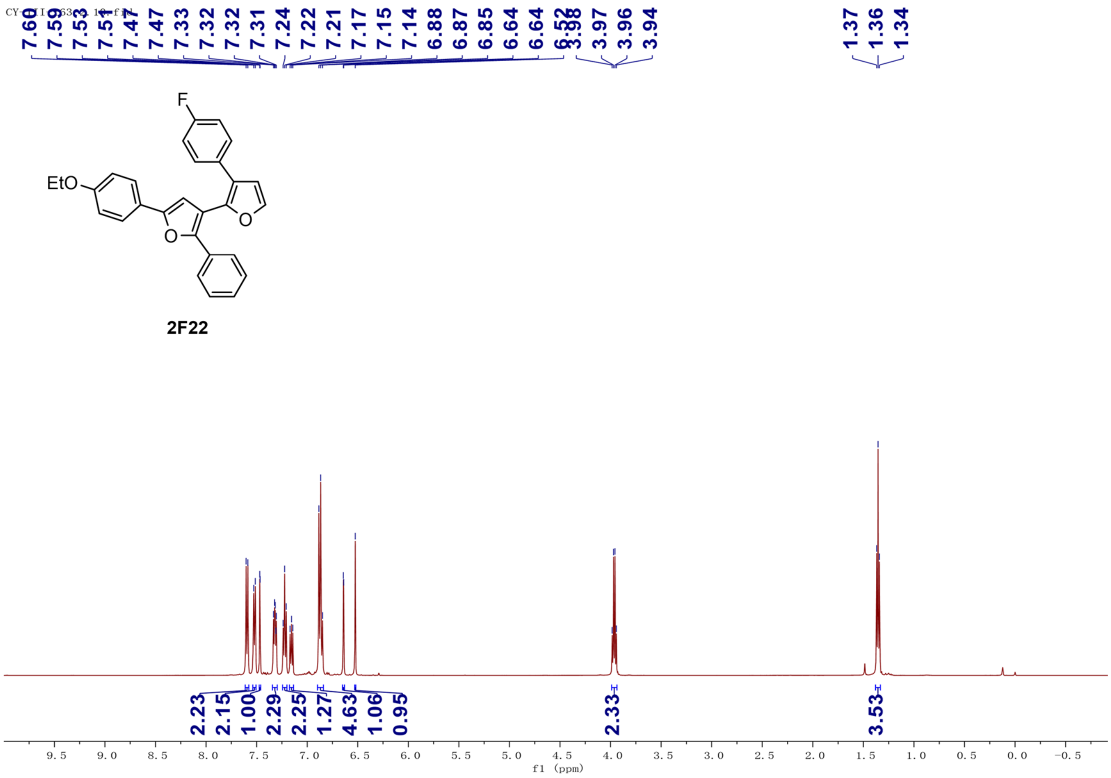

Supplementary Figure 167. ^{1}H NMR (500 MHz, $CDCl_3$) spectra for compound **2F22**

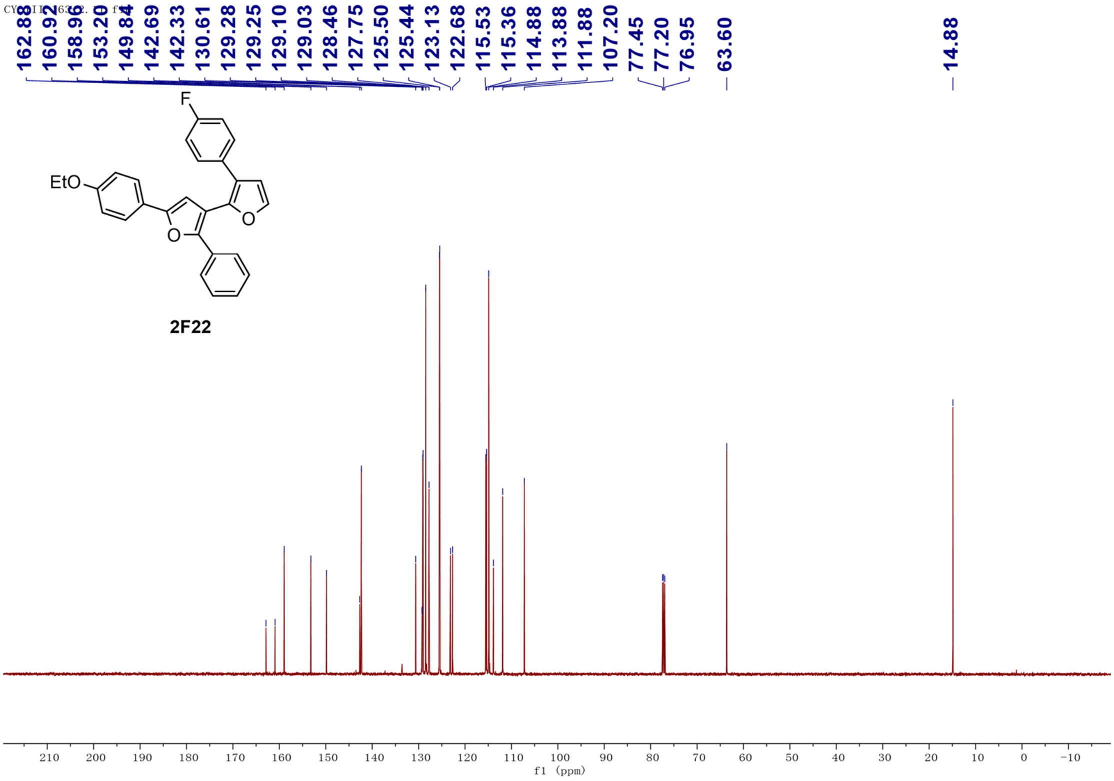

Supplementary Figure 168. ^{13}C NMR (126 MHz, $CDCl_3$) spectra for compound **2F22**

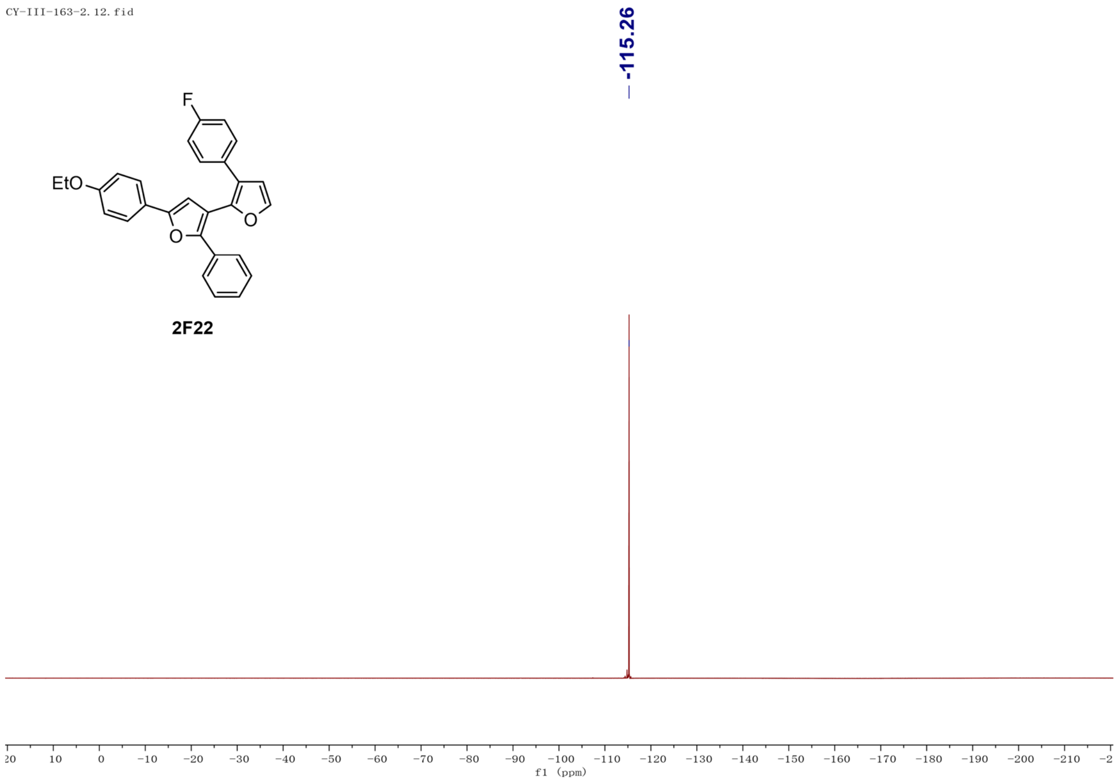

Supplementary Figure 169. ^{19}F NMR (471 MHz, $CDCl_3$) spectra for compound **2F22**

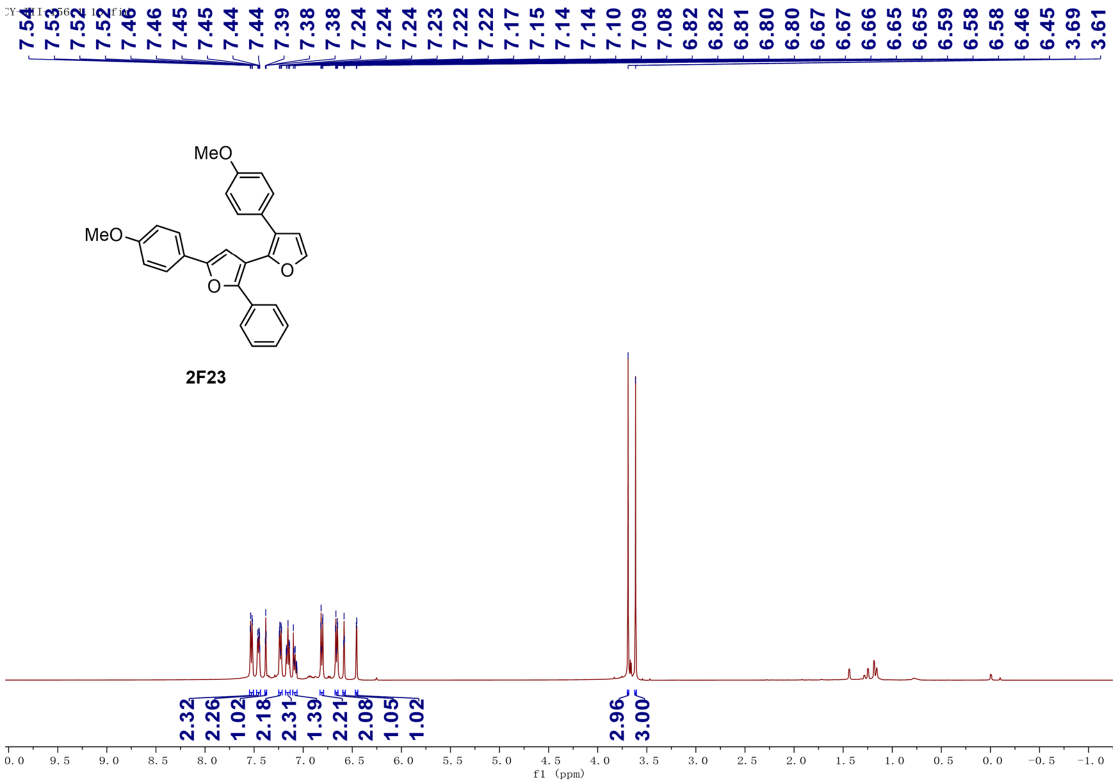

Supplementary Figure 170. ^{1}H NMR (500 MHz, $CDCl_3$) spectra for compound **2F23**

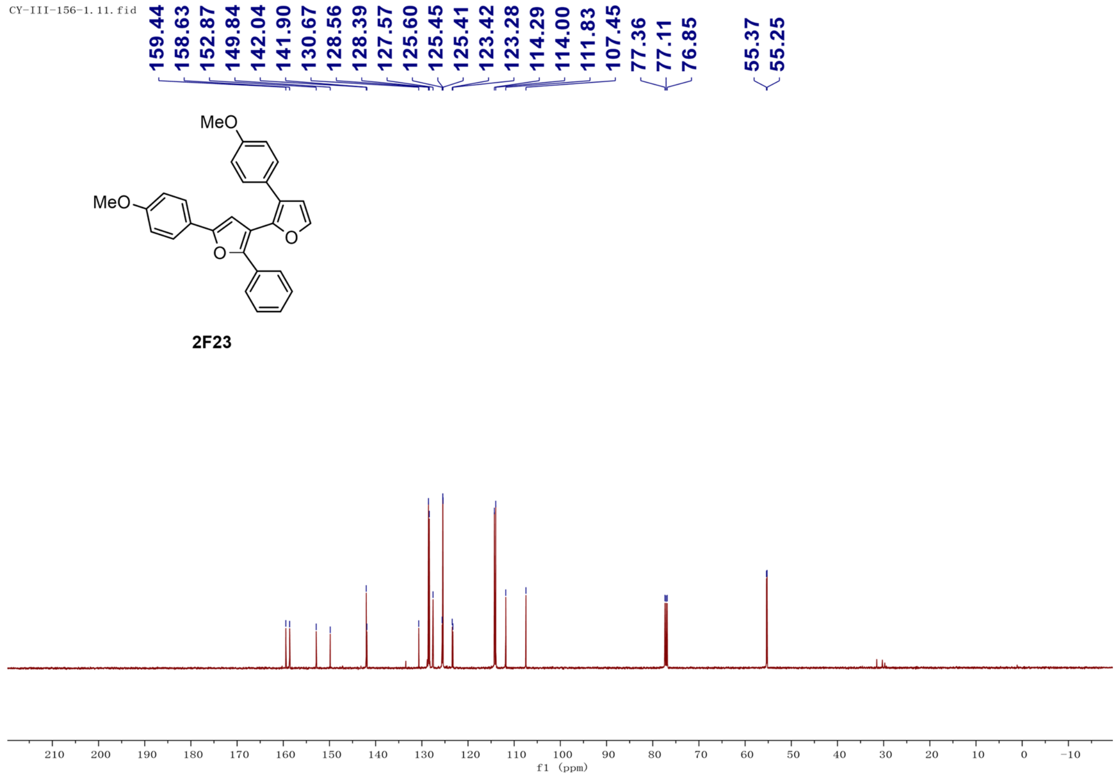

Supplementary Figure 171. ^{13}C NMR (126 MHz, $CDCl_3$) spectra for compound **2F23**

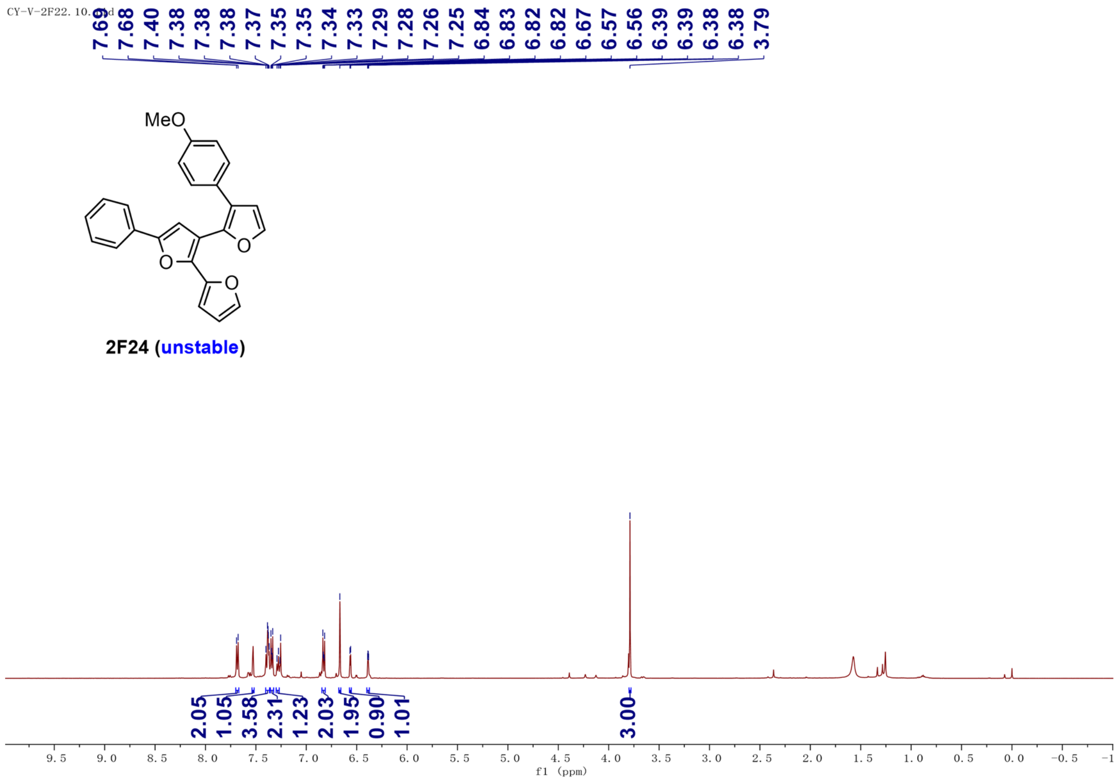

Supplementary Figure 172. ^{1}H NMR (500 MHz, $CDCl_3$) spectra for compound **2F24**

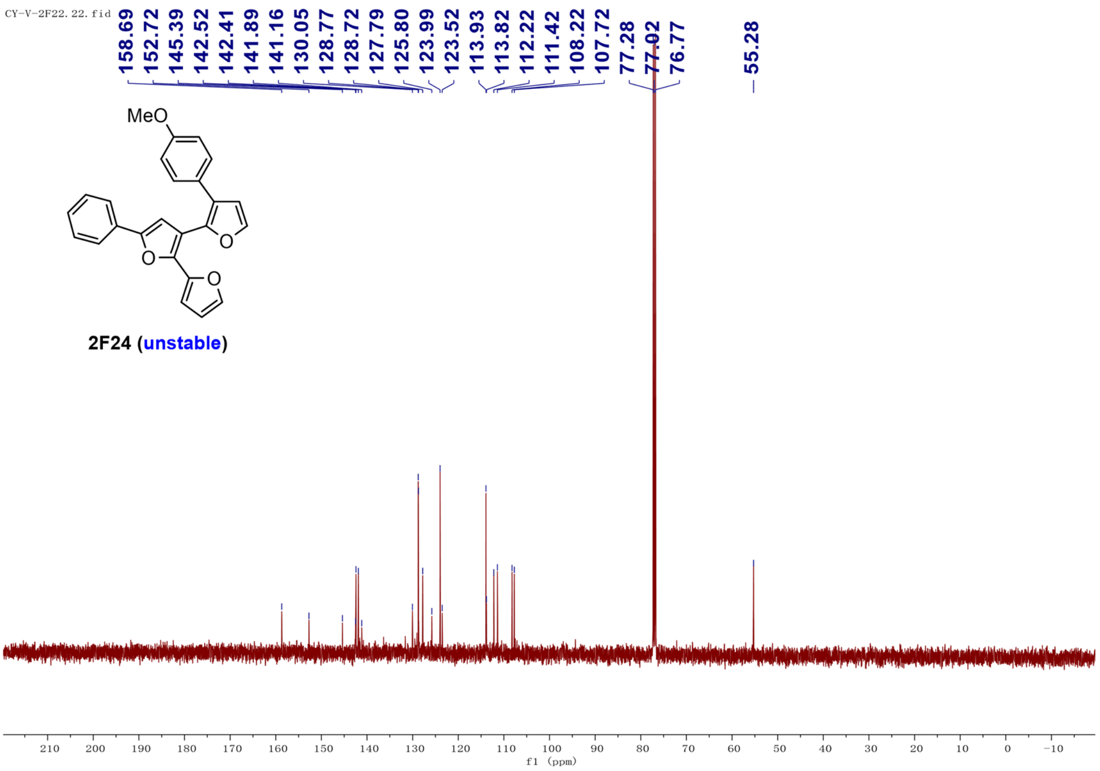

Supplementary Figure 173. ^{13}C NMR (126 MHz, $CDCl_3$) spectra for compound **2F24**

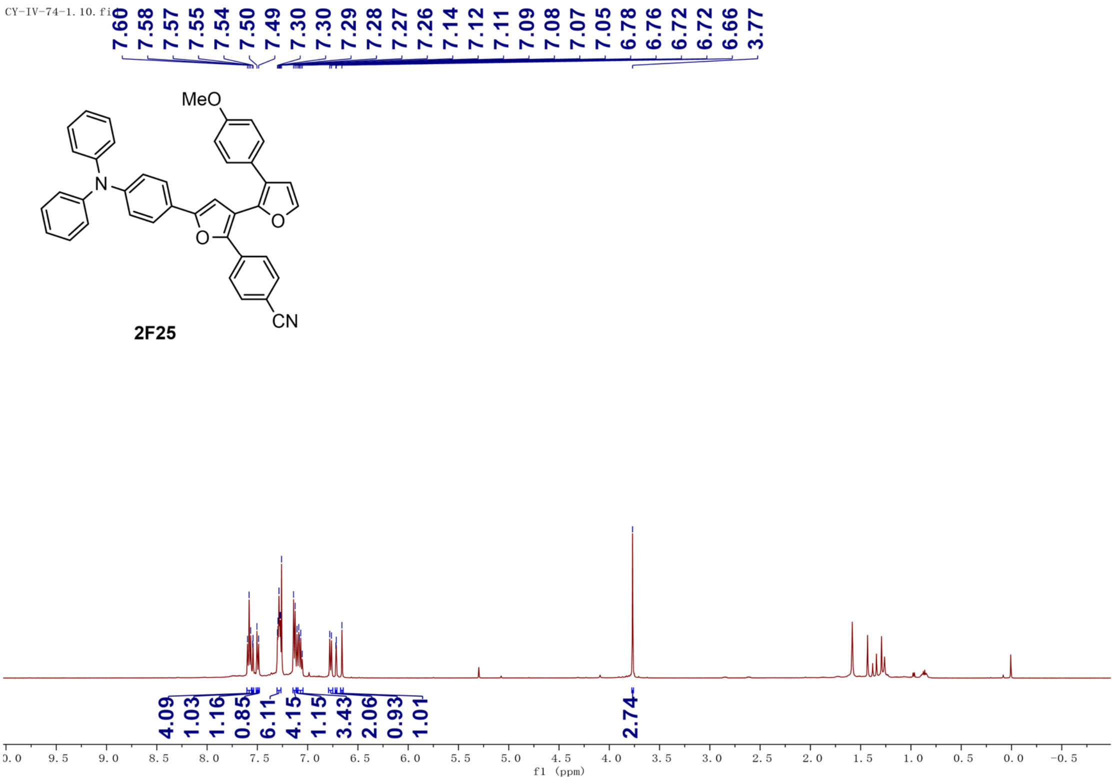

Supplementary Figure 174. ^{1}H NMR (500 MHz, $CDCl_3$) spectra for compound **2F25**

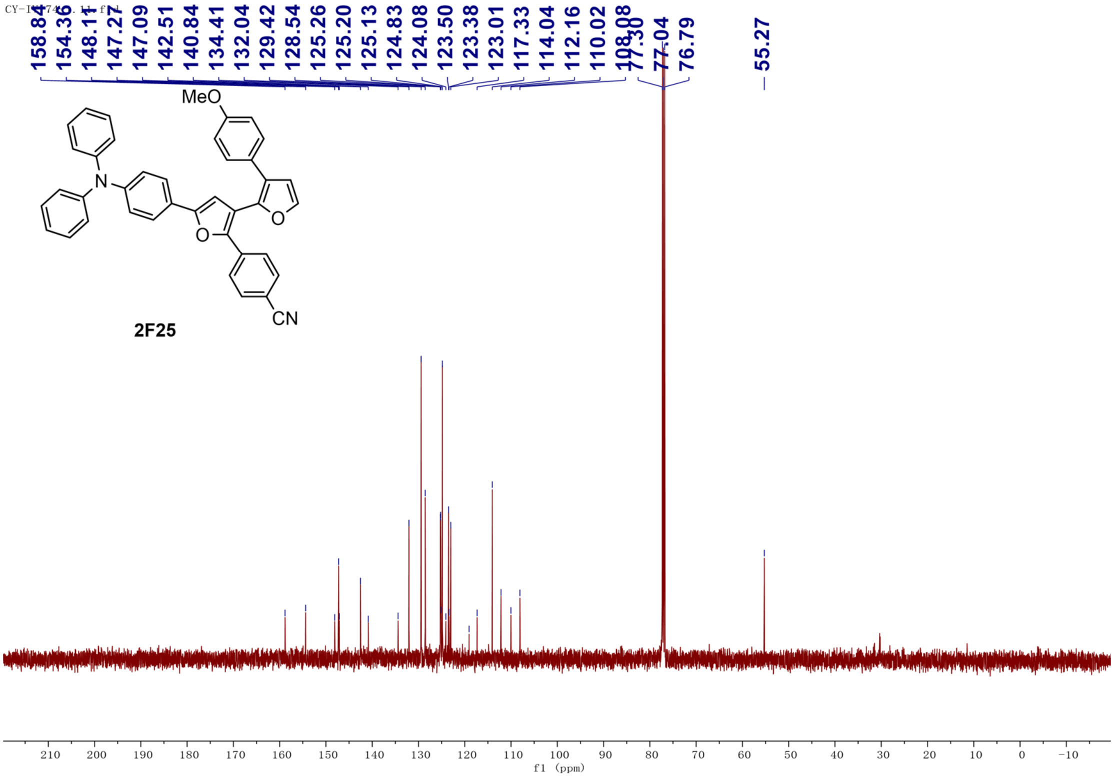

Supplementary Figure 175. ^{13}C NMR (126 MHz, $CDCl_3$) spectra for compound **2F25**

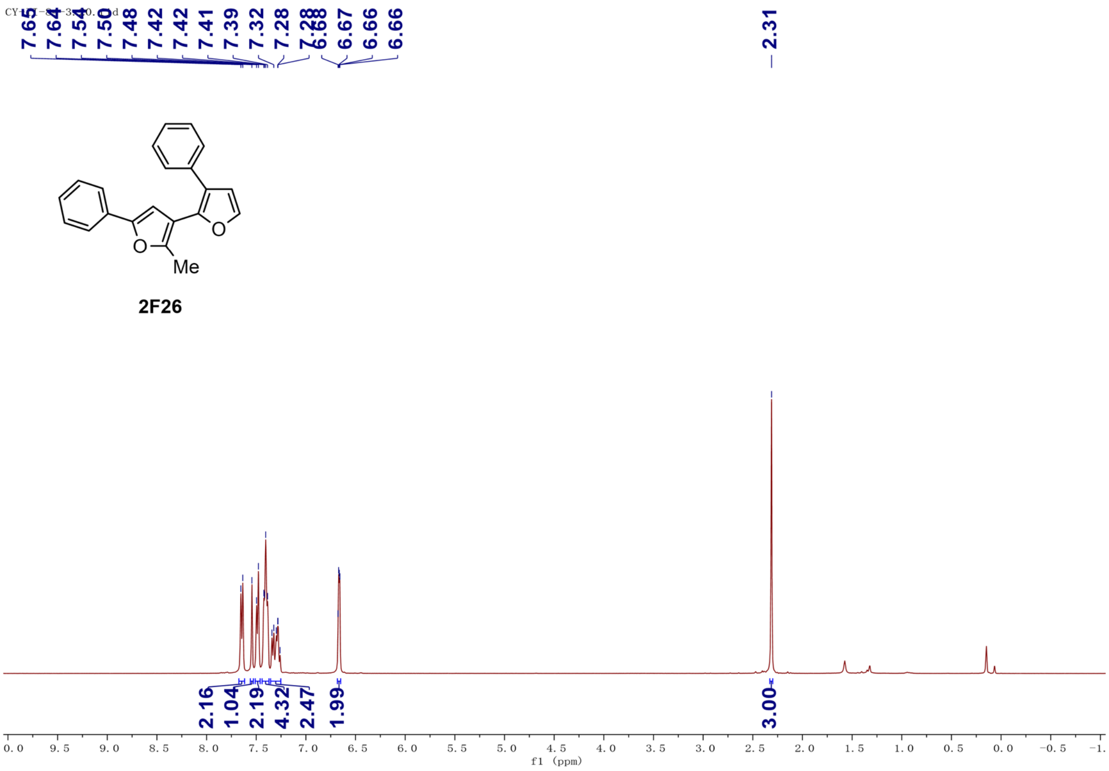

Supplementary Figure 176. ^{1}H NMR (400 MHz, $CDCl_3$) spectra for compound **2F26**

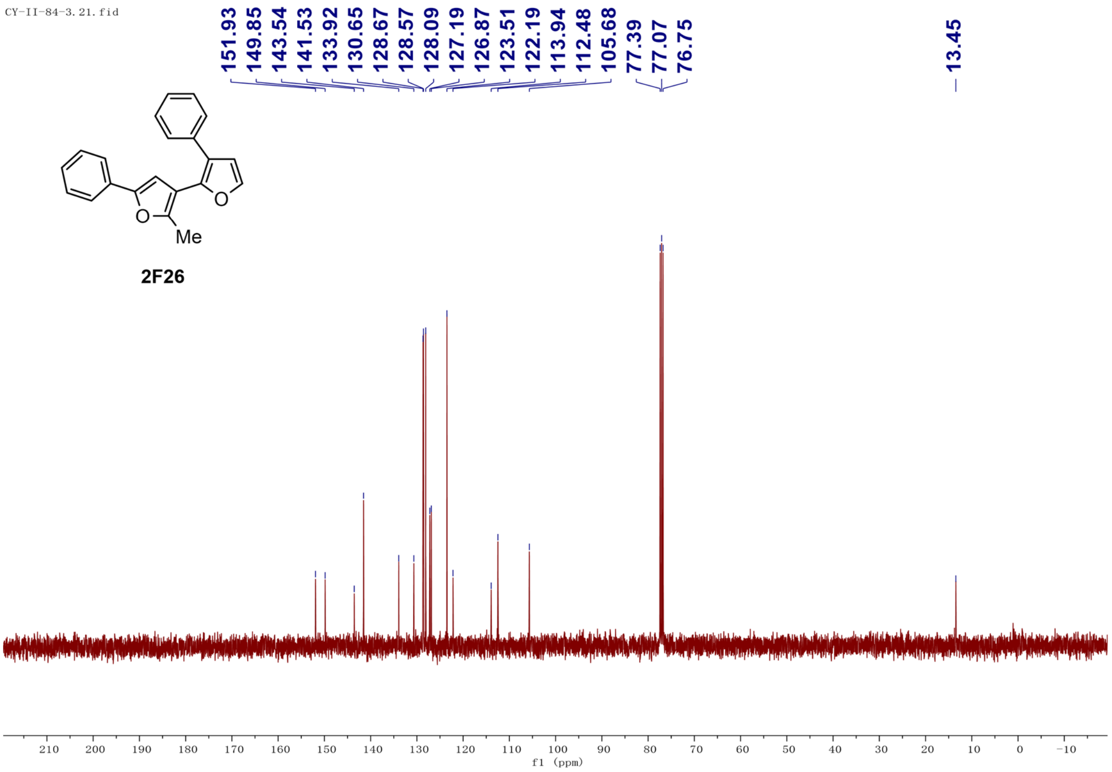

Supplementary Figure 177. ^{13}C NMR (101 MHz, $CDCl_3$) spectra for compound **2F26**

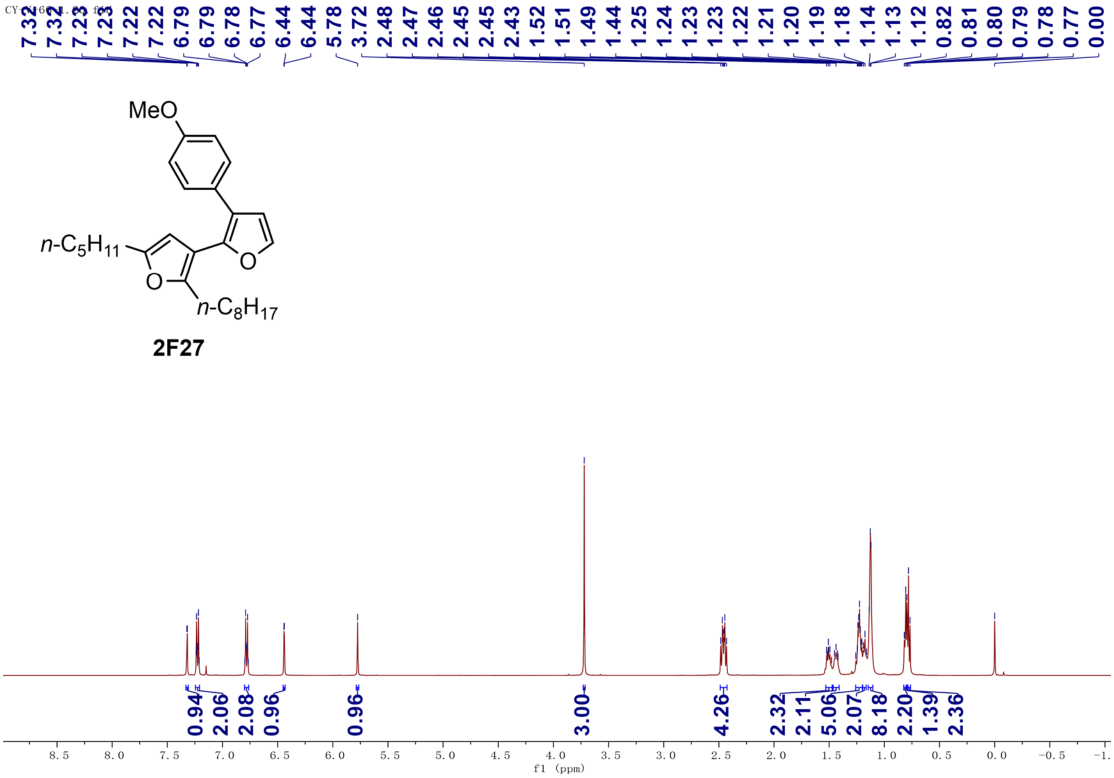

Supplementary Figure 178. ^{1}H NMR (500 MHz, $CDCl_3$) spectra for compound **2F27**

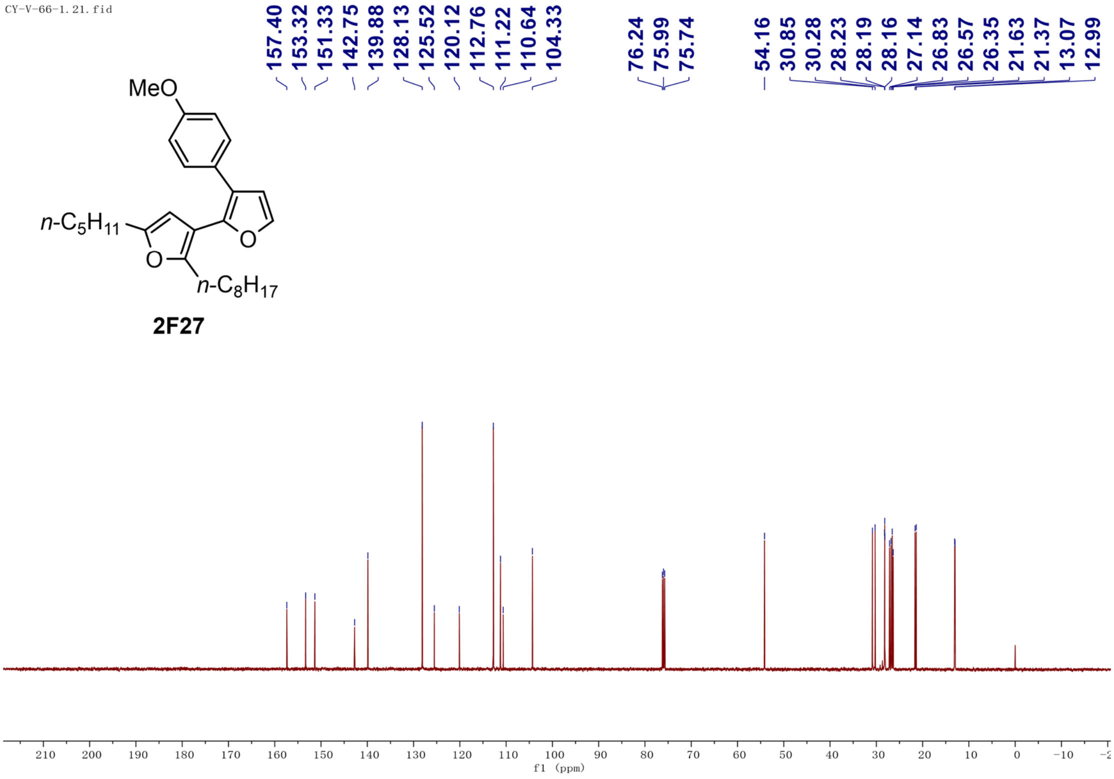

Supplementary Figure 179. ^{13}C NMR (126 MHz, $CDCl_3$) spectra for compound **2F27**

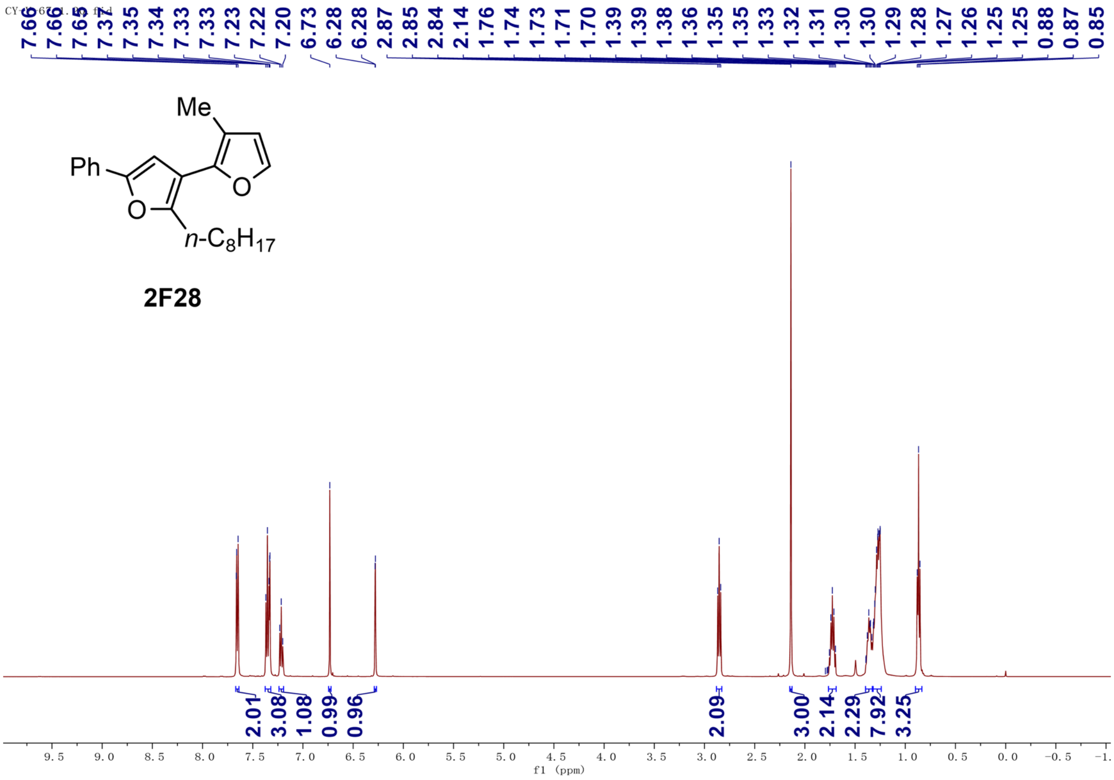

Supplementary Figure 180. ^{1}H NMR (500 MHz, $CDCl_3$) spectra for compound **2F28**

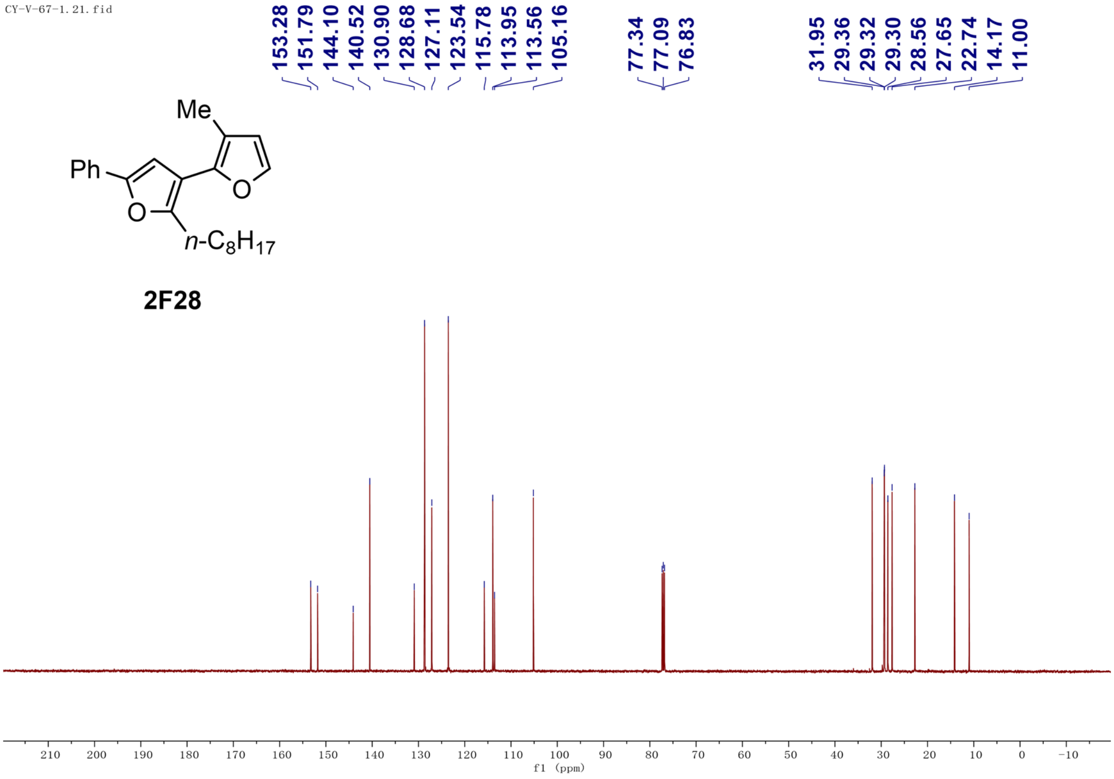

Supplementary Figure 181. ^{13}C NMR (126 MHz, $CDCl_3$) spectra for compound **2F28**

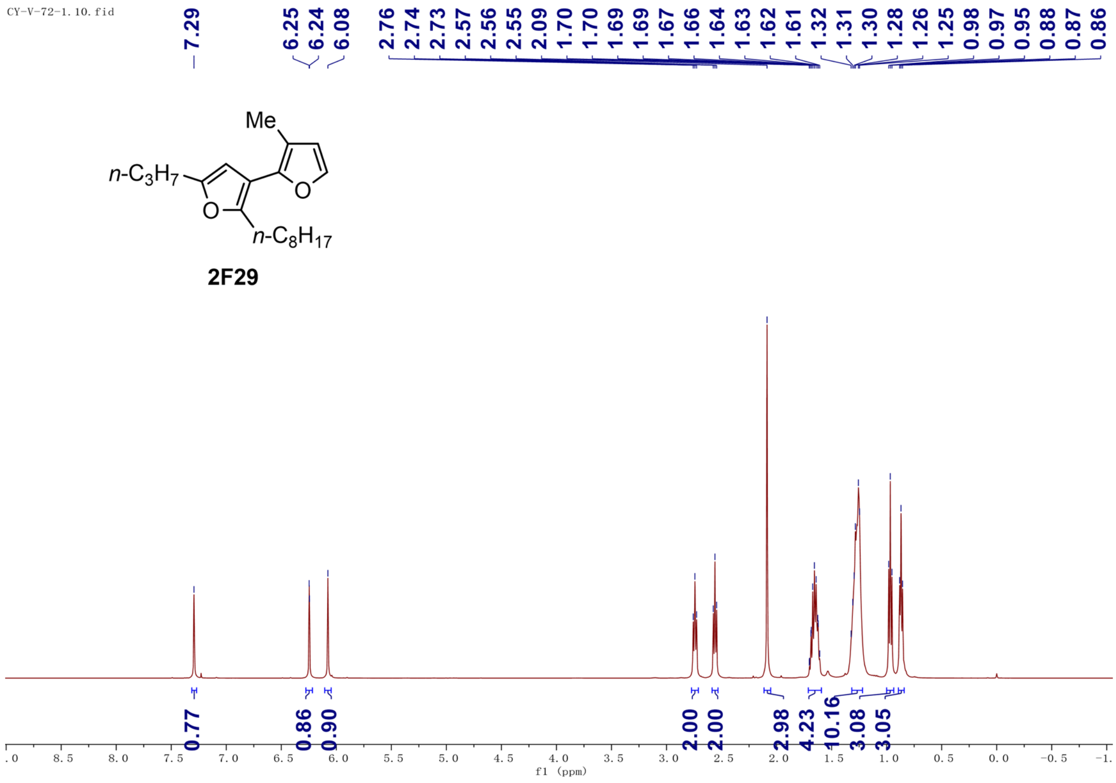

Supplementary Figure 182. ^{1}H NMR (500 MHz, $CDCl_3$) spectra for compound **2F29**

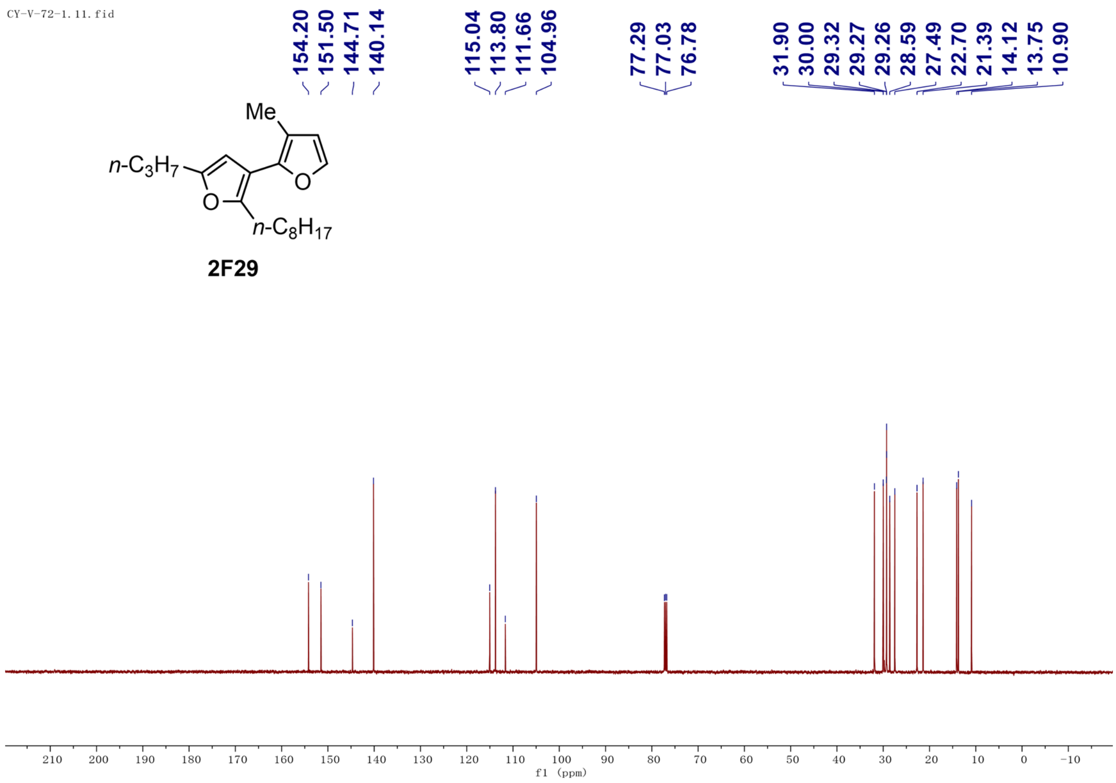

Supplementary Figure 183. ^{13}C NMR (126 MHz, $CDCl_3$) spectra for compound **2F29**

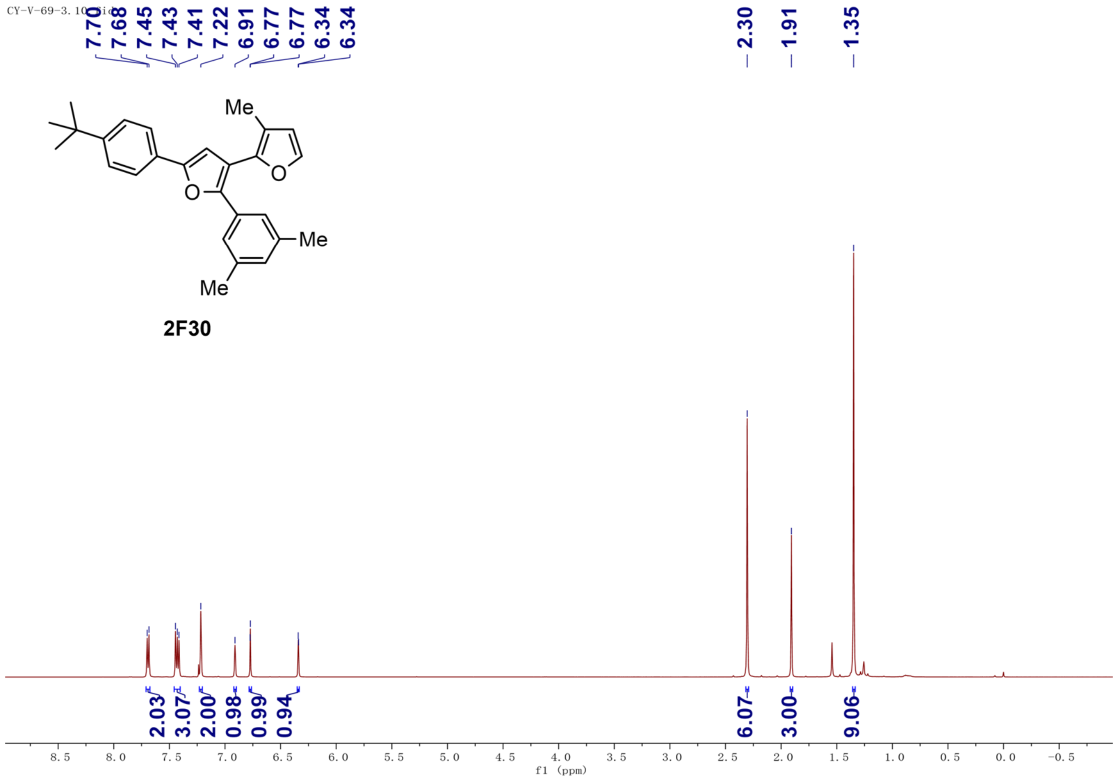

Supplementary Figure 184. ^{1}H NMR (500 MHz, $CDCl_3$) spectra for compound **2F30**

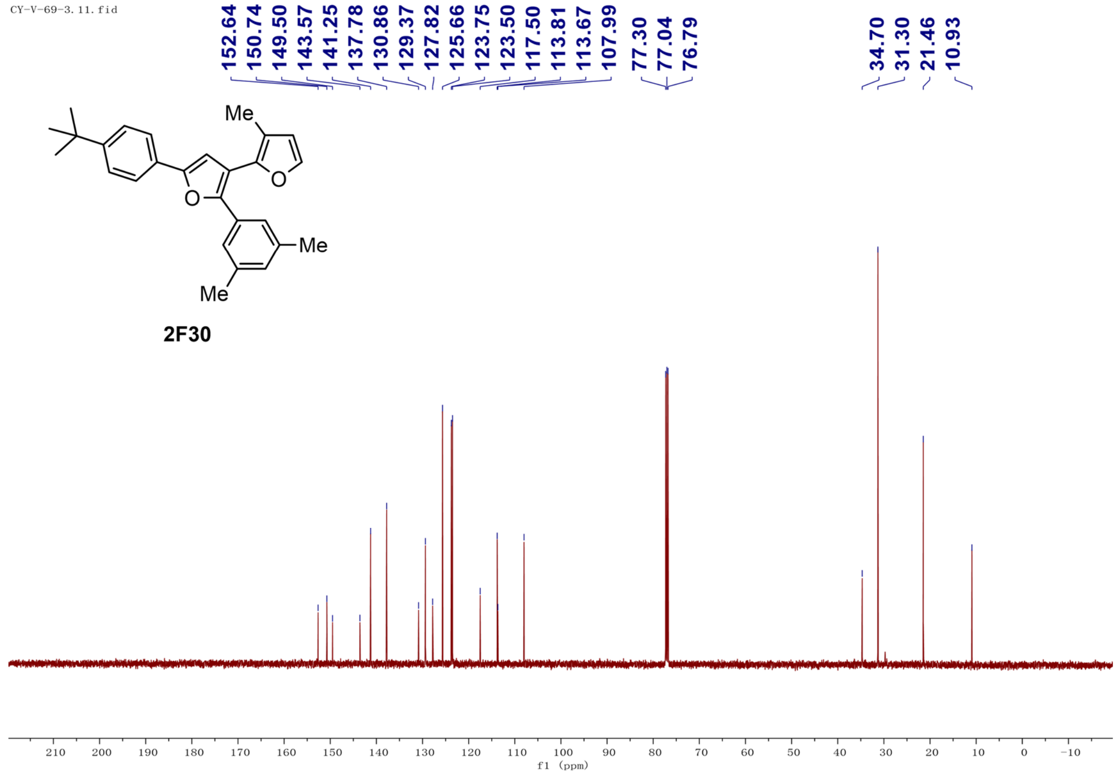

Supplementary Figure 185. ^{13}C NMR (126 MHz, $CDCl_3$) spectra for compound **2F30**

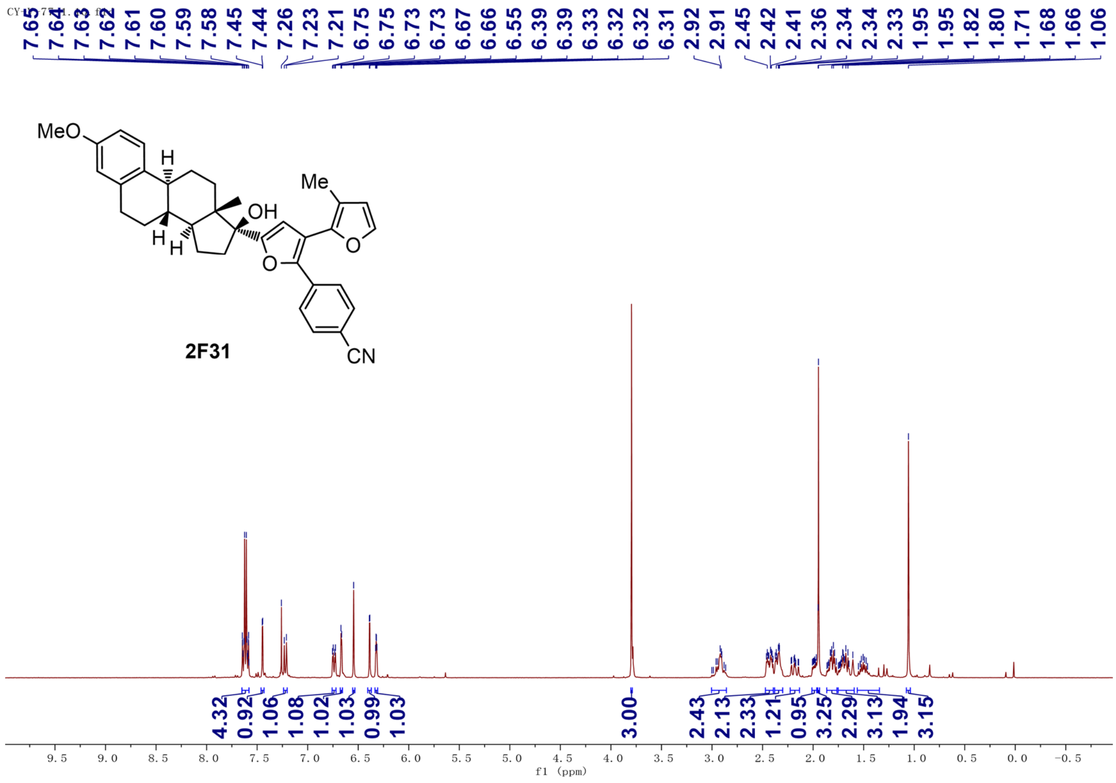

Supplementary Figure 186. ^{1}H NMR (400 MHz, $CDCl_3$) spectra for compound **2F31**

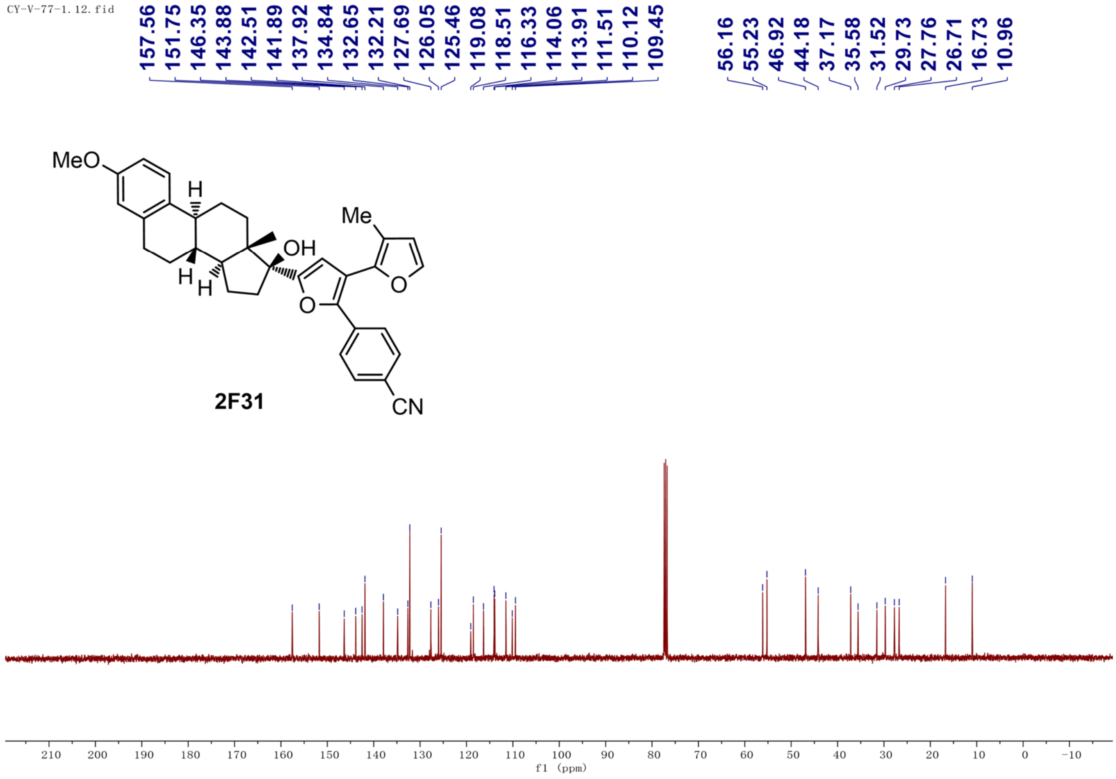

Supplementary Figure 187. ^{13}C NMR (101 MHz, $CDCl_3$) spectra for compound **2F31**

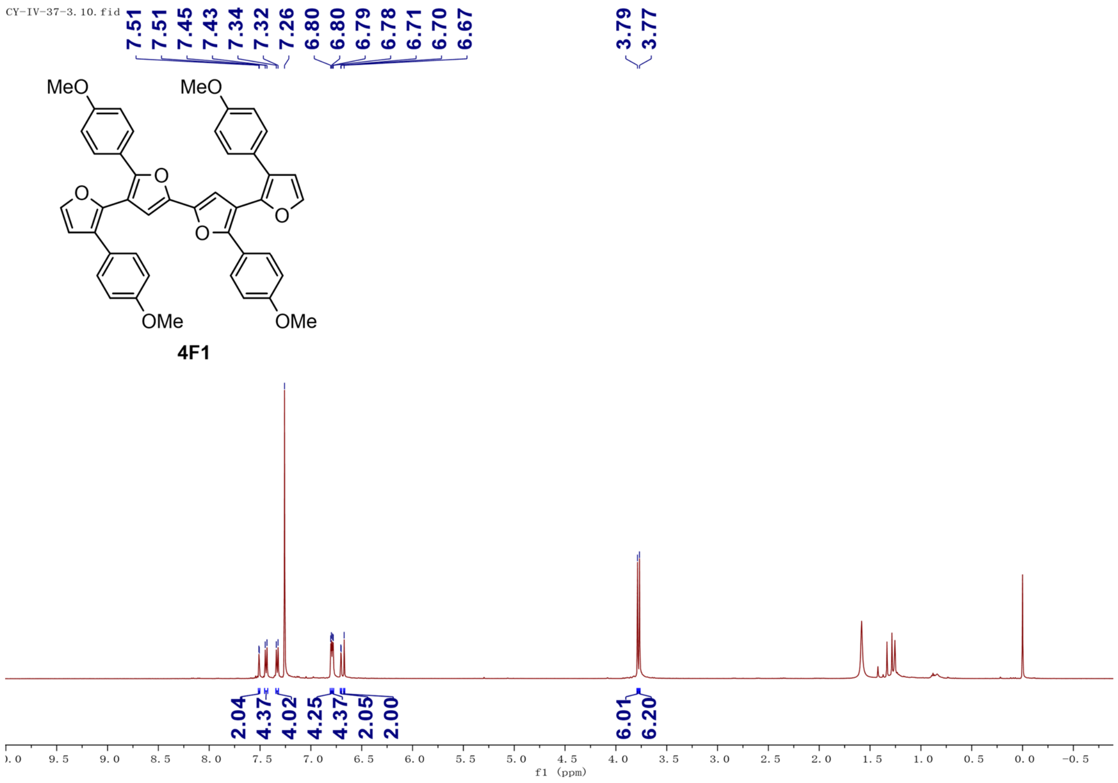

Supplementary Figure 188. ^{1}H NMR (500 MHz, $CDCl_3$) spectra for compound **4F1**

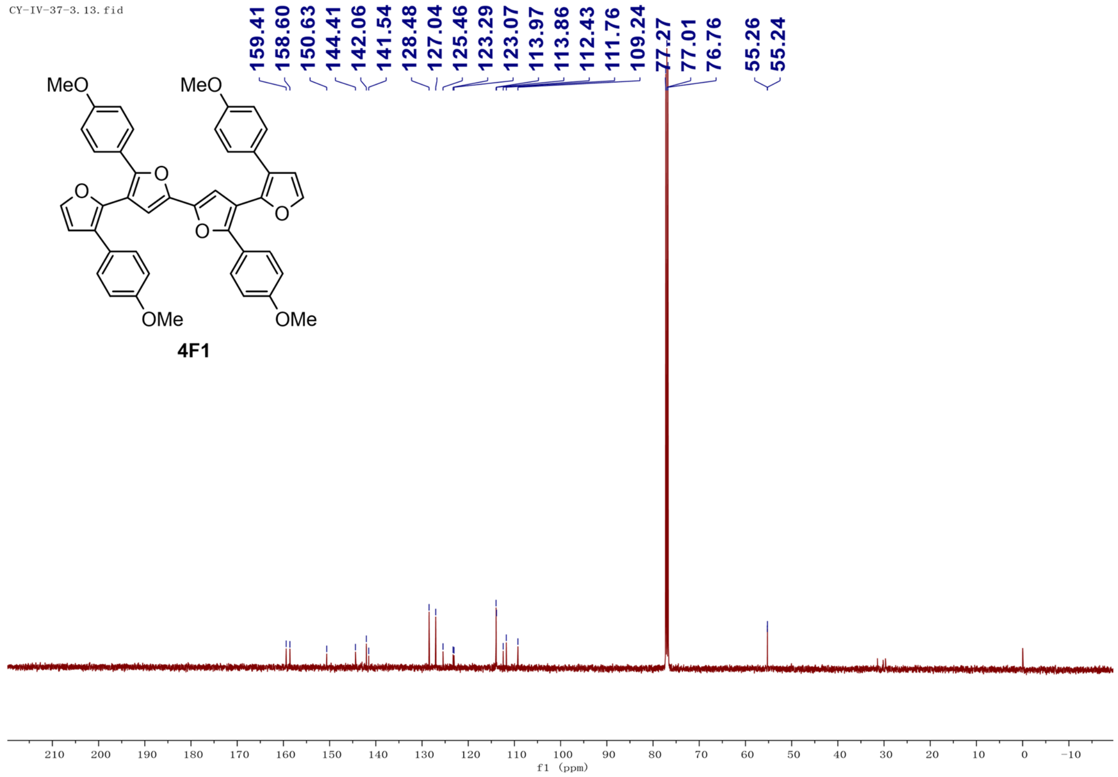

Supplementary Figure 189. ^{13}C NMR (126 MHz, $CDCl_3$) spectra for compound **4F1**

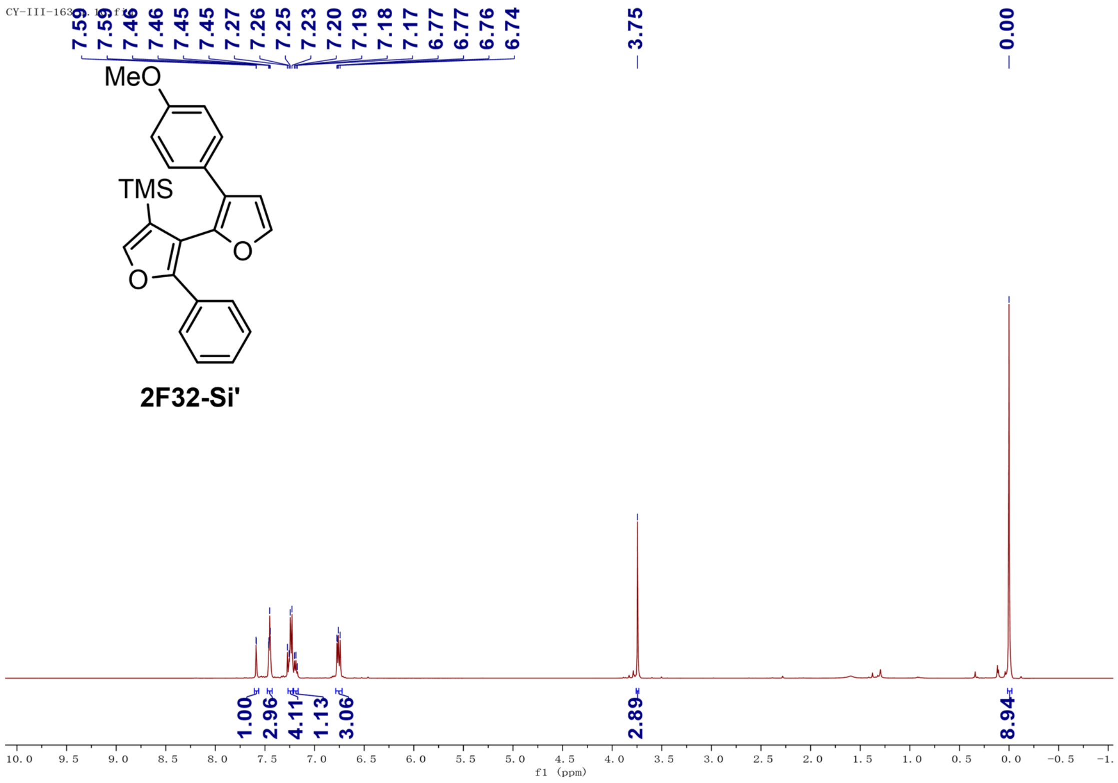

Supplementary Figure 190. ^{1}H NMR (500 MHz, $CDCl_3$) spectra for compound **2F32-Si'**

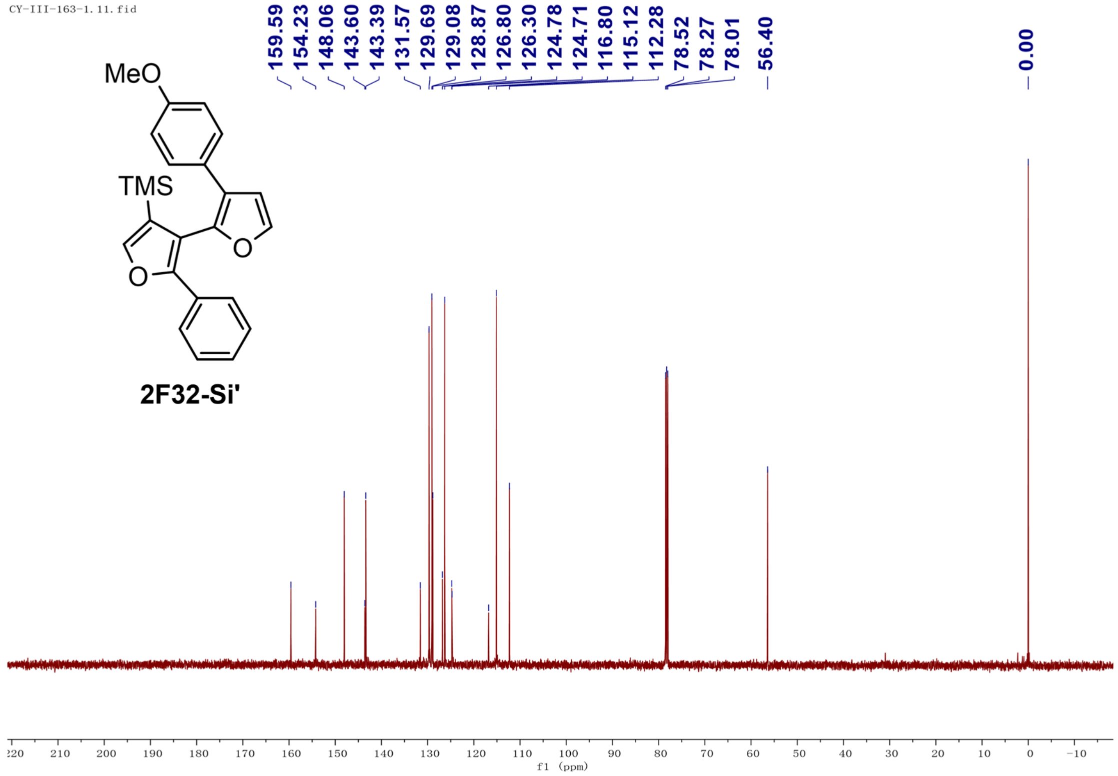

Supplementary Figure 191. ^{13}C NMR (126 MHz, $CDCl_3$) spectra for compound **2F32-Si'**

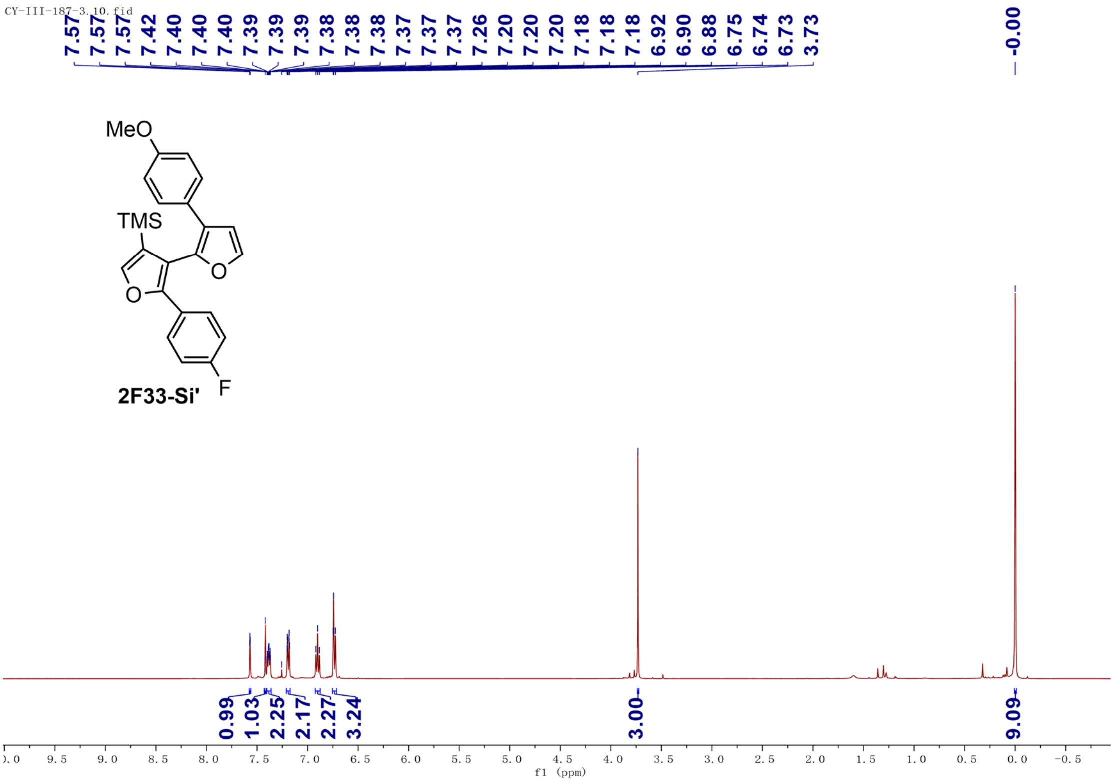

Supplementary Figure 192. ^{1}H NMR (500 MHz, $CDCl_3$) spectra for compound **2F33-Si'**

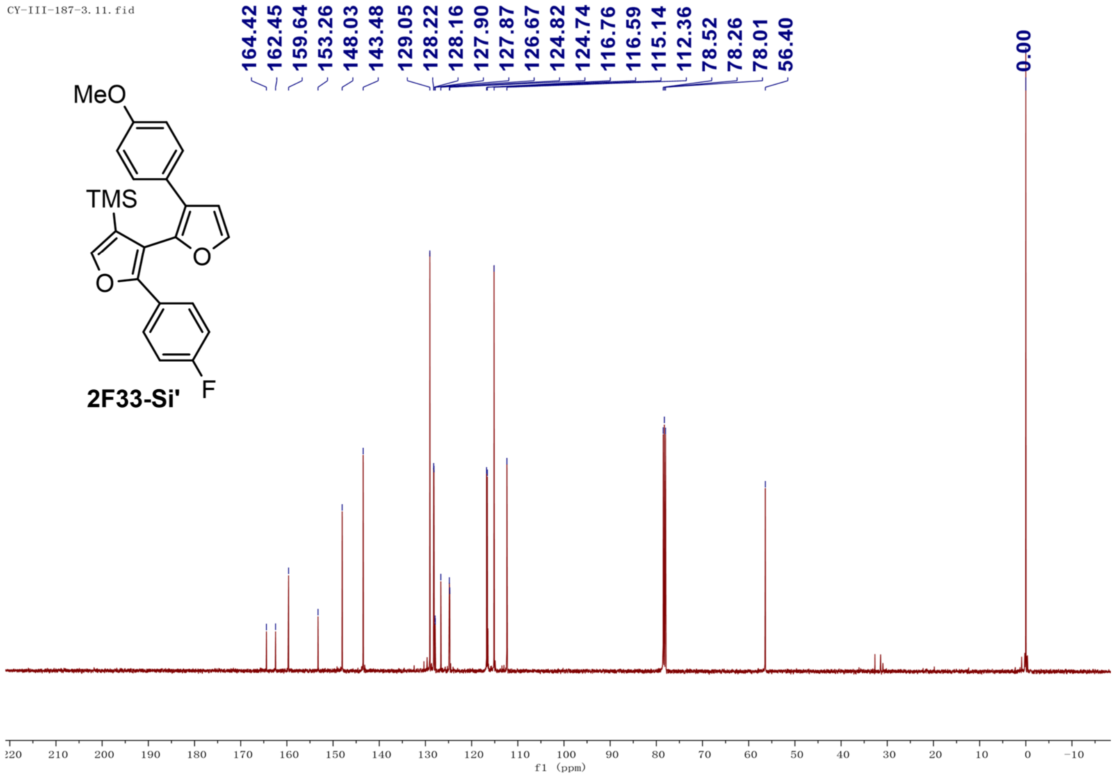

Supplementary Figure 193. ^{13}C NMR (126 MHz, $CDCl_3$) spectra for compound **2F33-Si'**

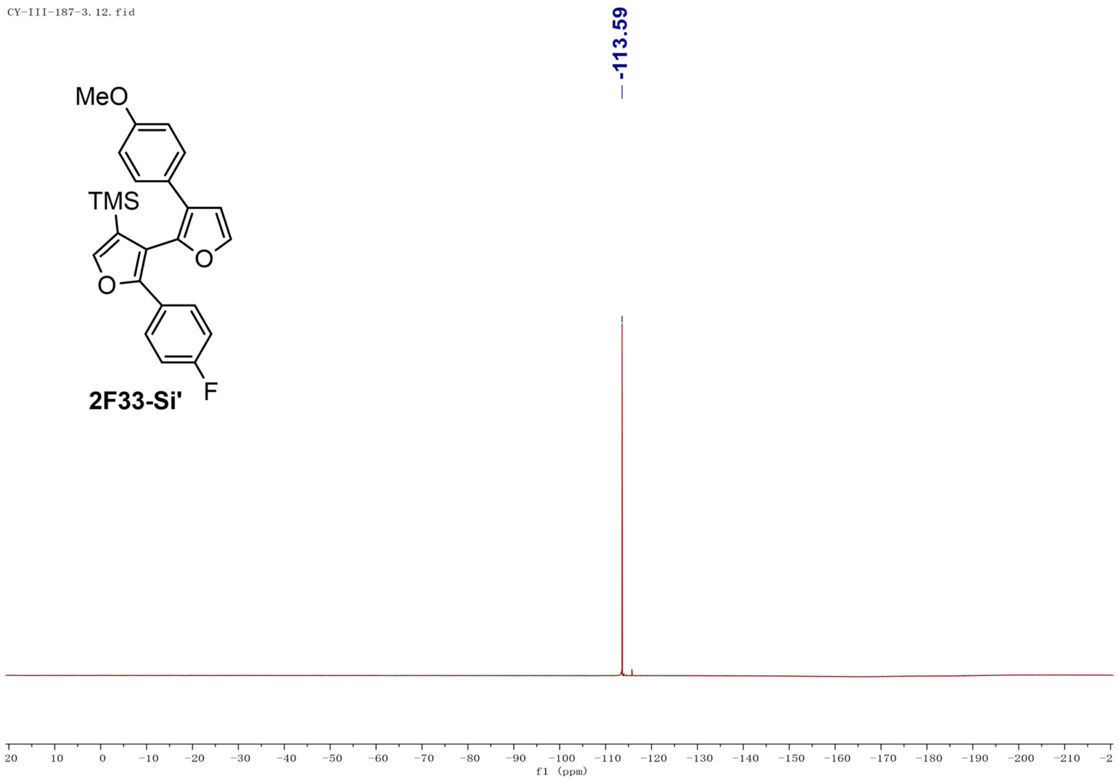

Supplementary Figure 194. ^{19}F NMR (471 MHz, $CDCl_3$) spectra for compound **2F33-Si'**

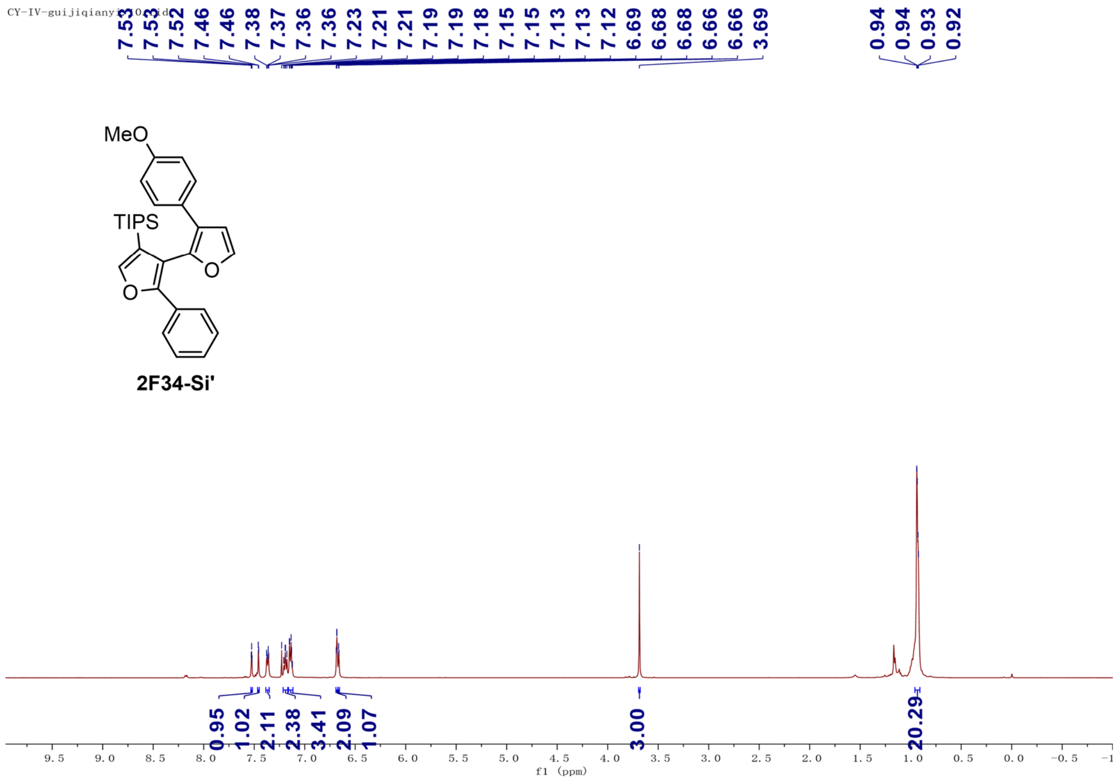

Supplementary Figure 195. ^{1}H NMR (500 MHz, $CDCl_3$) spectra for compound **2F34-Si'**

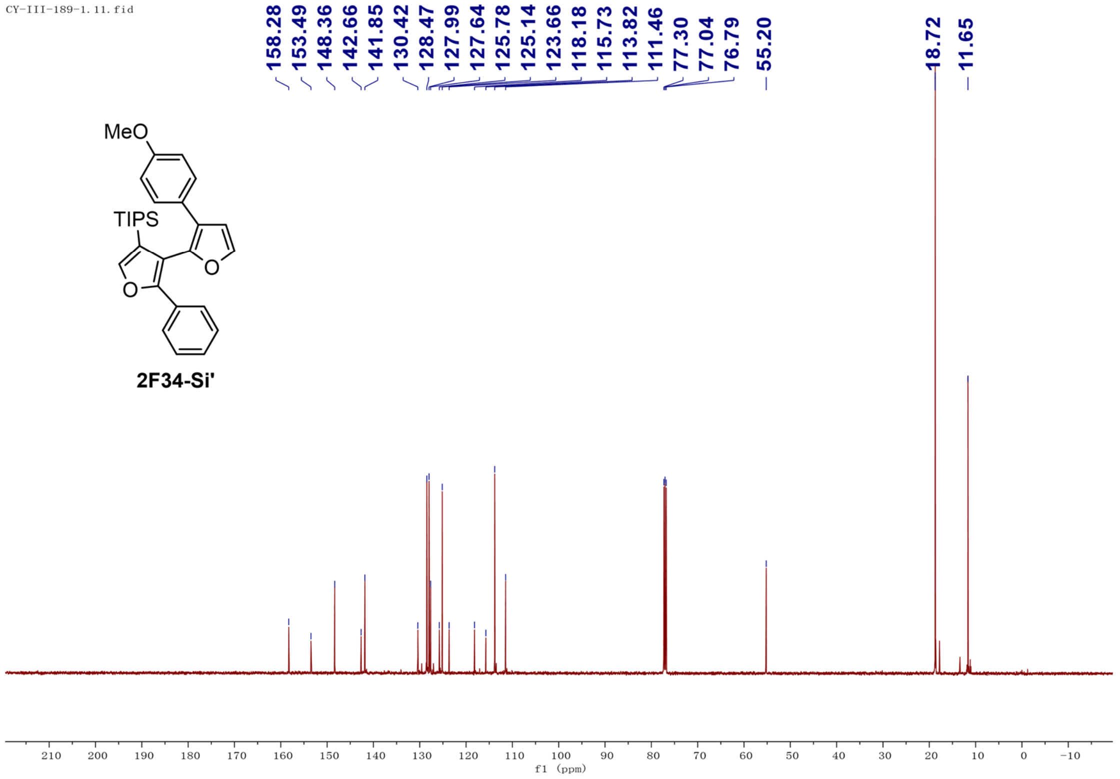

Supplementary Figure 196. ^{13}C NMR (126 MHz, $CDCl_3$) spectra for compound **2F34-Si'**

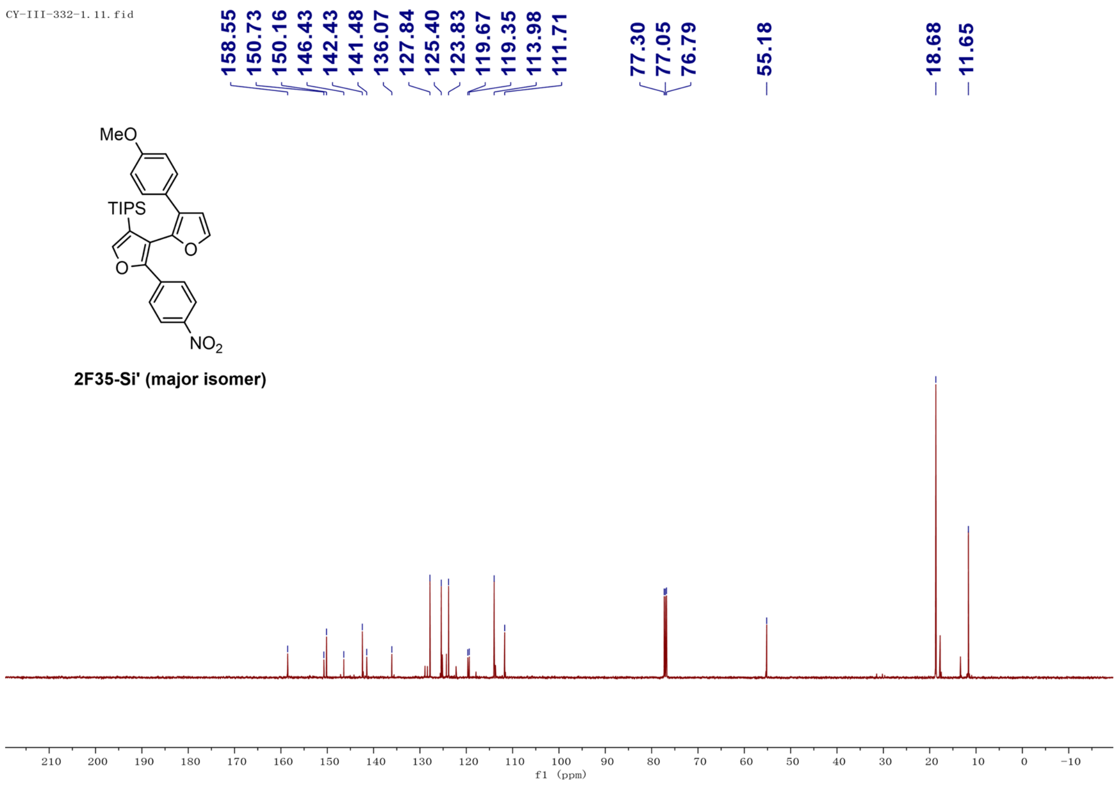

Supplementary Figure 197. ^{1}H NMR (500 MHz, $CDCl_3$) spectra for compound **2F35-Si'**

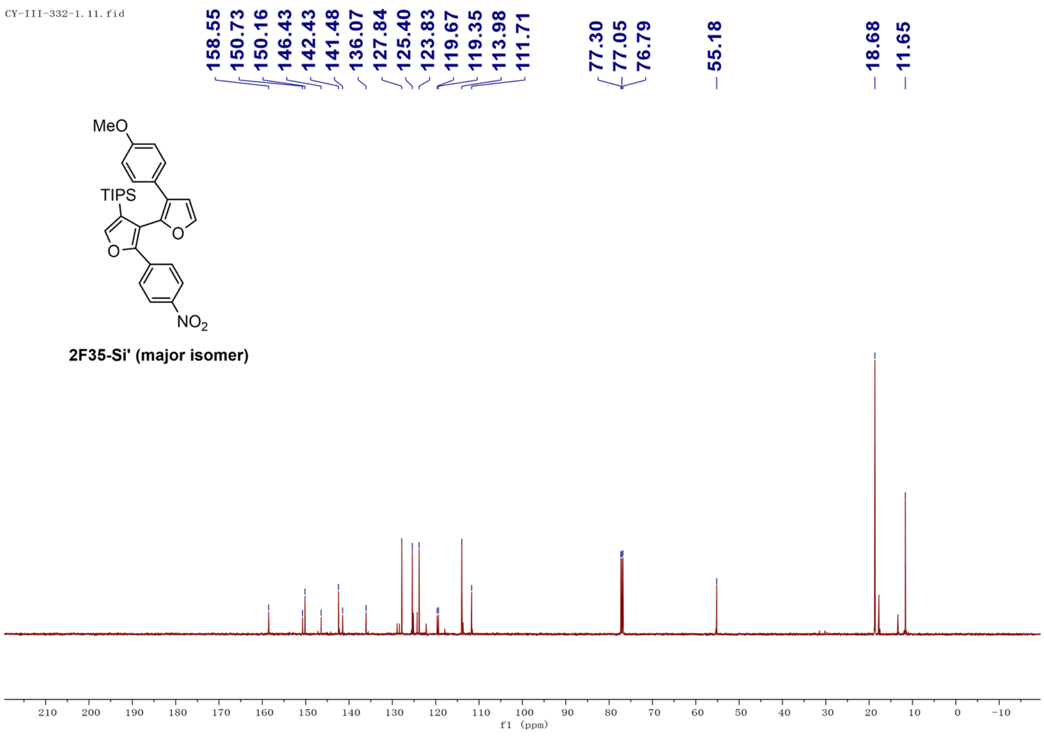

Supplementary Figure 198. ^{13}C NMR (126 MHz, $CDCl_3$) spectra for compound **2F35-Si'**

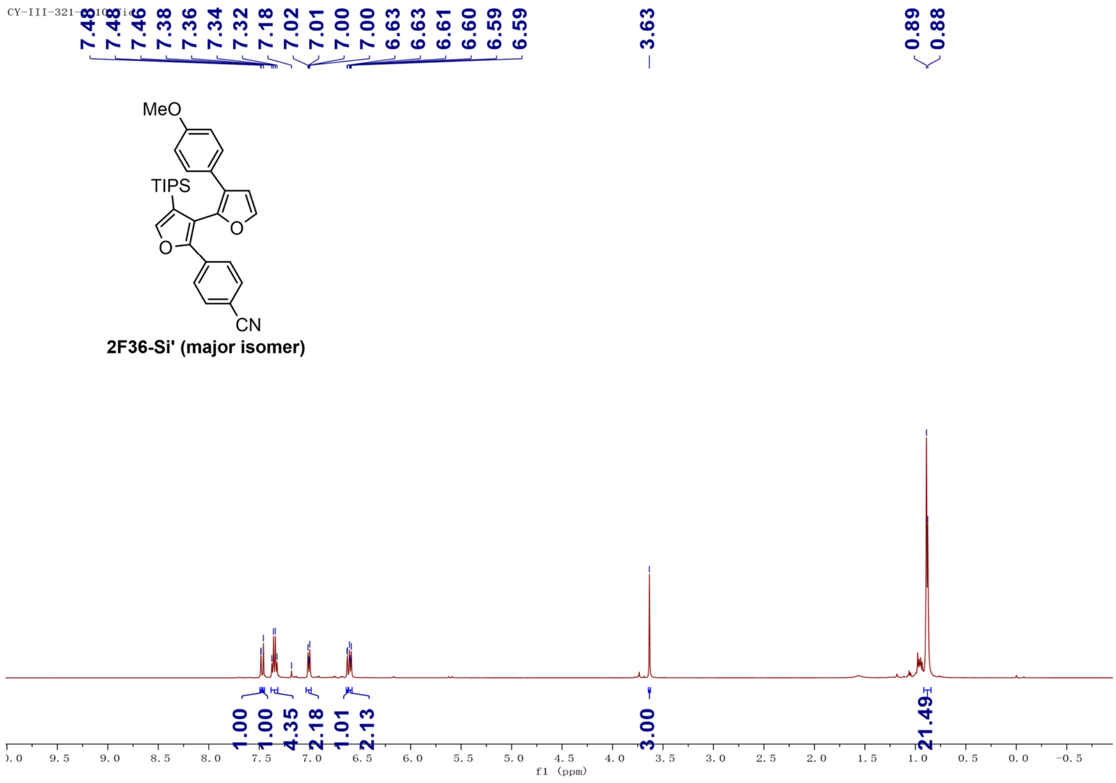

Supplementary Figure 199. ^{1}H NMR (500 MHz, $CDCl_3$) spectra for compound **2F36-Si'**

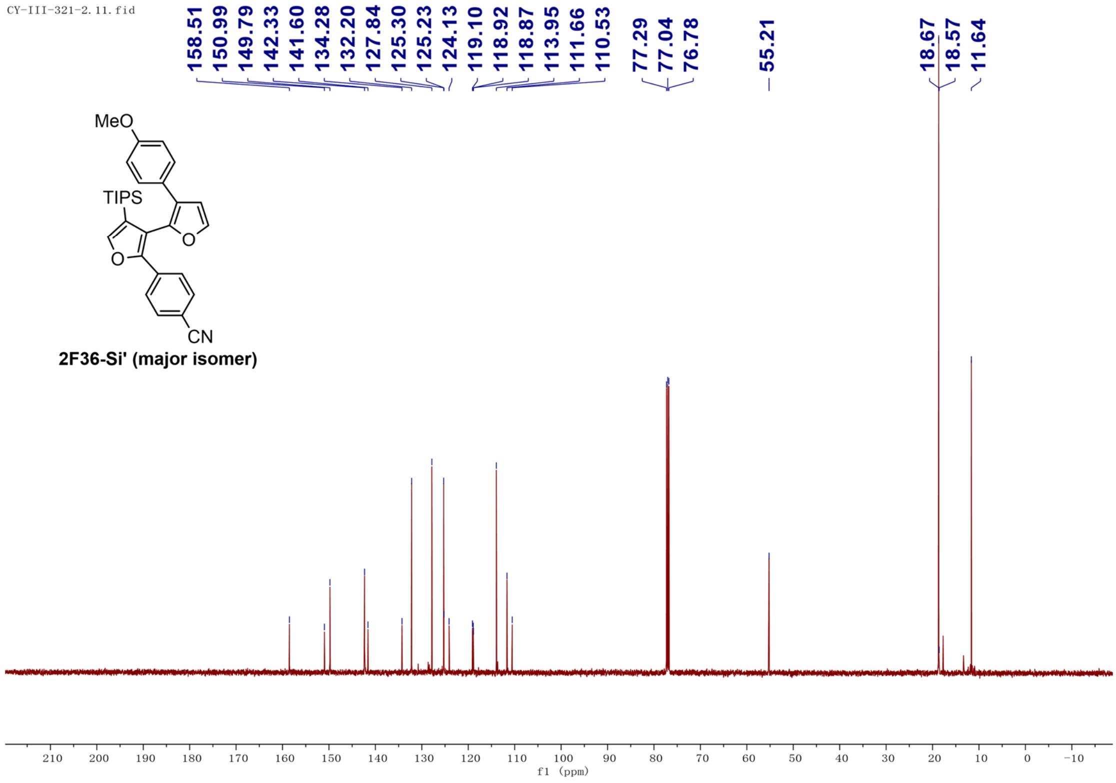

Supplementary Figure 200. ^{13}C NMR (126 MHz, $CDCl_3$) spectra for compound **2F36-Si'**

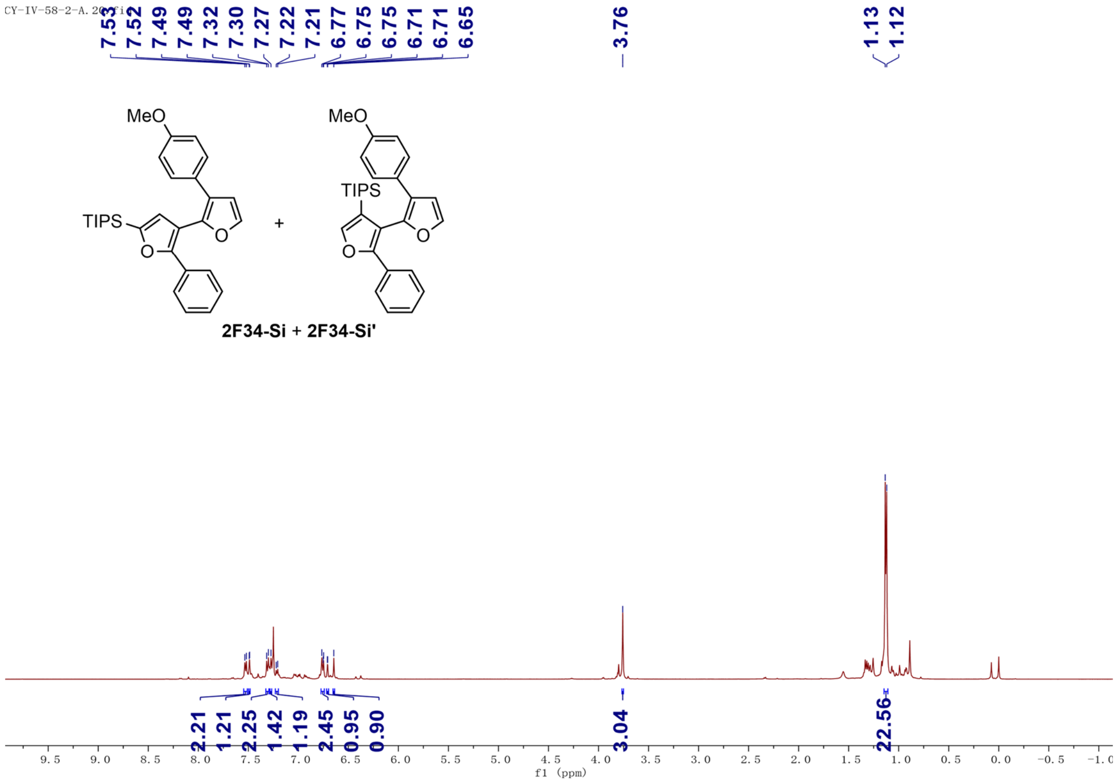

Supplementary Figure 201. ^{1}H NMR (500 MHz, $CDCl_3$) spectra for compound **2F34-Si**

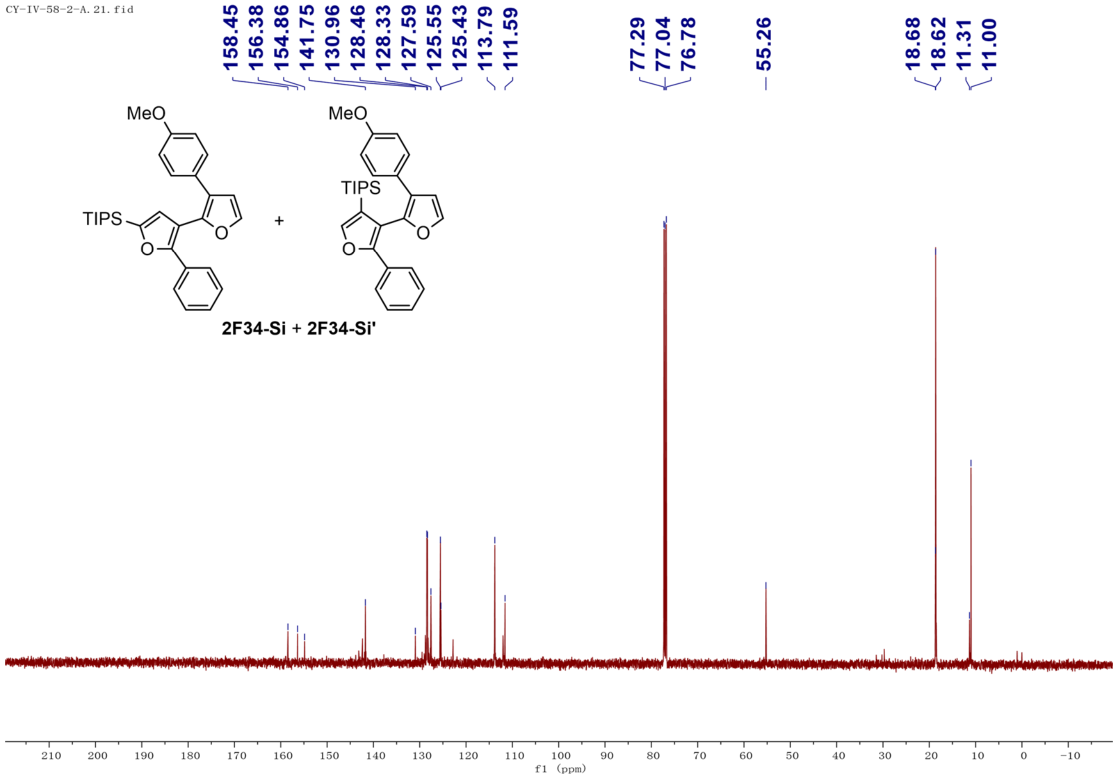

Supplementary Figure 202. ^{13}C NMR (126 MHz, $CDCl_3$) spectra for compound **2F34-Si**

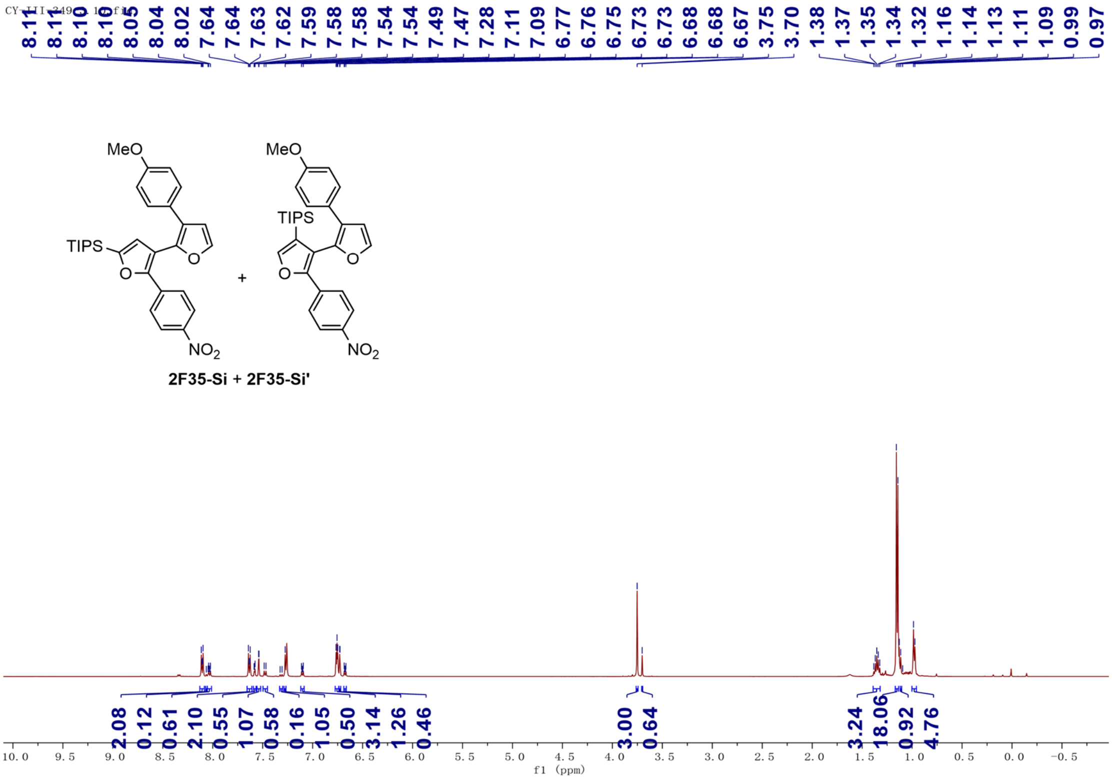

Supplementary Figure 203. ^{1}H NMR (500 MHz, $CDCl_3$) spectra for compound **2F35-Si**

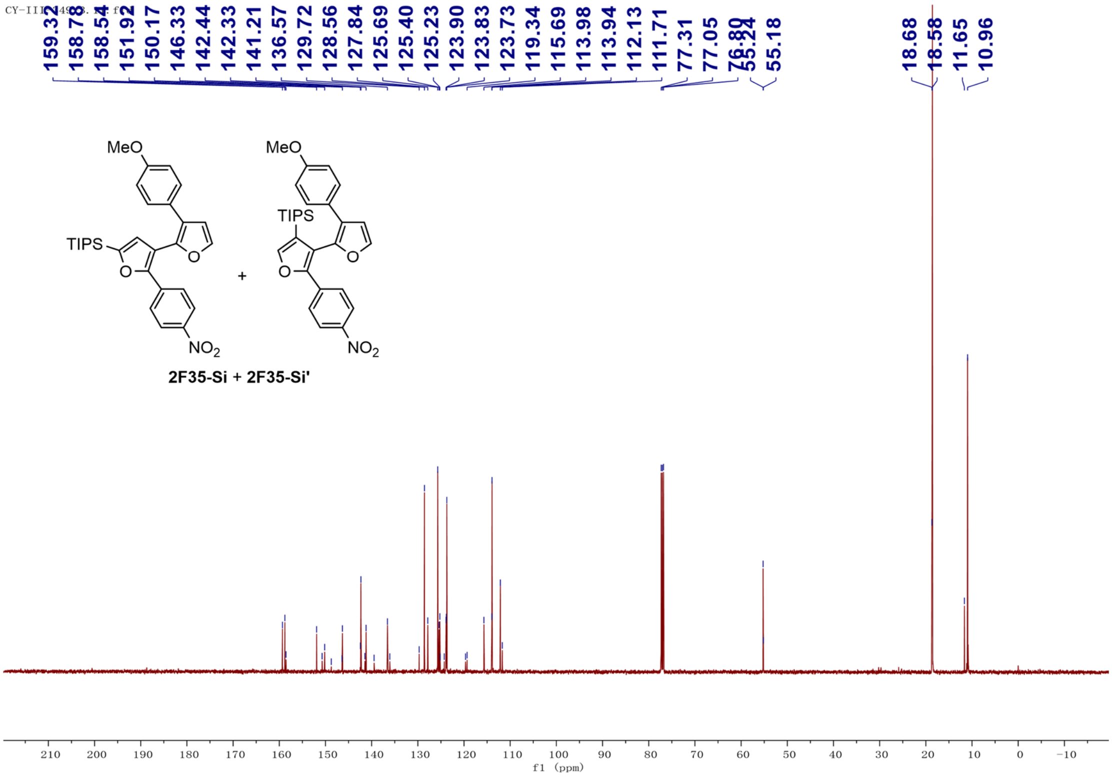

Supplementary Figure 204. ^{13}C NMR (126 MHz, $CDCl_3$) spectra for compound **2F35-Si**

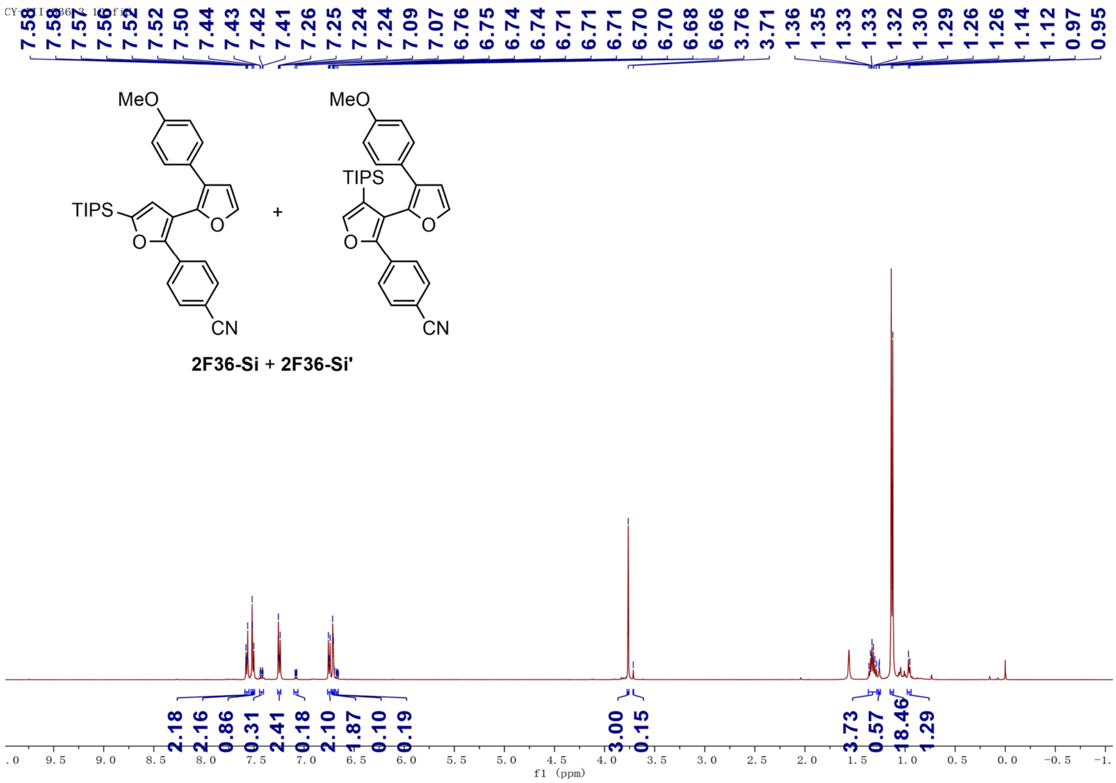

Supplementary Figure 205. ^{1}H NMR (500 MHz, $CDCl_3$) spectra for compound **2F36-Si**

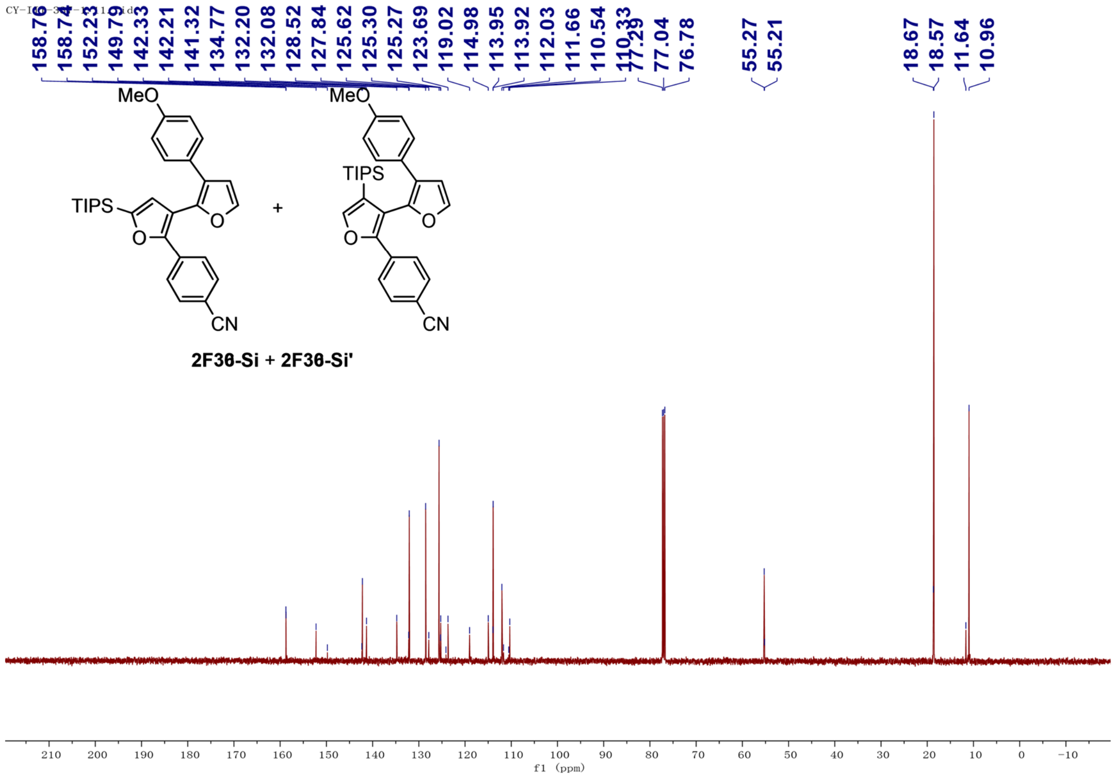

Supplementary Figure 206. ^{13}C NMR (126 MHz, $CDCl_3$) spectra for compound **2F36-Si**

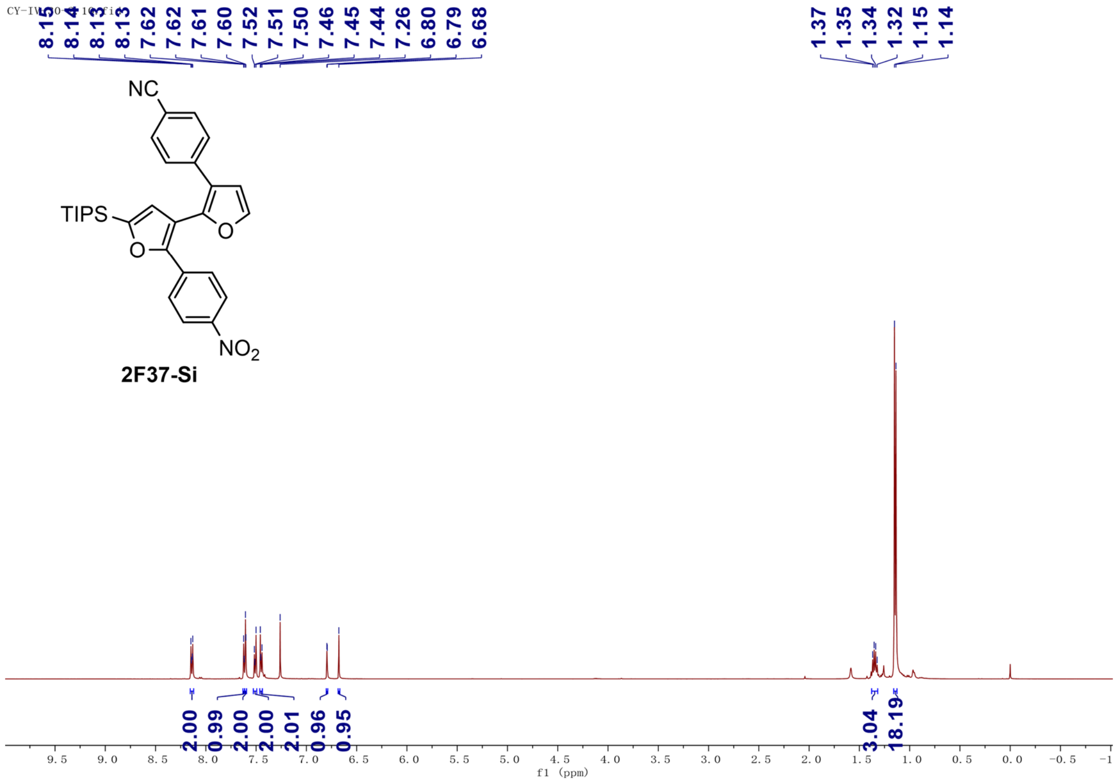

Supplementary Figure 207. ^{1}H NMR (500 MHz, $CDCl_3$) spectra for compound **2F37-Si**

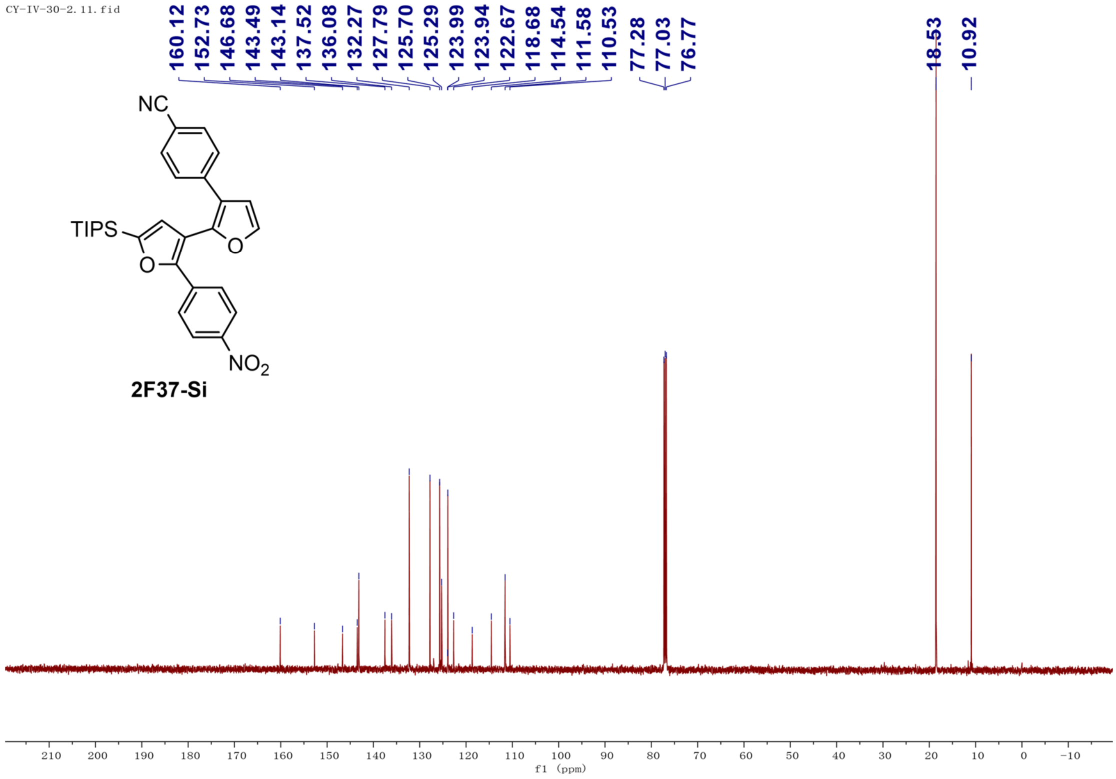

Supplementary Figure 208. ^{13}C NMR (126 MHz, $CDCl_3$) spectra for compound **2F37-Si**

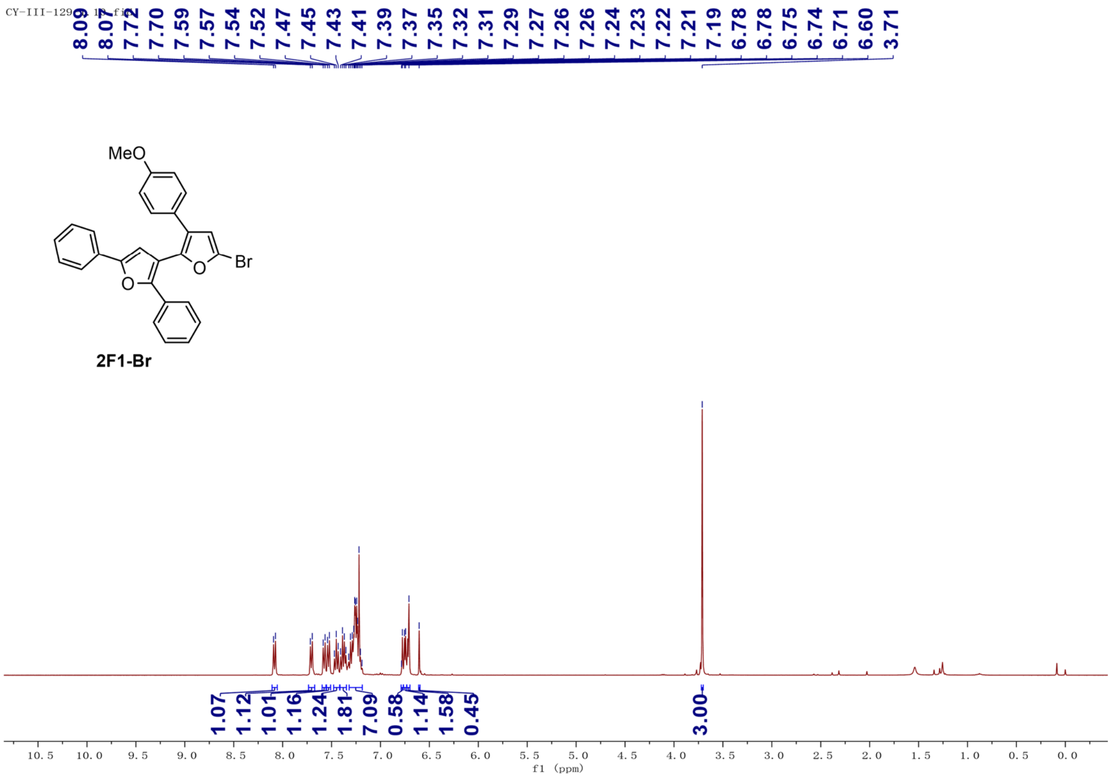

Supplementary Figure 209. ^{1}H NMR (400 MHz, $CDCl_3$) spectra for compound **2F1-Br**

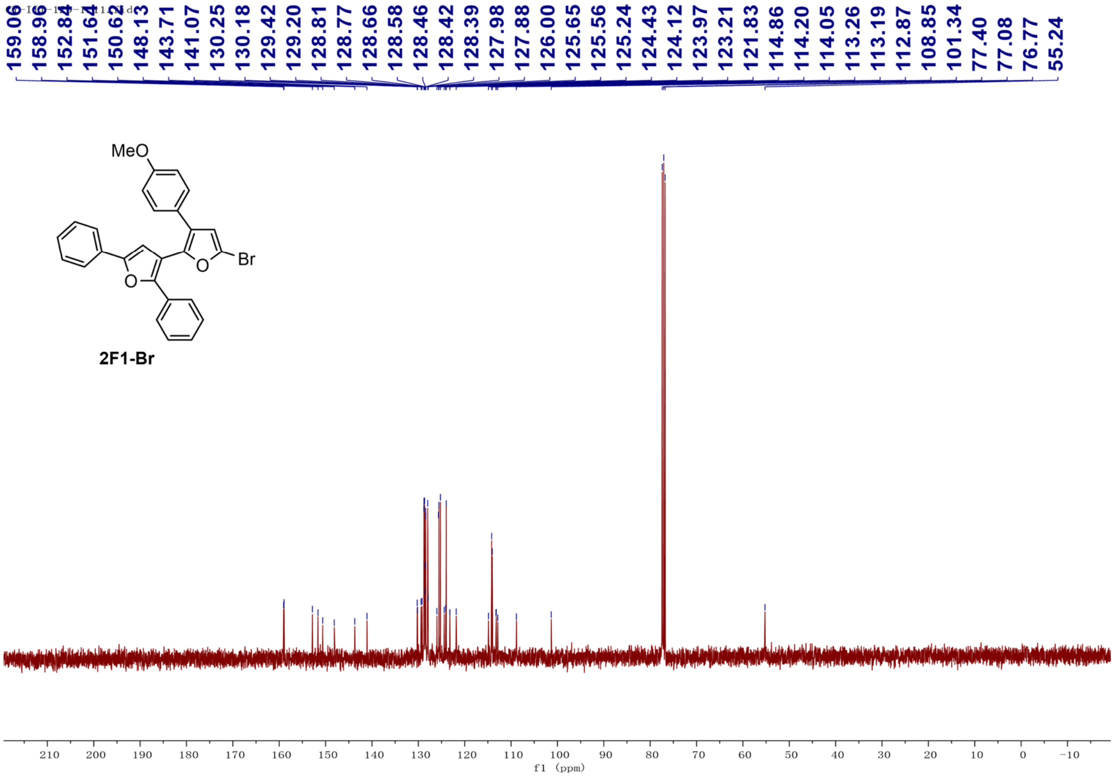

Supplementary Figure 210. ^{13}C NMR (101 MHz, $CDCl_3$) spectra for compound **2F1-Br**

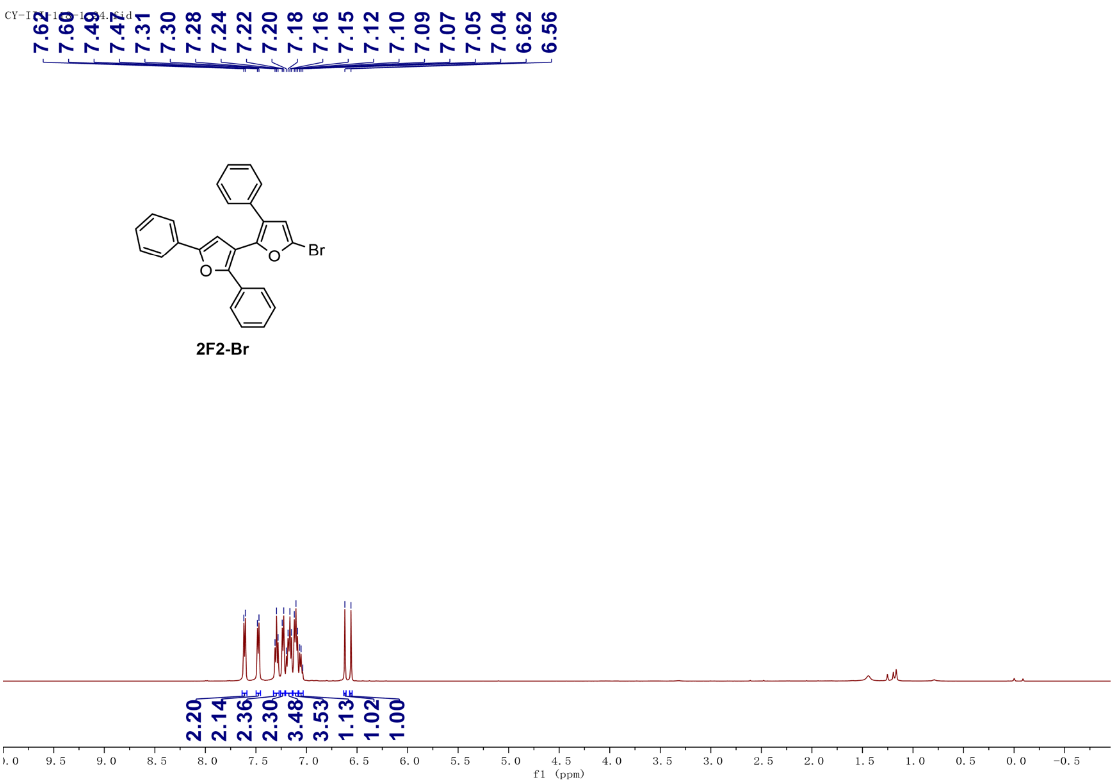

Supplementary Figure 211. ^{1}H NMR (500 MHz, $CDCl_3$) spectra for compound **2F2-Br**

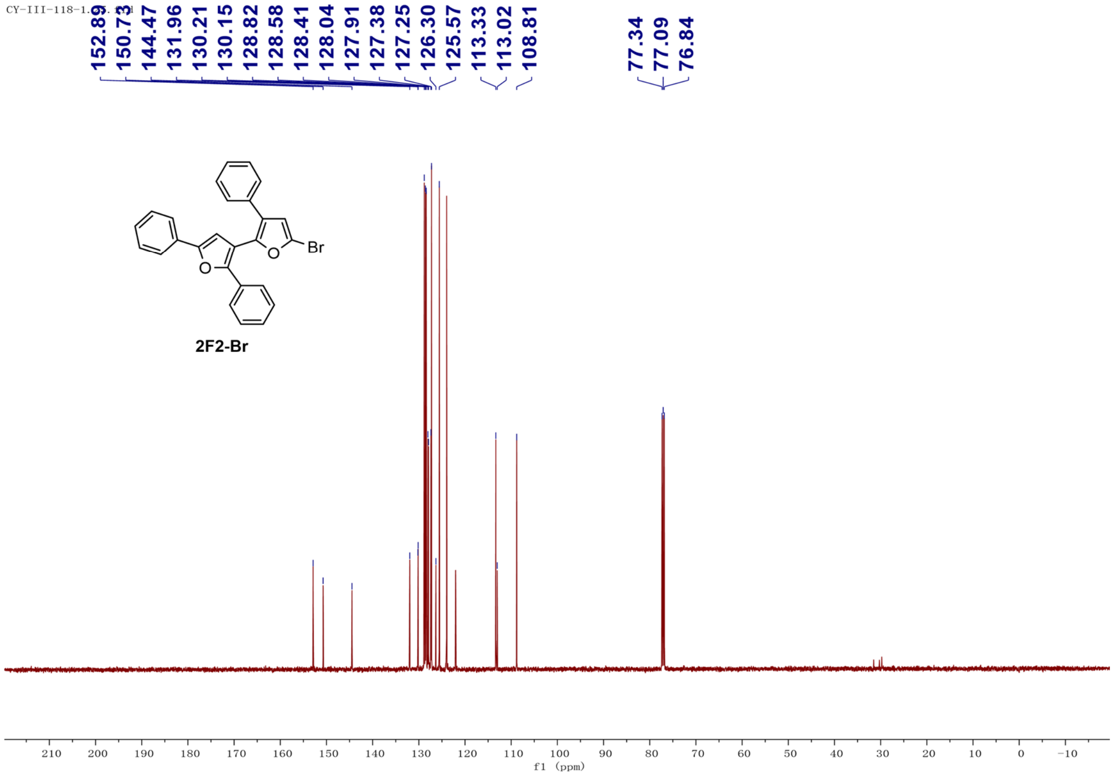

Supplementary Figure 212. ^{13}C NMR (126 MHz, $CDCl_3$) spectra for compound **2F2-Br**

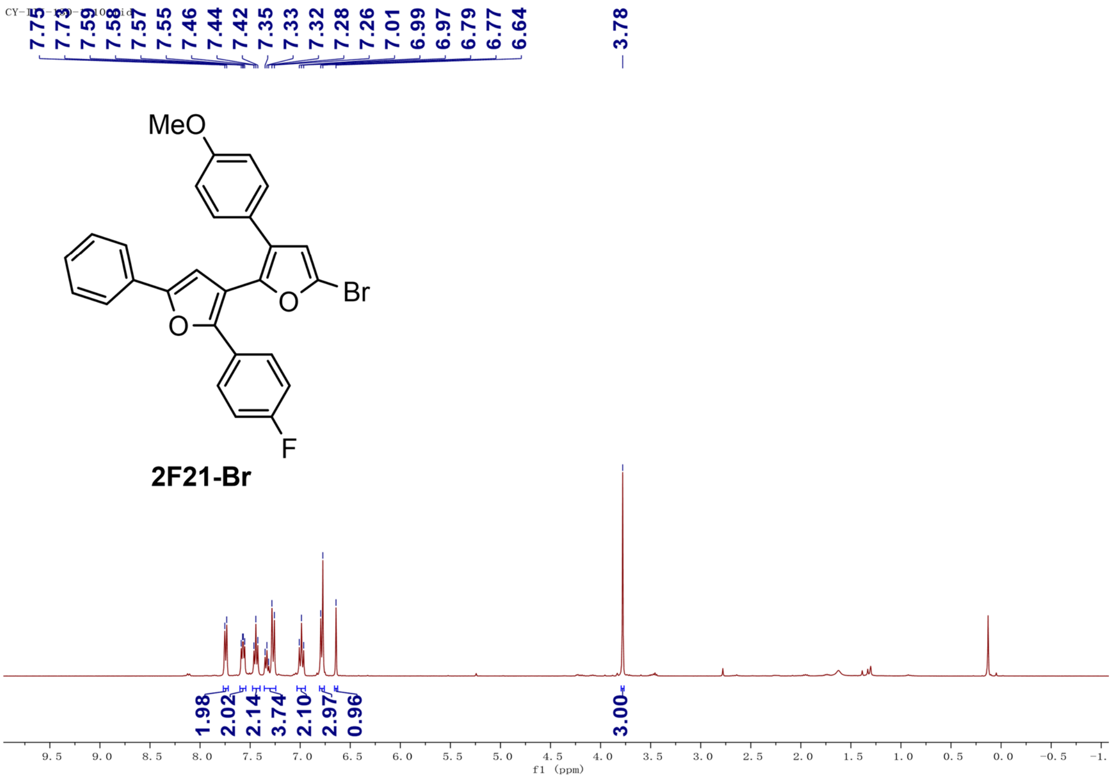

Supplementary Figure 213. ^{1}H NMR (400 MHz, $CDCl_3$) spectra for compound **2F21-Br**

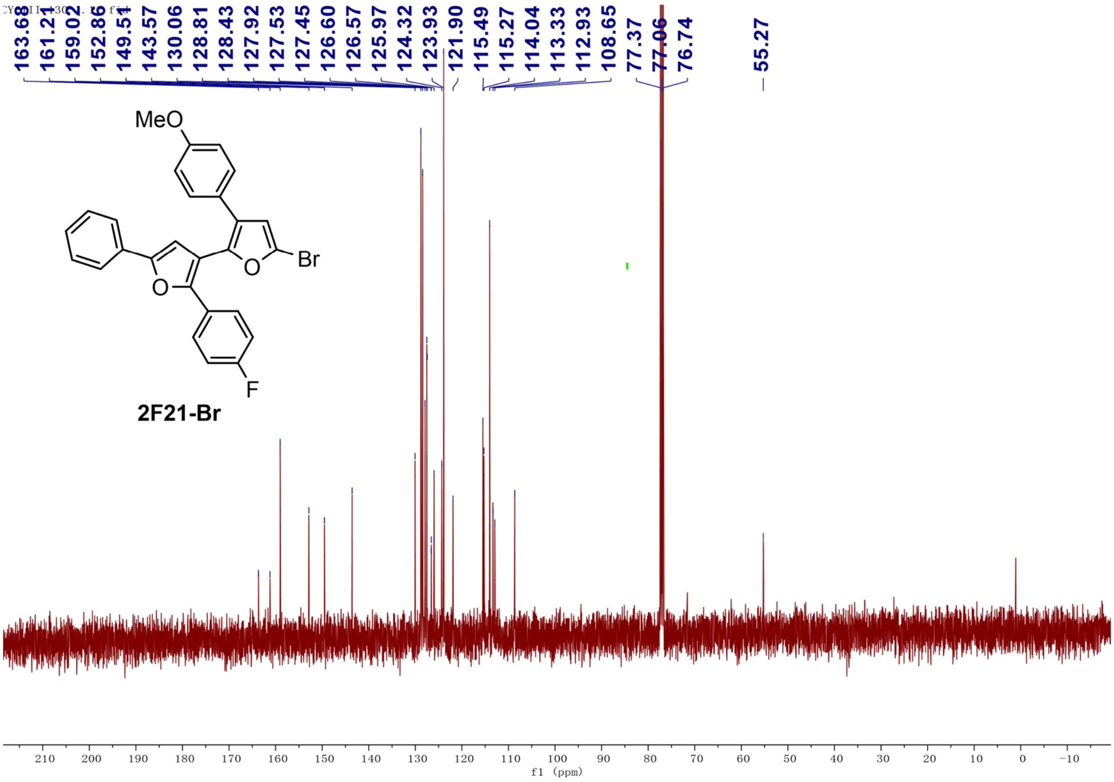

Supplementary Figure 214. ^{13}C NMR (101 MHz, $CDCl_3$) spectra for compound **2F21-Br**

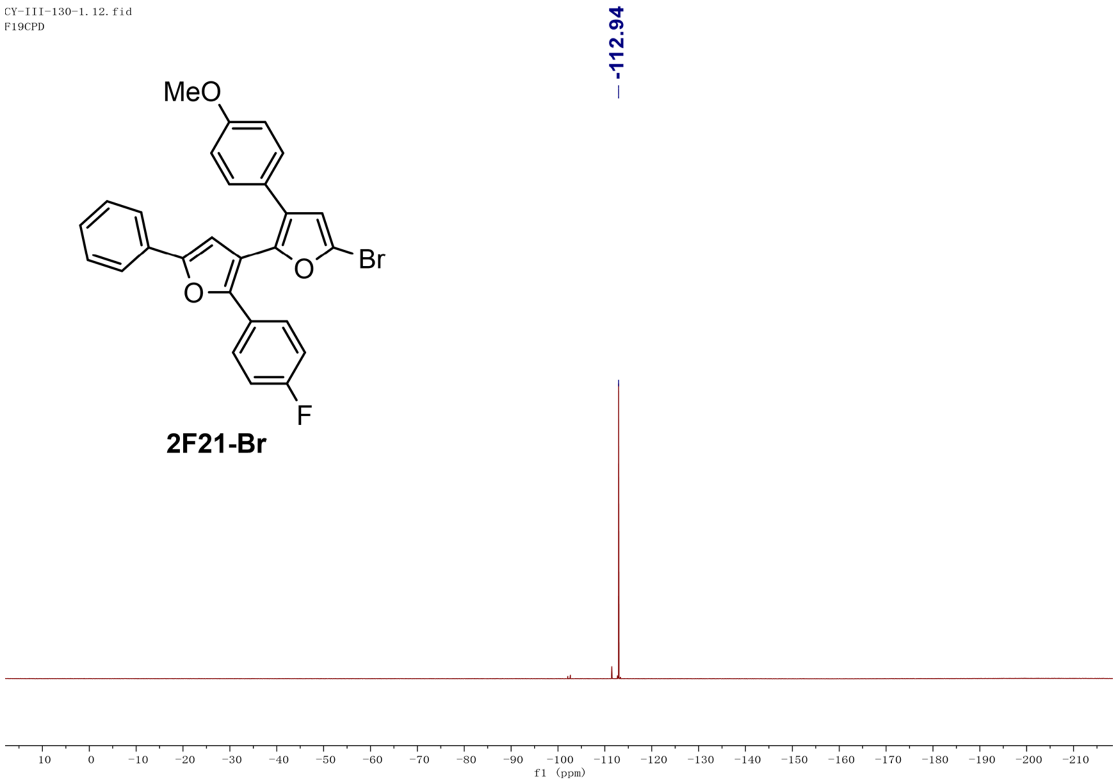

Supplementary Figure 215. ^{19}F NMR (376 MHz, $CDCl_3$) spectra for compound **2F21-Br**

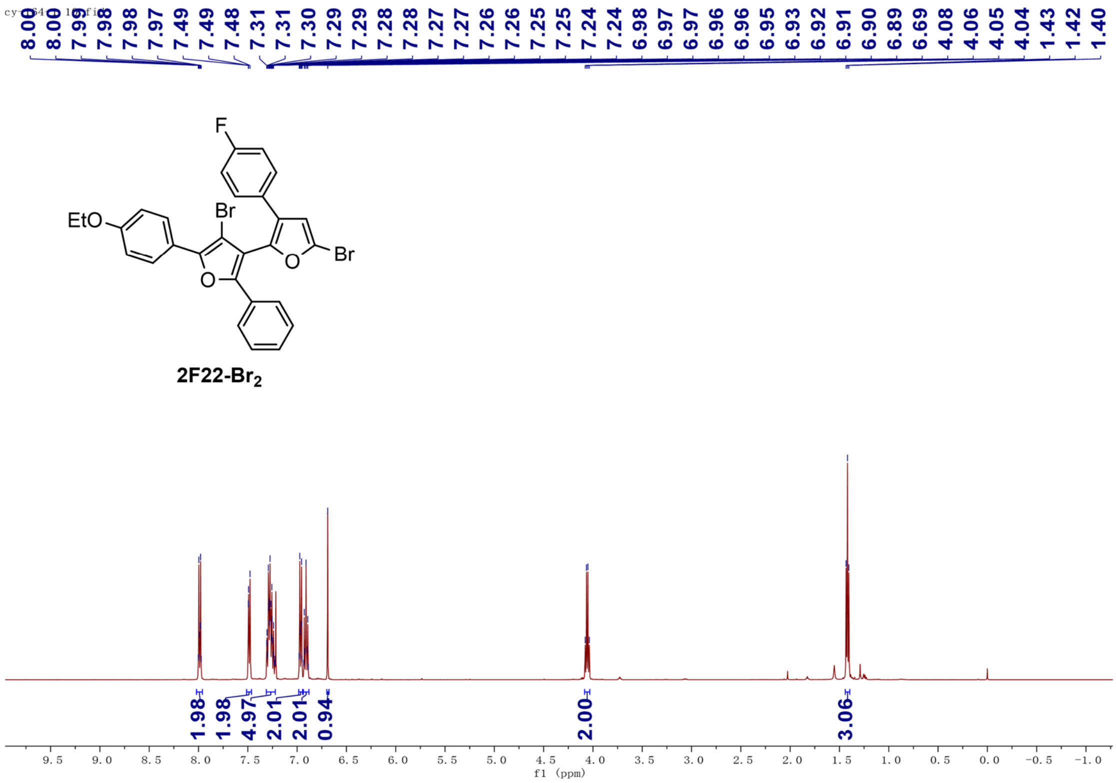

Supplementary Figure 216. ^{1}H NMR (500 MHz, $CDCl_3$) spectra for compound **2F22-Br₂**

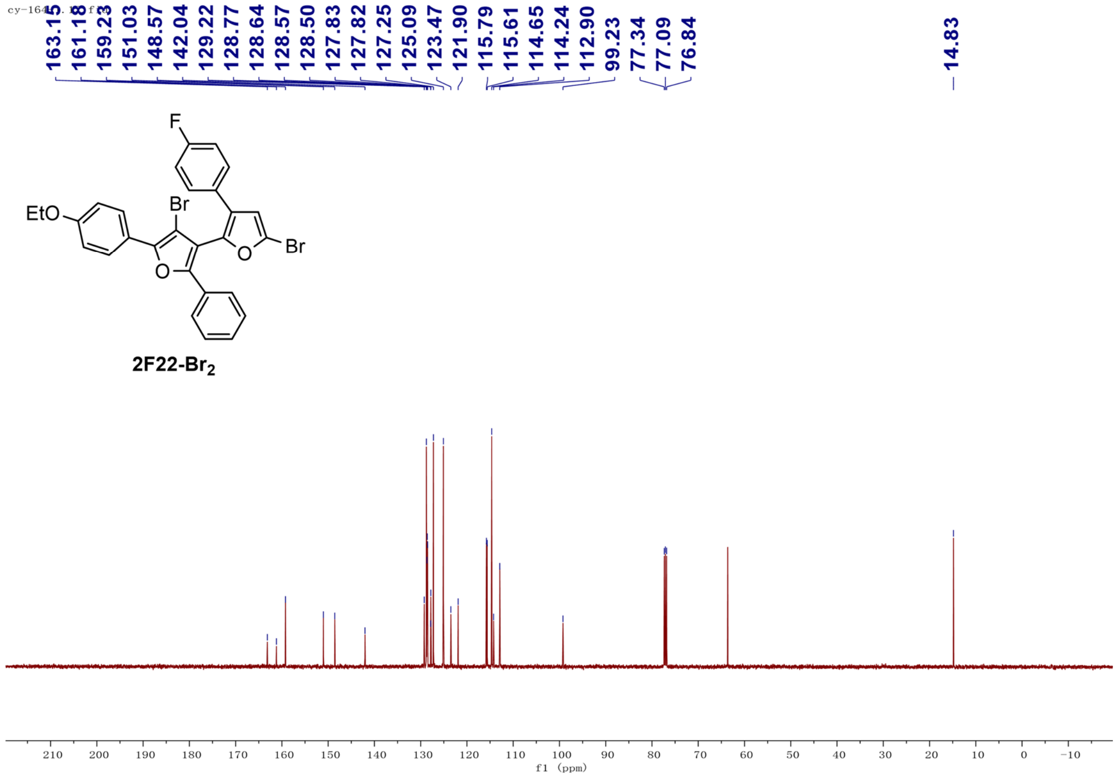

Supplementary Figure 217. ^{13}C NMR (126 MHz, $CDCl_3$) spectra for compound **2F22-Br₂**

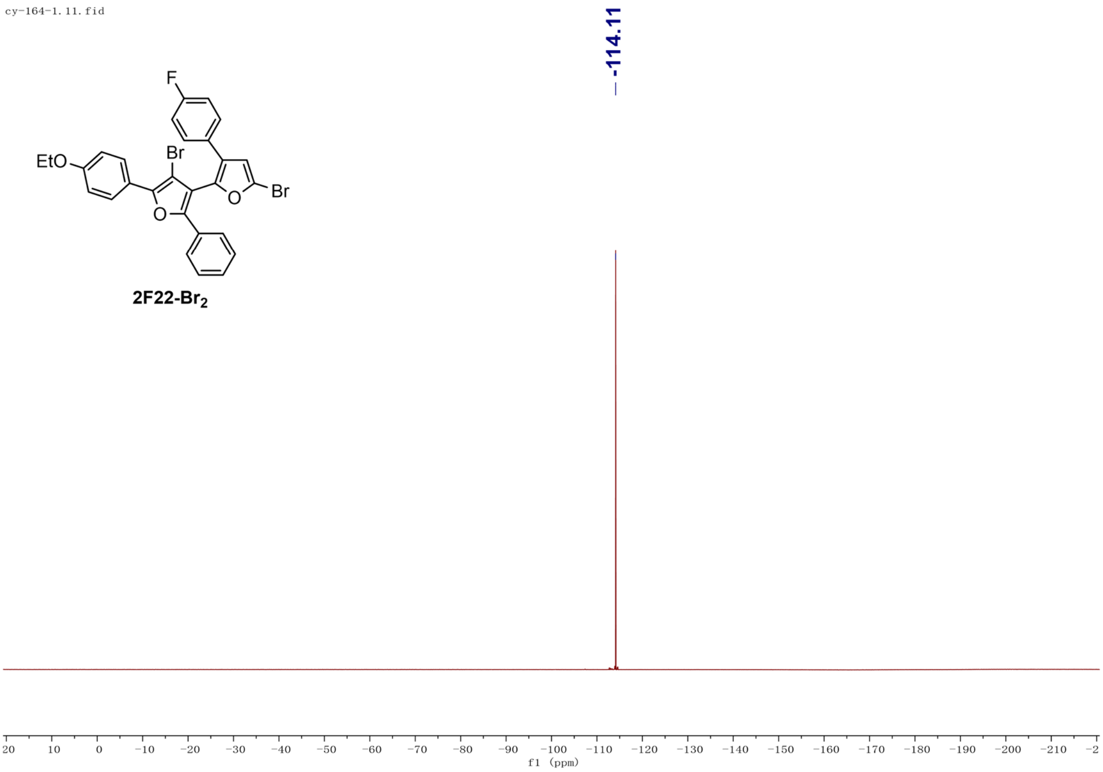

Supplementary Figure 218. ^{19}F NMR (471 MHz, $CDCl_3$) spectra for compound **2F22-Br$_2$**

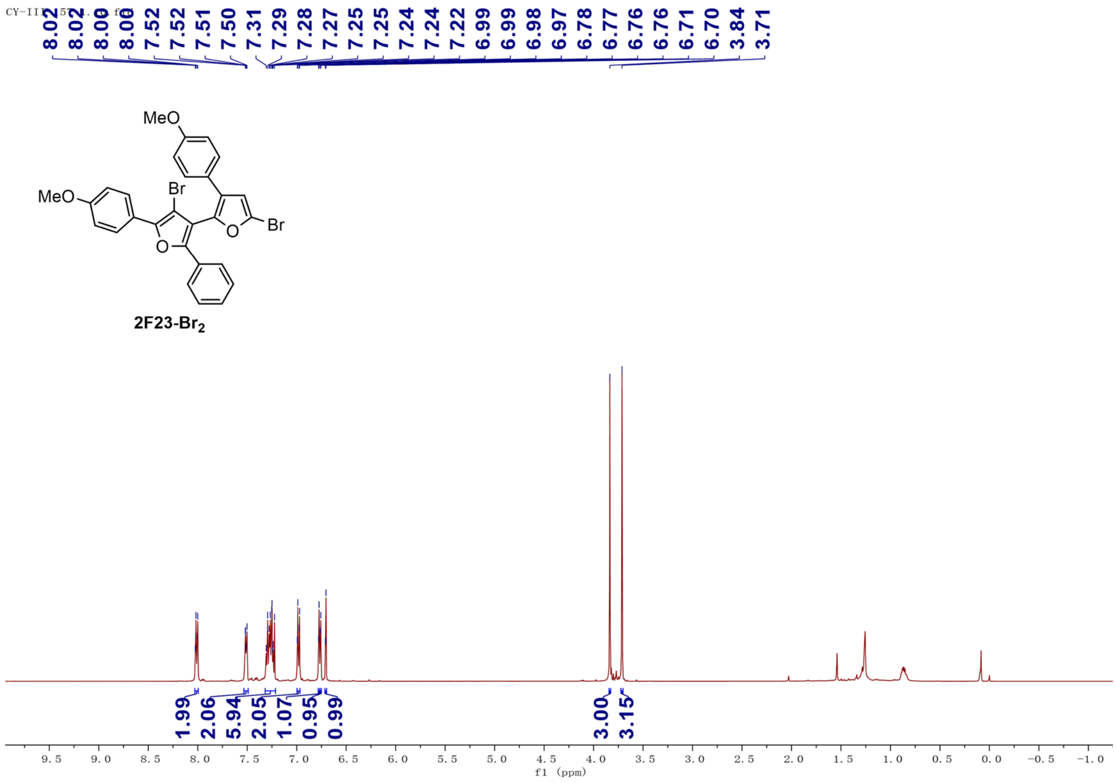

Supplementary Figure 219. ^{1}H NMR (500 MHz, $CDCl_3$) spectra for compound **2F23-Br$_2$**

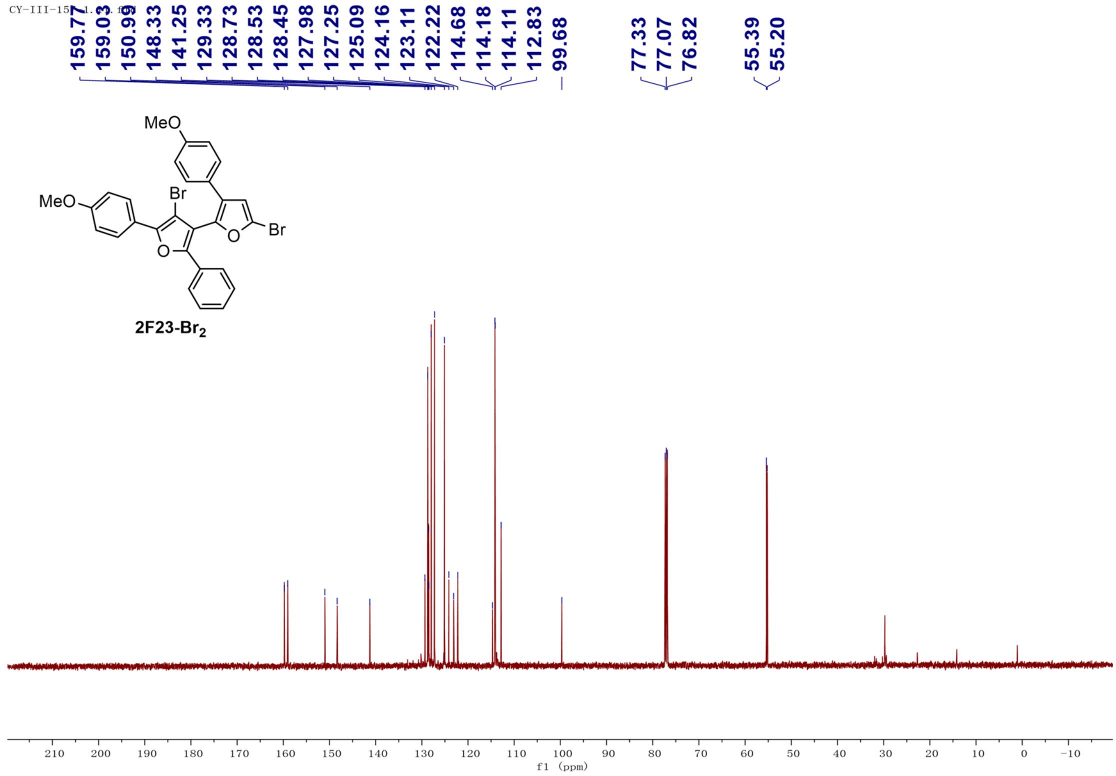

Supplementary Figure 220. ^{13}C NMR (126 MHz, $CDCl_3$) spectra for compound **2F23-Br$_2$**

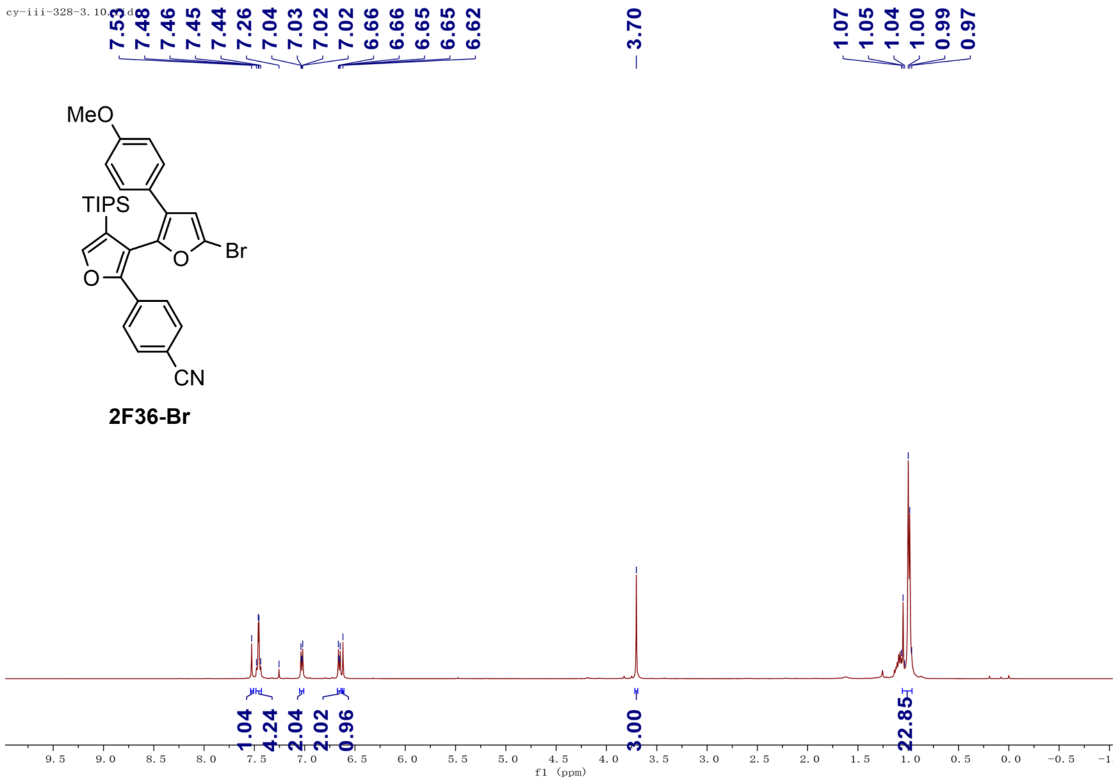

Supplementary Figure 221. ^{1}H NMR (500 MHz, $CDCl_3$) spectra for compound **2F36-Br**

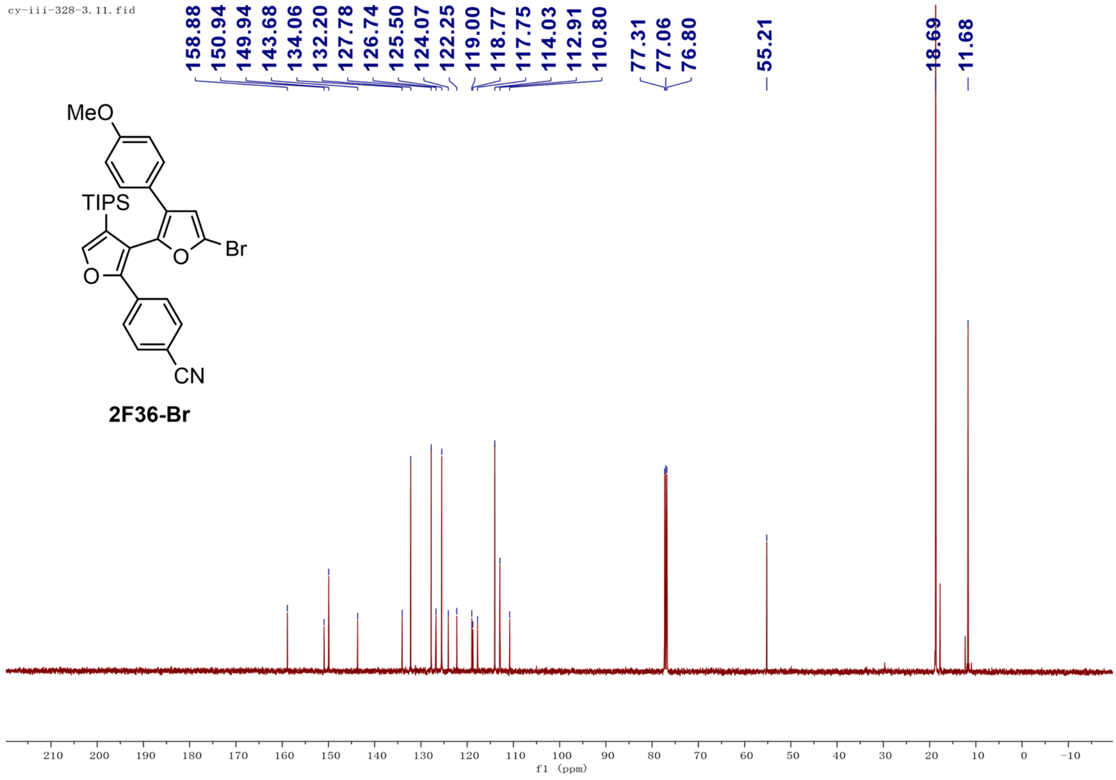

Supplementary Figure 222. ^{13}C NMR (126 MHz, $CDCl_3$) spectra for compound **2F36-Br**

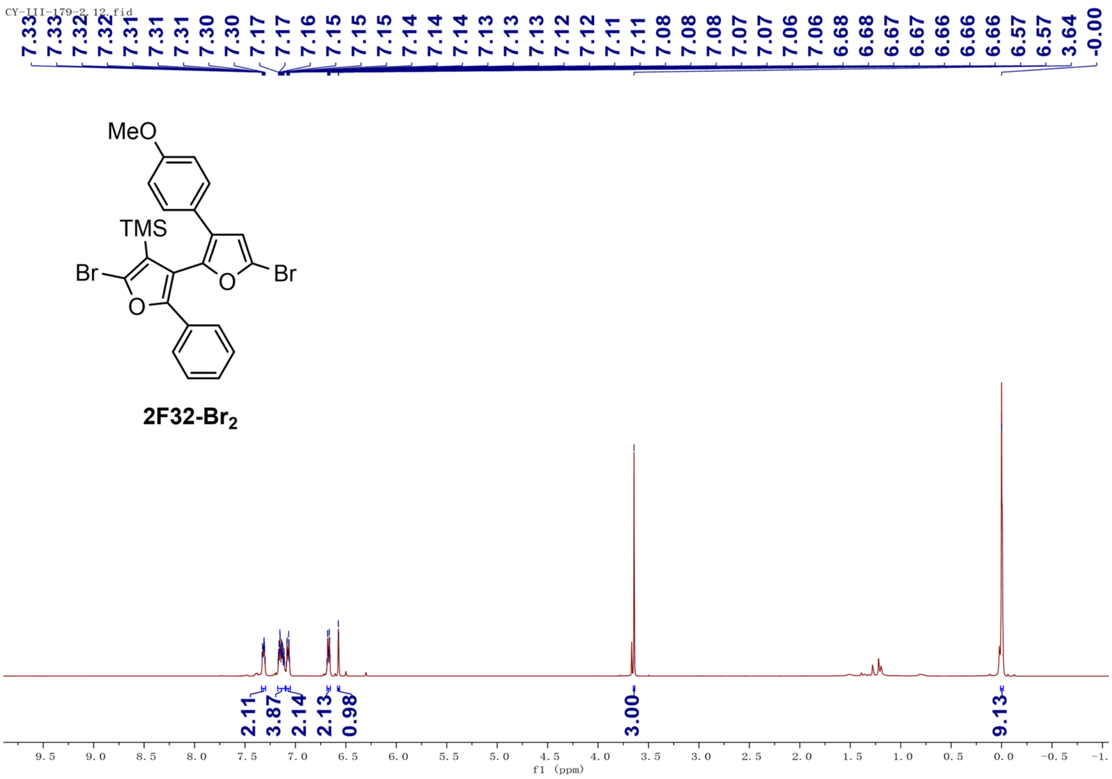

Supplementary Figure 223. ^{1}H NMR (500 MHz, $CDCl_3$) spectra for compound **2F32-Br₂**

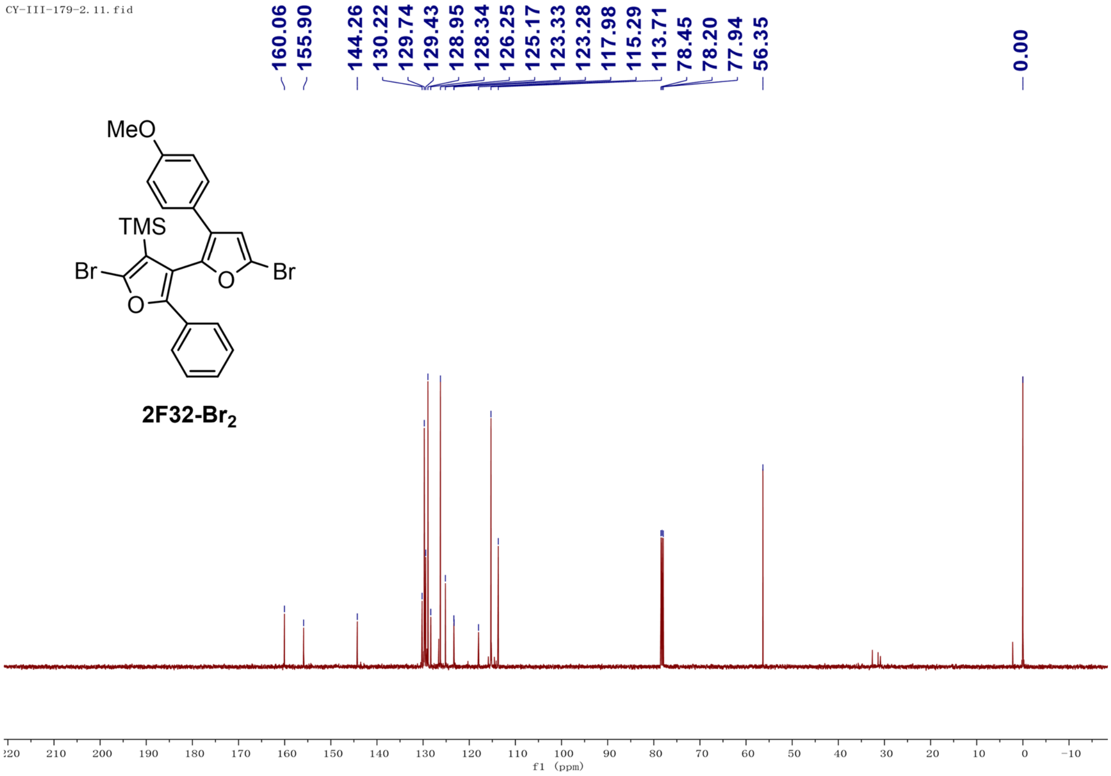

Supplementary Figure 224. ^{13}C NMR (126 MHz, $CDCl_3$) spectra for compound **2F32-Br₂**

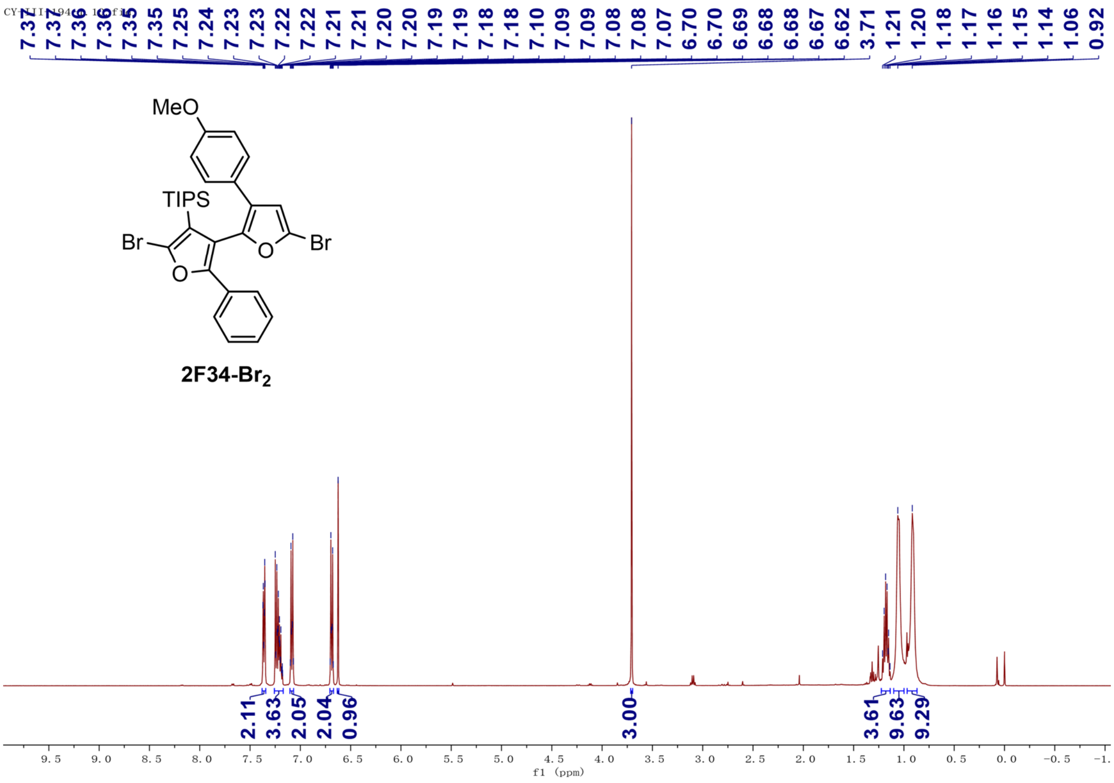

Supplementary Figure 225. ^{1}H NMR (500 MHz, $CDCl_3$) spectra for compound **2F34-Br$_2$**

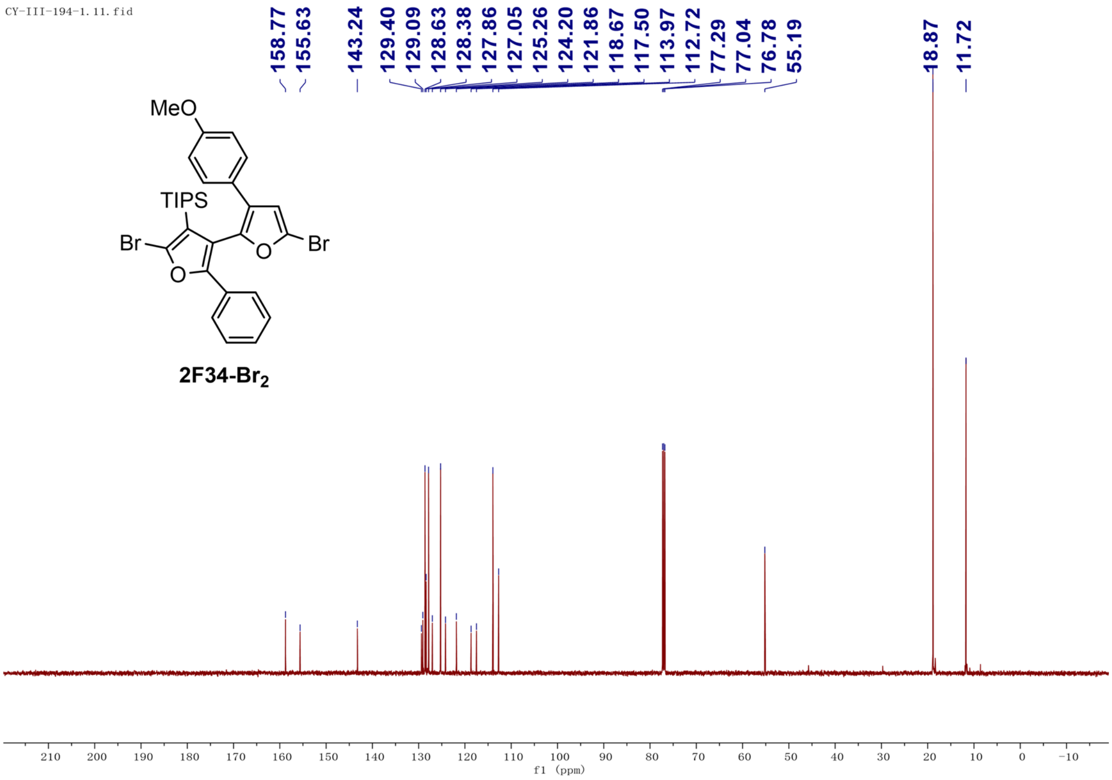

Supplementary Figure 226. ^{13}C NMR (126 MHz, $CDCl_3$) spectra for compound **2F34-Br$_2$**

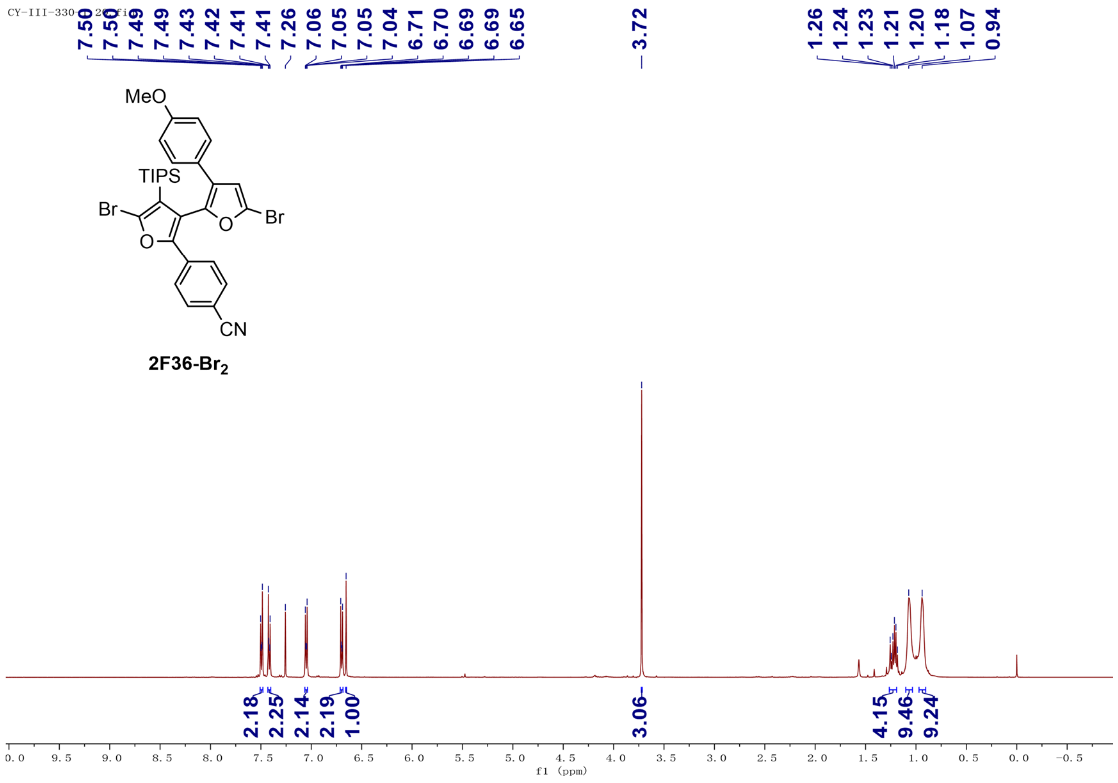

Supplementary Figure 227. ^{1}H NMR (500 MHz, $CDCl_3$) spectra for compound **2F36-Br$_2$**

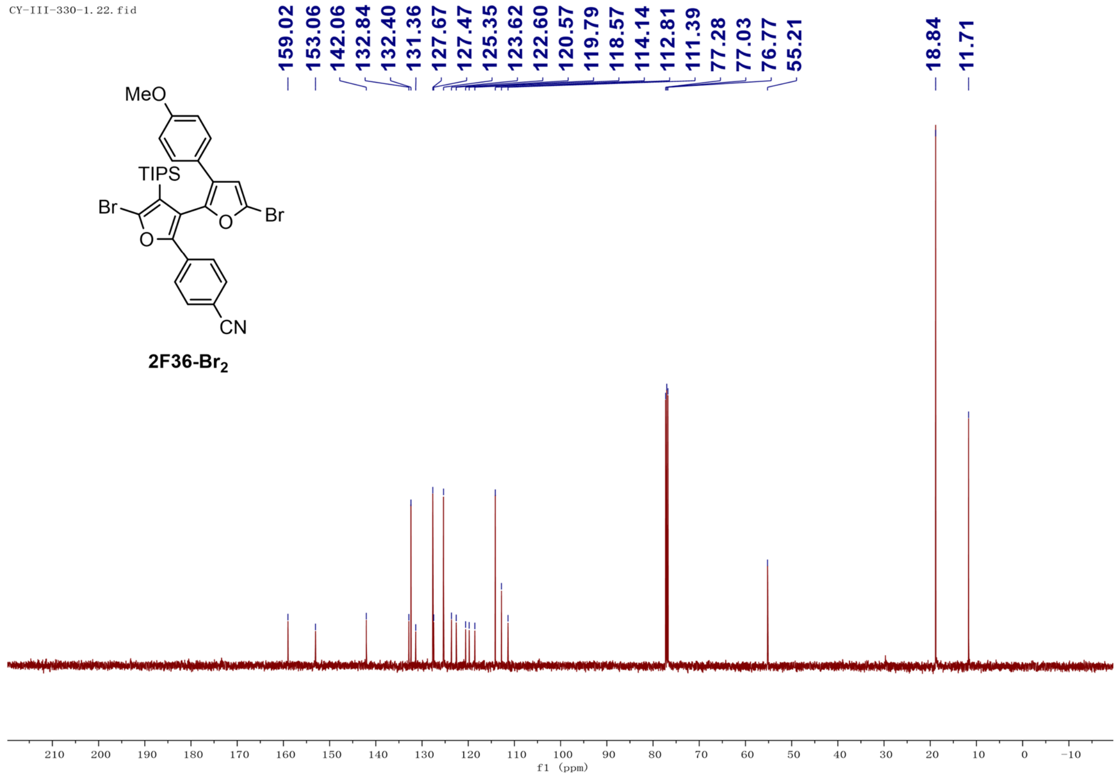

Supplementary Figure 228. ^{13}C NMR (126 MHz, $CDCl_3$) spectra for compound **2F36-Br$_2$**

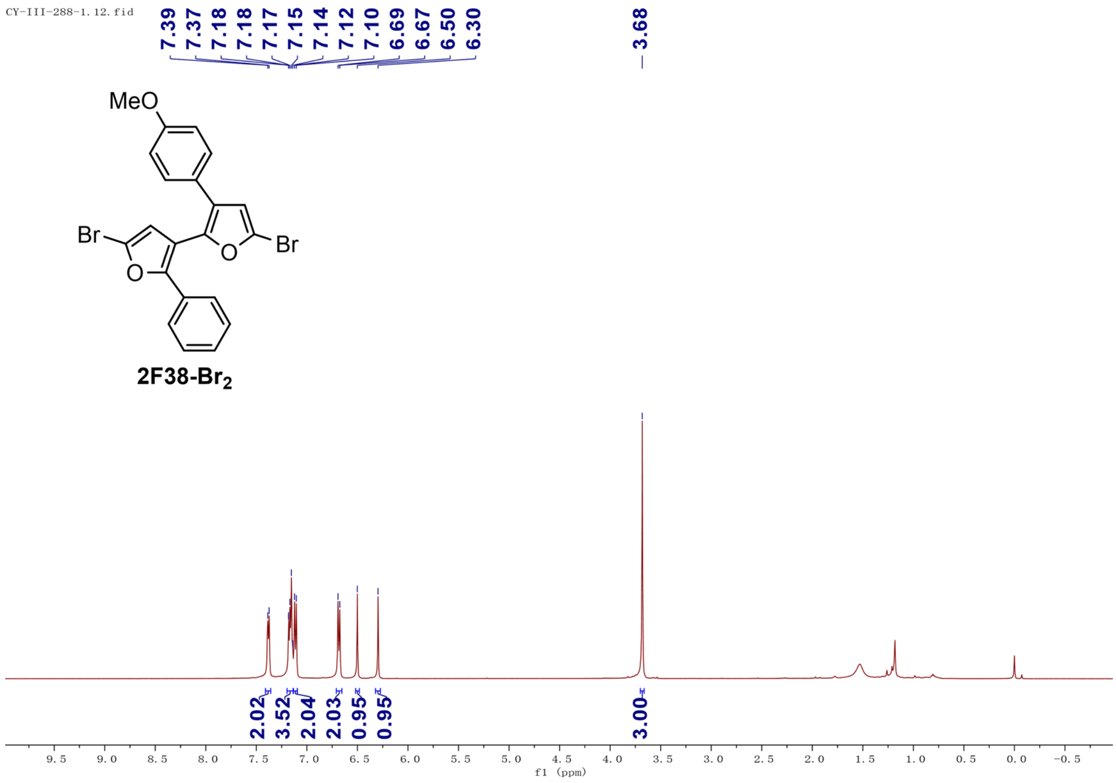

Supplementary Figure 229. ^{1}H NMR (500 MHz, $CDCl_3$) spectra for compound **2F38-Br$_2$**

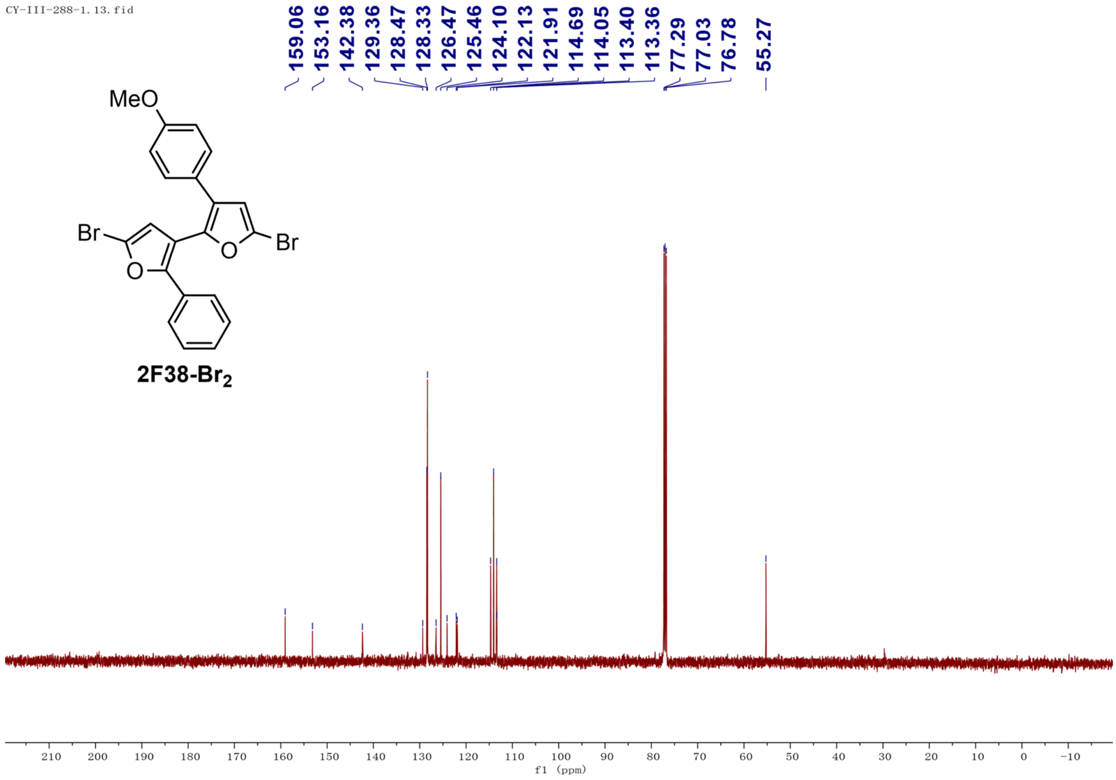

Supplementary Figure 230. ^{13}C NMR (126 MHz, $CDCl_3$) spectra for compound **2F38-Br$_2$**

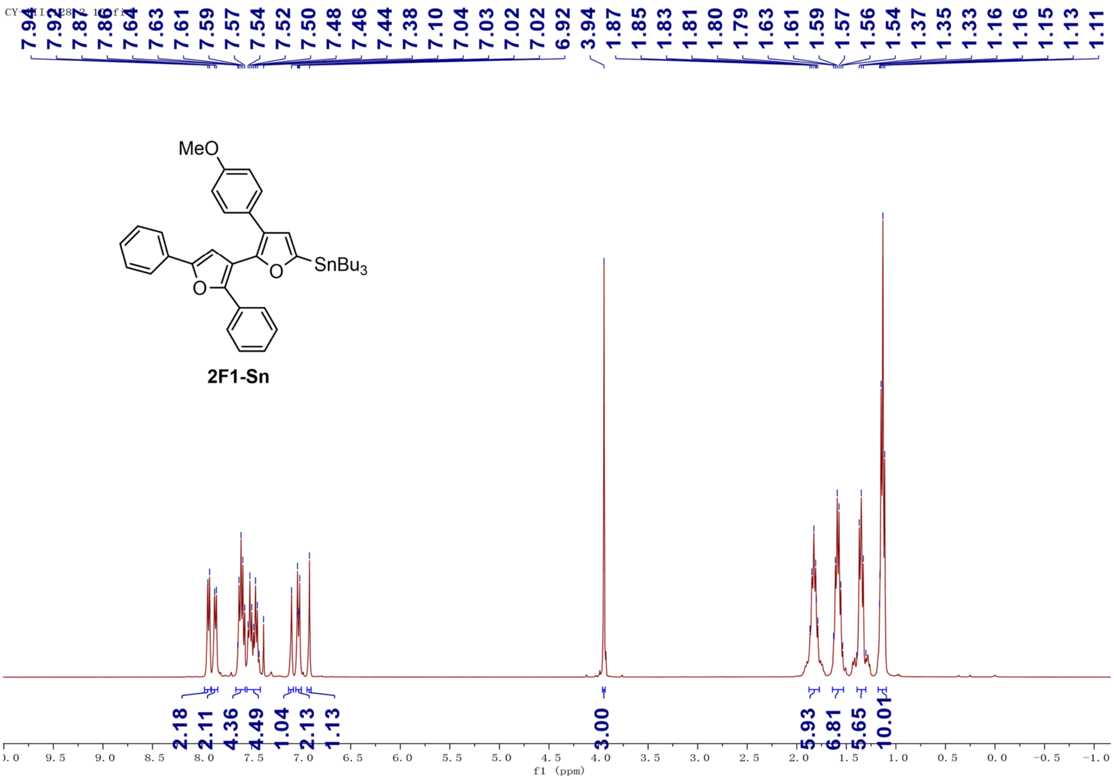

Supplementary Figure 231. ^{1}H NMR (400 MHz, $CDCl_3$) spectra for compound **2F1-Sn**

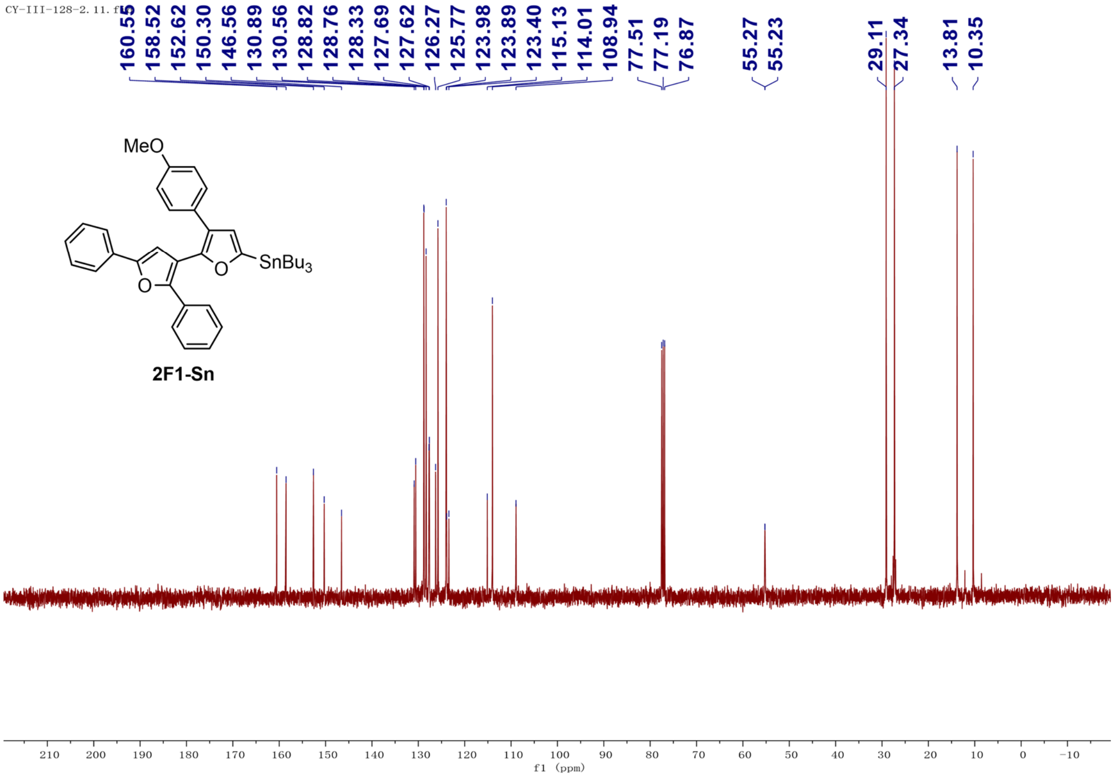

Supplementary Figure 232. ^{13}C NMR (101 MHz, $CDCl_3$) spectra for compound **2F1-Sn**

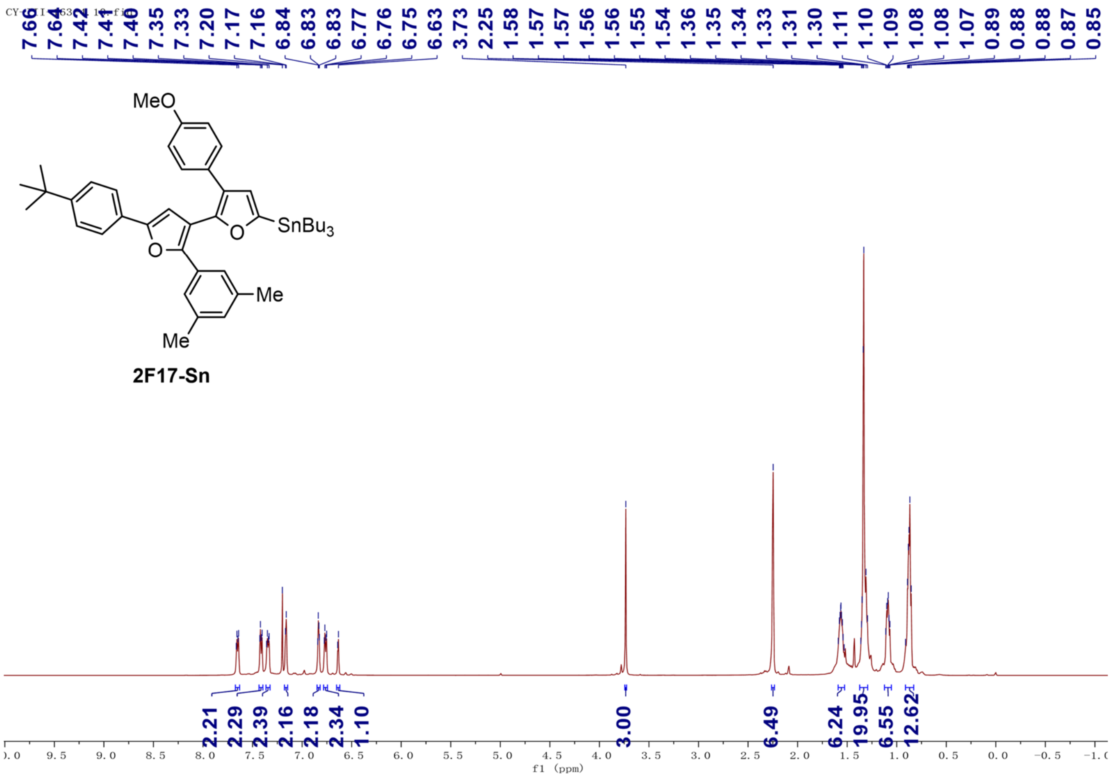

Supplementary Figure 233. ^{1}H NMR (500 MHz, $CDCl_3$) spectra for compound **2F17-Sn**

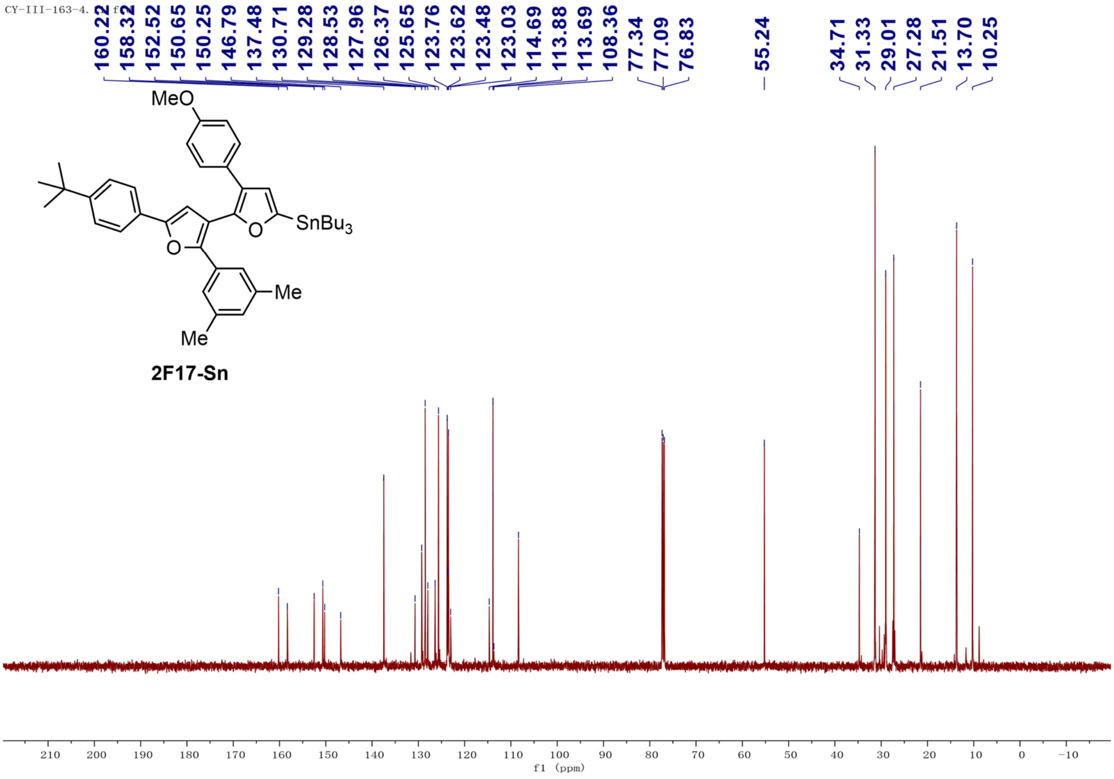

Supplementary Figure 234. ^{13}C NMR (126 MHz, $CDCl_3$) spectra for compound **2F17-Sn**

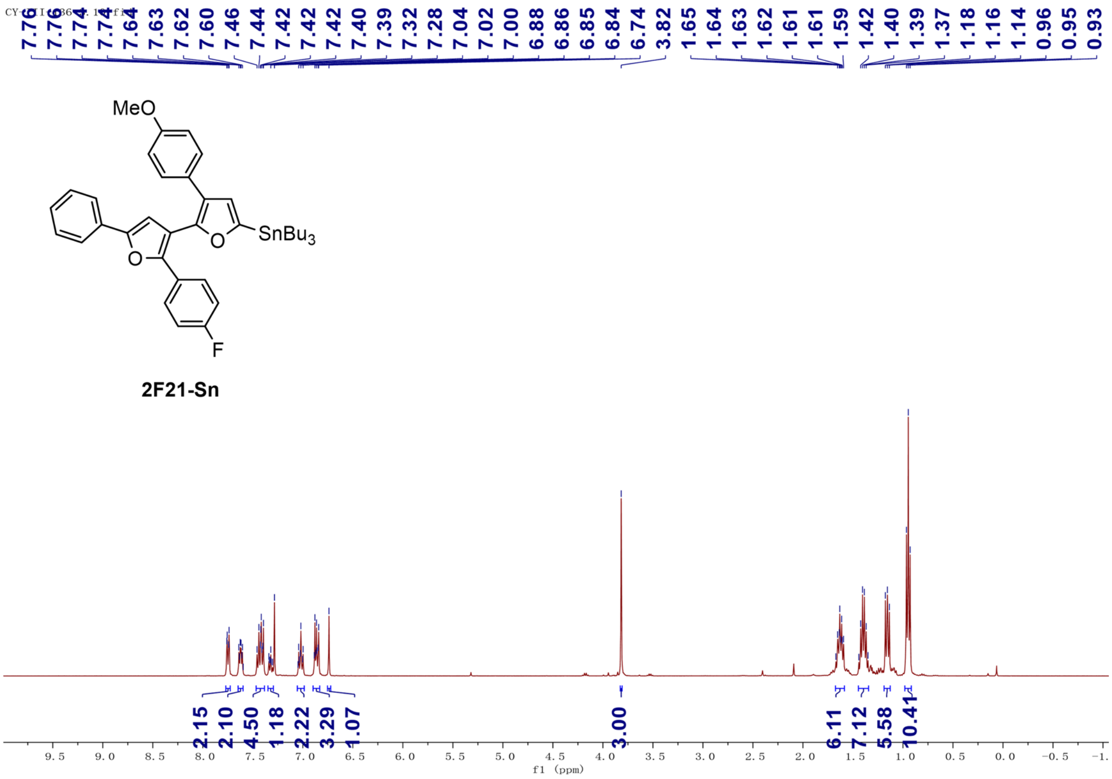

Supplementary Figure 235. ^{1}H NMR (400 MHz, $CDCl_3$) spectra for compound **2F21-Sn**

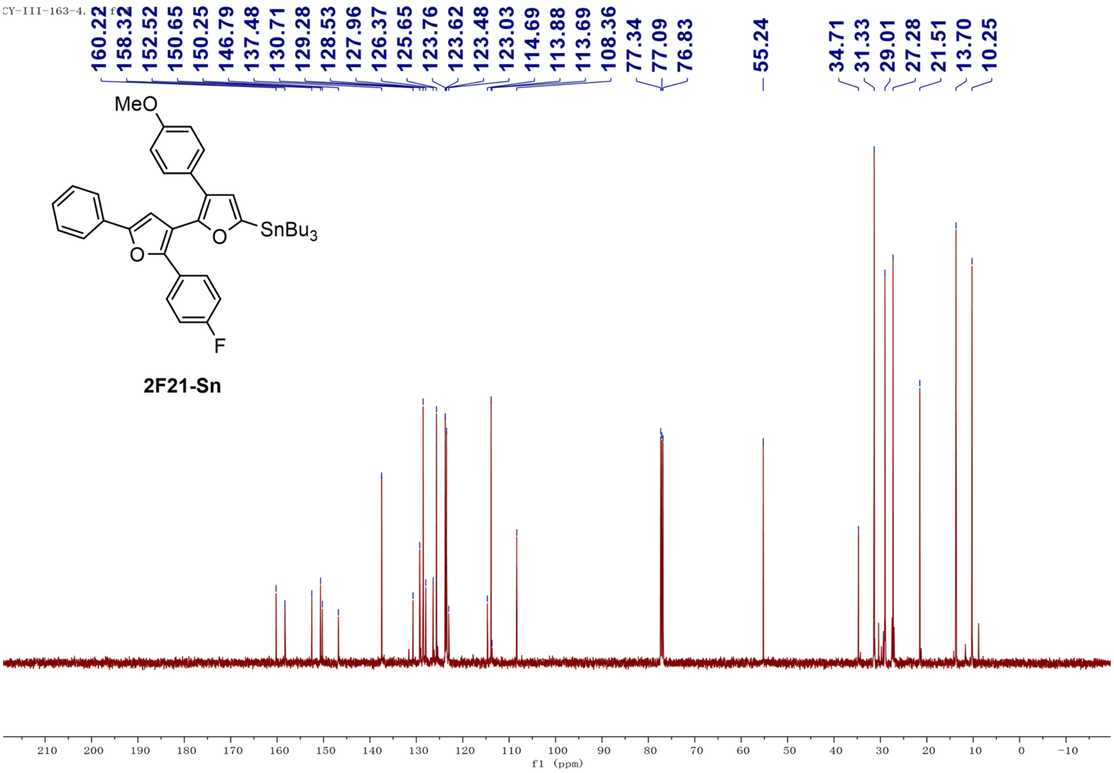

Supplementary Figure 236. ^{13}C NMR (101 MHz, $CDCl_3$) spectra for compound **2F21-Sn**

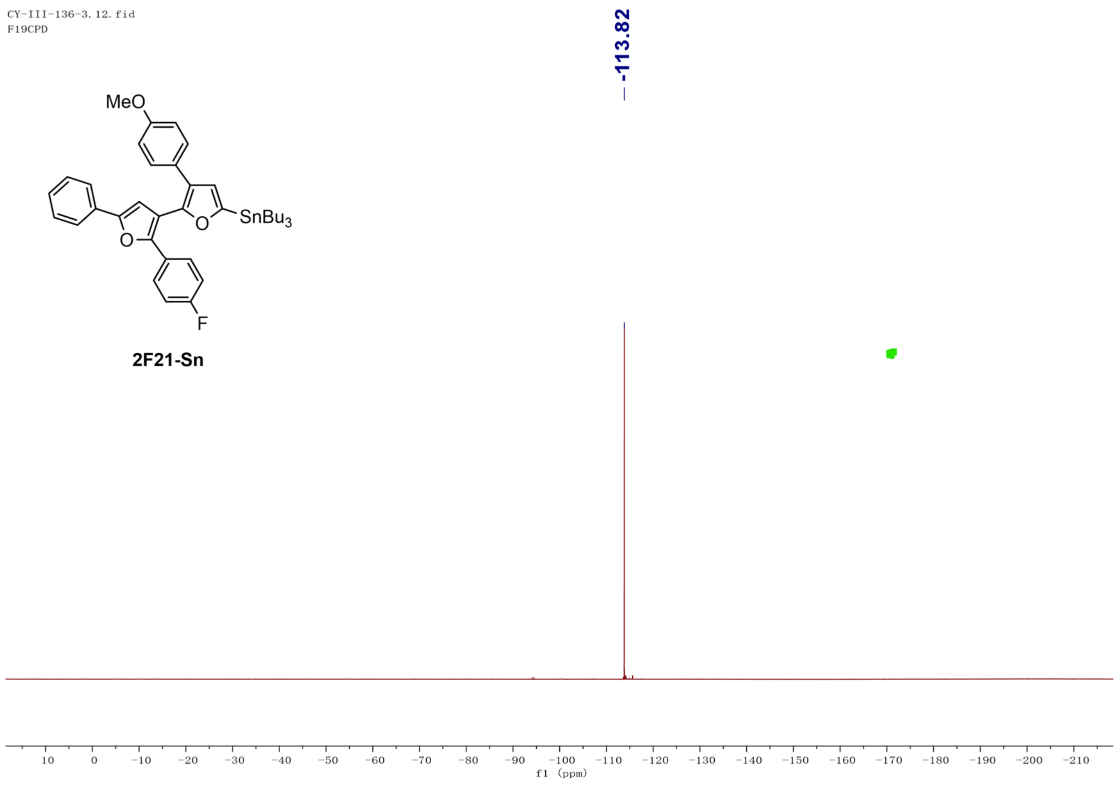

Supplementary Figure 237. ^{19}FNMR (376 MHz, $CDCl_3$) spectra for compound **2F21-Sn**

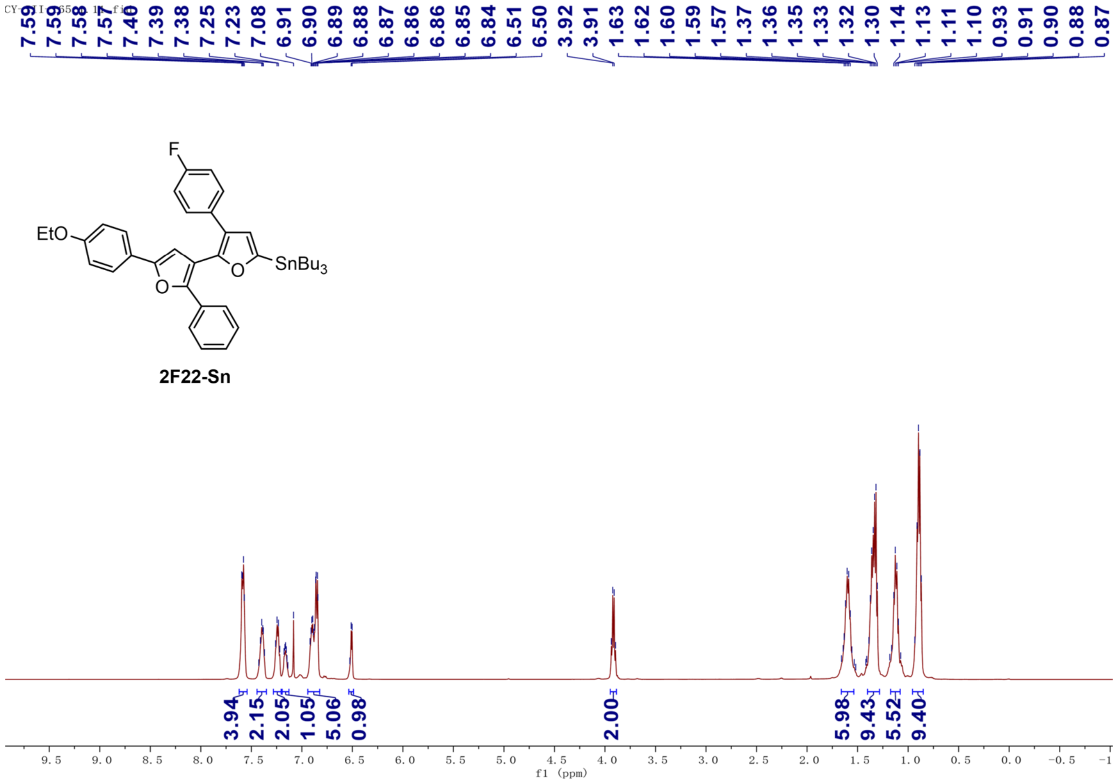

Supplementary Figure 238. ^{1}H NMR (500 MHz, $CDCl_3$) spectra for compound **2F22-Sn**

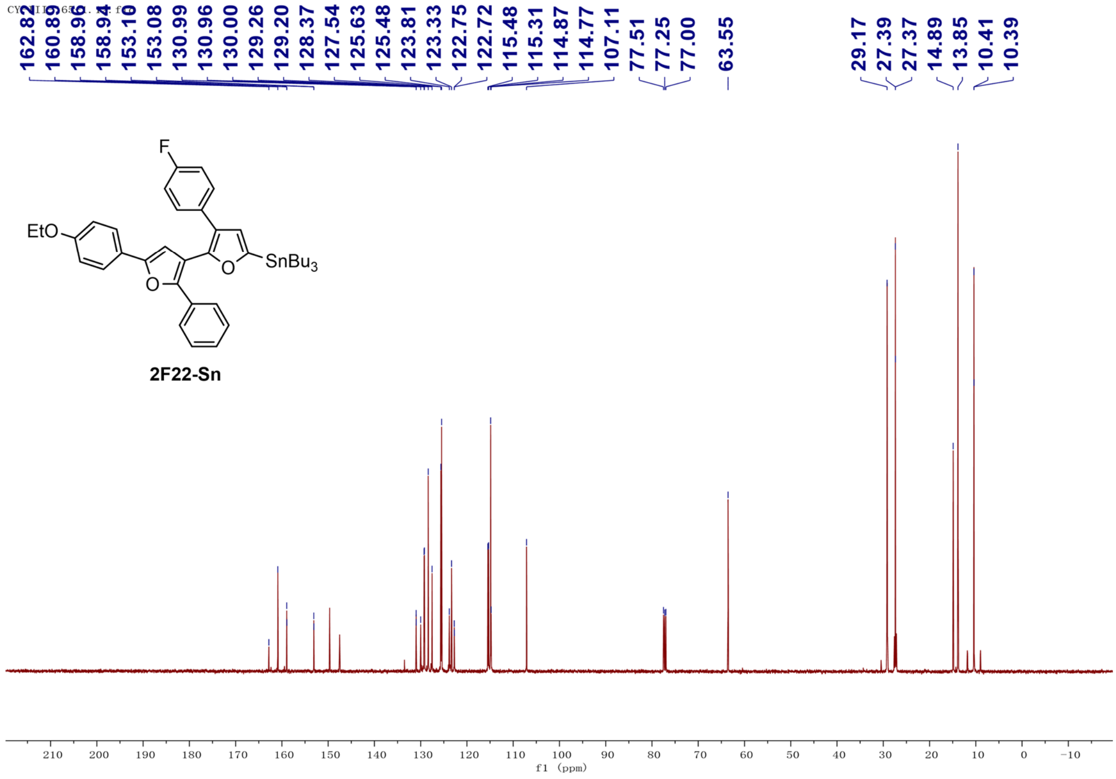

Supplementary Figure 239. ^{13}C NMR (126 MHz, $CDCl_3$) spectra for compound **2F22-Sn**

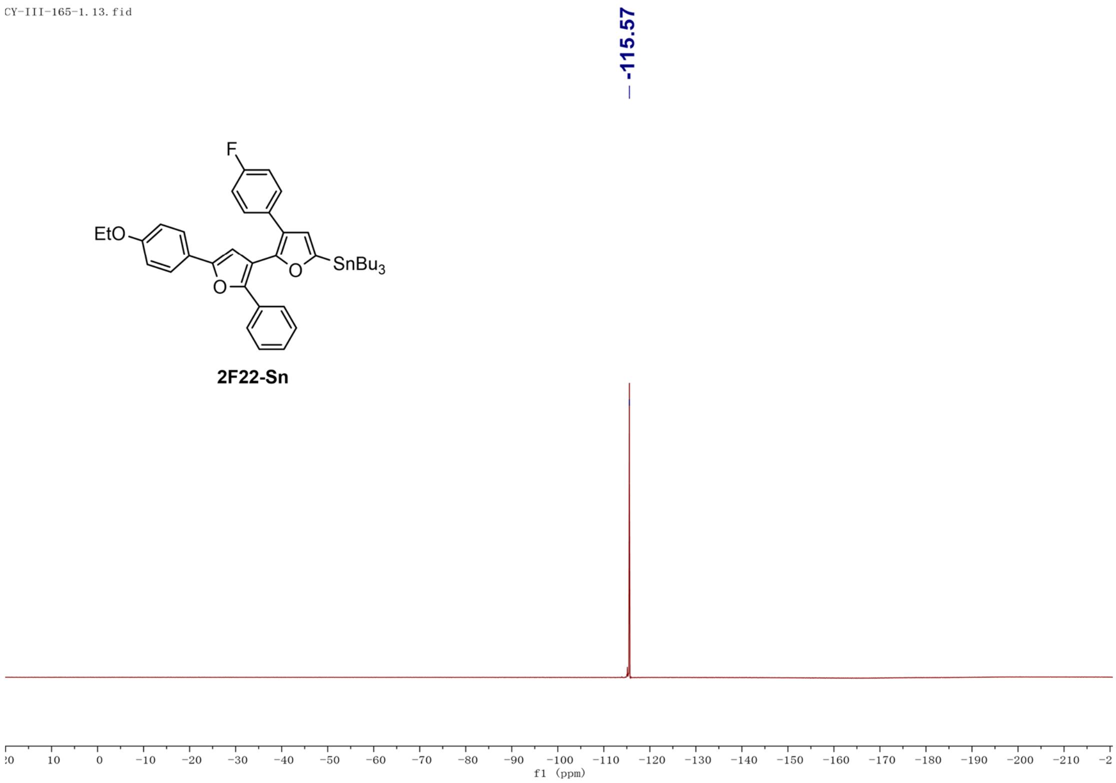

Supplementary Figure 240. ^{19}FNMR (471 MHz, $CDCl_3$) spectra for compound **2F22-Sn**

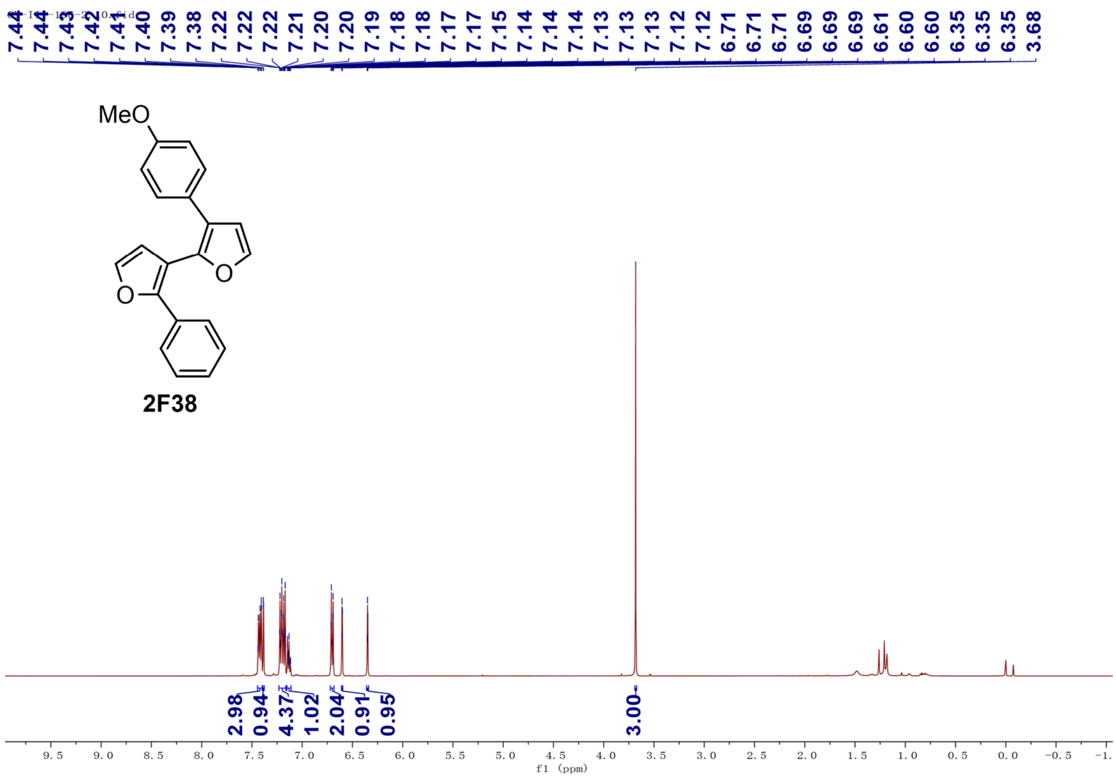

Supplementary Figure 241. ^{1}H NMR (500 MHz, $CDCl_3$) spectra for compound **2F38**

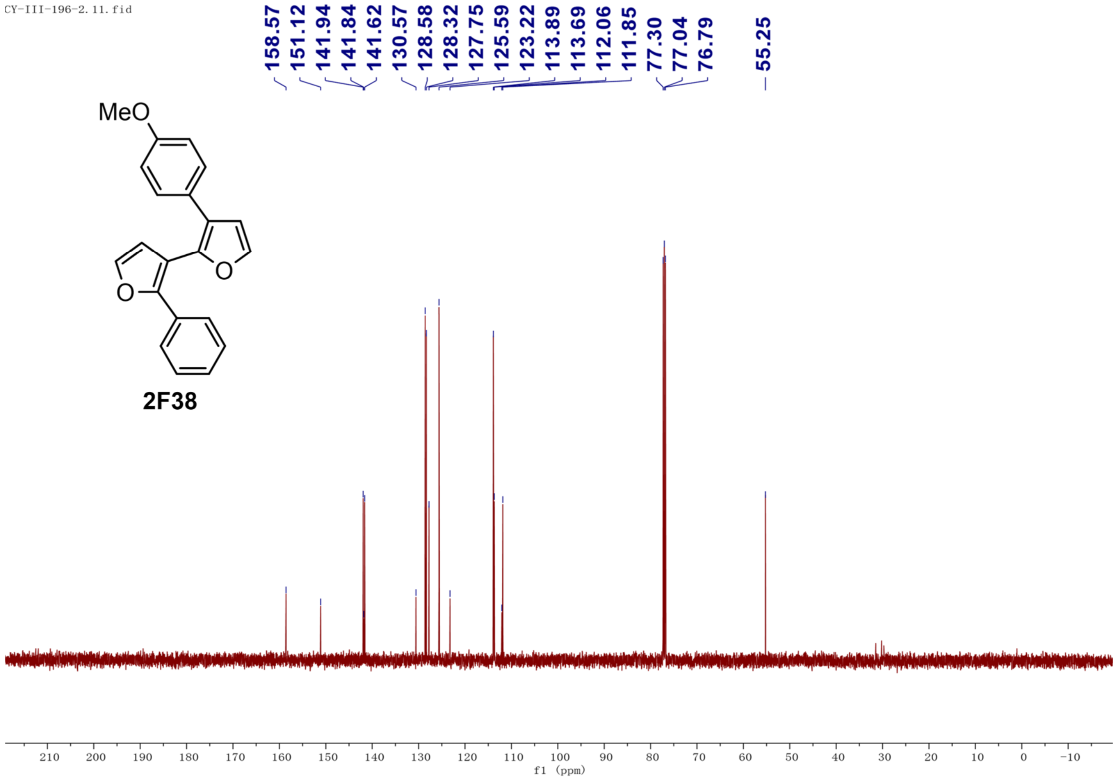

Supplementary Figure 242. ^{13}C NMR (126 MHz, $CDCl_3$) spectra for compound **2F38**

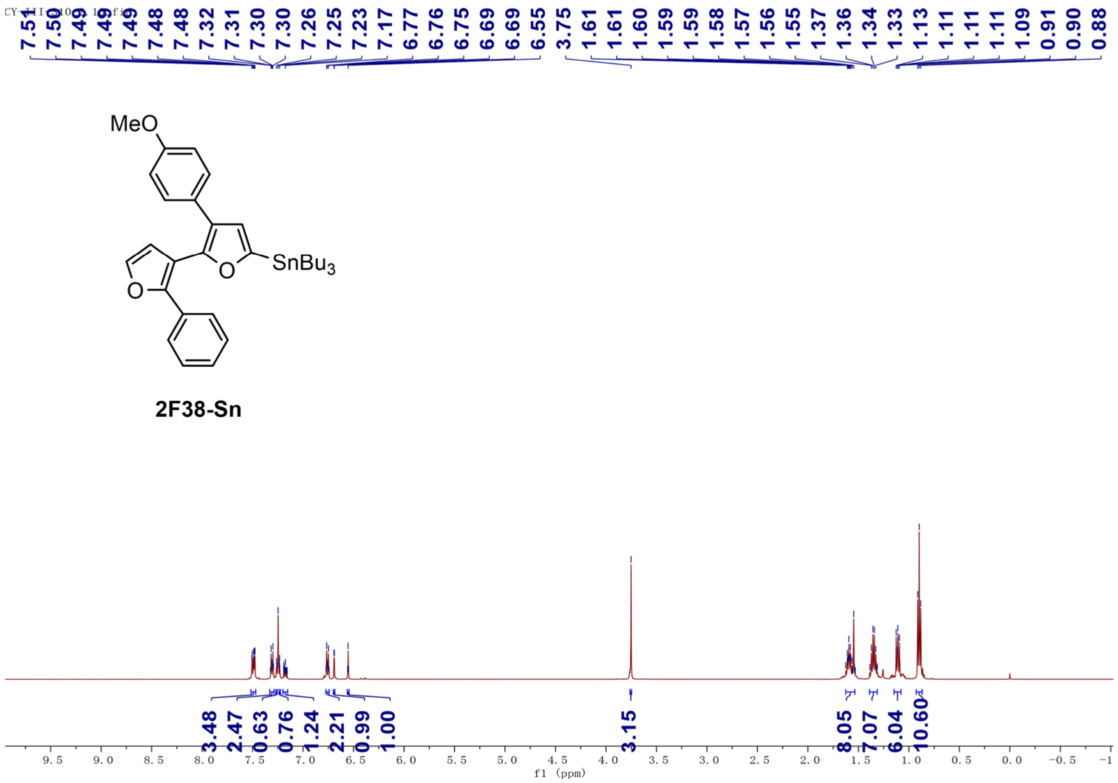

Supplementary Figure 243. ^{1}H NMR (500 MHz, $CDCl_3$) spectra for compound **2F38-Sn**

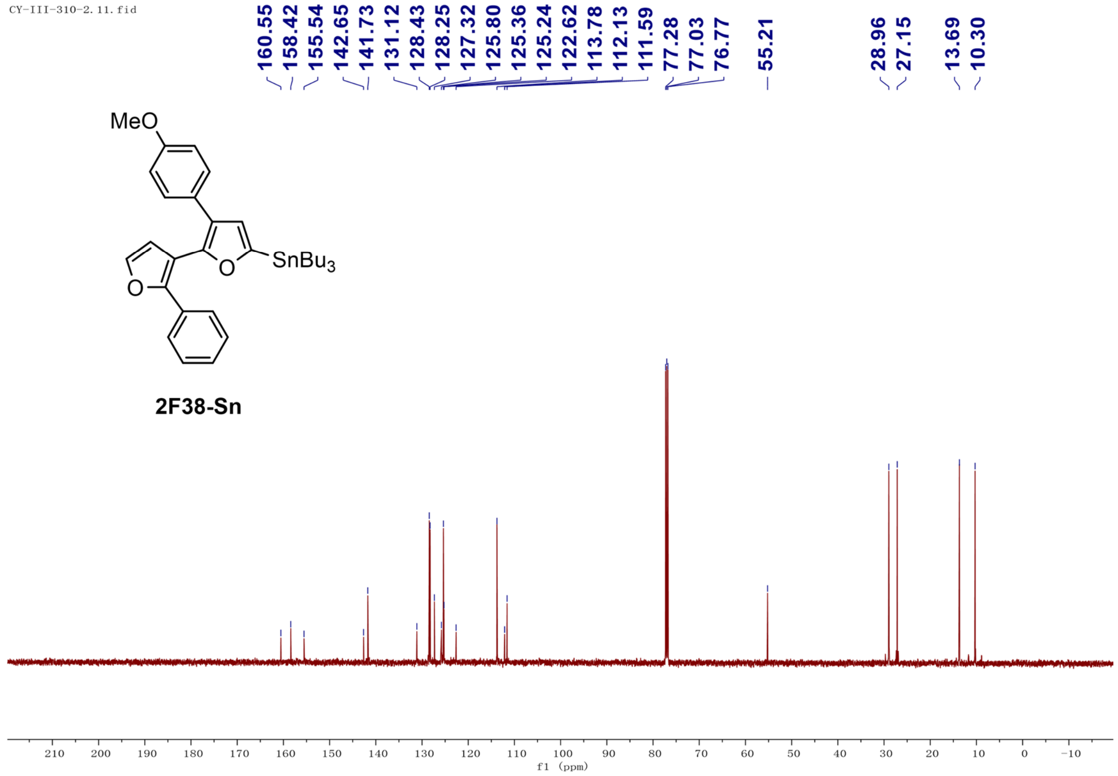

Supplementary Figure 244. ^{13}C NMR (126 MHz, $CDCl_3$) spectra for compound **2F38-Sn**

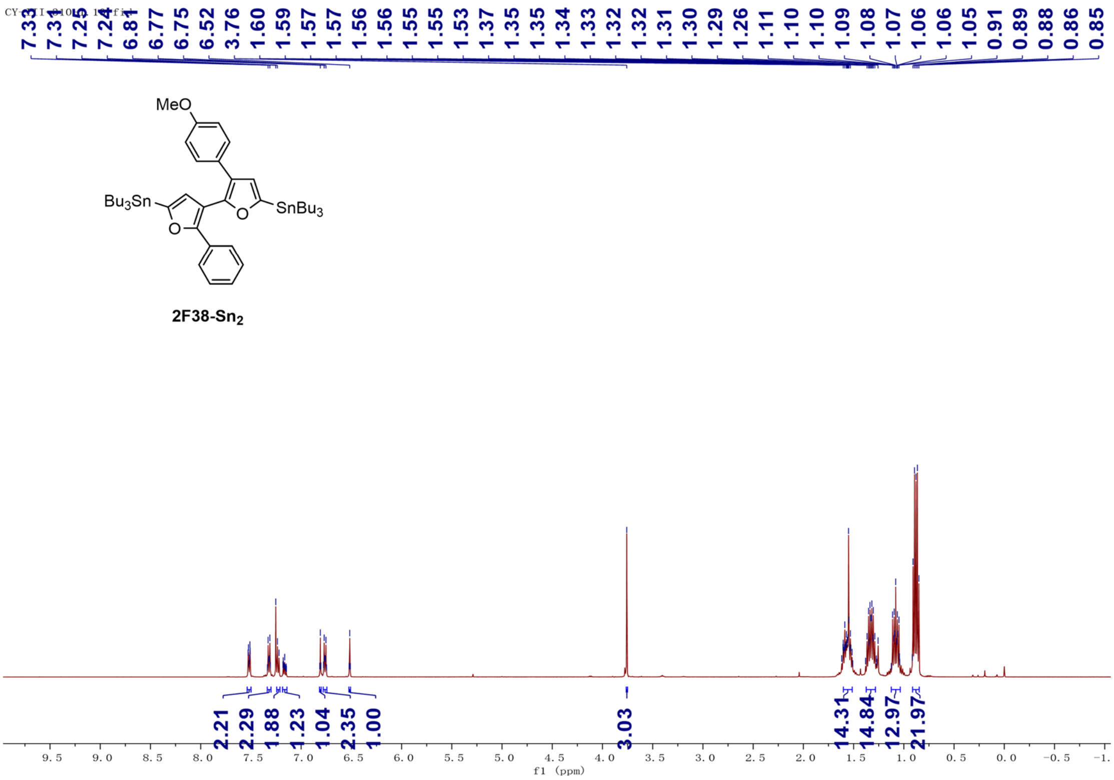

Supplementary Figure 245. ^{1}H NMR (500 MHz, $CDCl_3$) spectra for compound **2F38-Sn$_2$**

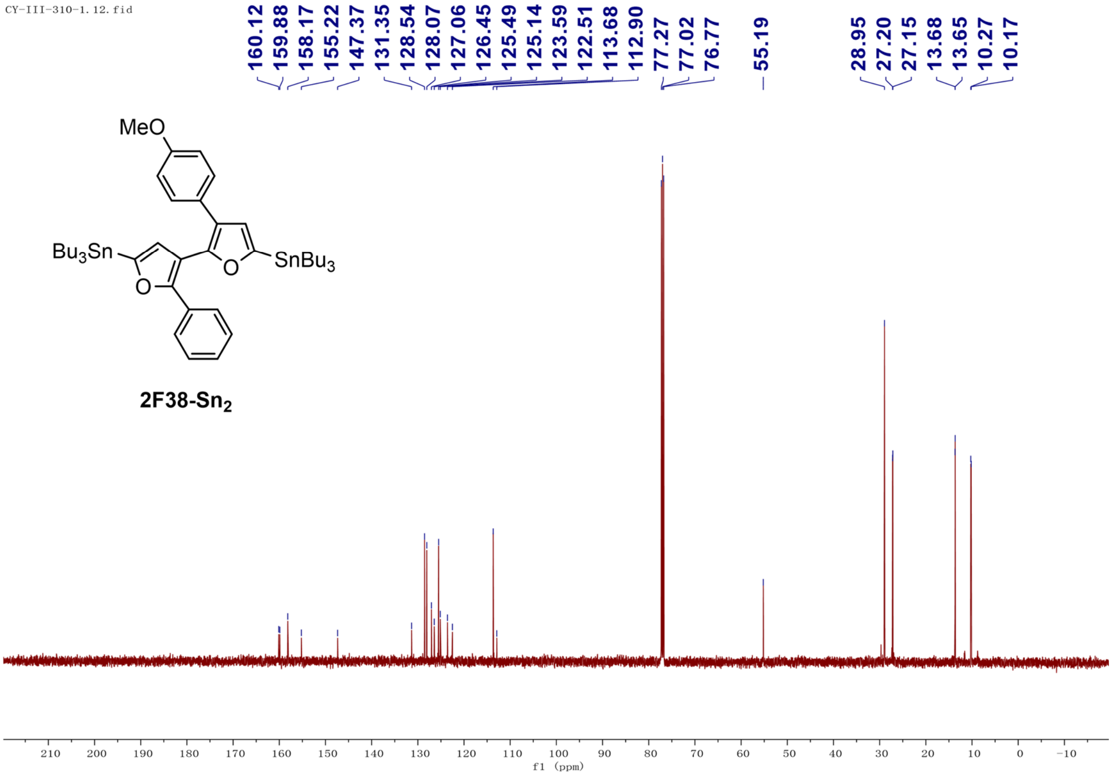

Supplementary Figure 246. ^{13}C NMR (126 MHz, $CDCl_3$) spectra for compound **2F38-Sn$_2$**

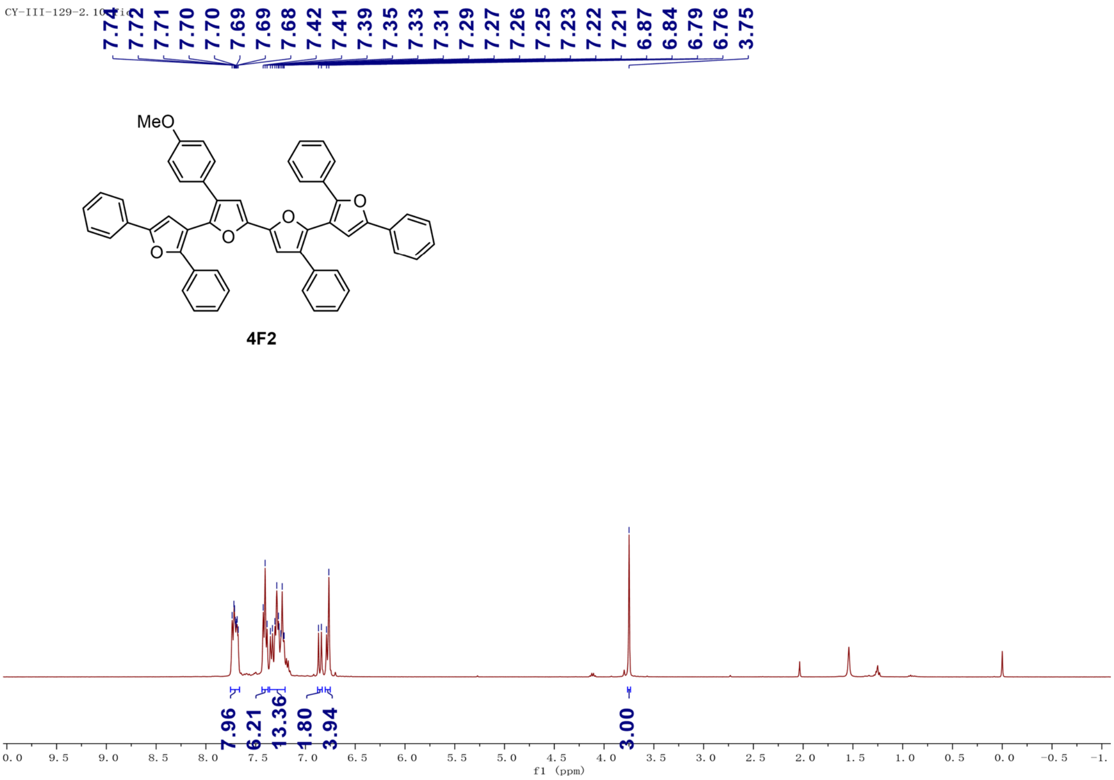

Supplementary Figure 247. ^{1}H NMR (400 MHz, $CDCl_3$) spectra for compound **4F2**

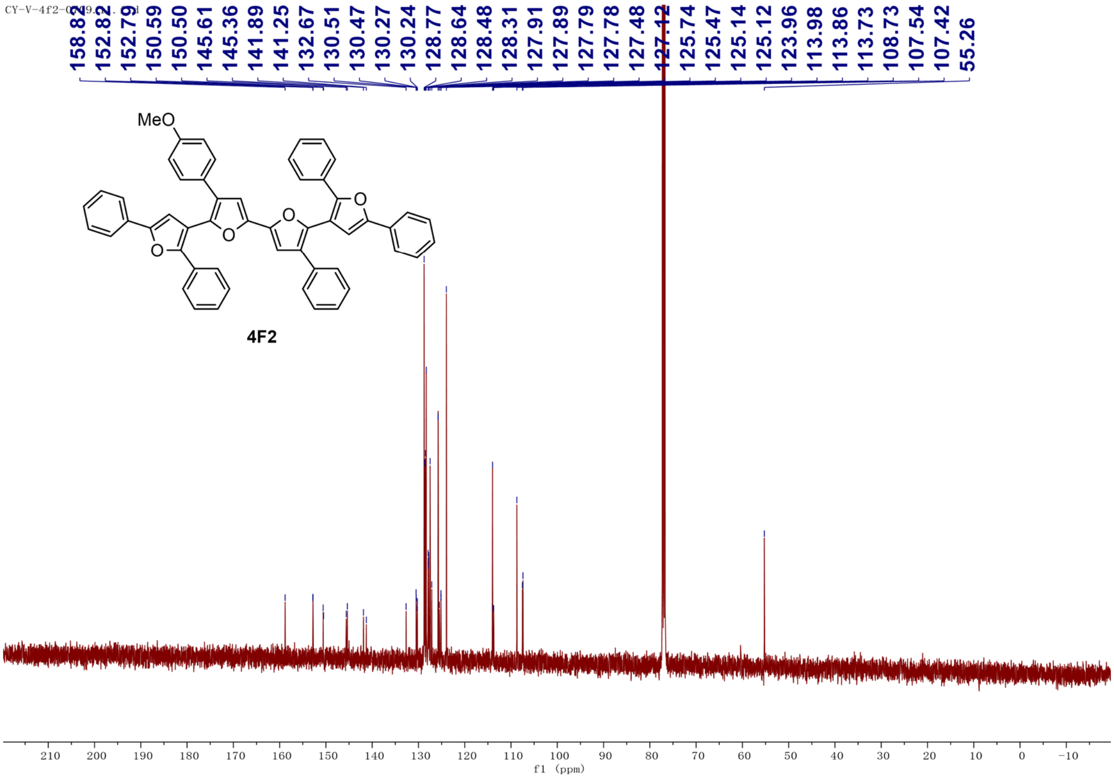

Supplementary Figure 248. ^{13}C NMR (101 MHz, $CDCl_3$) spectra for compound **4F2**

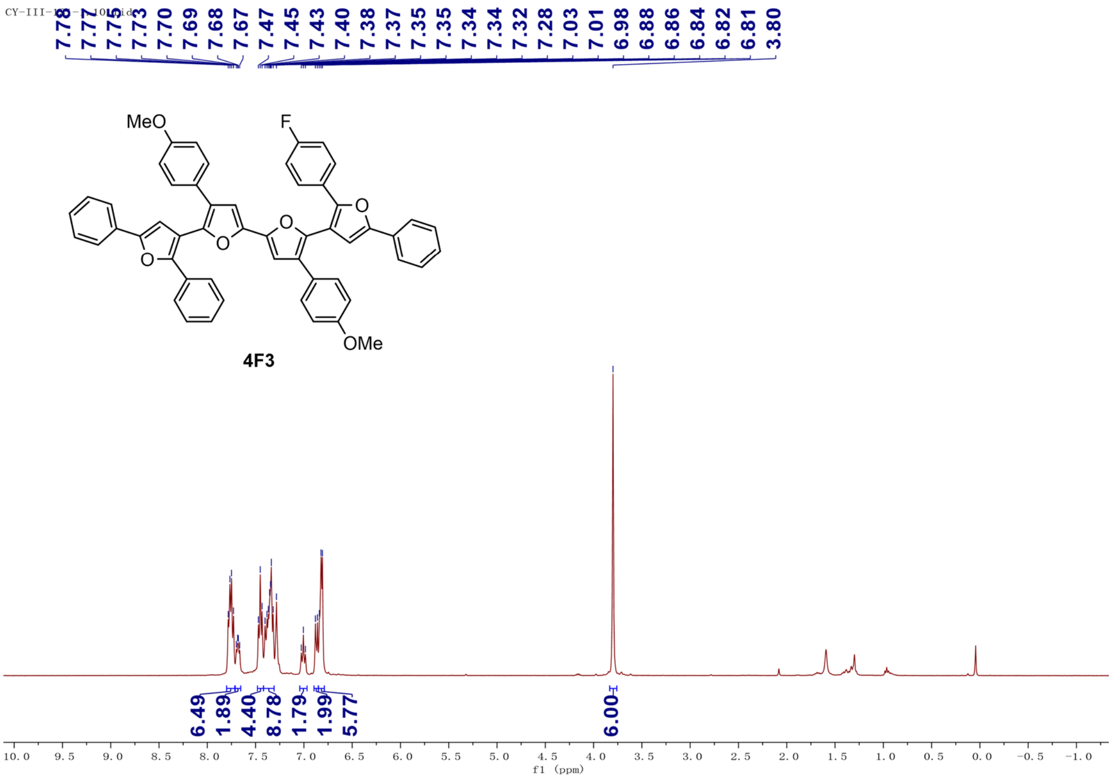

Supplementary Figure 249. ^{1}H NMR (400 MHz, $CDCl_3$) spectra for compound **4F3**

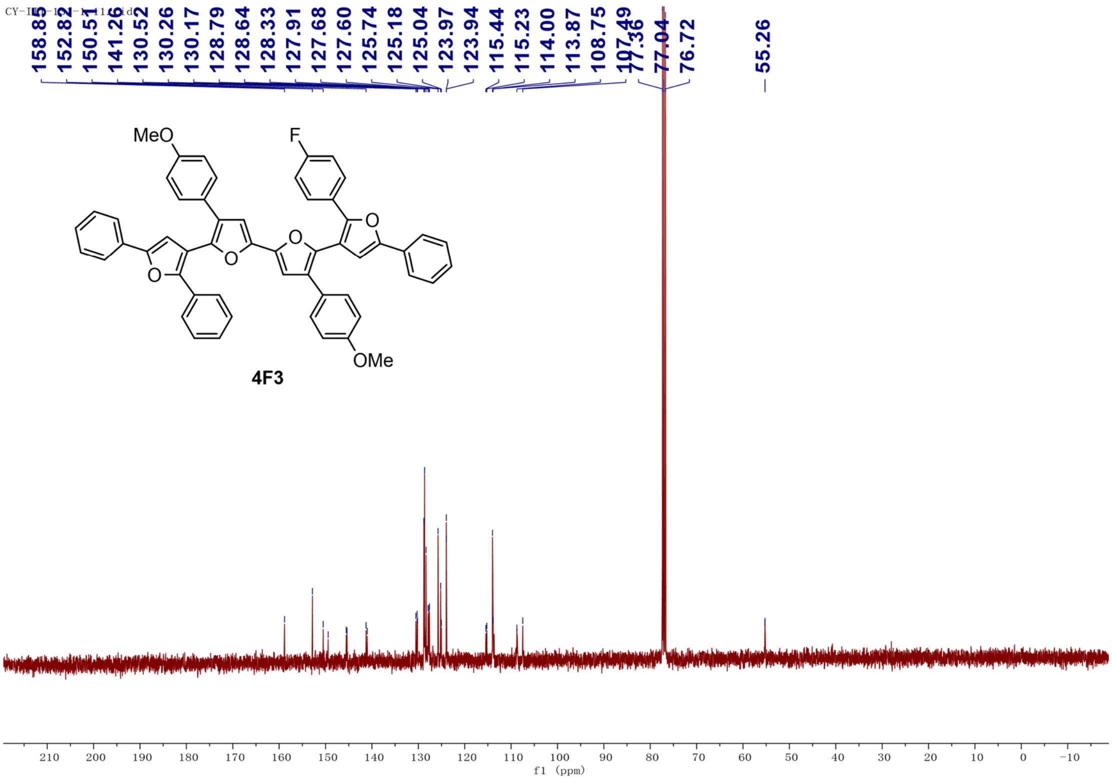

Supplementary Figure 250. ^{13}C NMR (101 MHz, $CDCl_3$) spectra for compound **4F3**

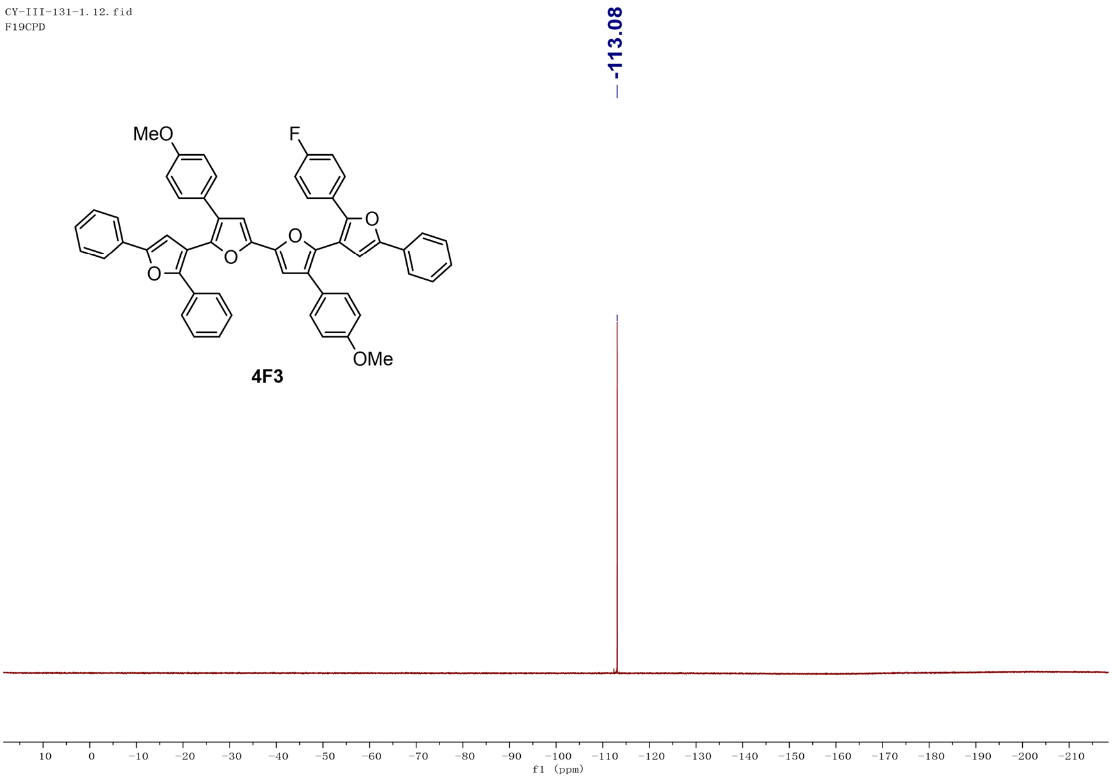

Supplementary Figure 251. ^{19}F NMR (376 MHz, $CDCl_3$) spectra for compound **4F3**

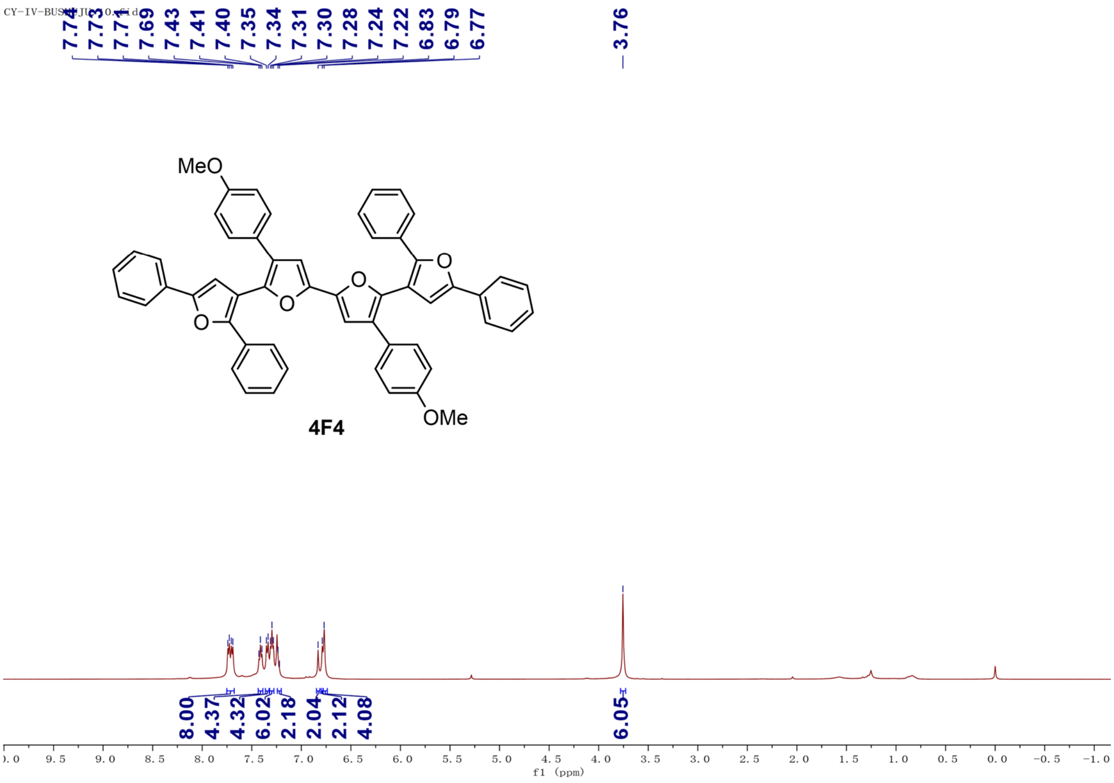

Supplementary Figure 252. ^{1}H NMR (500 MHz, $CDCl_3$) spectra for compound **4F4**

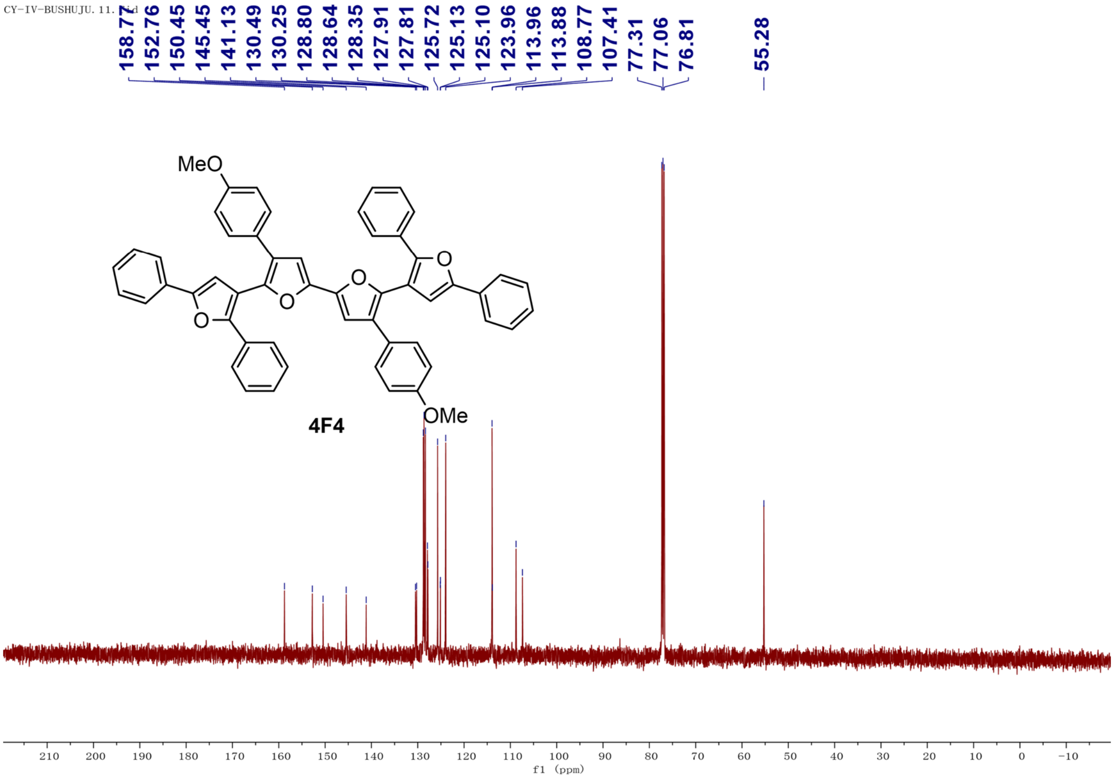

Supplementary Figure 253. ^{13}C NMR (126 MHz, $CDCl_3$) spectra for compound **4F4**

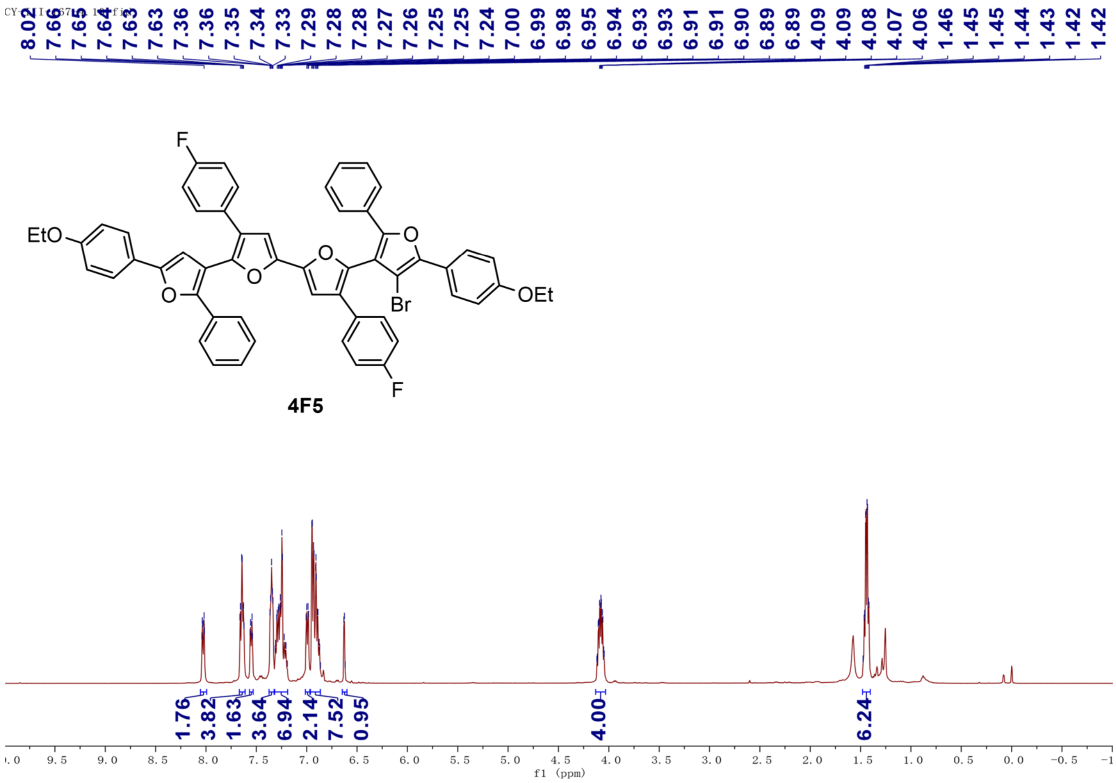

Supplementary Figure 254. ^{1}H NMR (500 MHz, $CDCl_3$) spectra for compound **4F5**

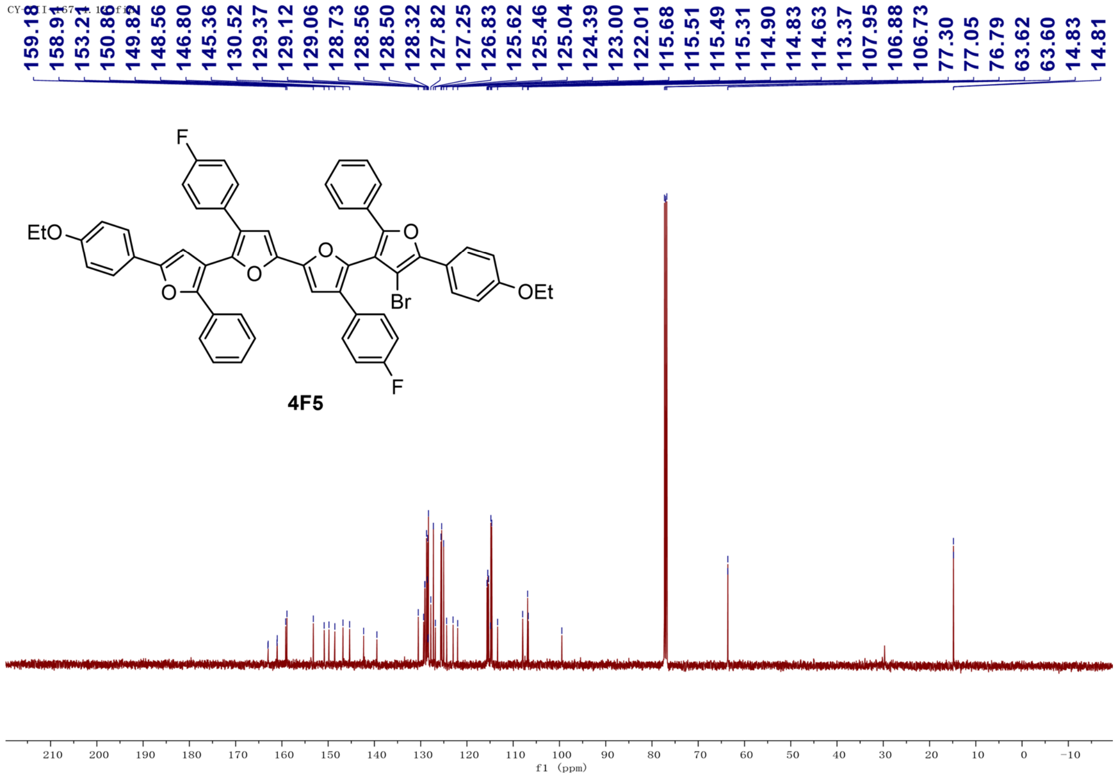

Supplementary Figure 255. ^{13}C NMR (126 MHz, $CDCl_3$) spectra for compound **4F5**

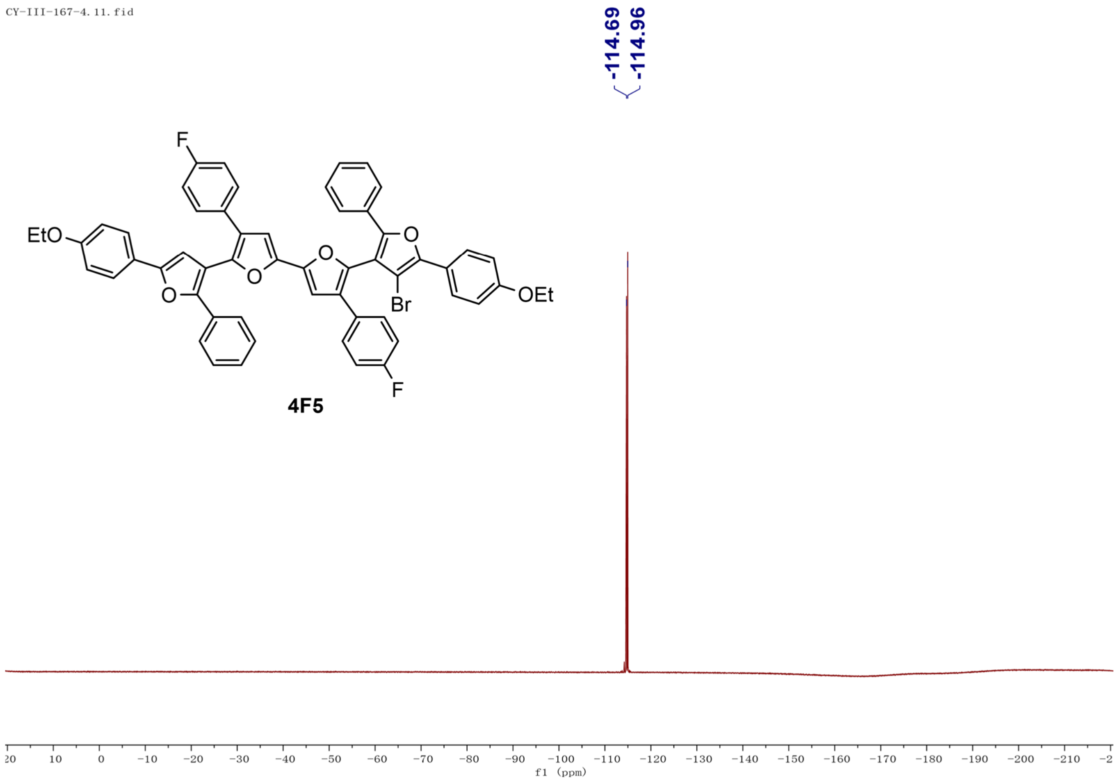

Supplementary Figure 256. ^{19}F NMR (471 MHz, $CDCl_3$) spectra for compound **4F5**

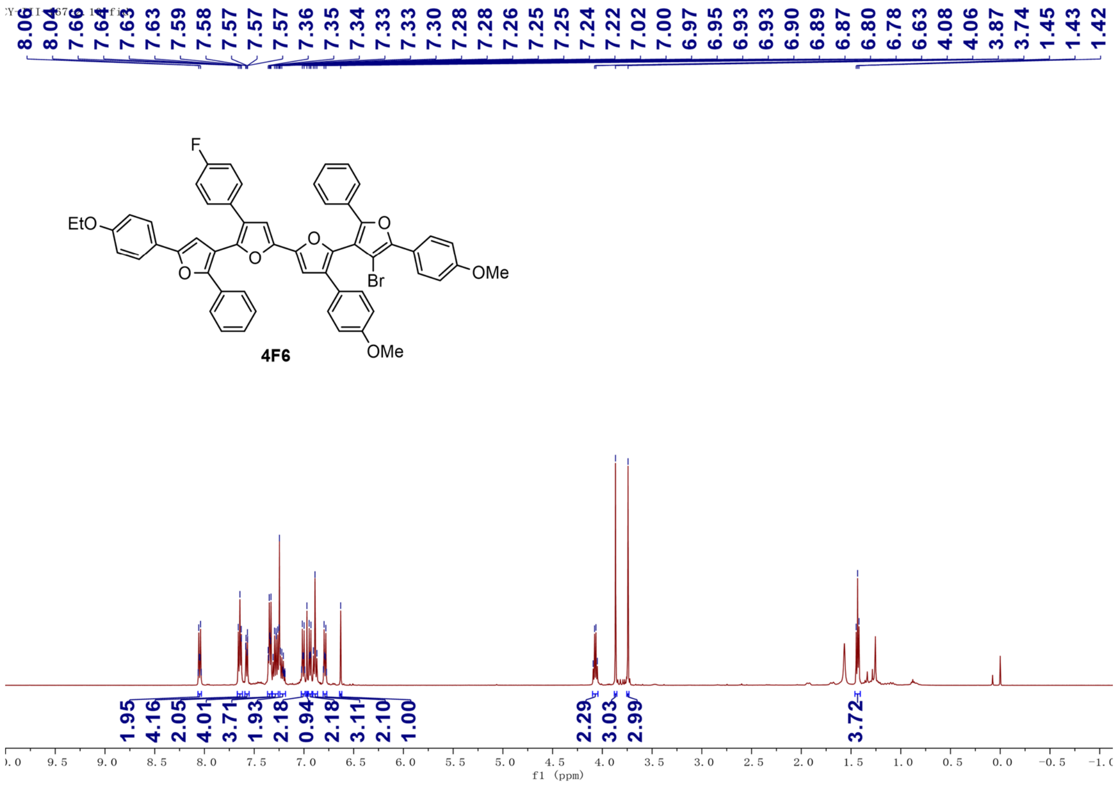

Supplementary Figure 257. ^{1}H NMR (500 MHz, $CDCl_3$) spectra for compound **4F6**

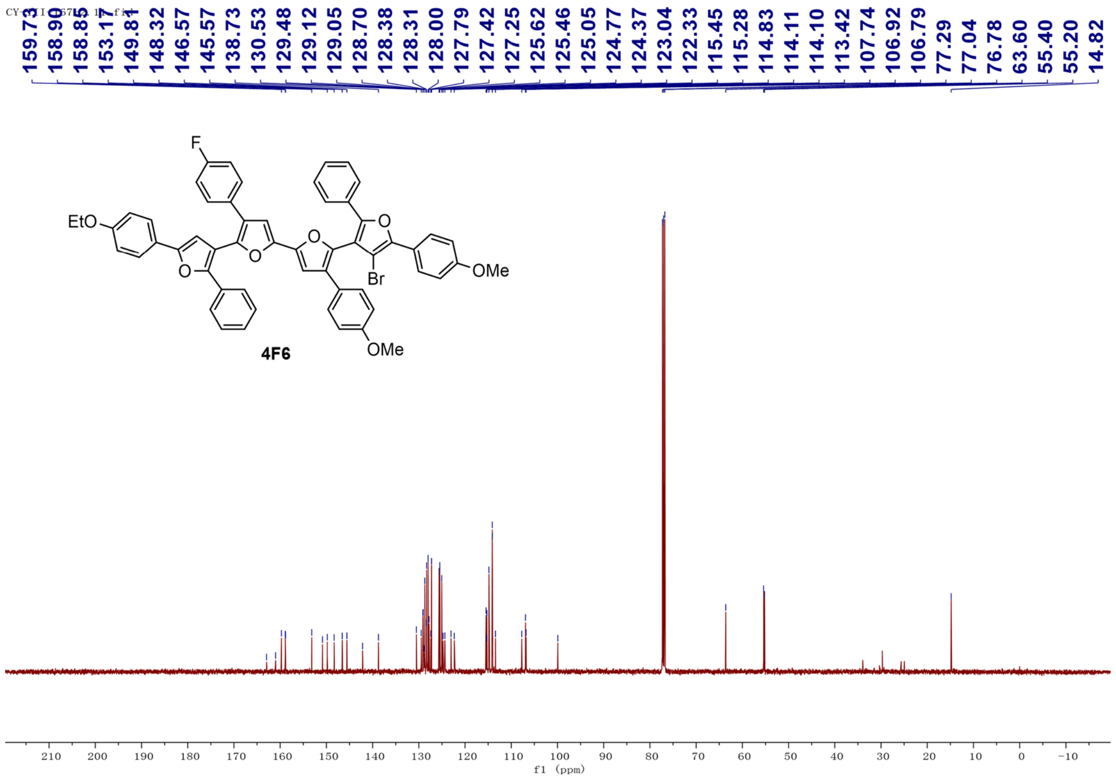

Supplementary Figure 258. ^{13}C NMR (126 MHz, $CDCl_3$) spectra for compound **4F6**

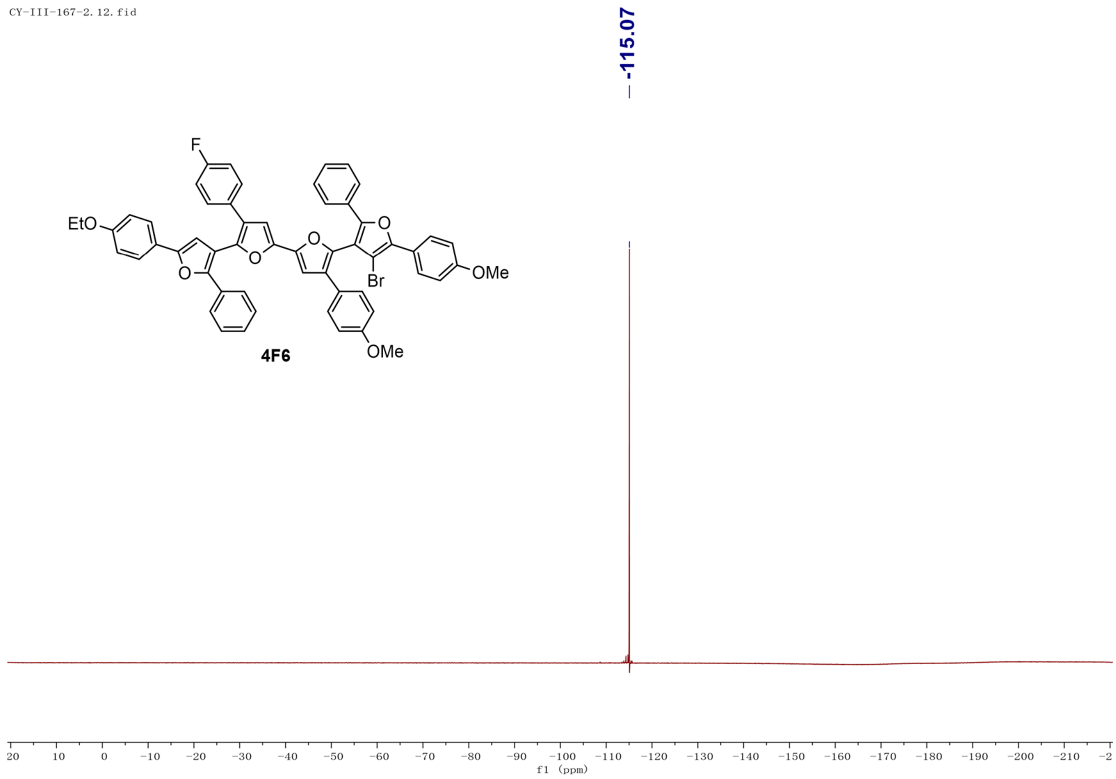

Supplementary Figure 259. ^{19}F NMR (471 MHz, $CDCl_3$) spectra for compound **4F6**

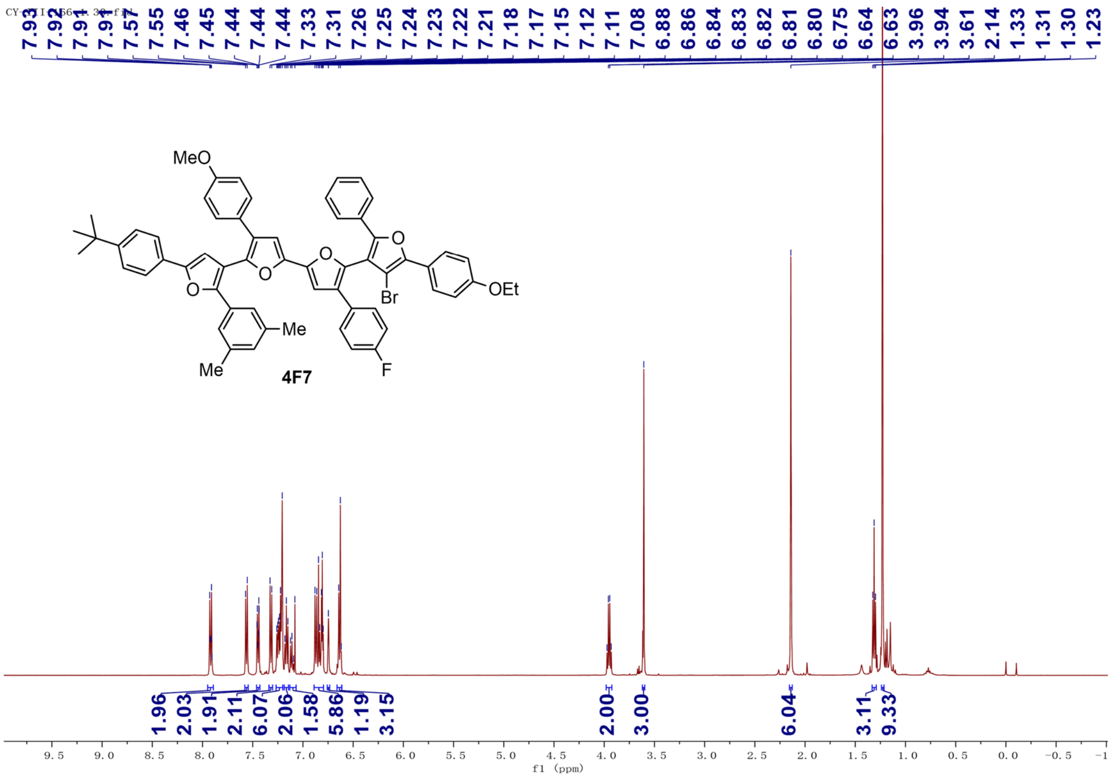

Supplementary Figure 260. ^{1}H NMR (500 MHz, $CDCl_3$) spectra for compound **4F7**

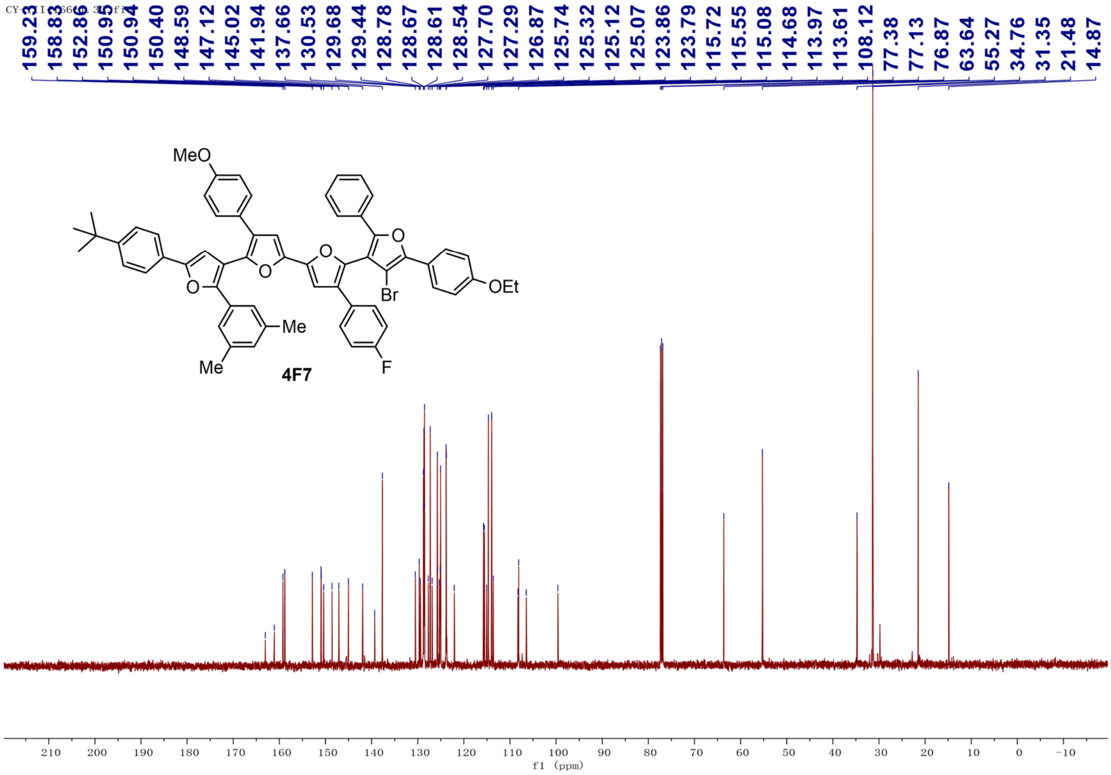

Supplementary Figure 261. ^{13}C NMR (126 MHz, $CDCl_3$) spectra for compound **4F7**

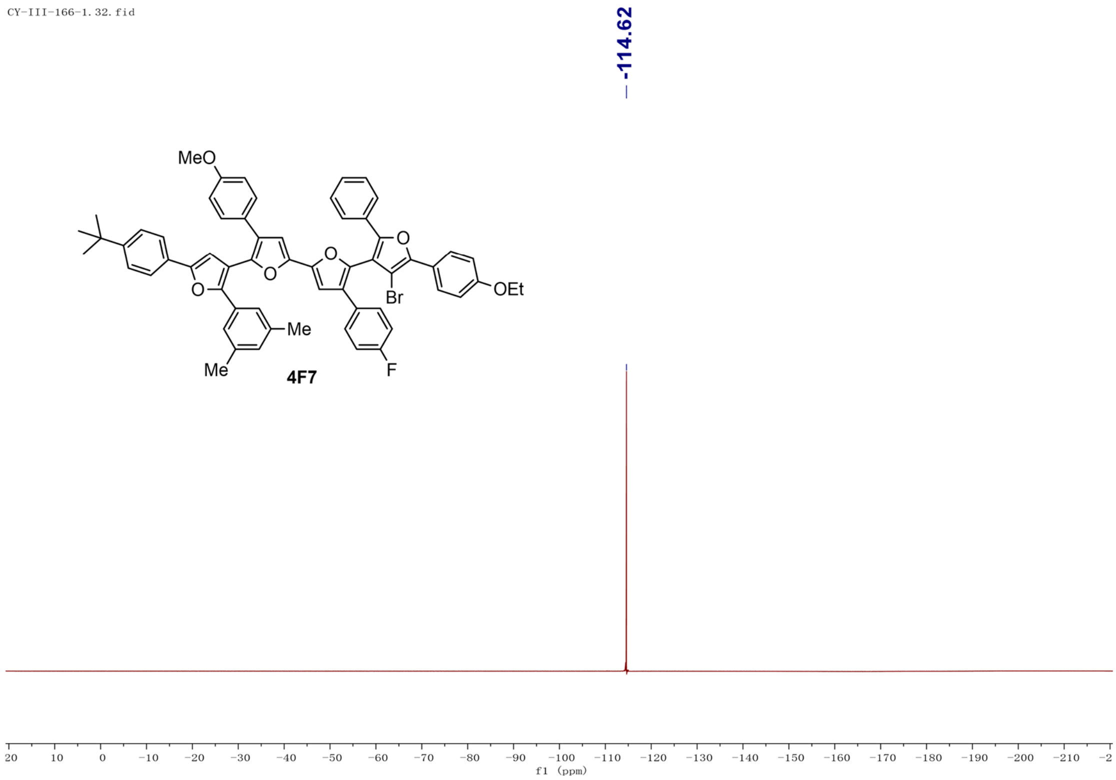

Supplementary Figure 262. ^{19}F NMR (471 MHz, $CDCl_3$) spectra for compound **4F7**

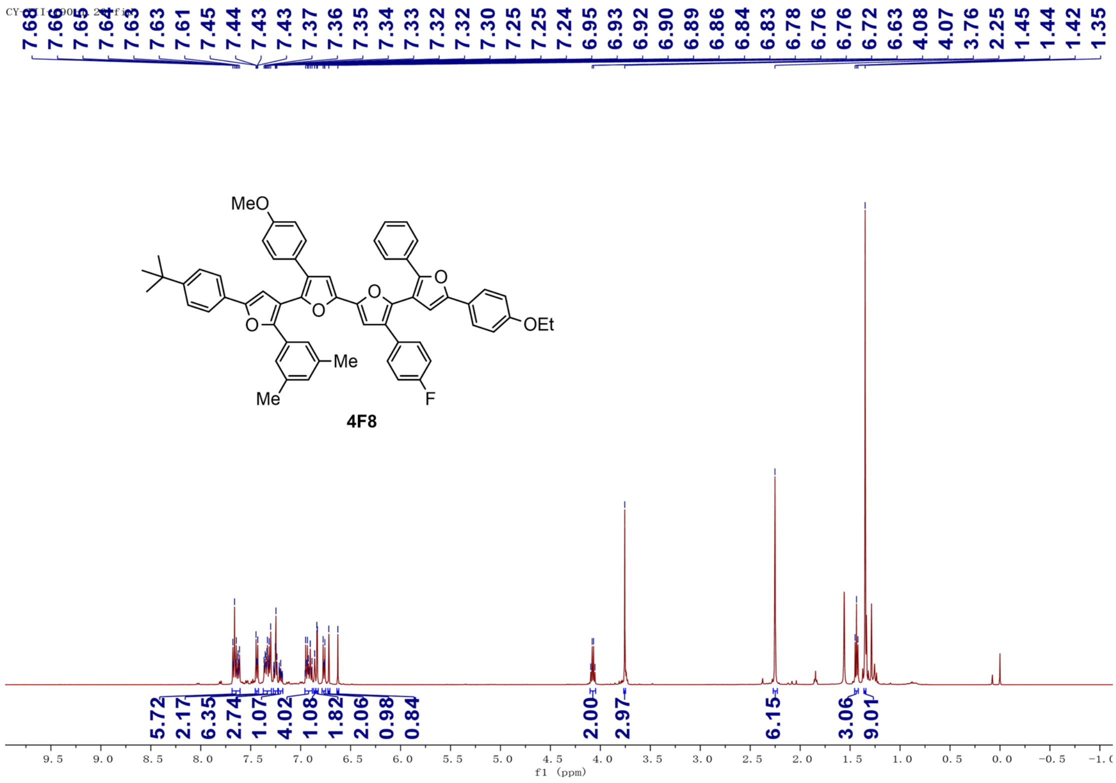

Supplementary Figure 263. ^{1}H NMR (500 MHz, $CDCl_3$) spectra for compound **4F8**

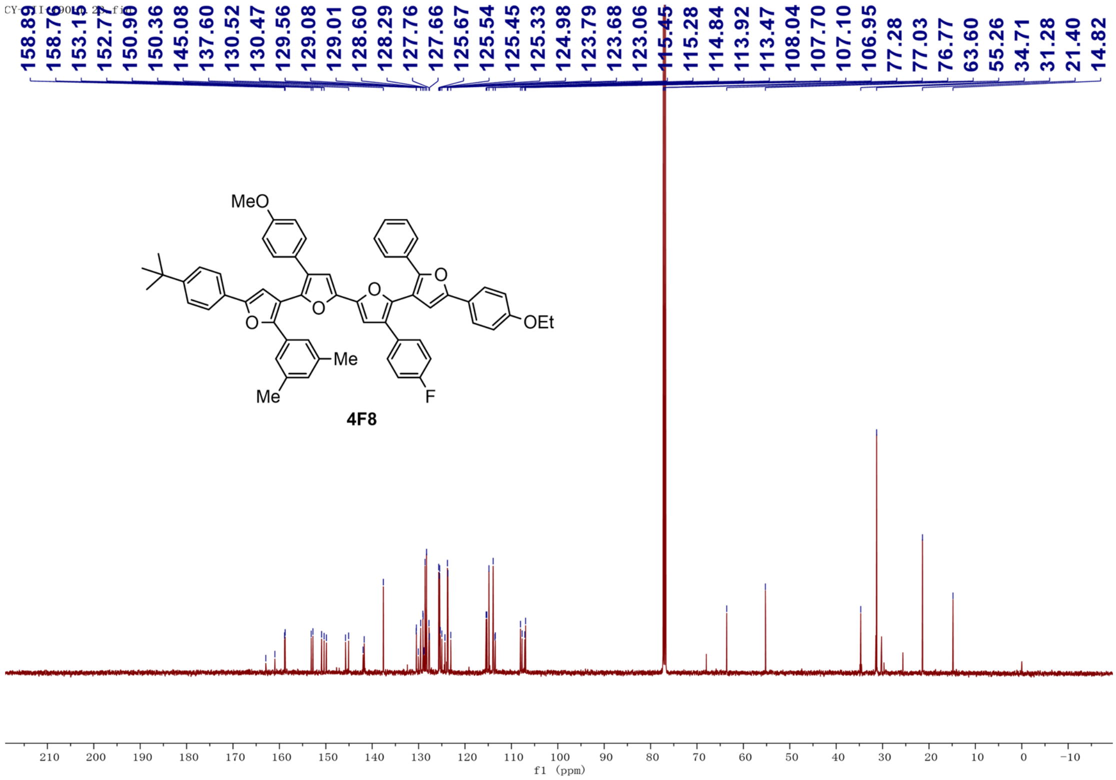

Supplementary Figure 264. ^{13}C NMR (126 MHz, $CDCl_3$) spectra for compound **4F8**

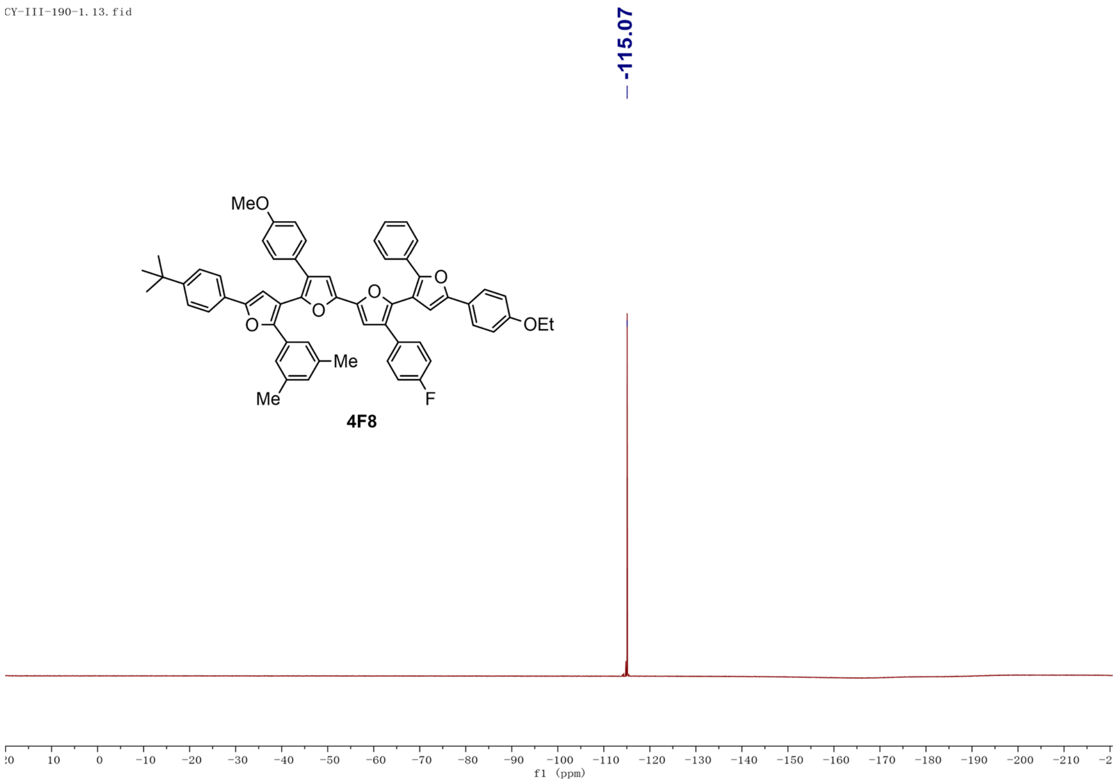

Supplementary Figure 265. ^{19}F NMR (471 MHz, $CDCl_3$) spectra for compound **4F8**

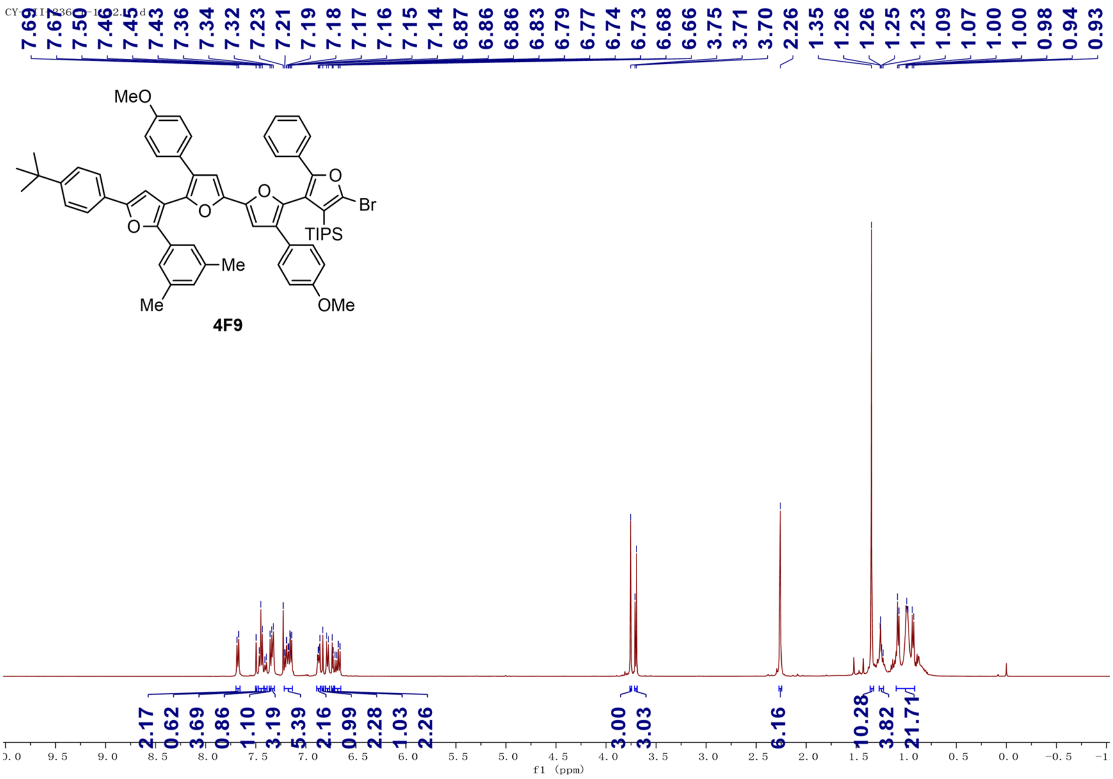

Supplementary Figure 266. ^{1}H NMR (500 MHz, $CDCl_3$) spectra for compound **4F9**

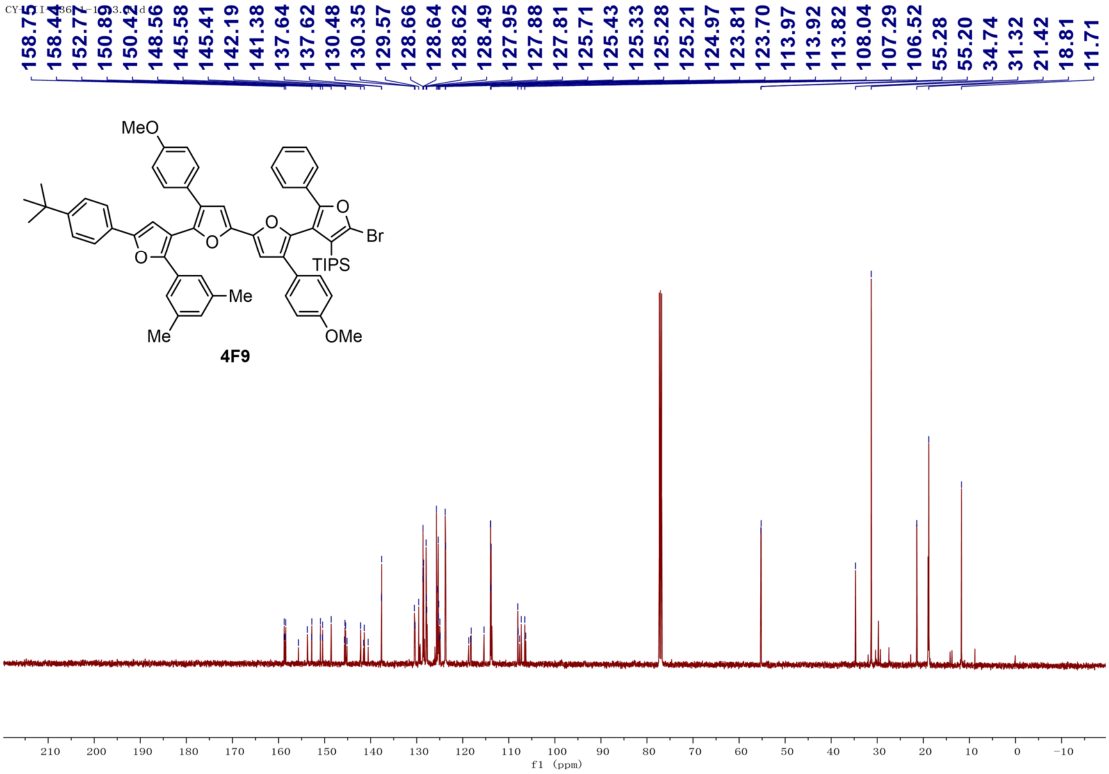

Supplementary Figure 267. ^{13}C NMR (126 MHz, $CDCl_3$) spectra for compound **4F9**

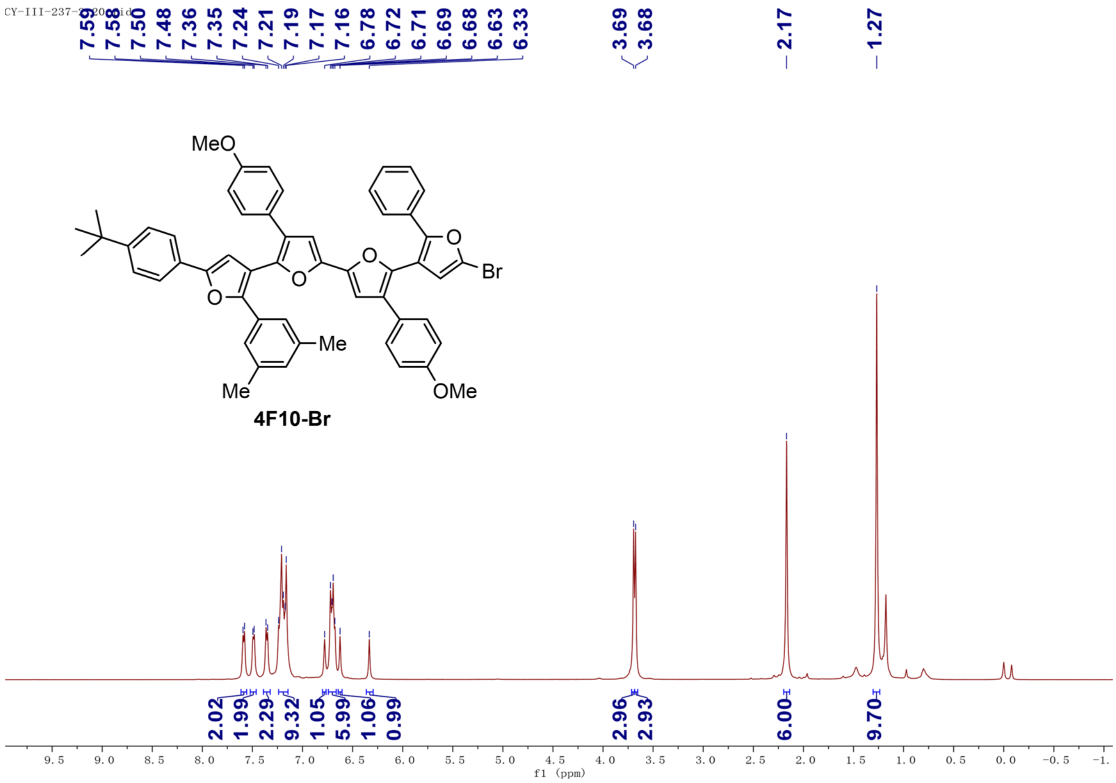

Supplementary Figure 268. ^{1}H NMR (500 MHz, $CDCl_3$) spectra for compound **4F10-Br**

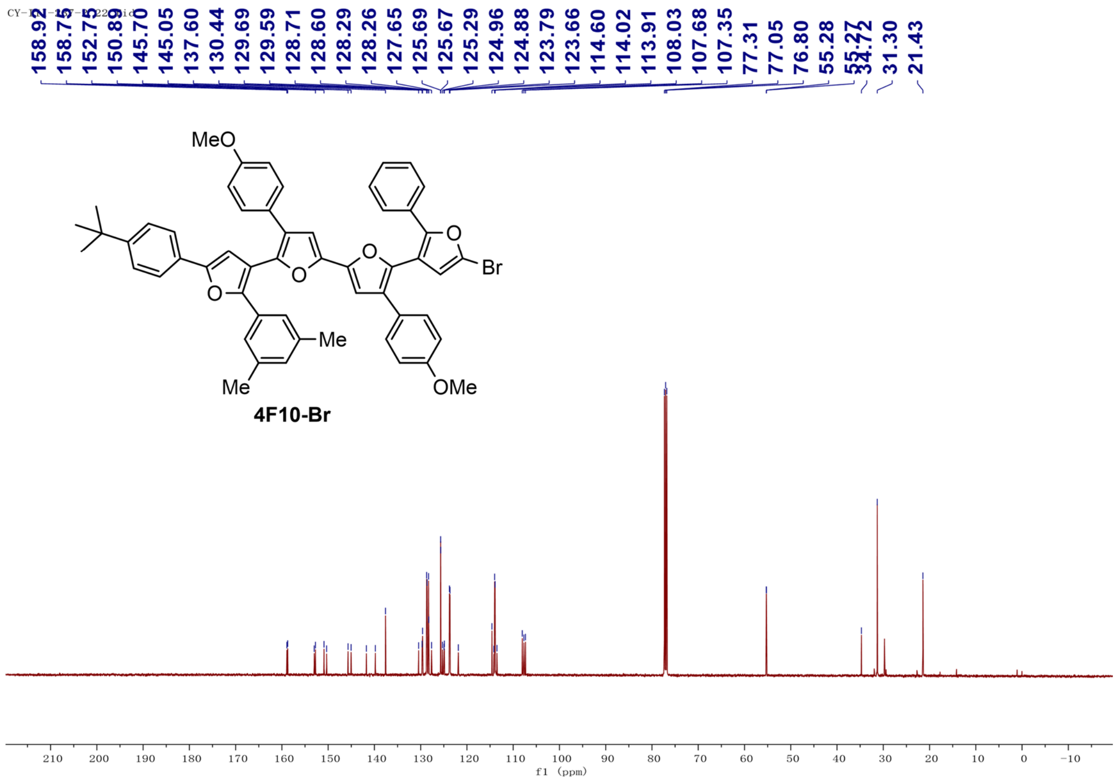

Supplementary Figure 269. ^{13}C NMR (126 MHz, $CDCl_3$) spectra for compound **4F10-Br**

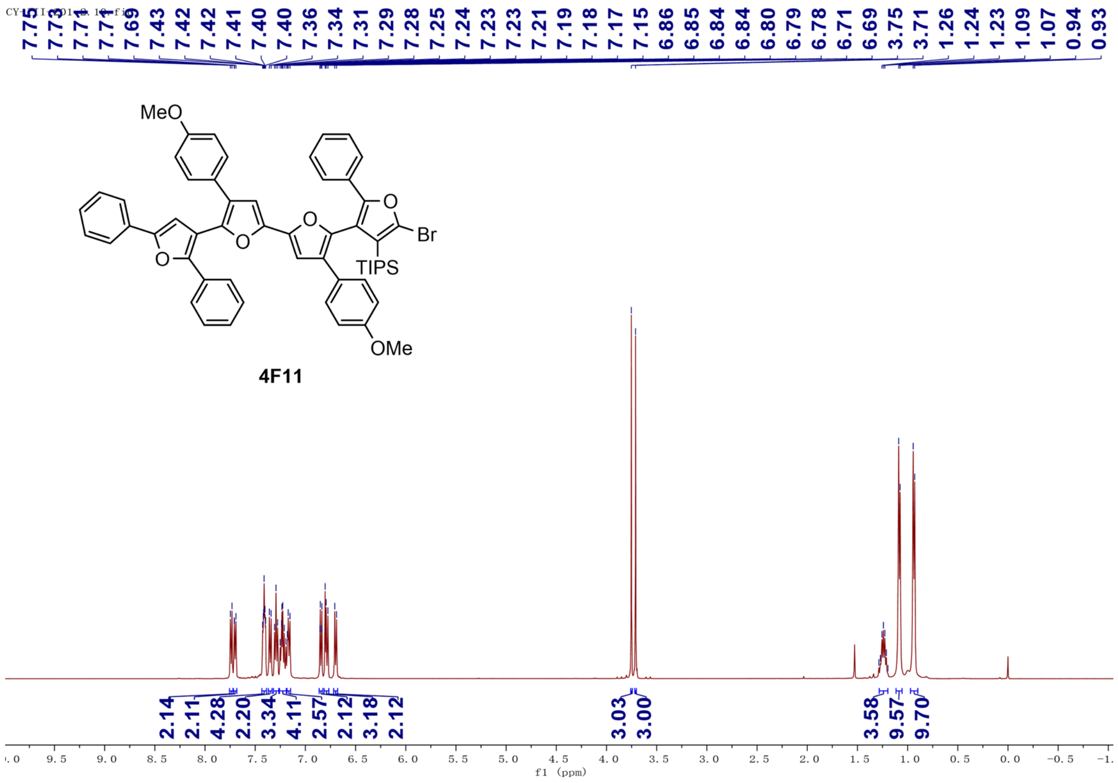

Supplementary Figure 270. ^{1}H NMR (500 MHz, $CDCl_3$) spectra for compound **4F11**

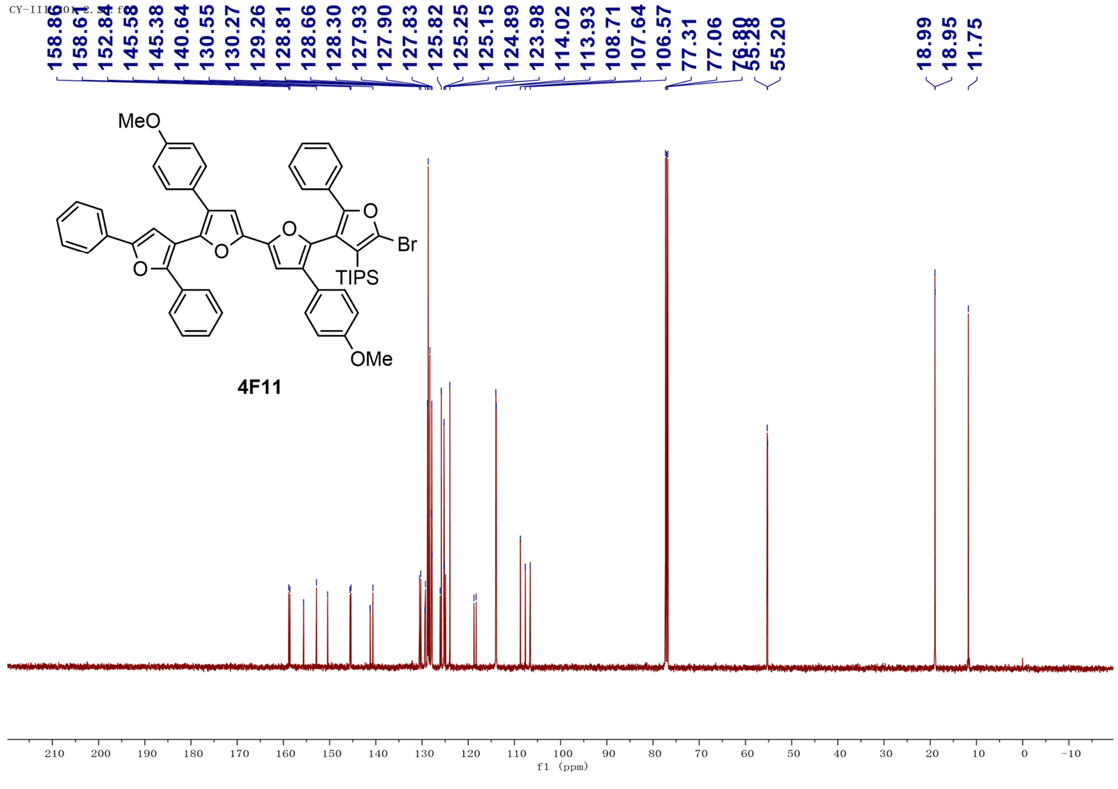

Supplementary Figure 271. ^{13}C NMR (126 MHz, $CDCl_3$) spectra for compound **4F11**

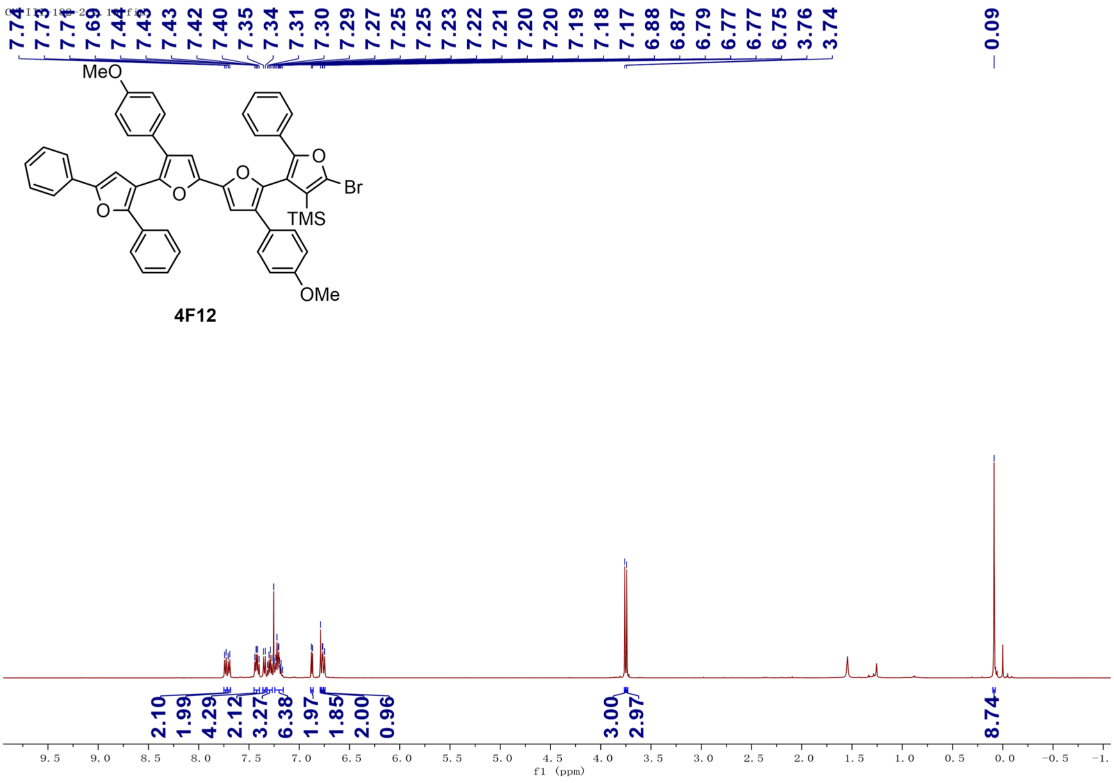

Supplementary Figure 272. ^{1}H NMR (500 MHz, $CDCl_3$) spectra for compound **4F12**

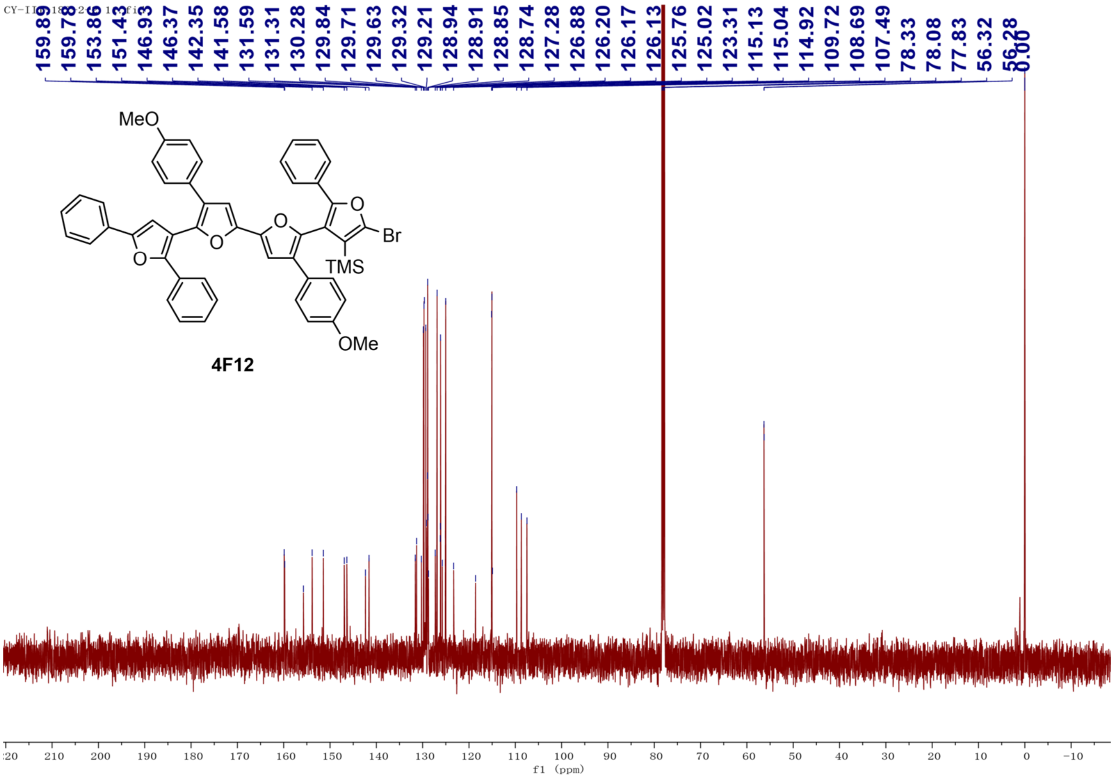

Supplementary Figure 273. ^{13}C NMR (126 MHz, $CDCl_3$) spectra for compound **4F12**

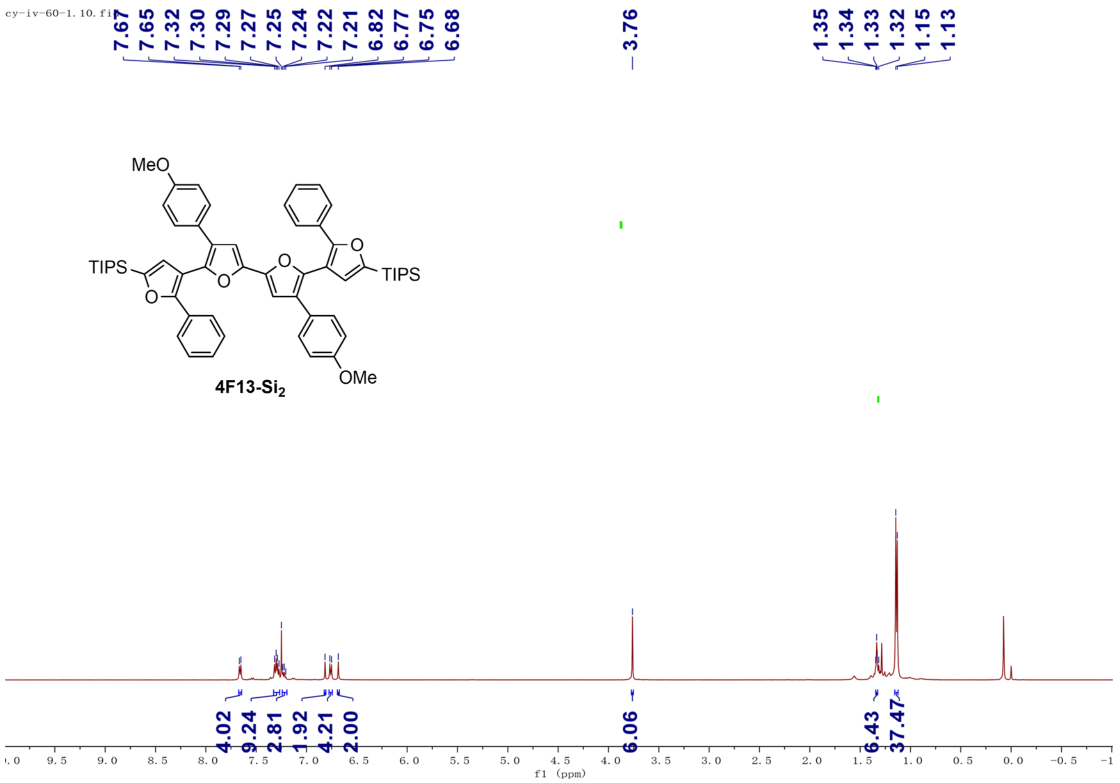

Supplementary Figure 274. ^{1}H NMR (500 MHz, $CDCl_3$) spectra for compound **4F13-Si$_2$**

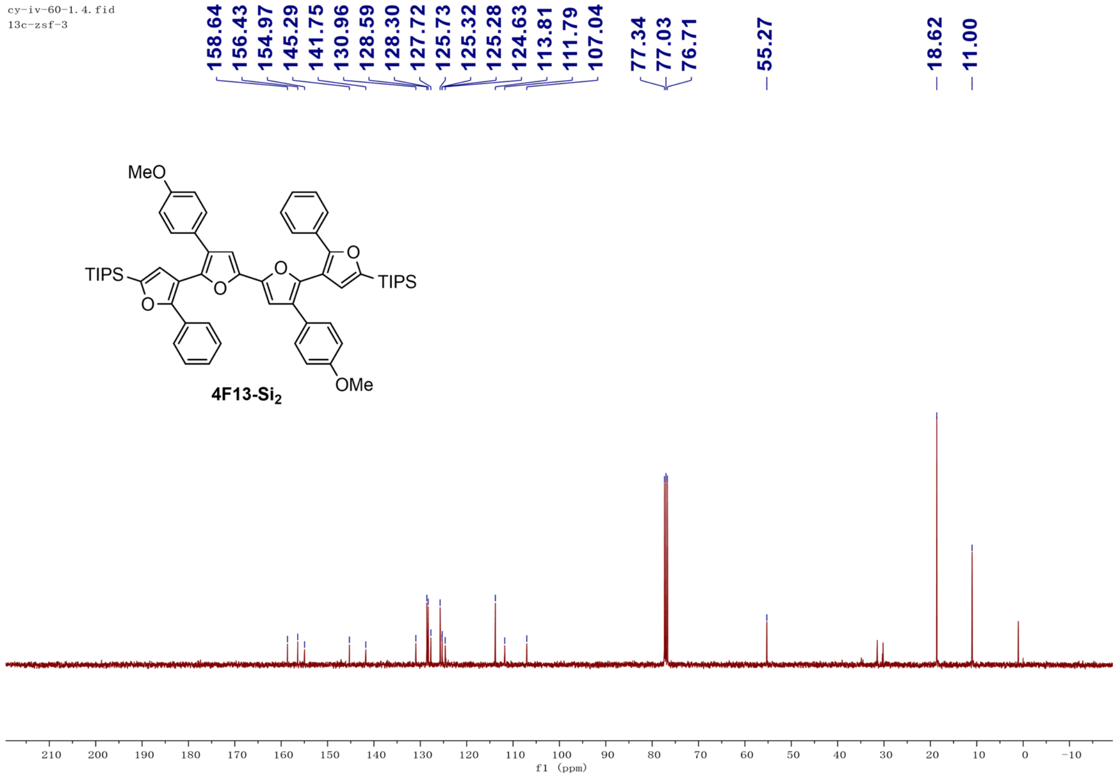

Supplementary Figure 275. ^{13}C NMR (126 MHz, $CDCl_3$) spectra for compound **4F13-Si$_2$**

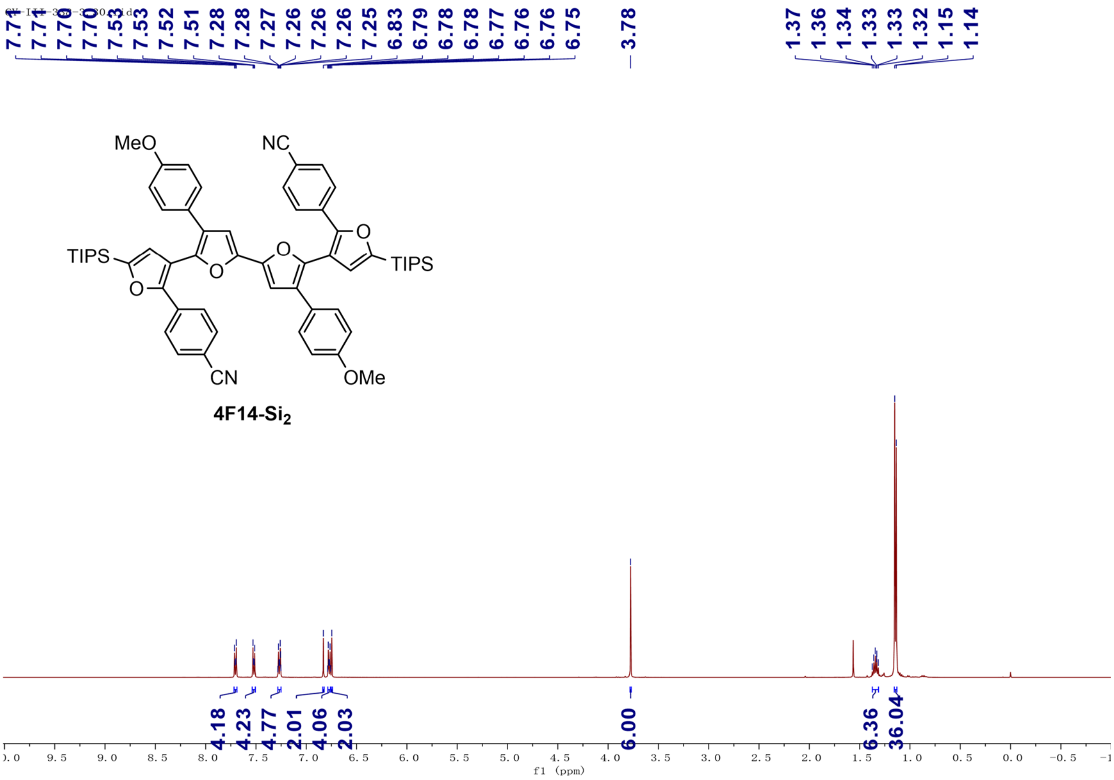

Supplementary Figure 276. ^{1}H NMR (500 MHz, $CDCl_3$) spectra for compound **4F14-Si$_2$**

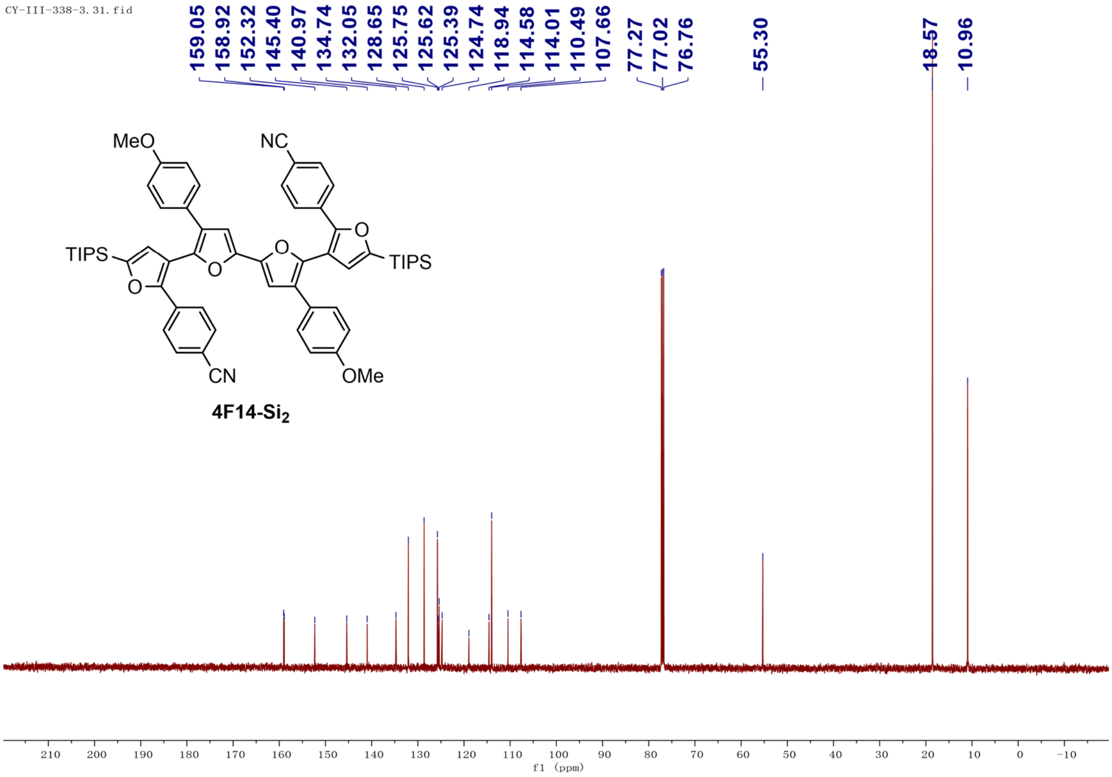

Supplementary Figure 277. ^{13}C NMR (126 MHz, $CDCl_3$) spectra for compound **4F14-Si$_2$**

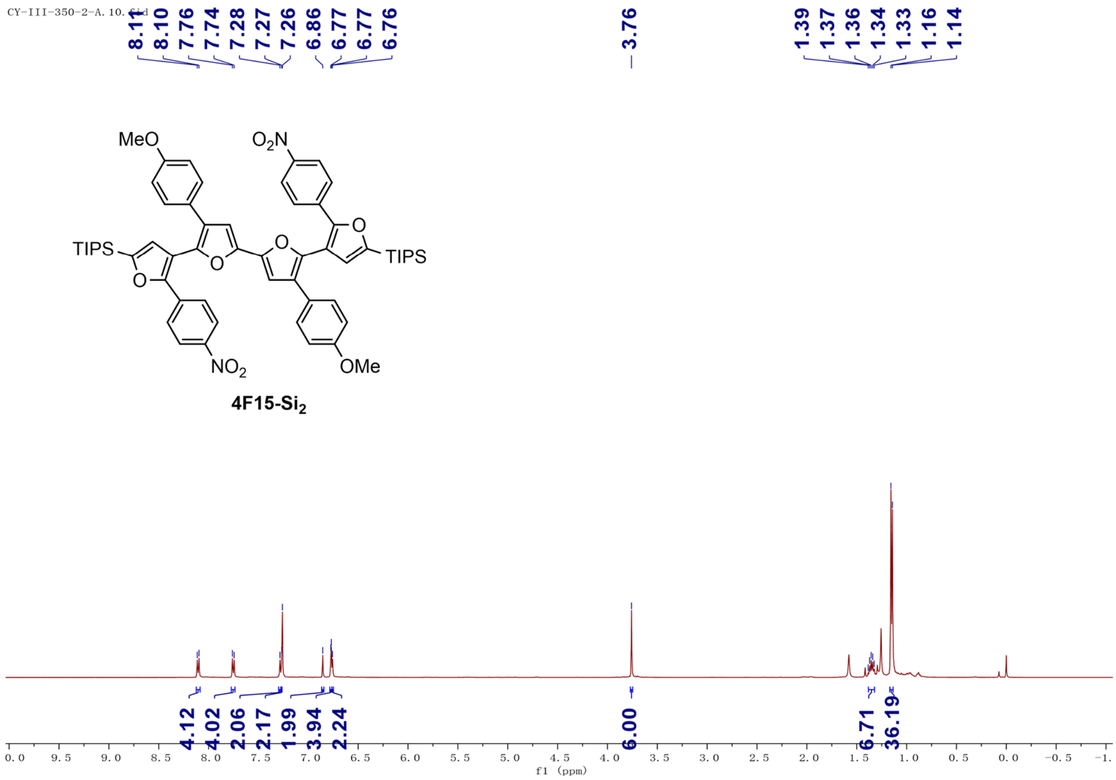

Supplementary Figure 278. ^{1}H NMR (500 MHz, CDCl$_3$) spectra for compound **4F15-Si$_2$**

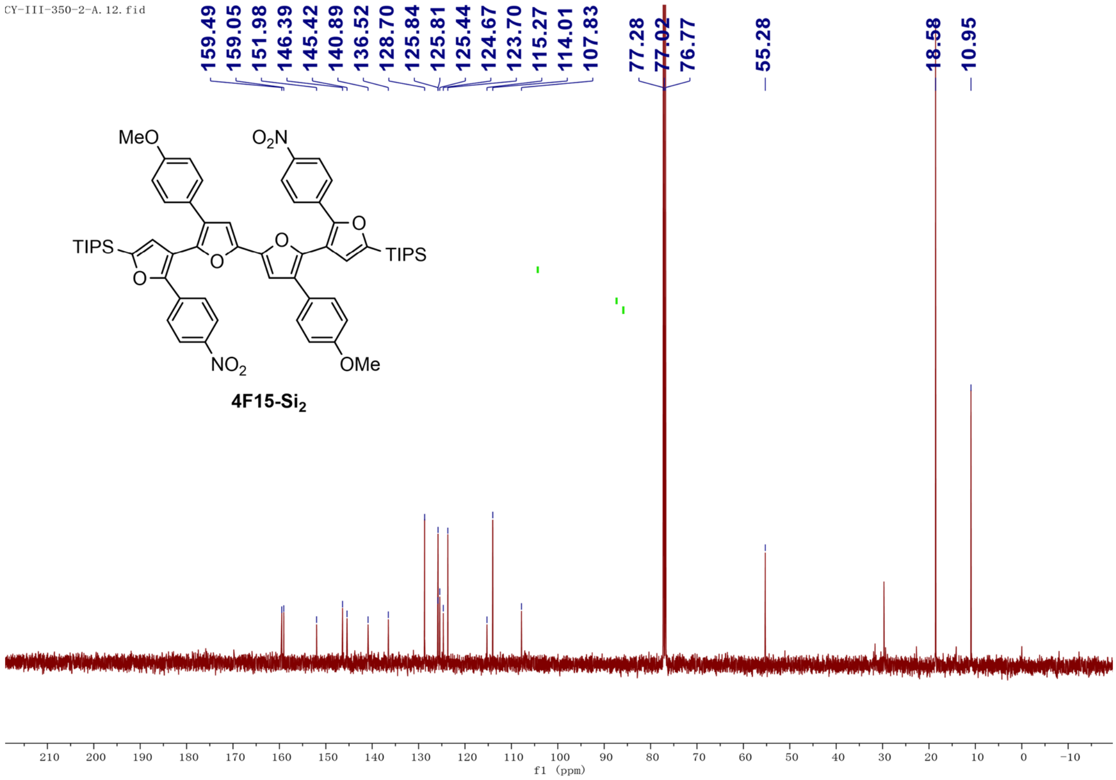

Supplementary Figure 279. ^{13}C NMR (126 MHz, CDCl$_3$) spectra for compound **4F15-Si$_2$**

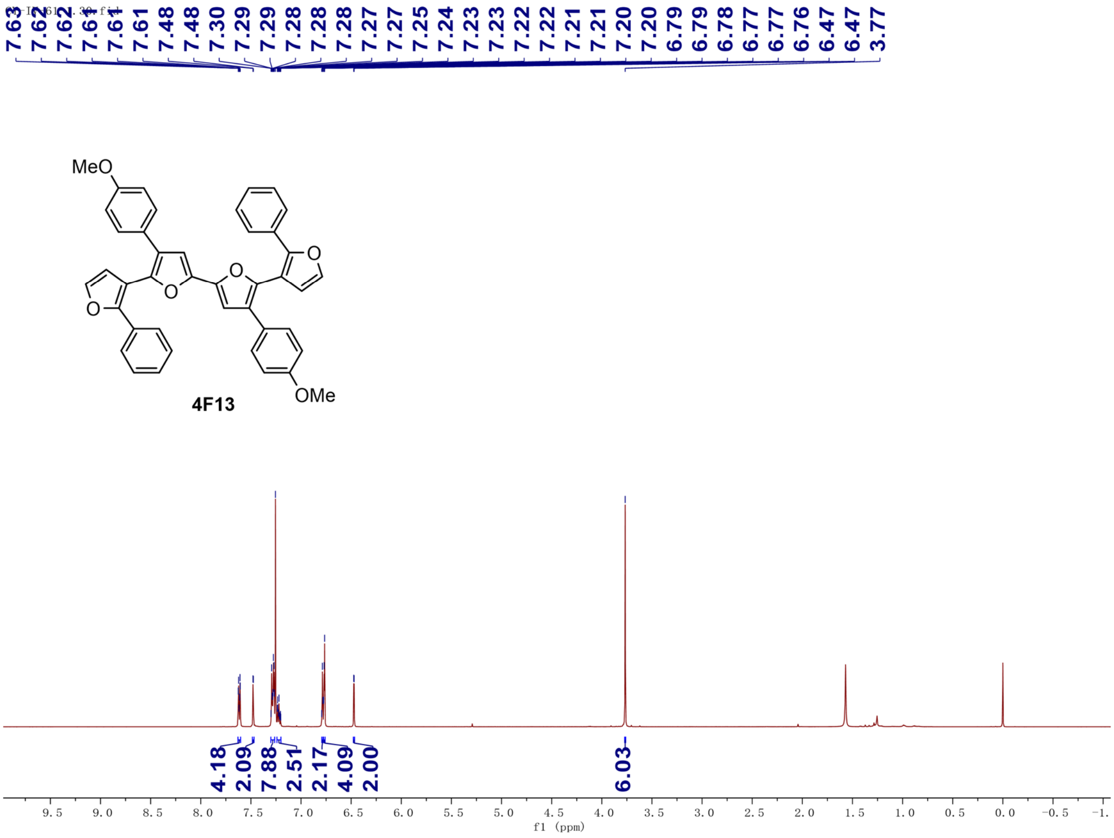

Supplementary Figure 280. ^{1}H NMR (500 MHz, $CDCl_3$) spectra for compound **4F13**

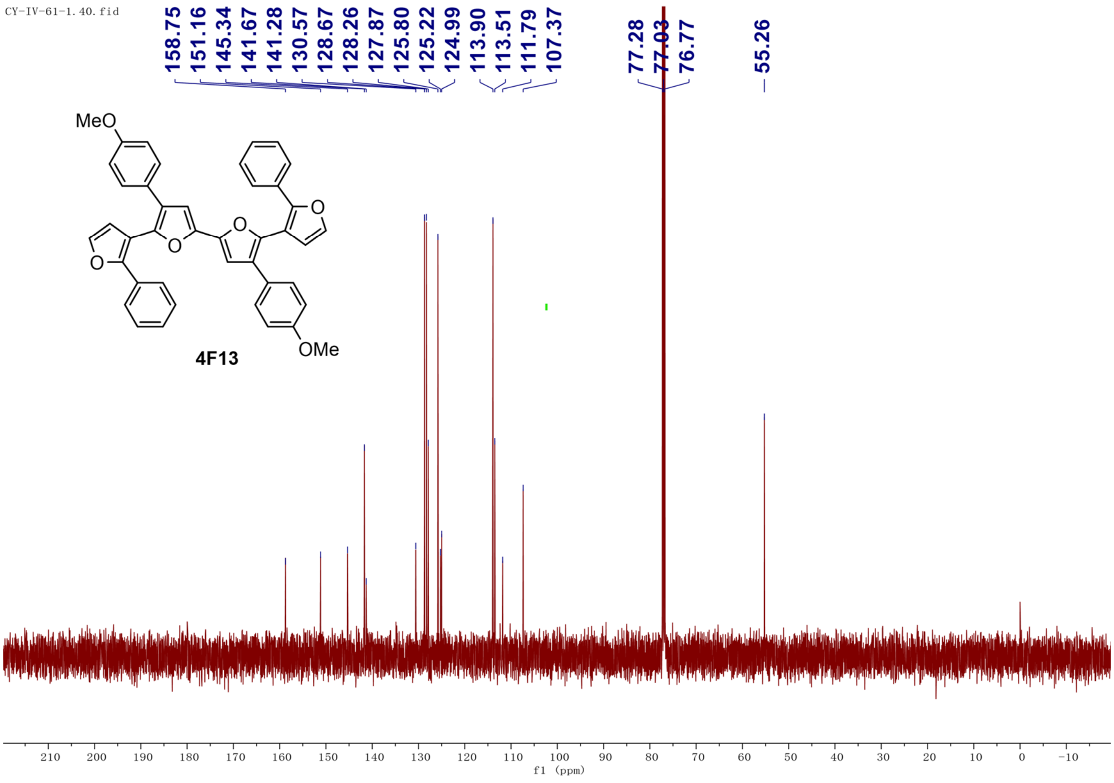

Supplementary Figure 281. ^{13}C NMR (126 MHz, $CDCl_3$) spectra for compound **4F13**

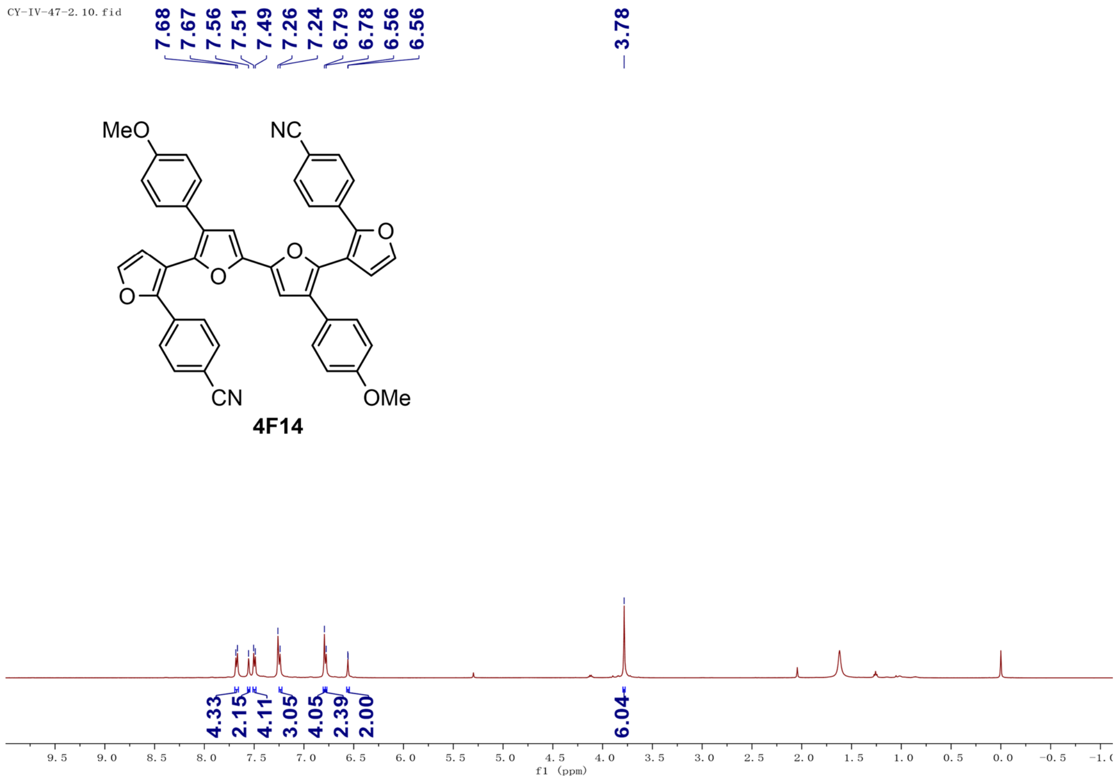

Supplementary Figure 282. ^{1}H NMR (500 MHz, $CDCl_3$) spectra for compound **4F14**

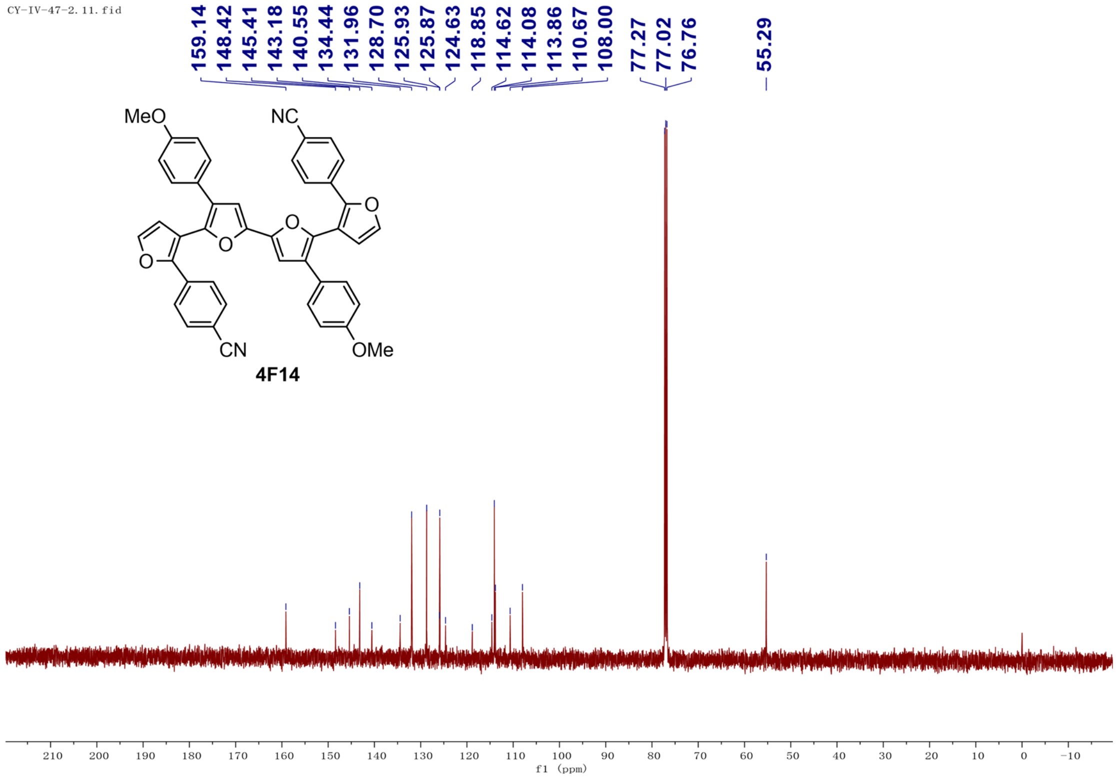

Supplementary Figure 283. ^{13}C NMR (126 MHz, $CDCl_3$) spectra for compound **4F14**

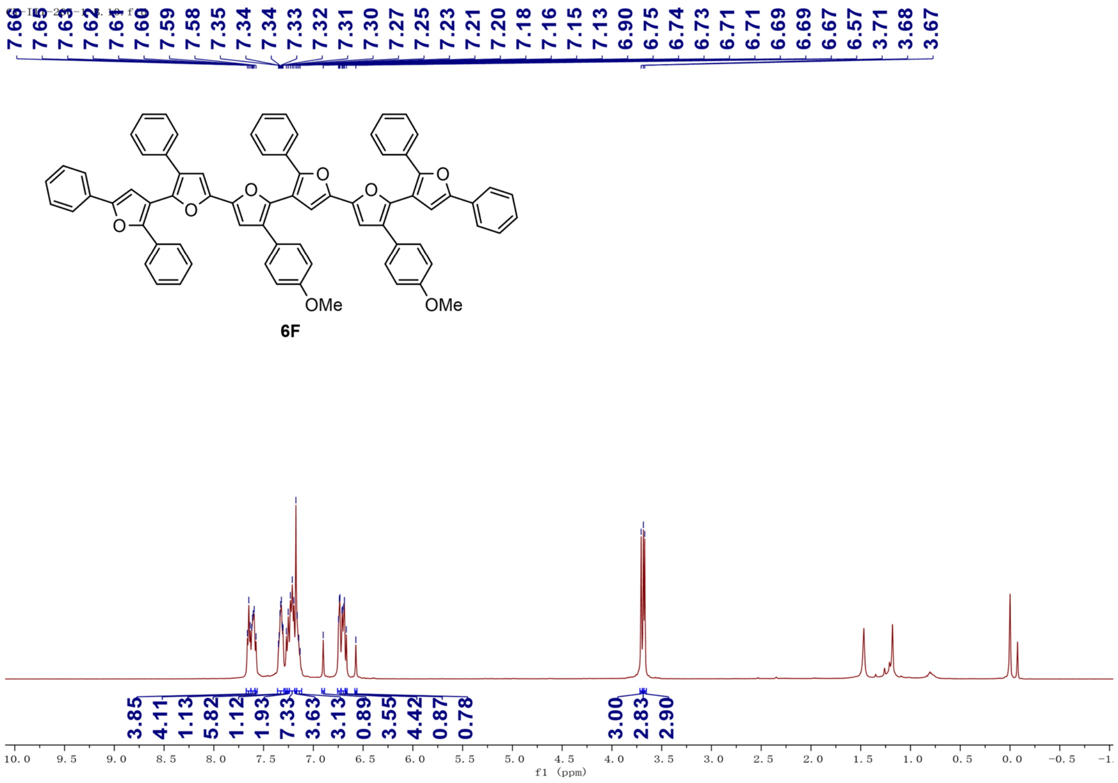

Supplementary Figure 284. ^{1}H NMR (500 MHz, $CDCl_3$) spectra for compound **6F**

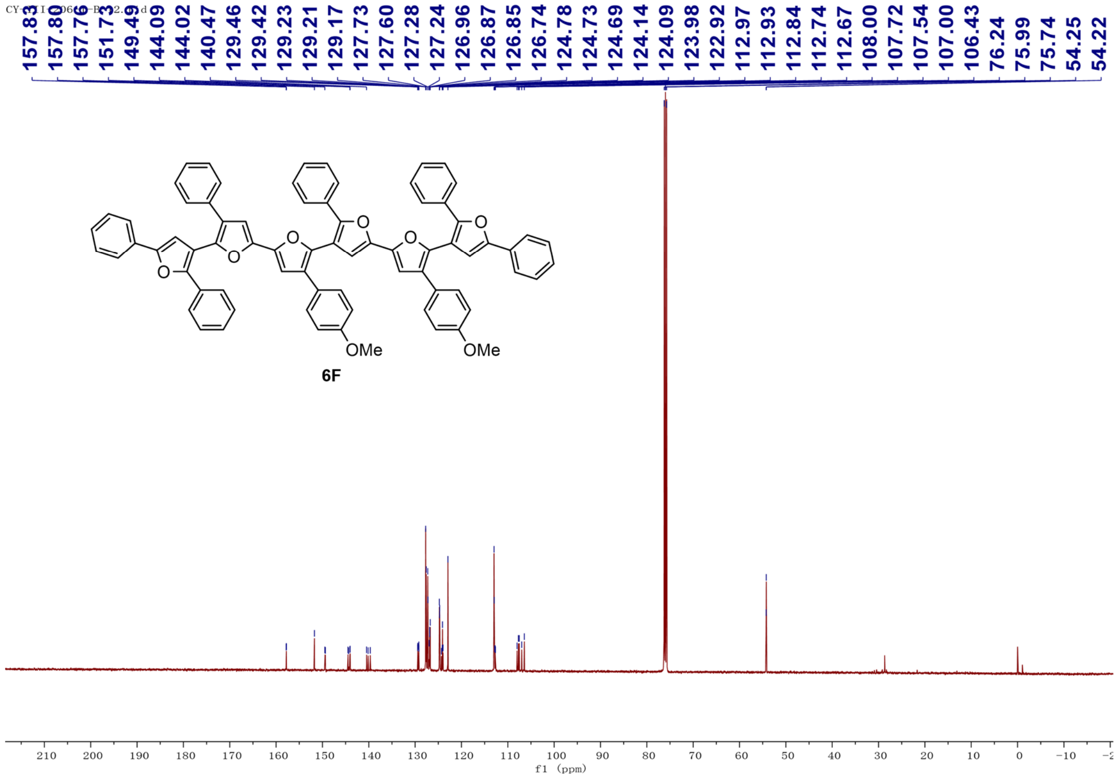

Supplementary Figure 285. ^{13}C NMR (126 MHz, $CDCl_3$) spectra for compound **6F**

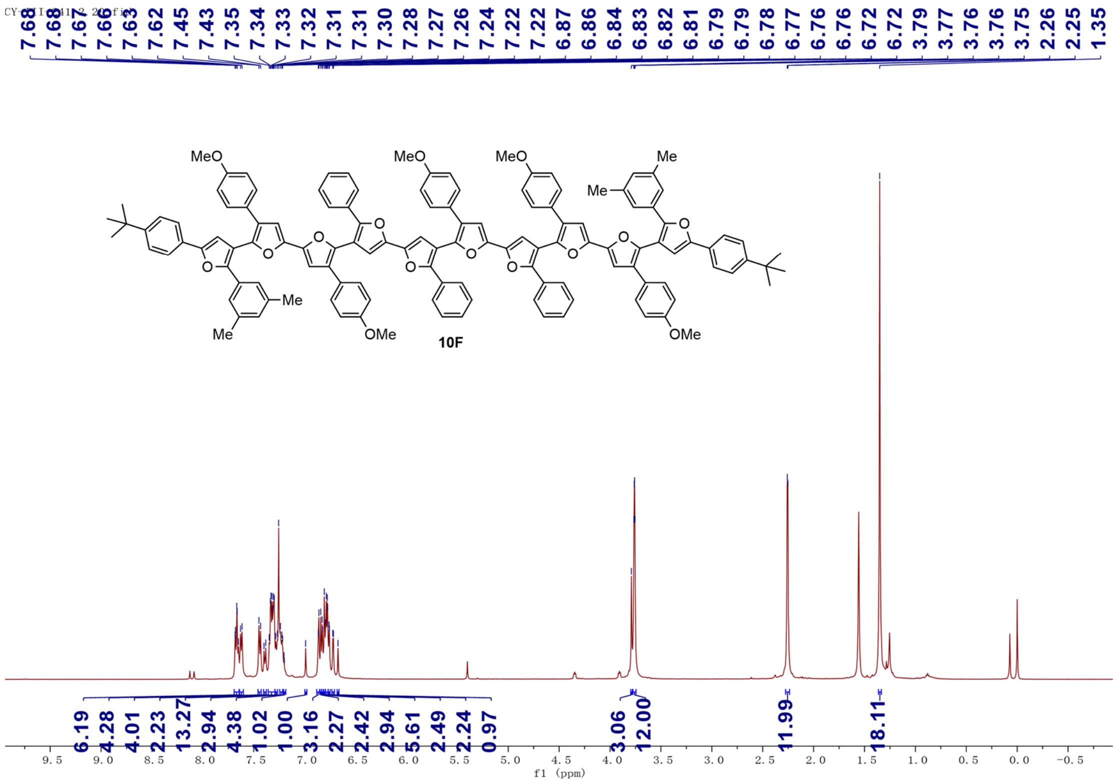

Supplementary Figure 286. ^{1}H NMR (500 MHz, $CDCl_3$) spectra for compound **10F**

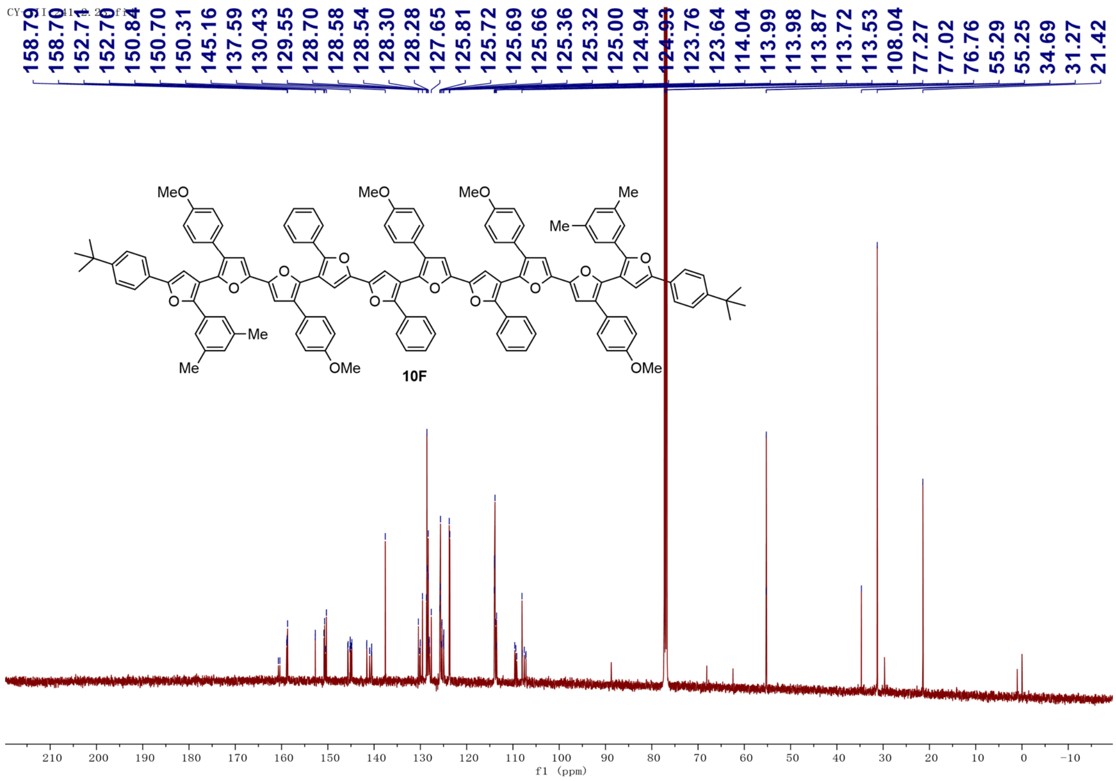

Supplementary Figure 287. ^{13}C NMR (126 MHz, $CDCl_3$) spectra for compound **10F**

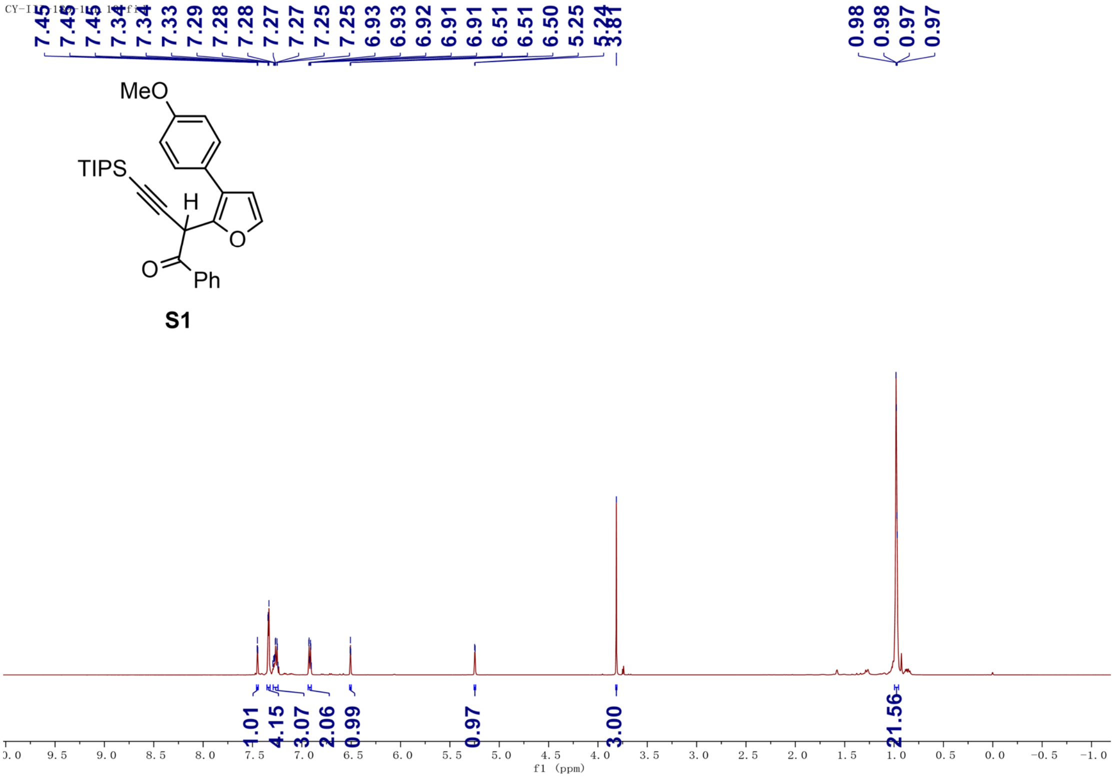

Supplementary Figure 288. ^{1}H NMR (500 MHz, $CDCl_3$) spectra for compound **S1**

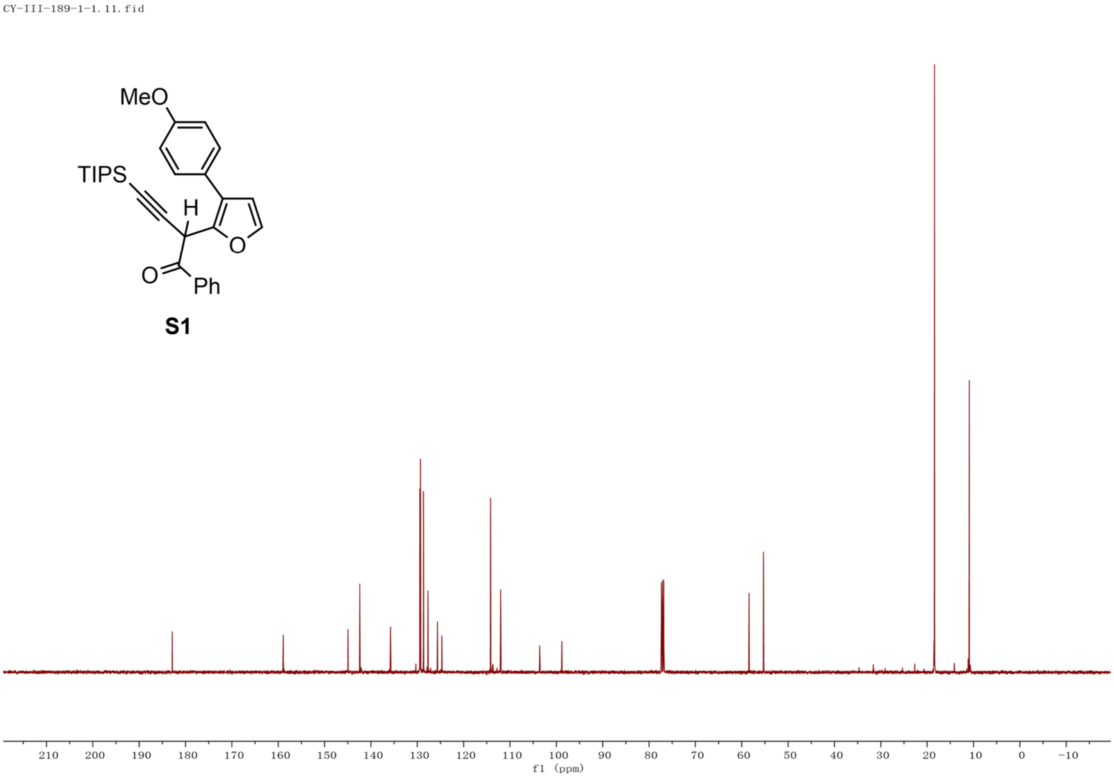

Supplementary Figure 289. ^{13}C NMR (126 MHz, $CDCl_3$) spectra for compound **S1**

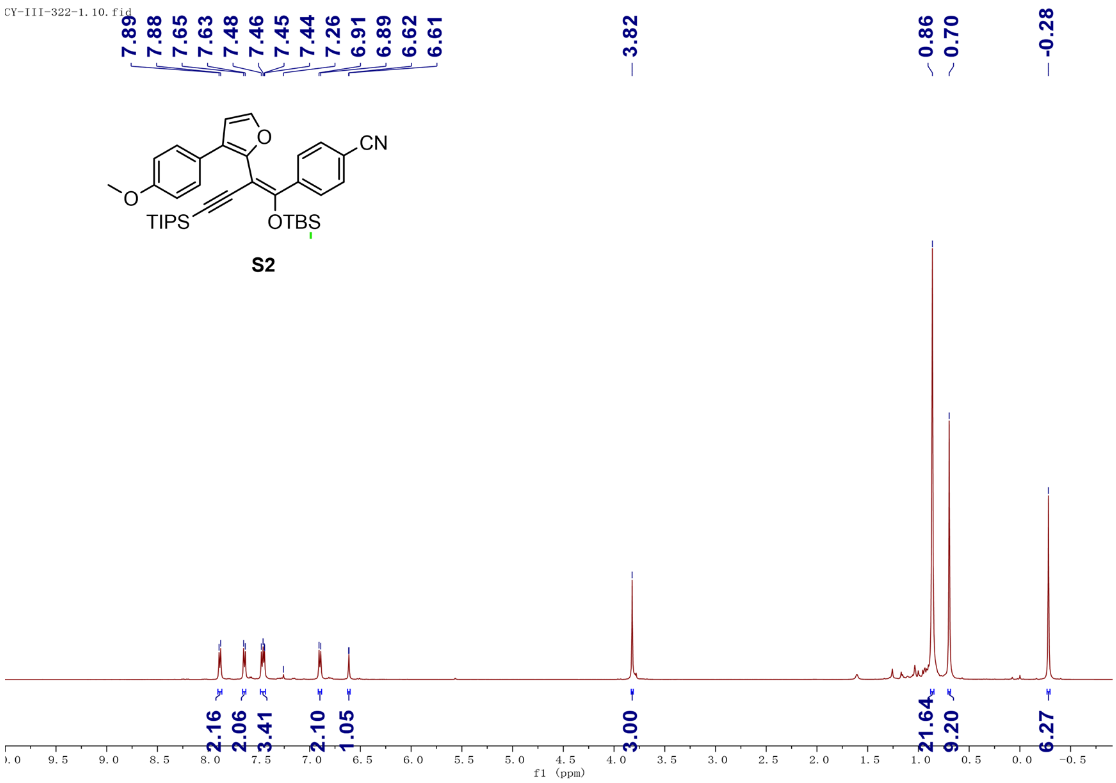

Supplementary Figure 290. ^{1}H NMR (500 MHz, $CDCl_3$) spectra for compound **S2**

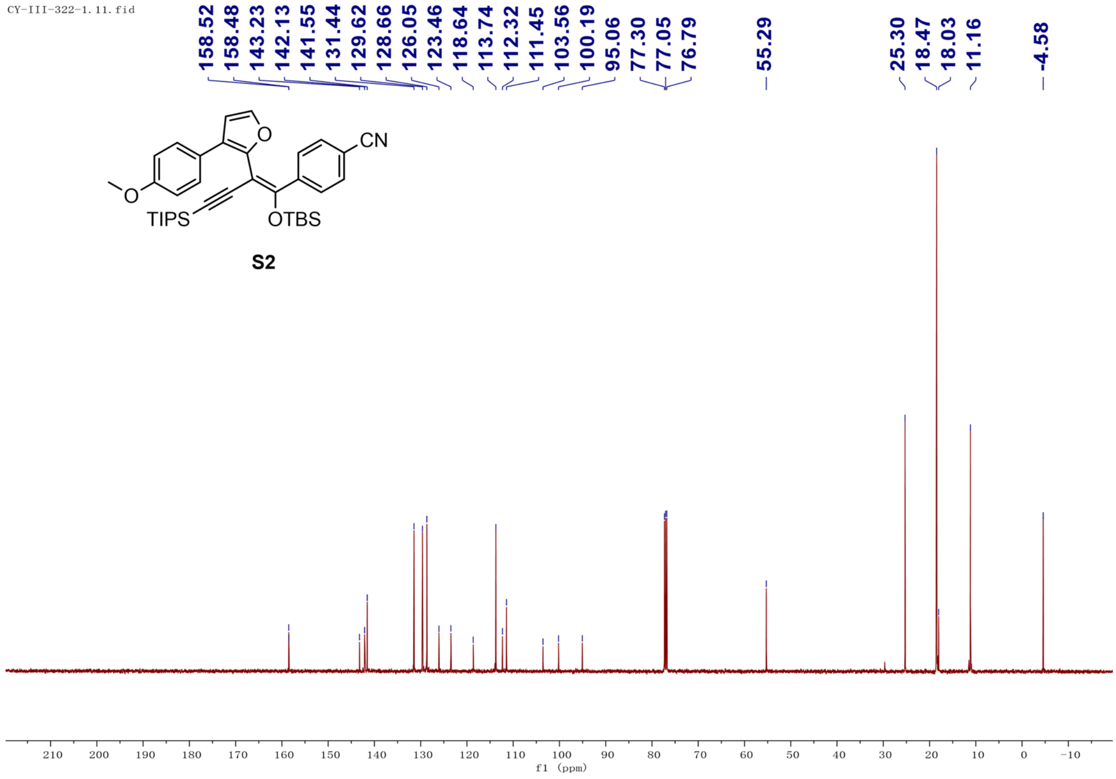

Supplementary Figure 291. ^{13}C NMR (126 MHz, $CDCl_3$) spectra for compound **S2**

IV. Supplementary References

[1] Tang, J. M.; Bhunia, S.; Abu Sohel, S. M.; Lin, M. Y.; Liao, H. Y.; Datta, S.; Das, A.; Liu, R. S, *J. Am. Chem. Soc.* **2007**, *129*, 15677; Hsu, Y. C.; Hsieh, S. A.; Li, P. H.; Liu, R. S, *Chem. Commun.* **2018**, *54*, 2114; d) Watson, I. D. G.; Ritter, S.; Toste, F. D. *J. Am. Chem. Soc.* **2009**, *131*, 2056; e) Tharra, P.; Baire, B, *J. Org. Chem.* **2015**, *80*, 8314.

[2] Wang, Y. H.; Liu, H.; Zhu, L. L.; Li, X. X.; Chen, Z. L. *Adv. Synth.Catal.* **2011**, *353,* 707. b) Vyas, V. K.; Knighton, R. C.; Bhanage, B. M.; Wills, M, *Org. Lett.* **2018**, *20,* 975.

[3] Li, Y.; Wei, H.; Wu, D.; Li, Z.; Wang, W.; Yin, G, *ACS Catal.* **2020**, *10*, 4888; b) Zhu, S.; Huang, H.; Zhang, Z.; Ma, T.; Jiang, H, *J. Org. Chem.* **2014**, *79*, 6113.

[4] Prenzel, D.; Sander, T.; Gebhardt, J.; Soni, H.; Hampel, F.; Gorling, A.; Maier, S.; Tykwinski, R. R, *Chem. Eur. J.* **2017**, *23,* 1846.

[5] Gidron, O.; Diskin-Posner, Y.; Bendikov, M, *J. Am. Chem. Soc.* **2010**, *132,* 2148.

[6] Ismail, M. A. *J. Chem. Res-s.* **2006**, *2006*, 733.

[7] Fallon, T.; Willis, A. C.; Rae, A. D.; Paddon-Row, M. N.; Sherburn, M. S, *Chem. Sci.* **2012**, *3*, 2133.

[8] Li, N. N.; Zhang, Y. L.; Mao, S.; Gao, Y. R.; Guo, D. D.; Wang, Y. Q, *Org. Lett.* **2014**, *16*, 2732.

[9] Wang, T.; Shi, S.; Hansmann, M. M.; Rettenmeier, E.; Rudolph, M.; Hashmi, A. S. K, *Angew. Chem. Int. Ed.* **2014**, *53*, 3715.

[10] For 1,2-alkynyl shifts in semipinacol-like rearrangements, see: a) Marson, C. M.; Walker, A. J.; Pickering, J.; Hobson, A. D.; Wrigglesworth, R.; Edge, S. J, *J. Org. Chem.* **1993**, *58*, 5944; b) Nagasawa, T.; Taya, K.; Kitamura, M.; Suzuki, K, *J. Am. Chem. Soc.* **1996**, *118*, 8949; c) Saito, T.; Suzuki, T.; Morimoto, M.; Akiyama, C.; Ochiai, T.; Takeuchi, K.; Matsumoto, T.; Suzuki, K, *J. Am. Chem. Soc.* **1998**, *120*, 11633.

[11] a) Danheiser, R. L.; Carini, D. J.; Basak, A, *J. Am. Chem. Soc.* **1981**, *103*, 1604; b) Wierschke, S. G.; Chandrasekhar, J.; Jorgensen, W. L, *J. Am. Chem. Soc.* **1985**, *107*, 1496.

[12] a) Dudnik, A. S.; Xia, Y.; Li, Y.; Gevorgyan, V, *J. Am. Chem. Soc.* **2010**, *132*, 7645.

[13] Frisch, M. J.; Trucks, G. W.; Schlegel, H. B.; Scuseria, G. E.; Robb, M. A.; Cheeseman, J. R.; Scalmani, G.; Barone, V.; Mennucci, B.; Petersson, G. A.; Nakatsuji, H.; Caricato, M.; Li, X.; Hratchian, H. P.; Izmaylov, A. F.; Bloino, J.; Zheng, G.; Sonnenberg, J. L.; Hada, M.; Ehara, M.; Toyota, K.; Fukuda, R.; Hasegawa, J.; Ishida, M.; Nakajima, T.; Honda, Y.; Kitao, O.; Nakai, H.; Vreven, T.; J. A. Montgomery, J.; Peralta, J. E.; Ogliaro, F.; Bearpark, M. J.; Heyd, J.; Brothers, E. N.; Kudin, K. N.; Staroverov, V. N.; Kobayashi, R.; J. Normand, K. R.; Rendell, A. P.; Burant, J. C.; Iyengar, S. S.; Tomasi, J.; Cossi, M.; Rega, N.; Millam, N. J.; Klene, M.; Knox, J. E.; Cross, J. B.; Bakken, V.; Adamo, C.; Jaramillo, J.; Gomperts, R.; Stratmann, R. E.; Yazyev, O.; Austin, A. J.; Cammi, R.; Pomelli, C.; Ochterski, J. W.; Martin, R. L.; Morokuma, K.; Zakrzewski, V. G.; Voth, G. A.; Salvador, P.; Dannenberg, J. J.; Dapprich, S.; Daniels, A. D.; Farkas, O.; Foresman, J. B.; Ortiz, J. V.; Cioslowski, J.; Fox, D. J. *Gaussian, Inc., Wallingford, CT, USA,* **2009**.

[14] Becke, A. D. *J. Chem. Phys.* **1993**, *98*, 5648.

[15] Lee, C.; Yang, W.; Parr, R. G. *Physical review. B, Condensed matter* **1988**, *37*, 785.

[16] Stephens, P. J.; Devlin, F. J.; Chabalowski, C. F.; Frisch, M. J. *J. Phys Chem.* **1994**, *98*, 11623.

[17] Krishnan, R.; Binkley, J. S.; Seeger, R.; Pople, J. A. *J. Chem. Phys.* **1980**, *72*, 650.

[18] Weigend, F.; Ahlrichs, R. *Phys. Chem. Chem. Phys.,* **2005**, *7*, 3297.

[19] Yanai, T.; Tew, D. P.; Handy, N. C. *Chem. Phys. Lett.* **2004**, *393*, 51.

[20] Lu, T.; Chen, F. *J. Comput. Chem.* **2012**, *33*, 580.
